# Constructing Physical and Genomic Maps for *Puccinia striiformis* f. sp. *tritici*, the Wheat Stripe Rust Pathogen, by Comparing Its EST Sequences to the Genomic Sequence of *P. graminis* f. sp. *tritici*, the Wheat Stem Rust Pathogen

**DOI:** 10.1155/2009/302620

**Published:** 2010-02-11

**Authors:** Jinbiao Ma, Xianming Chen, Meinan Wang, Zhensheng Kang

**Affiliations:** ^1^College of Life Sciences, Northwest A&F University, Yangling, Shaanxi 712100, China; ^2^Department of Plant Pathology, Washington State University, Pullman, WA 99164-6430, USA; ^3^USDA-ARS, Wheat Genetics Quality, Physiology, and Disease Research Unit, Pullman, WA 99164-6430, USA; ^4^College of Plant Protection, Northwest A&F University, Yangling, Shaanxi 712100, China

## Abstract

The wheat stripe rust fungus, *Puccinia striiformis* f. sp. *tritici* (*Pst*), does not have a known alternate host for sexual reproduction, which makes it impossible to study gene linkages through classic genetic and molecular mapping approaches. In this study, we compared 4,219 *Pst* expression sequence tags (ESTs) to the genomic sequence of *P. graminis* f. sp. *tritici* (*Pgt*), the wheat stem rust fungus, using BLAST searches. The percentages of homologous genes varied greatly among different *Pst* libraries with 54.51%, 51.21%, and 13.61% for the urediniospore, germinated urediniospore, and haustorial libraries, respectively, with an average of 33.92%. The 1,432 *Pst* genes with significant homology with *Pgt* sequences were grouped into physical groups corresponding to 237 *Pgt* supercontigs. The physical relationship was demonstrated by 12 pairs (57%), out of 21 selected *Pst* gene pairs, through PCR screening of a *Pst* BAC library. The results indicate that the *Pgt* genome sequence is useful in constructing *Pst* physical maps.

## 1. Introduction

*Puccinia striiformis* f. sp. *tritici* (*Pst*) is the causal agent of stripe rust, one of the most important diseases on wheat in many countries of the world [1, 2]. The disease is a major constraint to wheat production and is a serious threat to the global food security. Although the disease is economically important, only limited studies on the genome and functional genomics of the fungal pathogen have been reported [3–6]. This is an obstacle to our understanding of the pathogen's evolution, especially changes of virulence that often overcome resistance in wheat cultivars [1, 2, 7, 8]. 

*Pst* is an obligate biotrophic fungus that completely depends upon its host plants for continuing growth and reproduction. Techniques for transformation, gene knockout, and transient expression are still to be developed. This excludes the use of molecular techniques, such as restriction enzyme-mediated insertional mutagenesis and gene transformation. Unlike *P. graminis* f. sp. *tritici* (*Pgt*, the wheat stem rust pathogen) and *P. triticina* (*Pt*, the wheat leaf rust pathogen), *Pst* is a microcyclic rust fungus and has only three spore stages, urediniospore, teliospore, and basidiospore, and does not have known pycniospore and aeciospore stages [1, 2]. Because of the lack of the pycnial sexual stage and alternate host for sexual reproduction, it is impossible to study *Pst* genes through a classic genetic approach and map-based cloning. Thus, gene organization and physical relationships could not be studied for *Pst* using the molecular mapping approach. 

A physical map is useful for studying genome structures, determining gene organization, identifying important genes, and comparing related species for understanding evolutionary relationships. The discovery of conserved chromosomal segments between humans and animals in 1984 [9] led later to the construction of physical maps for human and mouse [10–13]. Interestingly, comparative gene mapping reveals that chicken, a nonmammalian vertebrate, has conserved genome sequence synteny with humans [14, 15]. Comparative genomic approaches have also been widely used to study related species in plants [16–19] and fungi [20–23]. These studies demonstrate that comparative genomic analysis is a powerful approach for studying genomes and genes in organisms that are hard to study using traditional genetic approaches. 

 Recently, several genetic libraries for *Pst* have become available, including a BAC library [3], a full-length cDNA library from urediniospores [4], germinated urediniospore or germ-tube EST library [5], and a haustorial EST library [6]. A total of more than 15,000 ESTs were sequenced, from which 4,219 unisequences were characterized and their putative functions were identified through sequence comparison with other fungal genes in GenBank databases. However, the physical and genetic relationships of these genes have not been determined. Since *Pst* genome sequencing has just been started, here we have used the available *Pgt* genome sequence (http://www.broadinstitute.org/annotation/genome/puccinia_group/MultiHome.html) for constructing physical maps for *Pst* genes. The study was based on the assumption that *Pst* and *Pgt* share considerable sequence homology and genome synteny. The specific objectives of this study were to (1) determine the homology of *Pst* EST unisequences to *Pgt* genomic sequences, (2) construct physical groups for the *Pst* genes using the *Pgt* sequences as the references, and (3) verify the physical relationships of selected *Pst* genes using PCR screening of the *Pst* BAC library. Although much of the physical relationship needs to be verified by whole-genome sequence, the physical maps generated in this study should provide a basic framework for assisting *Pst* sequence assembling and gene annotation with *Pgt* sequences and also should be useful for localizing functional genes, positional cloning of full-length genes, and generating information about exons and introns for *Pst* genes.

## 2. Materials and Methods

### 2.1. Data

 Genome-based EST mapping requires the genome map and transcript sequences. The three *Pst* cDNA libraries were generated from three different growing stages, urediniospores (Ured), germinated urediniospores (GermUred)/germ tubes, and haustoria (Haus). The Ured and Haus cDNA libraries were constructed from mRNA of PST-78, a typical US race [4, 6], and the GermUred library was from mRNA of CYR32, a typical Chinese race [5]. A total of 4,219 unisequences, which were obtained from more than 15,000 clones sequenced from the three libraries after removing sequences of poor quality (<100 bp inserts) and repetitions and forming contigs (4, 5, 6, Chen and associates, unpublished), were used in this study for comparing with the *Pgt* genomic sequence. The *Pgt* genome sequence was downloaded from the NCBI Genome Project *Puccinia graminis* Database (http://www.broad.mit.edu/annotation/genome/puccinia_graminis), consisting of 392 genome supercontigs and 4,775 contigs.

### 2.2. Mapping Pst EST Sequences against the Pgt Genome Sequence

 All *Pst* ESTs were mapped against the *Pgt* genome using the BLASTN program [24]. We used the high-speed service computer system of the Washington State University Bioinformatics Center for BLAST and homology searches. The *Pgt* genome and *Pst* EST sequences were transferred to a server computer using the SSH (Secure Shell) software as fasta format files. Sequences of low homologous alignment were filtered out using the e value of 1.00E-5 as a cut point. The alignable ESTs were assembled according to the 4,775 contigs in the 392 supercontigs of the *Pgt* genome sequence. Detailed alignment information was edited in an Excel file. To see the positions of the *Pst* ESTs corresponding to the *Pgt* genome, physical maps were constructed. Physical maps corresponding to *Pgt* supercontigs illustrated the physical position order of the genes, length of each EST, and the distances between genes. The genes localized in a single contig were marked using a sign of “*∣*” and the alignment start and end positions of the *Pgt* genome were given in parentheses. 

 Because the ESTs were transcribed from the genome and the introns were spliced after alternative splicing, the ESTs represent the exon sequences. Therefore, it was important that we were able to get the information about the alternative gene splicing and the intron number from the maps. If a *Pst* EST sequence was aligned to a location in the *Pgt* genome as a series of fragments, these genes were likely to show alternative splicing, and the number of exons was marked after the parentheses on the map. All sketch maps of *Pst* genes are shown in file 1 in Supplementary Material available online at doi: 10.1155/2009/302620.

### 2.3. Verification of Physical Relationships of Selected Pst Genes

 Although *Pgt* is most closely related to *Pst* among the fungi whose whole genome has been sequenced so far, their gene sequences and locations could be different for some genes. To validate the veracity of the alignment, we selected 42 genes as 21 pairs. The sequences of the 42 genes were used to design primers. The 42 primer pairs (Table 1) were used to amplify BAC clones. If a single BAC clone was amplified by primers of both genes in a pair, the two genes were concluded to be physically colocated. Because the BAC library has an average insert size of 50 Kb [4], the two genes in each pair were selected based on their distance in between smaller than 50 Kb. For each pair of genes, the primers for one of the genes were used to amplify the entire BAC library of 43,000 clones [3] using a three-dimensional approach as described by Ling and Chen [25]. To be more efficient, the primers for the second gene in the pair were used to amplify only the positive BAC clones from the screening. To speed up the PCR screening, two pairs of primers for two genes with similar annealing temperatures were used in a multiplex PCR amplification. 

 Multiplex PCR was performed in a GeneAmp PCR System 9700 thermo-cycler. A 20 *μ*L reaction mixture contained 1.0 *μ*L (30 ng/*μ*L) of a BAC clone DNA, 4.0 *μ*L Mg-free 5X PCR buffer (Promega, Madison, WI, USA), 0.1 *μ*L of 5 unit Taq DNA polymerase (Promega), 2 *μ*L of 25 mM MgCl_2_, 0.5 *μ*L of 2.5 mM dNTP (dATP, dCTP, dGTP and dTTP) (Sigma Chemical Co., St. Louis, MO, USA), and 1.0 *μ*L of 10 mM each primer synthesized by Operon Biotechnologies, Inc. (Huntsville, AL, USA). After 2 minutes of denaturation at 95°C, amplifications were programmed for 35 cycles, each consisting of 30 seconds at 95°C, 30 seconds at 45.9–59.4°C depending upon primer pairs shown in Table 1, 40 seconds at 72°C, and followed by a 10-minute extension step at 72°C. After PCR amplification, 5 *μ*L of the solution for each sample was electrophoresed in a 1.5% agarose gel in 05x TBE buffer (0.089 M Tris-borate, 0.089 M boric acid and 0.002 M EDTA). The 100 bp plus DNA ladder (Fermatas, Glen Burnie, MD, USA) was used to estimate the size of each amplified DNA fragment. The gel was run for 90 minutes at 100 volts, stained with ethidium bromide (0.5 *μ*g/mL) for 30 minutes, and photographed under ultraviolet light. The genomic DNA of *Pst* race PST-78 was used as positive control and autoclaved dd H_2_O was used as a negative control in the PCR amplification.

## 3. Results

### 3.1. Homology of Pst ESTs and Pgt Genomic Sequences

 Of the 4,219 *Pst* unisequences from the Ured, GermUred and Haus libraries were searched for homologous sequences in the *Pgt* genome, 1,432 had significant homology (e value < 1.00E-5) to *Pgt* genomic sequences. As shown in Table 2, the three libraries had different percentages of homologous genes with *Pgt*. The Ured library had the highest percentage, 54.51%, followed by the GermUred library (51.21%), while the Haus library had the lowest percentage (13.64%). In average, 33.94% of the 1,432 *Pst* genes had significant homology with the *Pgt* sequences. 

### 3.2. Physical Groups

**Figure 1:**
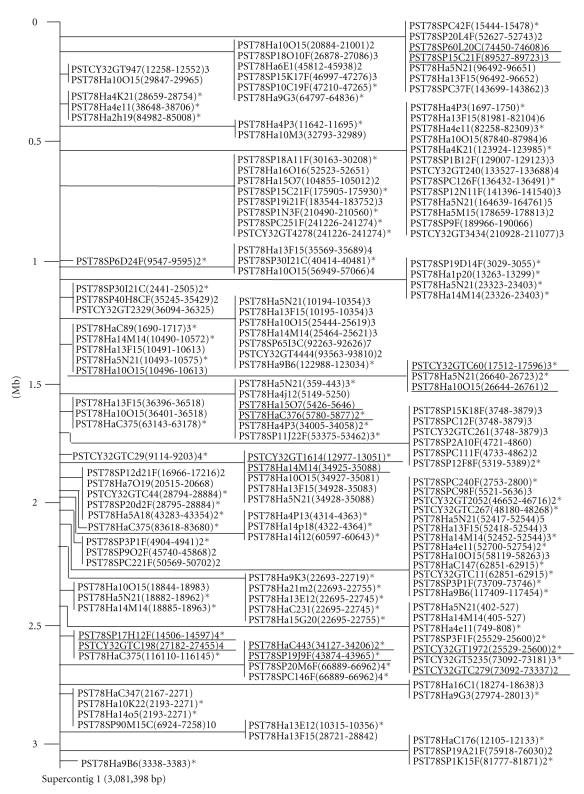
An example of physical maps for *Pst* ESTs based on corresponding sequence positions of homologous genes of *Pgt*. All 242 physical groups are presented in Supplementary file 1. The distance in mega base (Mb) is shown on the left. The clones in a group indicated by a vertical line are in the same contig and the start and end positions of the sequence matching the positions in the contig are shown in the “( )” following the clone identification number. The number after the “( )” indicates the number of the gene with multiple positions in the *Pgt* genome. An asterisk indicates that the number of matching base pairs is smaller than 100. The clones underlined were used in PCR amplification of the *Pst* BAC library.

**Figure 2:**
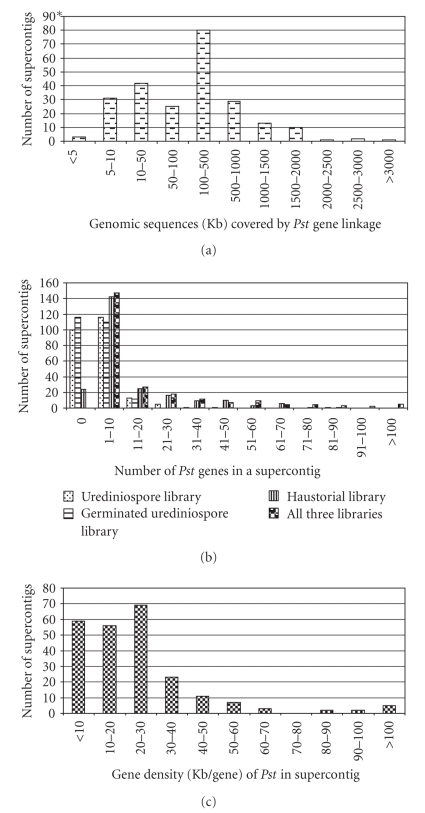
*Characterization of Pst physical groups corresponding to Pgt supercontigs*. (a) Frequencies of genomic sequence sizes that were covered by *Pst* gene physical groups. (b) Frequencies of number of *Pst* genes in a physical group corresponding to a *Pgt* supercontig in the three *Pst* cDNA libraries and the total. (c) Gene densities (Kb/gene) in the *Pst* physical groups corresponding to *Pgt* supercontigs.

 The 1,432 *Pst* genes were aligned to 237 physical groups corresponding to 237 *Pgt* supercontigs (Supplementary file 1). As an example, Figure 1 shows *Pst* genes aligned to *Pgt* supercontig 1. The number of genes for each supercontig from each *Pst* cDNA library is shown in Table 3. The 237 physical groups ranged from 2,878 to 3,081,398 bp with most of the groups ranging from 5.0 Kb to 2.0 Mb (Figure 2(a)). Overall, the 1,432 genes matched 787,413 bp and spanned over 86.55 Mb of the *Pgt* genomic sequences. Because the majority of the 1,432 unigenes were aligned to more than one sequence locus, a total of 4,604 gene loci were obtained (Table 3). The fold of multiple loci per unique gene was unbalanced among the three libraries with 1.30 for the GermUred library, 1.53 for the Ured library, and 10.58 for the Haus library. 

 The number of genes varied from 1 to 153, excluding “Supercontig 392”, which contained unassembled sequences, with an average of 19 genes per supercontig (Table 3, Figure 2(b)). Over 70% of supercontigs contained 20 or fewer genes that showed homology to *Pst *EST sequences while only 4 supercontigs (Supercontigs 1, 2, 3, and 17) had more than 100 genes. The genes from the three *Pst* libraries were unevenly aligned to the *Pgt* genome. A total of 712 unisequences were aligned to 134 supercontigs with an average of 5.3 genes per supercontig; 441 unisequences were aligned to 121 supercontigs with an average of 3.6 genes per supercontig; the 279 supercontigs were aligned to 213 supercontigs with an average of 1.3 genes per supercontig. The gene density (the number of base pairs per gene) ranged from 1,020 to 209,493 bp with an average of 18,799 bp (Table 3, Figure 2(c)). The majority of the supercontigs had a gene in a genomic region smaller than 30 Kb, which may be considered to be a relatively gene-rich region. In contrast, a few supercontigs had a gene in genome region larger than 60-Kb, which may be considered as relatively gene-poor region. These results indicated that genes expressed in different *Pst* growth stages tended to be clustered in different regions of the genome.

### 3.3. Exons of Pst Genes Revealed by Comparison with Pgt Genomic Sequences

 Of the 1,432 *Pst* genes, 911 (63.62%) had more than one exon and the remaining 521 (36.38%) had one exon. Of the 911 genes with multiple exons, 570 (62.57%) had two, 200 (21.95%) had three, 97 (10.65%) had four, 25 (2.74%) had five, 13 (1.43%) had six, 3 (0.33%) had seven, 2 (0.22%) had eight, and 1 (0.11%) had nine exons. The different numbers of exons indicate the different levels of complexity of the genes, which might reflect their variability resulting from the evolutionary process. 

### 3.4. Validation of Physical Relationships of Selected Pst Genes

**Figure 3:**
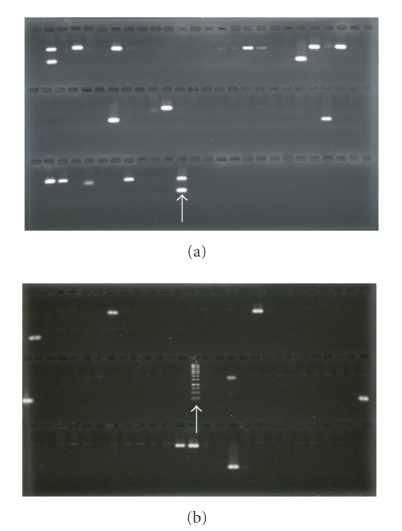
*Agarose gels showing positive amplification of the Pst BAC clones using multiplex PCR with primer pairs PSTCY32GT1071F/R (upper bands) and PST78SP3H2F/R (lower bands)*. (a) Amplification of 58 BAC plate pools to identify positive pools. (b) Amplification of row and column pools of a positive plate to identify individual positive clones. The arrow in (a) indicates the bands amplified with *Pst* genomic DNA and in (b) indicates molecular size marker.

 To validate the physical relationships of *Pst* genes, a total of 84 forward and reverse primers were designed for 42 genes to form 21 pairs (Table 1). The genes in each pair were selected based on their proximity within 50 Kb in the physic map. Clones that were positively amplified with the first pair of primers resulted from the three-dimensional pooling screening were amplified with the second pair of primers, as illustrated in Figure 3. Of the 21 pairs of genes tested, 12 pairs (57%) were successfully identified in same BAC clones. The results clearly showed that these genes in pairs were truly colocated in the *Pst* genome.

## 4. Discussion

Before the *Pst* genome is completely sequenced, which is under way, it is almost impossible to study genetic and physical relationships among genes of this obligate biotrophic fungus without sexual reproduction [2]. In this study, we explored the possibility to use the whole genome sequence of *Pgt*, the most closely related fungus sequenced so far, as a reference to construct physical maps for *Pst* genes. From a total of 4,219 unique genes, we identified 1,432 genes significantly homologous to sequences in the *Pgt* genome. Because of their high nucleotide identities to the *Pgt* genome sequences, we assumed that these genes should have high levels of synteny to the corresponding genes in the *Pgt* genome. Thus, using the *Pgt* genomic sequences, we grouped the 1,432 *Pst* unique genes with a total of 4,604 genomic loci into 237 physical groups corresponding to *Pgt* supercontigs. The proximity physical relationship was demonstrated for 12 pairs of genes using our *Pst *BAC library [3]. This study is the first to report the physical relationships for *Pst* genes and is the first to use the whole-genome sequence of a fungal species to study physical relationships of genes in a related species among the cereal rust fungi. 

 The homologous genes did not show an even distribution on the *Pgt* genome because no homologous *Pst* genes were found on 145 of a total of 382 *Pgt* contigs and gene densities varied greatly from 1,020 bp to 209,493 bp. Such an uneven distribution may be partially due to the different sizes of the *Pgt* supercontigs. The uneven distribution also could be caused by the relatively small number of genes. The 1,432 *Pst* genes are only about 8% of the total estimated number of genes based on the over 20,000 genes of *Pgt*. It also is possible that the genes expressed in each of the three developmental stages may cluster on certain genome regions. Nevertheless, the data may indicate the existence of gene-rich and gene-poor regions in the *Pst* and *Pgt* genomes. The information of gene-rich regions and *Pst*/*Pgt* homologous gene-rich regions will be useful in understanding the evolutionary relationships of the two related but different rust fungi. This hypothesis would be more clearly tested by comparing all *Pst* genes after the completion of the whole-genome sequencing and sequencing of more ESTs, which are currently being undertaken. 

 In this study, we only tested 42 *Pst* genes in 21 pairs in the PCR screening of the BAC library. In contrast to the 12 pairs that were demonstrated in the same BAC clones, positive results were not obtained for 9 of the 21 pairs. However, the unsuccessful amplification by the second genes in the 9 pairs does not exclude the possibility of physical relationships for the genes in each of these pairs. As the inserts of the BAC clones were relatively short, 50 Kb in average [3], the clones might be too small to harbor both genes in a pair. It is also possible that the *Pst* genes in each pair may have a longer distance than the reference distance in the *Pgt* genome, but they may still be linked to each other. 

 The *Pst* genes used in this study were from three libraries. The genes from the Ured library gave the highest percentage of genes homologous to *Pgt* and the genes from the Haus library gave the lowest percentage of homologous genes. The GermUred clones had similar percentage of *Pgt*-homologous genes to the Ured library, although the two libraries were made from different isolates while the Haus library was made with the same isolate as the Ured library [4–6]. The low proportion of the *Pst* genes from the Haus library similar to the *Pgt* sequences was surprising as we thought that two fungal species in the same genus should have higher homology than human and mouse that are in very different taxa [9]. Although this phenomenon needs more studies, we have learned from other rust fungi that genes expressed in haustoria tend to be more species specific [26, 27]. Comparisons of *Pst* genes expressed in different growth stages with the *Pgt* sequences tell us that genes expressed in urediniospore are more conserved among different *Puccinia* species while those expressed in haustoria are more unique. Such genetic differences may be related to their different requirements in temperature for infection of the same wheat host crop. 

 It is interesting that the smallest number of unique genes (279) from the Haus library produced the highest number (2,952) of genomic loci along the *Pgt* genome among the three libraries. The high fold (10.58x) of gene copies may compensate for the low number of homologous genes from haustoria, which may make the overall homology of *Pst* and *Pgt* genome sequences reasonably high. The genomic loci were aligned to more supercontigs than the genes from the Ured and GermUred libraries. These results indicate that haustorially expressed genes tend to have multiple copies and spread along the *Puccinia* genome. This phenomenon needs to be further studied using the whole genome sequence of *Pst*. 

 Although much of the physical relationship is still hypothetical and needs to be verified by the whole genome sequence of *Pst*, the physical groups constructed in this study can serve as references and starting points in assisting sequence assembling and gene annotation. A more detailed dissection of gene sequences, organization, structures, and clusters may allow us to pick genome regions and gene clusters to study their functions and developing molecular markers to tag virulence groups and characterize *Pst* populations. 

 In this study, we found that some ESTs could be matched to more than one location. Also, an alignment consisted of multiple exons while others do not have introns. We included the intronless sequences in the physical maps. Intronless sequences as pseudogenes have coincident nucleotide sequences with coding protein genes ubiquitously existing in the eukaryotes genome [28, 29]. Although pseudogenes may be functionless DNA fragments in the genome, they have evolved from mRNA reverse transcription and then reset in the genome. So, pseudogenes do not have introns and promoters but have poly(A) sequences. For a full-scale gene mapping, it represents the real gene transcription and sequence existence. Most of our EST sequences are not full-length and only have partial information of genes. This might be an explanation why a considerable number of ESTs were aligned to regions of the *Pgt* genome without introns. 

 We found that many of the *Pst* ESTs that matched to *Pgt* genomic sequences were shorter than 100 bp. These short sequences may be exons, whose lengths can vary greatly. Most vertebrate exons are between 50 and 400 bp long [30]. Using the complementary sequence feature method in humans, *Arabidopsis*, *Cryptococcus,* and *Plasmodium*, Saeys et al. [31] reported that one-third of all exons were smaller than 100 bp. Gudlaugsdottir et al. [32] reported significant variation in exon length for human and fission yeast ranging from 1 to thousands of base pairs. Because exon sizes can vary from a few base pairs to thousands of base pairs, we reserved even the segments smaller than 50 base pairs, which may have saved some unknown information in alignment and make the information available for the future *Pst* genome research. The number of exons in a gene may indicate its stability or variability, which may allow us to choose genes for studying various aspects of pathogen biology. Genes with only one exon may be chosen to study the genetic relationships at a higher taxonomic level, such as species and formae speciales, and those with multiple exons may be used to study genetic differences among isolates within a forma specialis. Genes with multiple exons may be better candidates for studying traits like virulence and adaptation to different environments as these traits have more variations. 

 In this study, we produced preliminary physical maps for *Pst *genes. The 4,604 genomic loci of 1,432 genes were placed on the physical map account about 8% of potential genes, if we assume that *Pst* and *Pgt* have a similar number of genes. Because we used only unique genes, some genes belonging to large families could be located on multiple genome sites. In the future, this physical map will be verified and ultimately be improved by the complete set of the *Pst* genes and connected with nontranscribed sequences. The physical groups should provide insights into gene organization, identification of functionally related genes, positional cloning of full-length genes, information on exons and introns, and assist in sequence assembly and gene annotation for the *Pst* whole-genome sequencing.

## Supplementary Material

Physical maps for Pst ESTs based on corresponding sequence positions of homologous genes of Pgt. A total of 242 physical groups are constructed. The distance in mega base (Mb) is shown on the left. The clones in a group indicated by a vertical line are in the same contig and the start and end positions of the sequence matching the positions in the contig are shown in the “( )” following the clone identification number. The number after the “( )” indicates the number of the gene with multiple positions in the Pgt genome. An asterisk indicate that the number of matching base pairs is smaller than 100. The clones underlined were used in PCR amplification of the Pst BAC library.

## Figures and Tables

**Table 1:** *Primer sequences of Pst genes and amplification of same BAC clones*. The table shows sequences and annealing temperatures (Tm) of primers based on *Pst* EST sequences used in PCR screening the *Pst* BAC library to determine the physical relationships of genes in pairs identified through BLAST search comparison with the *Pgt* whole genome sequence. The presence and absence of both genes of a pair in the same BAC clone are indicated by “Y” and “N”, respectively.

Gene pair	EST clone	Forward (5′ − 3′)	Reverse (5′ − 3′)	Tm (°C)	In same BAC clone
1	PST78SP60L20C	CTGGTAATGGAGGTGGAACT	CTGGGGTAGGTAAGAAGGTC	53.8/53.1	Y
	PST78SP15C21F	AAGCCCCTCCAGTAGAAC	CACATCCCAGACGGTAAAT	51.0/52.6	

2	PST78Ha6E1	GACTGGTGTCATTGCTGAA	TGGAGGAAGATACTTGGAGA	51.2/51.8	N
	PST78SP15K17F	CCGAAATACCCCAGAACT	GTCAACGATGAACAGAAGAG	52.0/49.0	

3	PST78HaC443	CCTCGTCTTCACCTTCATTA	TGTTTAGTTCTTGTCAGCGT	52.6/50.7	Y
	PST78SP19J9F	AGAATCAGCACGAAAGGG	TTGAGGTAGGTTGGACGG	52.0/53.0	

4	PSTCY32GTC198	ACATTATTCGCTTCCCTTTC	TGAGTTTTGTGGTATCGGTC	54.0/53.0	Y
	PST78SP17H12F	CACAACTACCAGACCCAG	CGTAGGAAGTCGGATAAG	47.0/47.0	

5	PST78SP65I3C	CAGGAGGAAAACAACCATAA	ATCGTACTAAGCACCCATCA	52.8/52.9	N
	PST78Ha9B6	GTTTGATTGAGCGGGATTC	CCCATTCATCTGCCTGTTT	55.0/55.0	

6	PSTCY32GTC60	GGAACTACCAGGACTACCC	CACCCATACTTCTGAGGC	50.5/50.0	Y
	PST78Ha10O15	GGGTAGTGCTCCCAAAC	TTCTGCCGTCAAAGTGT	49.7/48.6	

7	PST78HaC375	CAGTCGTCCTCAAAATCCTA	TCCCCAGCACTATTCCTTAT	52.6/54.1	N
	PST78Ha10O15	GGGTAGTGCTCCCAAAC	TTCTGCCGTCAAAGTGT	49.7/48.6	

8	PSTCY32GT1614	ACACGTAAGGACAGCAGAA	GGATAACAAGGAAGGGAGA	50.7/51.2	Y
	PST78Ha14M14	TCAGAAACAAGACCCACC	CCCACTTCACTACCCATTA	50.0/50.3	

9	PST78SP6D14F	GAATCCACTCCATCCCACT	TACGGCTCCGAGAACGAC	53.7/56.4	N
	PST78SP14N13F	AACACCAGCAGCACAACT	TGTAGCCCTAACCTTCGT	50.8/50.1	

10	PSTCY32GTC331	CGTCCTTGGCTGAATCTC	CGGCTACACCACGAACAT	53.0/54.3	N
	PST78SP20d10F	ACACCAGCATCGCAAAAC	GAACGAGCGTGAGGGAGA	54.4/56.1	

11	PSTCY32GTC164	TGCCAGTCCGAGTATCAAGA	GTAGCAGATTCCGAGTCCAA	56.2/55.1	Y
	PST78SP11F24F	CGGAGGAACAGCTACAAAAG	GGAGAAGGGATAACCCAGAC	55.3/55.0	

12	PSTCY32GTC285	CAGCCTCACTAACAACATCG	TAATAGGACAGGAGCAGACA	53.8/50.3	N
	PST78SP18M12F	AACCCTGCCACAATGATGAC	GGACGGGGAAACAATAGAGC	57.7/58.7	

13	PSTCY32GT429	GGCTGCTGAATATGACCGAA	GCCTGCCACATCACTACCTG	58.5/58.5	N
	PST78SPC50F	GAGGCGTCTGGTGGGATAAG	CCGTAAAGAGGTTTCCGAGATGAT	59.4/63.1	

14	PSTCY32GTC220	CAACTGACACCGCTGAAA	CGCCTTCTTGGAATGACT	52.5/52.8	Y
	PST78SP66B11CF	ATGATGGCGGATAGAACA	GCTACCCGACCTCACTTT	51/51.9	

15	PST78SP10f11F	CCGCAGTCGCTGTATGTA	TGTATCCAACTTGCCCAC	53.0/51.3	Y
	PSTCY32GT407	ACGACTGCTTCTGCTTCA	ATCCTCGCCATTCTTCTT	51.3/51.5	

16	PST78SP3H2F	CGAAGACCAGCAAAATGT	CACGGAGATGGAAAGAAT	51.0/49.6	Y
	PST78Ha8F3	ACTTTACCAAGATGACCC	GTGAAGTAATCCCAAACC	45.9/46.5	

17	PSTCY32GT1071	ACCCTGGAAAAGGCGAAAT	GCGATGATGCCCGATGTA	58.7/57.9	Y
	PST78SPC194F	GACGCCAGTCGTAGCACA	GGGATTGAGGGACGCATA	55.3/56.0	

18	PST78SP65M2C	AATCTTATGTTCAAGTTCGGTT	TTCGTTTCTGTTAATACTCCTA	53.2/49.9	N
	PSTCY32GT5910	ACCAAACGAAAGAACAAG	TTCACTCTACCAACAGCA	47.6/45.9	

19	PSTCY32GT2267	AAGACCACCTCGCTCAAC	GGAAATACGTCCGCAAAT	52.3/53.2	N
	PSTCY32GT1090	CGACGACTACCACGACAT	ACGATAGCTTGCCATCAC	51.1/50.8	

20	PST78Ha1507	GCCAATCAAGGATGCTCT	GAAGTTCCGCCGTAGTGT	52.6/52.8	Y
	PST78HaC376	CCCTACTACGACCTCCA	CTGCTTCTACCCATCCA	47.3/48.9	

21	PSTCY32GTC279	CCAAACAACCAAACGACGAA	GACCGAAAGCGGGTGAATAG	59.3/59.7	Y
	PSTCY32GT1972	CTCAAGATACATCGTCCC	AAGTTGGTCAGGCAGTTC	46.9/49.5	

**Table 2:** *Homology of Pst and Pgt genes*. The total numbers of *Puccinia striiformis* f. sp. *tritici* (*Pst*) unigene ESTs from three cDNA libraries compared with and the numbers and percentages of ESTs with significant sequence homology with the whole genomic sequences of *P. graminis* f. sp. *tritici* (*Pgt*).

* Pst* cDNA library	No. of unigenes	No. of unigenes with Significant	Percentage (%) of unigenes with
		Homology to *Pgt* genes^a^	significant Homology to *Pgt* genes^a^
Urediniospores	1,306	712	54.51
Germinated urediniospores	869	441	51.21
Haustoria	2,044	279	13.65

Total	4,219	1,432	33.94

^a^ The *E* value of 1 × 10^−5^ was used as a cut point to determine significant homology.

**Table 3:** *Physical groups of Pst corresponding to Pgt supercontigs*. Size of each *Pgt* supercontig and *Pst* EST coverage in the supercontig, number of contigs, number of ESTs in the urediniospore (Ured), germinated urediniospore (GermUred) and haustoria (Haus) libraries, and total average size of aligned ESTs and *Pst* gene density in each *Pst *physical group/*Pgt* supercontig.

*Pst* physical		Size (bp)	No. of					Average	*Pst* gene
		covered	*Pst*	No. of *Pst* ESTs in each library and total				size (bp)	density in
group/*Pgt *	Size (bp)	By *Pst *	EST					of aligned	supercontig
supercontig	in *Pgt *	ESTs	contigs	Ured	GermUred	Haus	Total	*Pst* ESTs	(bp/gene)
1	3,081,398	29,571	35	48	18	87	153	193	20,140
2	2,570,998	18,205	39	30	15	70	115	158	22,357
3	2,616,274	16,097	38	21	20	67	108	149	24,225
4	1,978,325	9,744	33	18	7	61	86	113	23,004
5	2,008,477	15,513	32	25	15	58	98	158	20,495
6	1,808,965	14,784	30	13	10	64	87	170	20,793
7	1,797,936	8,626	25	10	11	32	53	163	33,923
8	1,737,638	10,749	30	4	11	60	75	143	23,169
9	1,714,174	14,977	25	12	13	46	71	211	24,143
10	1,640,743	14,619	25	33	19	42	94	156	17,455
11	1,547,344	12,529	26	12	7	66	85	147	18,204
12	1,543,397	8,518	20	8	4	42	54	158	28,581
13	1,556,540	8,681	23	7	10	36	53	164	29,369
14	1,510,324	12,100	26	16	11	46	73	166	20,689
15	1,374,611	15,098	24	16	10	53	71	213	19,361
16	1,217,956	12,940	22	11	10	49	70	185	17,399
17	1,242,959	18,124	28	25	9	74	109	166	11,403
18	1,195,459	5,965	16	10	6	28	44	136	27,170
19	1,137,327	7,582	13	6	3	49	58	131	19,609
20	1,198,131	9,437	24	9	5	42	56	169	21,395
21	1,084,580	7,679	17	8	6	49	63	122	17,216
22	1,051,806	5,445	17	6	9	13	28	194	37,565
23	1,003,138	10,246	10	8	8	29	45	228	22,292
24	1,008,357	9,373	18	13	8	34	55	170	18,334
25	1,068,291	9,855	18	17	10	34	61	162	17,513
26	1,006,249	10,628	19	8	6	45	59	180	17,055
27	1,005,714	8,119	15	7	5	36	48	169	20,952
28	964,966	6,986	14	10	13	20	43	162	22,441
29	999,150	4,693	16	5	5	24	34	138	29,387
30	919,905	7,064	10	14	7	22	43	164	21,393
31	986,084	6,076	13	7	3	27	37	164	26,651
32	894,979	7,030	16	7	6	40	53	133	16,886
33	889,308	108,494	3	17	8	20	45	2411	19,762
34	856,319	4,895	8	5	4	22	31	158	27,623
35	904,227	9,214	20	16	3	33	52	177	17,389
36	867,522	3,694	10	5	3	11	19	194	45,659
37	820,150	3,615	12	5	0	22	27	134	30,376
38	803,102	3,888	9	8	8	7	23	169	34,917
39	752,863	4,021	11	3	4	28	35	115	21,510
40	667,254	18,530	11	6	8	24	38	488	17,559
41	658,202	4,815	8	11	5	5	21	229	31,343
42	682,257	6,622	10	10	4	26	40	166	17,056
43	654,820	3,637	12	7	4	20	31	117	21,123
44	680,700	4,941	13	2	1	33	36	137	18,908
45	611,533	5,287	9	8	2	30	40	132	15,288
46	603,048	3,148	10	8	5	9	22	143	27,411
47	640,690	3,675	12	9	6	10	25	147	25,628
48	569,981	2,235	7	5	3	15	23	97	24,782
49	609,911	2,601	9	2	3	16	21	124	29,043
50	685,923	5,158	15	2	2	43	47	110	14,594
51	574,326	2,770	9	8	4	14	26	107	22,089
52	572,077	2,928	9	10	1	9	20	146	28,604
53	591,244	2,420	9	2	1	15	18	134	32,847
54	525,265	6,453	8	2	2	23	27	239	19,454
55	542,982	2,301	7	7	3	6	16	144	33,936
56	577,102	1,706	9	5	0	11	16	107	36,069
57	480,201	2,436	5	2	3	16	21	116	22,867
58	496,650	2,974	10	8	4	10	22	135	22,575
59	489,205	5,657	5	4	3	13	20	283	24,460
60	436,003	2,794	4	2	3	13	18	155	24,222
61	469,309	5,578	11	6	6	20	32	174	14,666
62	428,160	8,885	9	30	6	34	70	127	6,117
63	433,102	1,713	7	6	2	7	15	114	28,873
64	440,512	1,559	10	2	2	9	13	120	33,886
65	407,335	1,490	5	1	2	7	10	149	40,734
66	388,993	1,775	6	3	2	8	13	137	29,923
67	403,504	5,231	9	4	2	22	28	187	14,411
68	403,089	2,587	8	6	5	8	19	136	21,215
69	367,522	1,464	5	6	0	2	8	183	45,940
70	386,059	1,545	5	0	1	8	9	172	42,895
71	392,332	2,229	9	0	1	19	20	111	19,617
72	398,881	3,281	12	6	1	15	22	149	18,131
73	360,371	5,159	8	0	1	30	31	166	11,625
74	351,149	2,082	5	2	5	2	9	231	39,017
75	350,882	796	7	2	1	7	10	80	35,088
76	341,344	2,159	4	2	0	14	16	135	21,334
77	304,582	1,463	4	0	2	11	13	113	23,429
78	296,996	2,909	2	5	6	6	17	171	17,470
79	286,933	1,664	3	0	1	9	10	166	28,693
80	312,600	2,919	7	2	0	21	23	127	13,591
81	341,312	1,538	9	4	2	8	13	118	26,255
82	375,963	1,130	5	1	0	6	7	161	53,709
83	301,412	4,474	9	6	2	20	28	160	10,765
84	306,211	2,780	4	6	4	5	15	185	20,414
85	286,187	2,285	5	6	5	2	13	176	22,014
86	268,723	3,915	6	2	2	23	27	145	9,953
87	273,404	1,018	3	0	0	12	12	85	22,784
88	271,488	2,241	4	5	1	7	13	172	20,884
89	281,218	997	4	6	1	1	8	125	35,152
90	282,829	2,792	5	1	2	7	10	279	28,283
91	268,653	2,792	5	4	4	3	11	254	24,423
92	249,303	1,055	4	2	2	2	6	176	41,551
93	254,338	2,955	8	2	2	20	24	123	10,597
94	224,654	1,369	6	3	1	8	12	114	18,721
95	268,031	845	4	3	1	3	7	121	38,290
96	240,035	984	5	2	2	5	9	109	26,671
97	283,953	370	3	2	0	1	3	123	94,651
98	259,162	1,865	4	1	3	6	10	187	25,916
99	245,804	345	4	0	0	5	5	69	49,161
100	212,873	1,355	4	4	1	2	7	194	30,410
101	249,761	566	4	2	0	4	6	94	41,627
102	224,856	1,583	4	3	2	5	10	158	22,486
103	231,854	1,007	5	5	0	4	9	112	25,762
104	230,218	1,733	4	1	3	4	8	217	28,777
105	209,493	158	1	1	0	0	1	158	209,493
106	179,026	857	2	0	0	9	9	95	19,892
107	191,896	1,271	5	3	1	4	8	159	23,987
108	172,930	455	2	0	0	3	3	152	57,643
109	232,811	1,142	6	3	1	5	9	127	25,868
110	174,613	802	4	0	1	5	6	134	29,102
111	183,422	2,657	5	0	0	20	20	133	9,171
112	205,437	810	2	1	0	3	4	203	51,359
113	163,692	624	4	0	1	5	6	104	27,282
114	173,085	754	3	0	2	5	7	108	24,726
115	158,618	1,640	4	1	3	5	9	182	17,624
116	160,911	427	1	1	1	0	2	214	80,456
117	159,147	691	3	4	0	1	5	138	31,829
118	164,903	2,902	7	4	5	7	16	181	10,306
119	180,073	674	3	0	0	5	5	135	36,015
120	132,002	219	1	0	0	1	1	219	132,002
121	136,681	318	2	0	0	4	4	80	34,170
122	141,975	1,011	5	1	0	8	9	112	15,775
123	142,526	697	3	1	0	5	6	116	23,754
124	122,507	109	2	0	0	2	2	55	61,254
125	141,384	74	1	1	0	0	1	74	141,384
126	132,883	396	4	1	0	5	6	66	22,147
127	158,529	334	2	0	0	4	4	84	39,632
128	133,434	518	2	1	1	1	3	173	44,478
129	156,670	102	1	0	0	1	1	102	156,670
130	127,762	653	2	0	0	3	3	218	42,587
131	114,788	871	3	1	1	5	7	124	16,398
132	135,433	436	4	2	0	2	4	109	33,858
133	128,998	576	3	0	1	3	4	144	32,250
135	163,446	463	2	1	0	2	3	154	54,482
137	105,823	42	1	0	0	1	1	42	105,823
138	90,221	617	2	2	2	0	4	154	22,555
139	93,586	451	3	0	1	6	7	64	13,369
140	96,224	620	1	1	0	0	1	620	96,224
141	98,031	1,362	4	0	0	14	14	97	7,002
142	132,305	323	2	0	0	4	4	81	33,076
143	87,623	181	1	0	0	1	1	181	87,623
144	85,648	237	1	0	0	2	2	119	42,824
145	74,269	118	2	0	0	2	2	59	37,135
146	66,839	291	1	1	0	0	1	291	66,839
147	66,397	266	2	1	0	2	3	89	22,132
148	64,884	982	3	3	4	2	9	109	7,209
150	63,036	779	3	4	2	0	6	130	10,506
151	78,814	2,274	4	0	0	12	12	190	6,568
152	68,096	1,607	2	0	0	11	11	146	6,191
153	58,505	159	1	0	0	1	1	159	58,505
154	59,544	409	0	2	0	1	3	136	19,848
155	57,757	158	1	0	0	1	1	158	57,757
156	63,803	440	1	0	0	2	2	220	31,902
159	48,833	198	2	0	0	2	2	99	24,417
161	70,147	322	1	0	0	3	3	107	23,382
162	96,527	127	2	1	0	1	2	64	48,264
163	42,923	1,510	1	1	0	5	6	252	7,154
164	56,756	571	1	0	0	5	5	114	11,351
165	46,219	239	1	0	0	5	5	48	9,244
166	62,331	189	1	0	0	1	1	189	62,331
167	55,078	172	1	0	0	3	3	57	18,359
169	56,434	309	1	0	0	3	3	103	18,811
170	41,057	492	2	0	0	4	4	123	10,264
171	47,090	263	1	0	0	2	2	132	23,545
172	51,493	74	1	0	0	1	1	74	51,493
173	32,945	87	1	0	0	2	2	44	16,473
178	50,831	239	1	1	1	0	2	120	25,416
180	26,426	118	1	0	0	1	1	118	26,426
181	25,288	543	1	0	1	5	6	91	4,215
183	25,255	54	1	0	0	1	1	54	25,255
184	25,182	550	1	2	0	1	3	183	8,394
189	22,821	40	1	1	0	0	1	40	22,821
191	21,049	312	1	0	0	1	1	312	21,049
193	20,340	312	1	0	0	1	1	312	20,340
195	20,131	38	1	0	0	1	1	38	20,131
197	19,986	343	1	0	1	0	1	343	19,986
202	18,478	80	1	0	0	1	1	80	18,478
203	18,097	65	1	1	0	0	1	65	18,097
204	19,139	43	1	0	0	1	1	43	19,139
205	19,822	671	2	0	0	6	6	112	3,304
207	17,451	498	1	0	0	4	4	125	4,363
209	16,612	166	1	0	0	1	1	166	16,612
210	16,297	163	1	0	0	1	1	163	16,297
213	15,902	189	1	0	0	2	2	95	7,951
214	16,066	603	1	0	0	3	3	201	5,355
216	15,718	318	1	0	0	2	2	159	7,859
219	15,090	55	1	0	0	1	1	55	15,090
220	14,899	34	1	0	1	0	1	34	14,899
221	14,629	71	1	0	0	2	2	36	7,315
225	13,856	702	1	2	1	0	3	234	4,619
227	13,483	124	1	1	0	0	1	124	13,483
228	13,460	488	1	0	0	3	3	163	4,487
236	12,526	158	1	0	0	1	1	158	12,526
237	12,336	1,760	1	0	0	8	8	220	1,542
238	12,284	618	1	2	0	0	2	309	6,142
239	15,052	80	1	1	0	0	1	80	15,052
241	12,067	1,285	1	3	1	4	8	161	1,508
246	11,389	538	1	0	1	0	1	538	11,389
249	10,855	35	1	1	0	0	1	35	10,855
256	10,451	546	1	0	0	3	3	182	3,484
262	10,127	78	1	0	0	1	1	78	10,127
263	10,113	277	1	0	0	5	5	55	2,023
265	10,029	532	1	0	0	4	4	133	2,507
267	9,743	129	1	0	0	1	1	129	9,743
268	9,738	215	1	0	1	2	3	72	3,246
270	9,523	125	1	0	0	1	1	125	9,523
271	9,385	101	1	0	0	2	2	51	4,693
273	9,255	96	1	0	0	1	1	96	9,255
276	8,976	139	1	0	1	0	1	139	8,976
278	8,937	70	1	0	0	1	1	70	8,937
286	8,437	50	1	0	0	1	1	50	8,437
287	8,404	215	1	0	0	3	3	72	2,801
291	8,194	79	1	0	0	1	1	79	8,194
292	8,128	469	1	0	0	4	4	117	2,032
293	8,101	545	1	0	0	5	5	109	1,620
295	8,046	863	1	1	1	0	2	432	4,023
298	7,881	91	1	0	0	1	1	91	7,881
299	7,678	2,047	1	0	0	5	5	409	1,536
307	7,428	53	1	0	0	1	1	53	7,428
311	7,373	83	1	0	0	1	1	83	7,373
313	7,328	154	1	1	0	0	1	154	7,328
325	6,734	338	1	0	0	2	2	169	3,367
331	6,471	370	1	3	1	0	4	93	1,618
340	6,192	281	1	0	0	3	3	94	2,064
351	5,919	167	1	1	0	0	1	167	5,919
359	5,526	604	1	0	0	3	3	201	1,842
360	5,501	281	1	0	0	1	1	281	5,501
363	5,438	477	1	0	0	4	4	119	1,360
368	5,333	375	1	0	0	2	2	188	2,667
371	5,291	383	1	0	0	4	4	96	1,323
374	5,126	432	1	0	0	3	3	144	1,709
375	5,108	266	1	0	0	1	1	266	5,108
376	5,100	304	1	0	0	5	5	61	1,020
377	5,096	63	1	0	1	0	1	63	5,096
384	4,789	30	1	0	0	1	1	30	4,789
386	4,740	98	1	0	0	1	1	98	4,740
392	2,878	39,740	1	76	12	62	150	265	19

Total	86,550,604	787,413	1,447	1,088	572	2,952	4,604	35,082	18,799
