# Supplementary material for: Constructing Physical and Genomic Maps for Puccinia striiformis f. sp. tritici, the Wheat Stripe Rust Pathogen, by Comparing Its EST Sequences to the Genomic Sequence of P. graminis f. sp. tritici, the Wheat Stem Rust Pathogen
Source: Comp Funct Genomics. 2010 Feb 11;2009:302620. doi: 10.1155/2009/302620 (PMC2821759; doi:10.1155/2009/302620)
Supplement: Supplementary file 1 — Physical maps for Pst ESTs based on corresponding sequence positions of homologous genes of Pgt. A total of 242 physical groups are constructed. The distance in mega base (Mb) is shown on the left. The clones in a group indicated by a vertical line are in the same contig and the start and end positions of the sequence matching the positions in the contig are shown in the “( )” following the clone identification number. The number after the “( )” indicates the number of the gene with multiple positions in the Pgt genome. An asterisk indicate that the number of matching base pairs is smaller than 100. The clones underlined were used in PCR amplification of the Pst BAC library. [file 302620.f1.pdf]

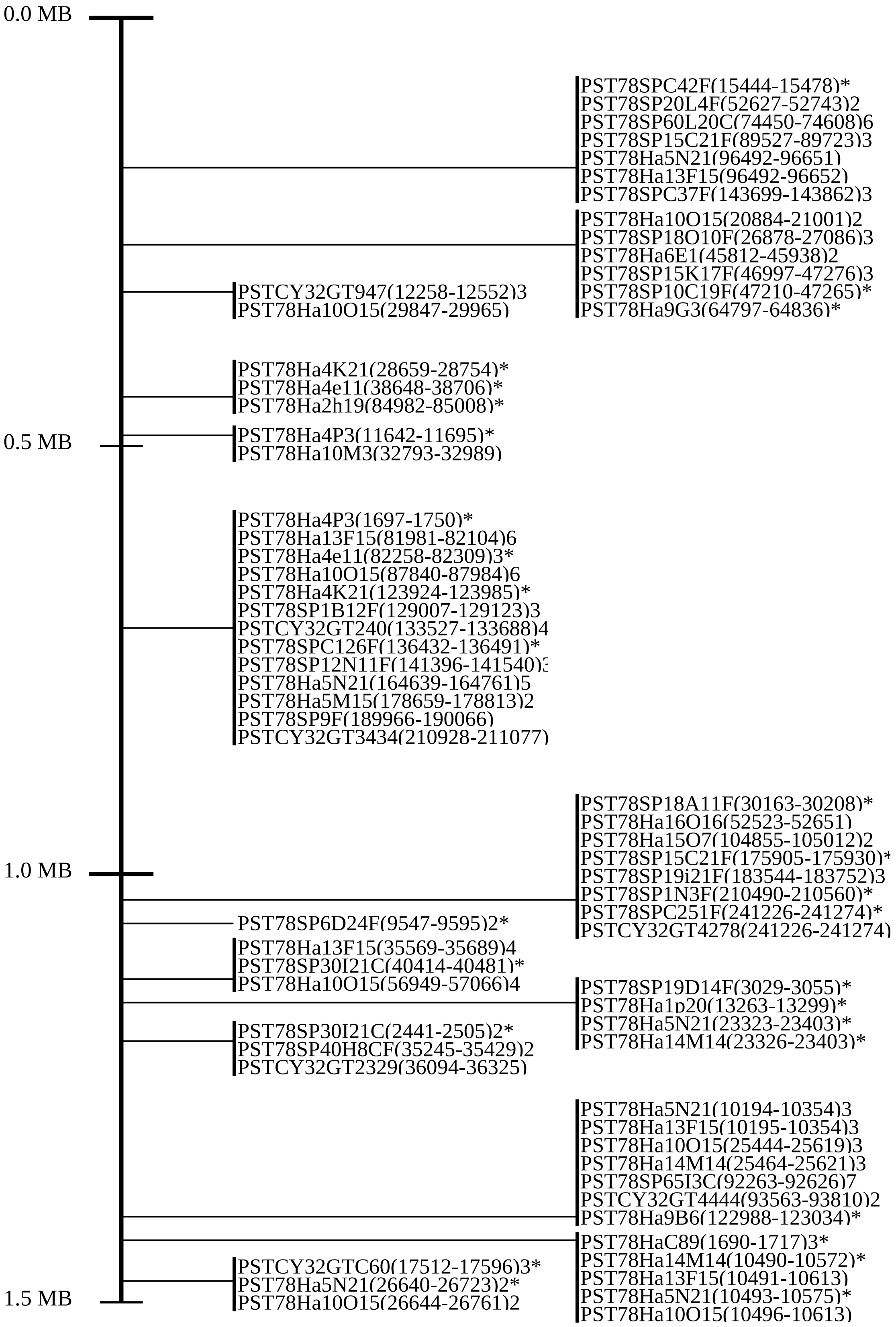

Supercontig 1 (A)
length: 3,081,398 bp

*** = less than 100bp alignment** **| = clones in the same contig**

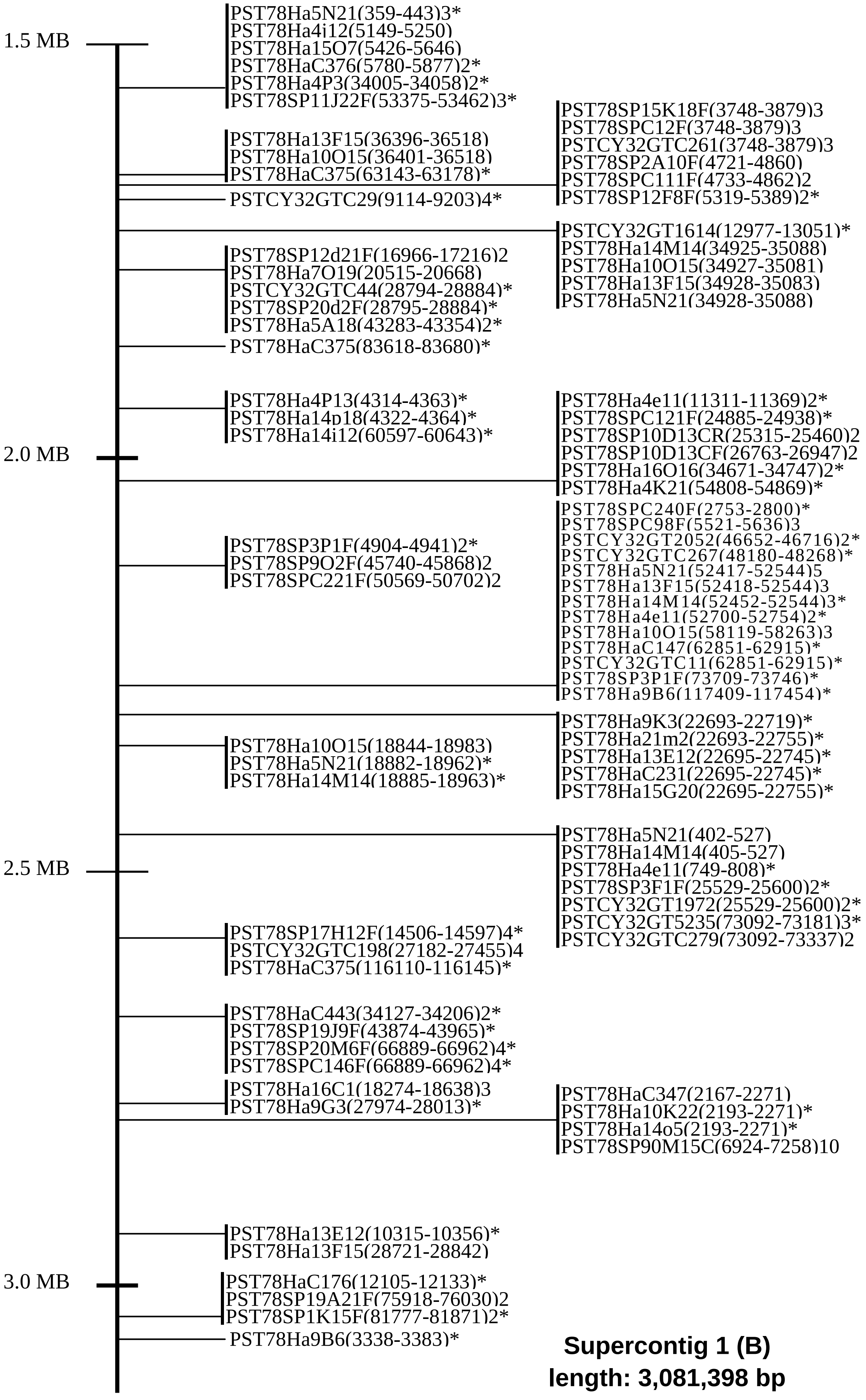

*** = less than 100bp alignment** **| = clones in the same contig**

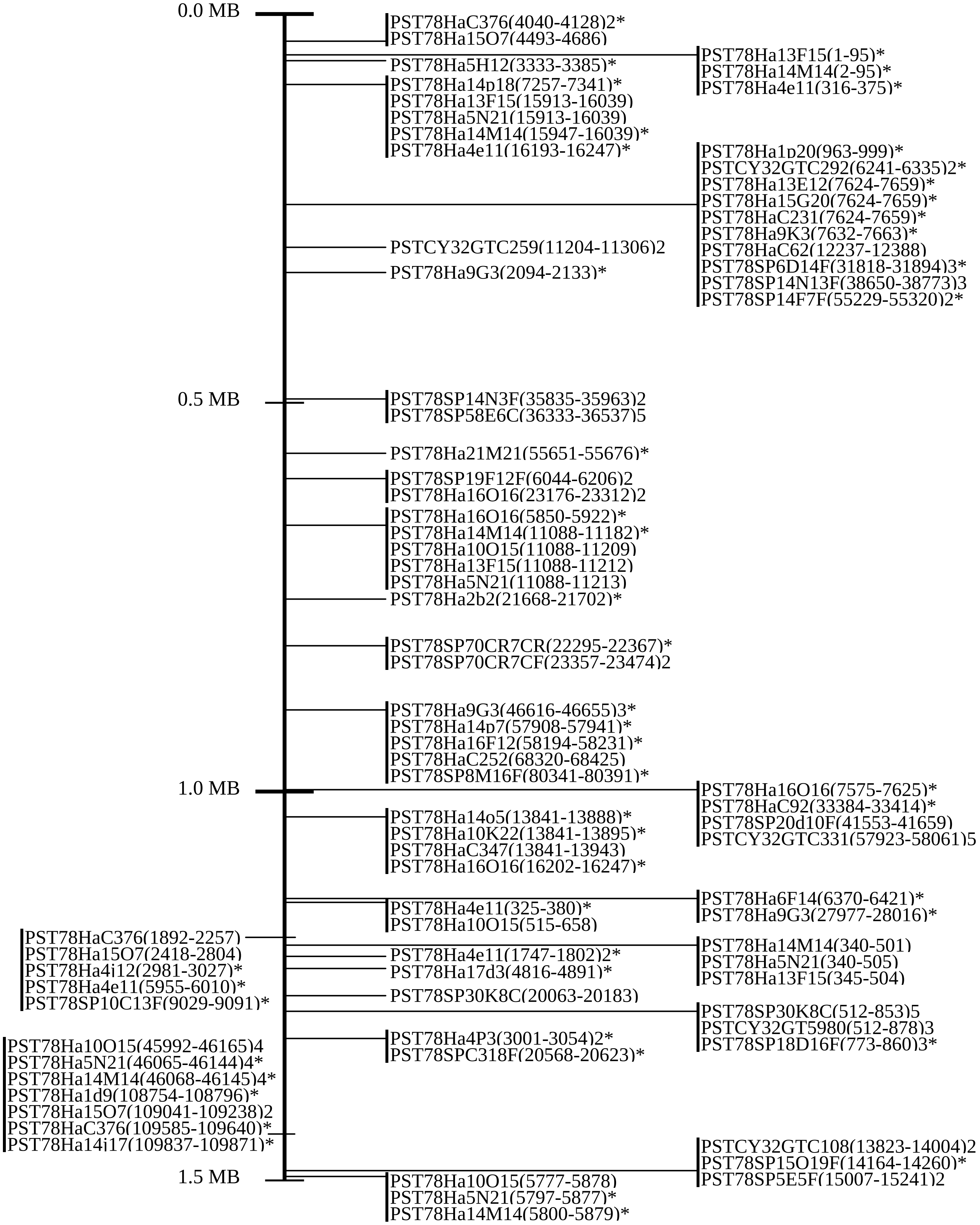

Supercontig 2 (A)
length: 2,570,998 bp

*** = less than 100bp alignment** **| = clones in the same contig**

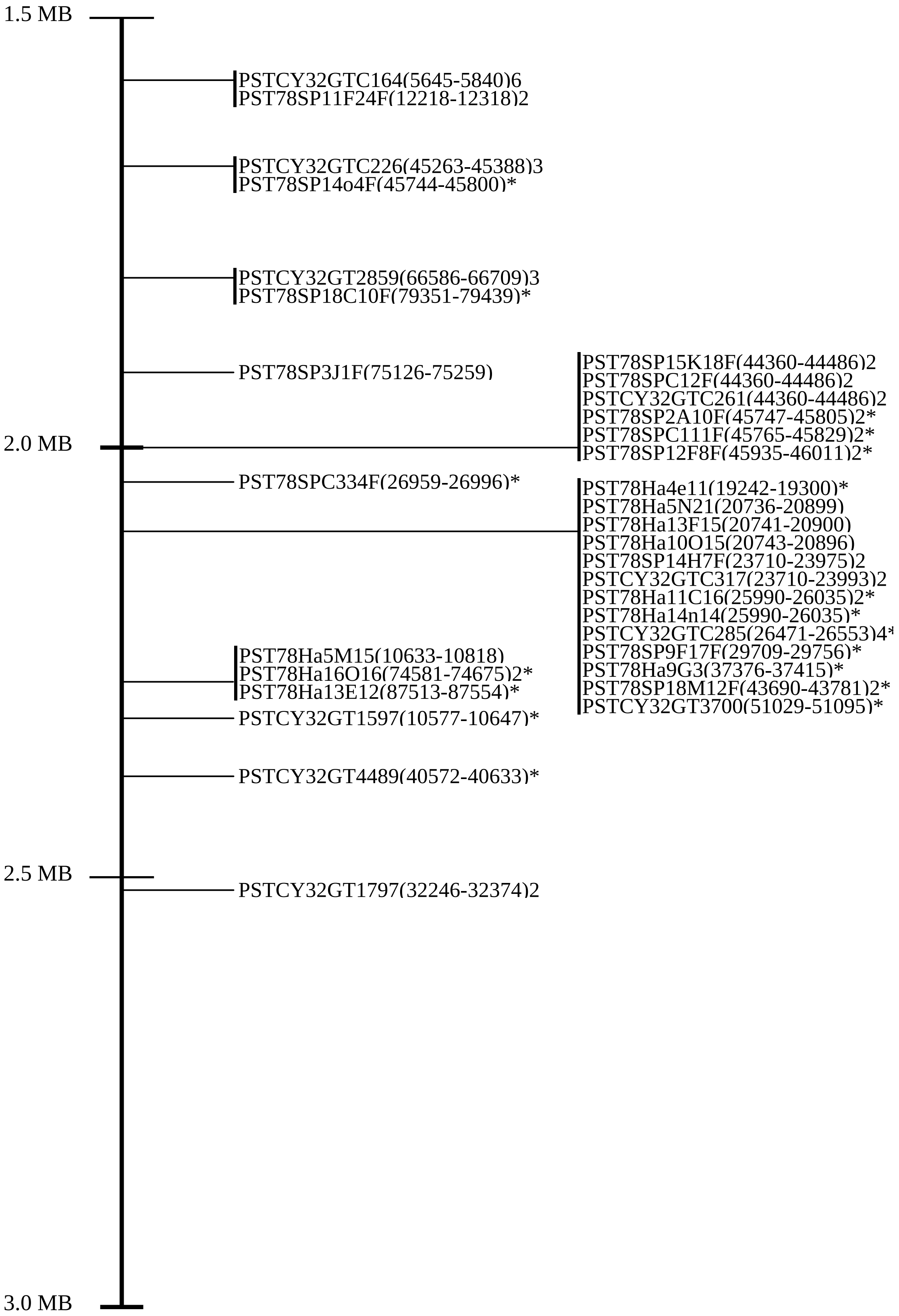

Supercontig 2 (B)
length: 2,570,998 bp

*** = less than 100bp alignment** **| = clones in the same contig**

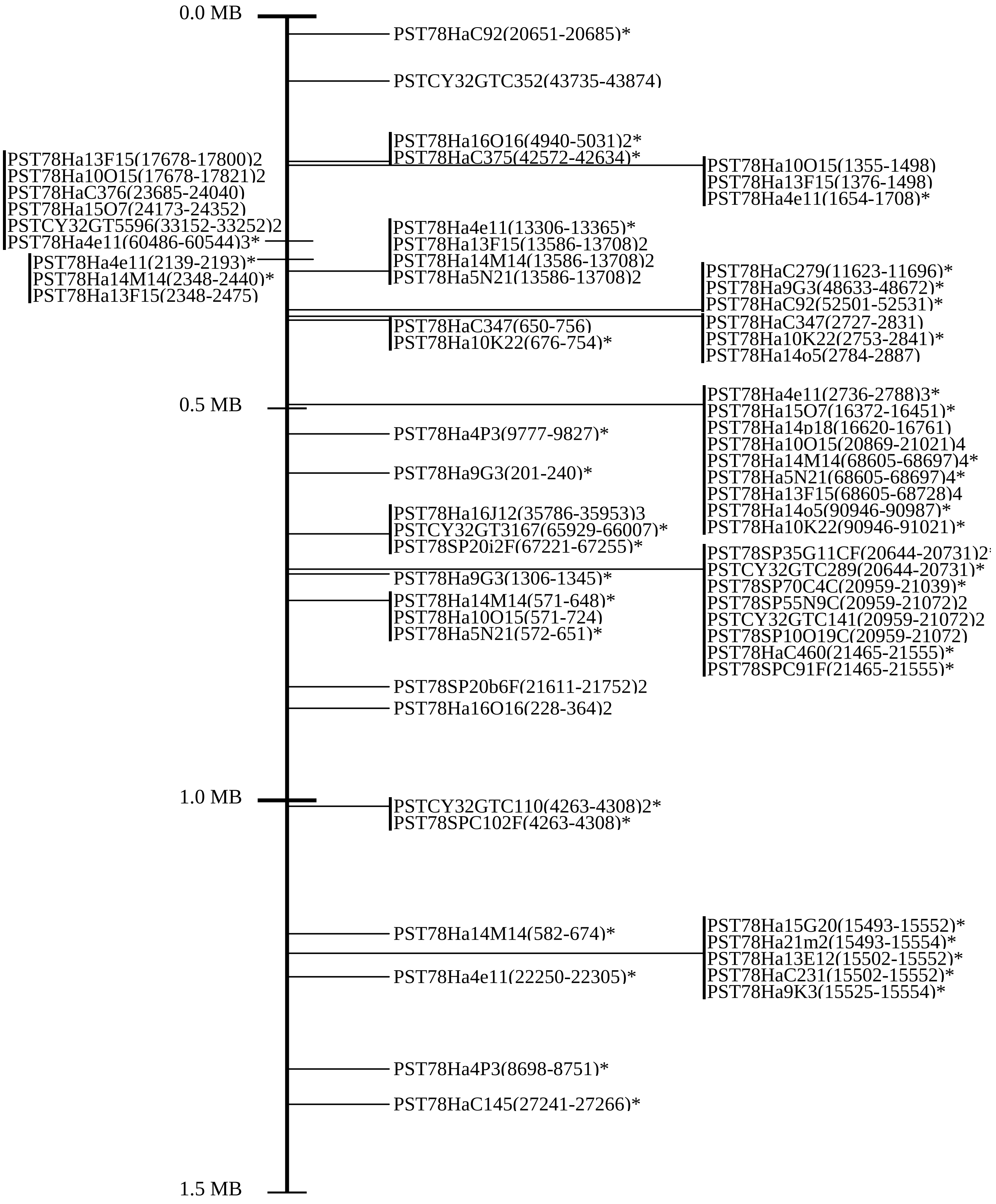

Supercontig 3 (A)
length: 2,616,274 bp

*** = less than 100bp alignment** **| = clones in the same contig**

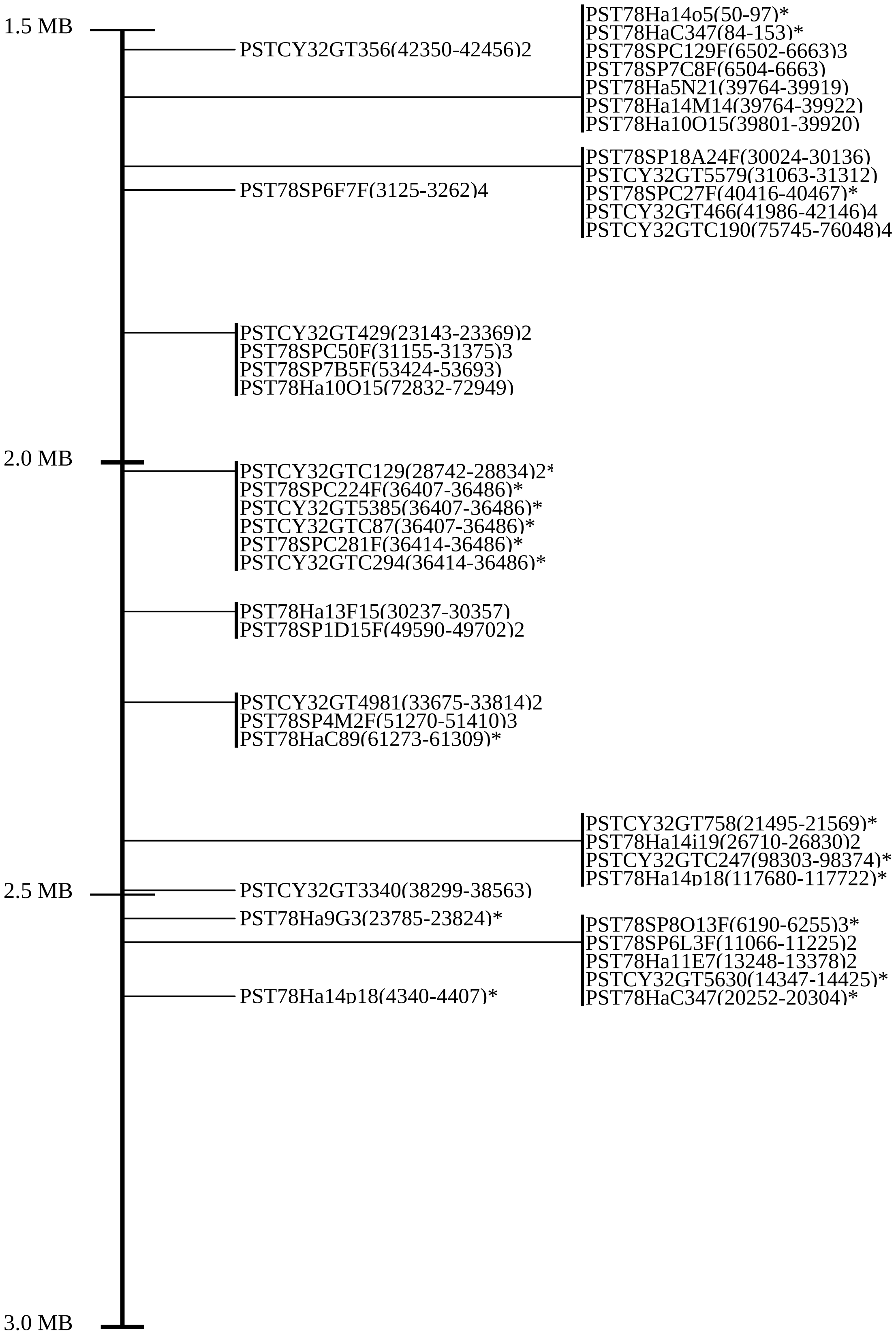

Supercontig 3 (B)
length: 2,616,274 bp

*** = less than 100bp alignment** | **| = clones in the same contig**

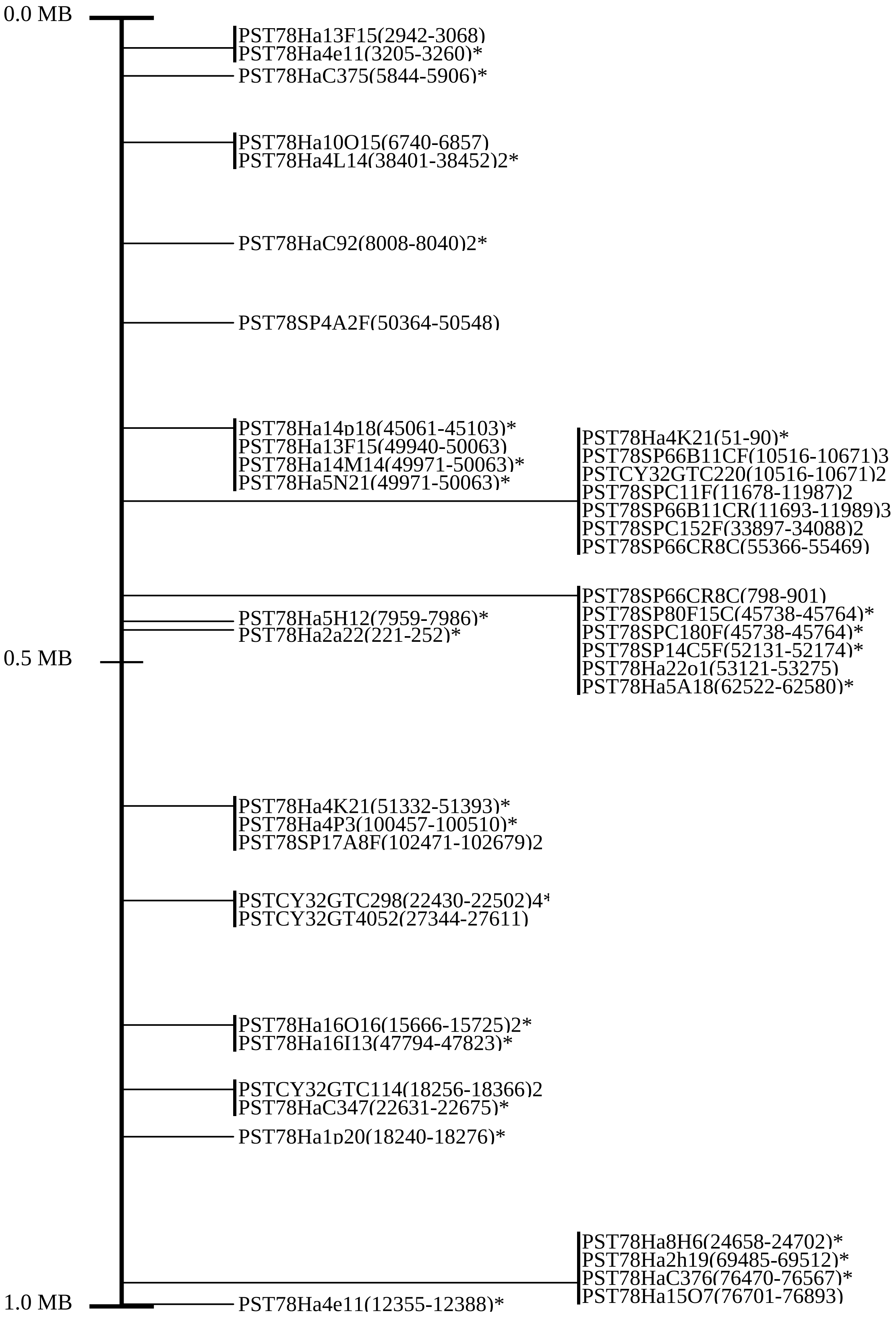

Supercontig 4 (A)
length: 1,978,325 bp

*** = less than 100bp alignment** **| = clones in the same contig**

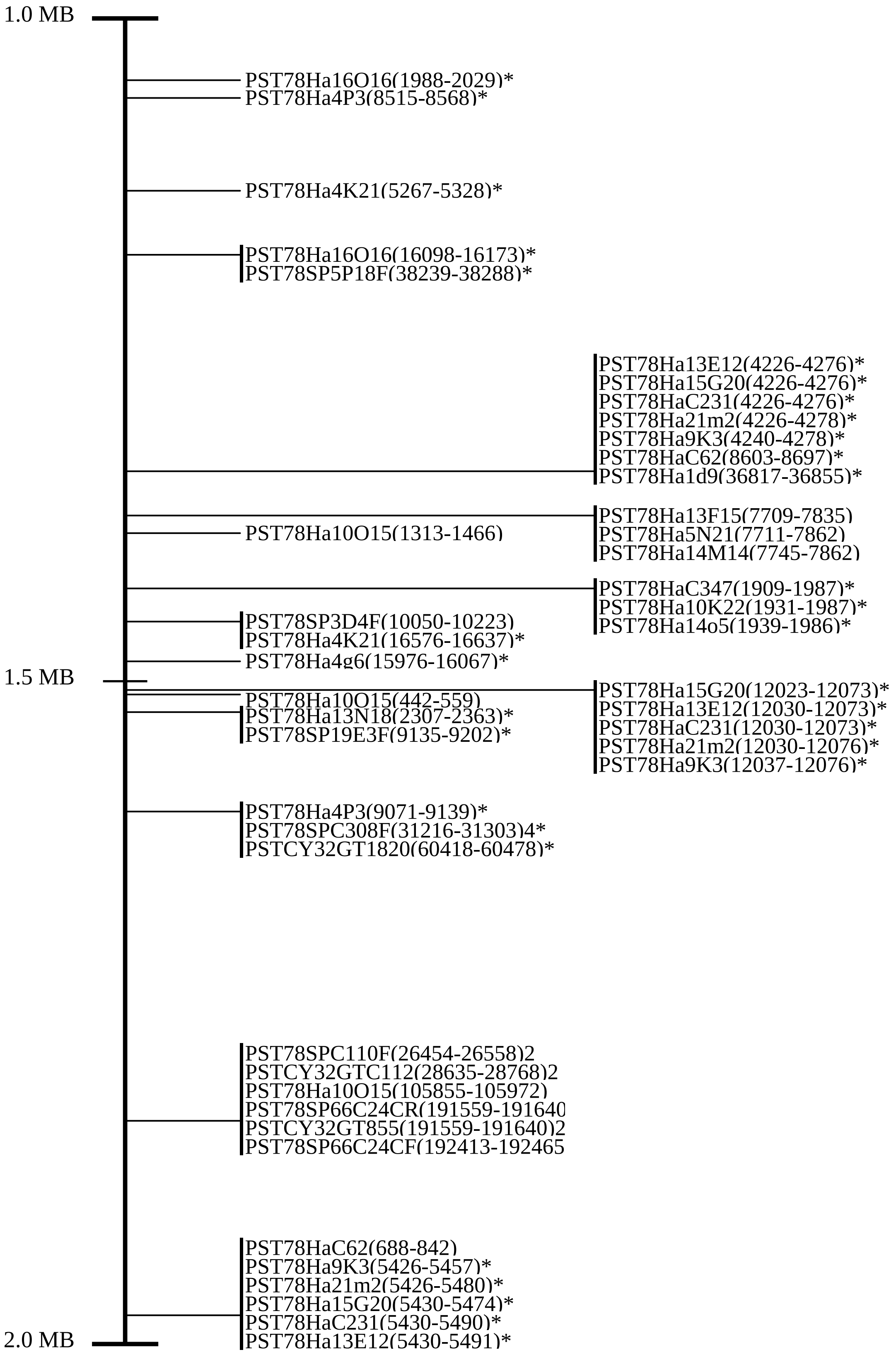

Supercontig 4 (B)
length: 1,978,325 bp

*** = less than 100bp alignment** **| = clones in the same contig**

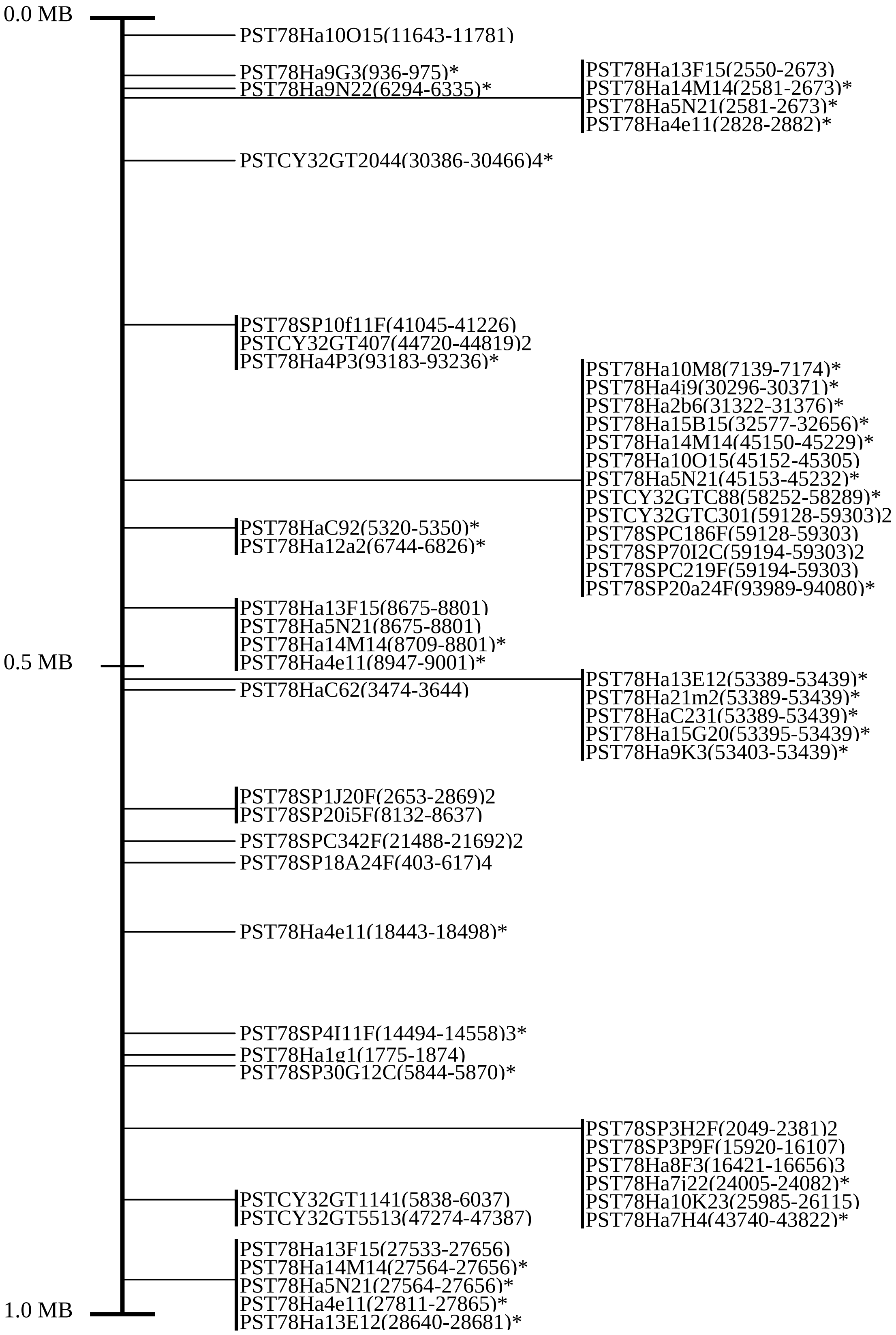

Supercontig 5 (A)
length: 2,008,477 bp

*** = less than 100bp alignment** **| = clones in the same contig**

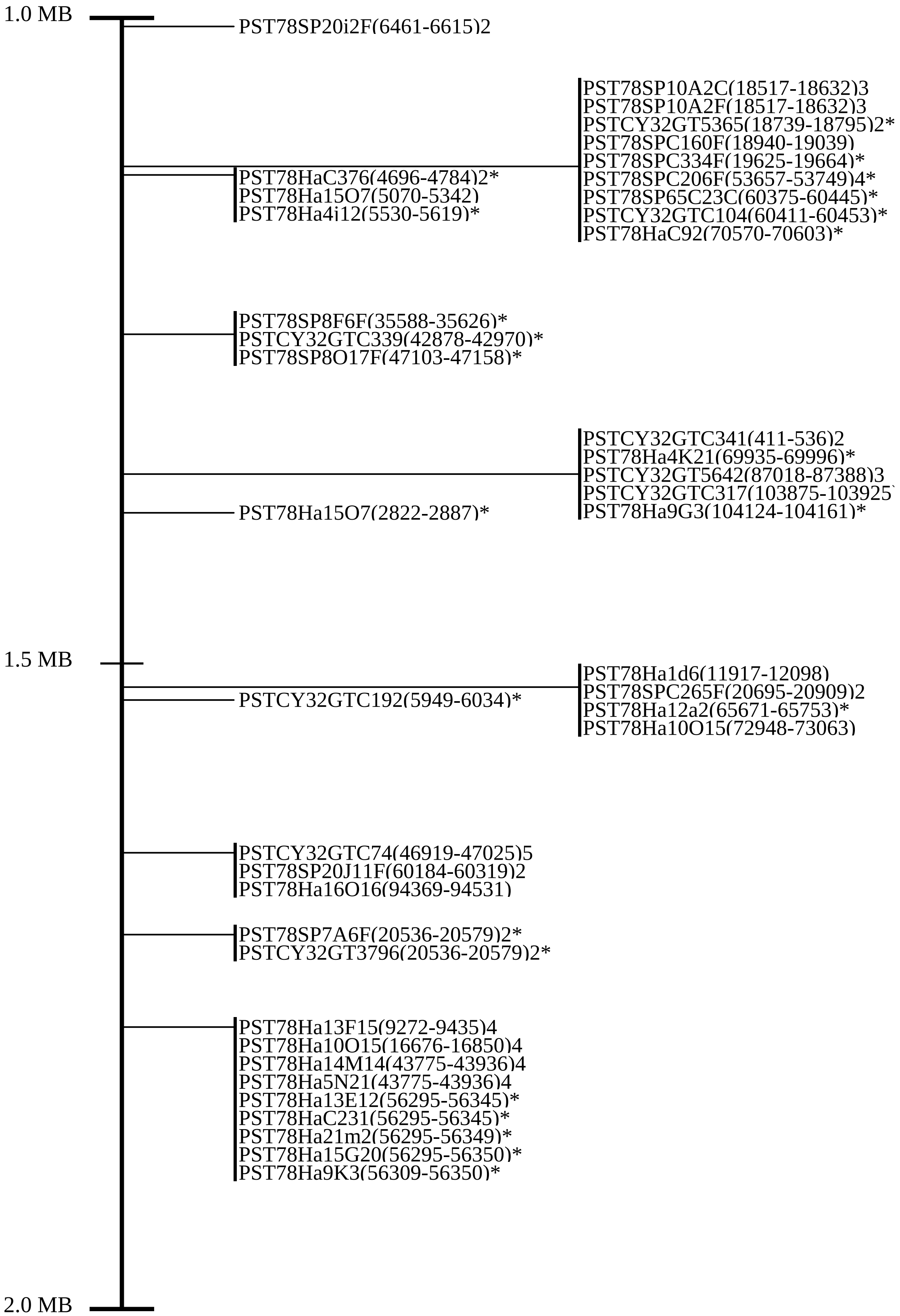

Supercontig 5 (B)
length: 2,008,477 bp

*** = less than 100bp alignment** **| = clones in the same contig**

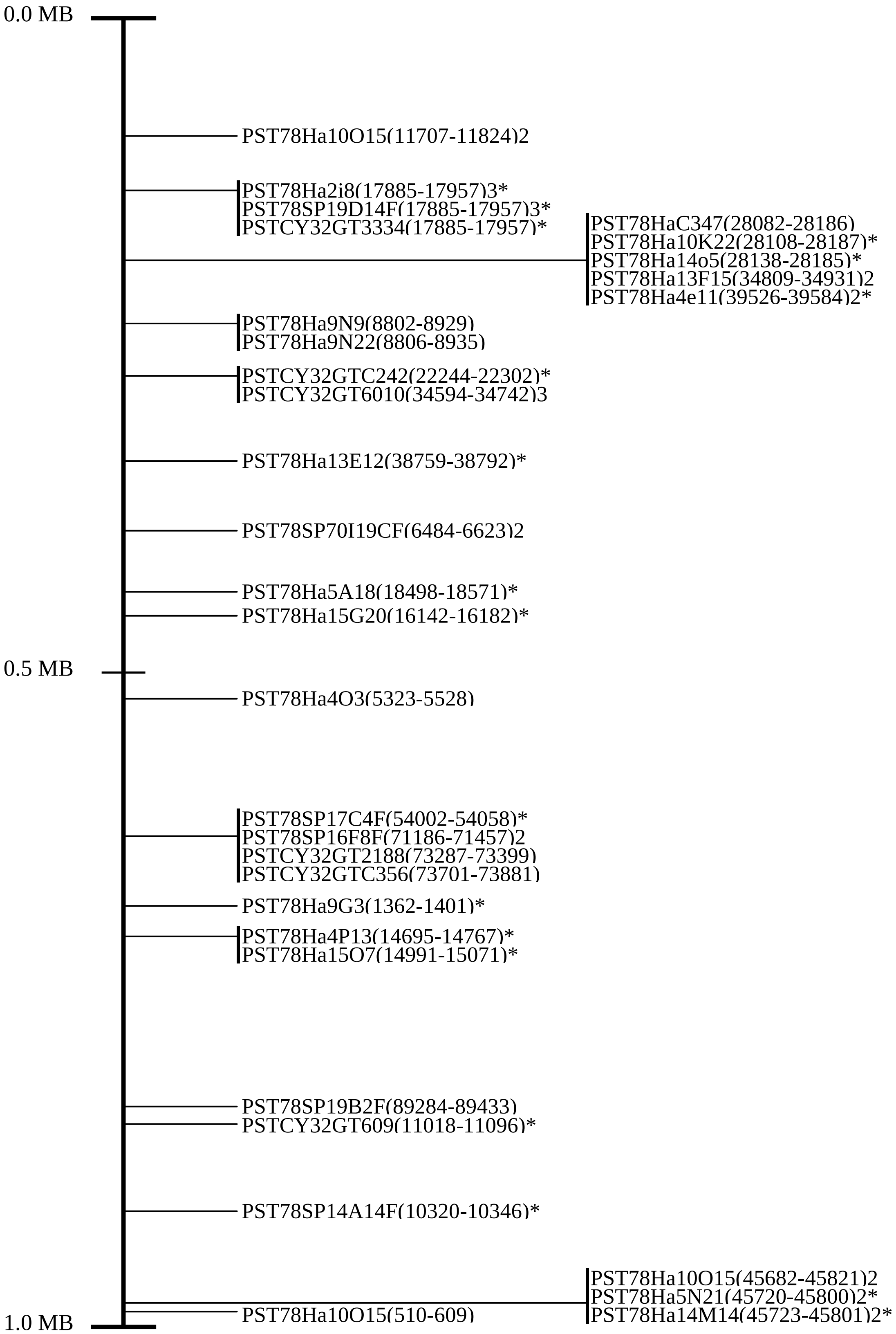

Supercontig 6 (A)
length: 1,808,965 bp

*** = less than 100bp alignment** **| = clones in the same contig**

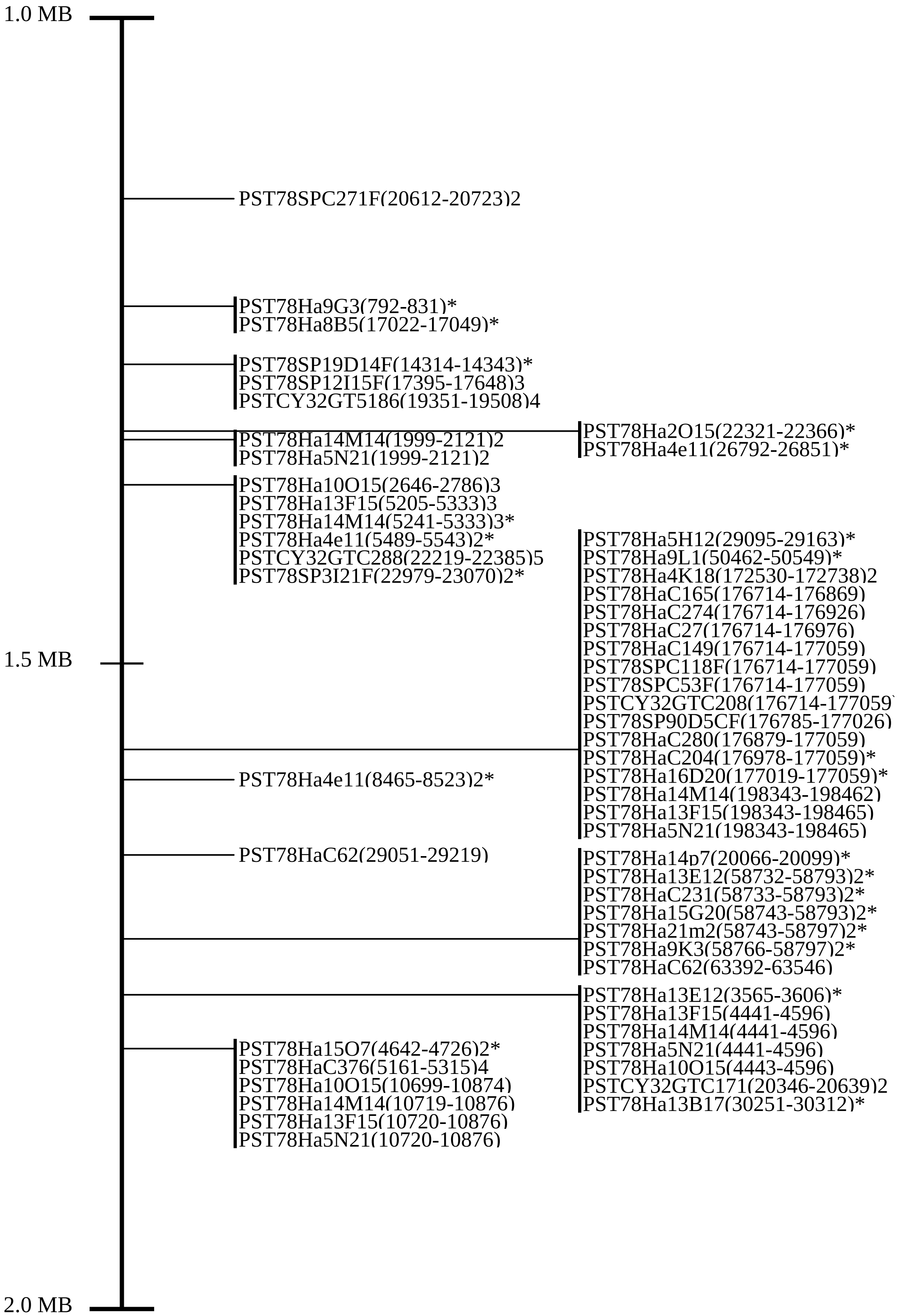

Supercontig 6 (B)
length: 1,808,965 bp

*** = less than 100bp alignment**

| = clones in the same contig

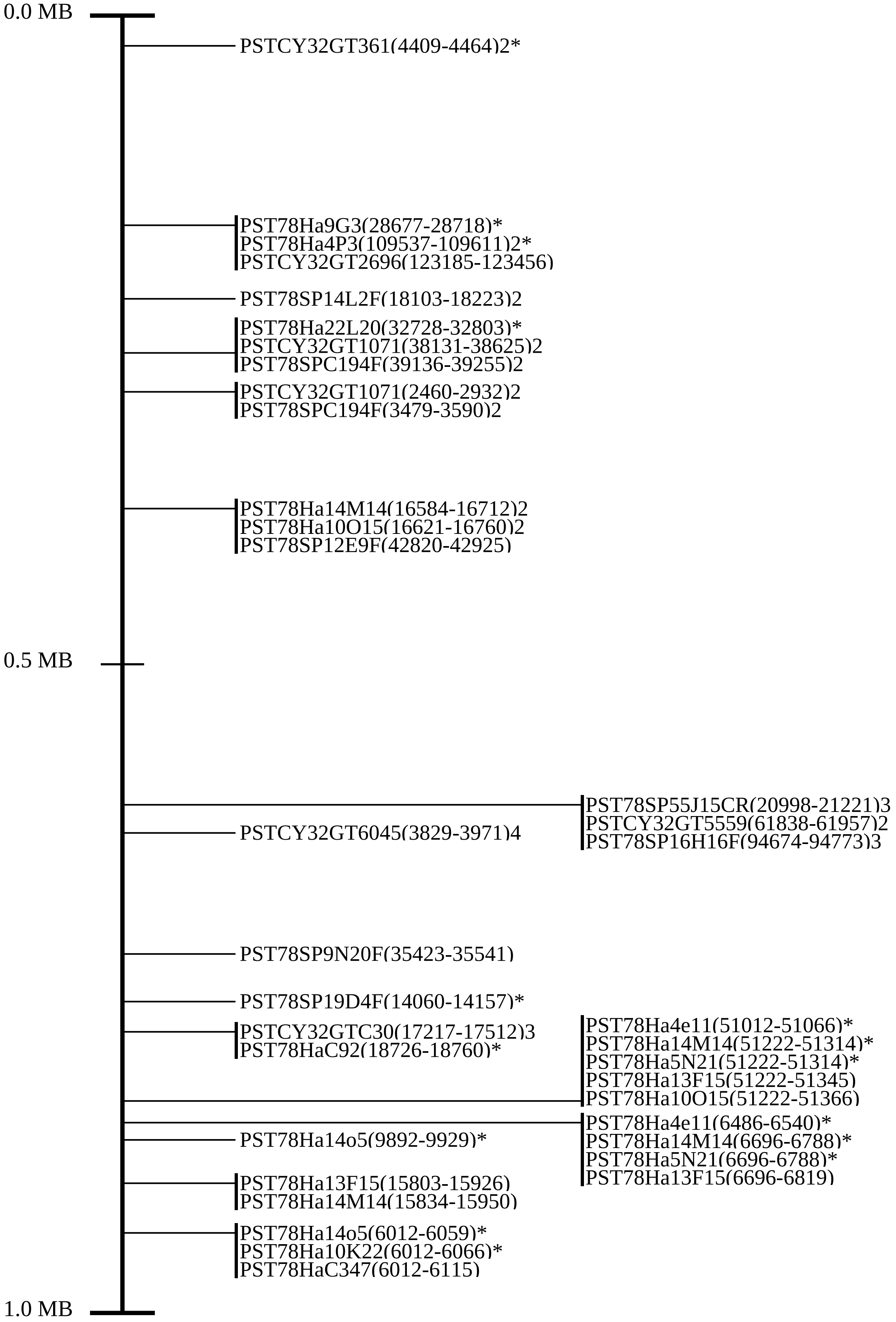

Supercontig 7 (A)
length: 1,797,936 bp

*** = less than 100bp alignment** **| = clones in the same contig**

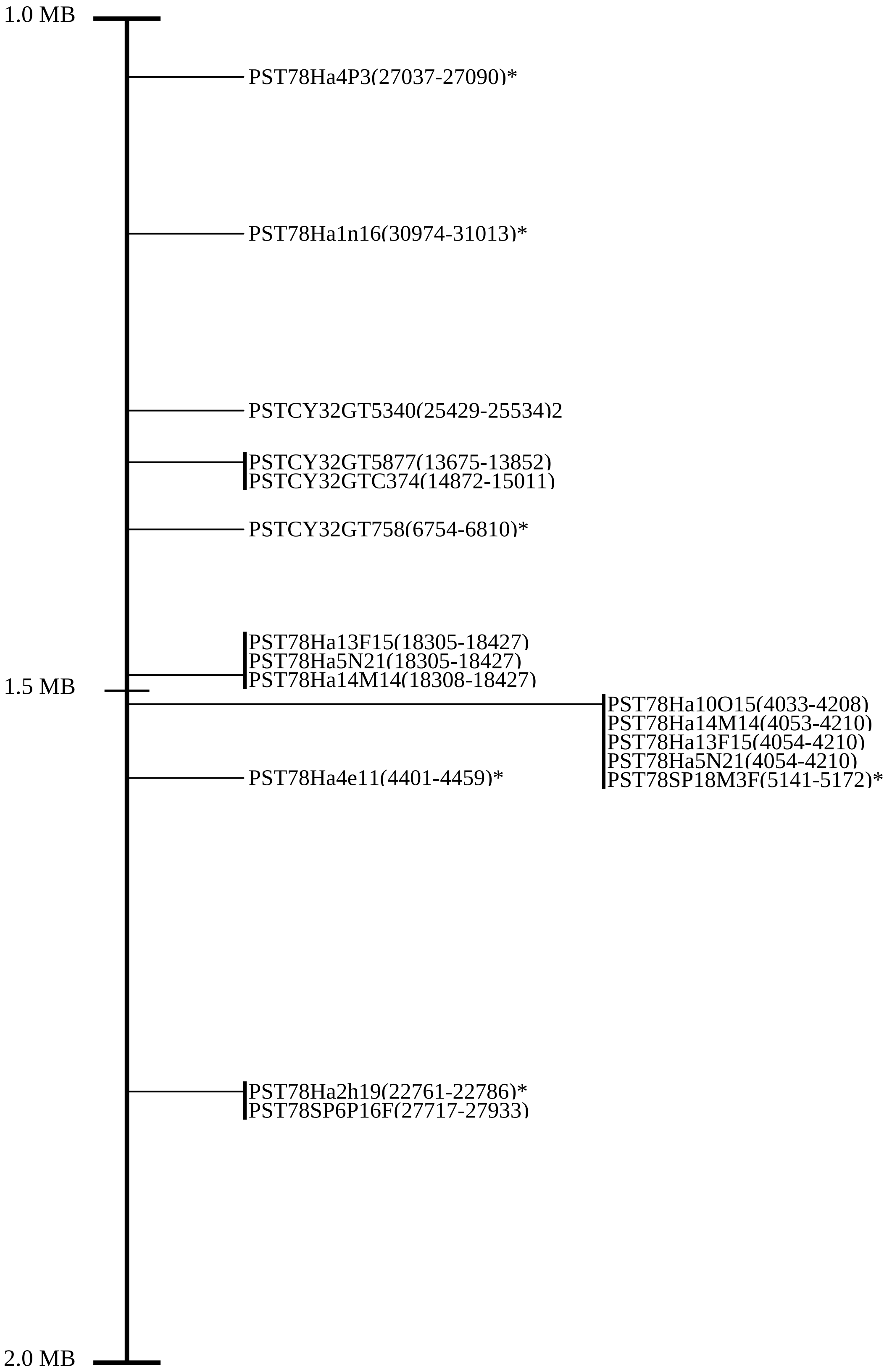

Supercontig 7 (B)
length: 1,797,936 bp

*** = less than 100bp alignment** **| = clones in the same contig**

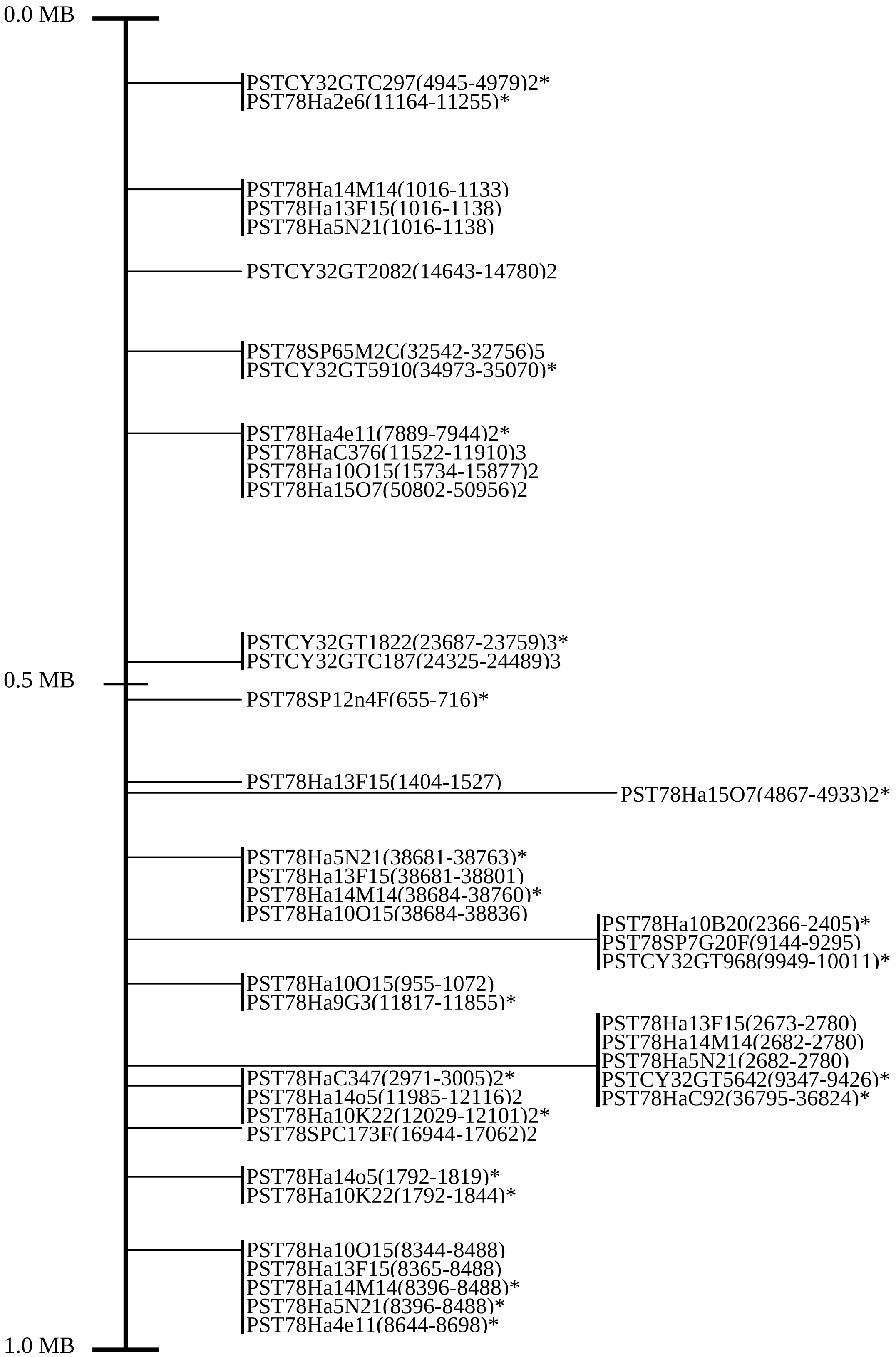

Supercontig 8 (A)
length: 1,737,638 bp

*** = less than 100bp alignment** **| = clones in the same contig**

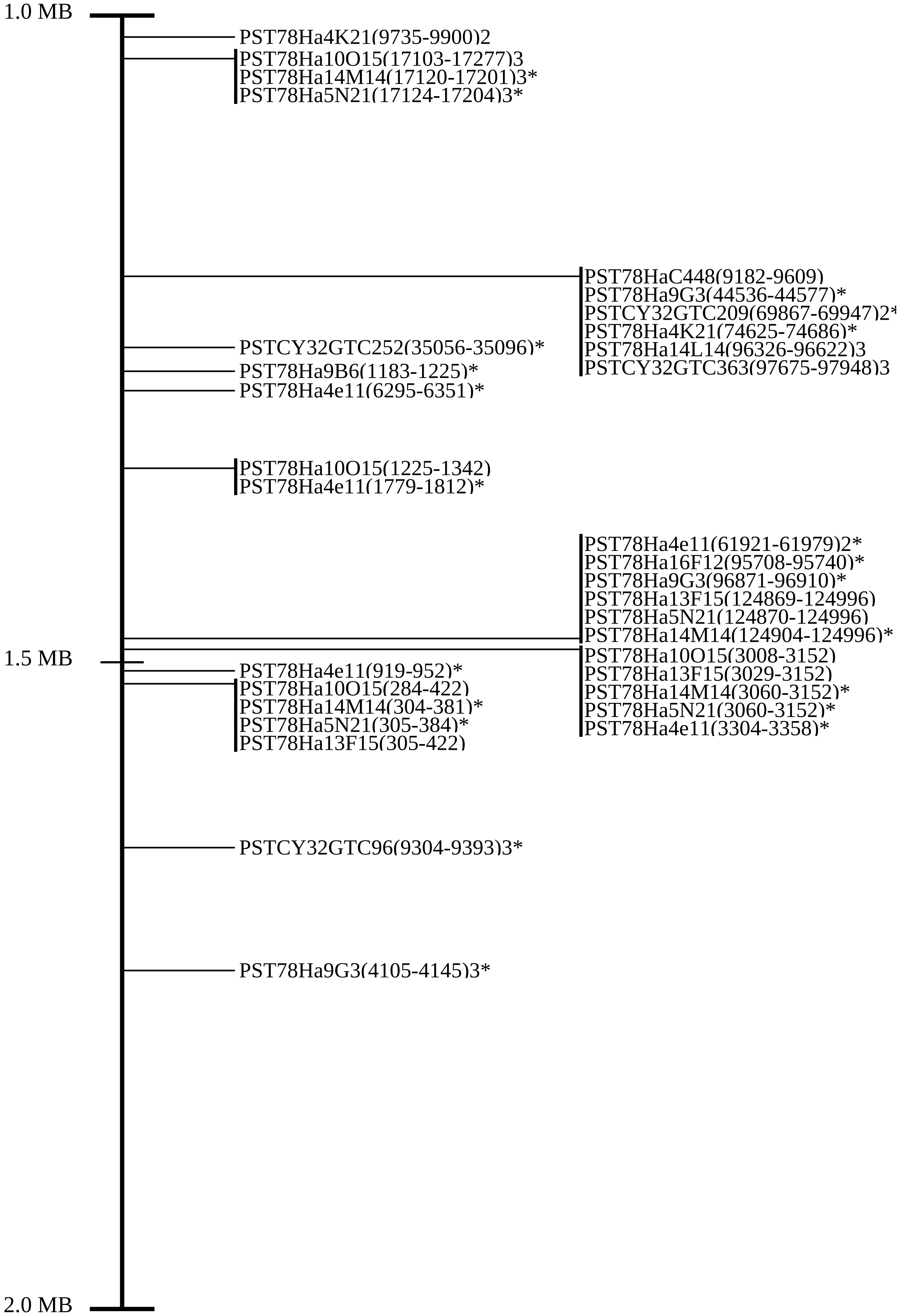

Supercontig 8 (B)
length: 1,737,638 bp

*** = less than 100bp alignment** **| = clones in the same contig**

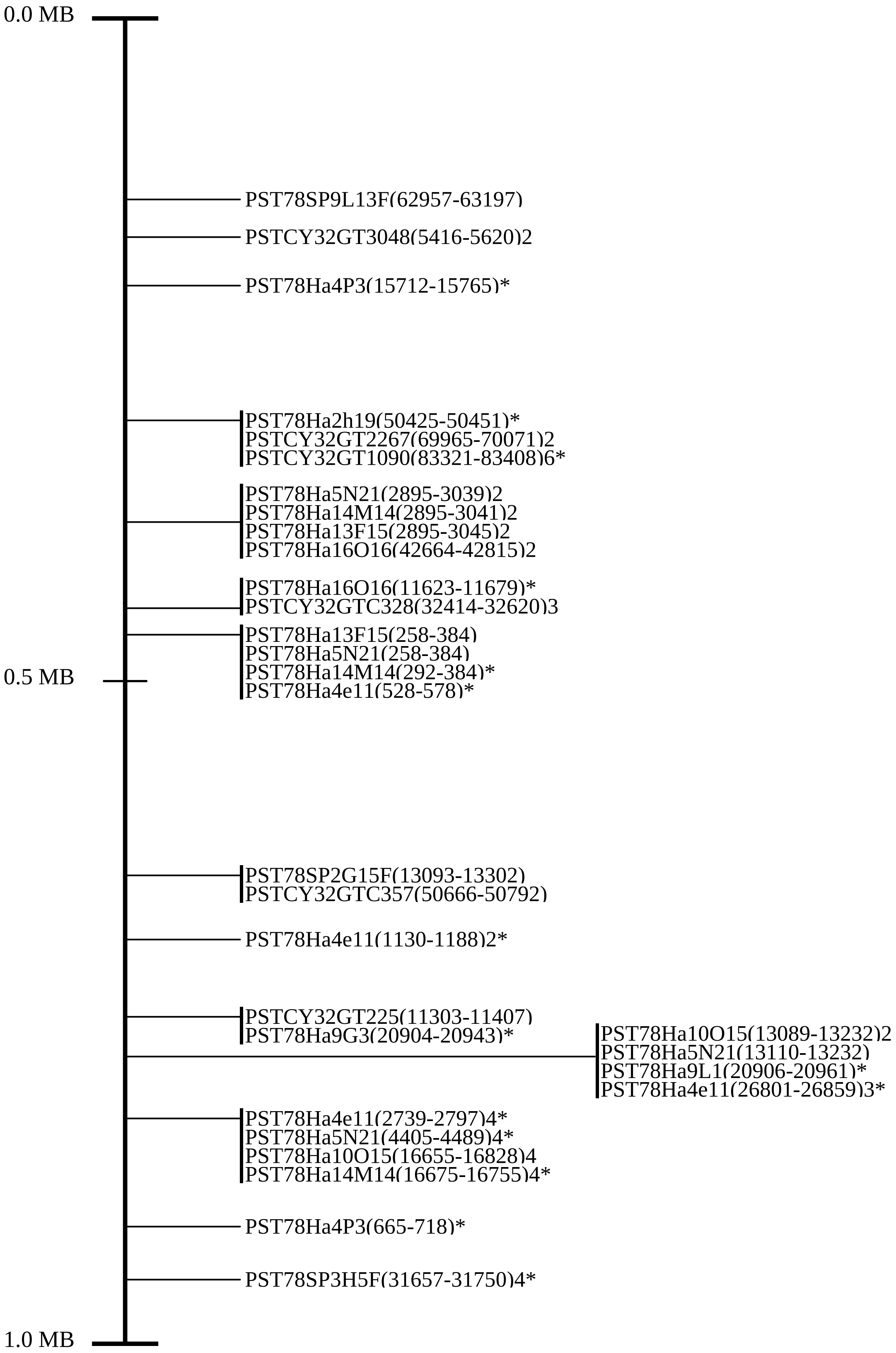

Supercontig 9 (A)
length: 1,714,174 bp

*** = less than 100bp alignment** **| = clones in the same contig**

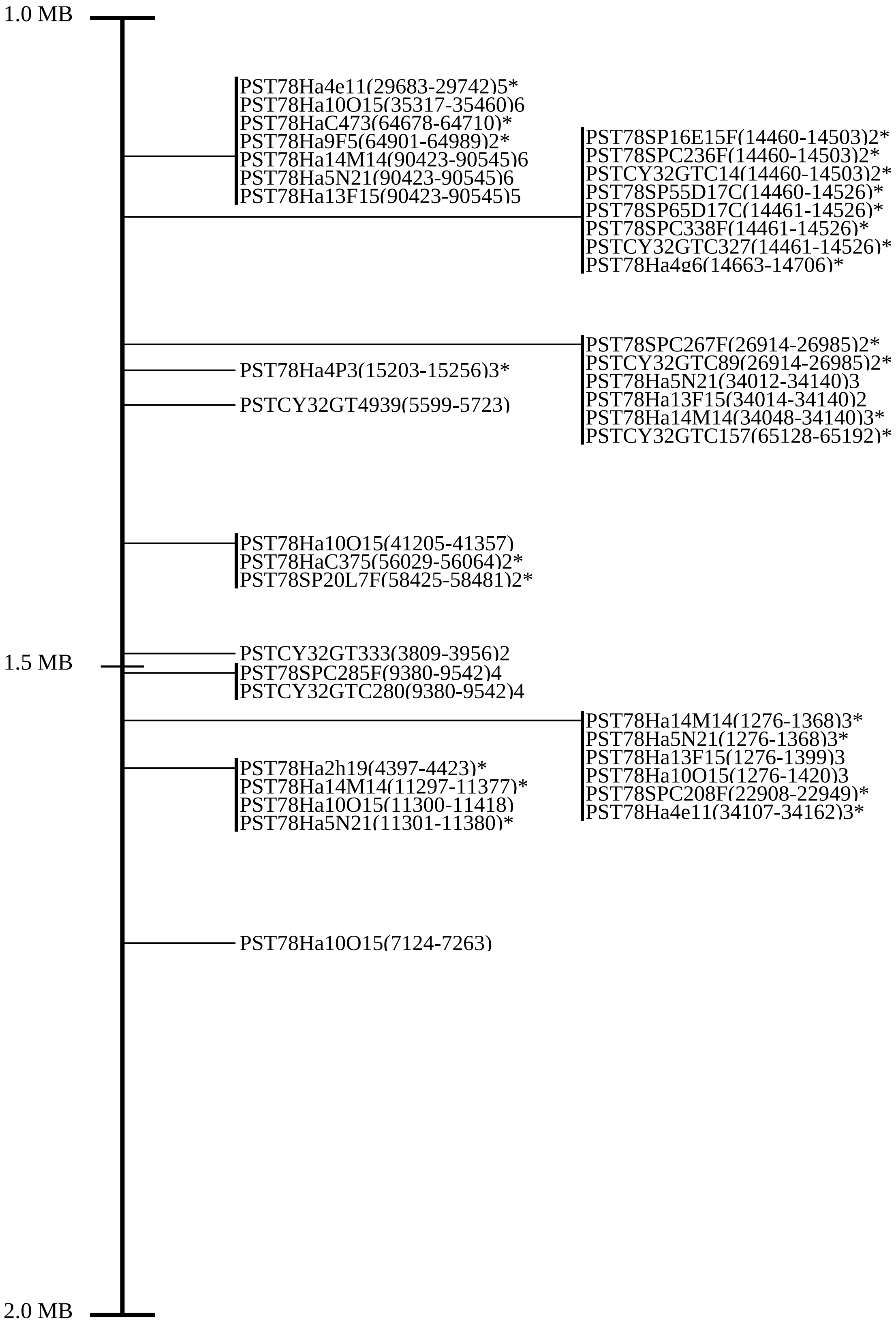

Supercontig 9 (B)
length: 1,714,174 bp

*** = less than 100bp alignment** **| = clones in the same contig**

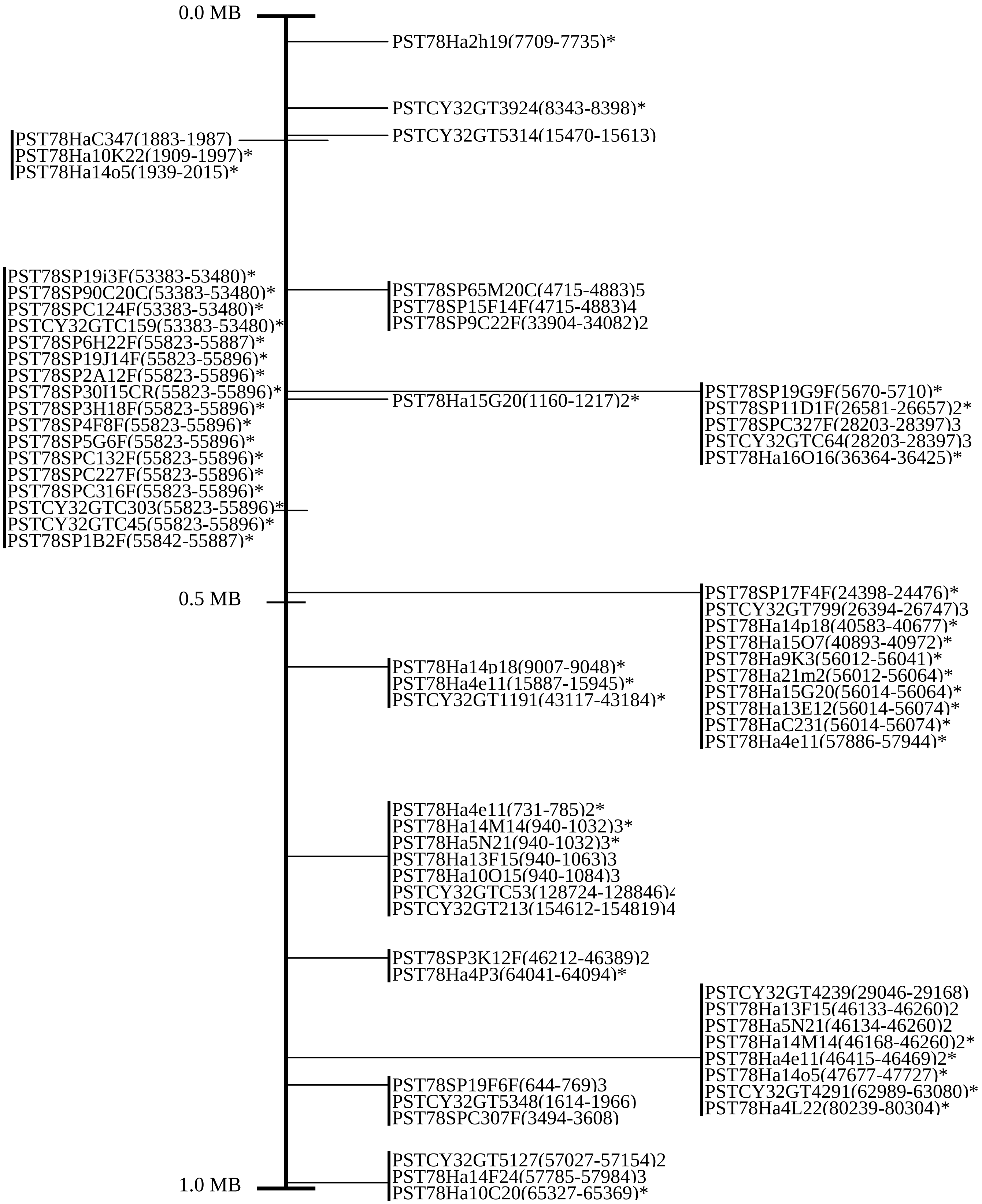

Supercontig 10 (A)
length: 1,640,743 bp

*** = less than 100bp alignment** **| = clones in the same contig**

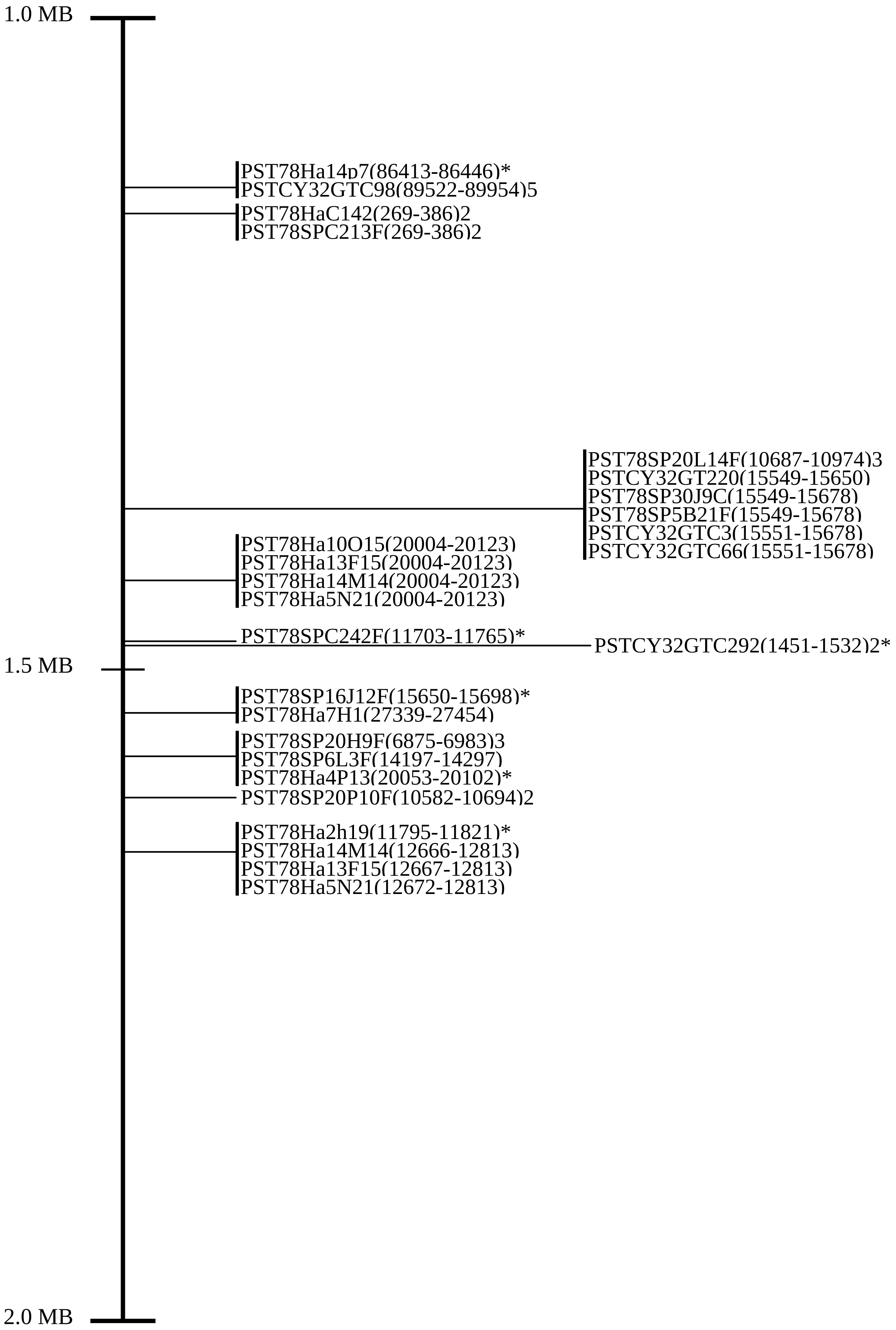

Supercontig 10 (B)
length: 1,640,743 bp

*** = less than 100bp alignment** **| = clones in the same contig**

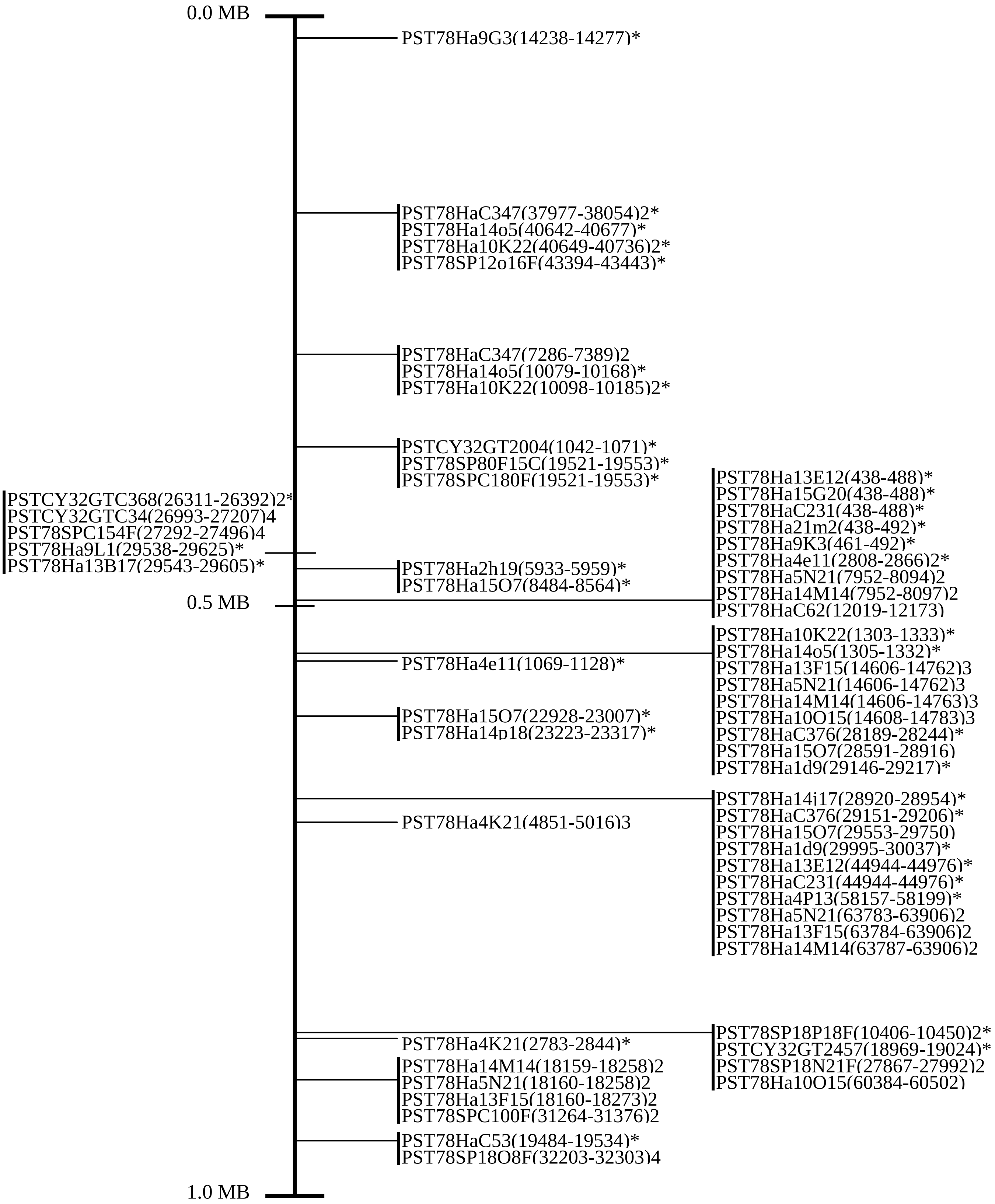

Supercontig 11 (A)
length: 1,547,344 bp

*** = less than 100bp alignment** **| = clones in the same contig**

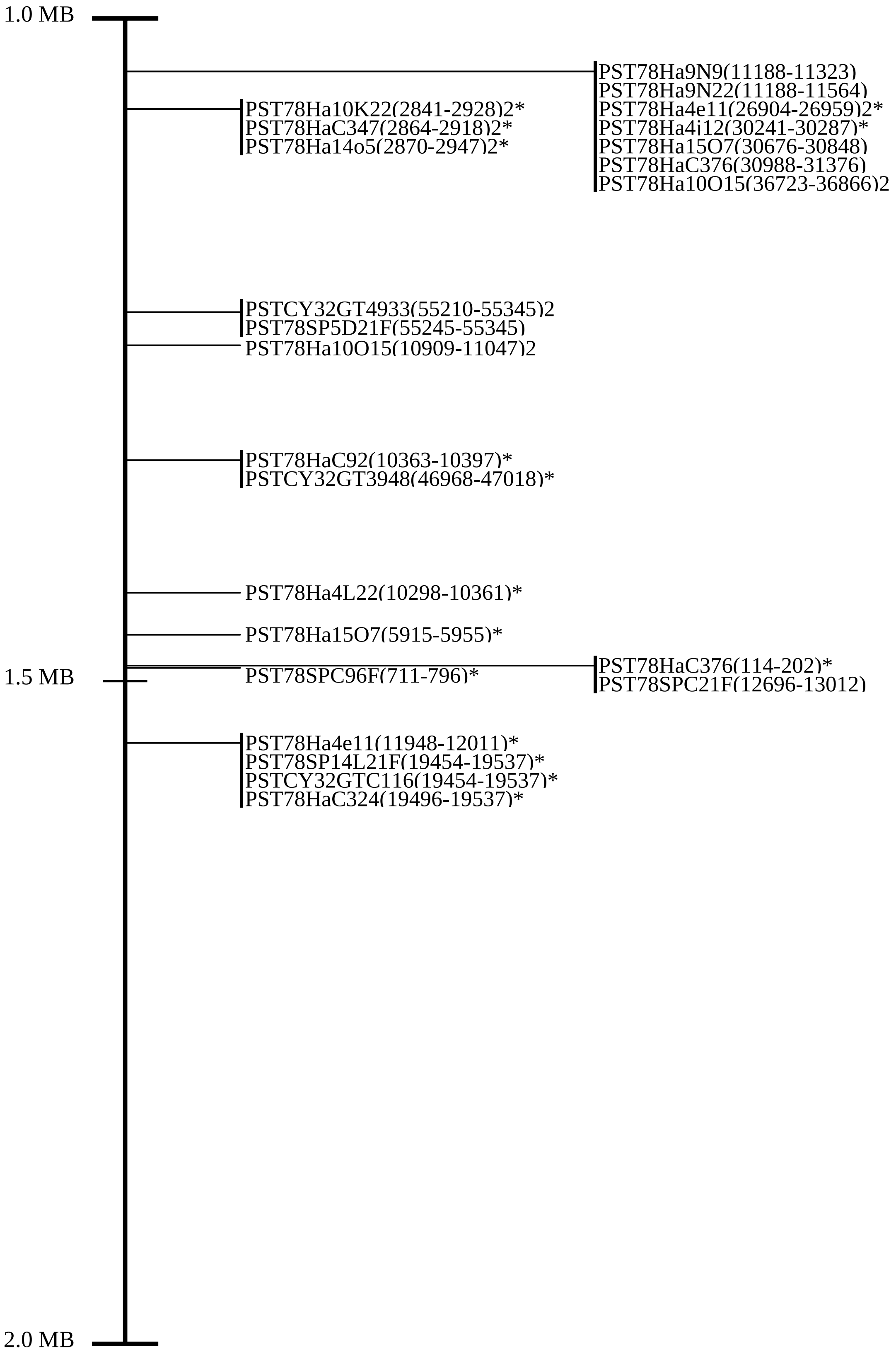

Supercontig 11 (B)
length: 1,547,344 bp

*** = less than 100bp alignment** **| = clones in the same contig**

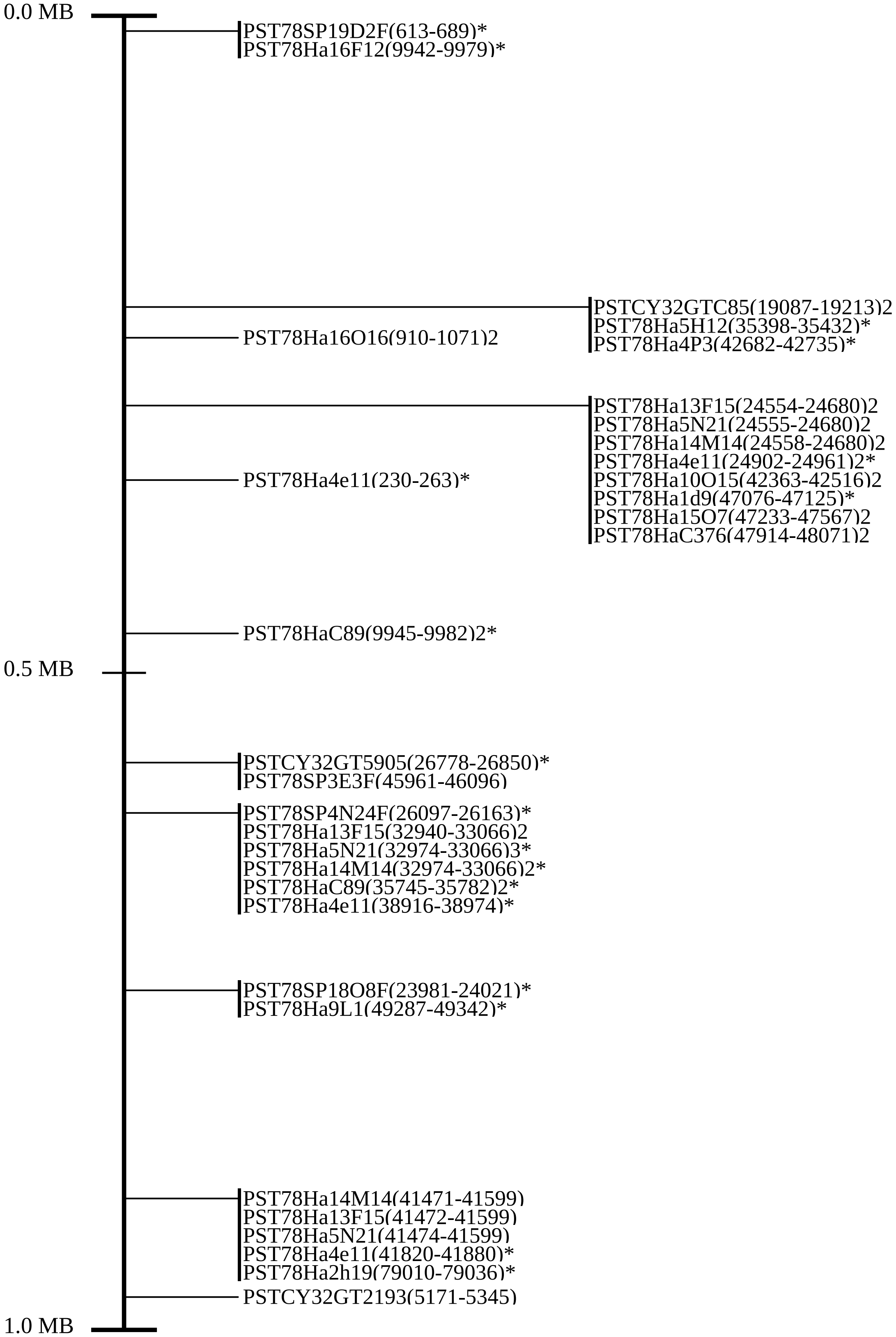

Supercontig 12 (A)
length: 1,543,397 bp

*** = less than 100bp alignment** **| = clones in the same contig**

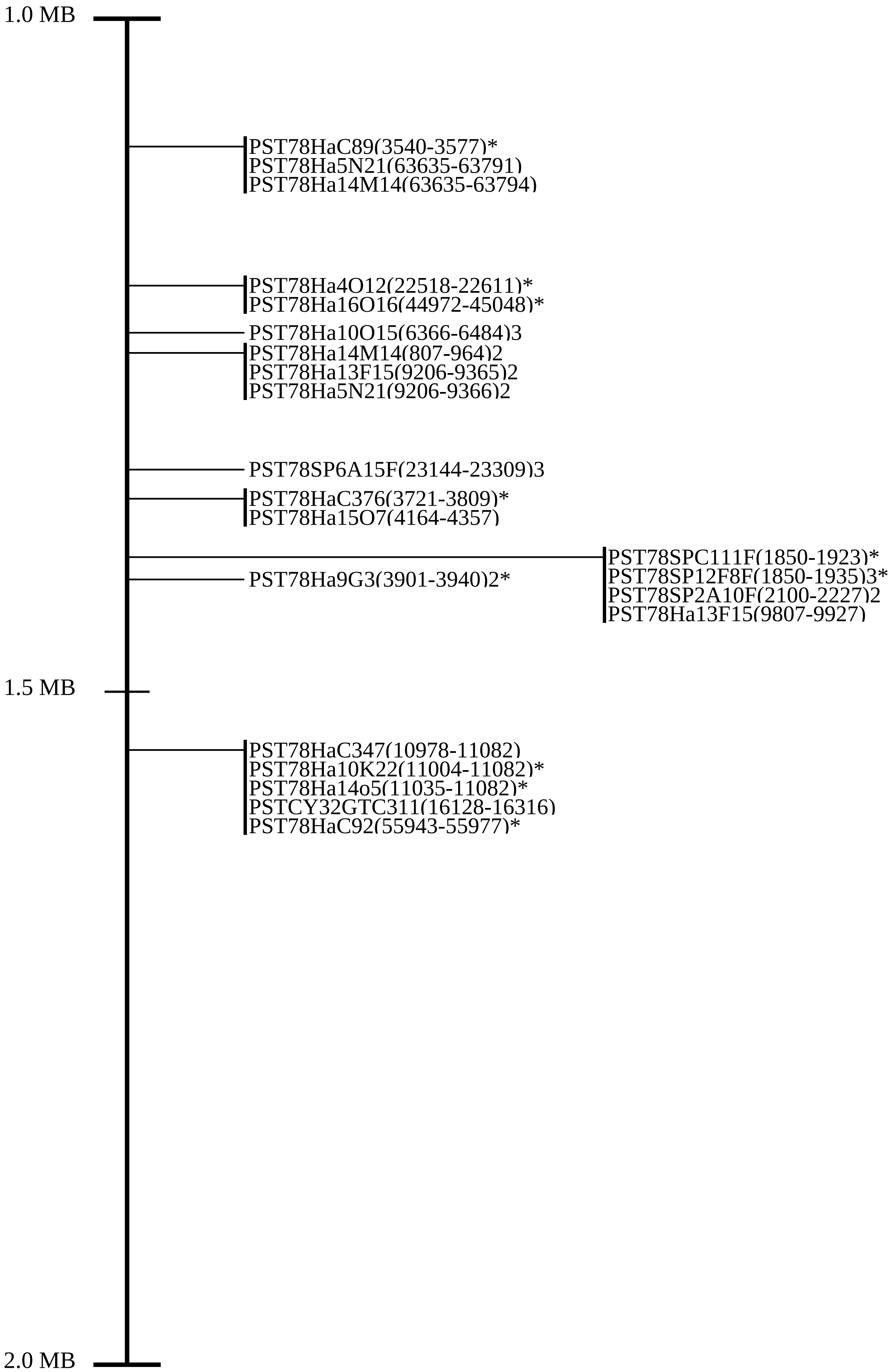

Supercontig 12 (B)
length: 1,543,397 bp

*** = less than 100bp alignment** **| = clones in the same contig**

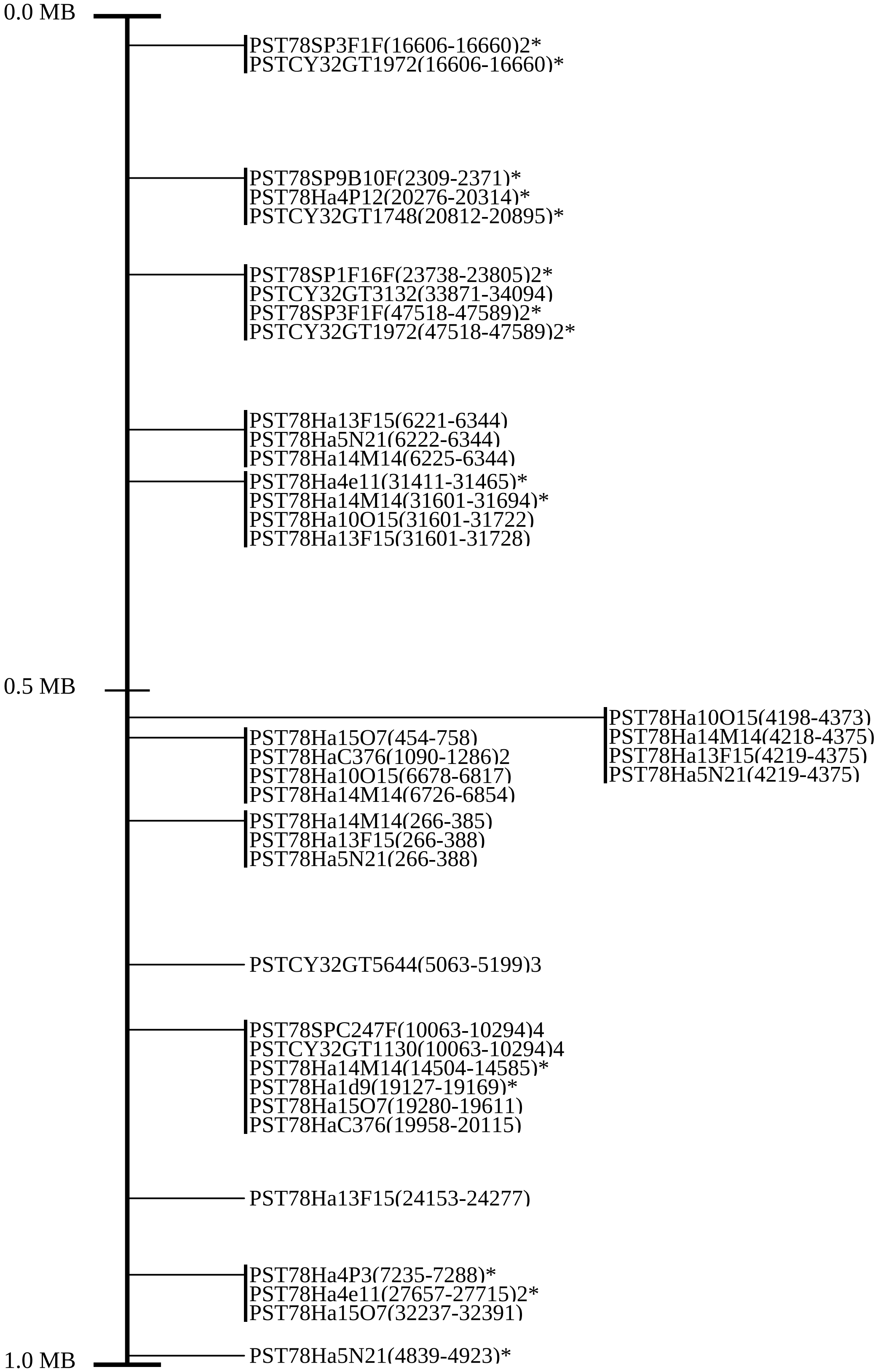

Supercontig 13 (A)
length: 1,556,540 bp

*** = less than 100bp alignment** **| = clones in the same contig**

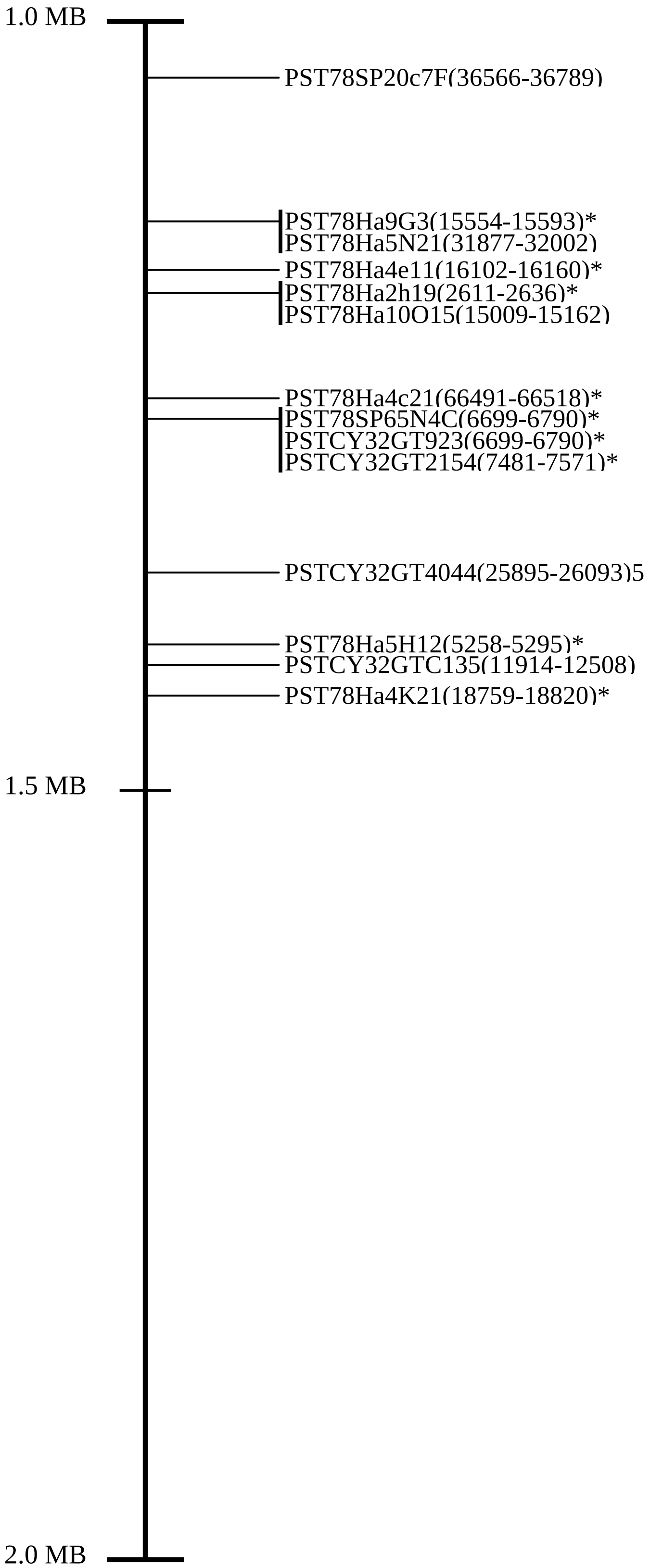

Supercontig 13 (B)
length: 1,556,540 bp

*** = less than 100bp alignment** **| = clones in the same contig**

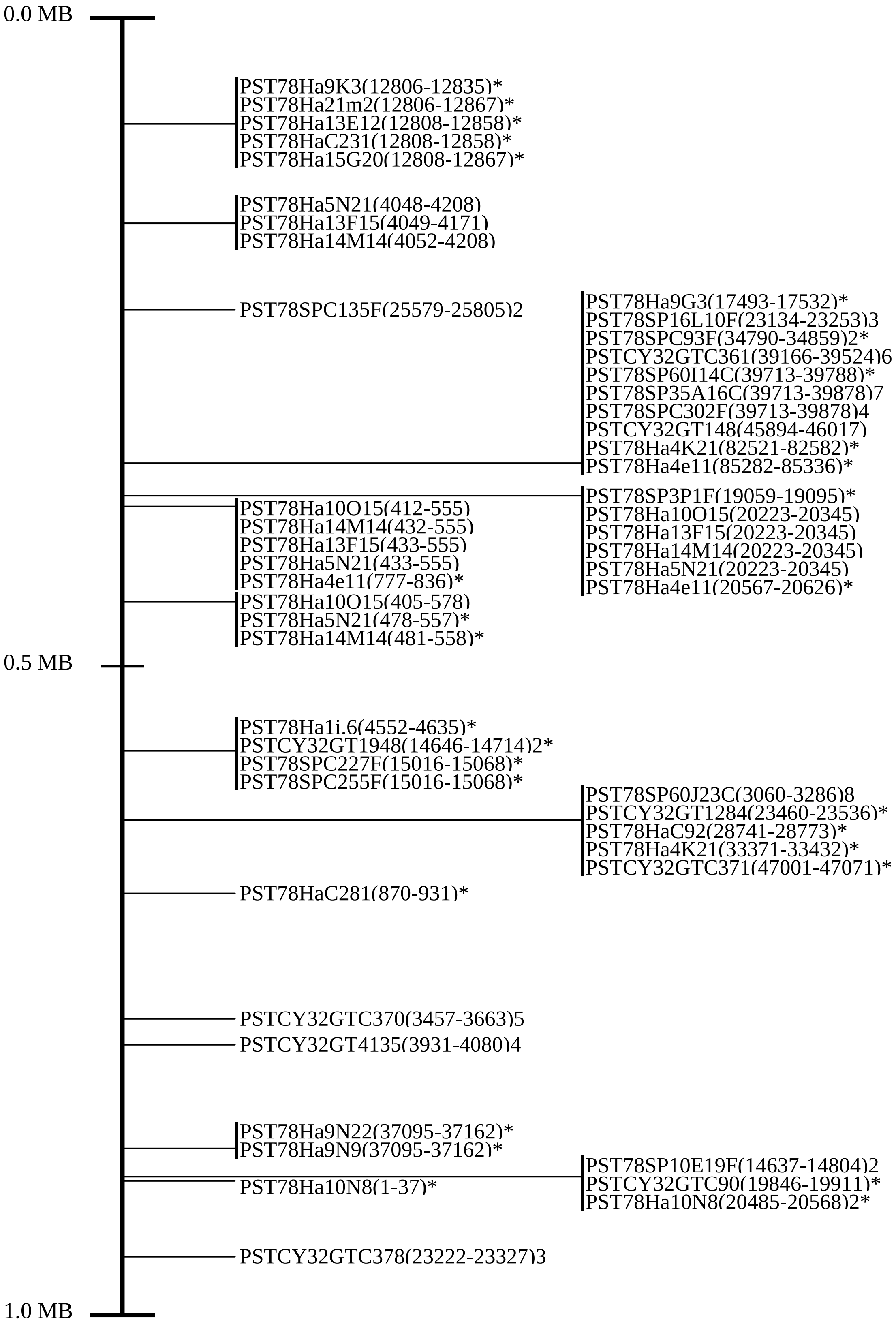

Supercontig 14 (A)
length: 1,510,324 bp

*** = less than 100bp alignment** **| = clones in the same contig**

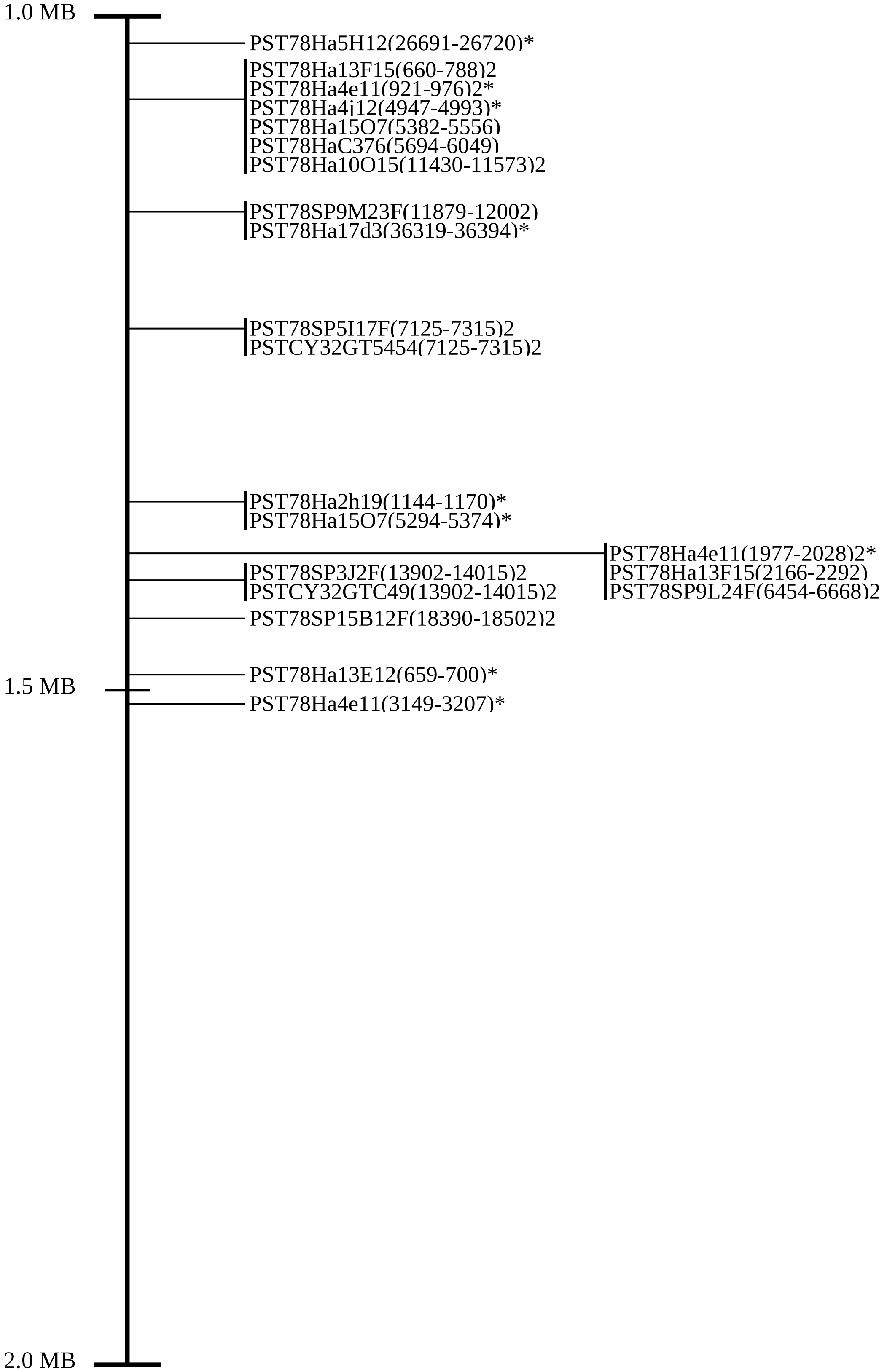

Supercontig 14 (B)
length: 1,510,324 bp

*** = less than 100bp alignment** **| = clones in the same contig**

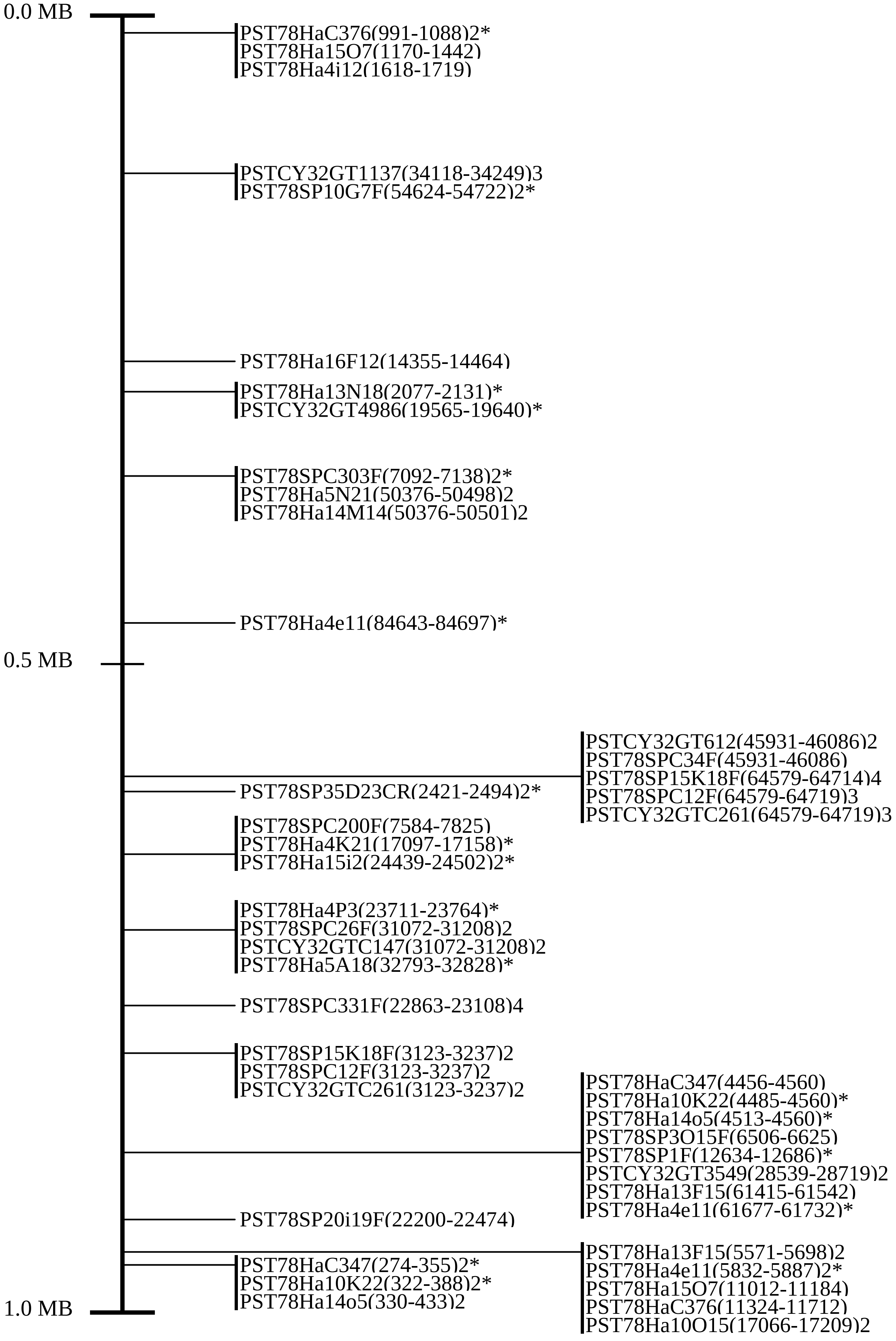

Supercontig 15 (A)
length: 1,374,611 bp

*** = less than 100bp alignment** **| = clones in the same contig**

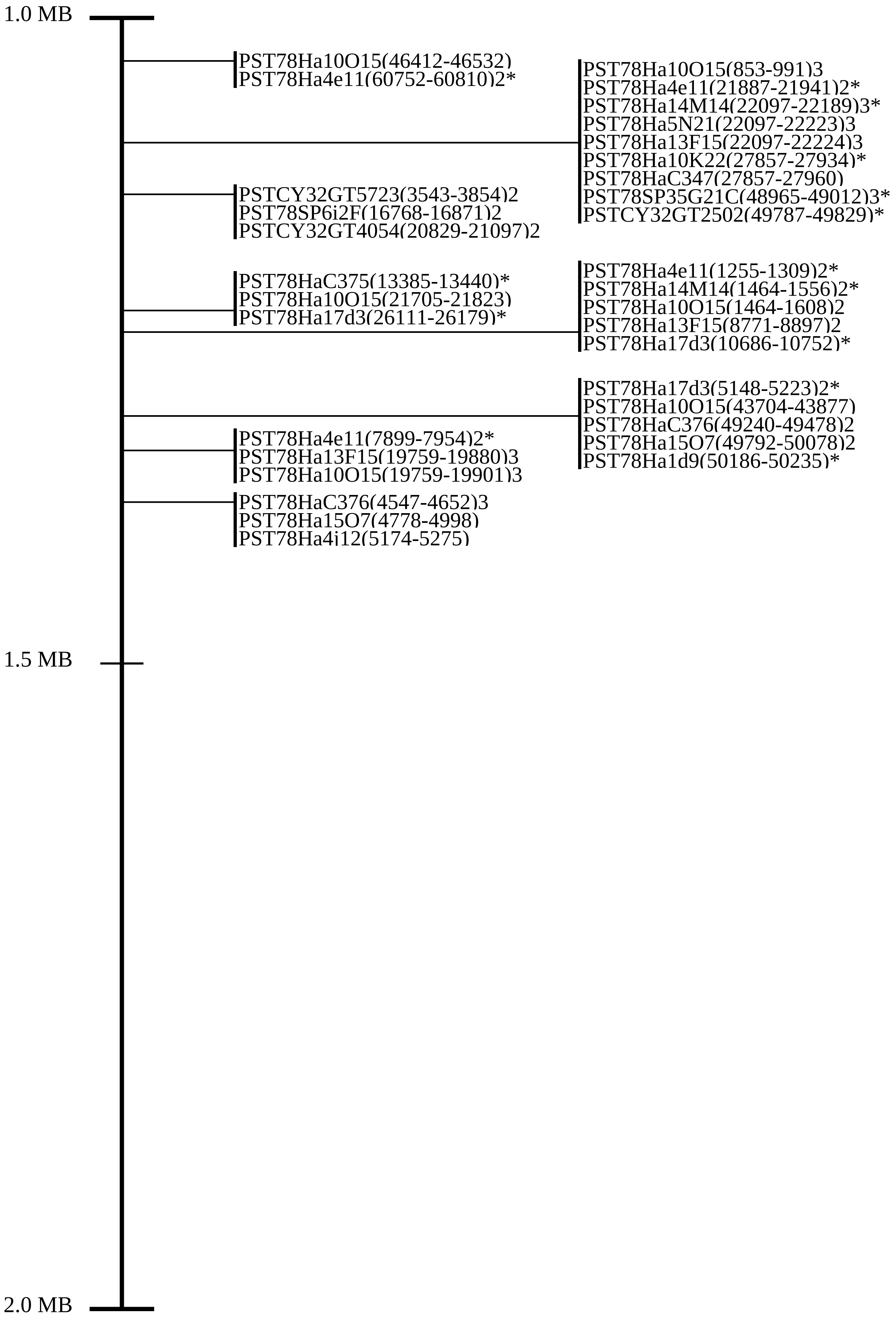

Supercontig 15 (B)
length: 1,374,611 bp

*** = less than 100bp alignment**

| = clones in the same contig

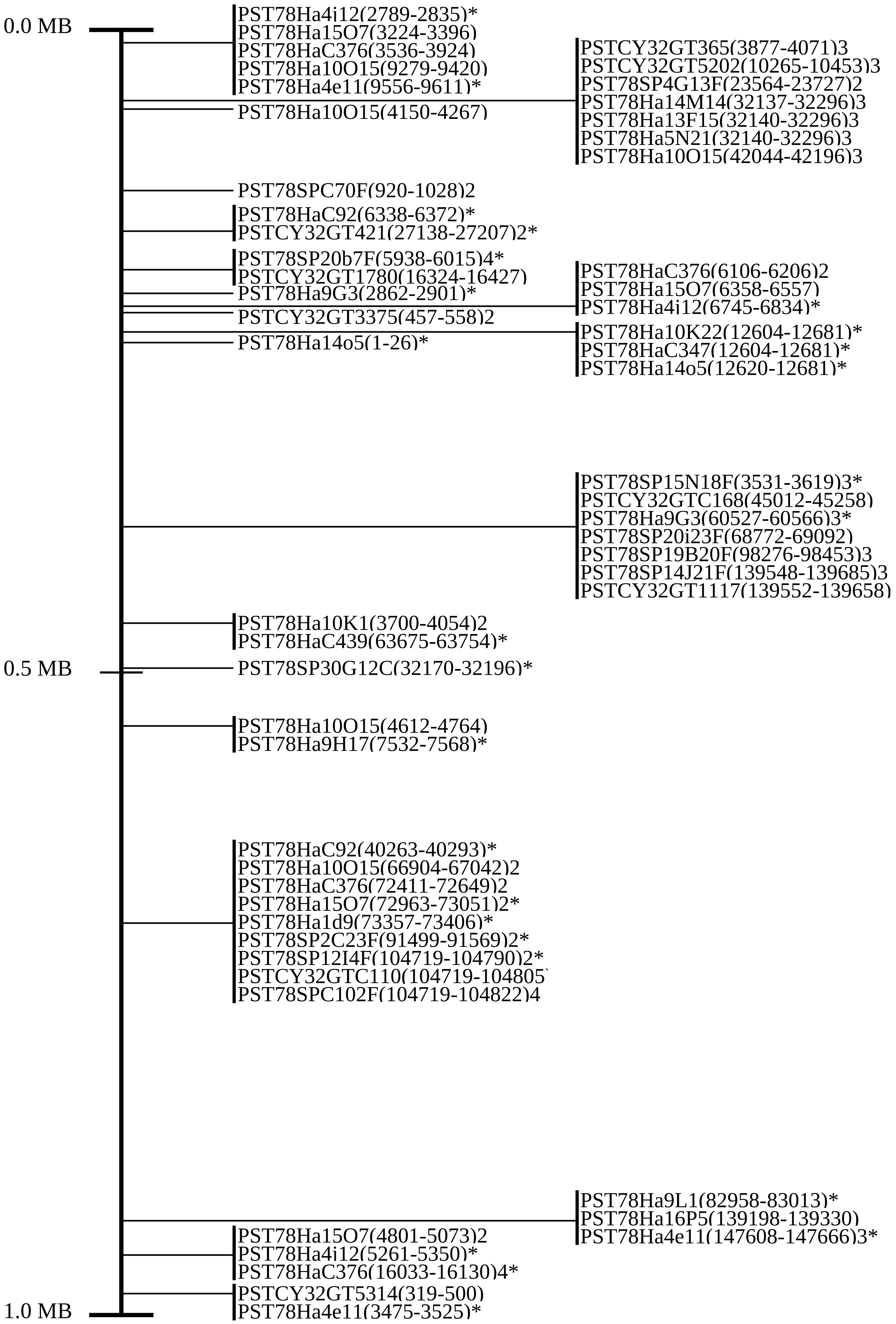

Supercontig 16 (A)
length: 1,217,956 bp

*** = less than 100bp alignment** **| = clones in the same contig**

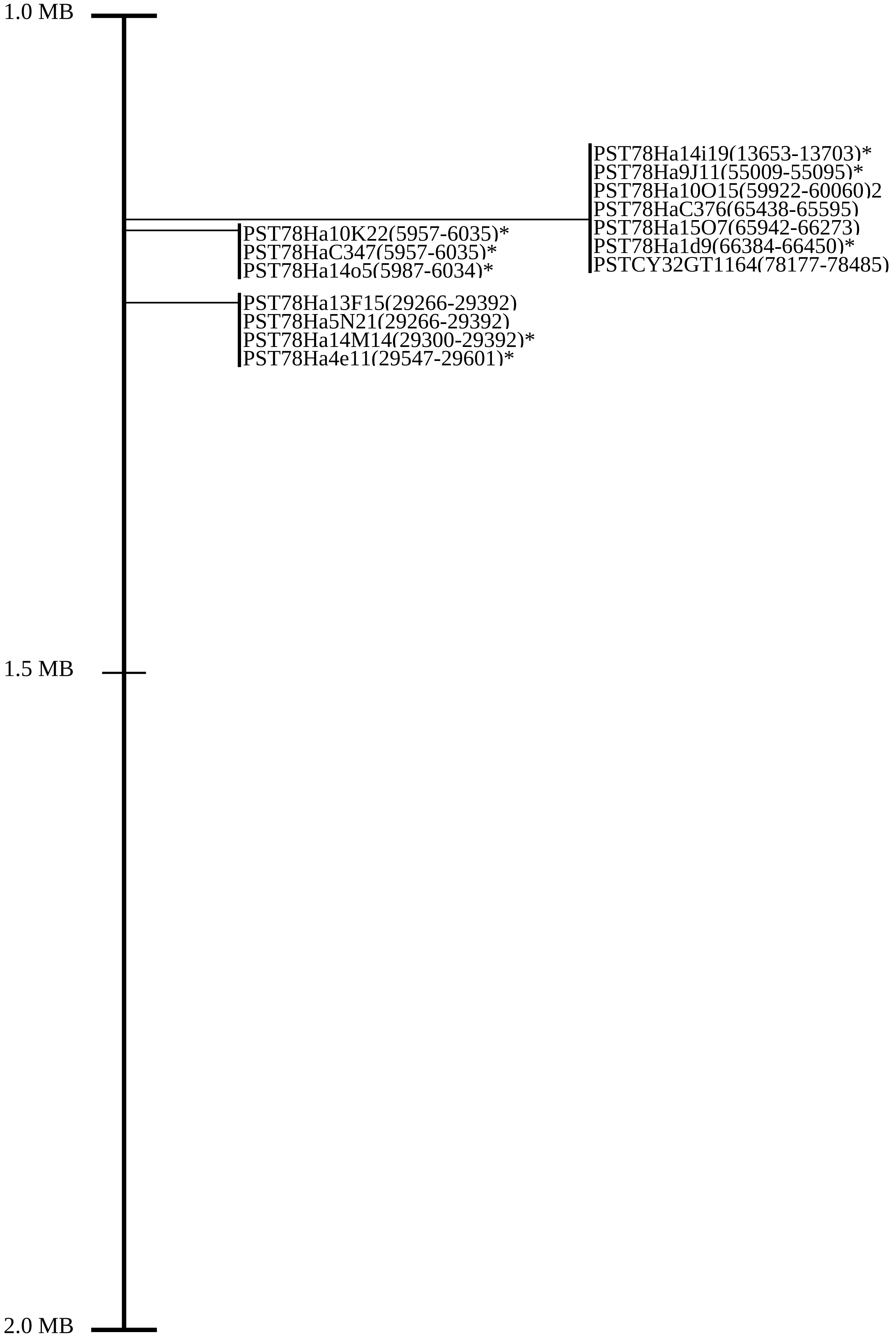

Supercontig 16 (B)
length: 1,217,956 bp

*** = less than 100bp alignment**

| = clones in the same contig

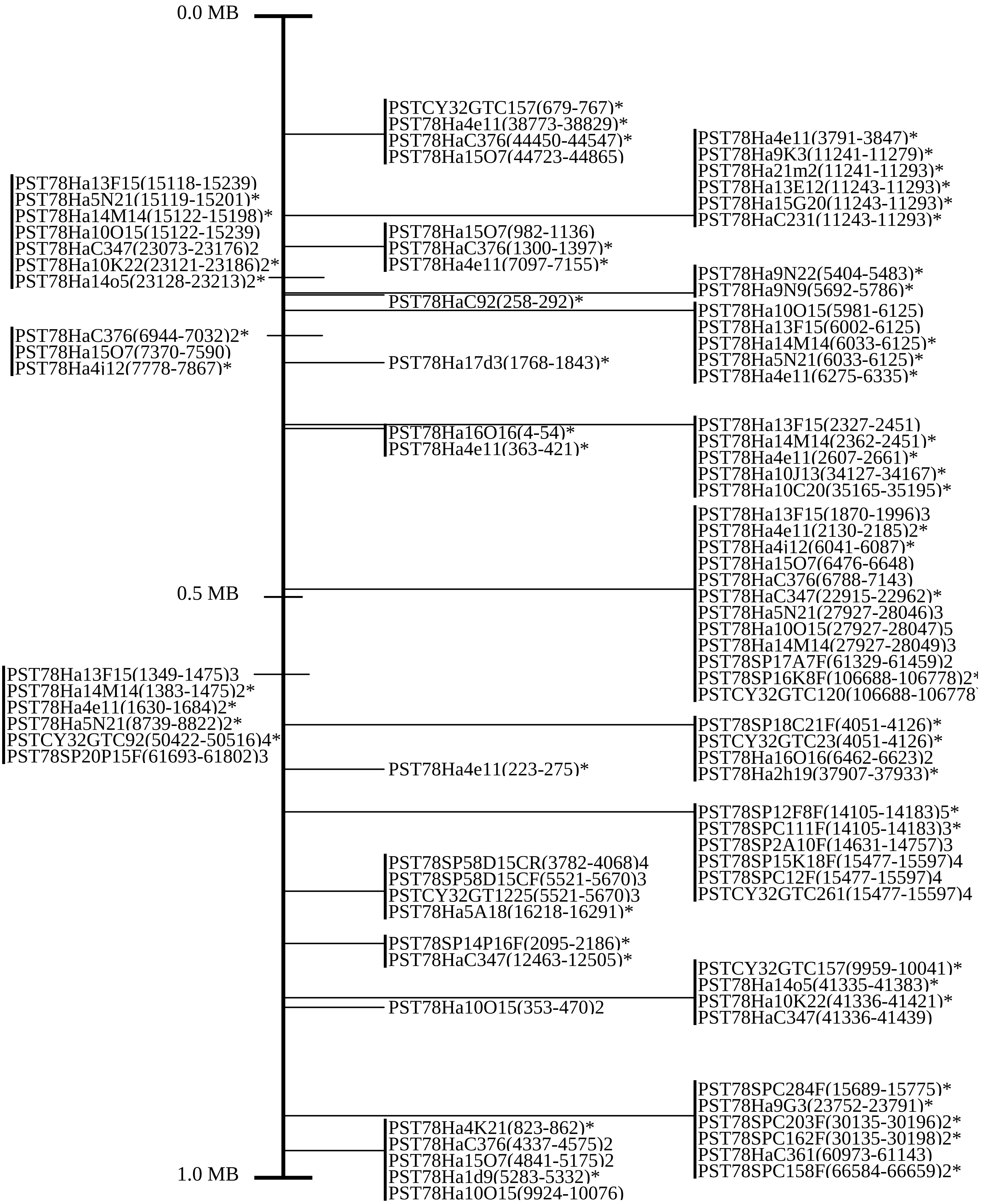

Supercontig 17 (A)
length: 1,242,959 bp

*** = less than 100bp alignment** | **| = clones in the same contig**

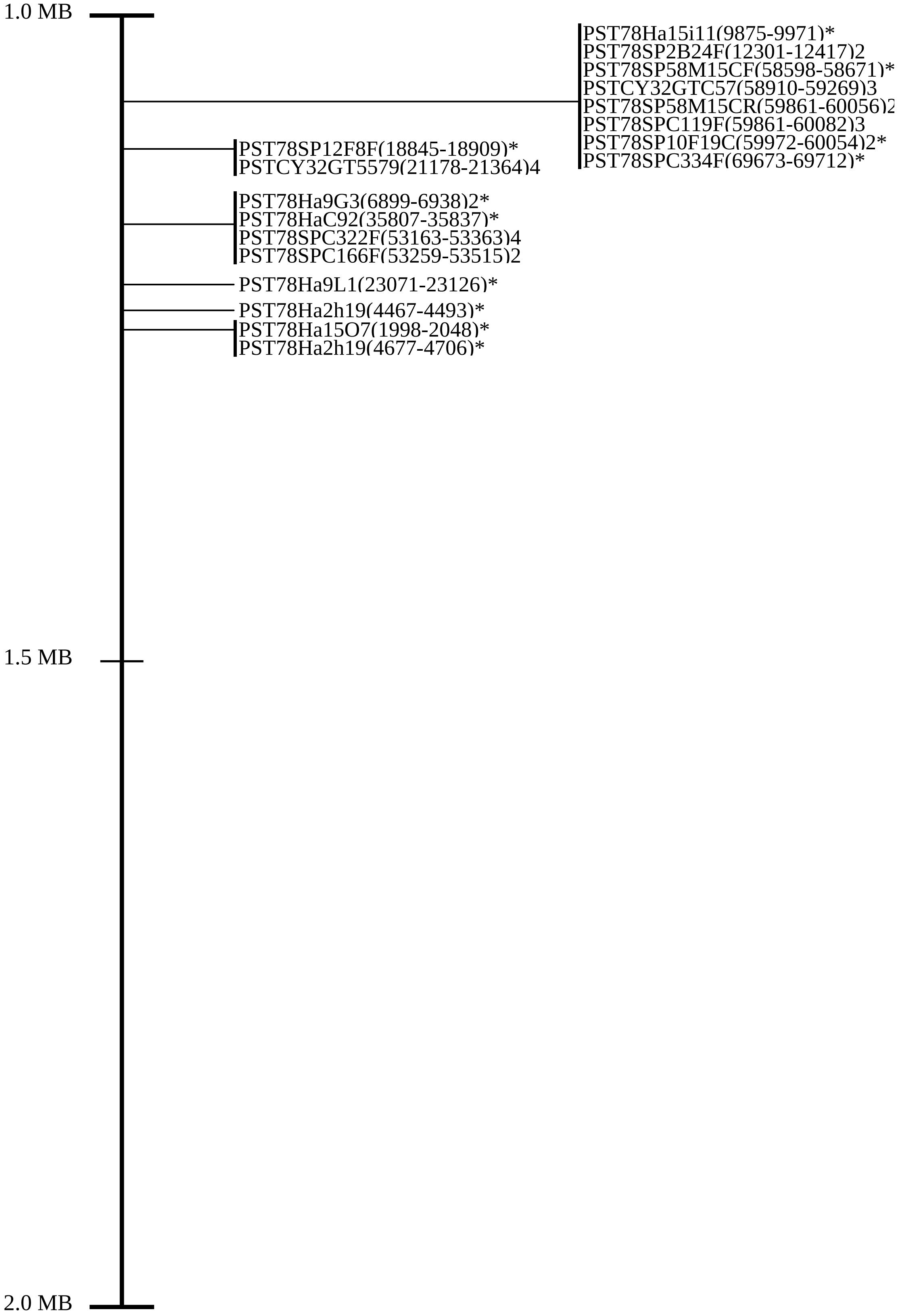

Supercontig 17 (B)
length: 1,242,959 bp

*** = less than 100bp alignment** **| = clones in the same contig**

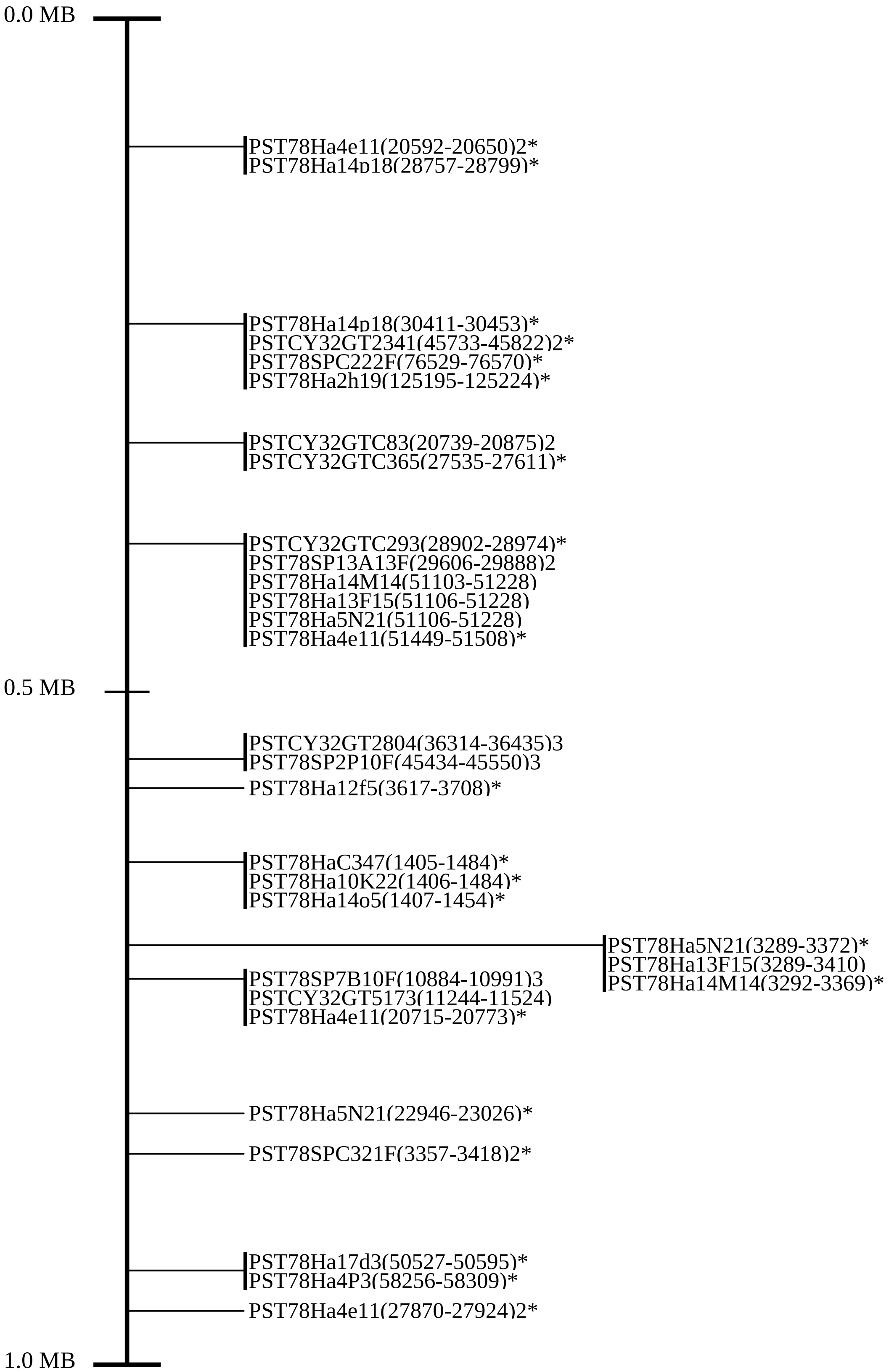

Supercontig 18 (A)
length: 1,195,459 bp

*** = less than 100bp alignment** **| = clones in the same contig**

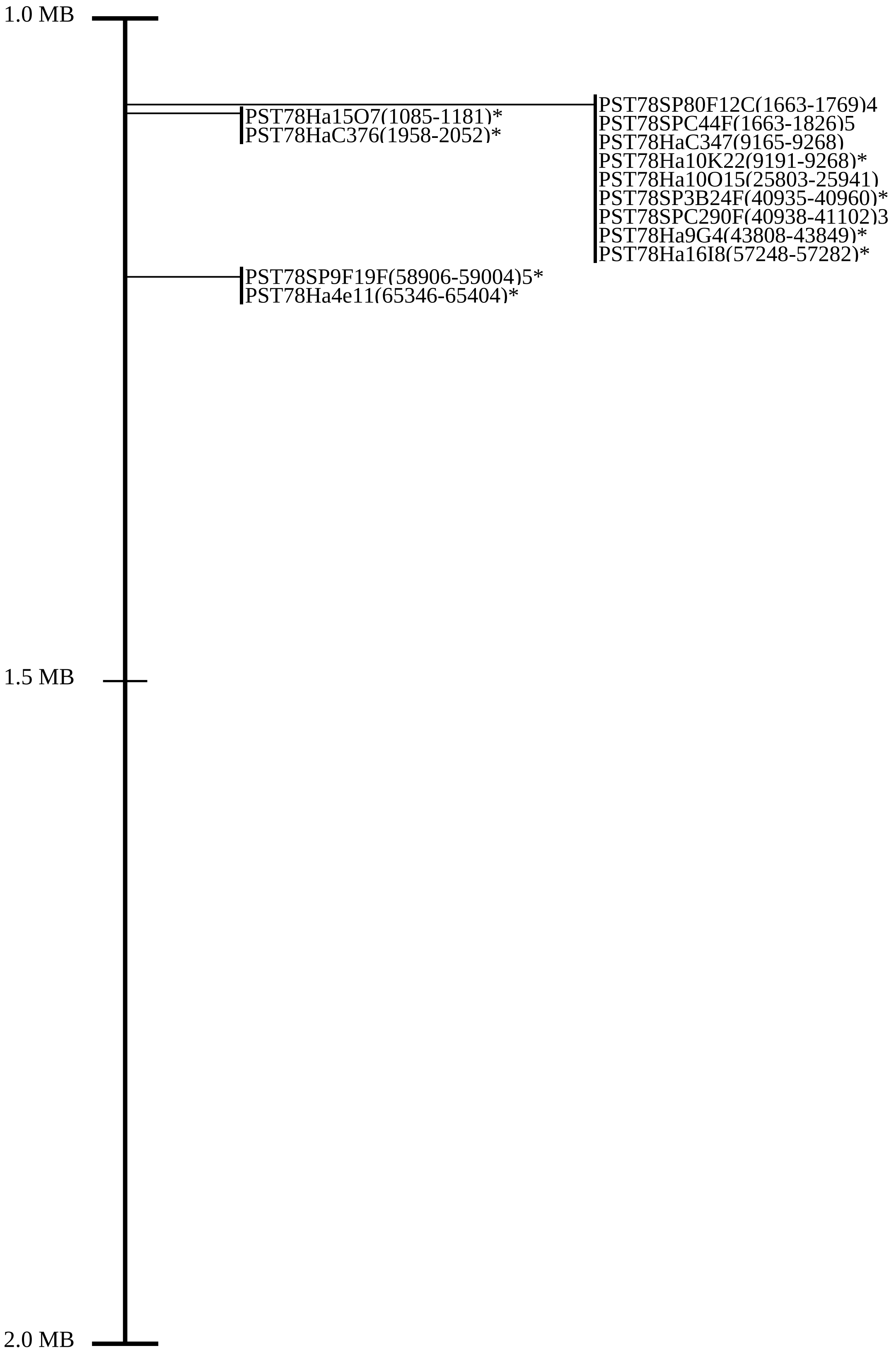

Supercontig 18 (B)
length: 1,195,459 bp

*** = less than 100bp alignment** **| = clones in the same contig**

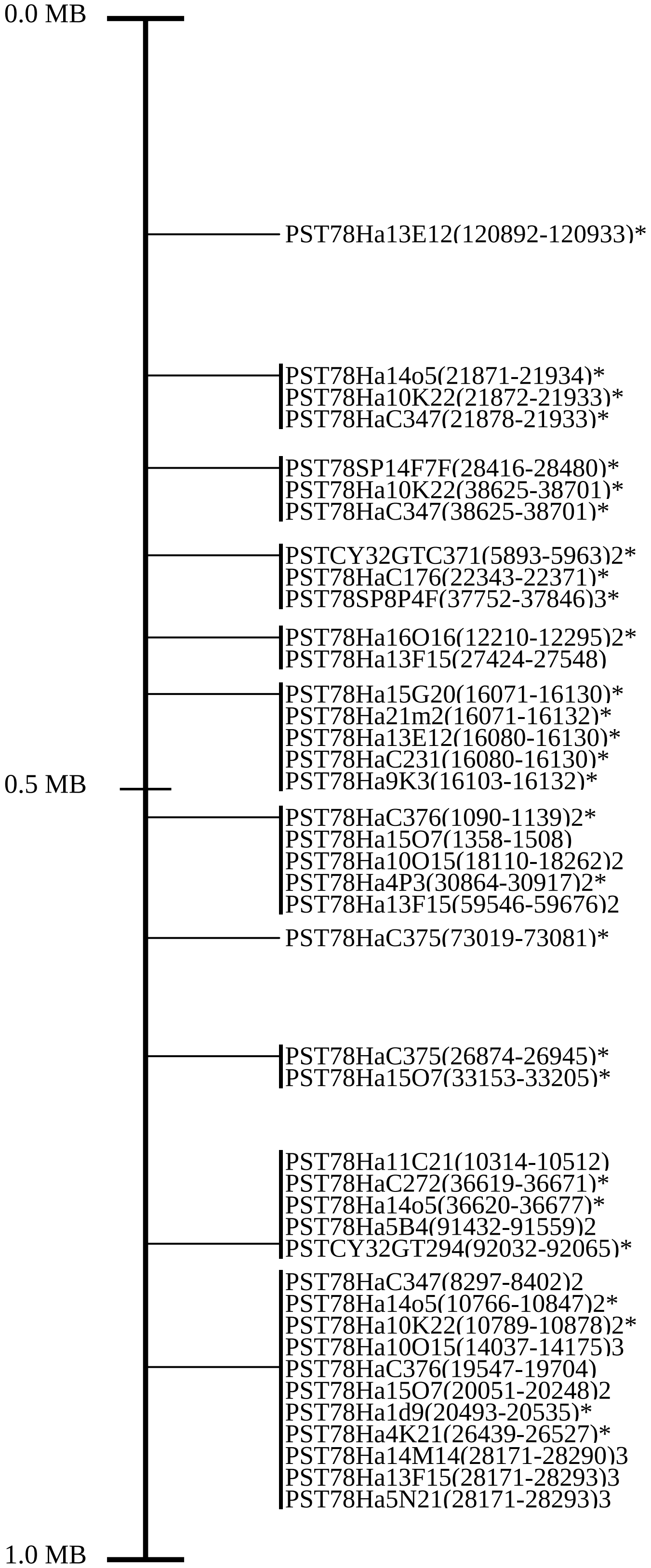

Supercontig 19
length: 1,137,327 bp

*** = less than 100bp alignment** **| = clones in the same contig**

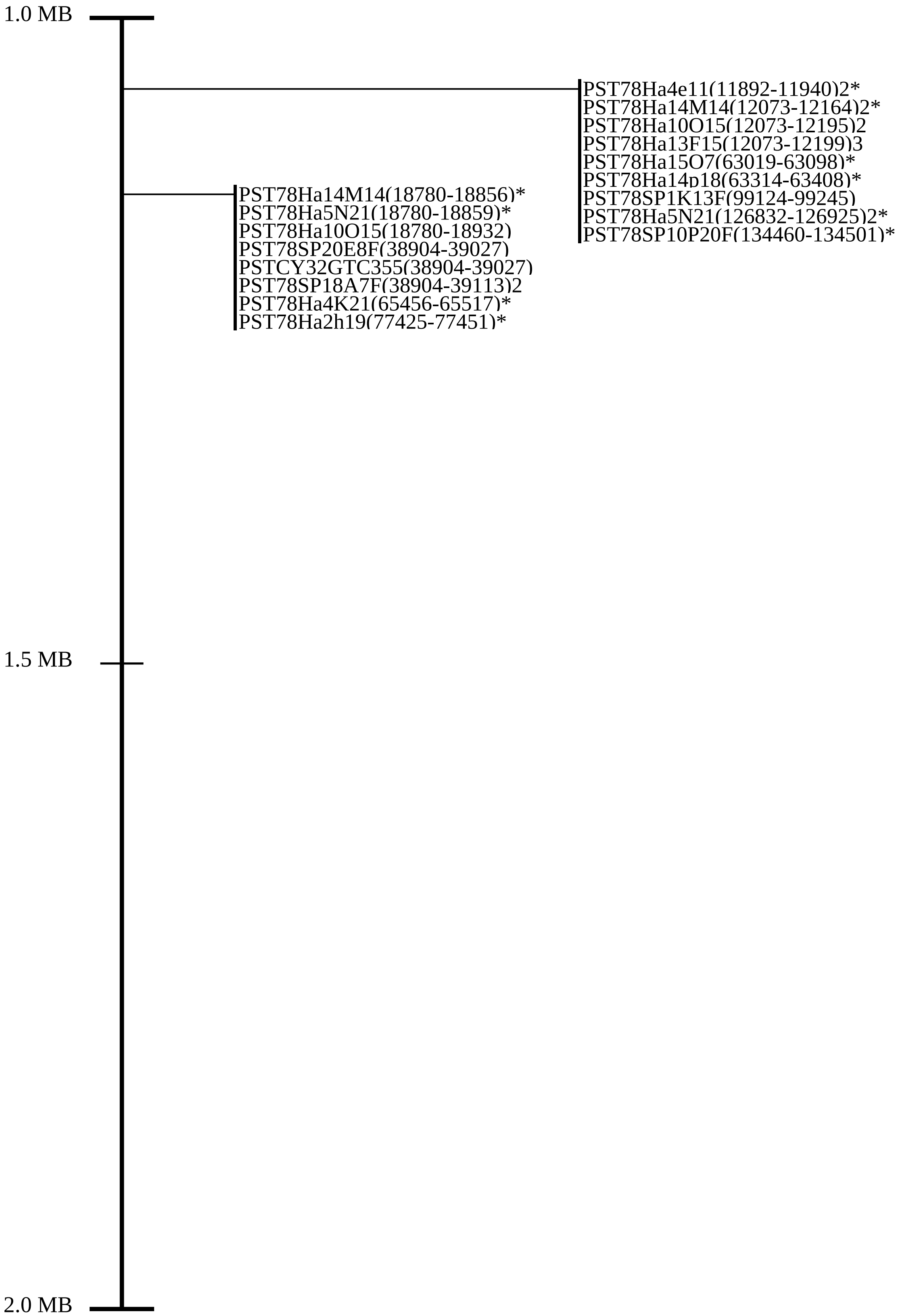

Supercontig 19 (B)
length: 1,137,327 bp

*** = less than 100bp alignment** **| = clones in the same contig**

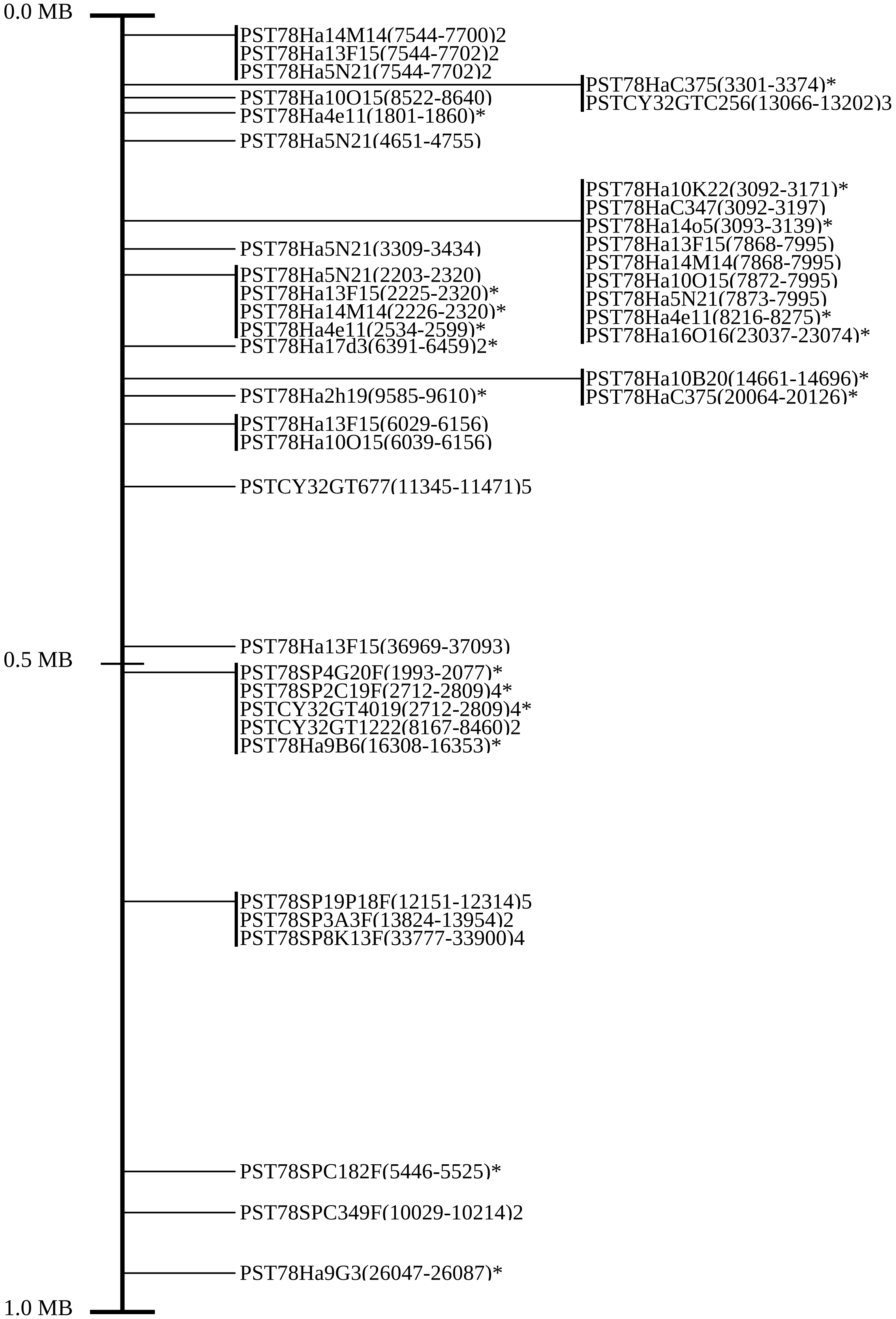

Supercontig 20 (A)
length: 1,198,131 bp

*** = less than 100bp alignment** **| = clones in the same contig**

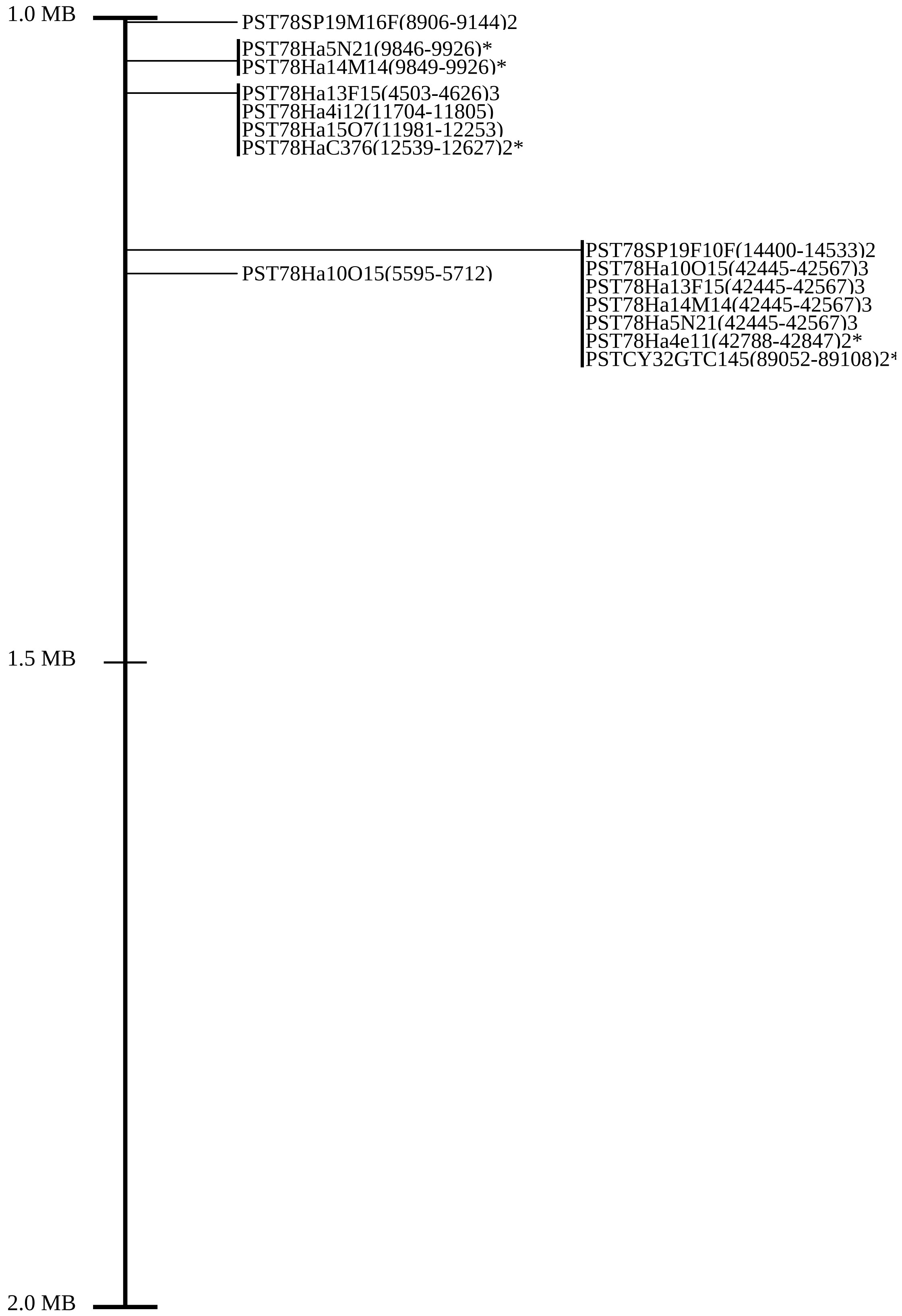

Supercontig 20 (B)
length: 1,198,131 bp

*** = less than 100bp alignment** **| = clones in the same contig**

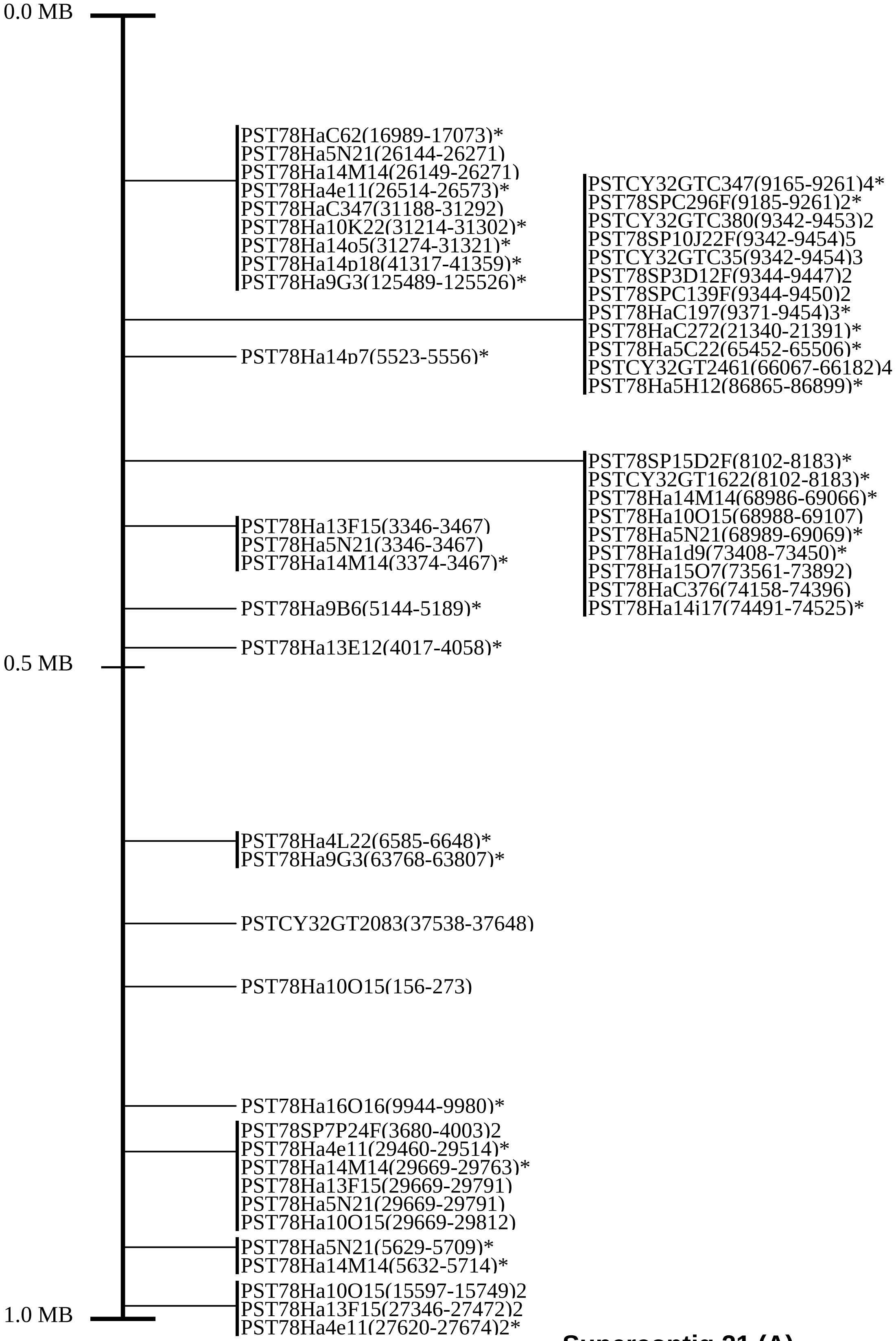

Supercontig 21 (A)
length: 1,084,580 bp

*** = less than 100bp alignment** **| = clones in the same contig**

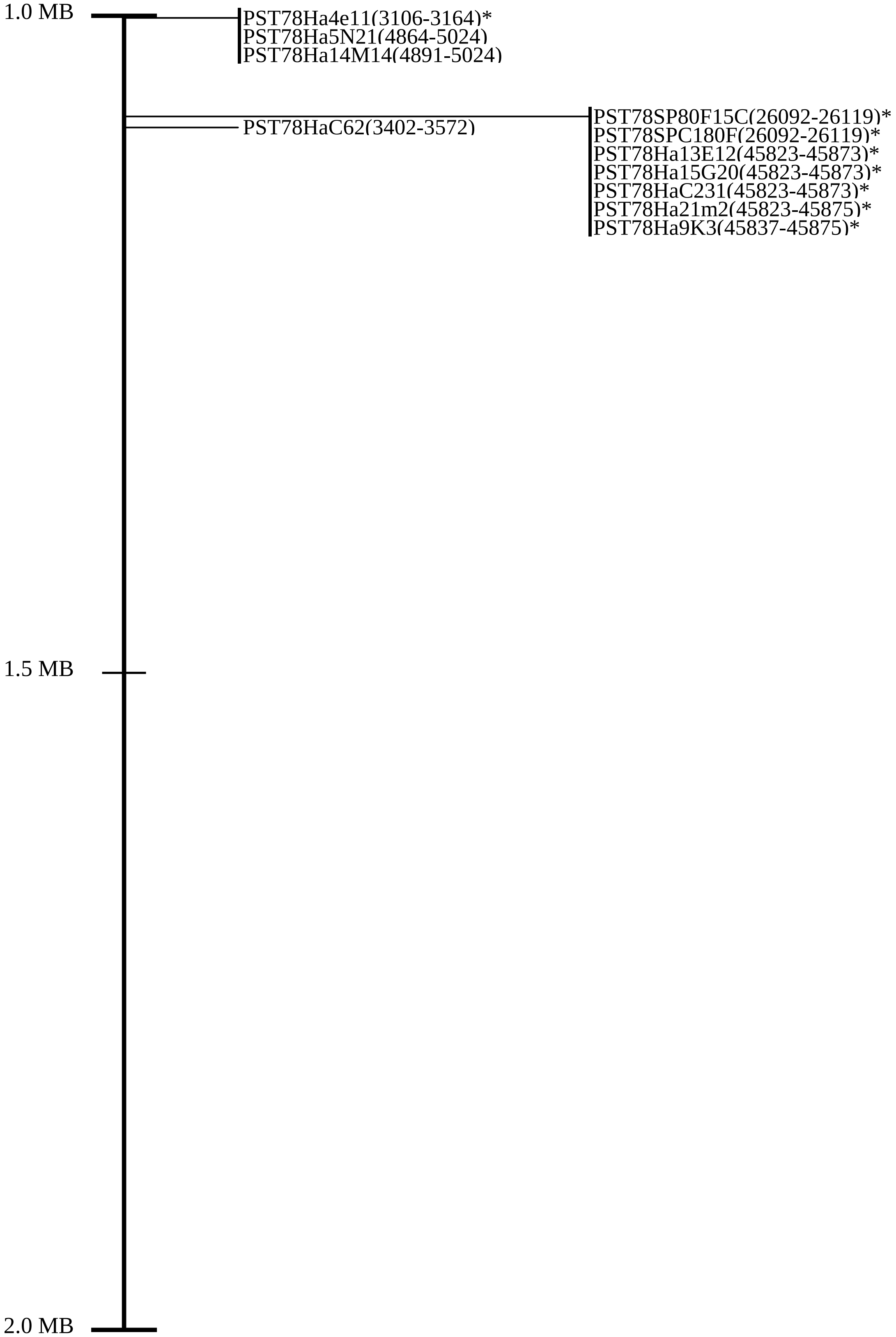

Supercontig 21 (B)
length: 1,084,580 bp

*** = less than 100bp alignment** **| = clones in the same contig**

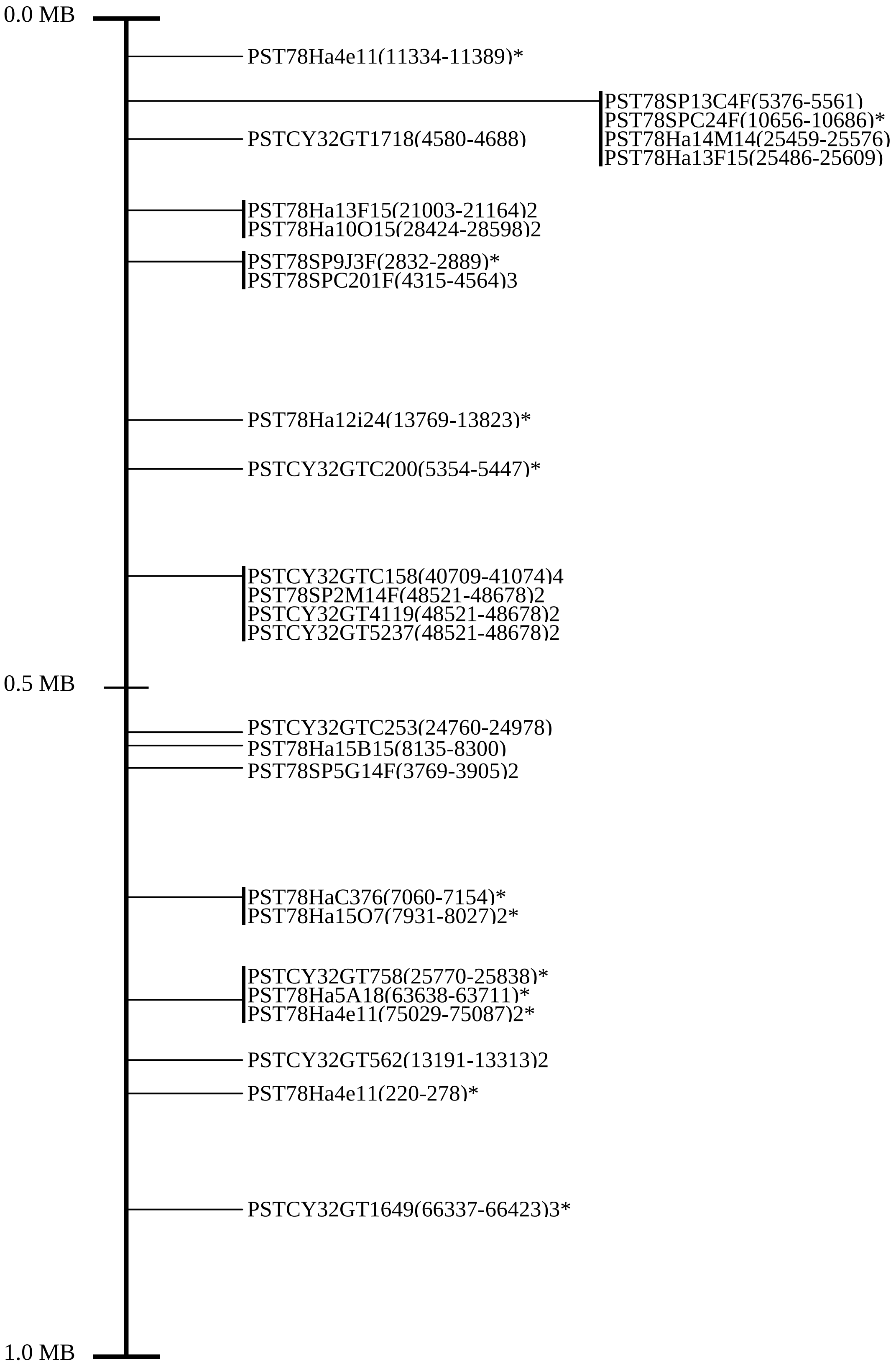

Supercontig 22 (A)
length: 1,051,806 bp

*** = less than 100bp alignment** **| = clones in the same contig**

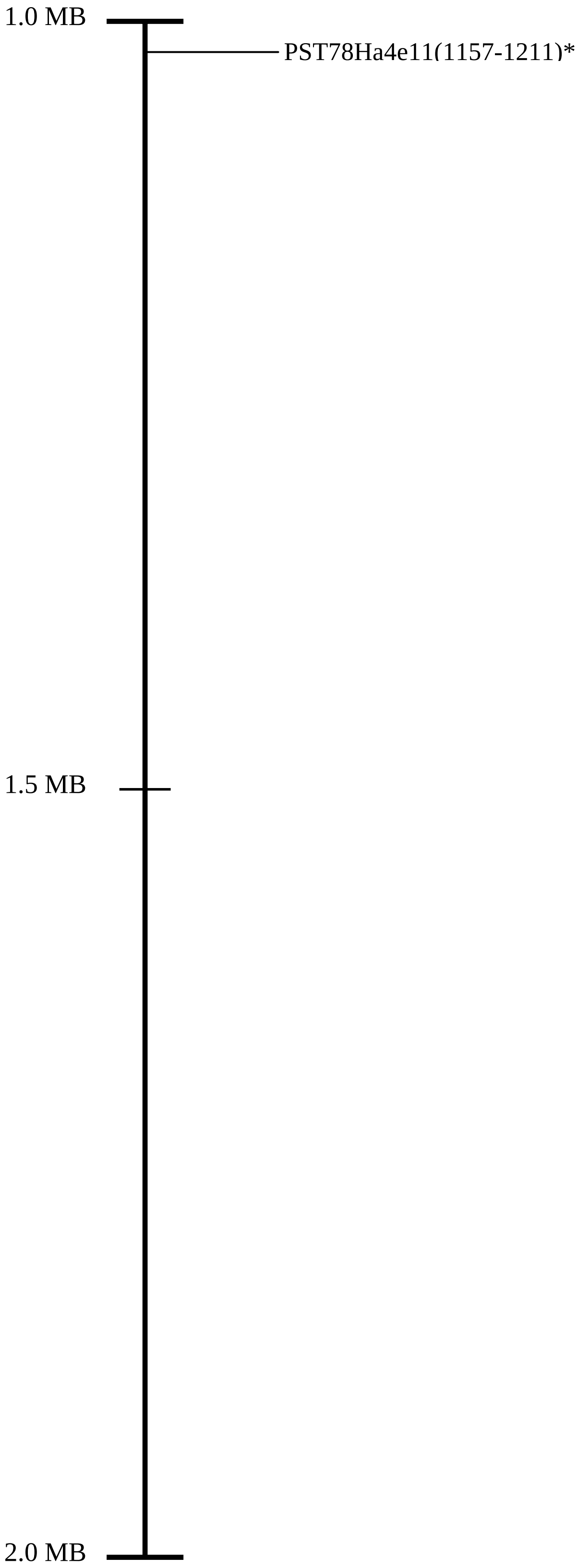

Supercontig 22 (B)
length: 1,051,806 bp

*** = less than 100bp alignment**

| = clones in the same contig

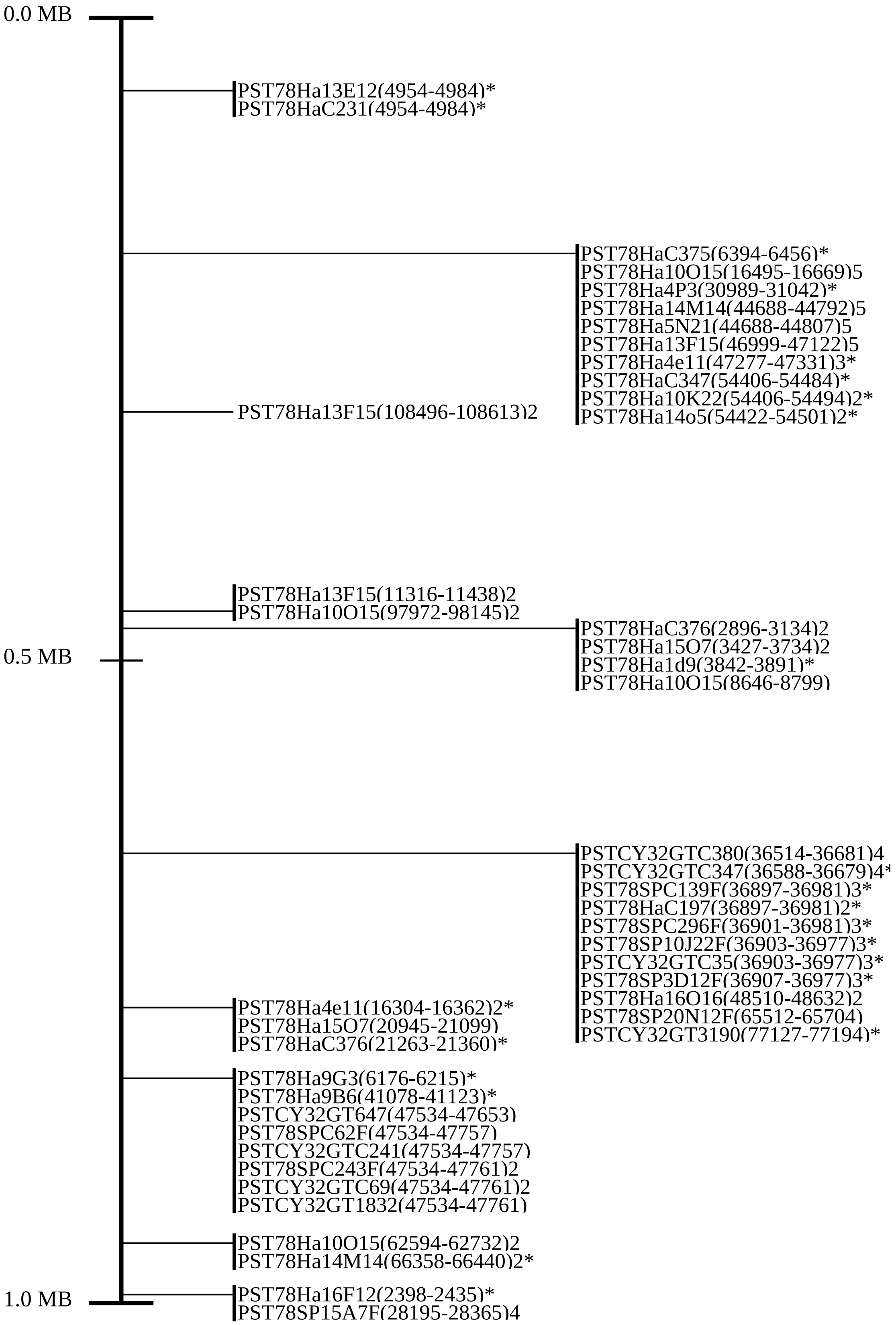

Supercontig 23
length: 1,003,138 bp

*** = less than 100bp alignment** **| = clones in the same contig**

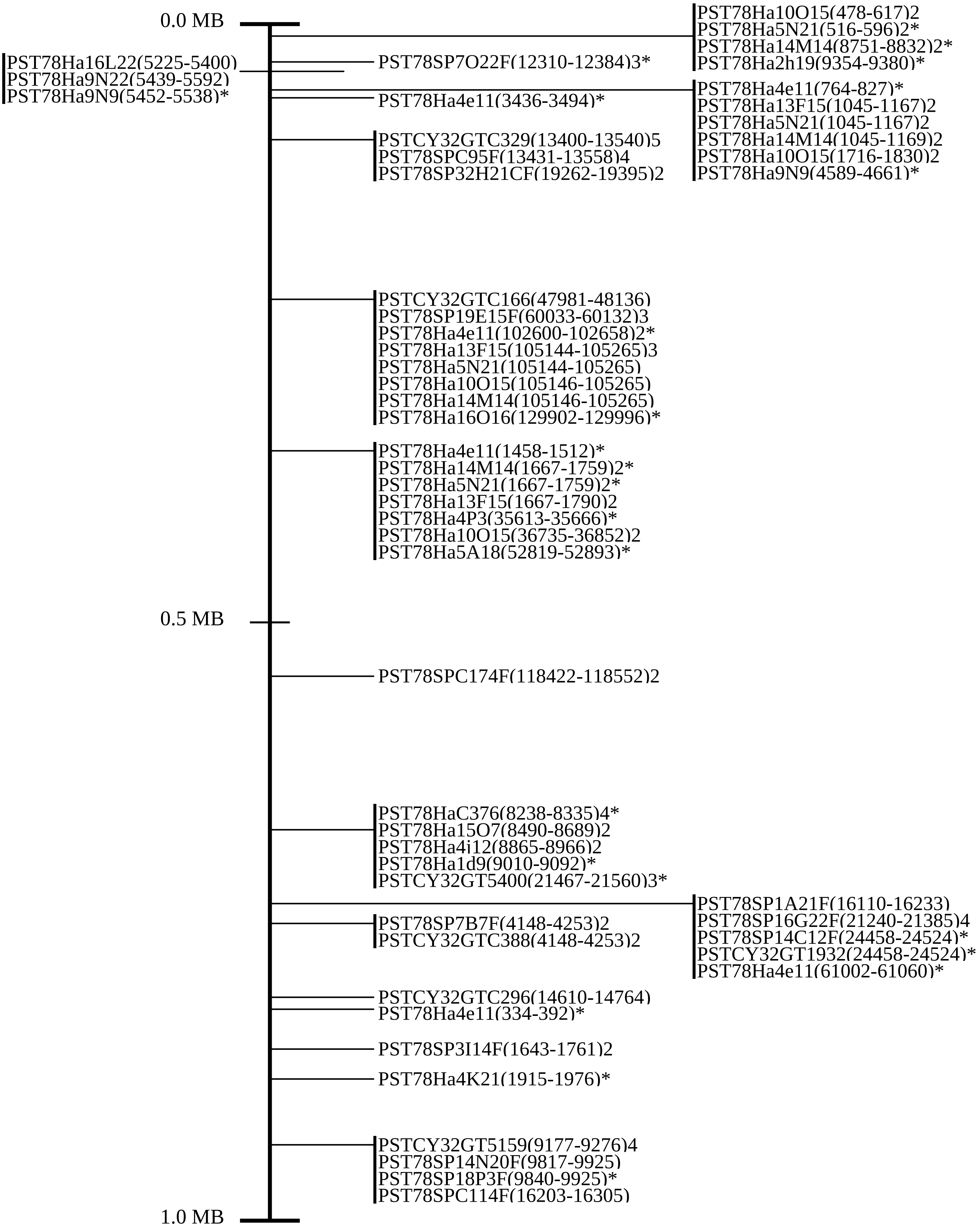

Supercontig 24 (A)
length: 1,008,357 bp

*** = less than 100bp alignment** **| = clones in the same contig**

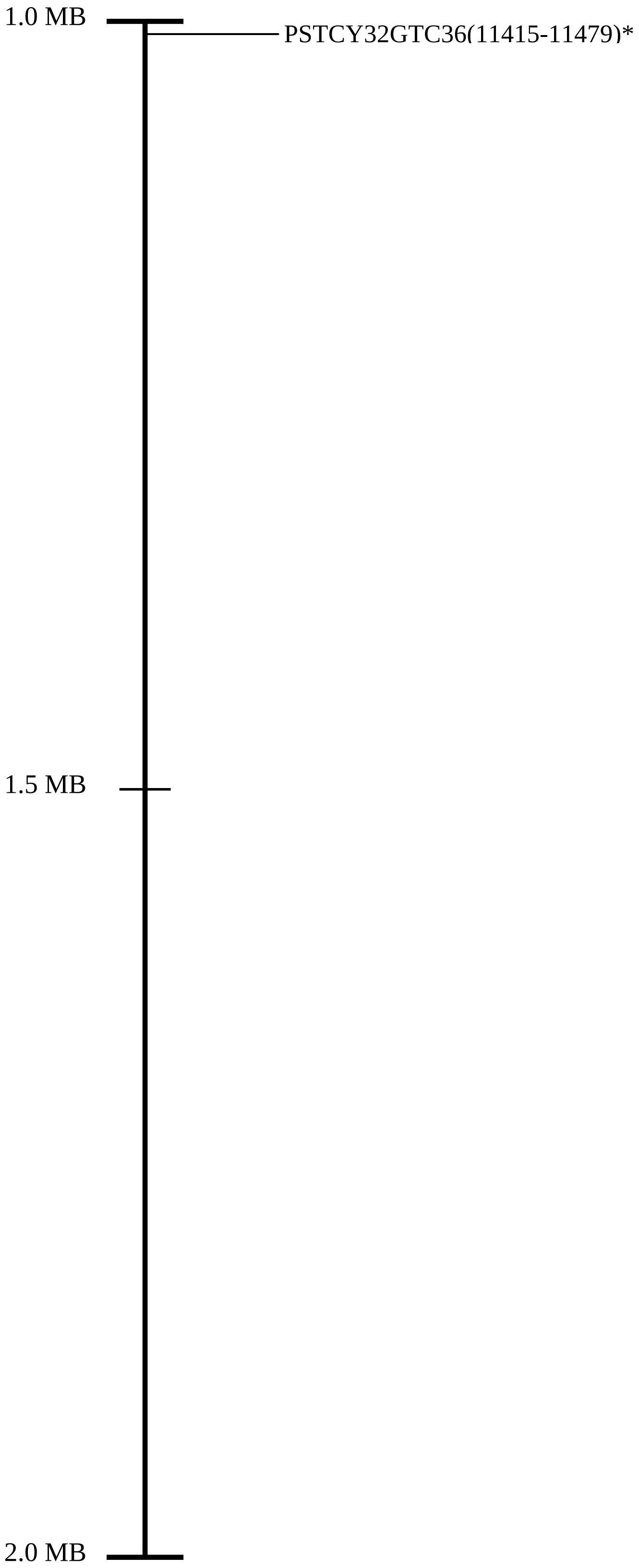

Supercontig 24 (B)
length: 1,008,357 bp

*** = less than 100bp alignment**

| = clones in the same contig

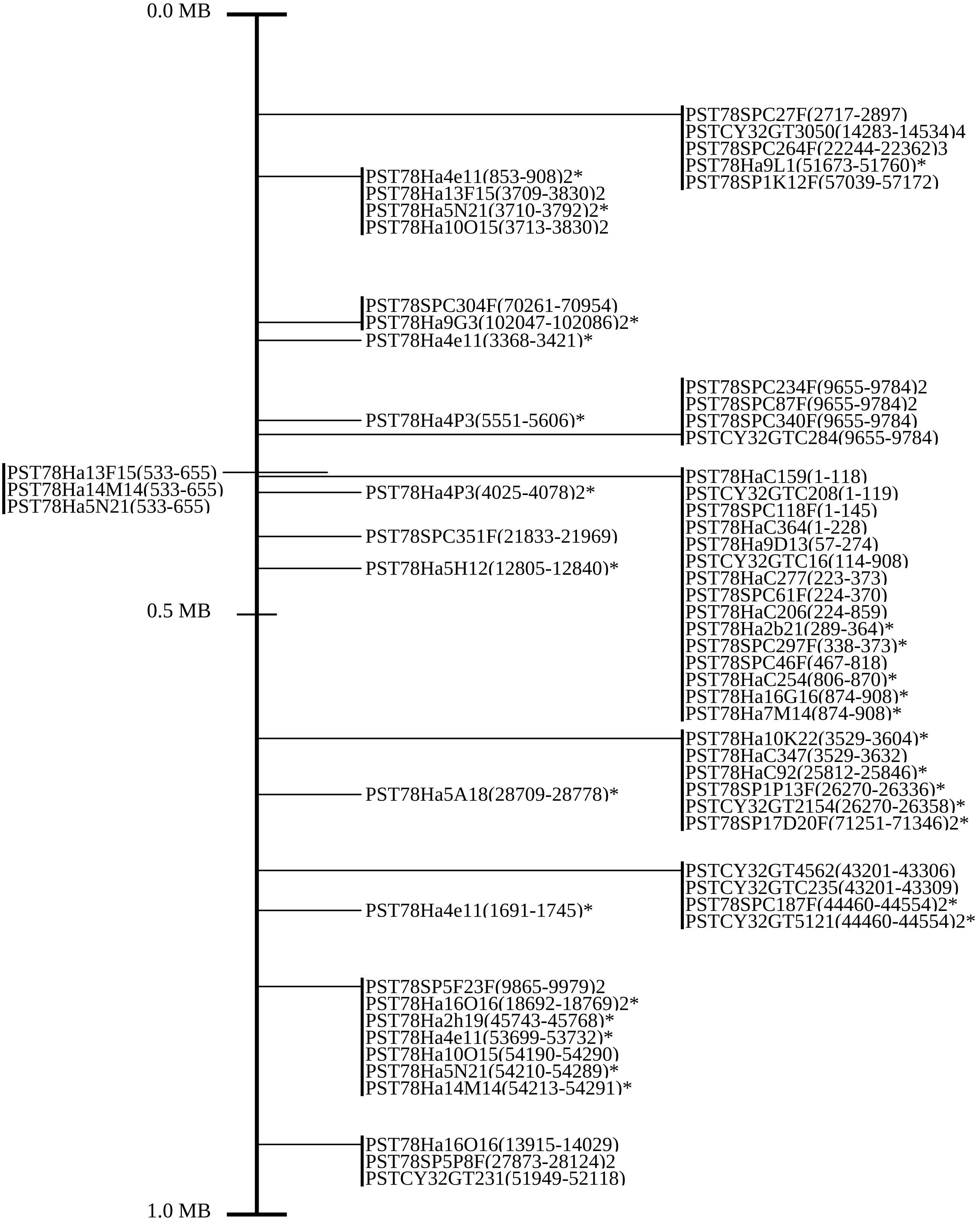

Supercontig 25 (A)
length: 1,068,291 bp

*** = less than 100bp alignment** **| = clones in the same contig**

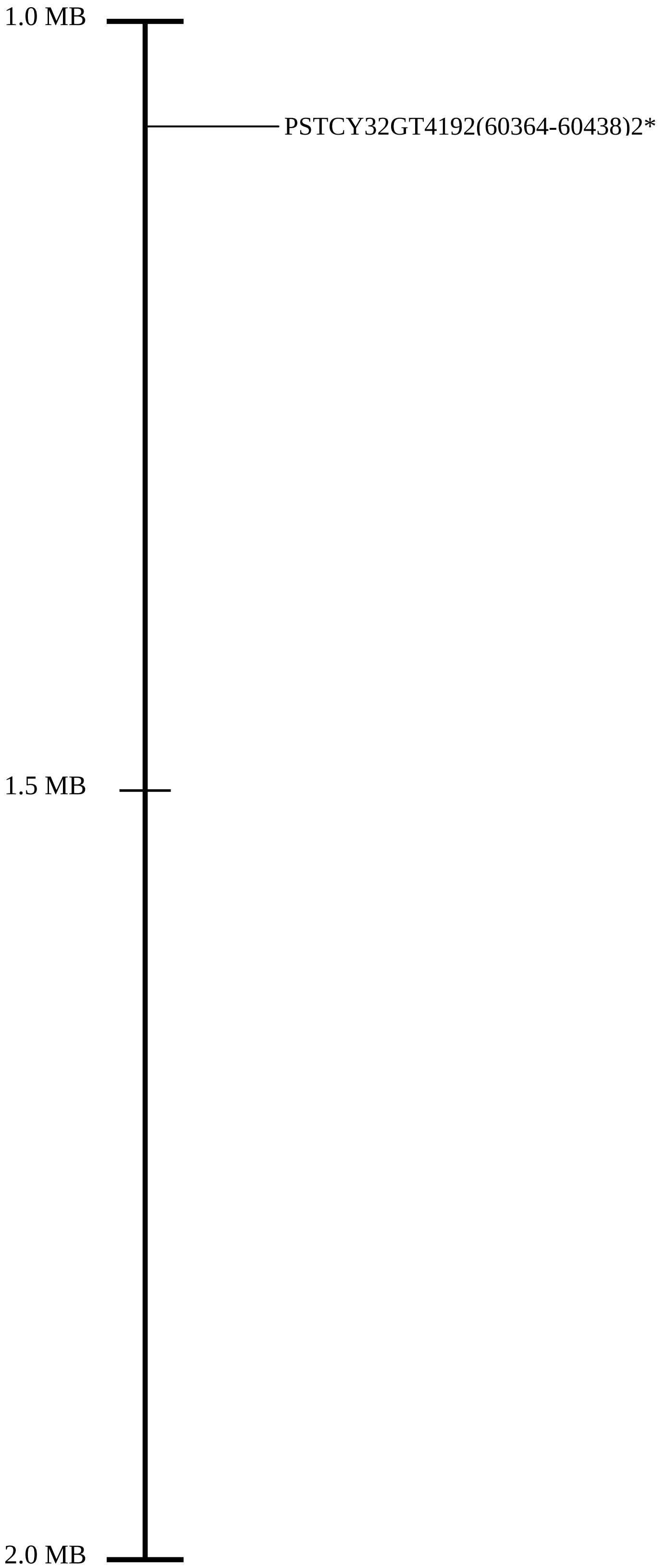

Supercontig 25 (B)
length: 1,068,291 bp

*** = less than 100bp alignment**

| = clones in the same contig

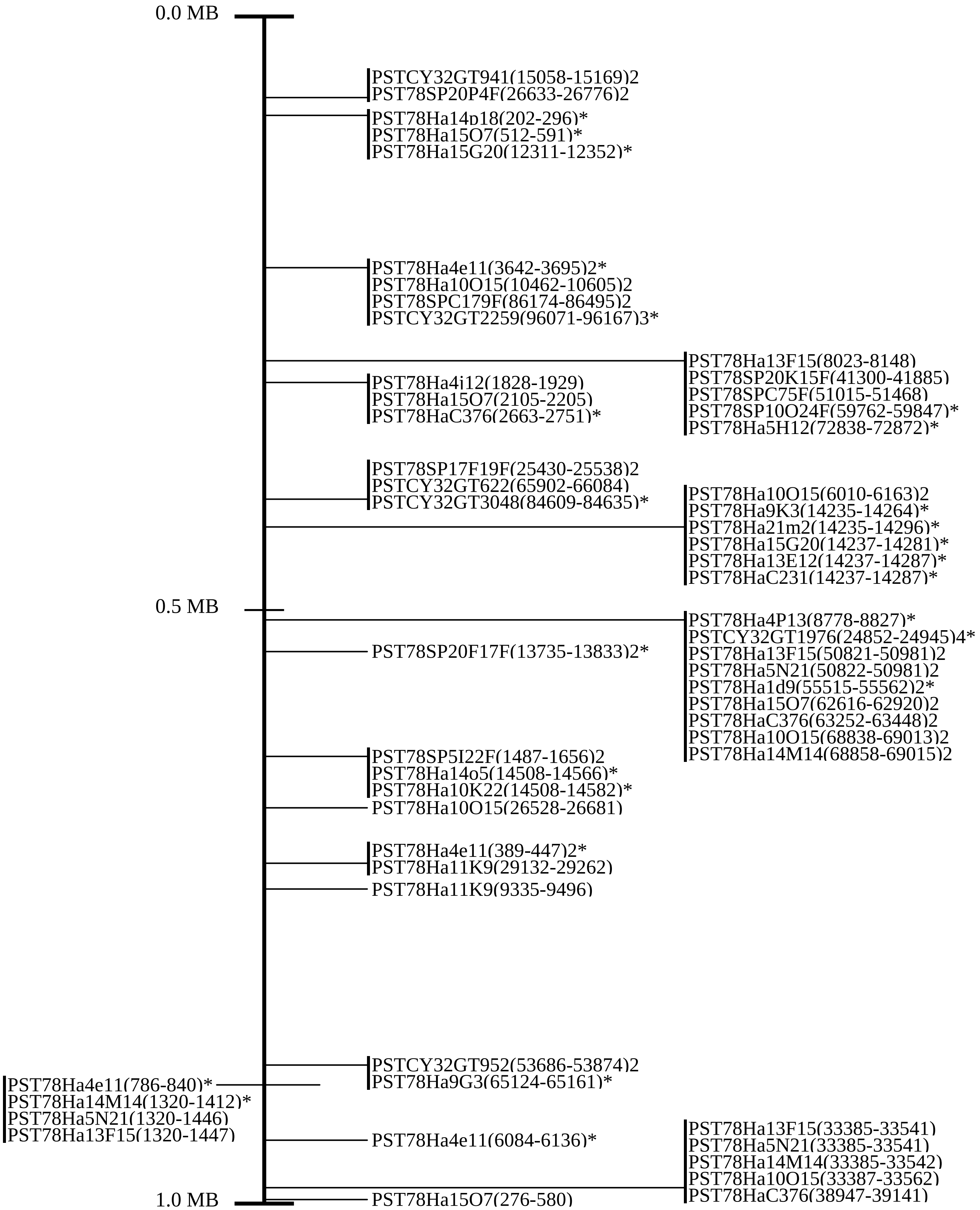

Supercontig 26 (A)
length: 1,006,249 bp

*** = less than 100bp alignment** | **| = clones in the same contig**

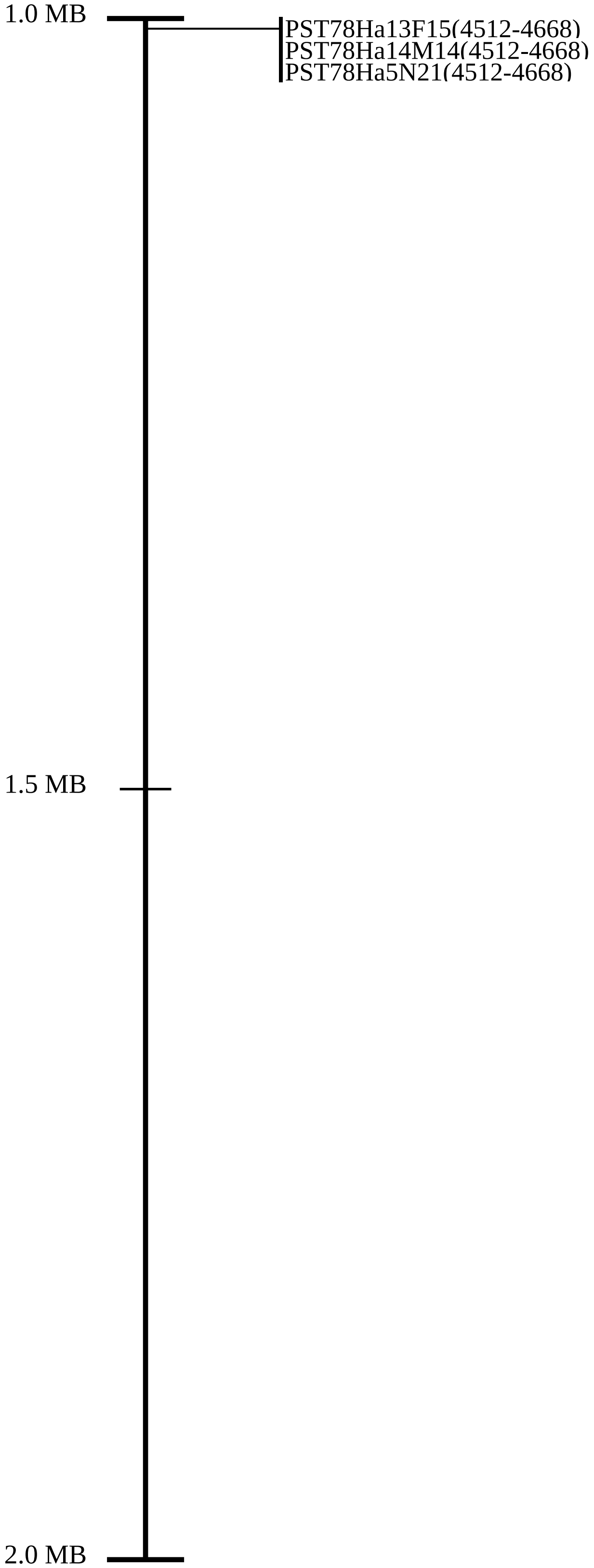

Supercontig 26 (B)
length: 1,006,249 bp

*** = less than 100bp alignment** **| = clones in the same contig**

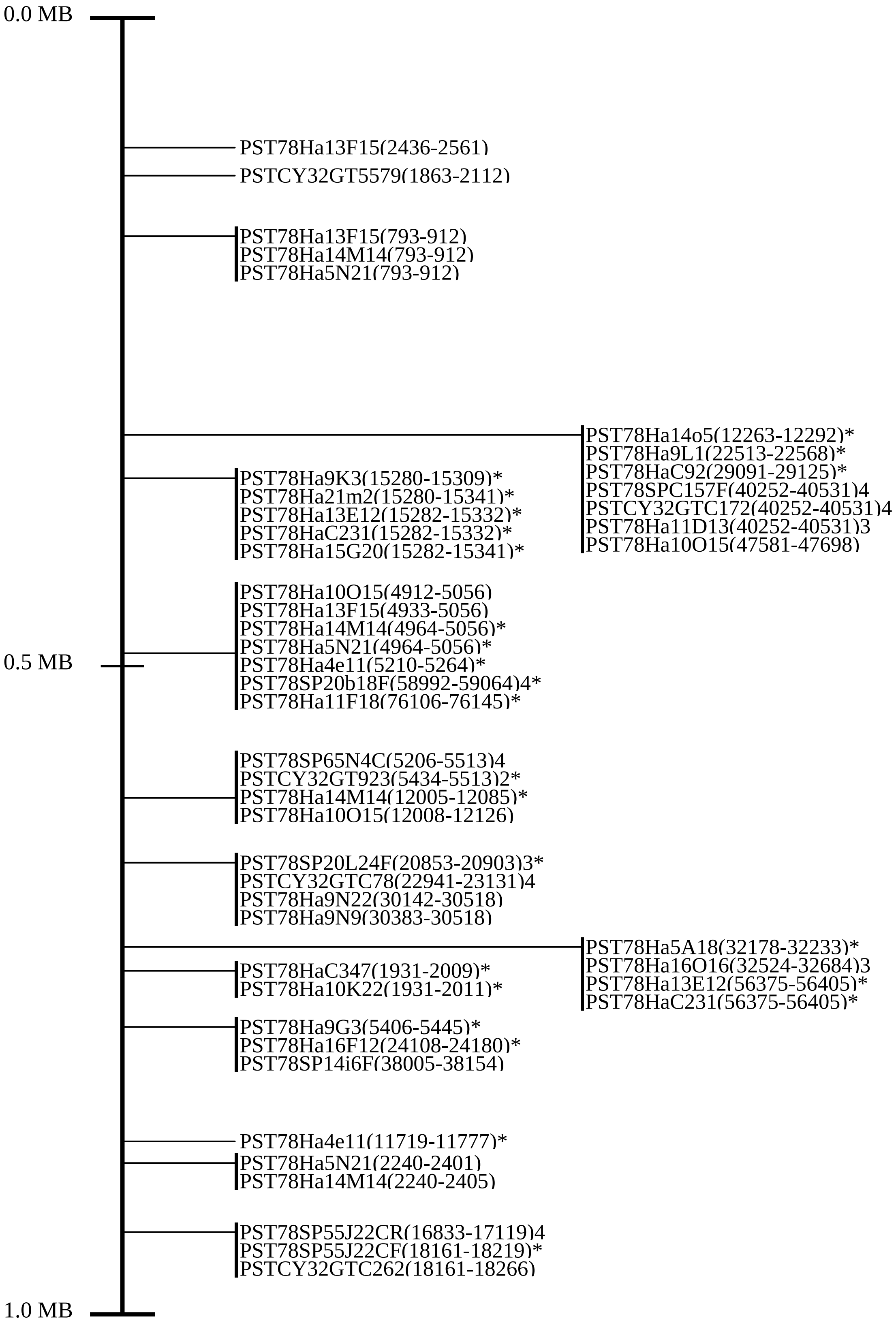

Supercontig 27 (A)
length: 1,005,714 bp

*** = less than 100bp alignment** **| = clones in the same contig**

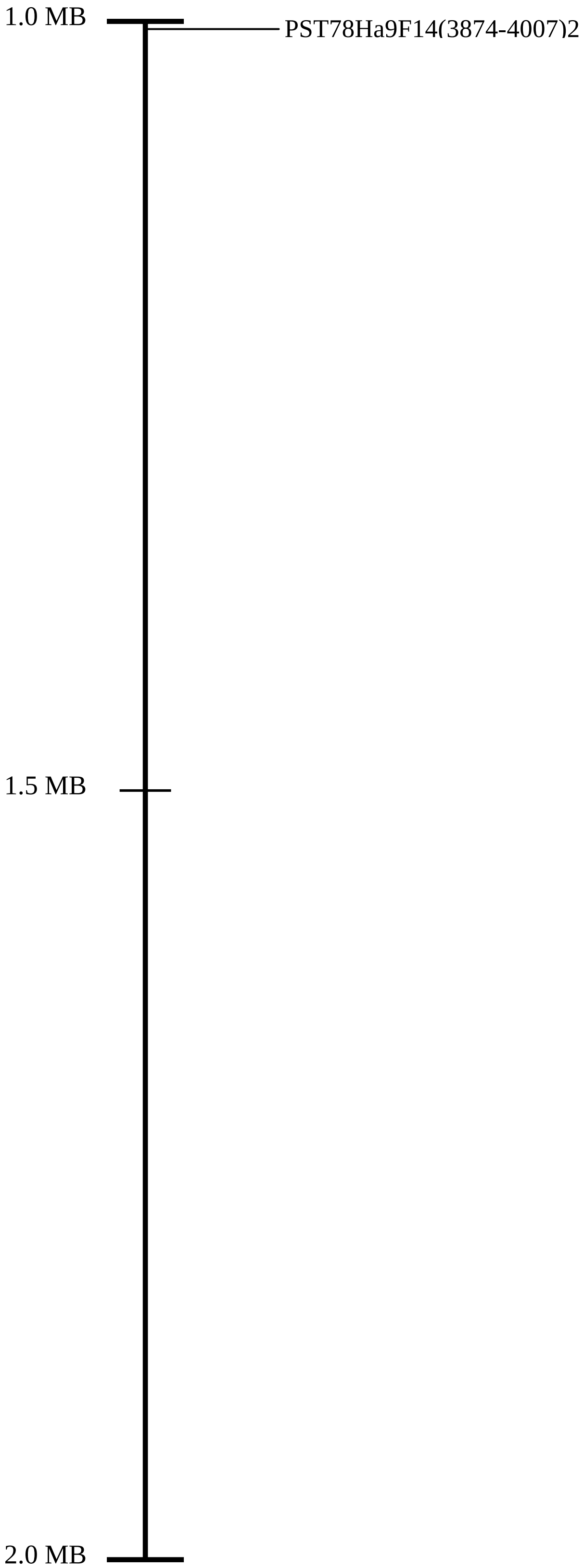

Supercontig 27
length: 1,005,714 bp

*** = less than 100bp alignment** **| = clones in the same contig**

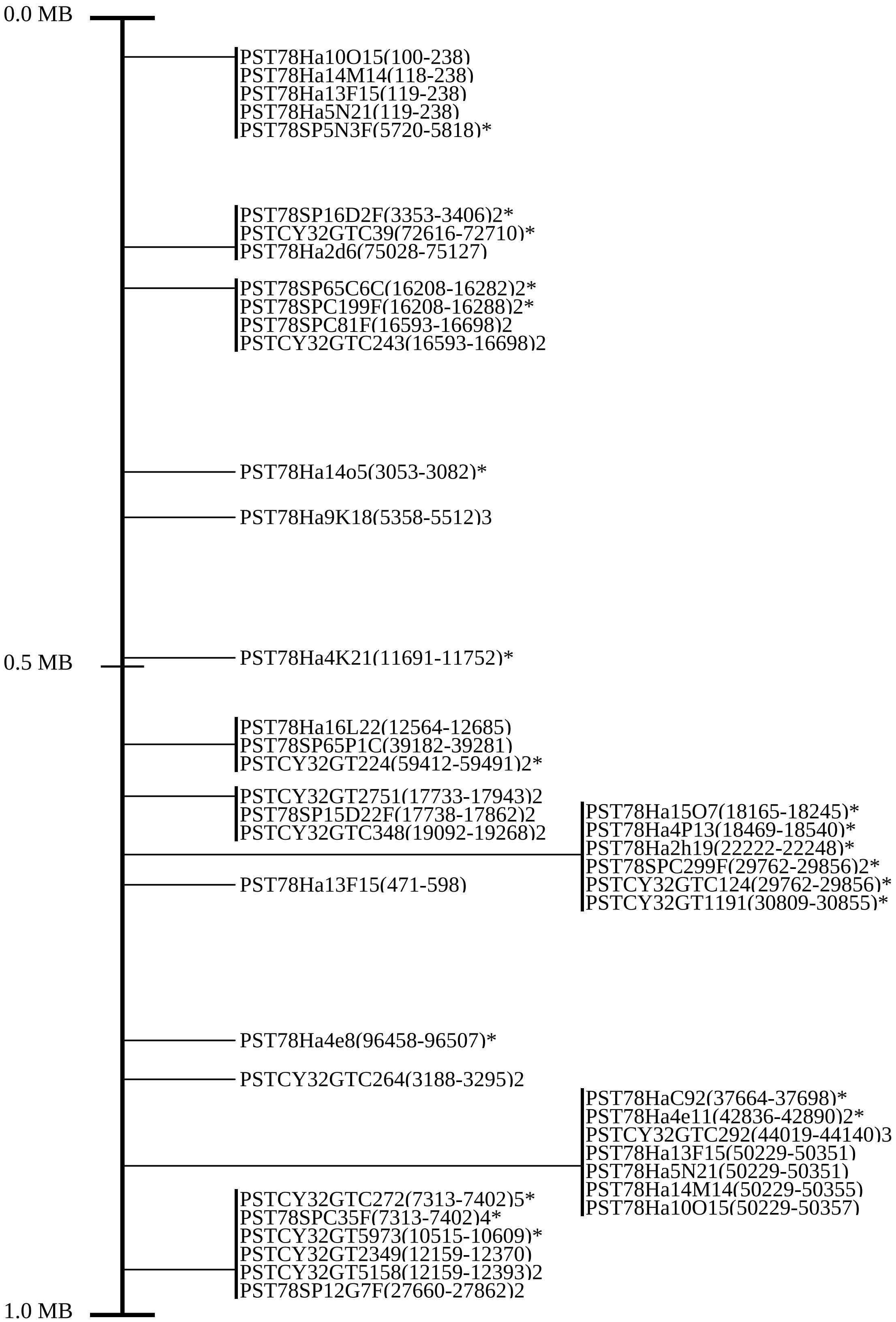

Supercontig 28
length: 964,966 bp

*** = less than 100bp alignment** **| = clones in the same contig**

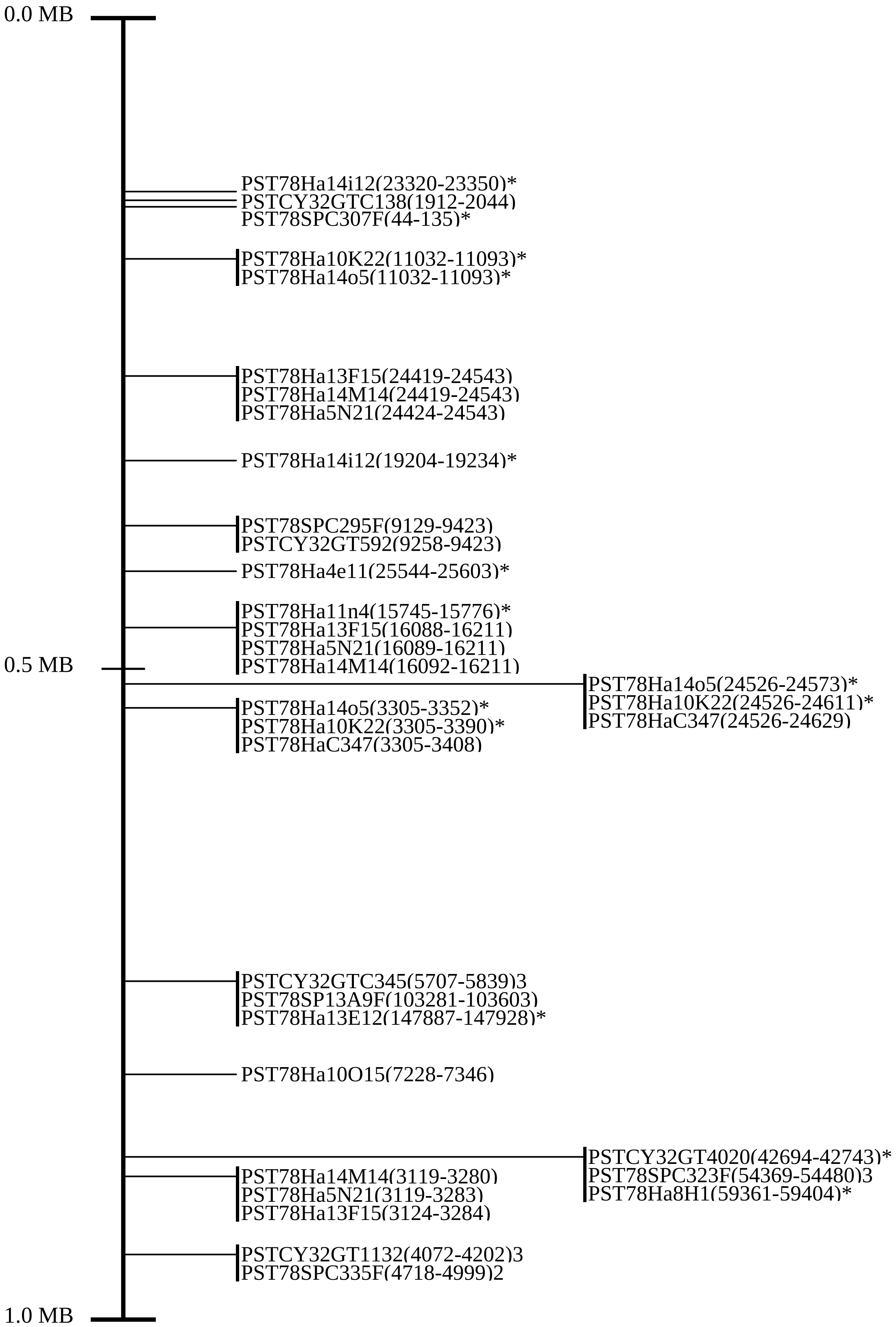

Supercontig 29
length: 999,150 bp

*** = less than 100bp alignment**

| = clones in the same contig

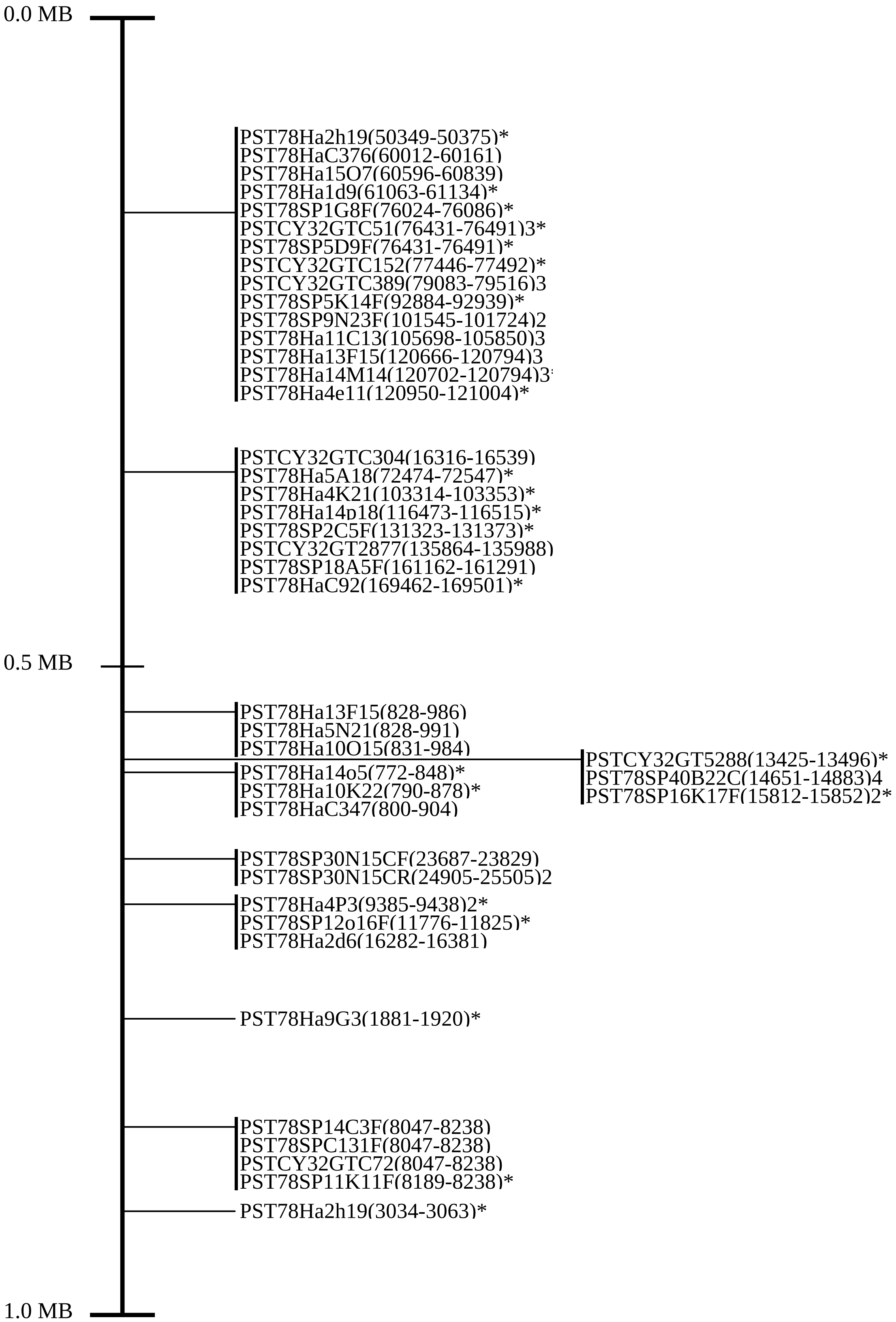

Supercontig 30
length: 919,905 bp

*** = less than 100bp alignment** **| = clones in the same contig**

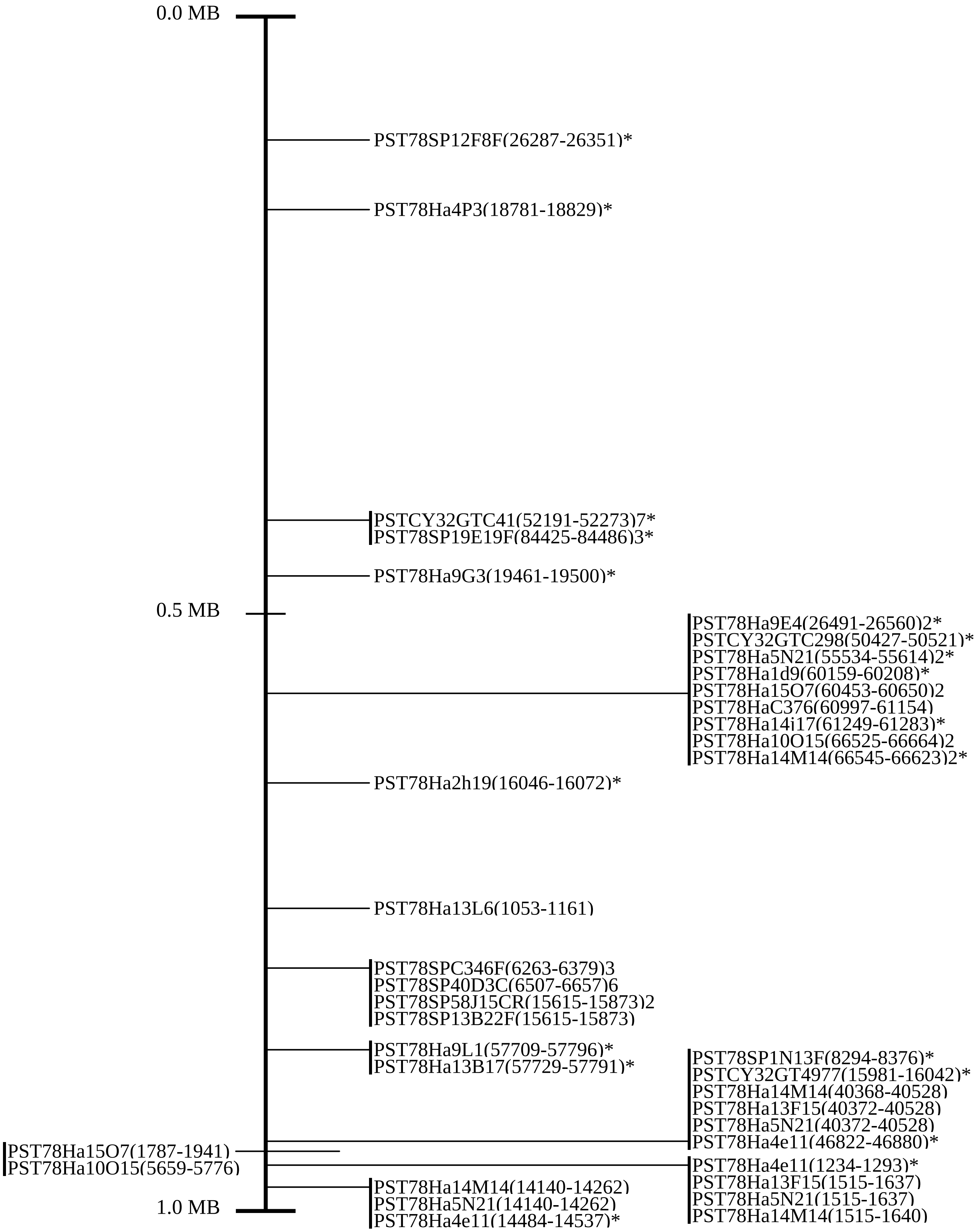

Supercontig 31
length: 986,084 bp

*** = less than 100bp alignment** **| = clones in the same contig**

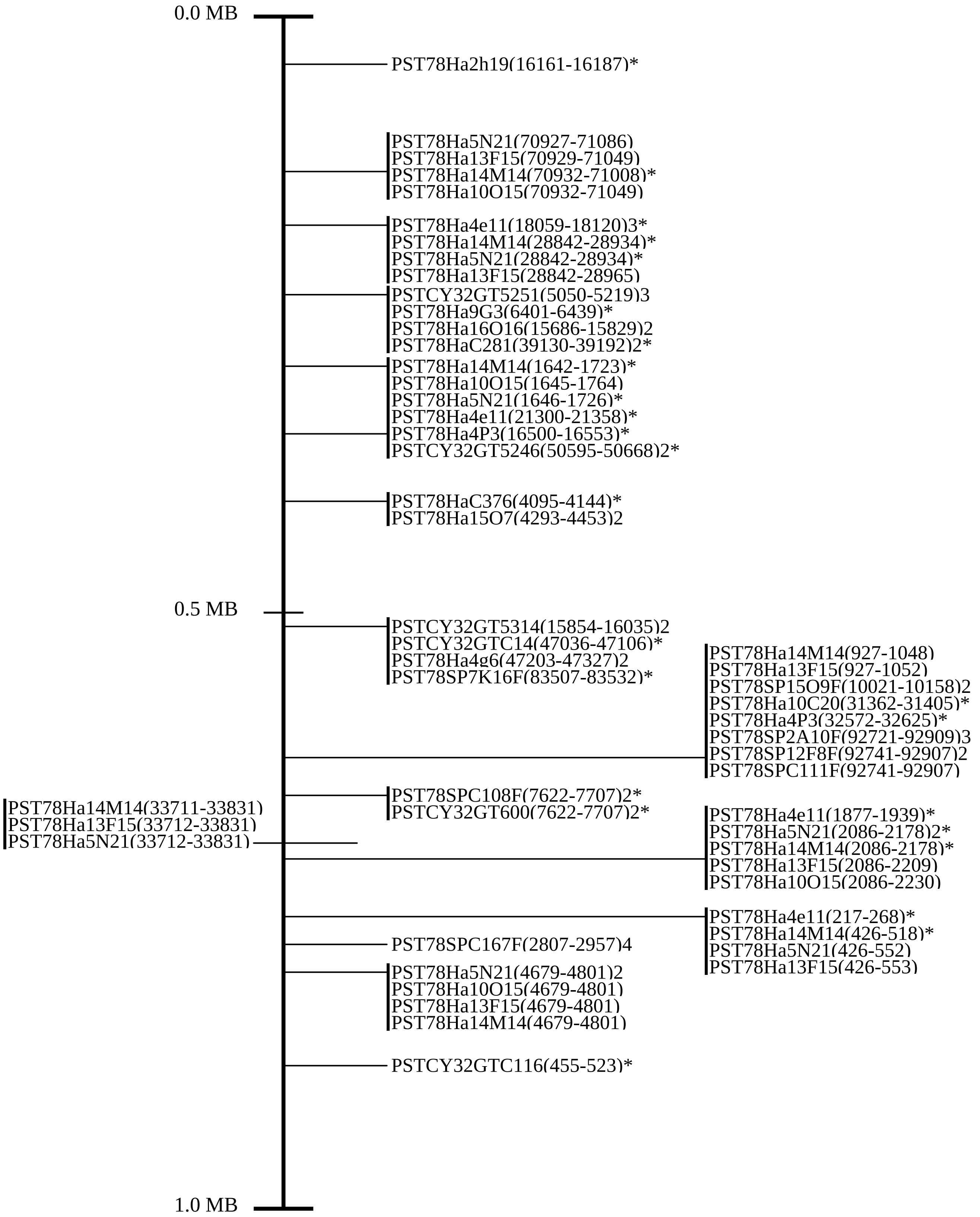

Supercontig 32
length: 894,979 bp

*** = less than 100bp alignment** **| = clones in the same contig**

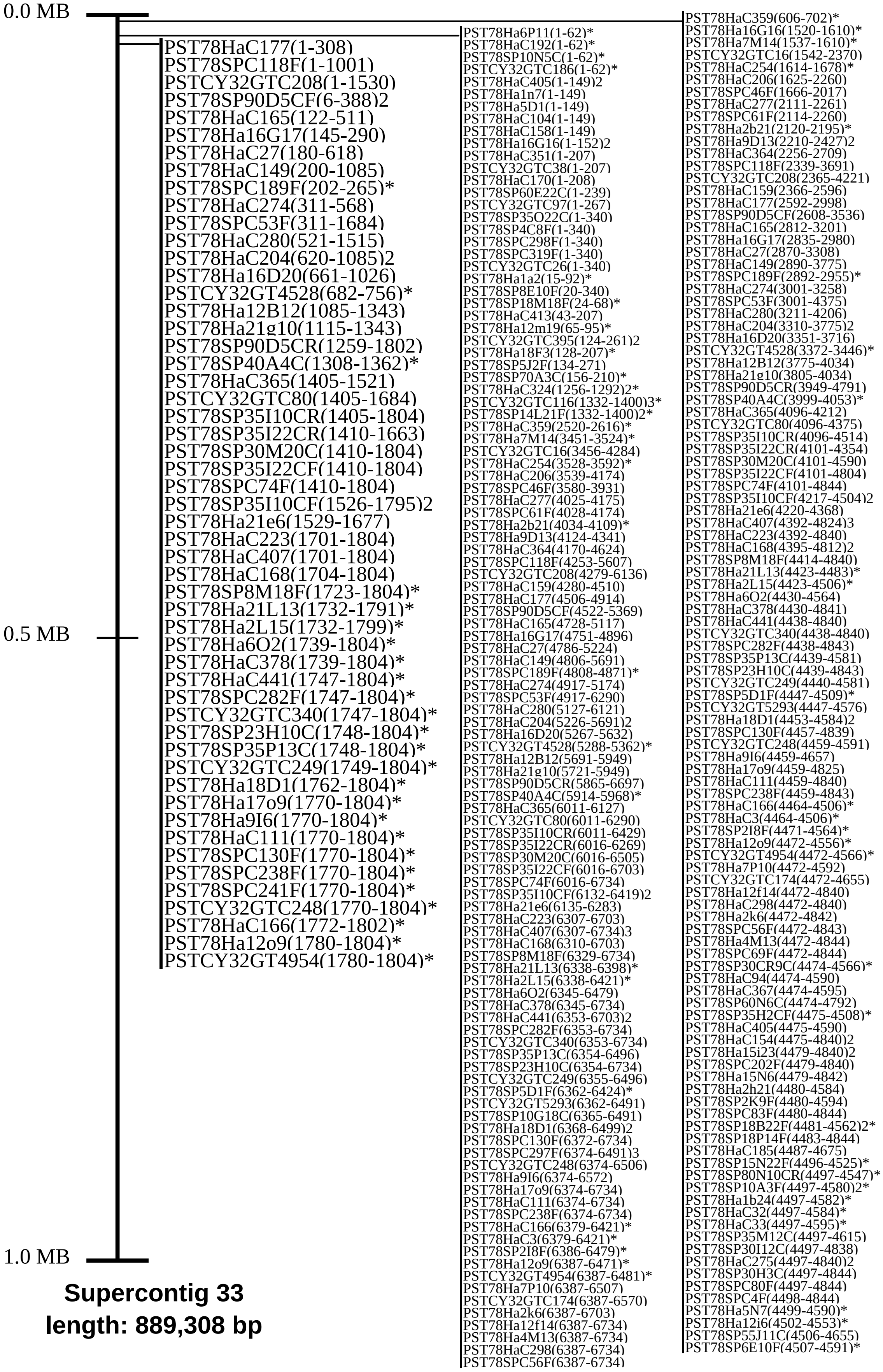

Supercontig 33
length: 889,308 bp

*** = less than 100bp alignment**

| = clones in the same contig

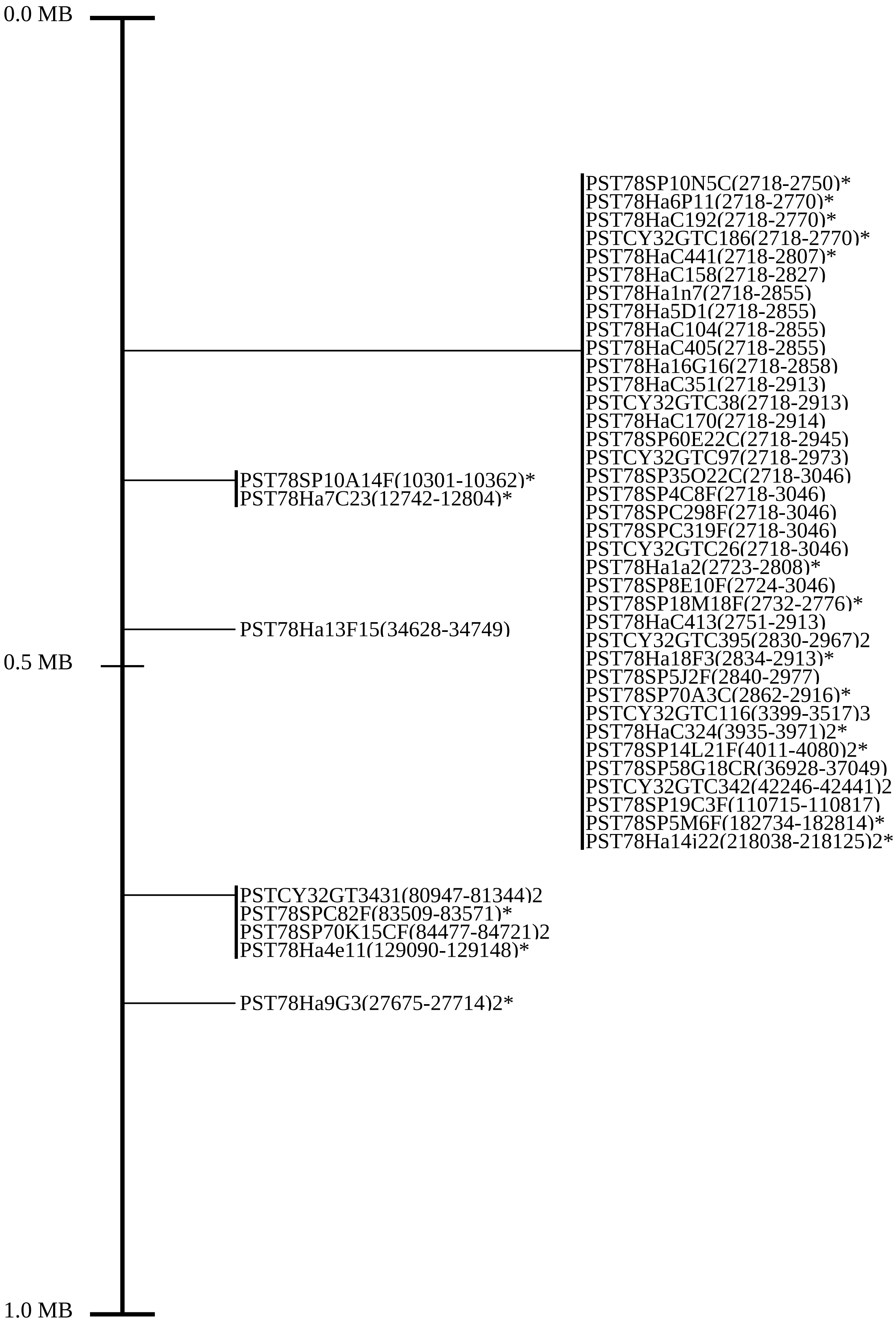

Supercontig 33
length: 889,308 bp

*** = less than 100bp alignment** **| = clones in the same contig**

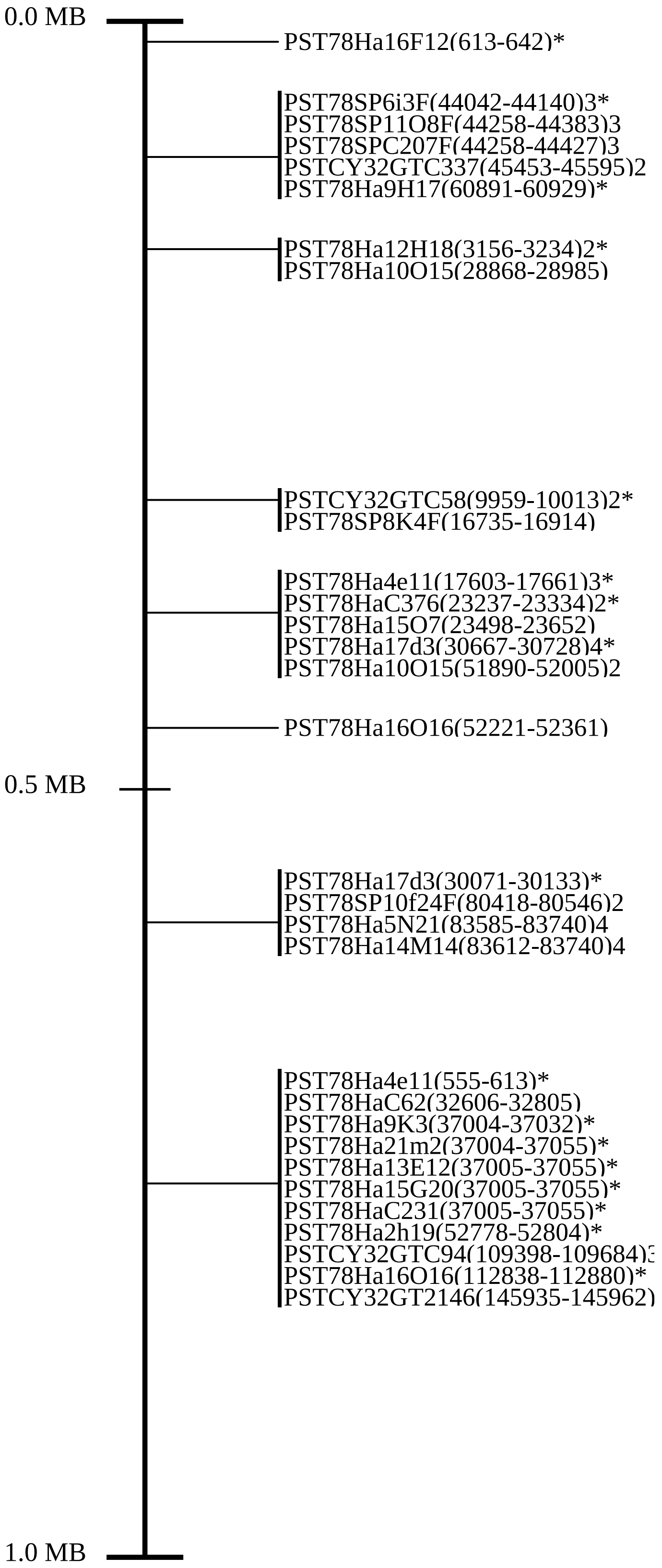

Supercontig 34
length: 856,319 bp

*** = less than 100bp alignment** **| = clones in the same contig**

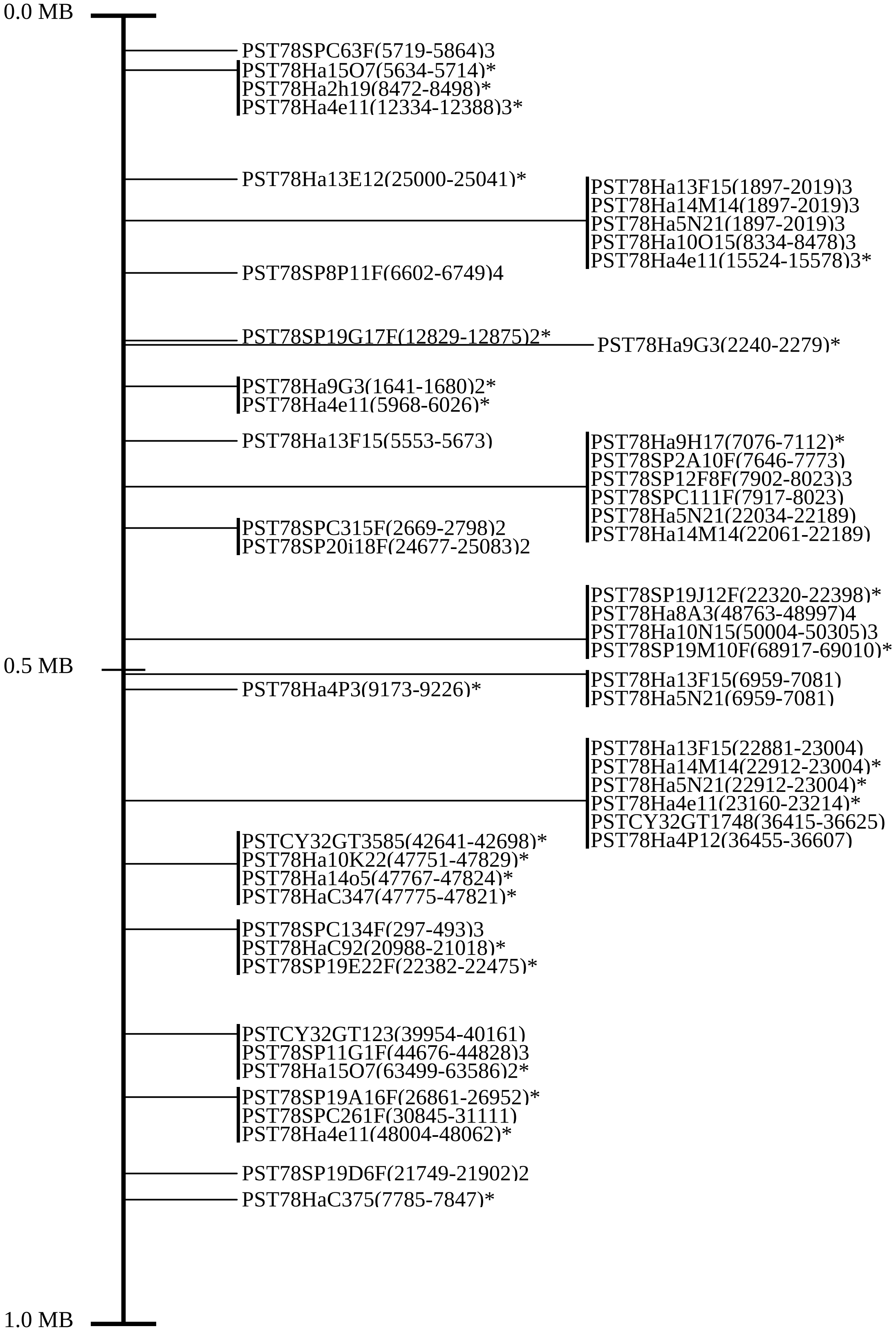

Supercontig 35
length: 904,227 bp

*** = less than 100bp alignment** **| = clones in the same contig**

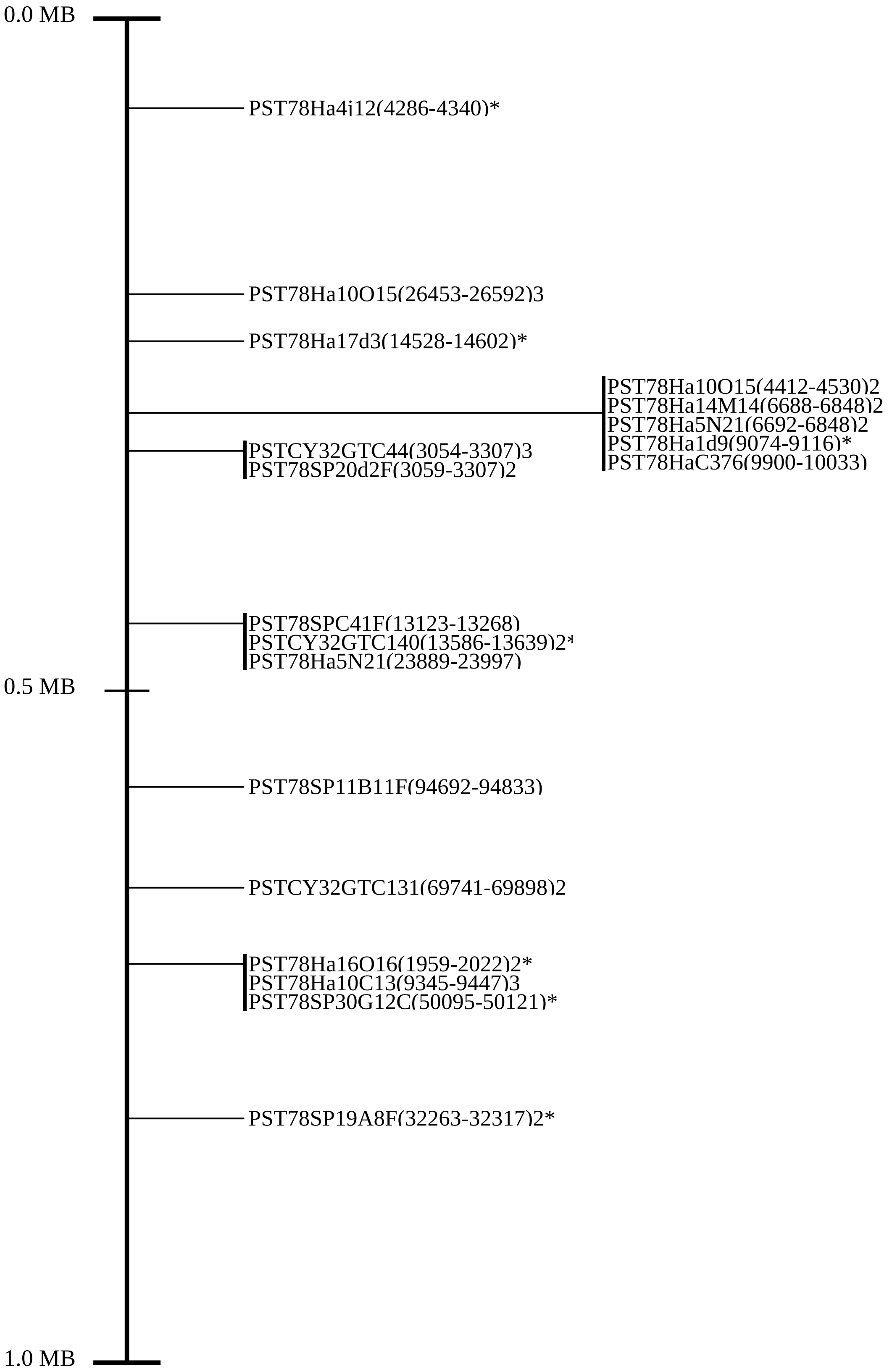

Supercontig 36
length: 867,522 bp

*** = less than 100bp alignment**

| = clones in the same contig

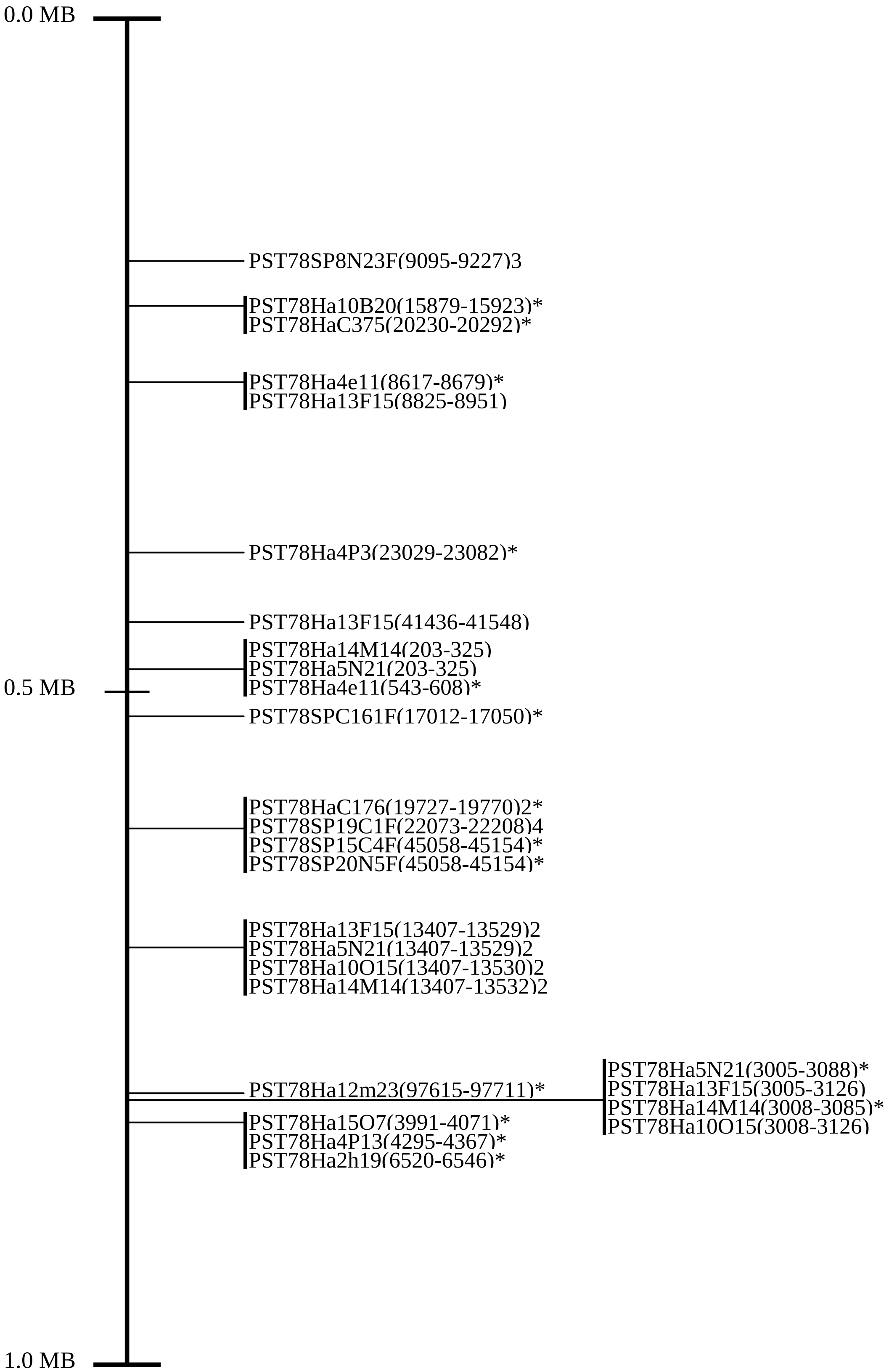

Supercontig 37
length: 820,150 bp

*** = less than 100bp alignment** **| = clones in the same contig**

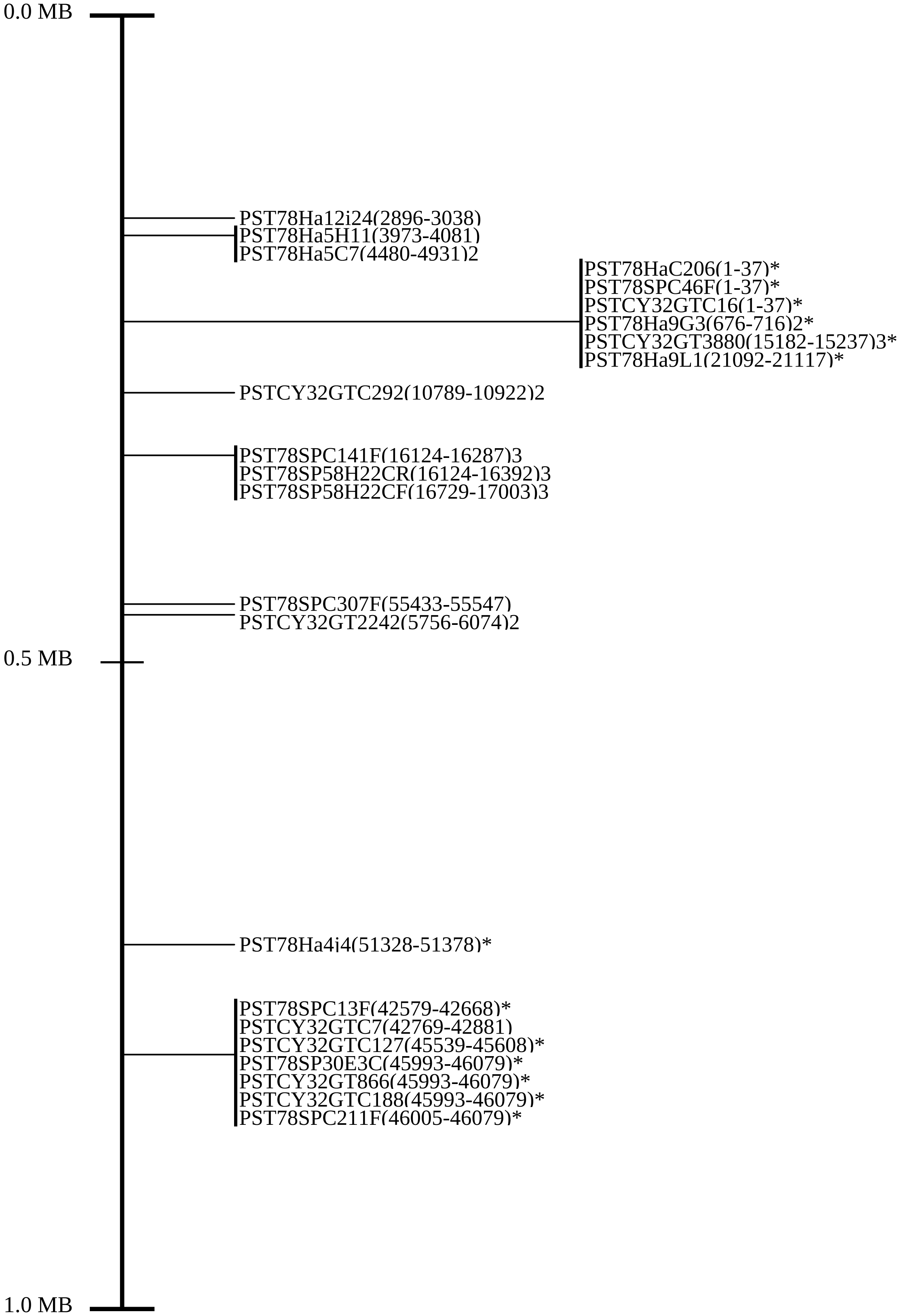

Supercontig 38
length: 803,102 bp

*** = less than 100bp alignment** **| = clones in the same contig**

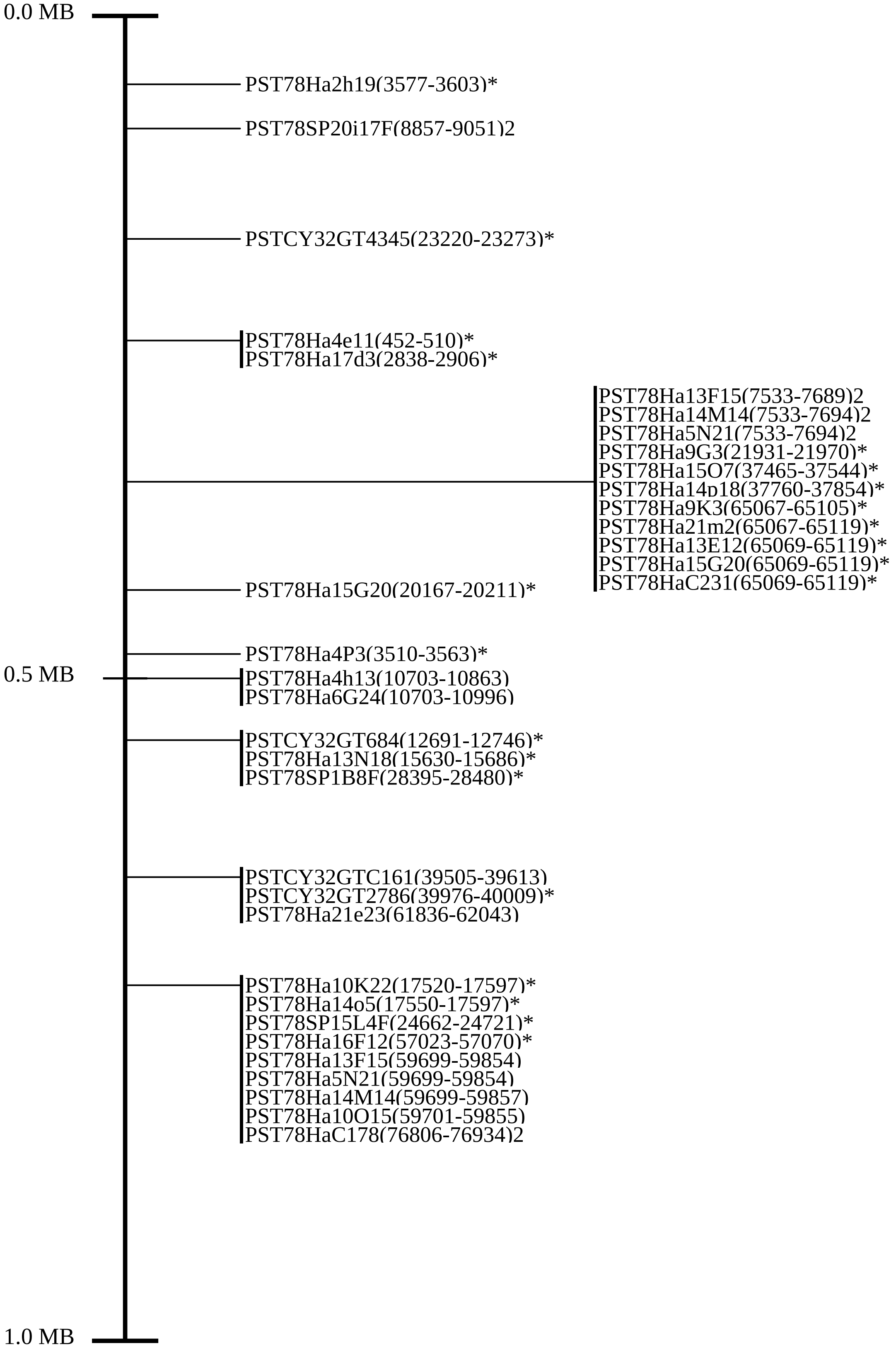

Supercontig 39
length: 752,863 bp

*** = less than 100bp alignment** **| = clones in the same contig**

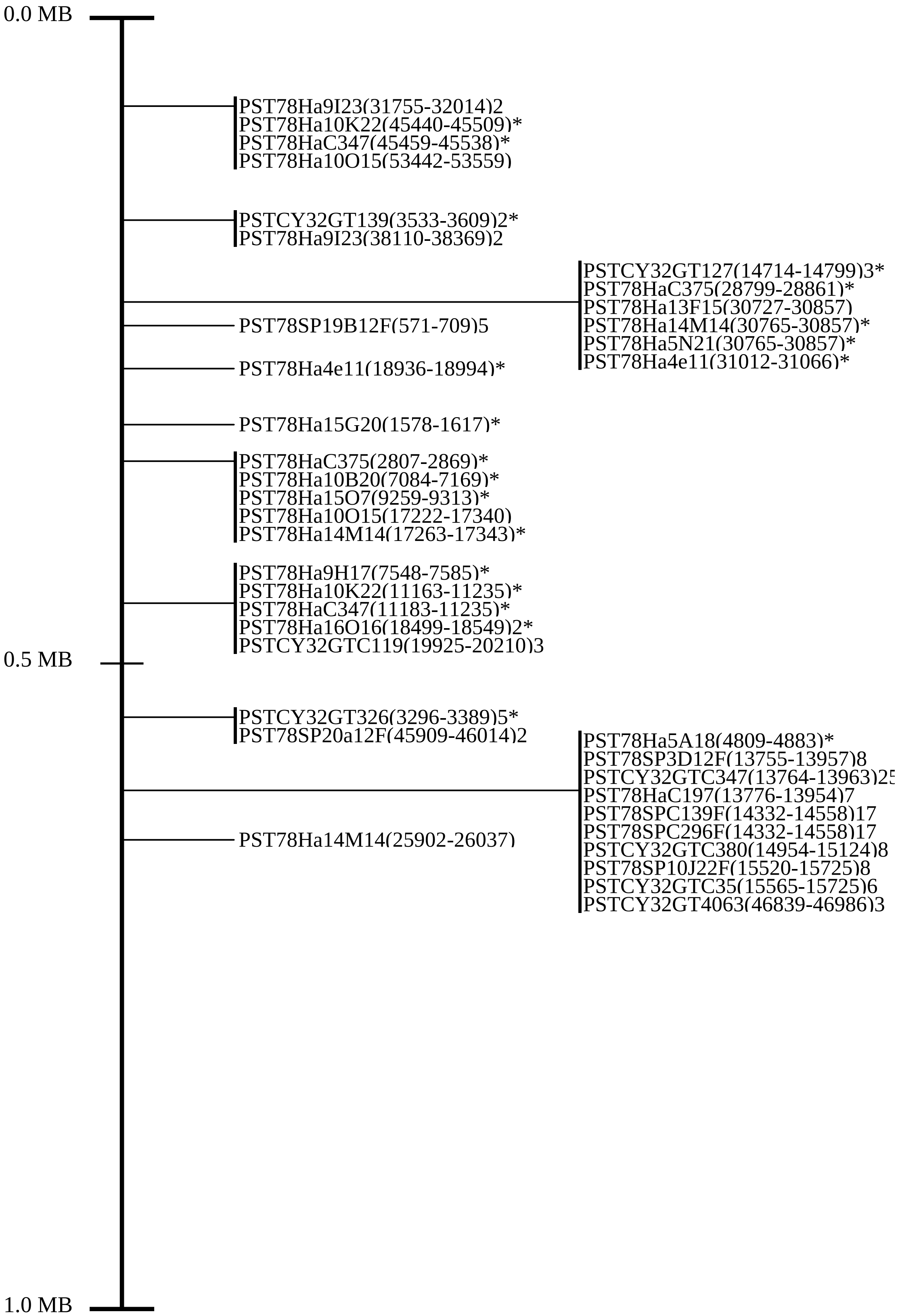

Supercontig 40
length: 667,254 bp

*** = less than 100bp alignment** **| = clones in the same contig**

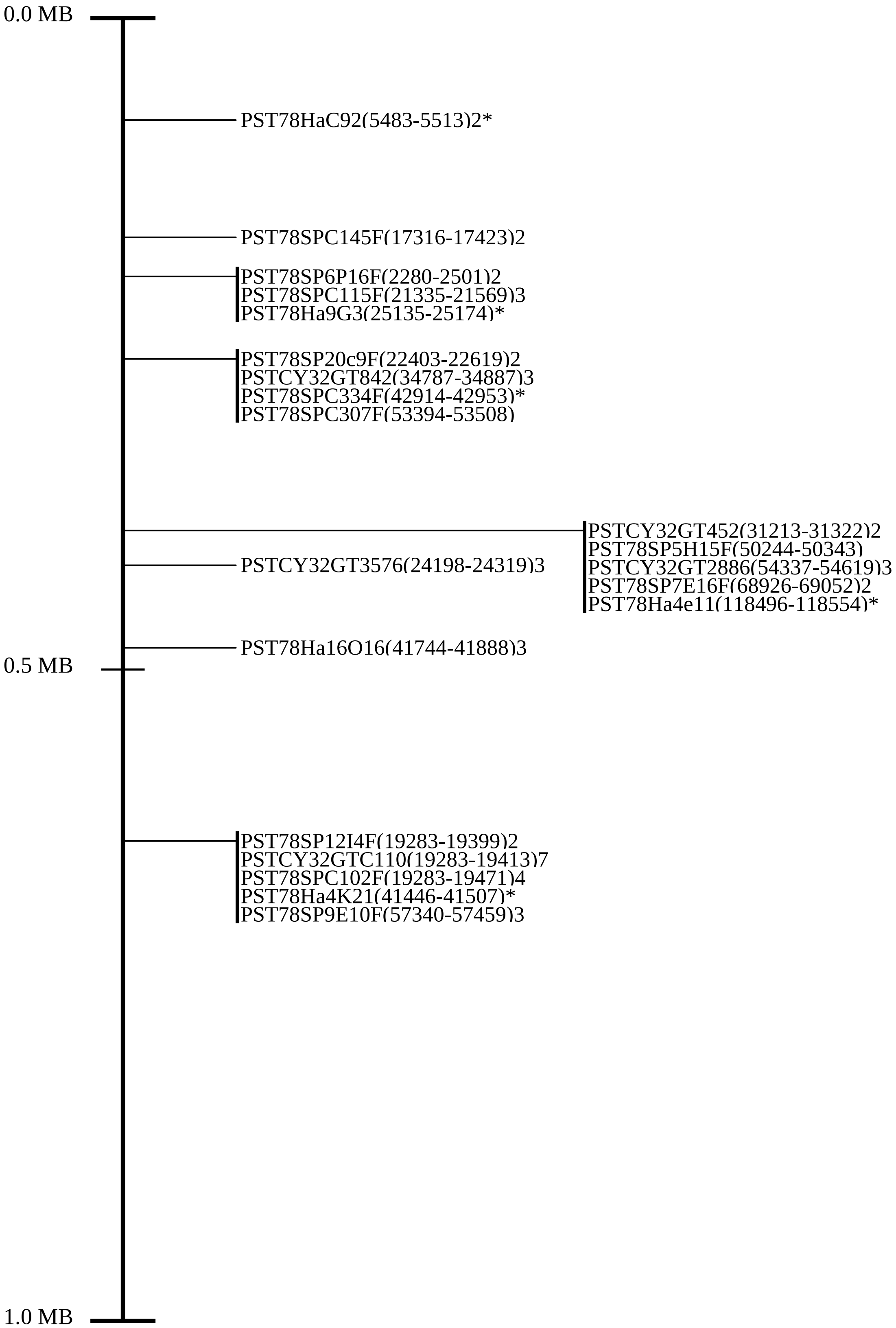

Supercontig 41
length: 658,202 bp

*** = less than 100bp alignment** **| = clones in the same contig**

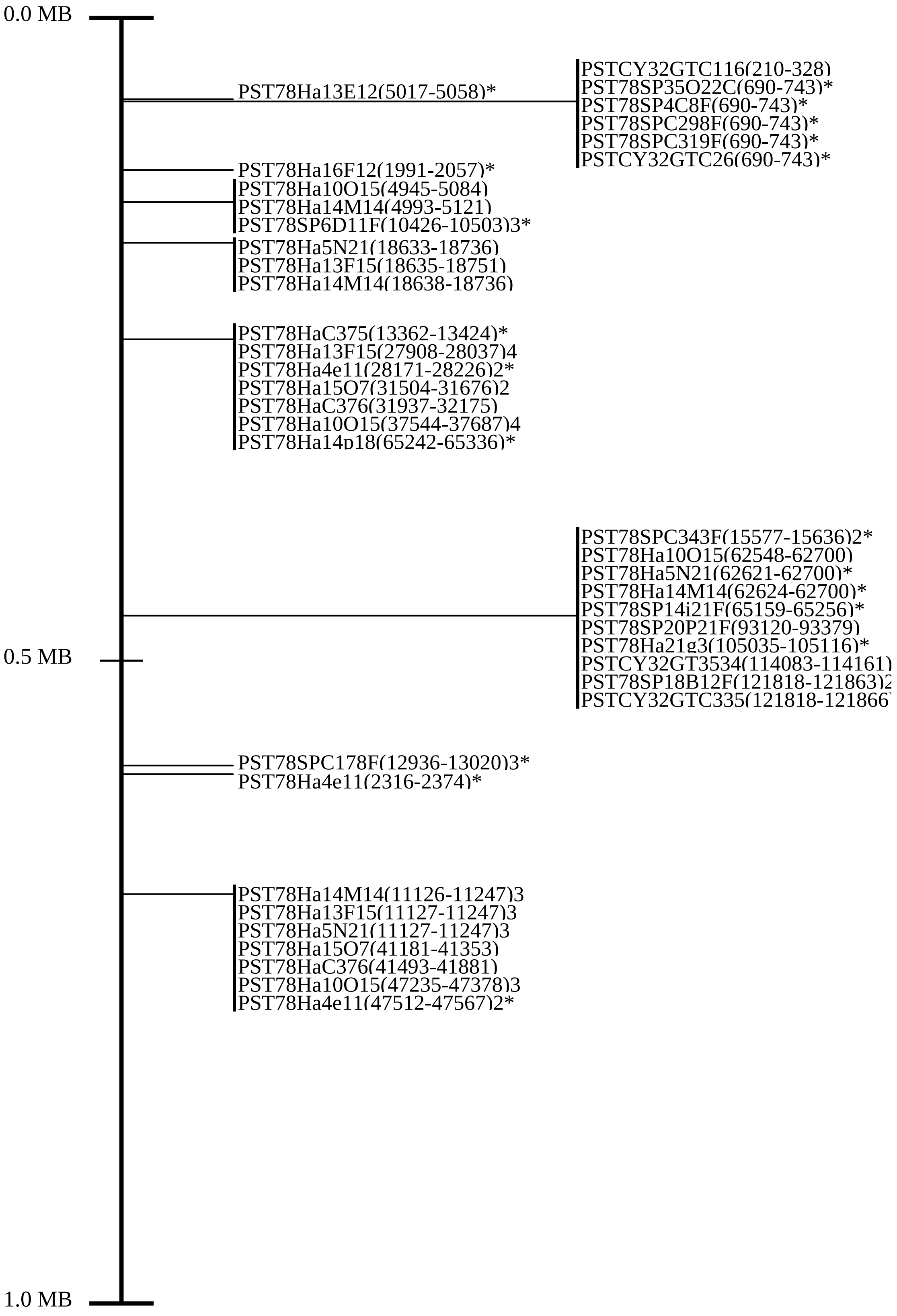

Supercontig 42
length: 682,257 bp

*** = less than 100bp alignment** **| = clones in the same contig**

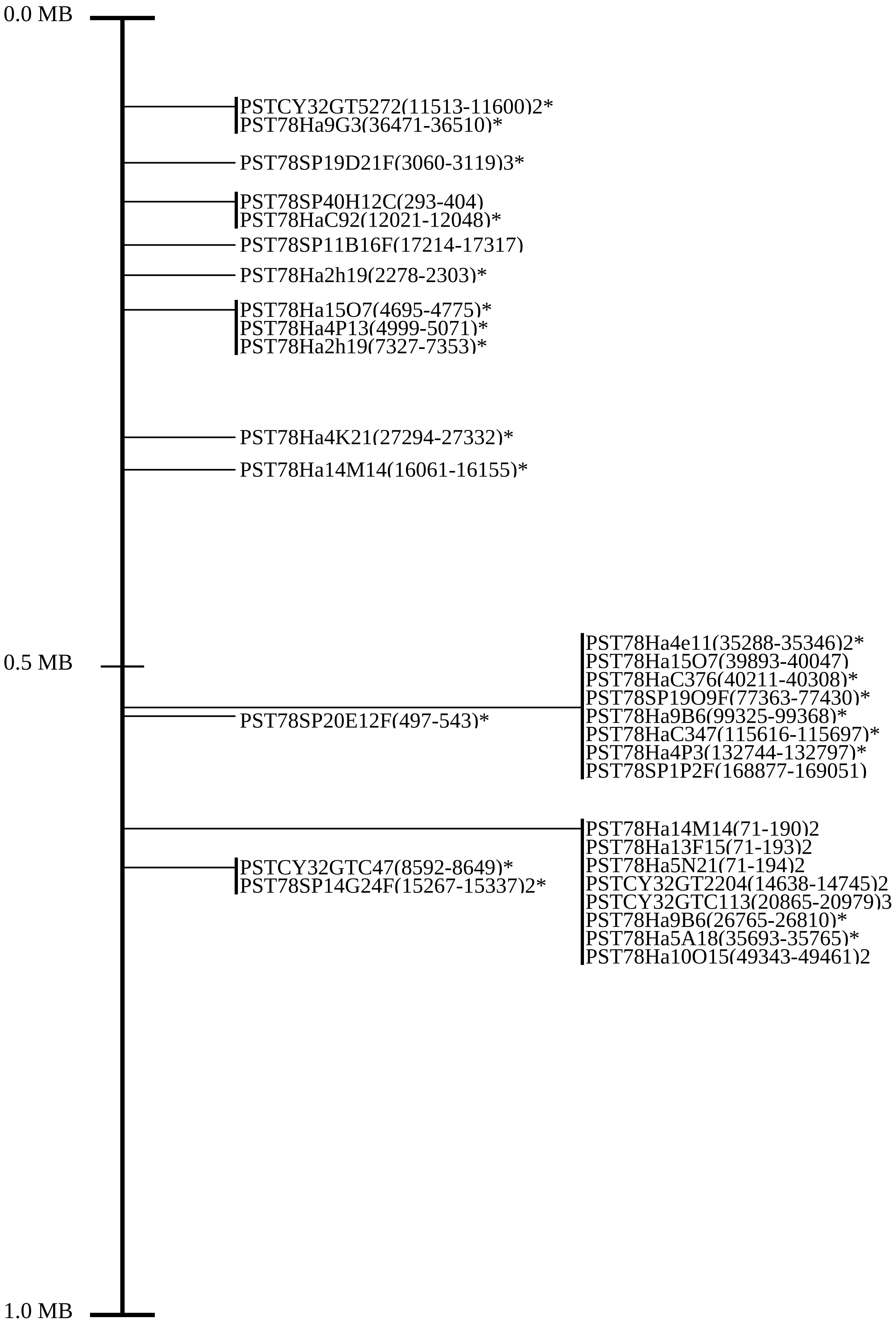

Supercontig 43
length: 654,820 bp

*** = less than 100bp alignment** | **| = clones in the same contig**

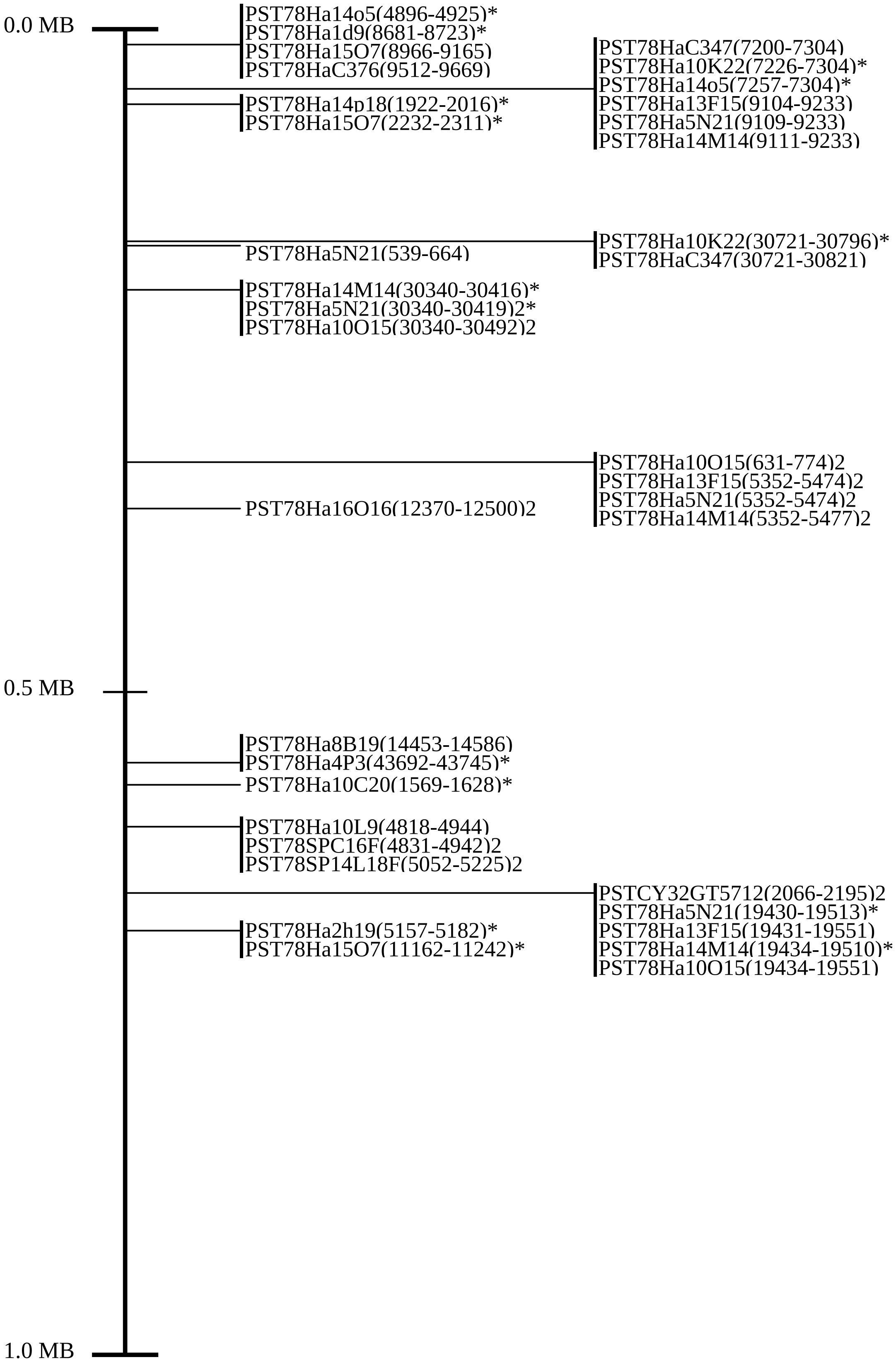

Supercontig 44
length: 680,700 bp

*** = less than 100bp alignment** **| = clones in the same contig**

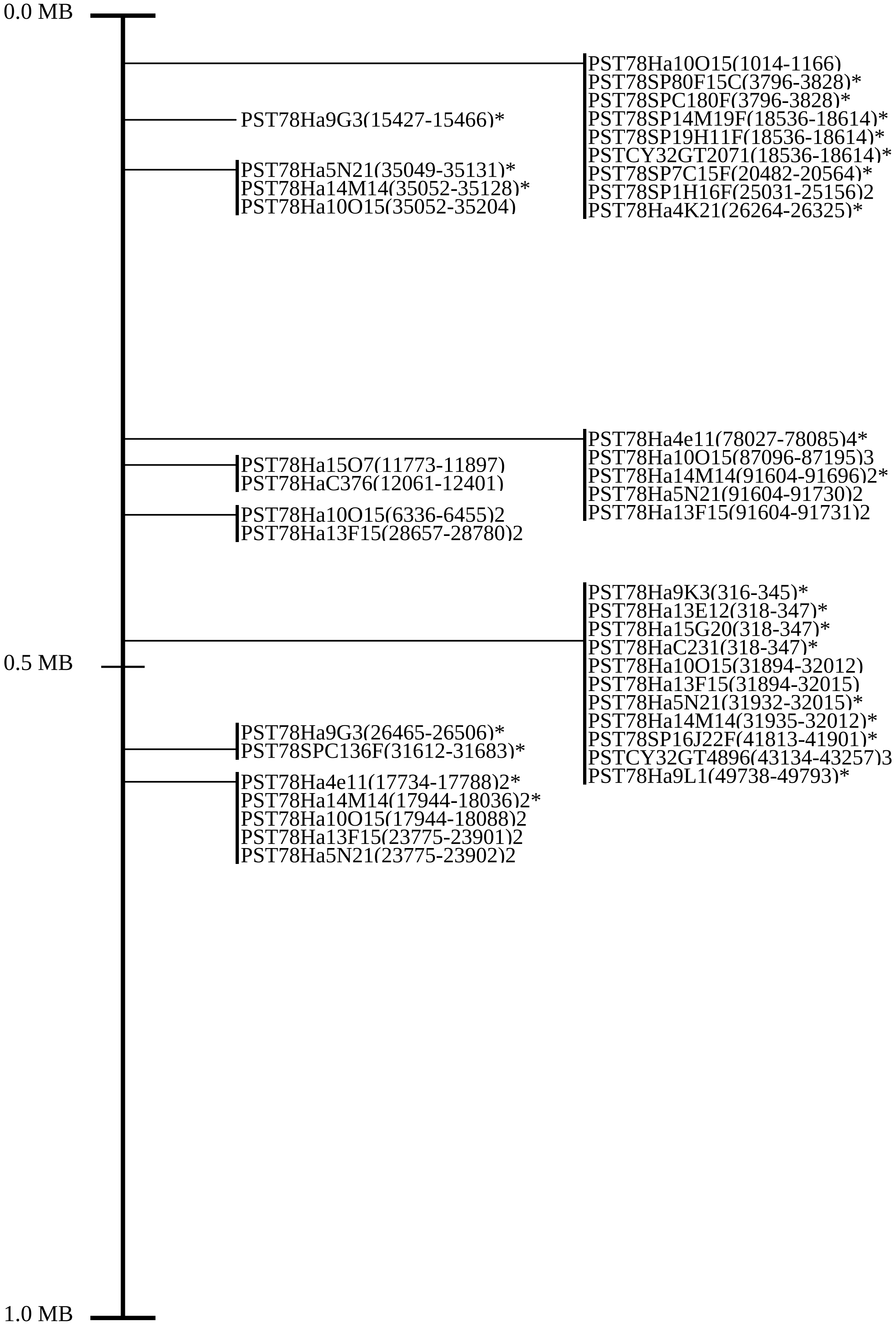

Supercontig 45
length: 611,533 bp

*** = less than 100bp alignment** **| = clones in the same contig**

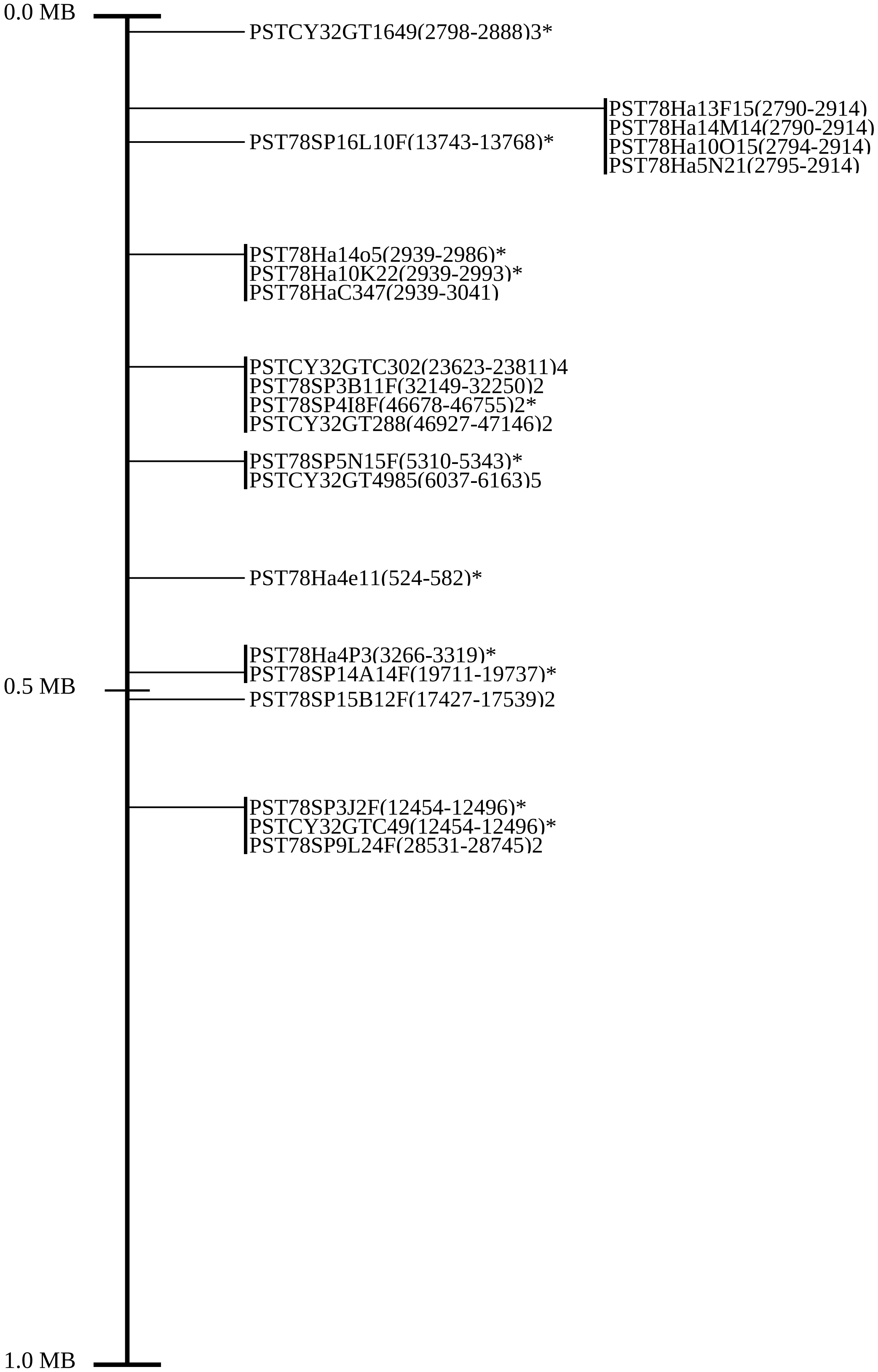

Supercontig 46
length: 603,048 bp

*** = less than 100bp alignment** **| = clones in the same contig**

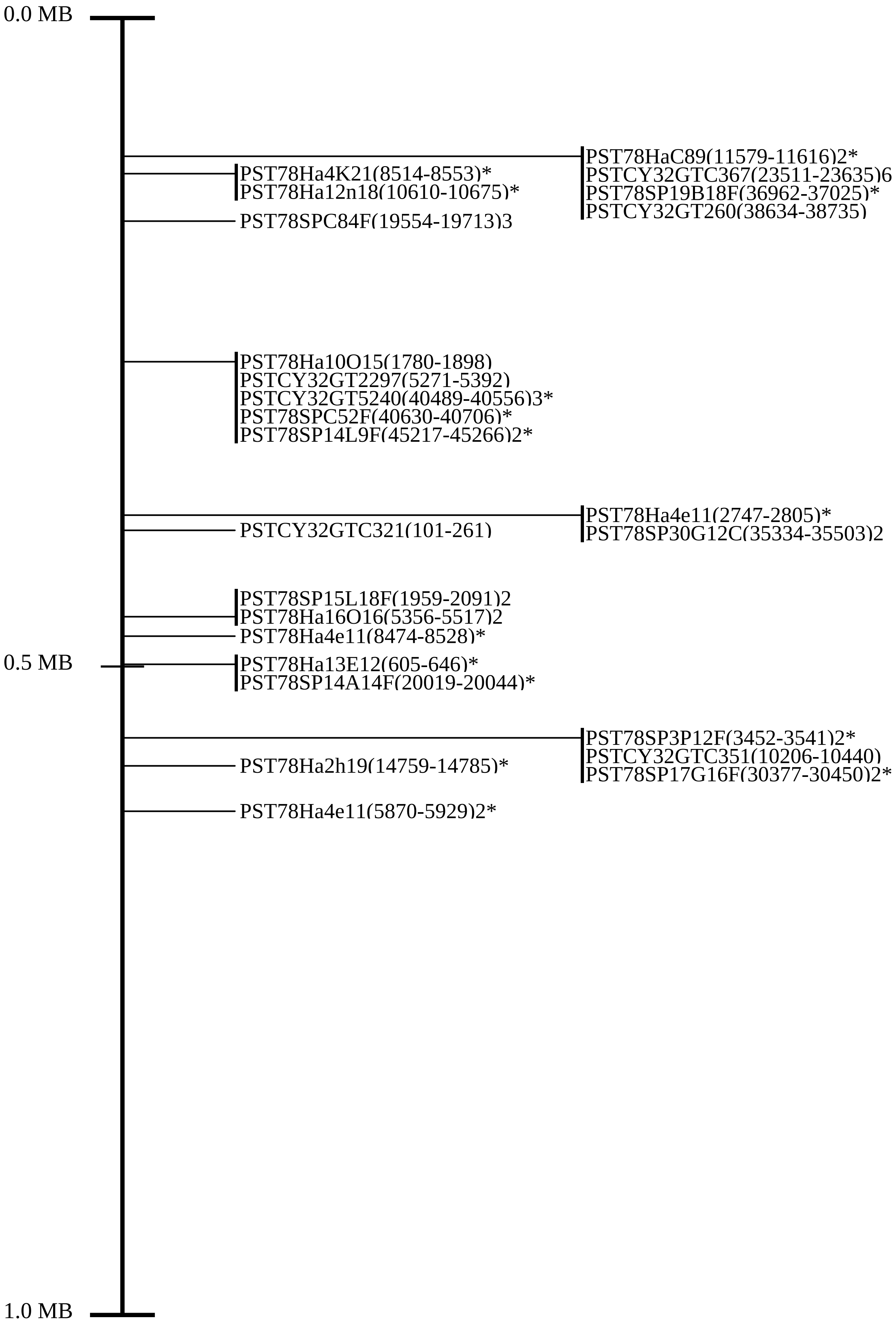

Supercontig 47
length: 640,690 bp

*** = less than 100bp alignment** **| = clones in the same contig**

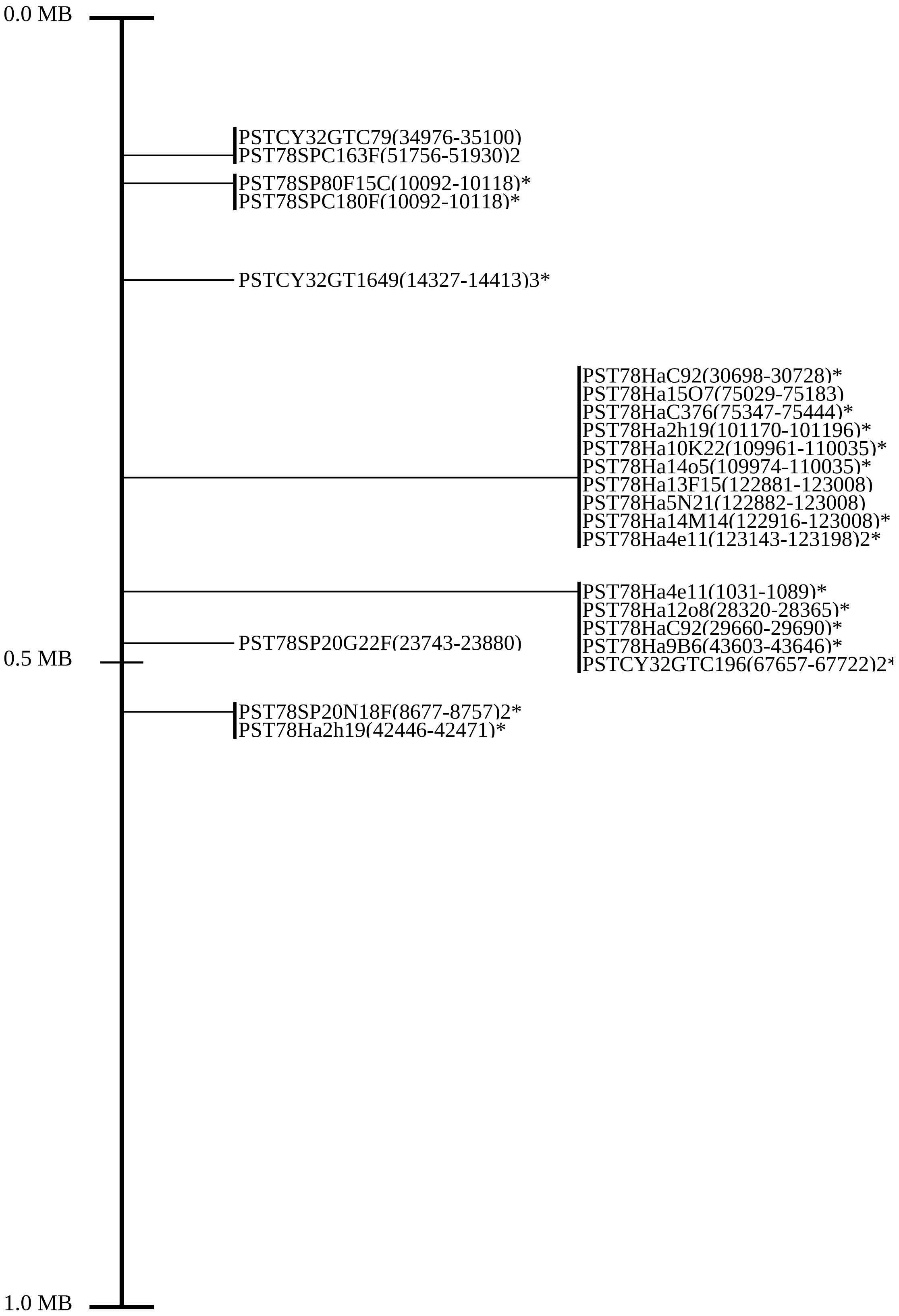

Supercontig 48
length: 569,981 bp

*** = less than 100bp alignment** **| = clones in the same contig**

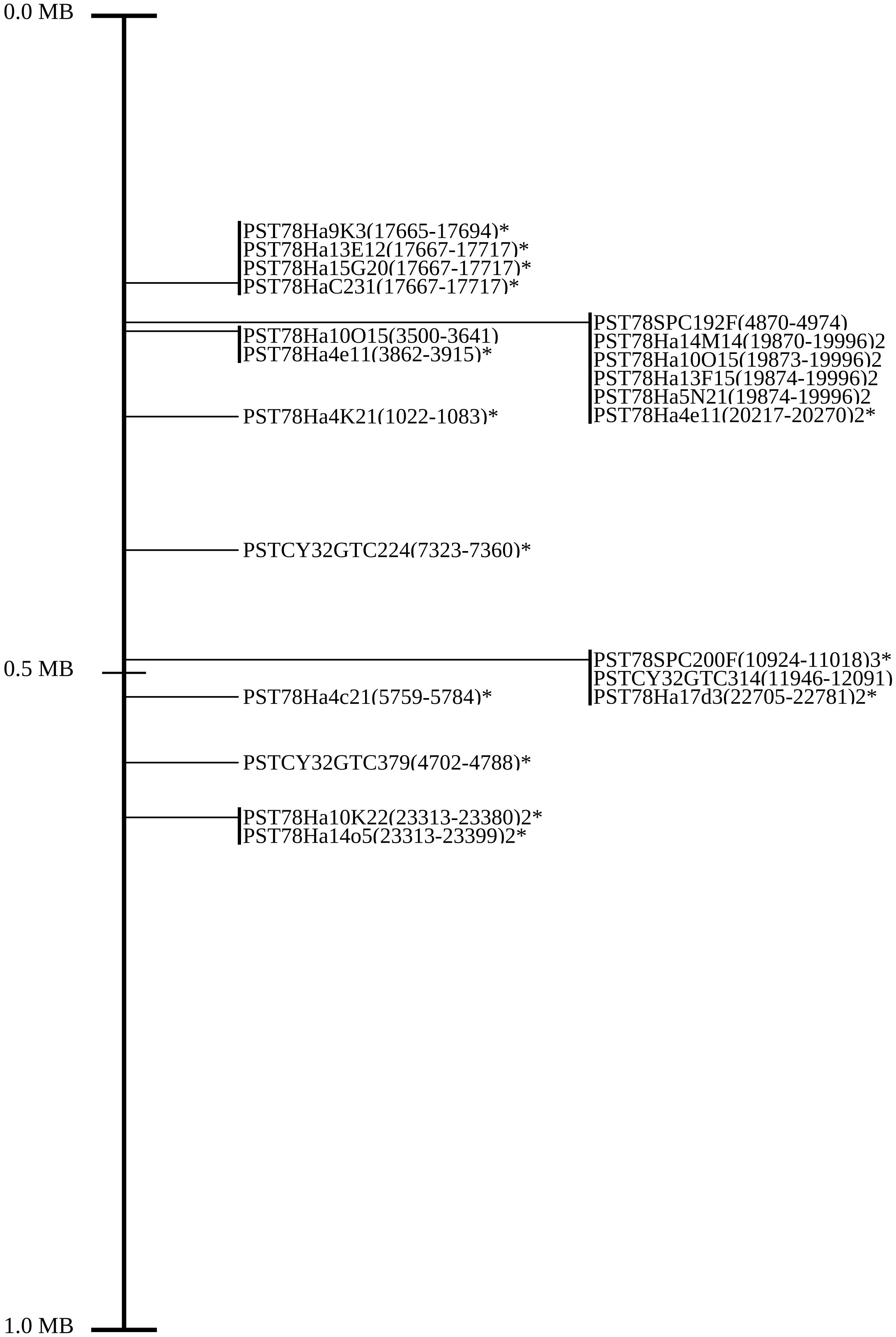

Supercontig 49
length: 609,911 bp

*** = less than 100bp alignment** **| = clones in the same contig**

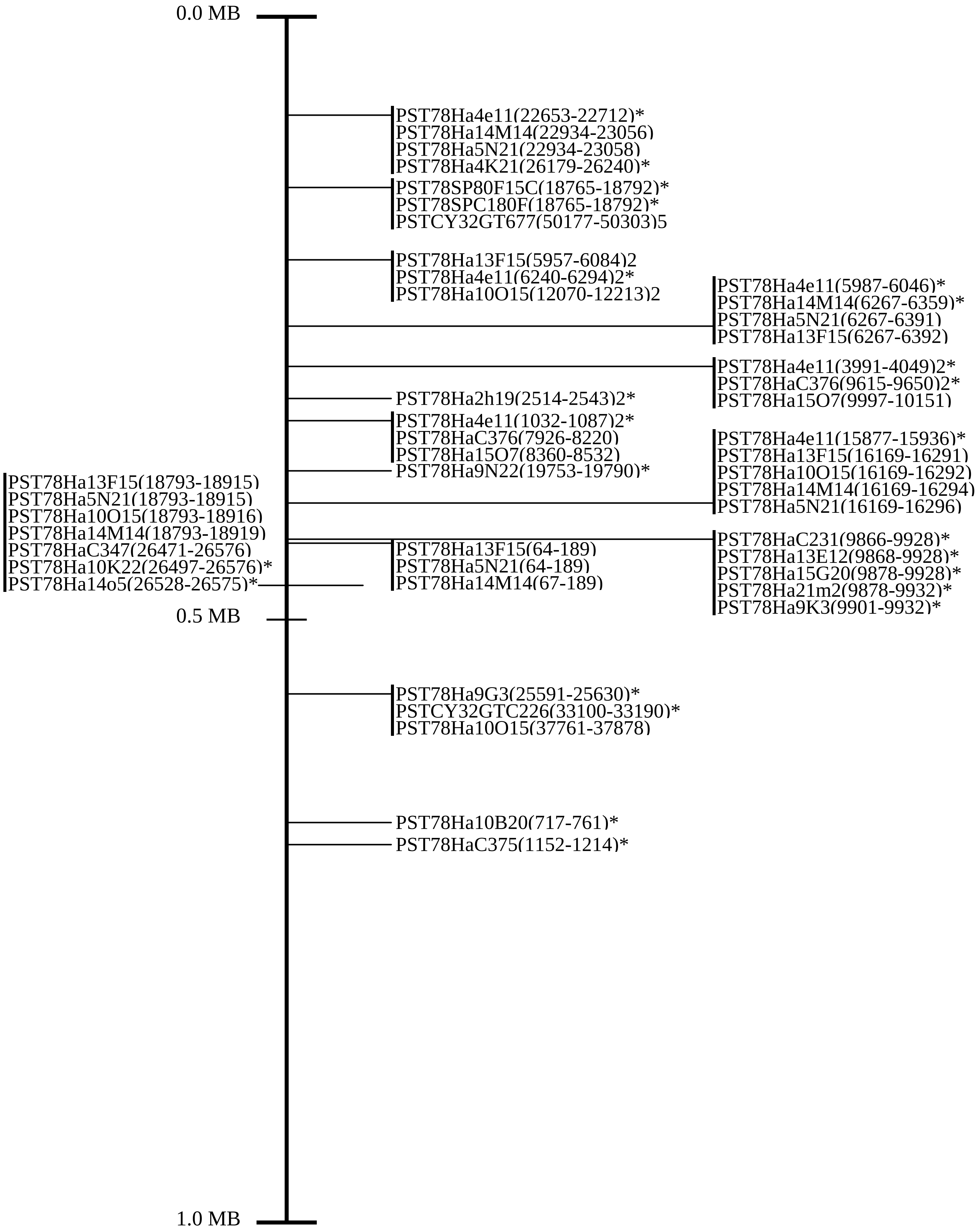

Supercontig 50
length: 685,923 bp

*** = less than 100bp alignment** **| = clones in the same contig**

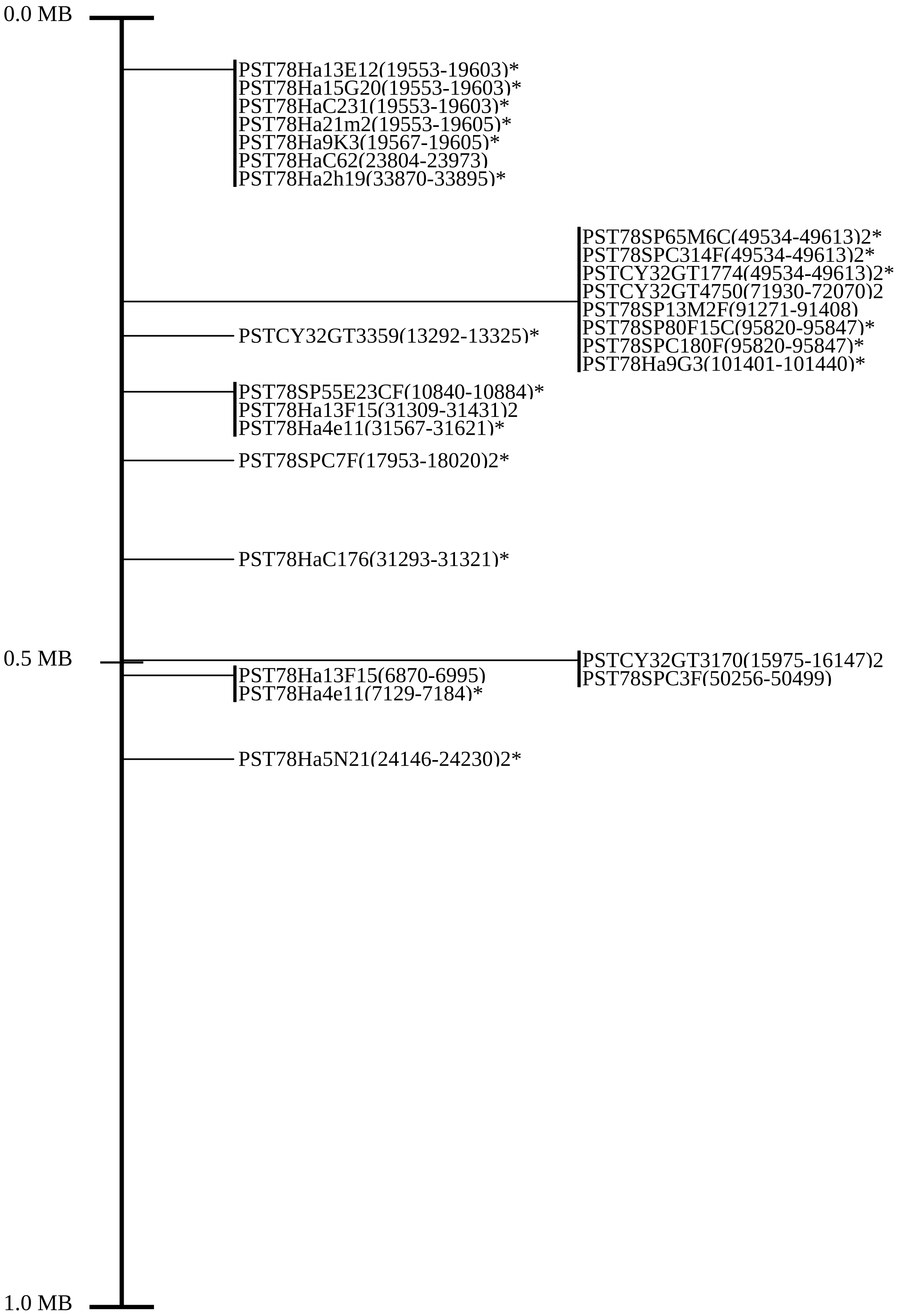

Supercontig 51
length: 574,326 bp

*** = less than 100bp alignment** **| = clones in the same contig**

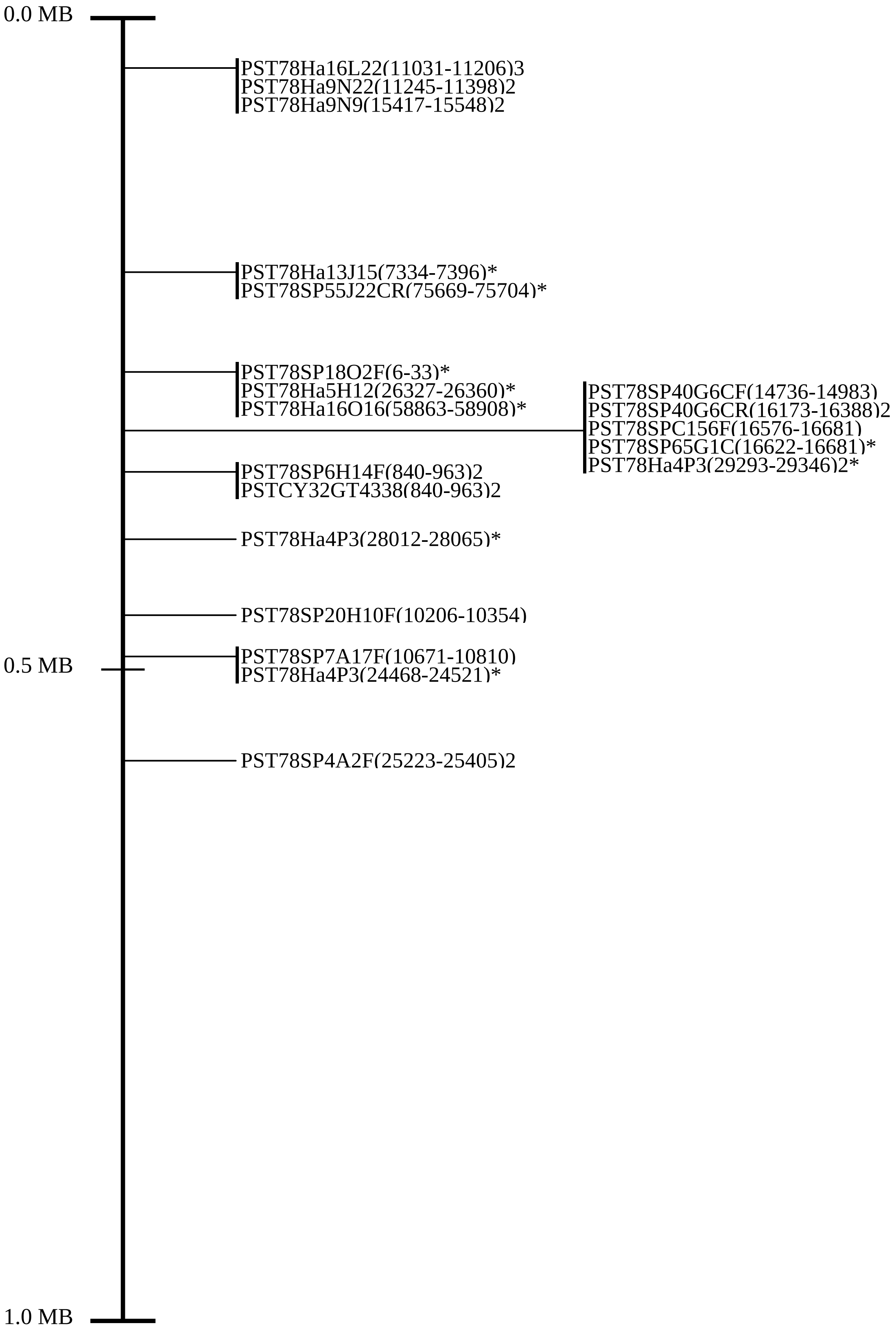

Supercontig 52
length: 572,077 bp

*** = less than 100bp alignment** **| = clones in the same contig**

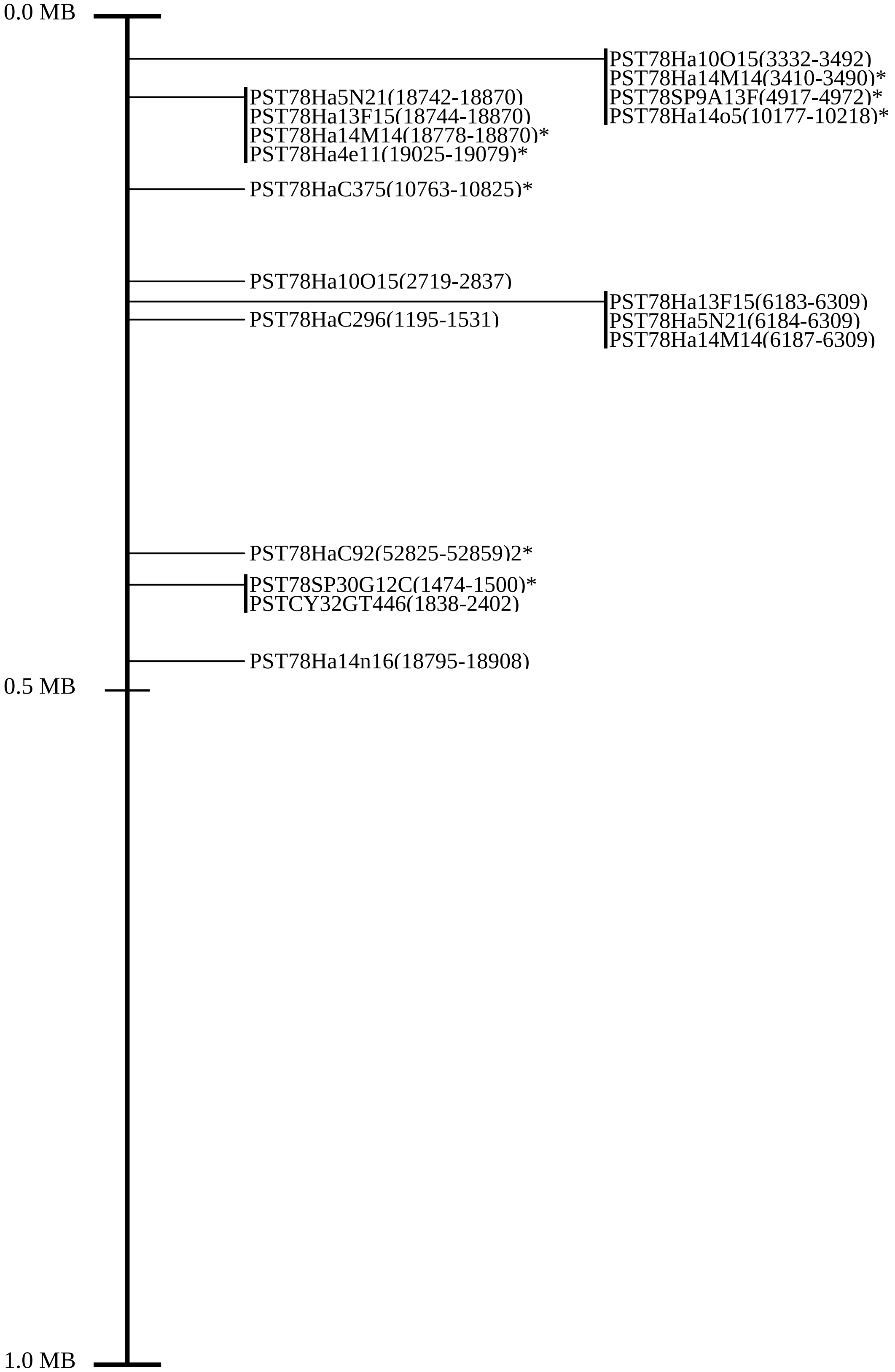

Supercontig 53
length: 591,244 bp

*** = less than 100bp alignment**

| = clones in the same contig

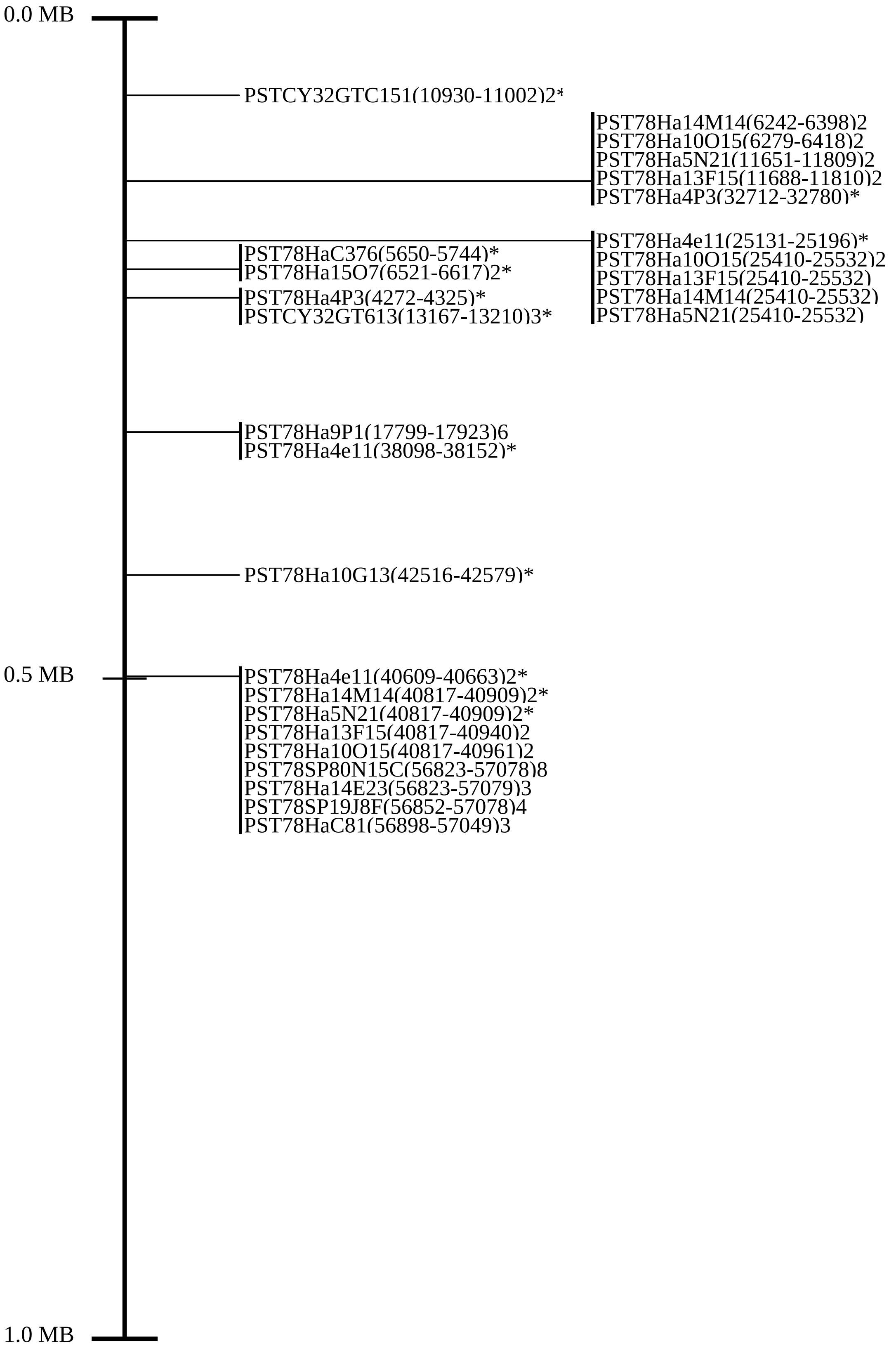

Supercontig 54
length: 525,265 bp

*** = less than 100bp alignment** **| = clones in the same contig**

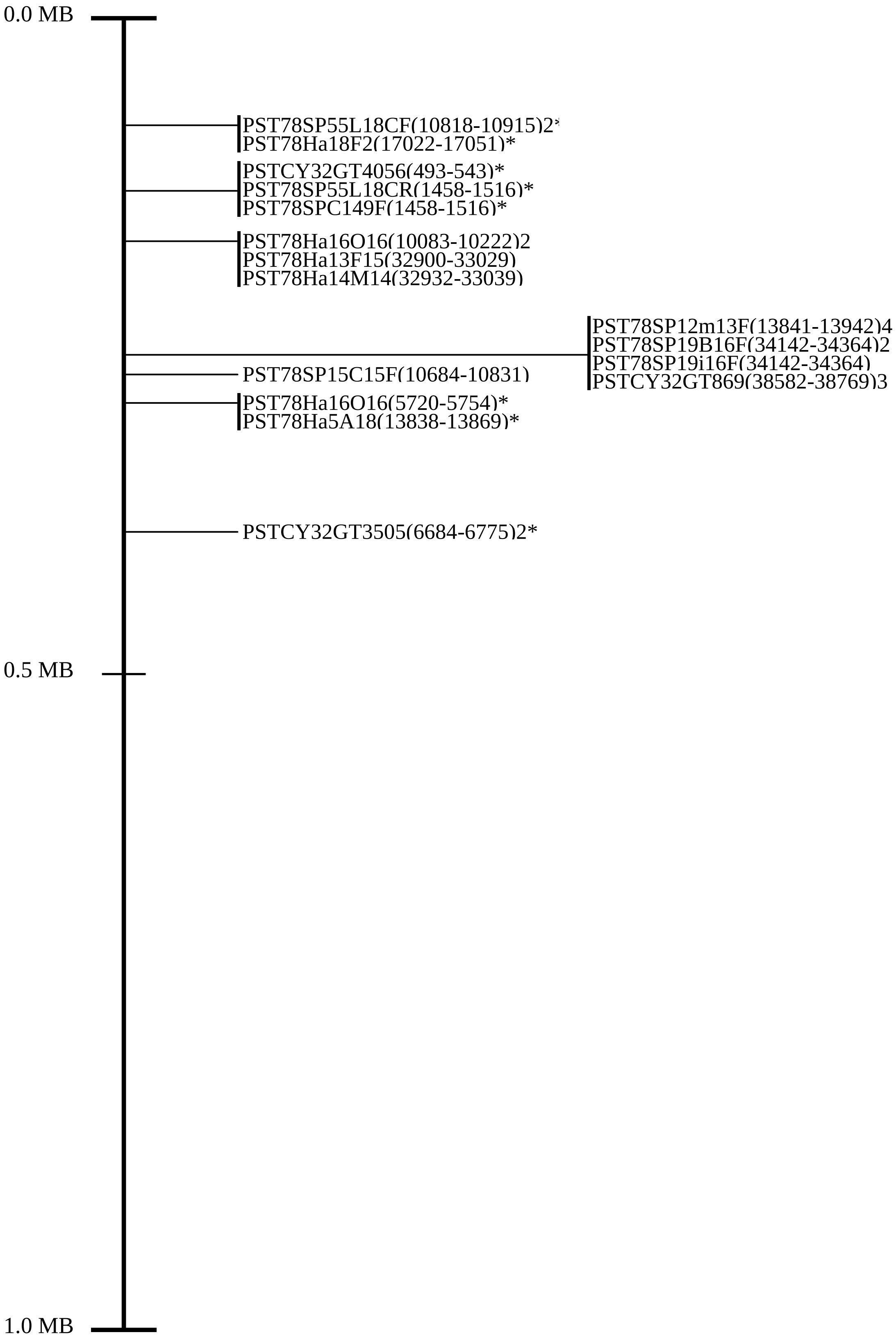

Supercontig 55
length: 542,982 bp

*** = less than 100bp alignment** | **| = clones in the same contig**

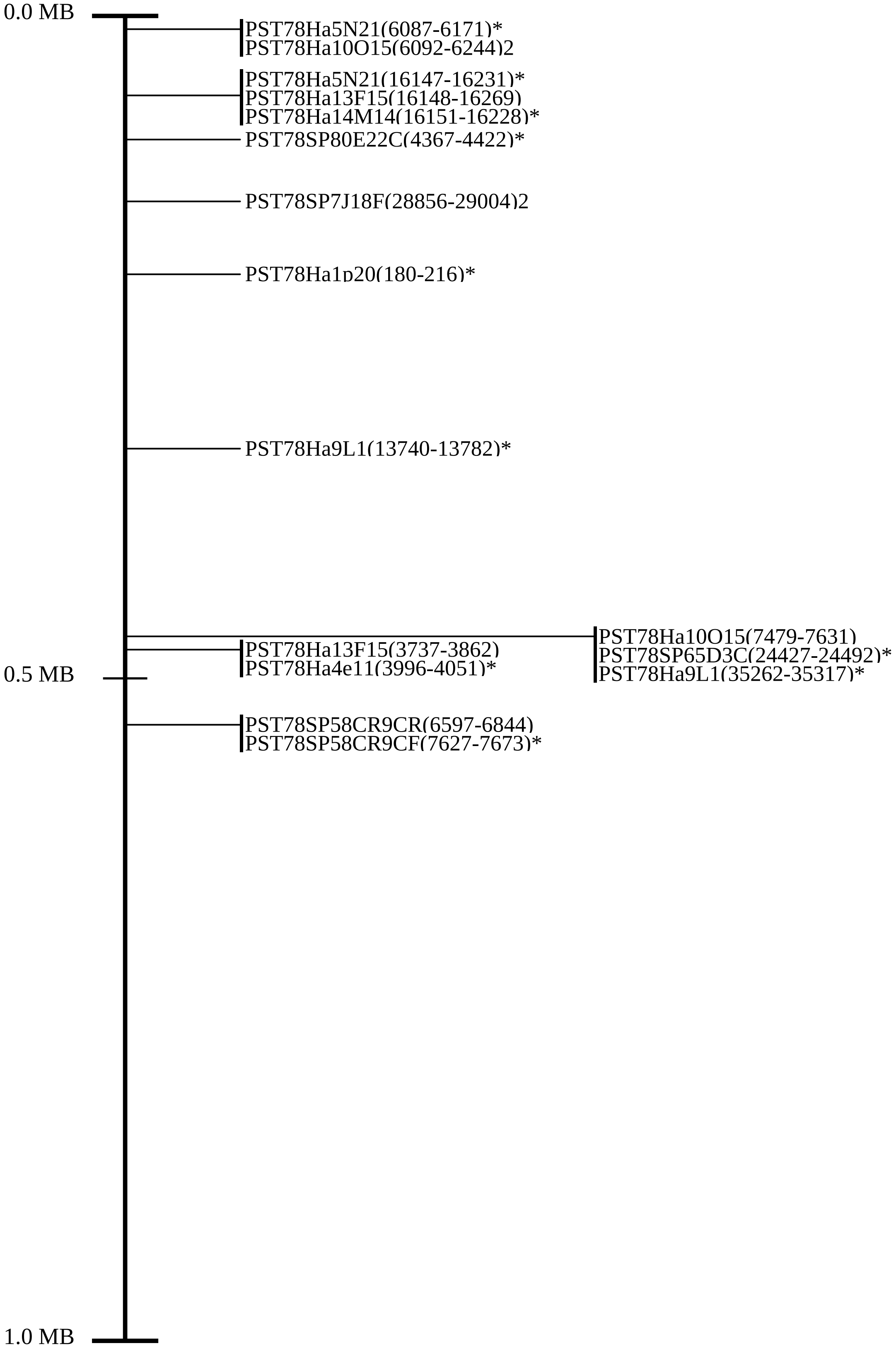

Supercontig 56
length: 577,102 bp

*** = less than 100bp alignment** **| = clones in the same contig**

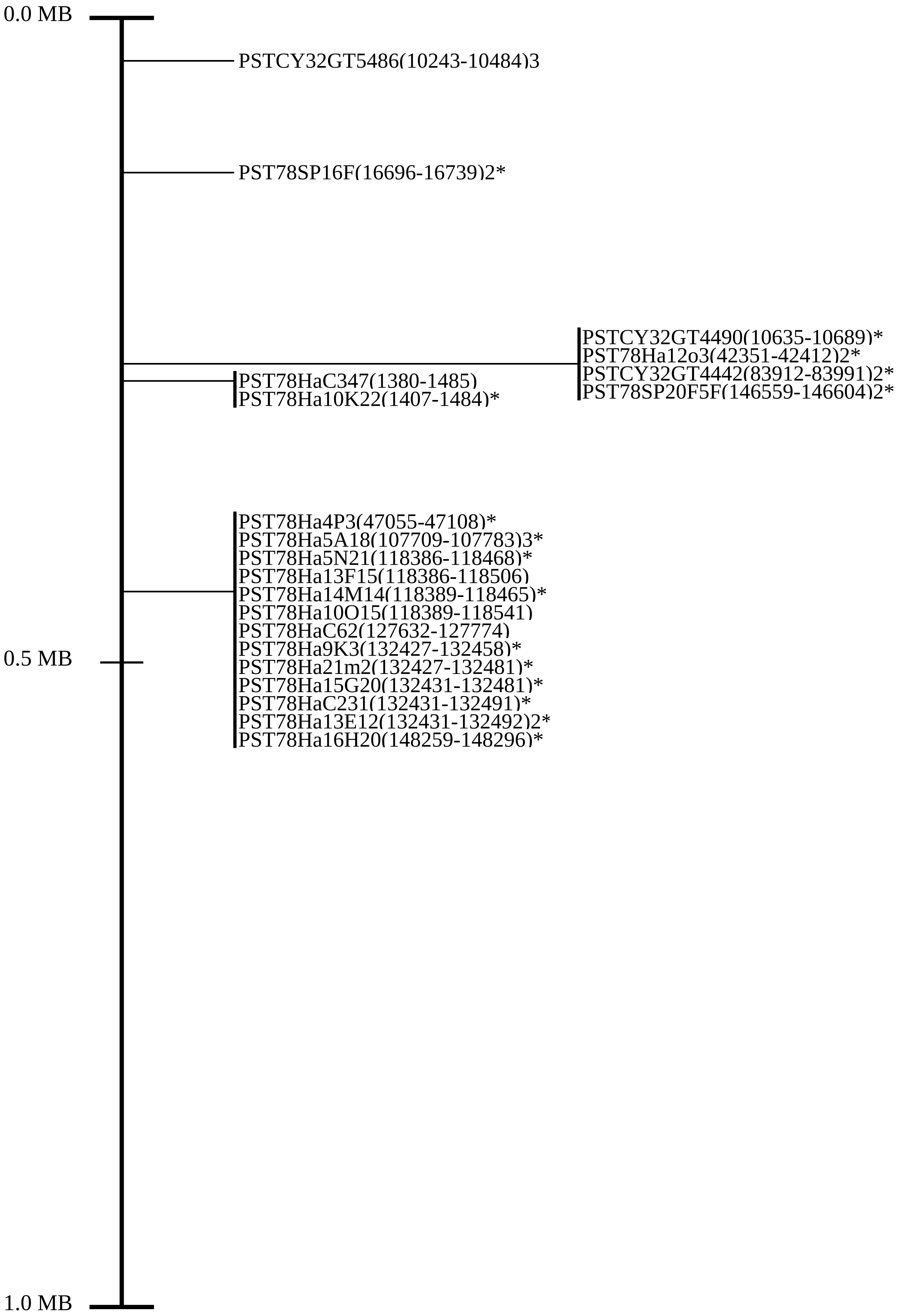

Supercontig 57
length: 480,201 bp

*** = less than 100bp alignment** **| = clones in the same contig**

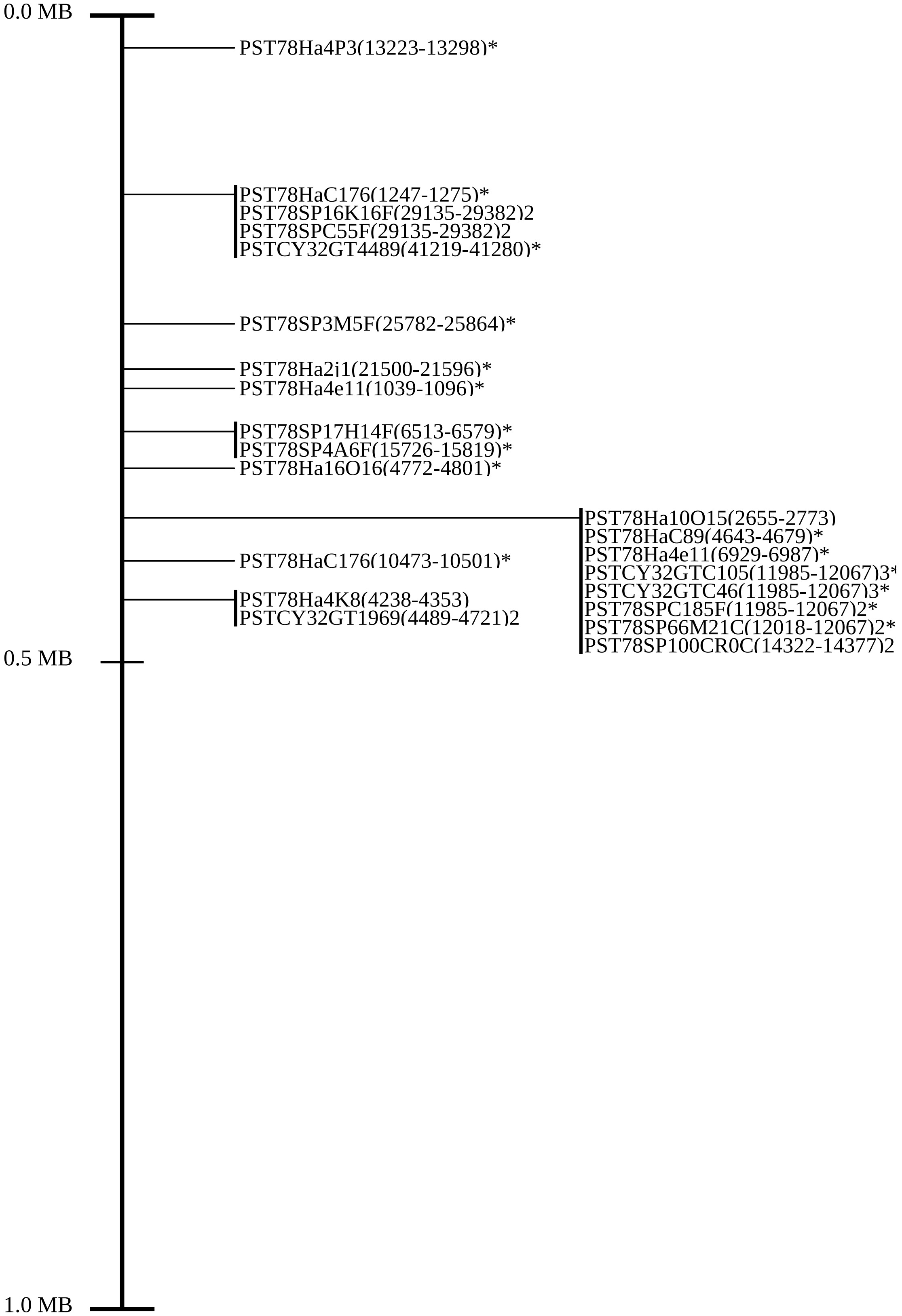

Supercontig 58
length: 496,650 bp

*** = less than 100bp alignment** **| = clones in the same contig**

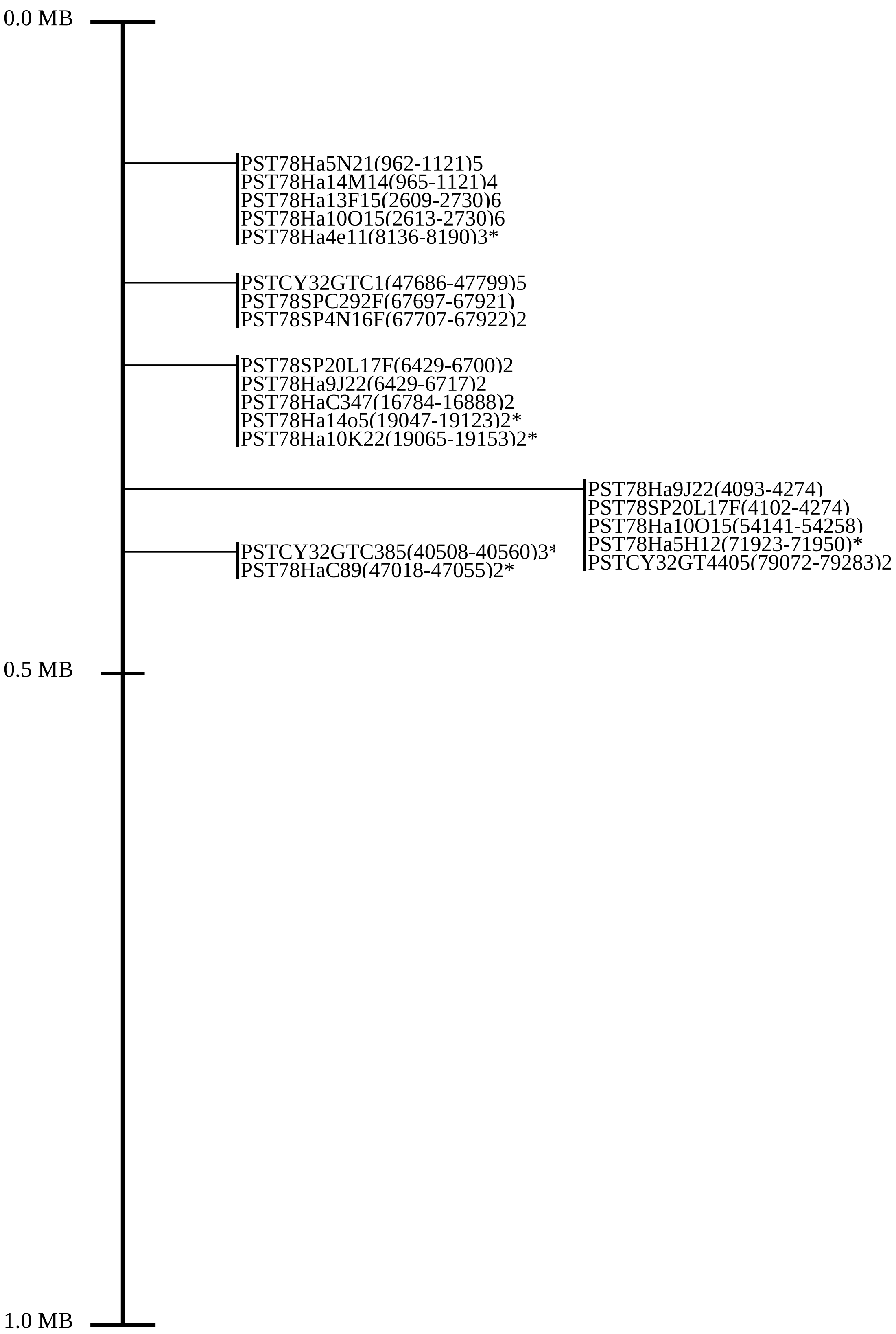

Supercontig 59
length: 489,205 bp

*** = less than 100bp alignment** **| = clones in the same contig**

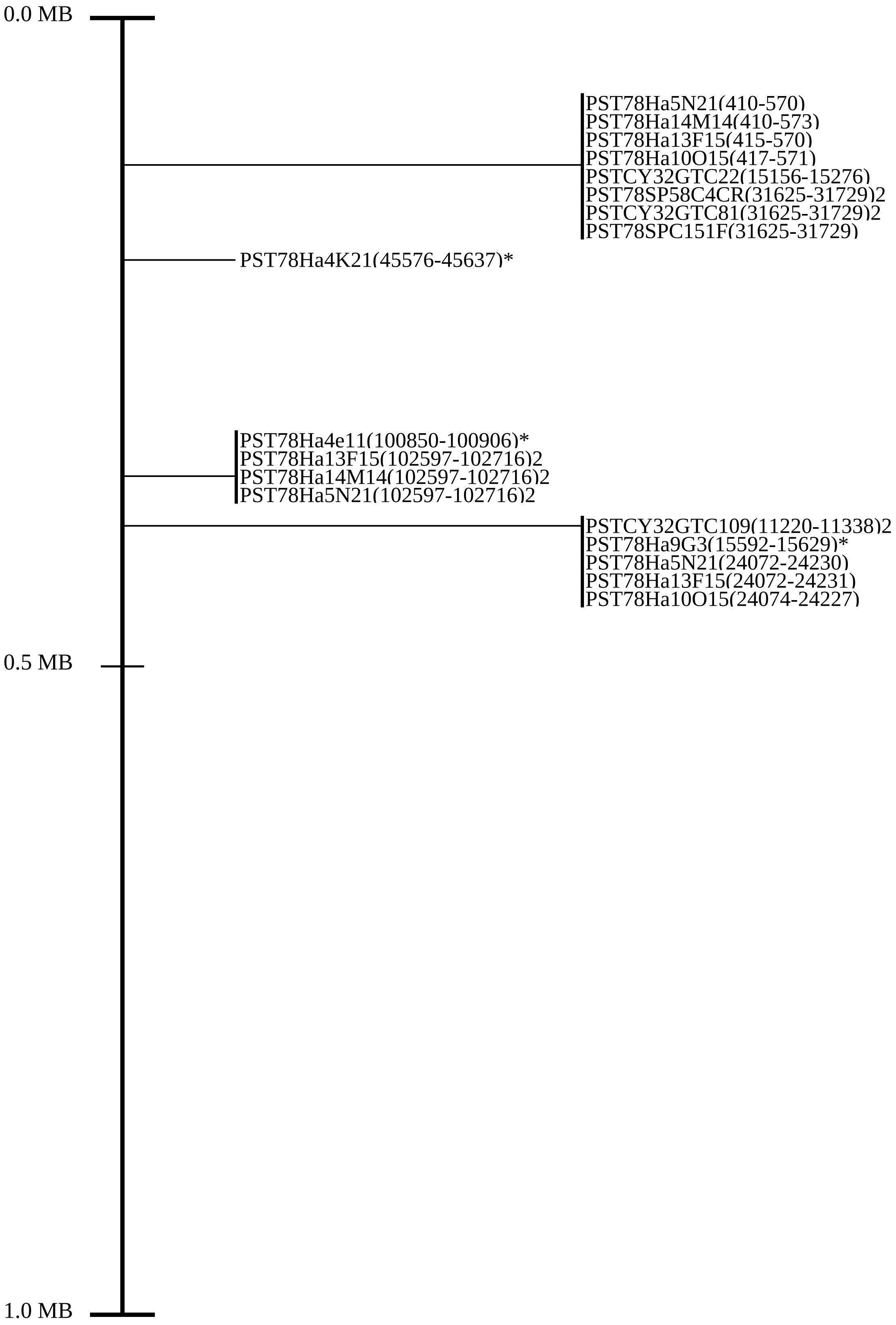

Supercontig 60
length: 436,003 bp

*** = less than 100bp alignment** **| = clones in the same contig**

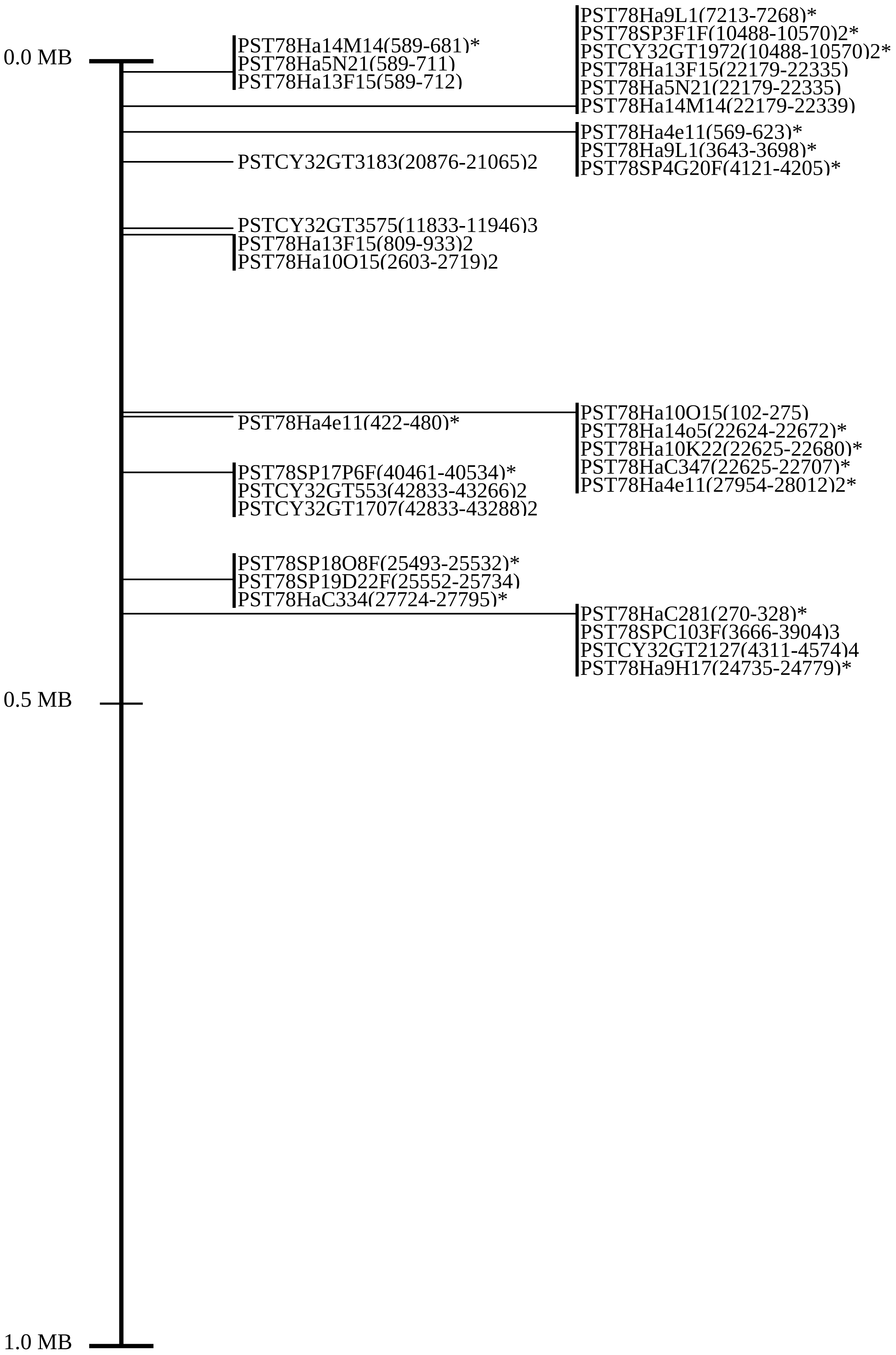

Supercontig 61
length: 469,309 bp

*** = less than 100bp alignment** **| = clones in the same contig**

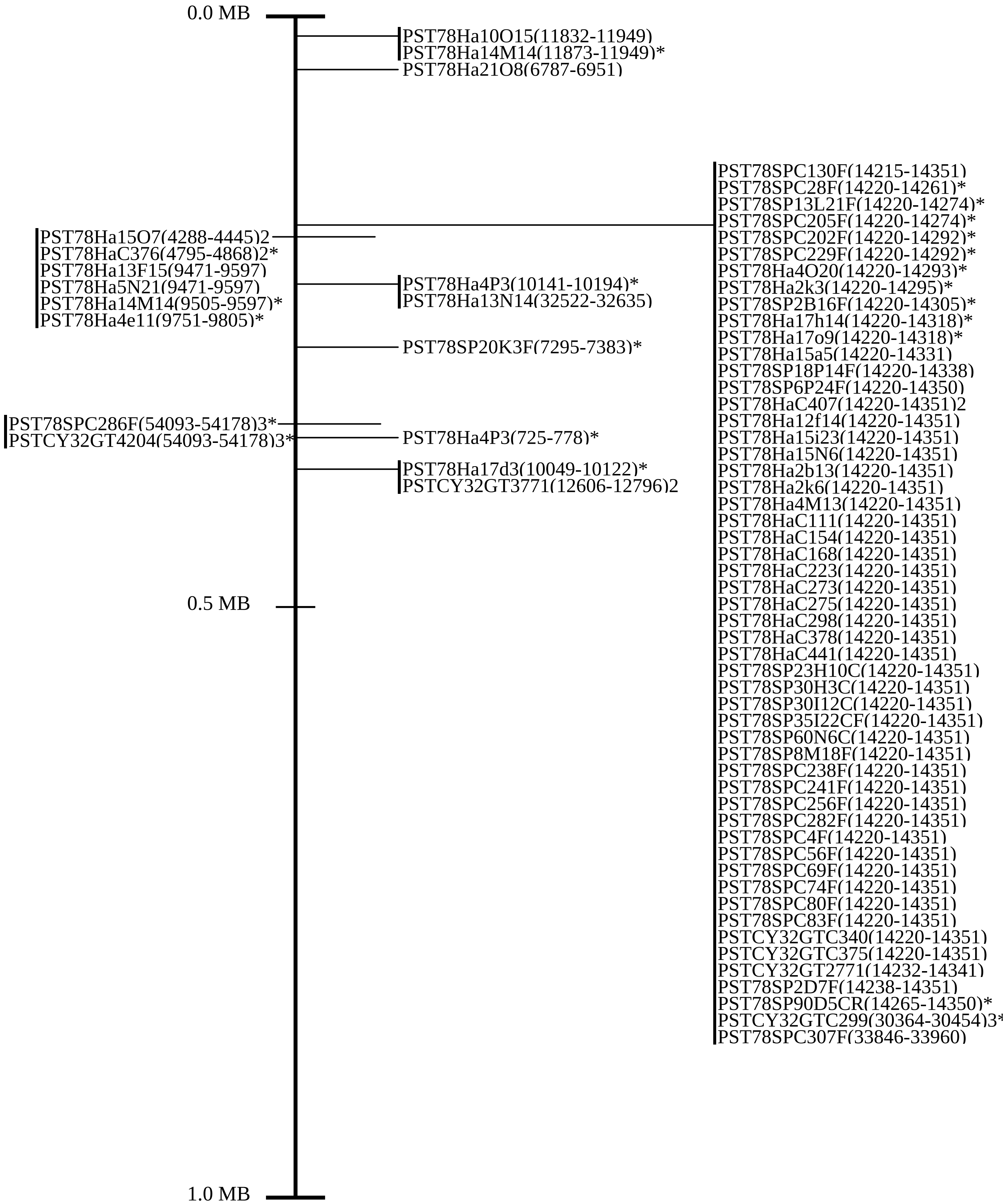

Supercontig 62
length: 428,160 bp

*** = less than 100bp alignment** **| = clones in the same contig**

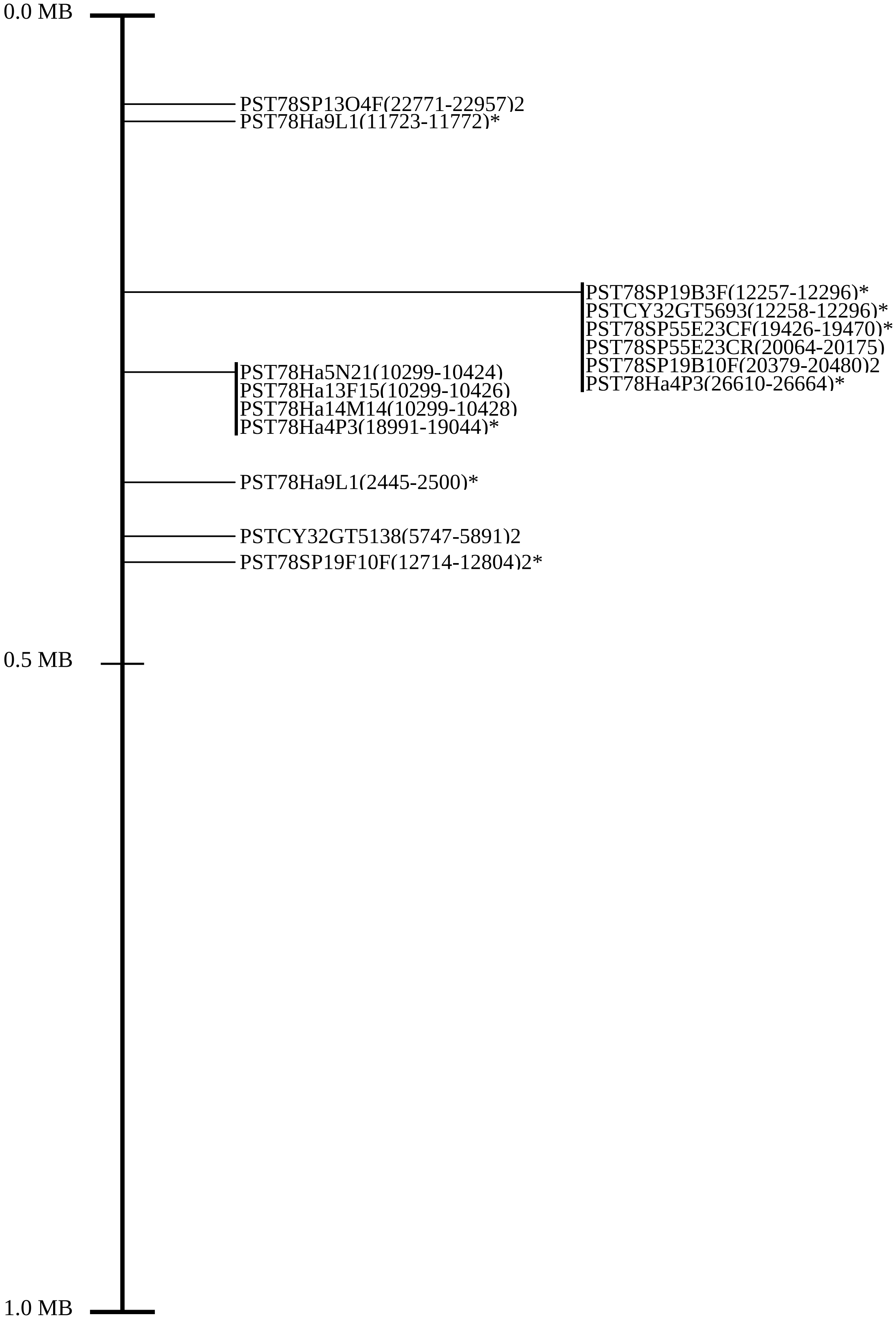

Supercontig 63
length: 433,102 bp

*** = less than 100bp alignment** **| = clones in the same contig**

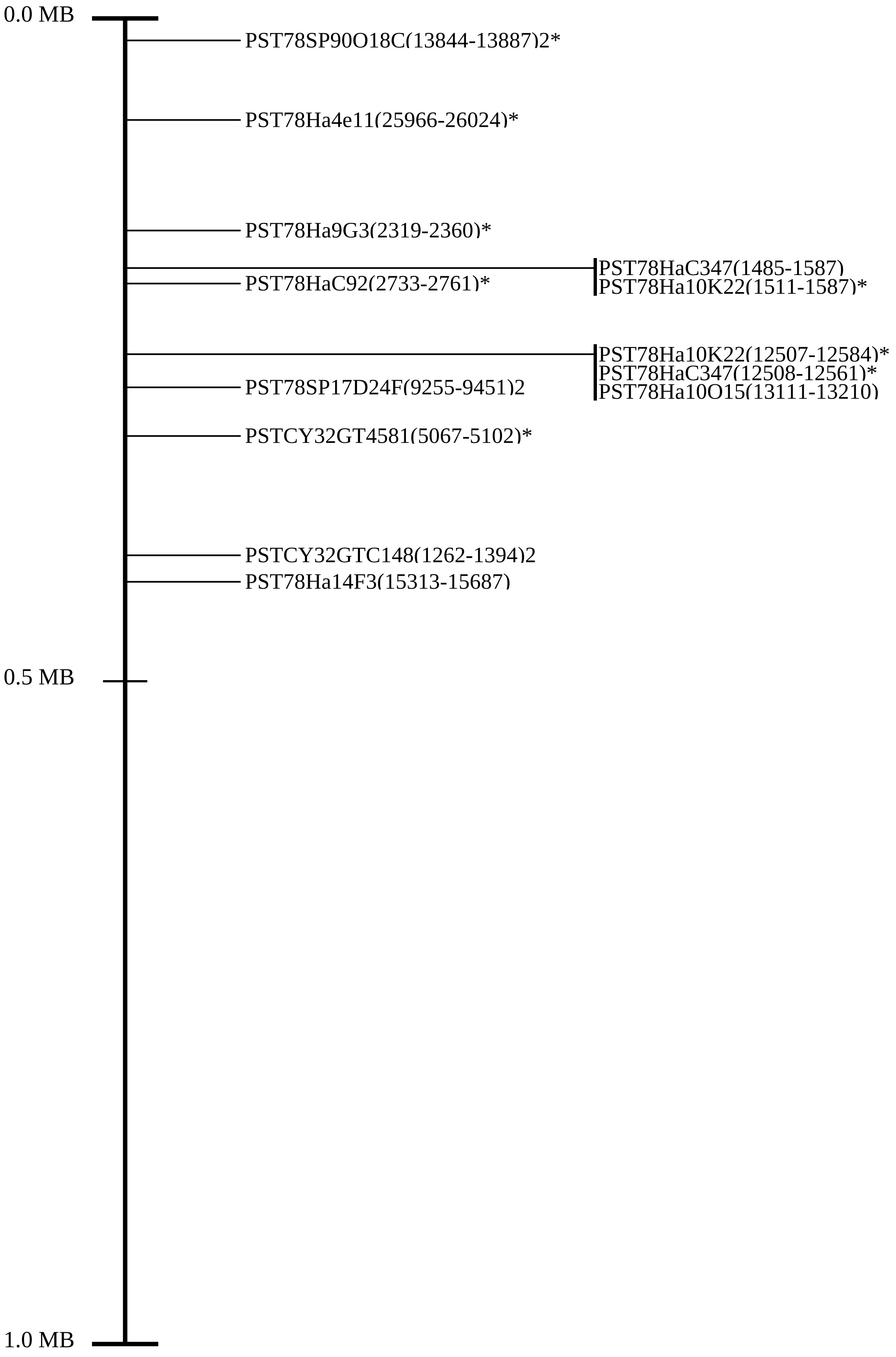

Supercontig 64
length: 440,512 bp

*** = less than 100bp alignment** **| = clones in the same contig**

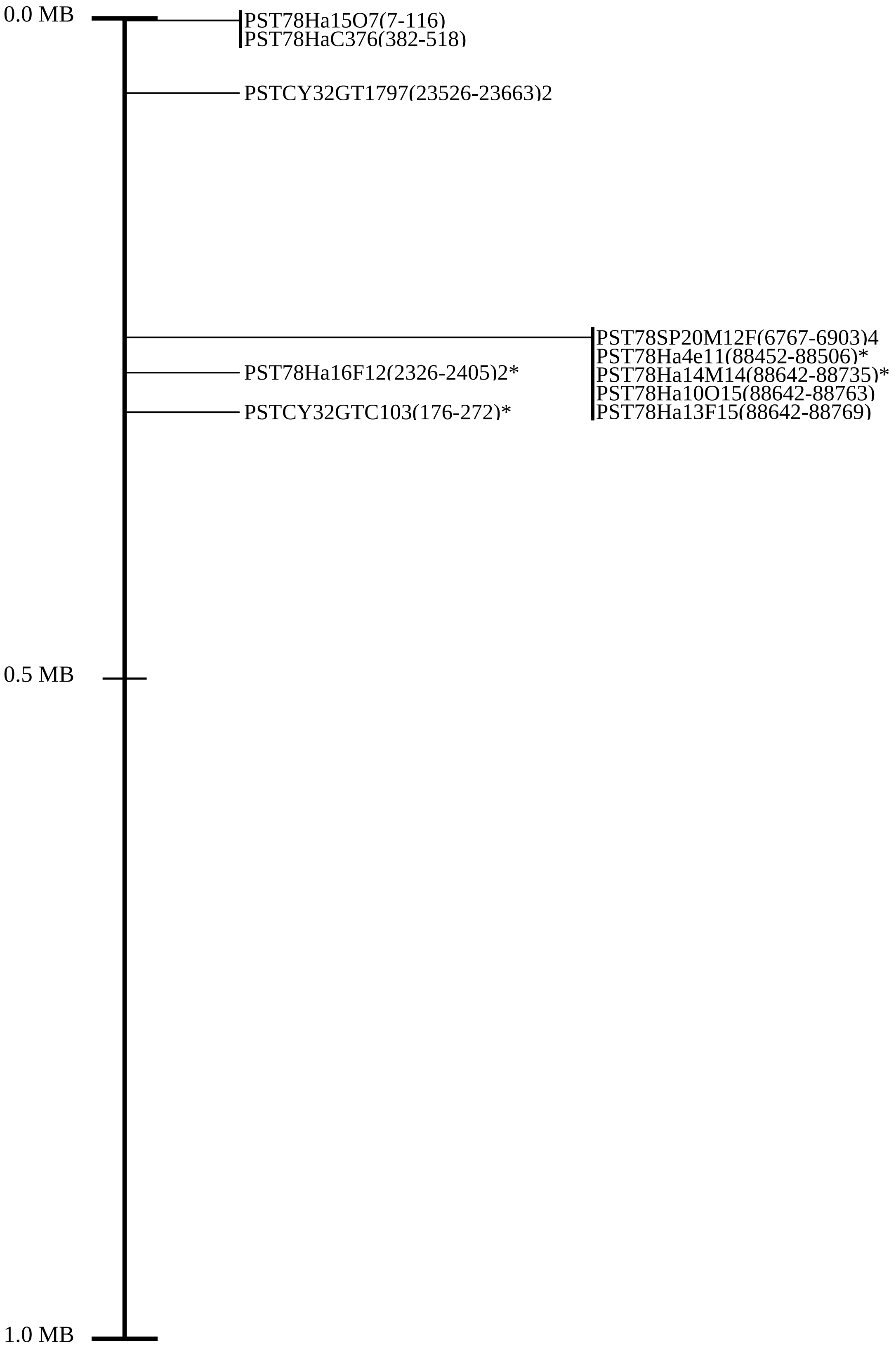

Supercontig 65
length: 407,335 bp

*** = less than 100bp alignment** **| = clones in the same contig**

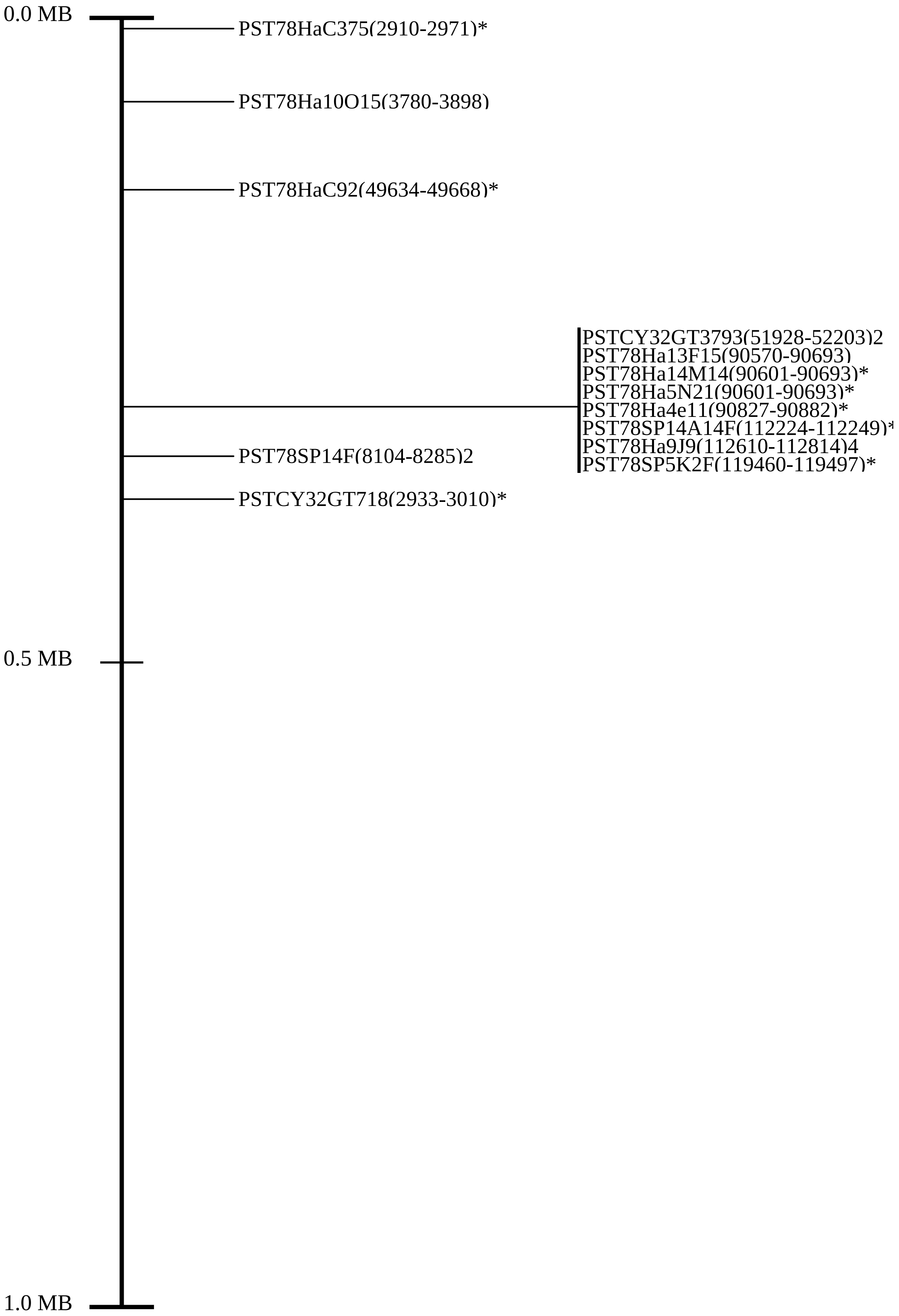

Supercontig 66
length: 388,993 bp

*** = less than 100bp alignment** **| = clones in the same contig**

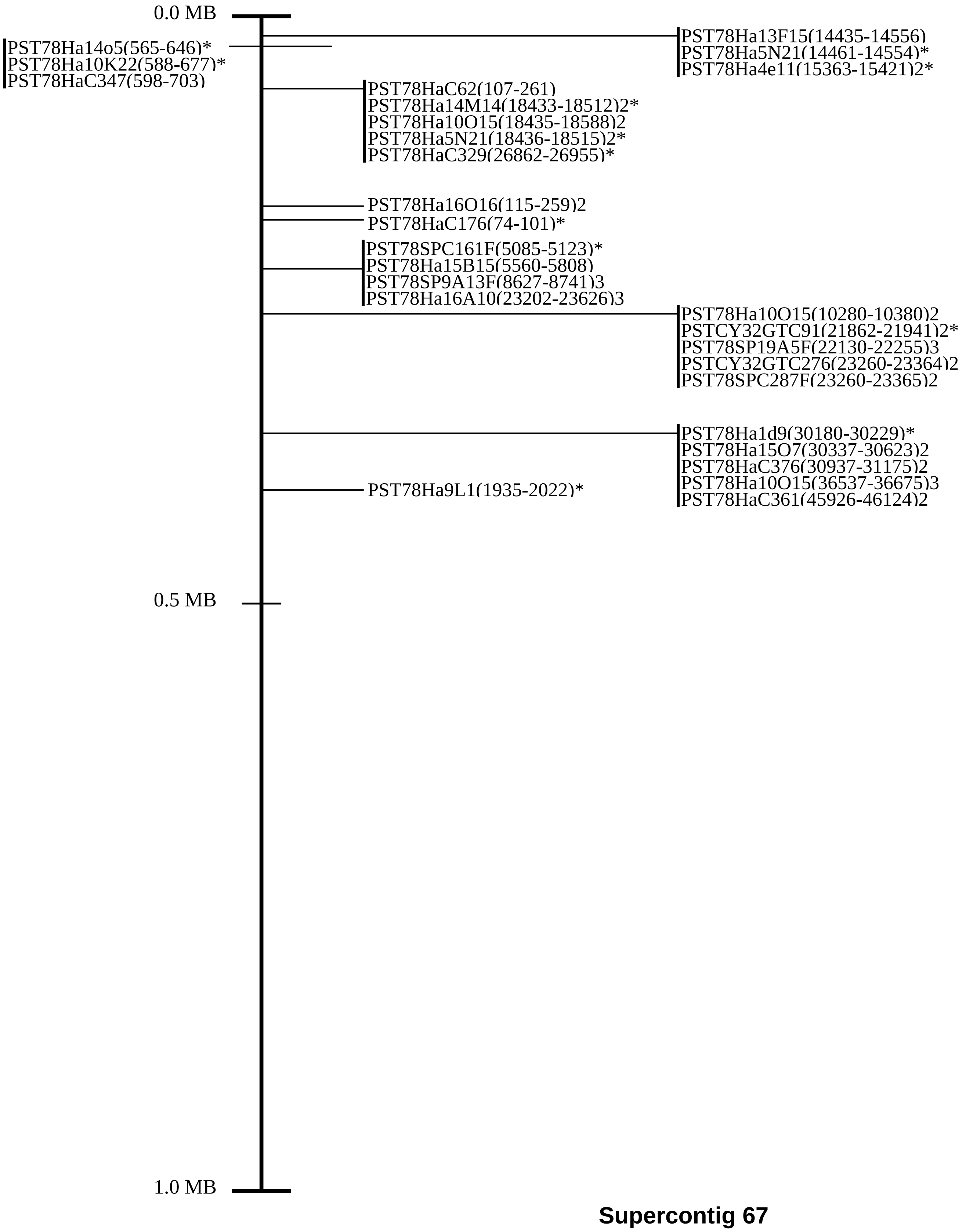

Supercontig 67
length: 403,504 bp

*** = less than 100bp alignment**

| = clones in the same contig

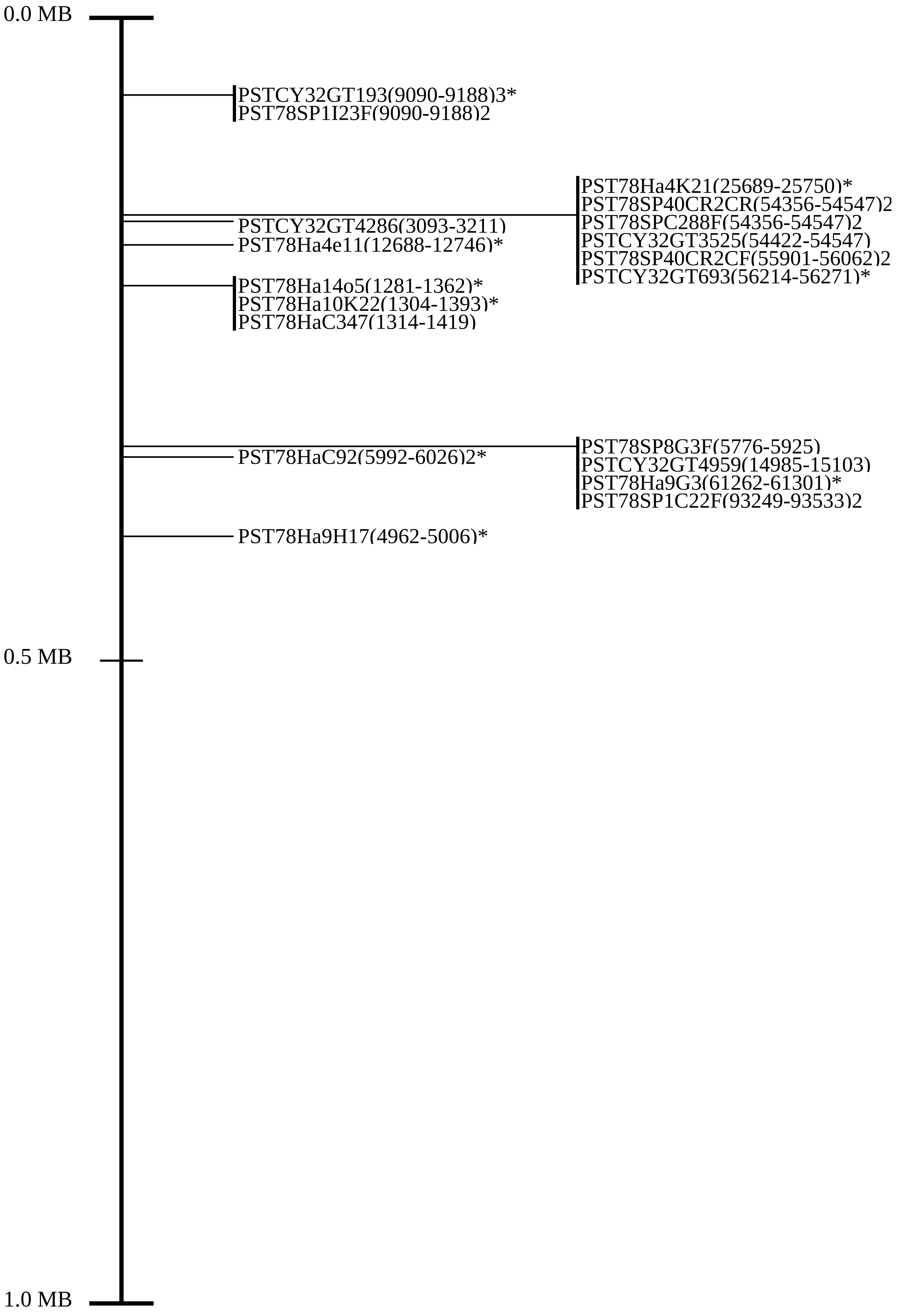

Supercontig 68
length: 403,089 bp

*** = less than 100bp alignment** **| = clones in the same contig**

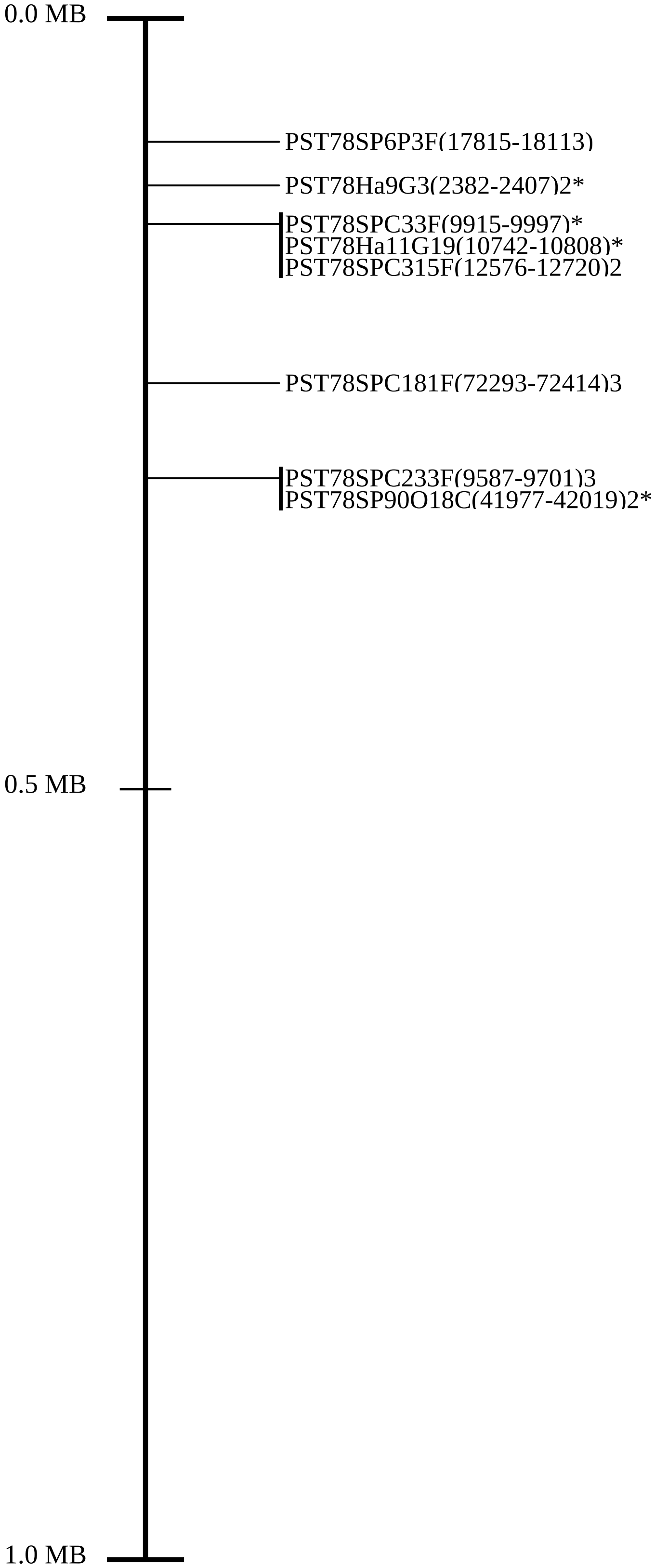

Supercontig 69
length: 367,522 bp

*** = less than 100bp alignment**

| = clones in the same contig

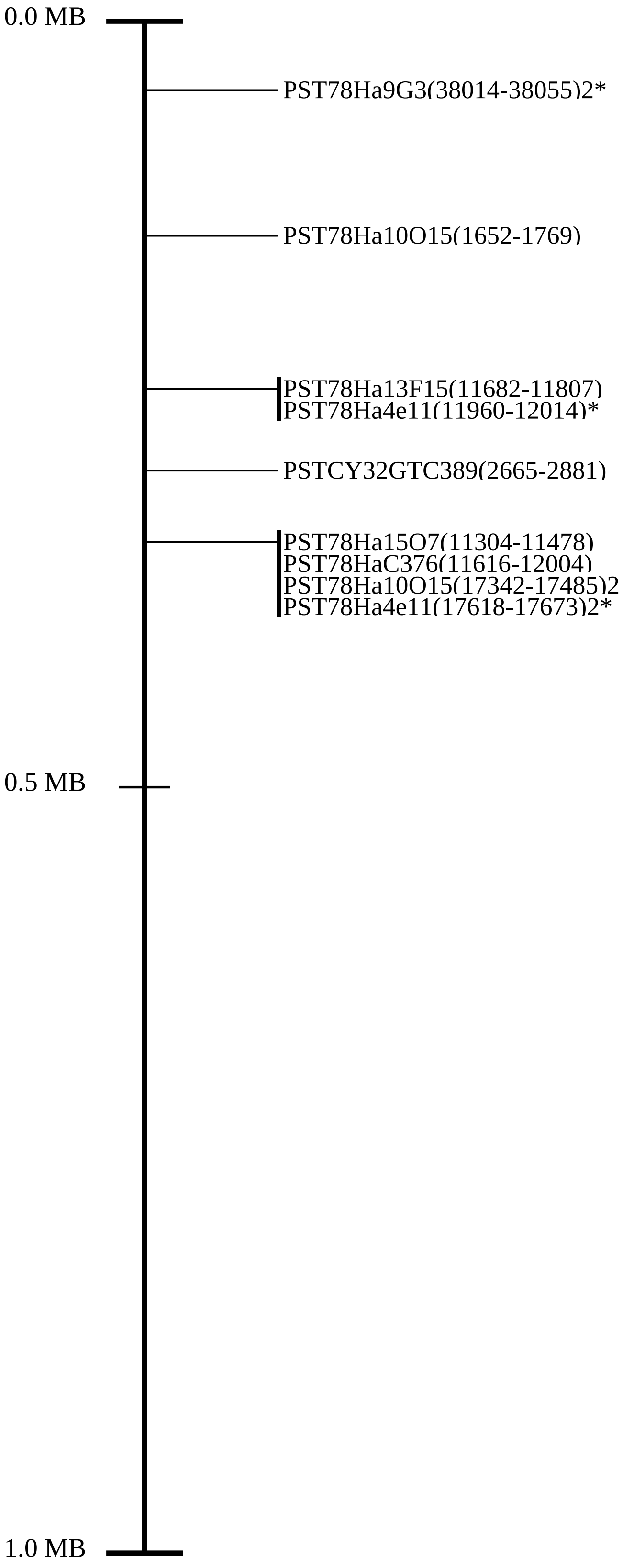

Supercontig 70
length: 386,059 bp

*** = less than 100bp alignment** **| = clones in the same contig**

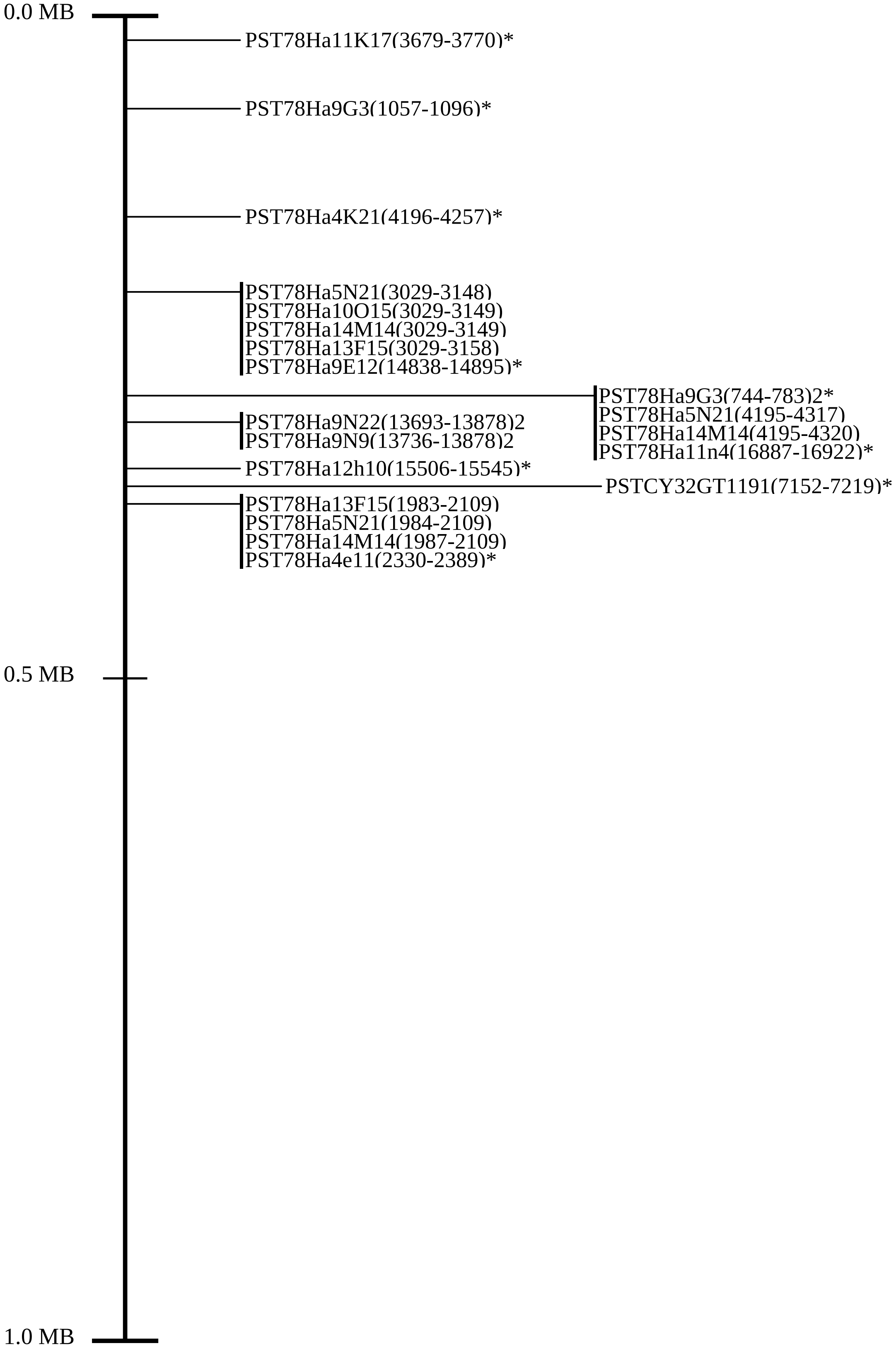

Supercontig 71
length: 392,332 bp

*** = less than 100bp alignment** **| = clones in the same contig**

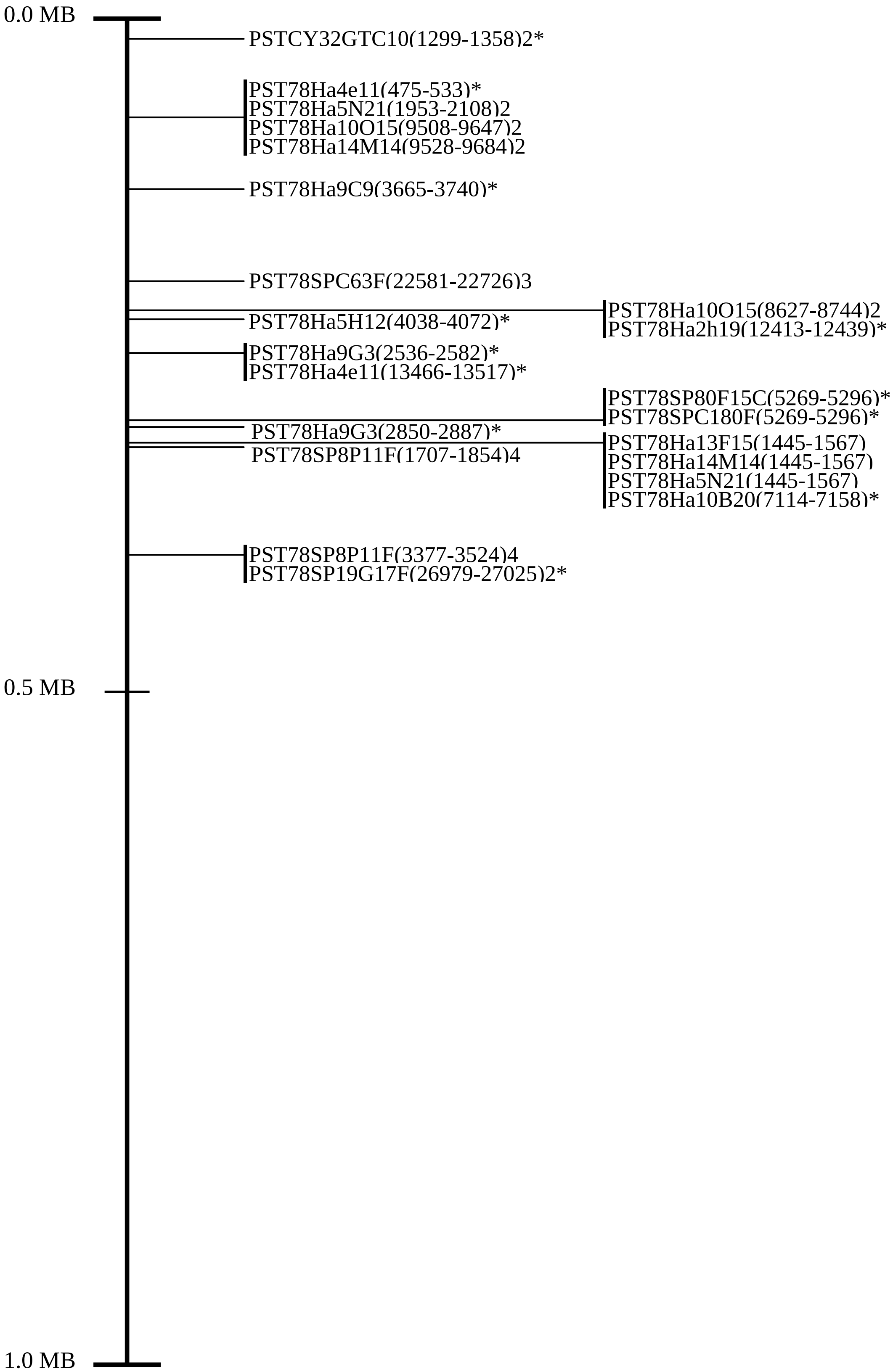

Supercontig 72
length: 398,881 bp

*** = less than 100bp alignment** **| = clones in the same contig**

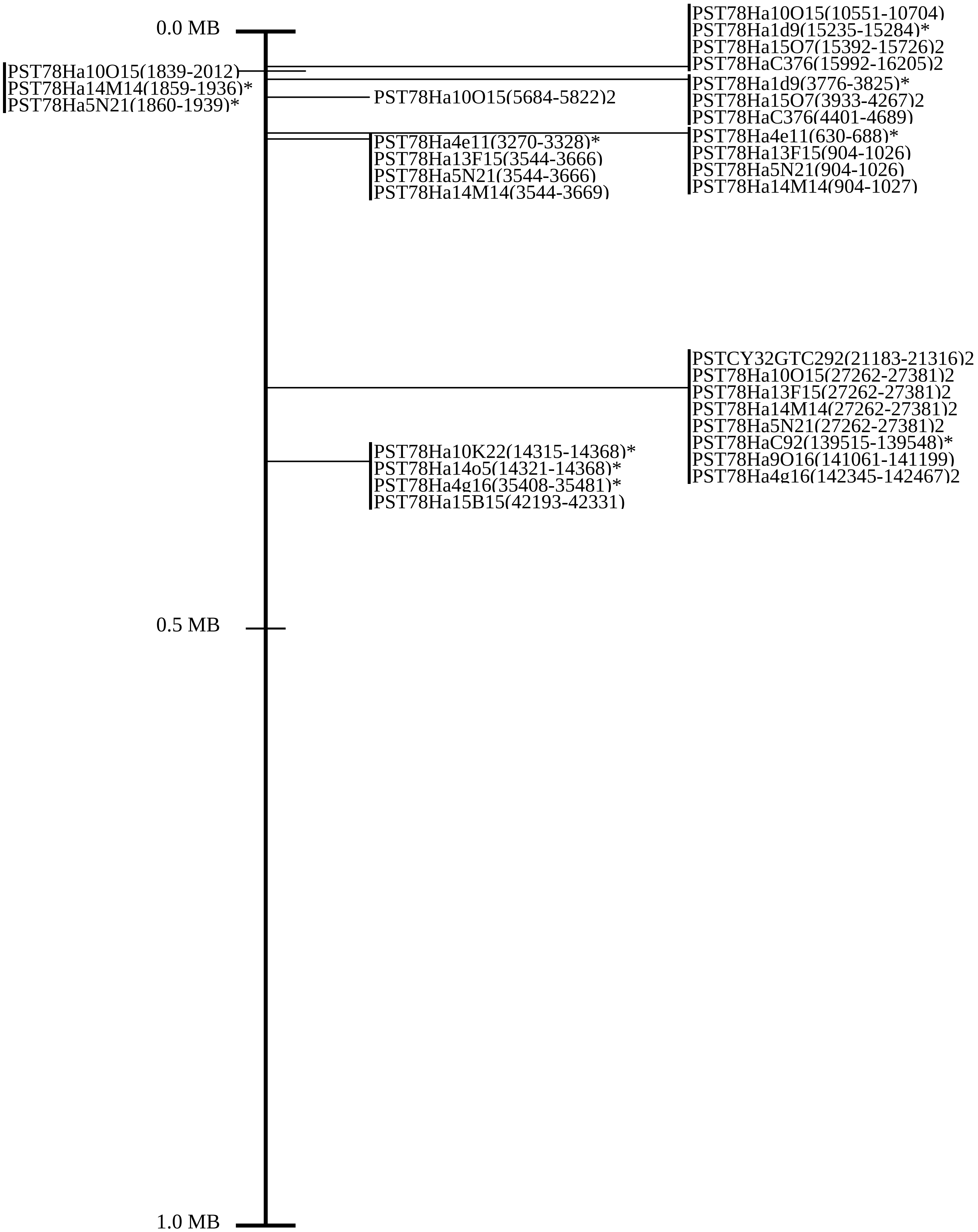

Supercontig 73
length: 360,371 bp

*** = less than 100bp alignment** **| = clones in the same contig**

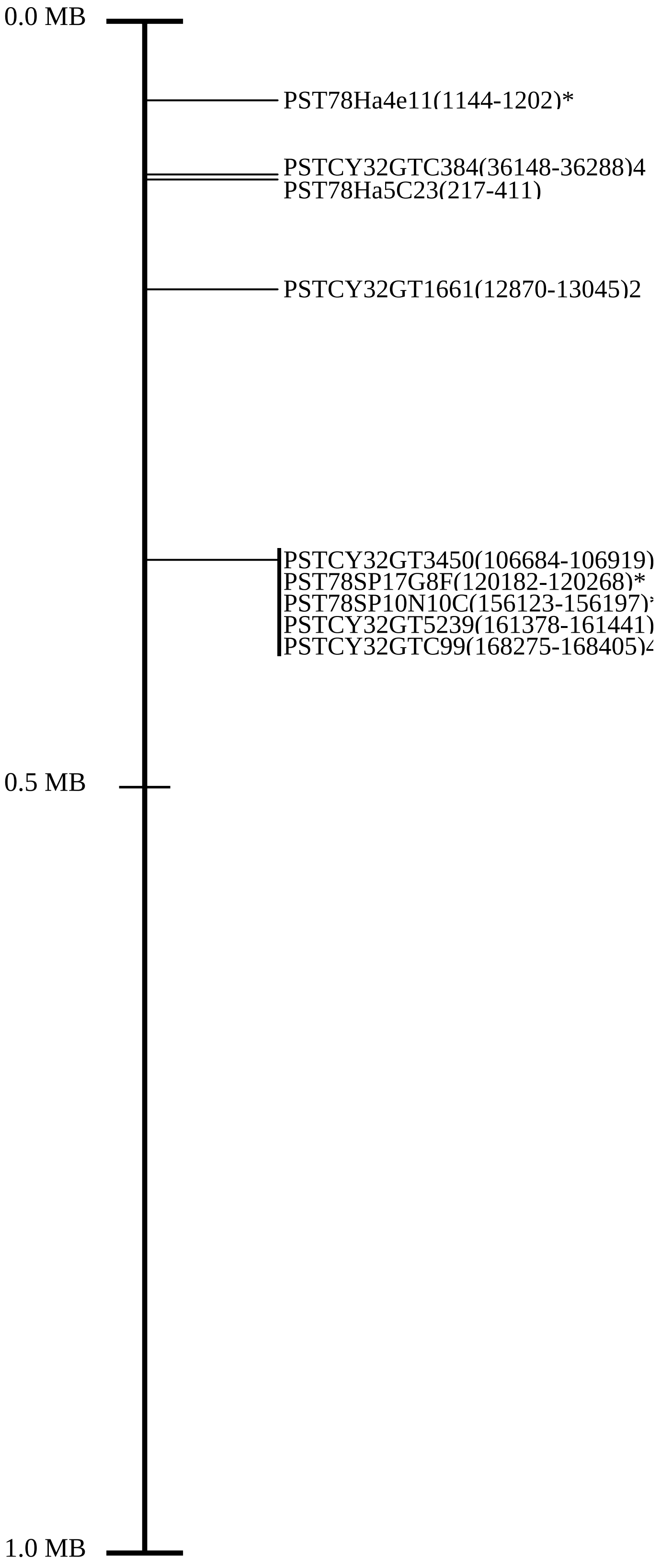

Supercontig 74
length: 351,149 bp

*** = less than 100bp alignment** **| = clones in the same contig**

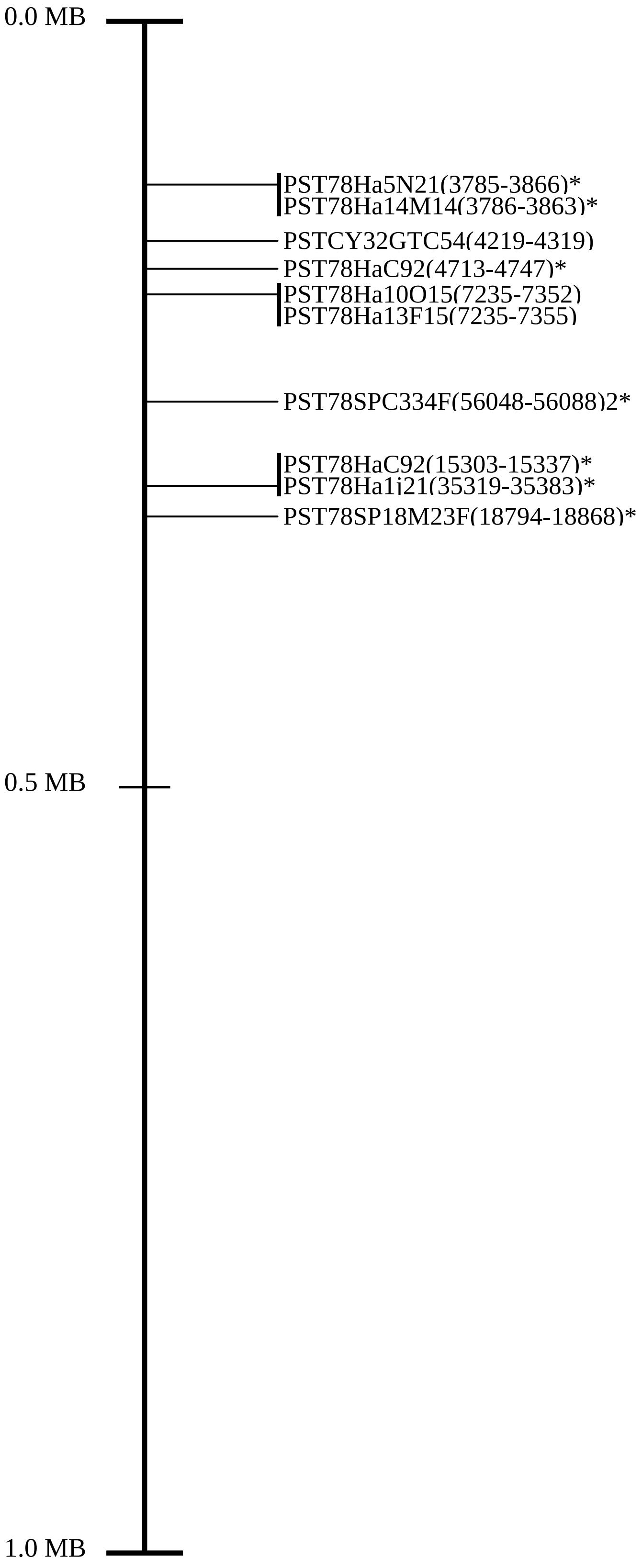

Supercontig 75
length: 350,882 bp

*** = less than 100bp alignment** **| = clones in the same contig**

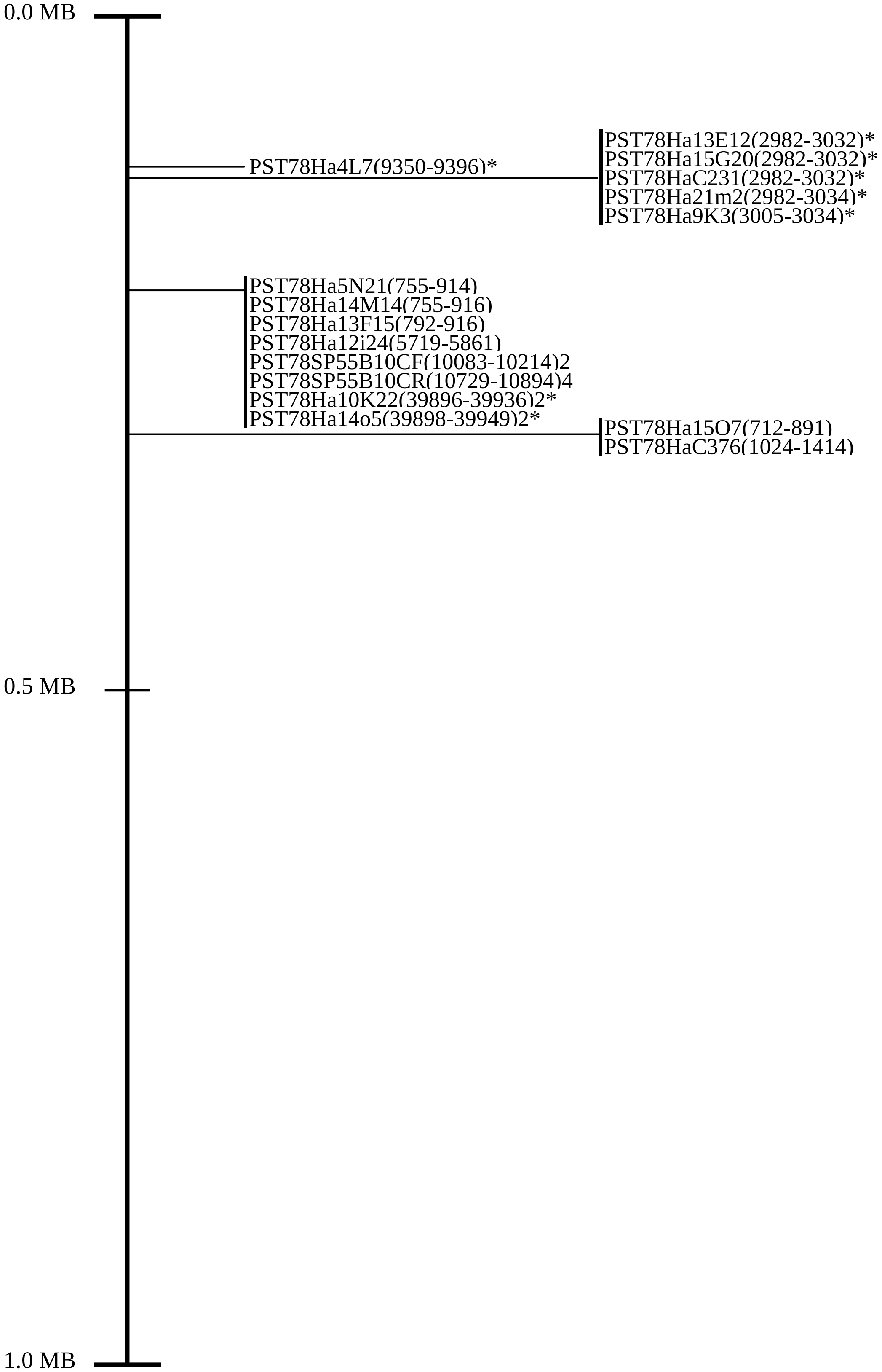

Supercontig 76
length: 341,344 bp

*** = less than 100bp alignment** **| = clones in the same contig**

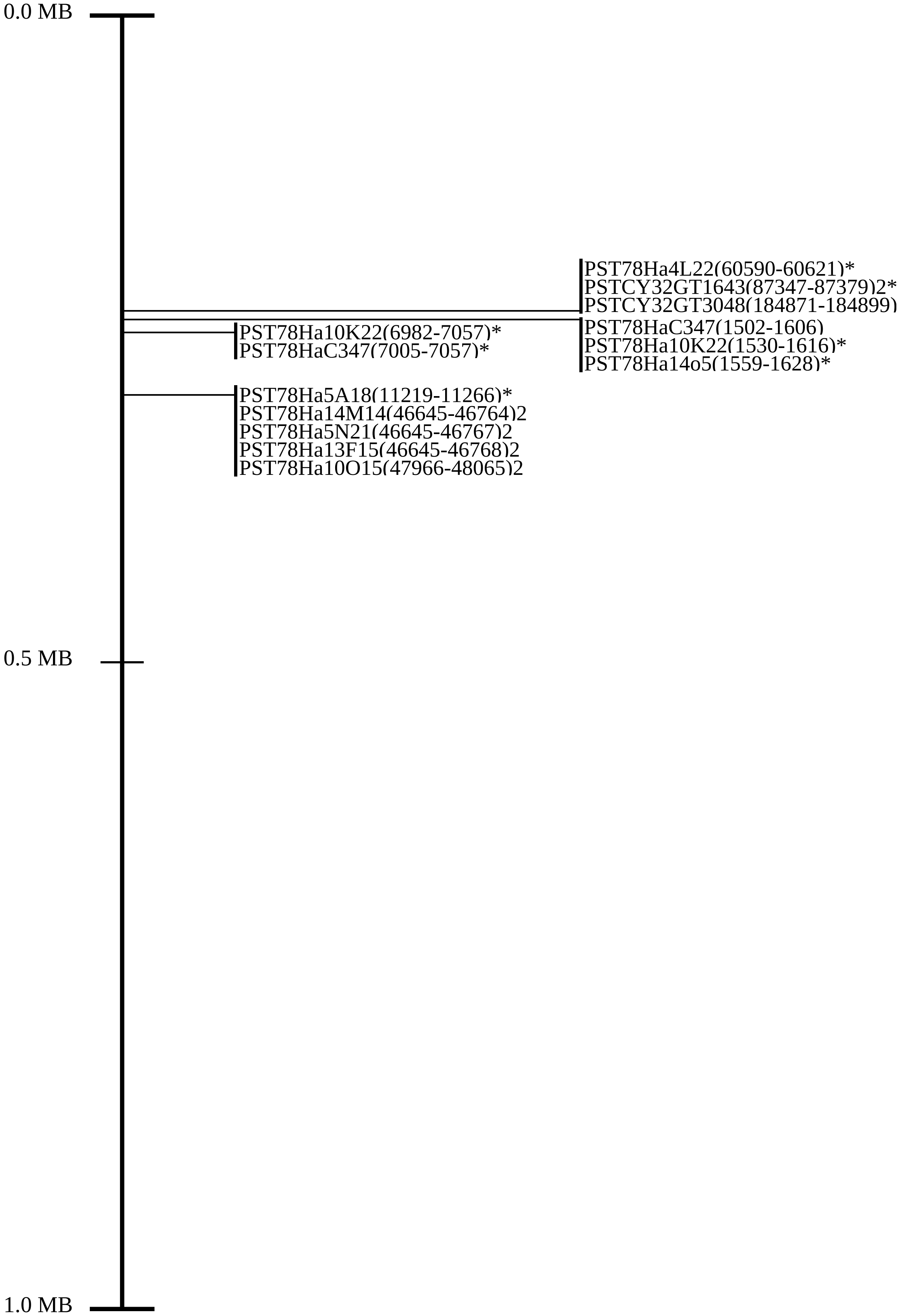

Supercontig 77
length: 304,582 bp

*** = less than 100bp alignment**

| = clones in the same contig

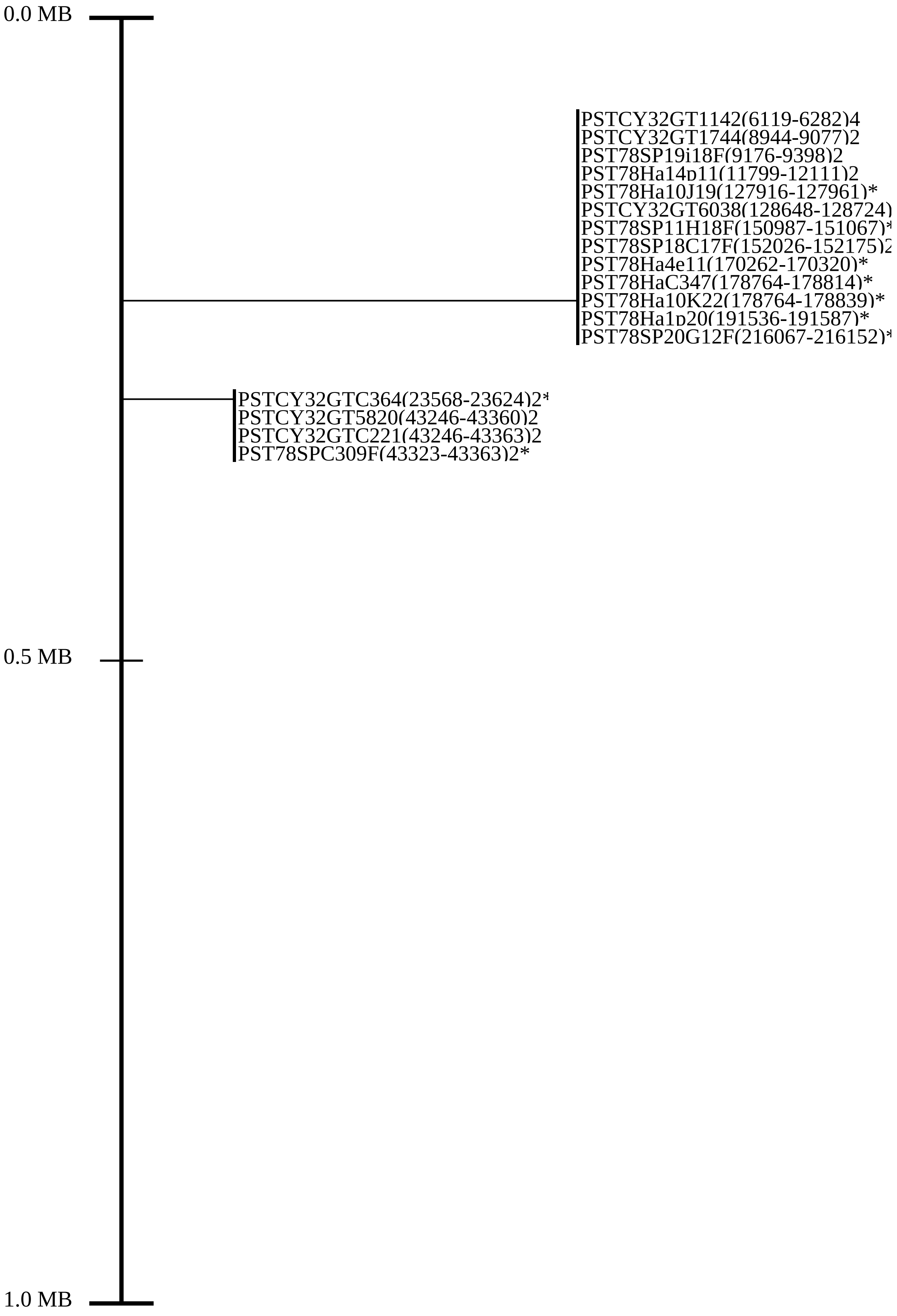

Supercontig 78
length: 296,996 bp

*** = less than 100bp alignment** **| = clones in the same contig**

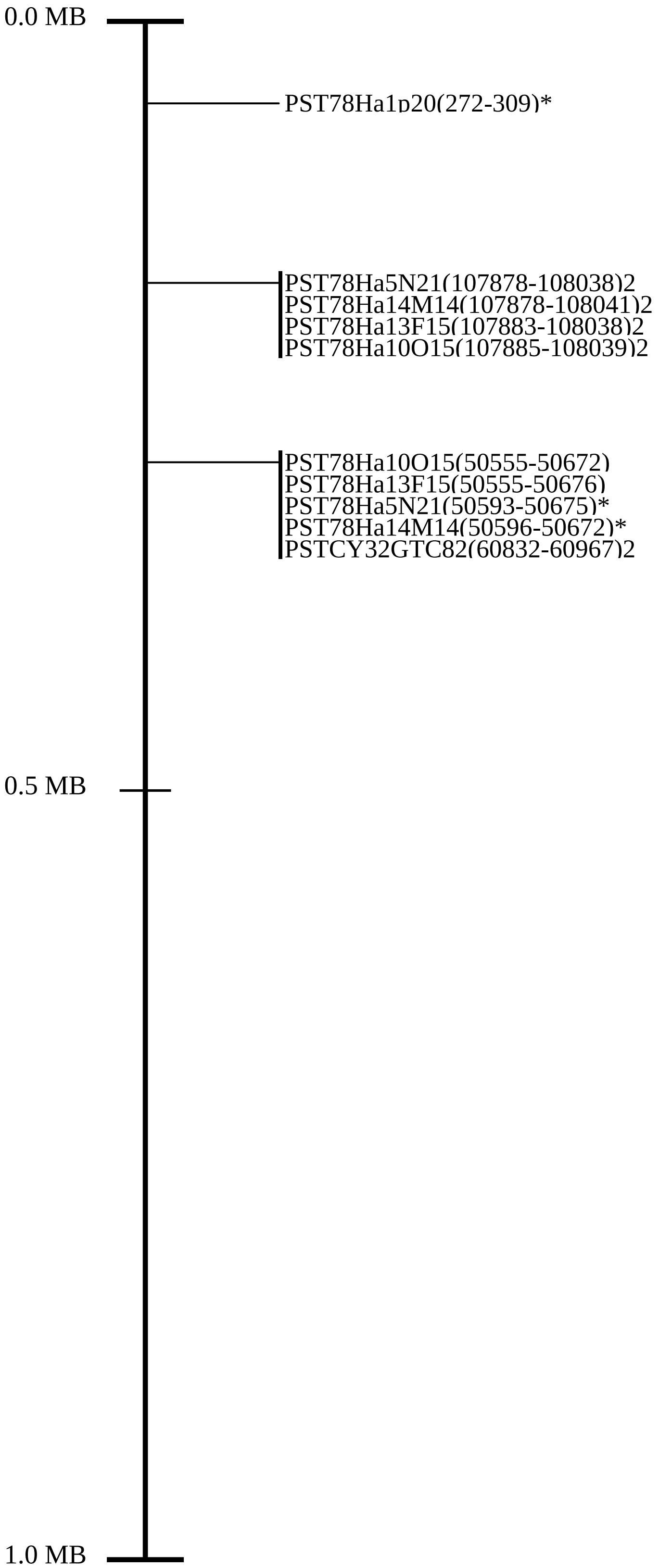

Supercontig 79
length: 286,933 bp

*** = less than 100bp alignment**

| = clones in the same contig

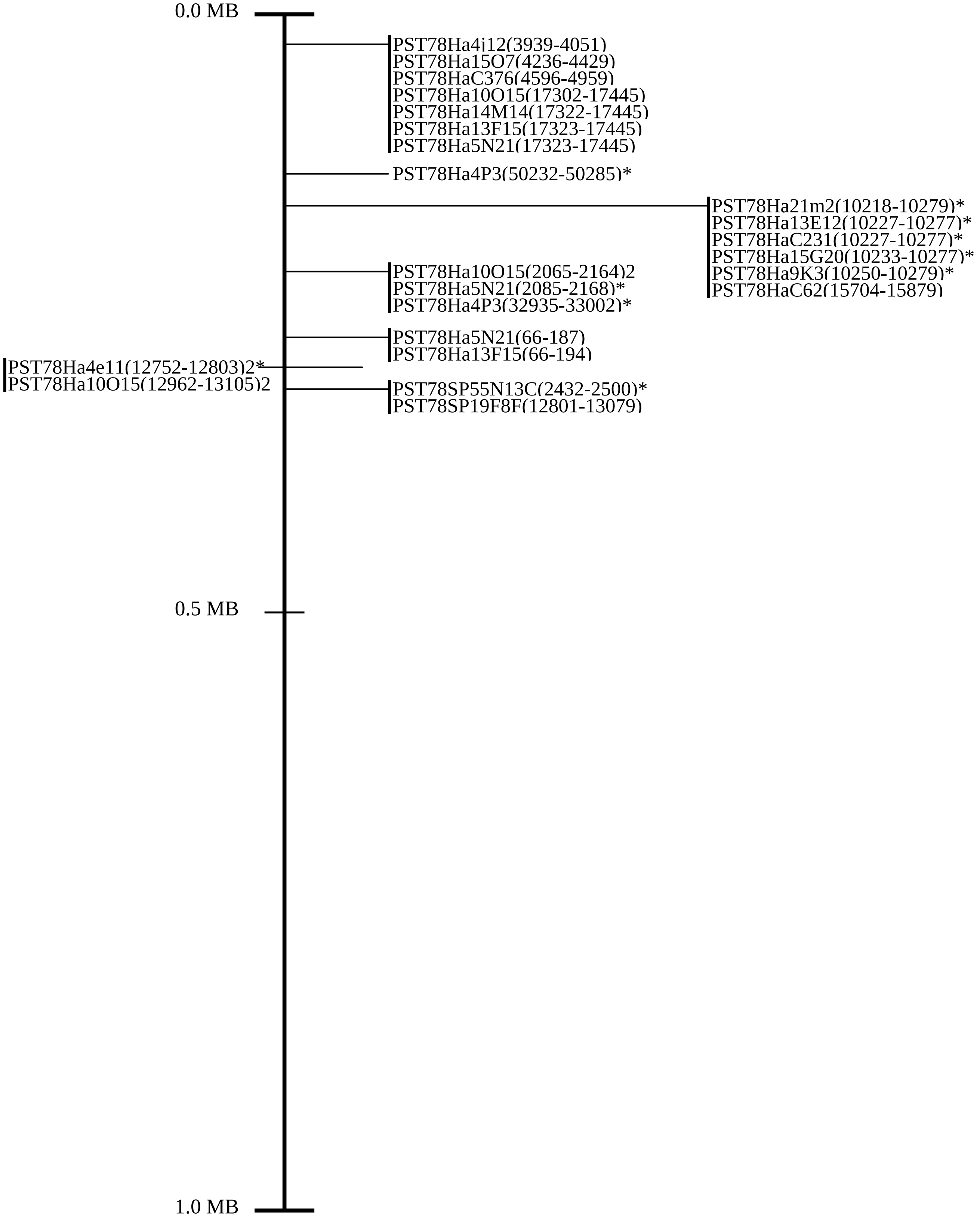

Supercontig 80
length: 312,600 bp

*** = less than 100bp alignment** **| = clones in the same contig**

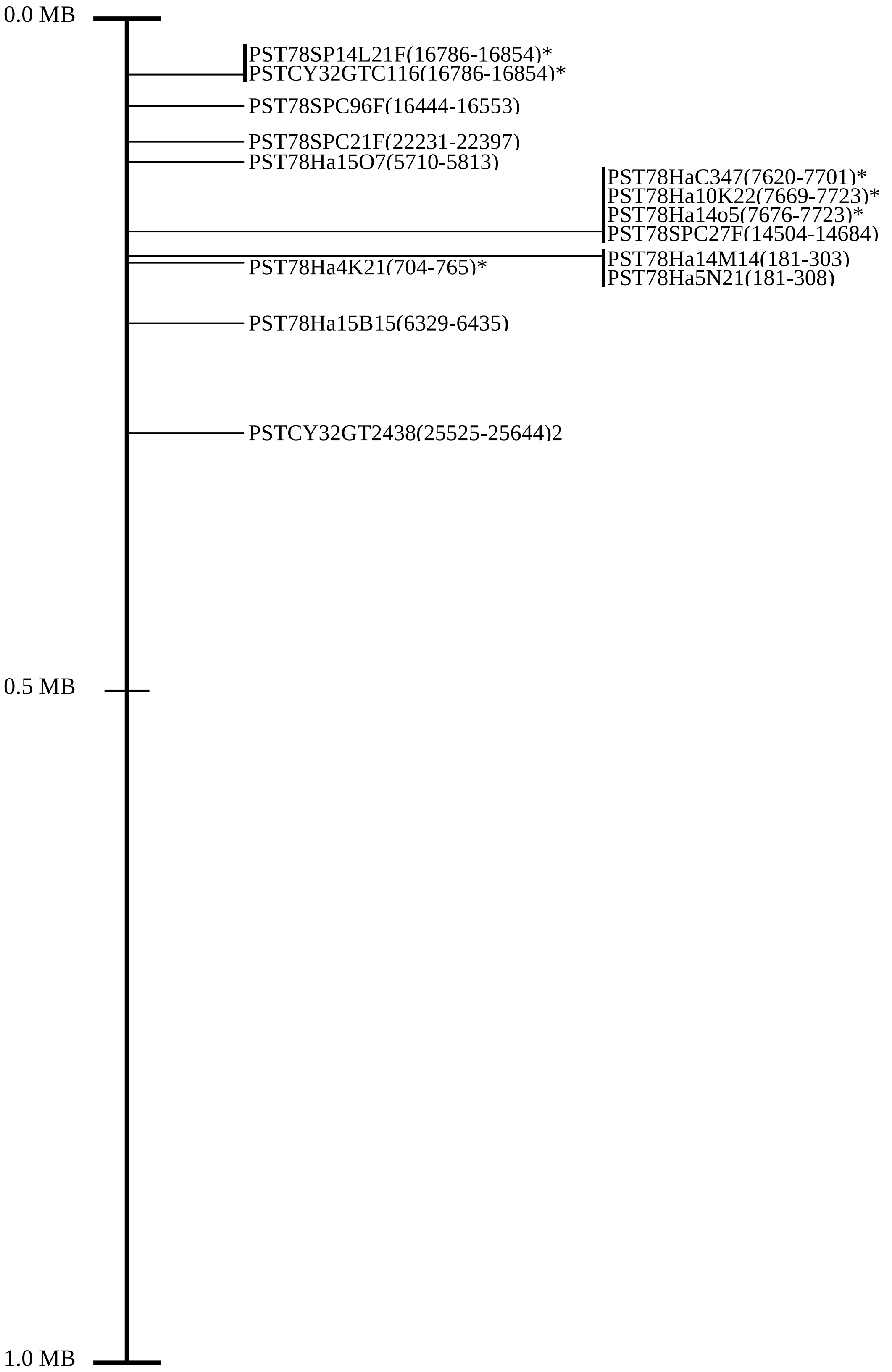

Supercontig 81
length: 341,312 bp

*** = less than 100bp alignment** **| = clones in the same contig**

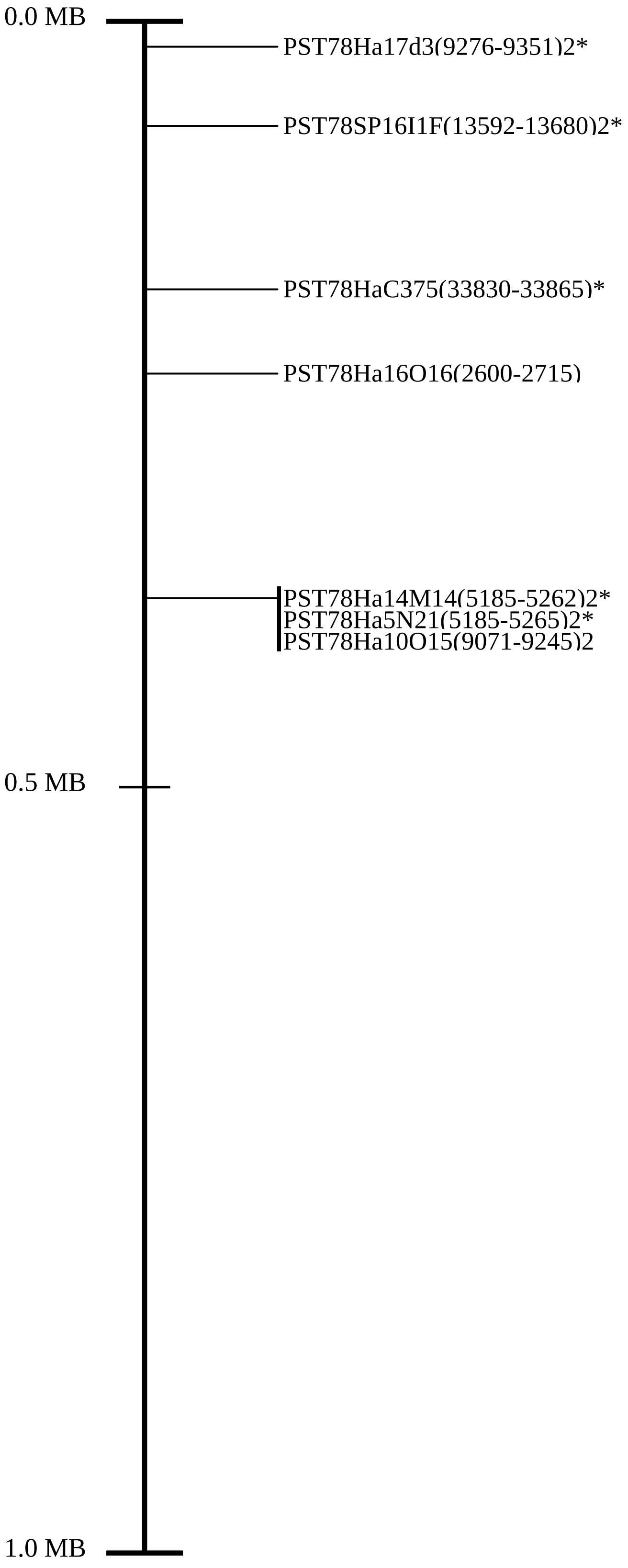

Supercontig 82
length: 375,963 bp

*** = less than 100bp alignment** **| = clones in the same contig**

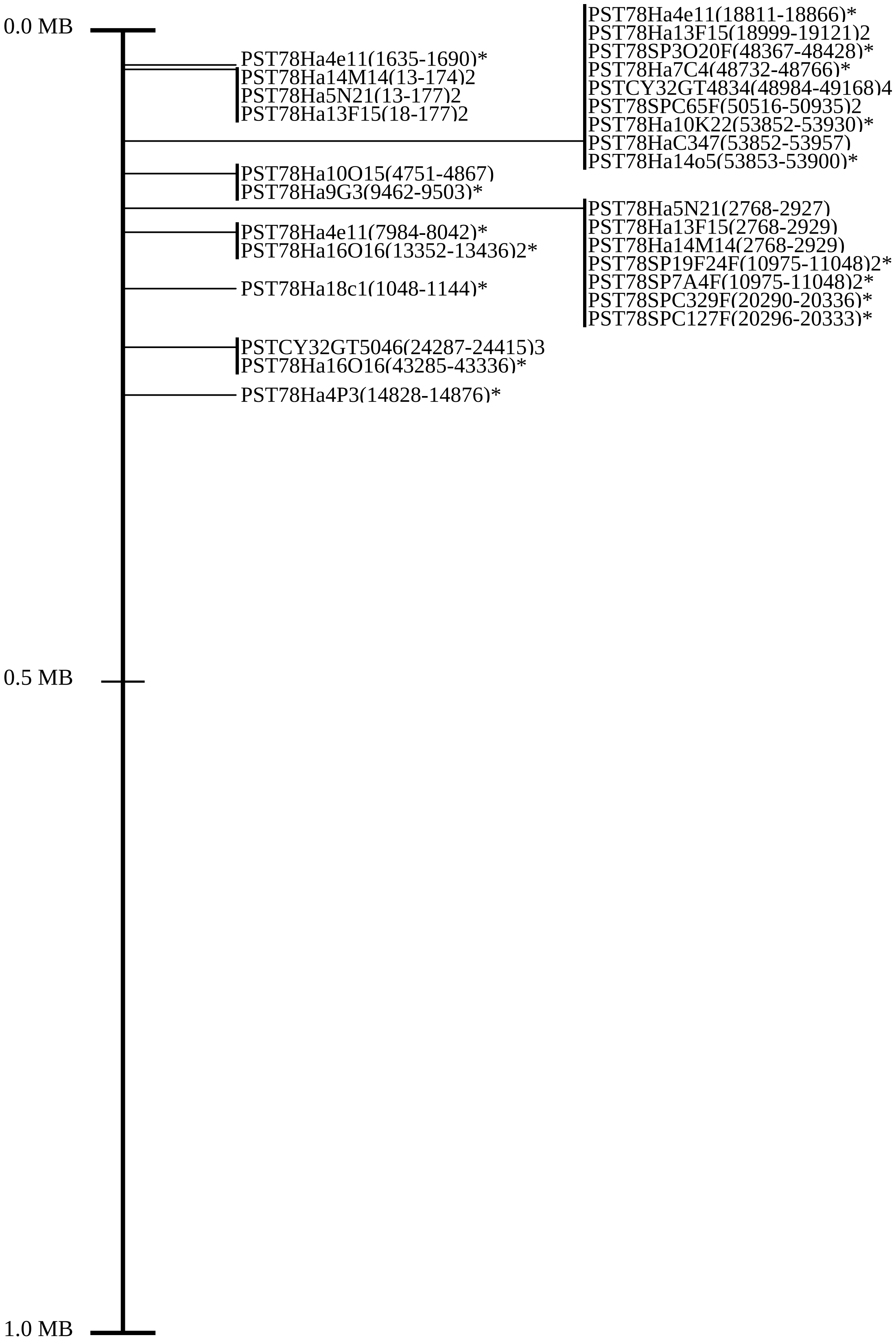

Supercontig 83
length: 301,412 bp

*** = less than 100bp alignment** **| = clones in the same contig**

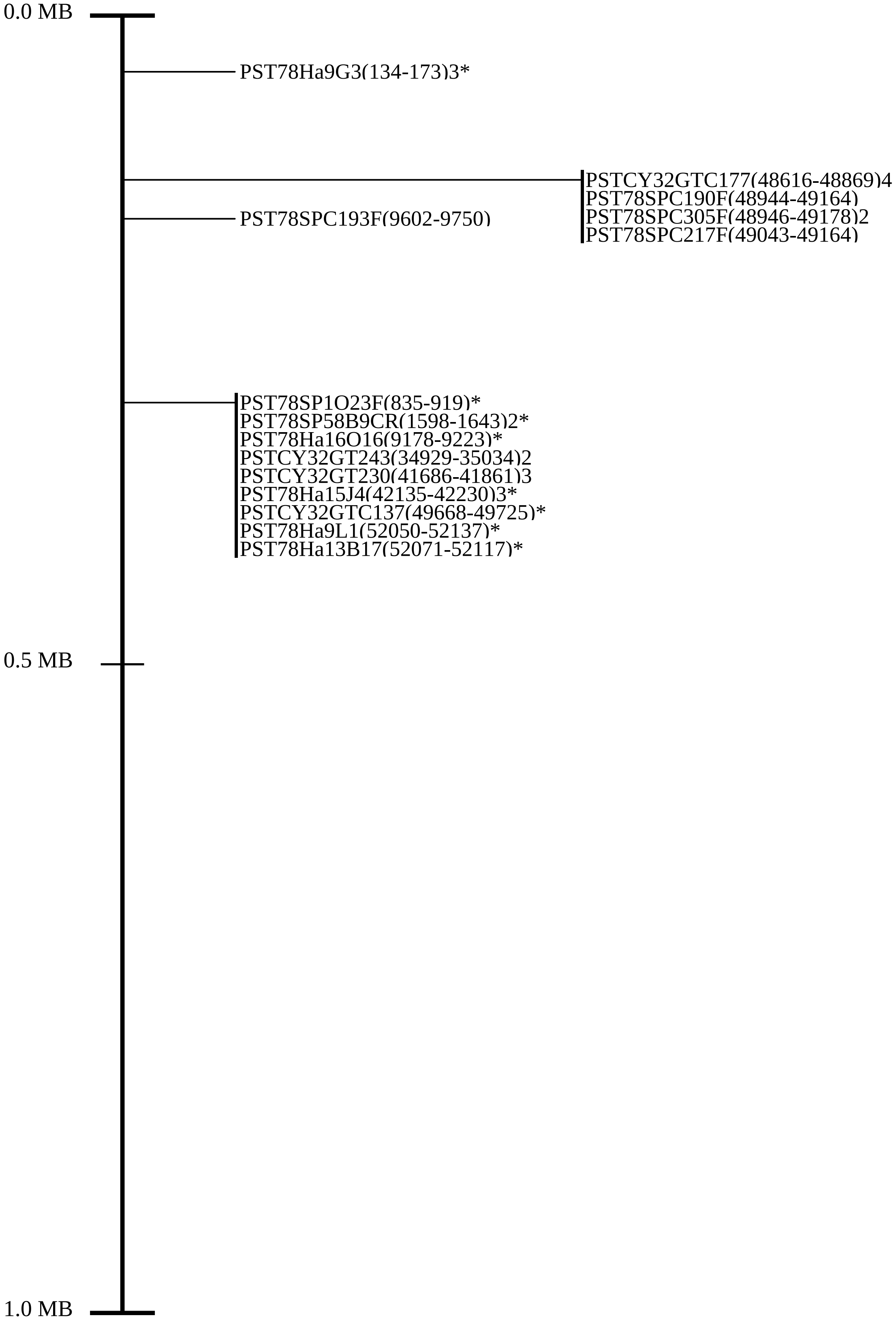

Supercontig 84
length: 306,211 bp

*** = less than 100bp alignment** **| = clones in the same contig**

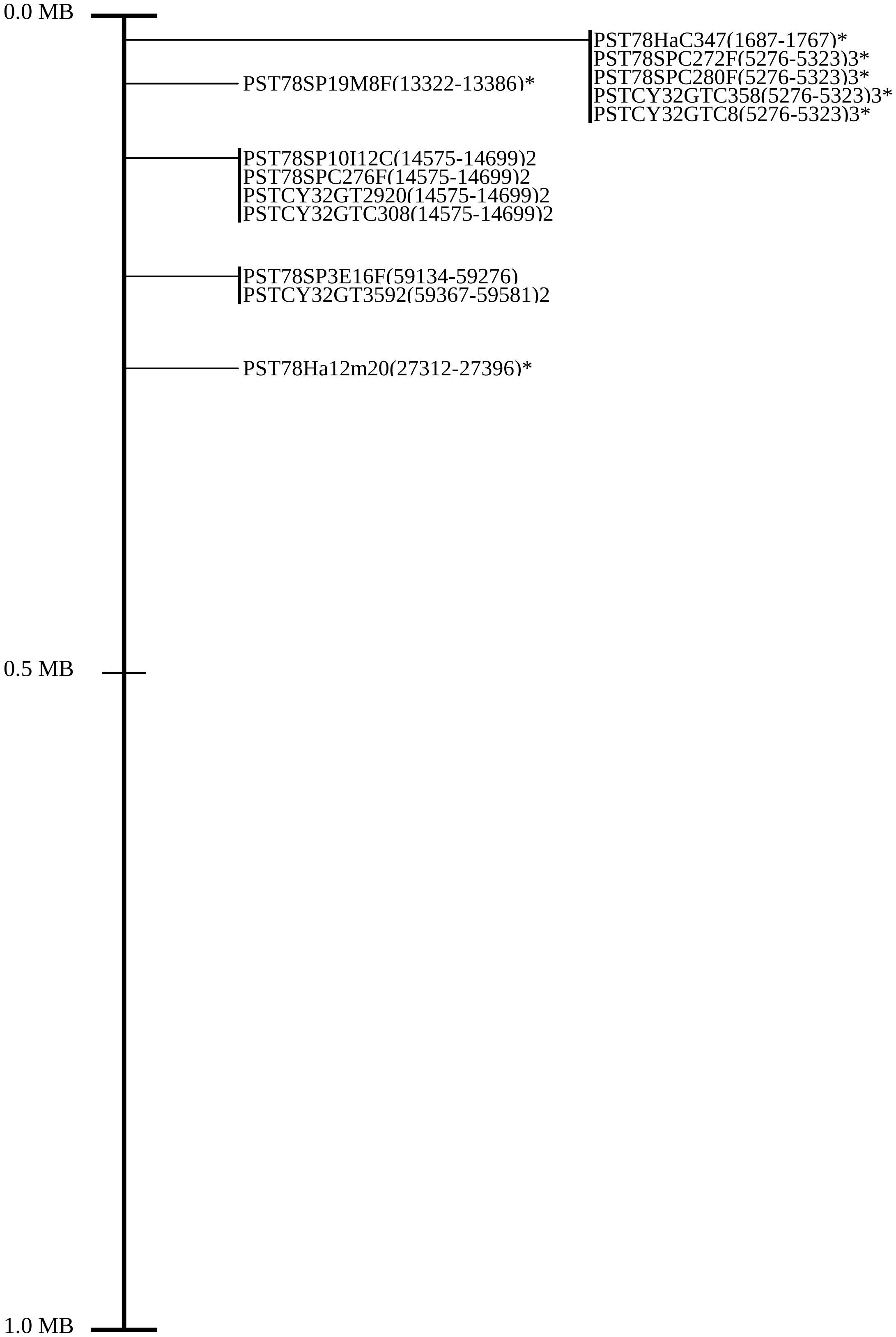

Supercontig 85
length: 286,187 bp

*** = less than 100bp alignment** **| = clones in the same contig**

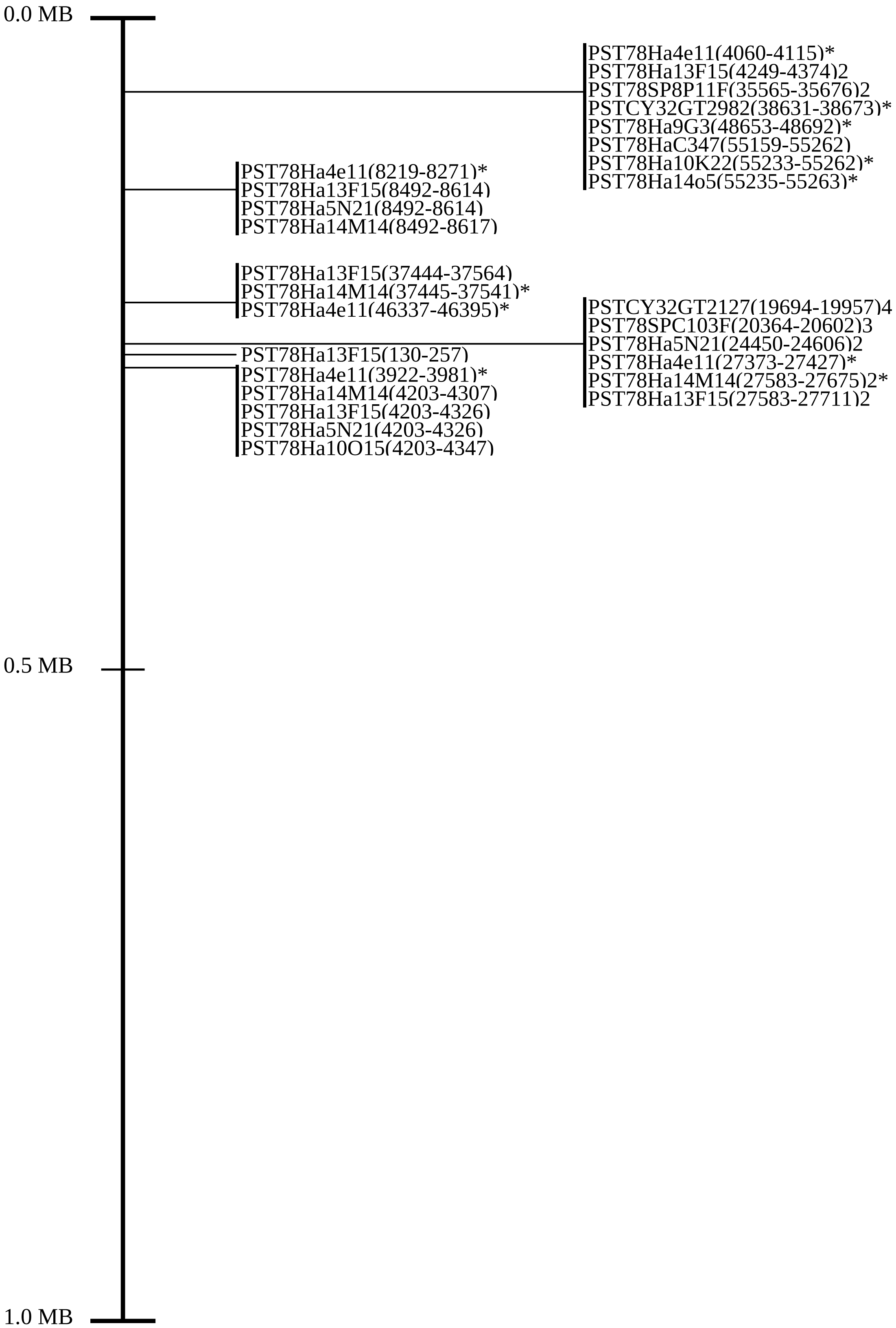

Supercontig 86
length: 268,723 bp

*** = less than 100bp alignment** **| = clones in the same contig**

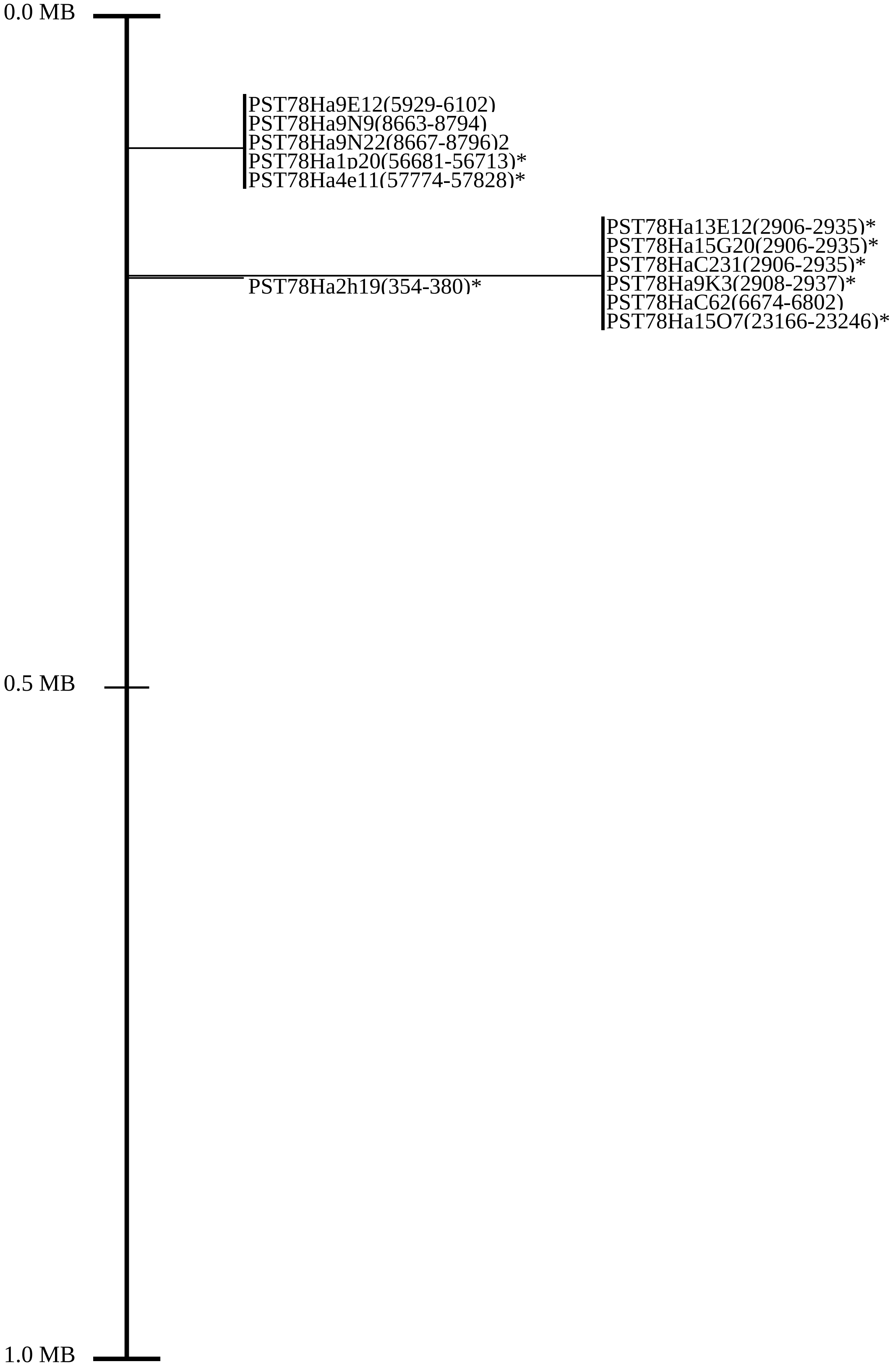

Supercontig 87
length: 273,404 bp

*** = less than 100bp alignment** **| = clones in the same contig**

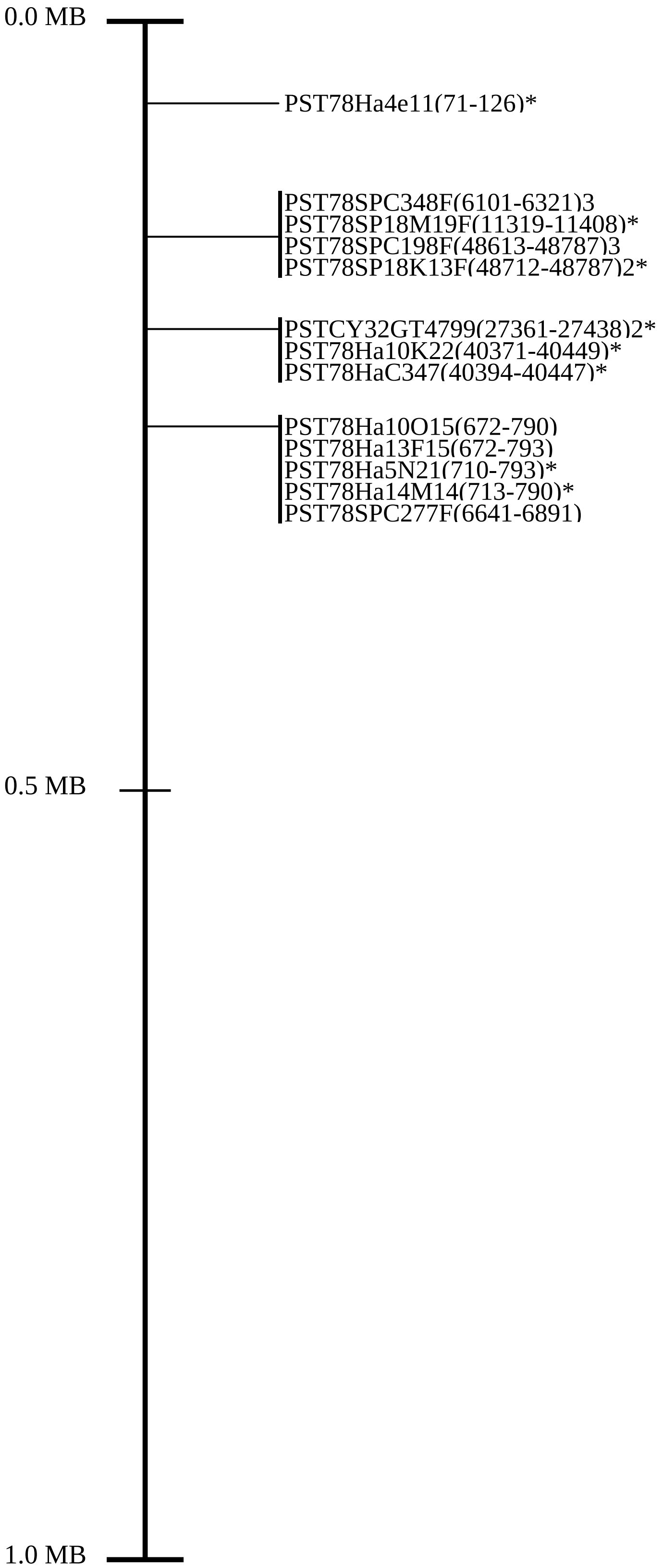

Supercontig 88
length: 271,488 bp

*** = less than 100bp alignment** **| = clones in the same contig**

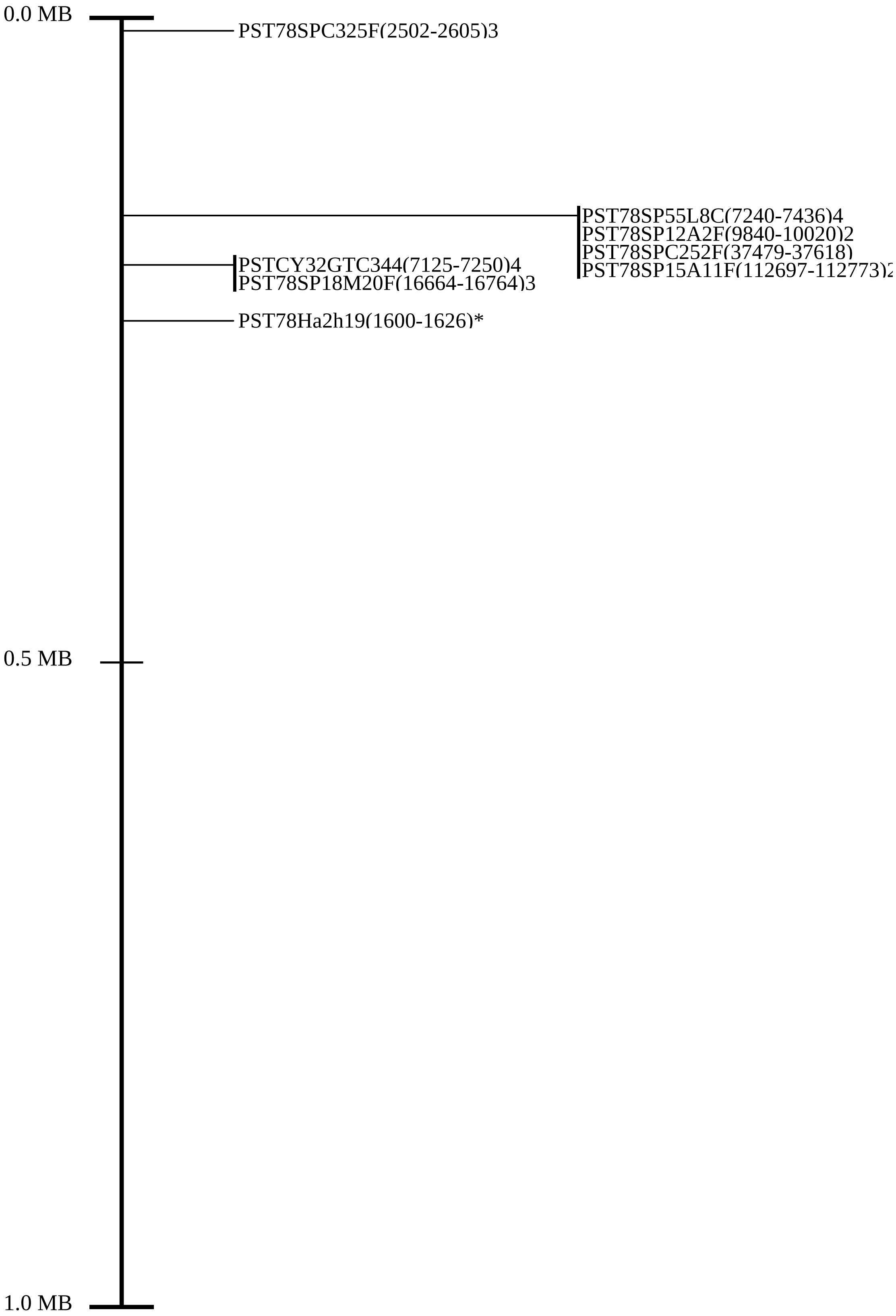

Supercontig 89
length: 281,218 bp

*** = less than 100bp alignment** **| = clones in the same contig**

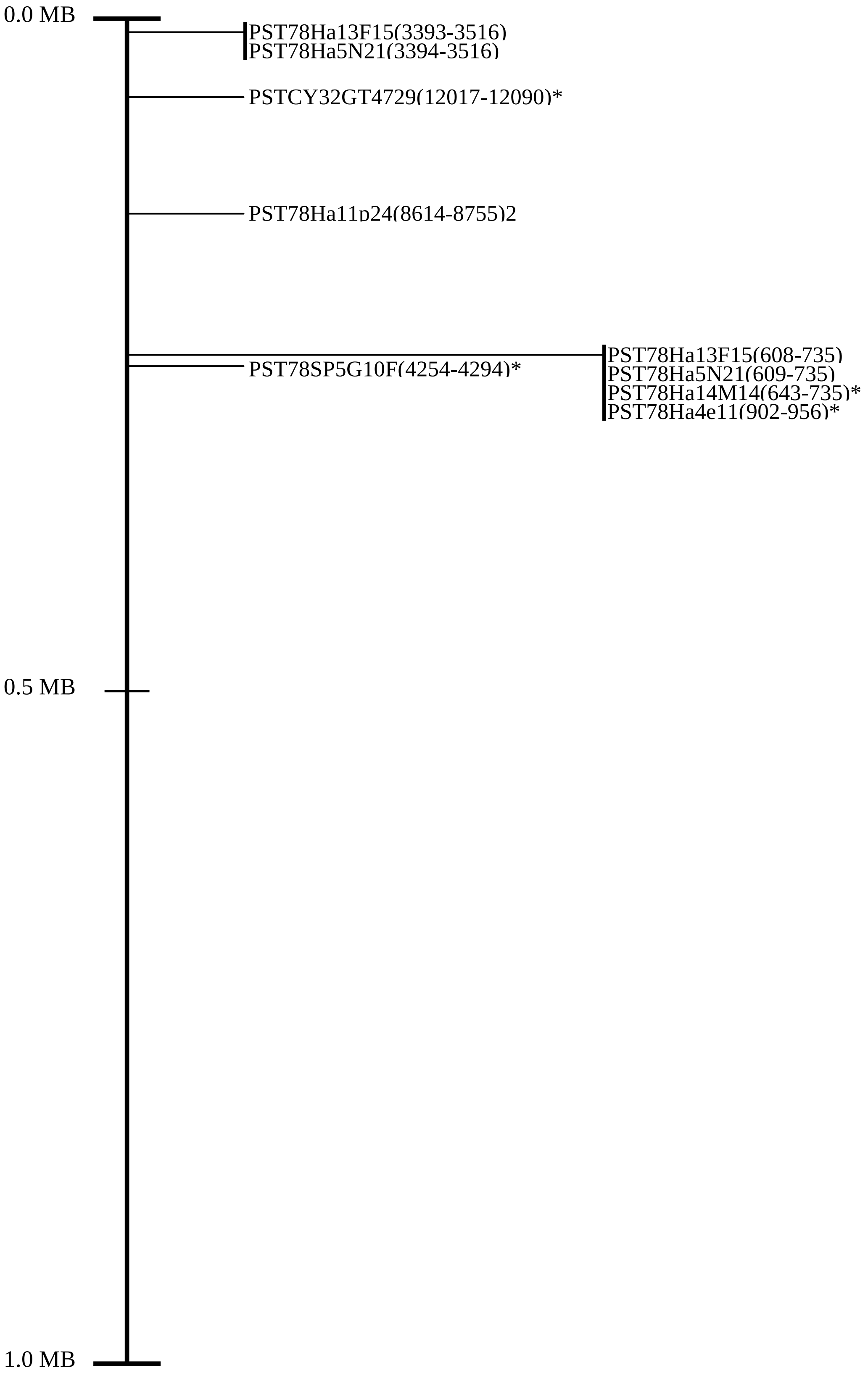

Supercontig 90
length: 282,829 bp

*** = less than 100bp alignment** **| = clones in the same contig**

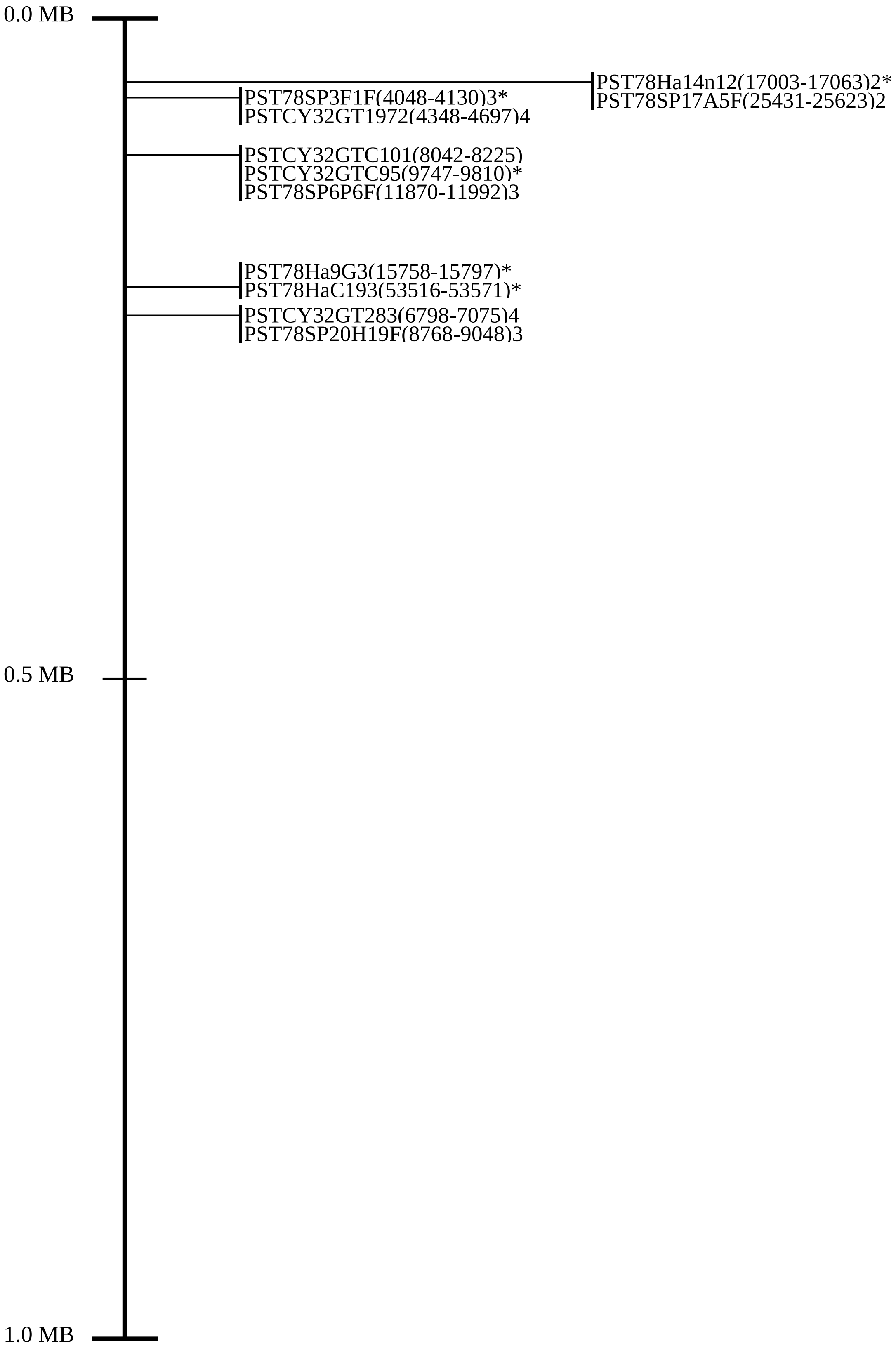

Supercontig 91
length: 268,653 bp

*** = less than 100bp alignment** **| = clones in the same contig**

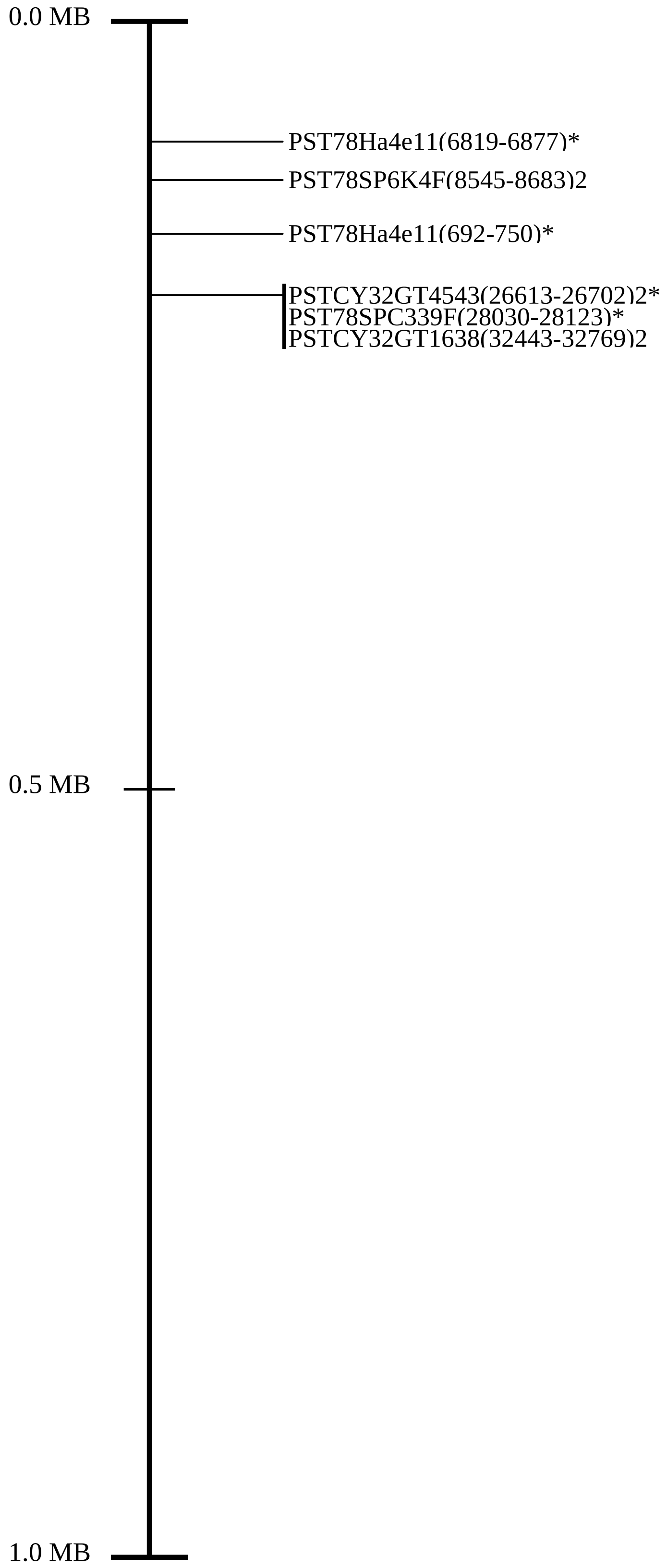

Supercontig 92
length: 249,303 bp

*** = less than 100bp alignment**

| = clones in the same contig

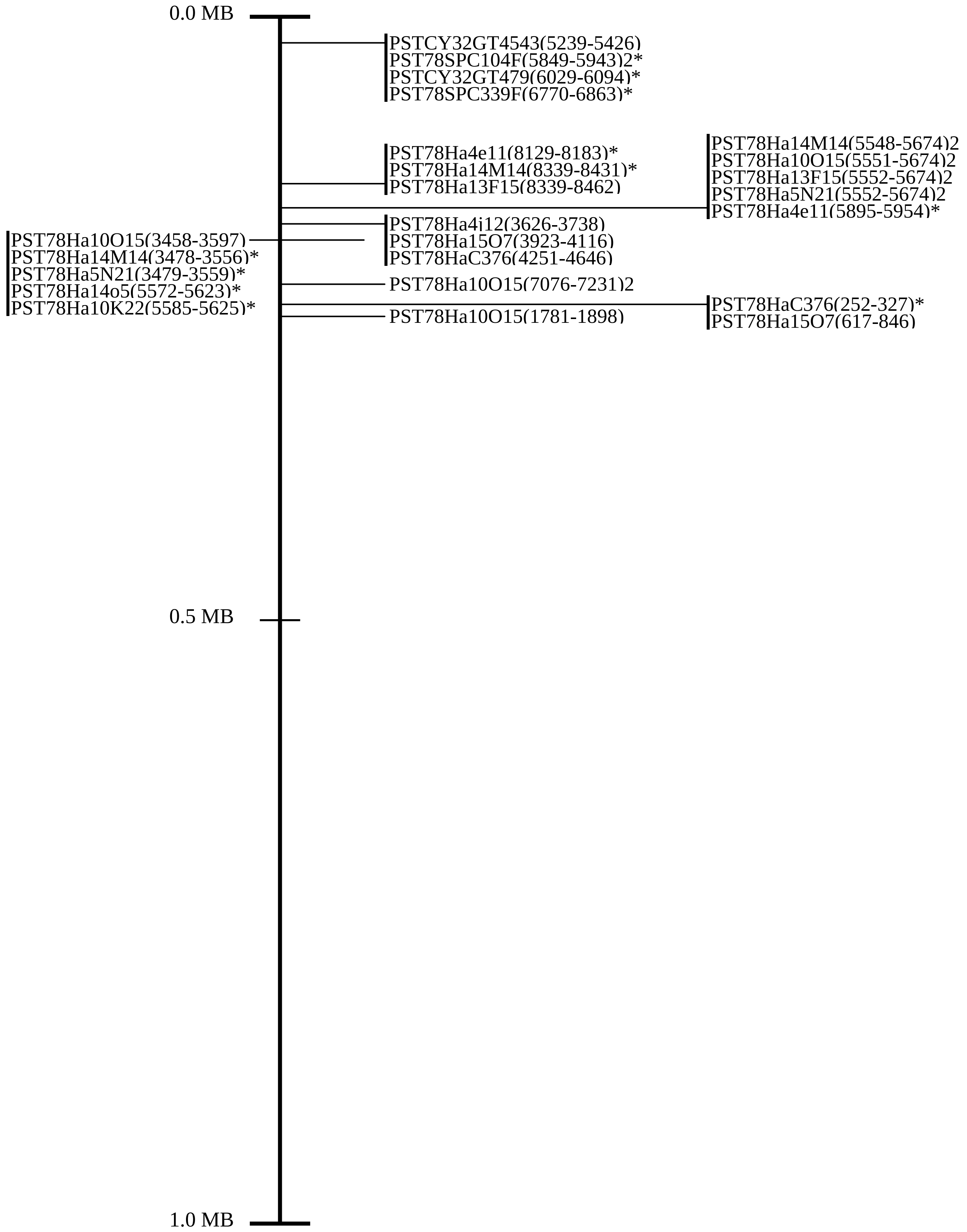

Supercontig 93
length: 254,338 bp

*** = less than 100bp alignment**

| = clones in the same contig

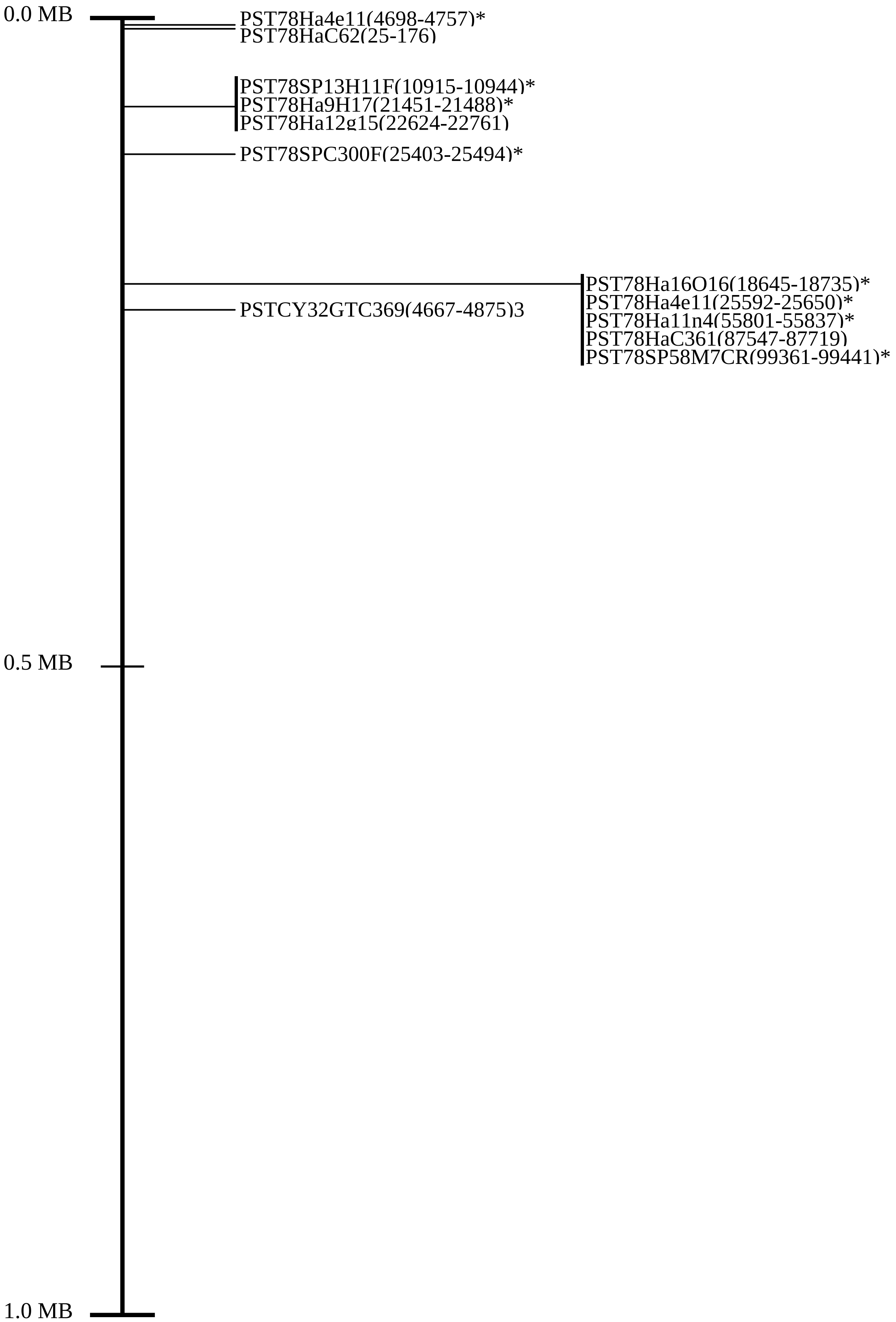

Supercontig 94
length: 224,654 bp

*** = less than 100bp alignment** **| = clones in the same contig**

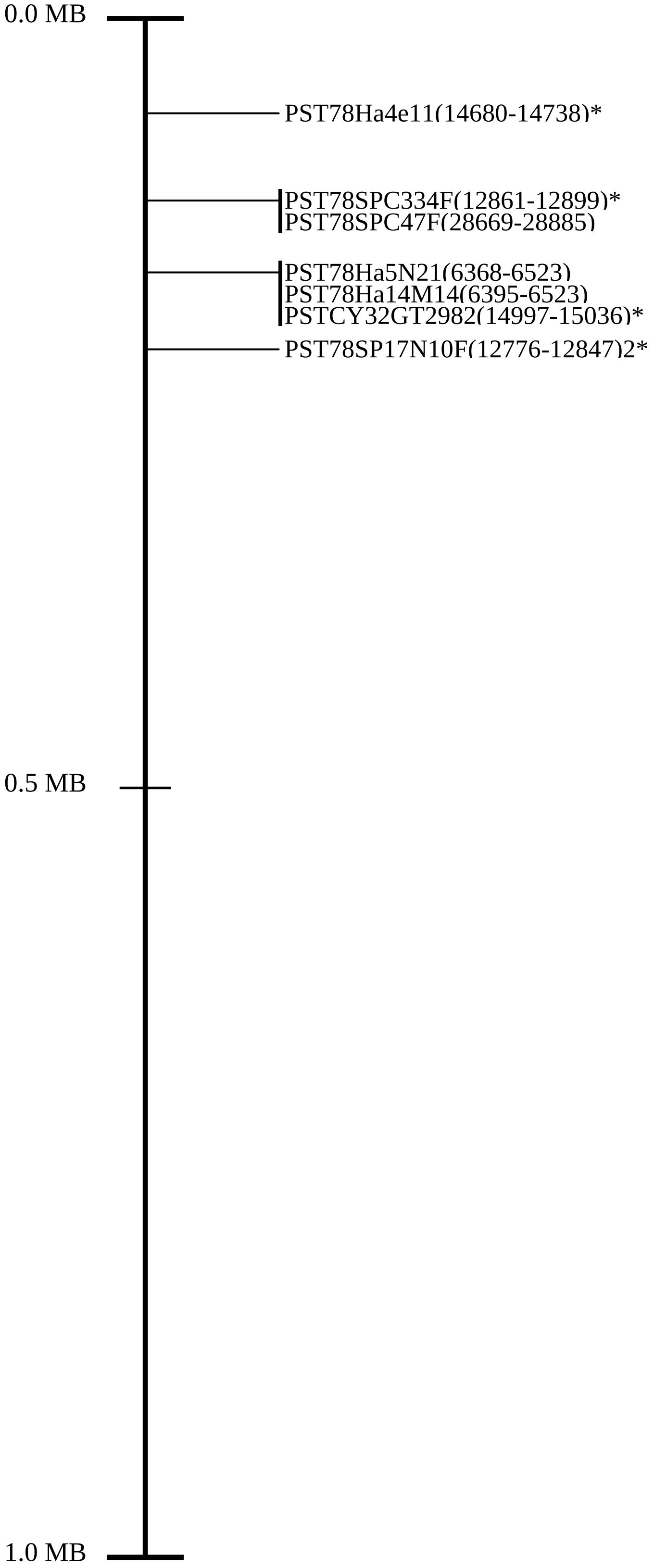

Supercontig 95
length: 268,031 bp

*** = less than 100bp alignment** **| = clones in the same contig**

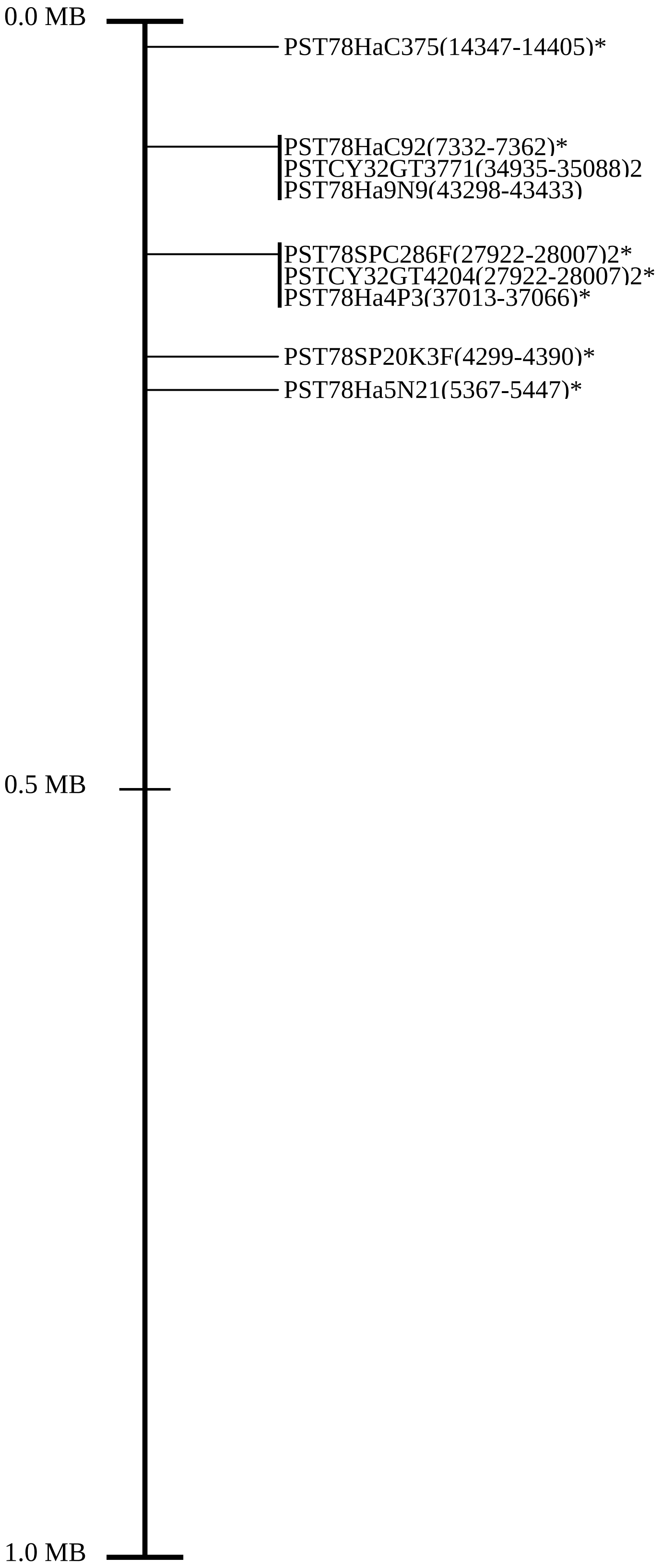

Supercontig 96
length: 240,035 bp

*** = less than 100bp alignment** **| = clones in the same contig**

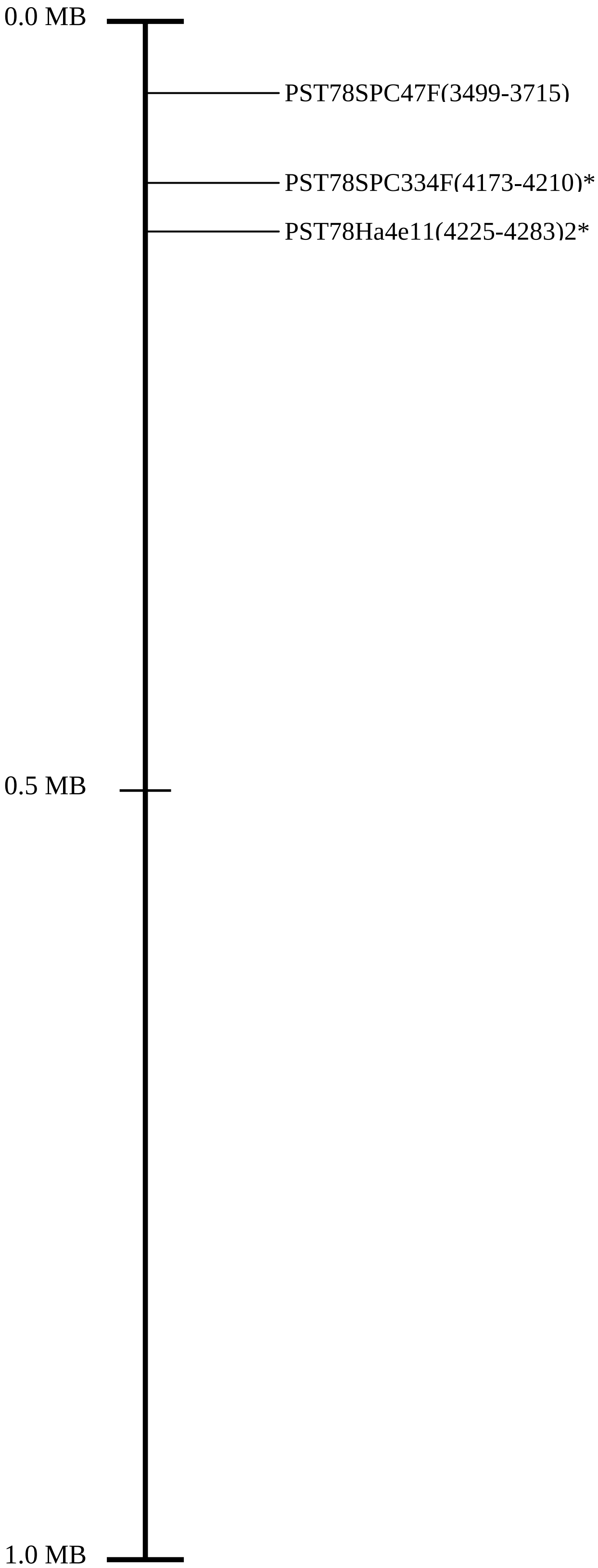

Supercontig 97
length: 283,953 bp

*** = less than 100bp alignment** **| = clones in the same contig**

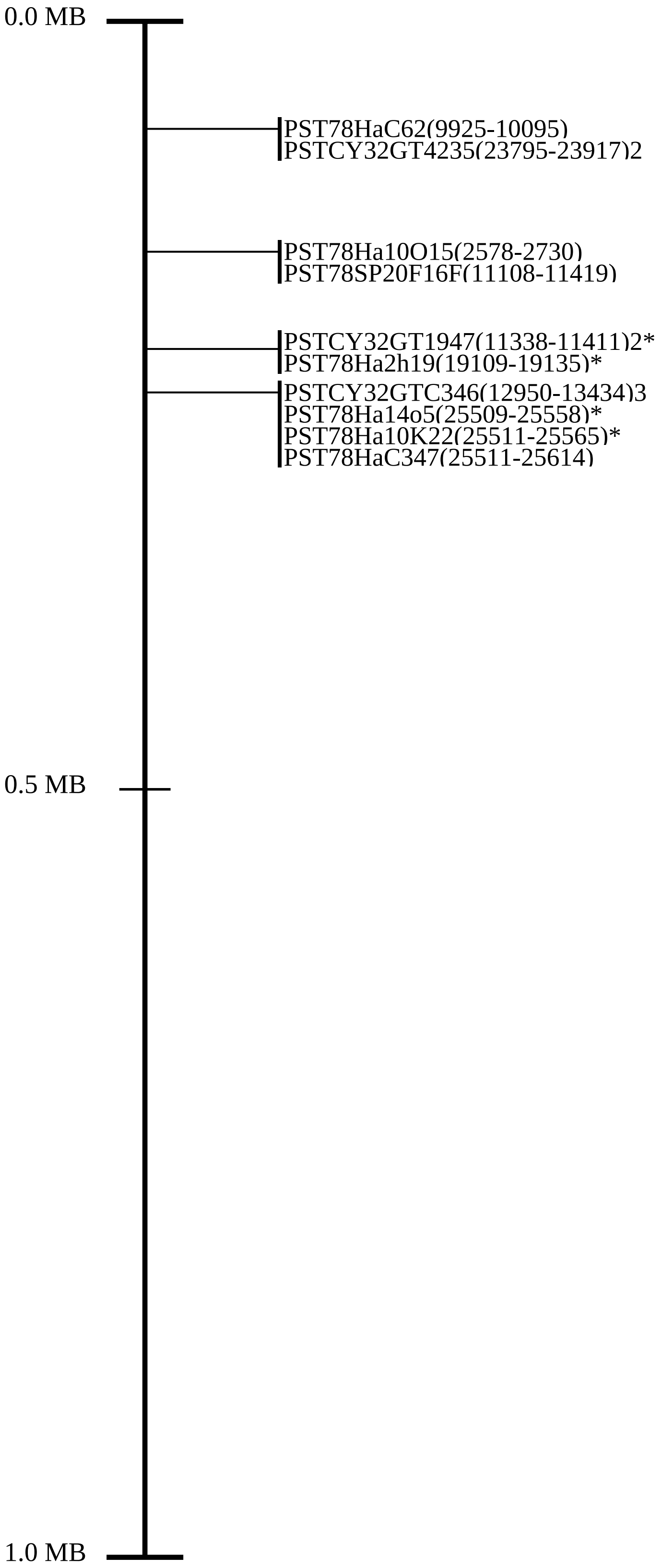

Supercontig 98
length: 259,162 bp

*** = less than 100bp alignment** **| = clones in the same contig**

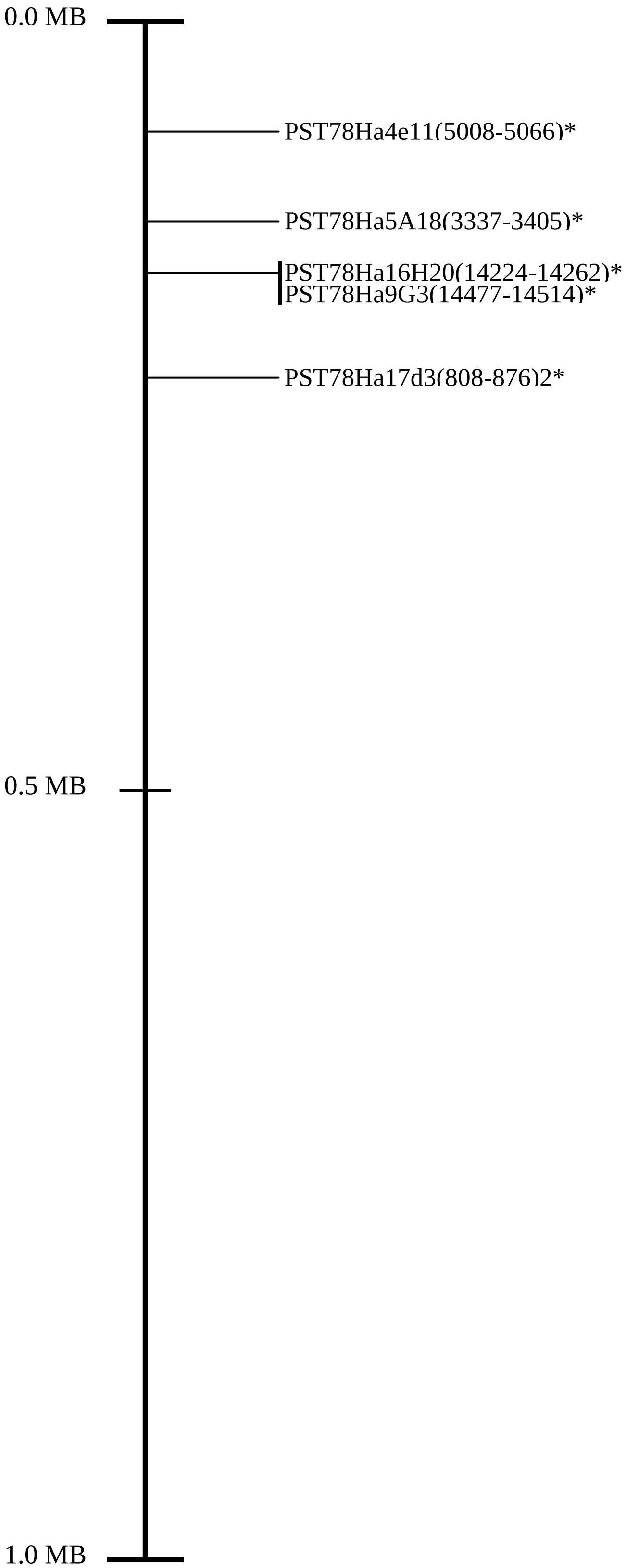

Supercontig 99
length: 245,804 bp

*** = less than 100bp alignment** **| = clones in the same contig**

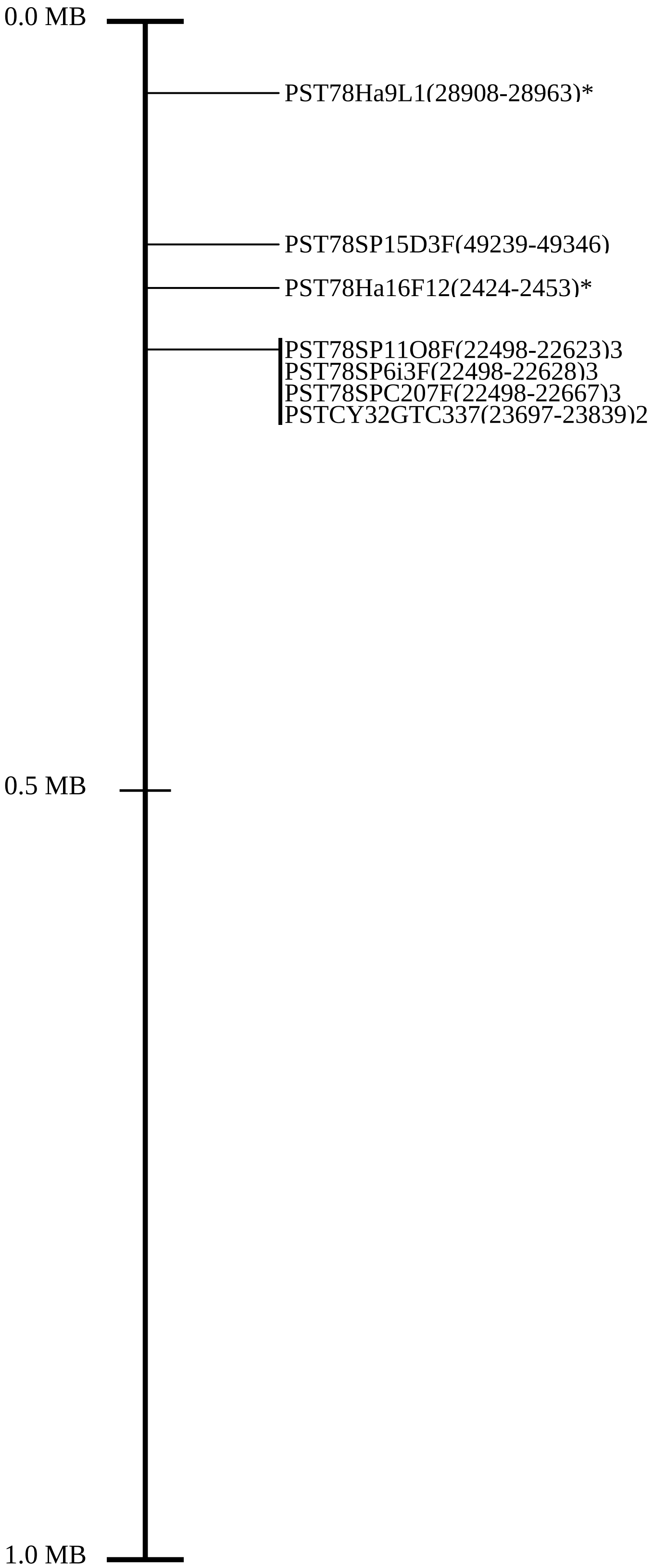

Supercontig 100
length: 212,873 bp

*** = less than 100bp alignment** **| = clones in the same contig**

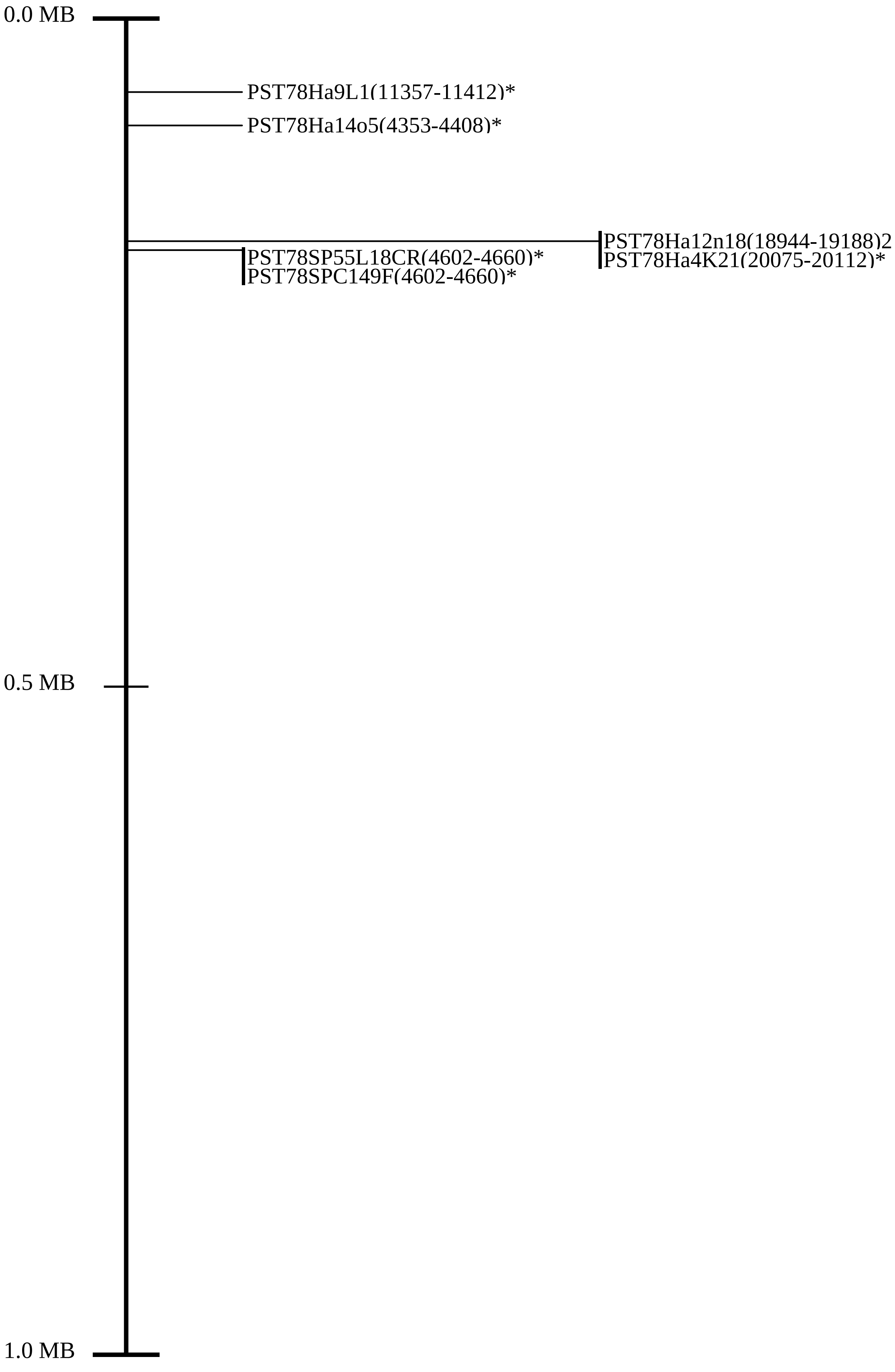

Supercontig 101
length: 249,761 bp

*** = less than 100bp alignment** **| = clones in the same contig**

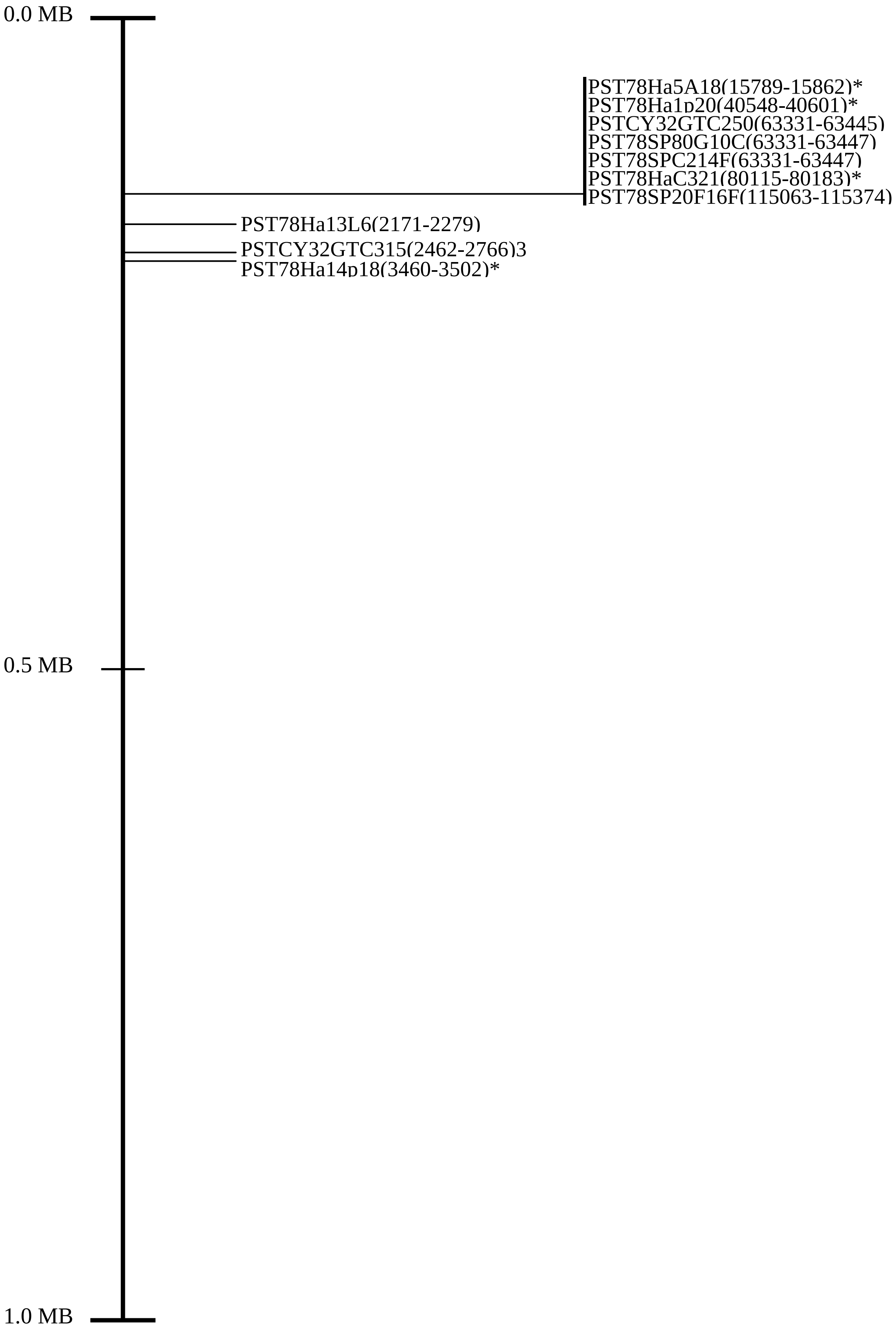

Supercontig 102
length: 224,856 bp

*** = less than 100bp alignment** **| = clones in the same contig**

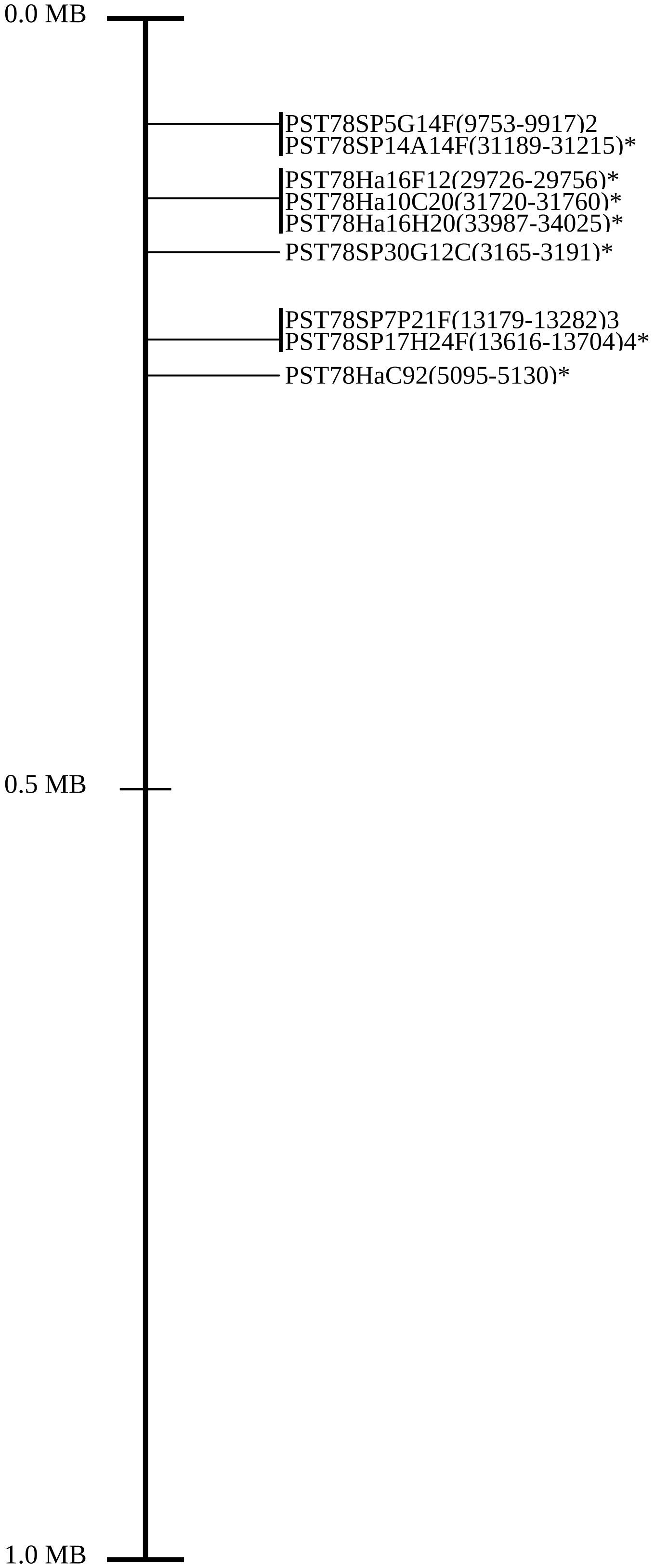

Supercontig 103
length: 231,854 bp

*** = less than 100bp alignment** **| = clones in the same contig**

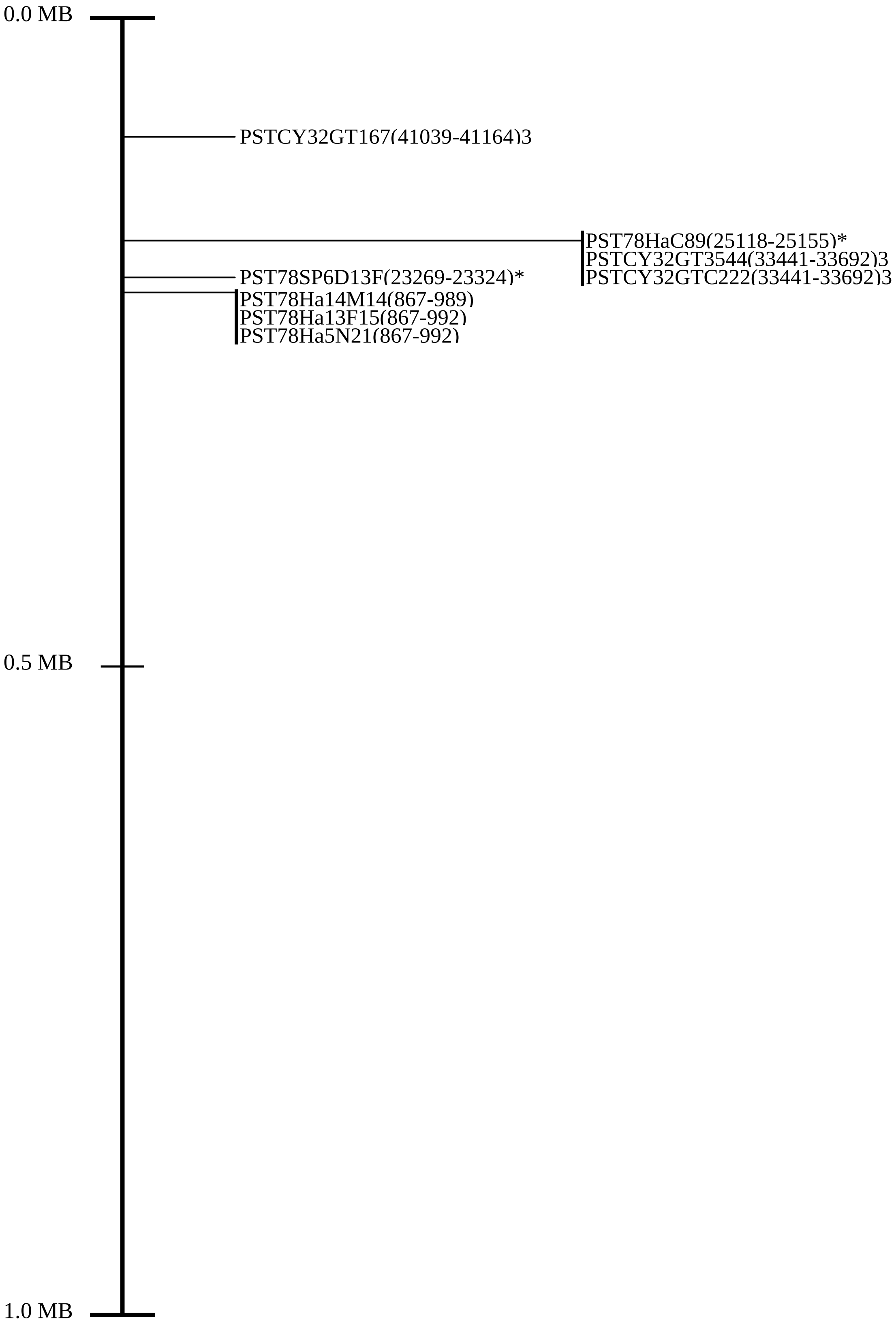

Supercontig 104
length: 230,218 bp

*** = less than 100bp alignment** **| = clones in the same contig**

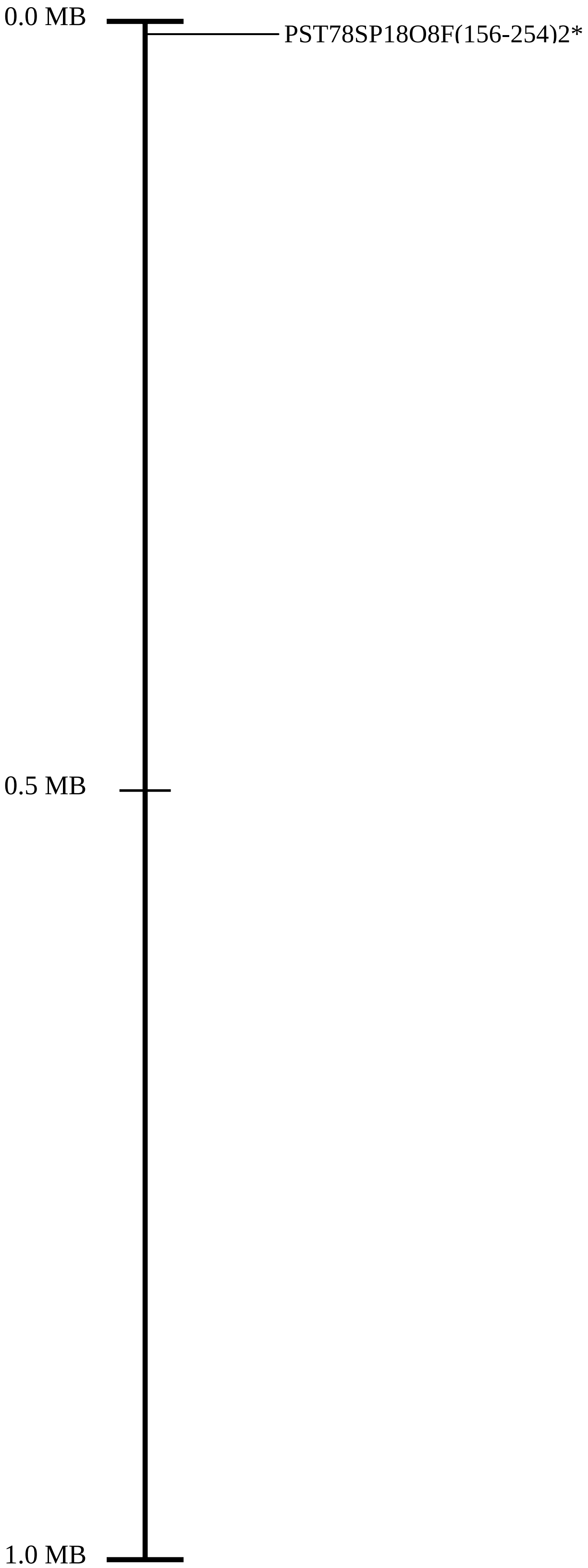

Supercontig 105
length: 209,493 bp

*** = less than 100bp alignment**

| = clones in the same contig

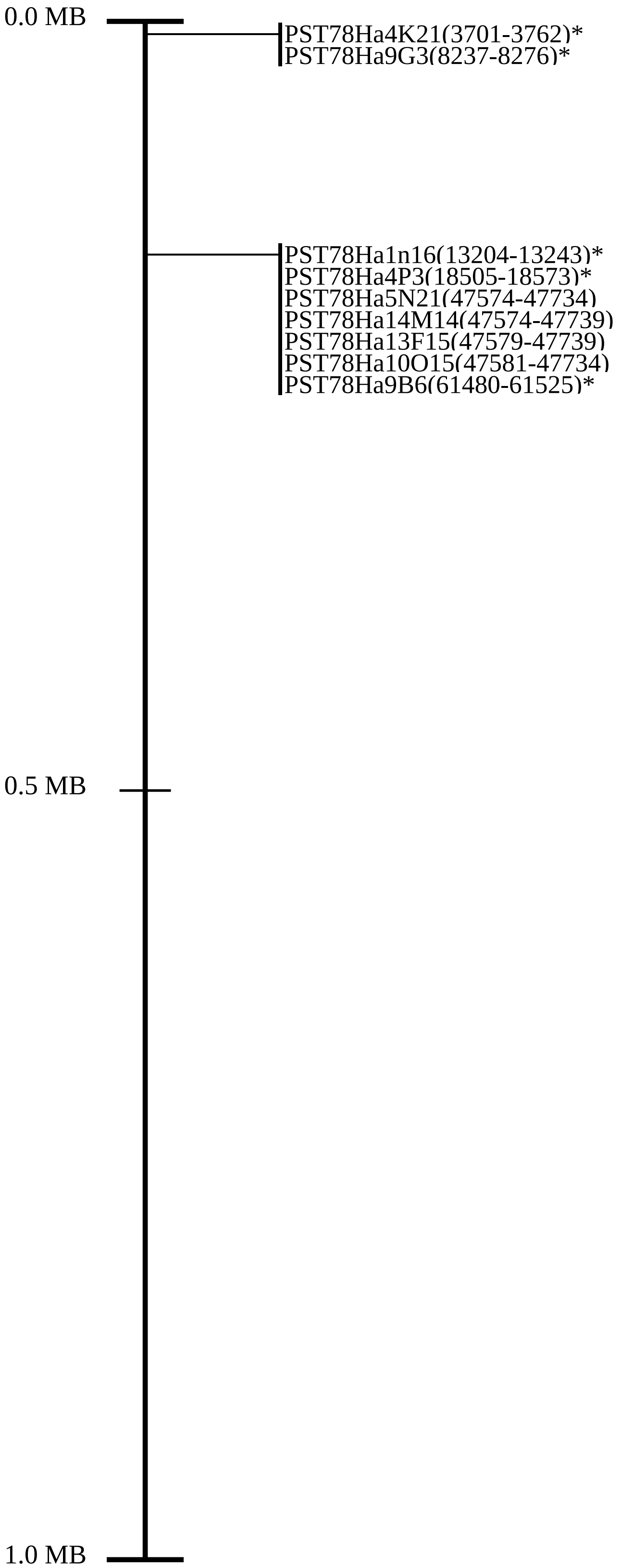

Supercontig 106
length: 179,026 bp

*** = less than 100bp alignment** **| = clones in the same contig**

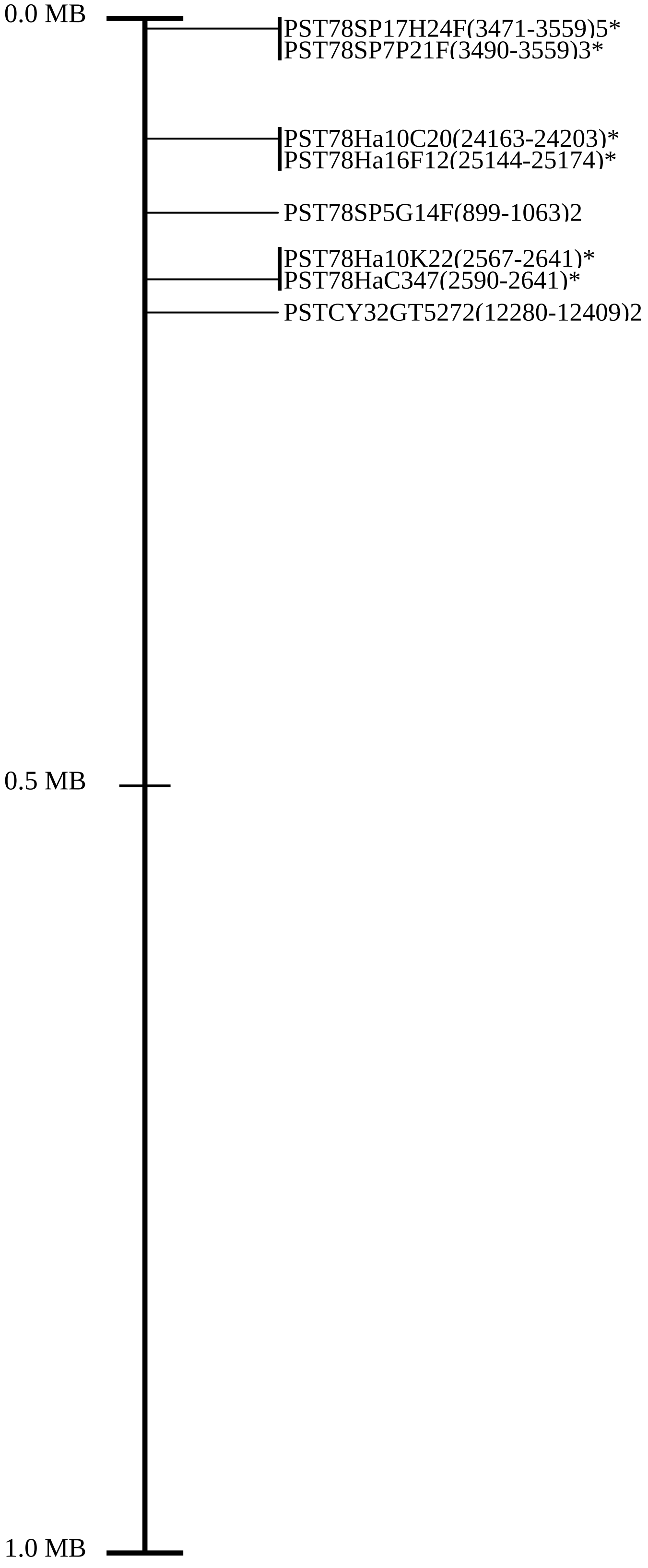

Supercontig 107
length: 191,896 bp

*** = less than 100bp alignment** **| = clones in the same contig**

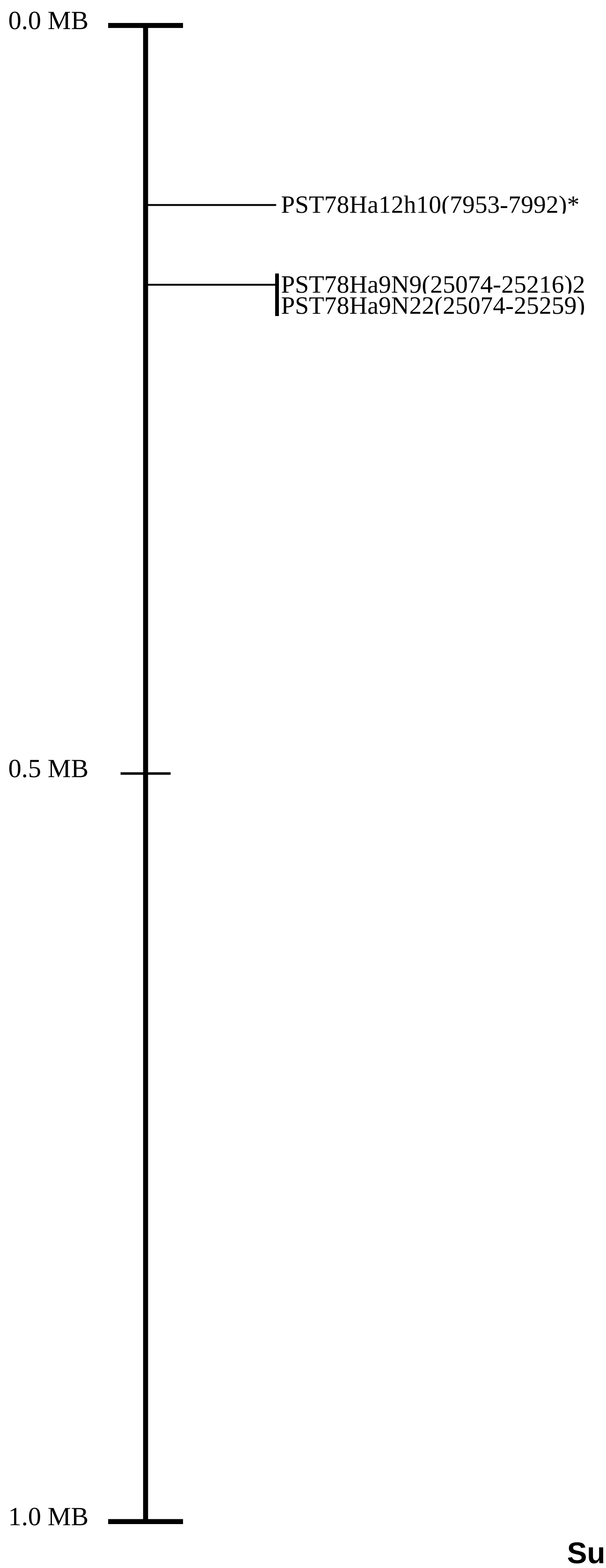

Supercontig 108
length: 172,930 bp

*** = less than 100bp alignment**

| = clones in the same contig

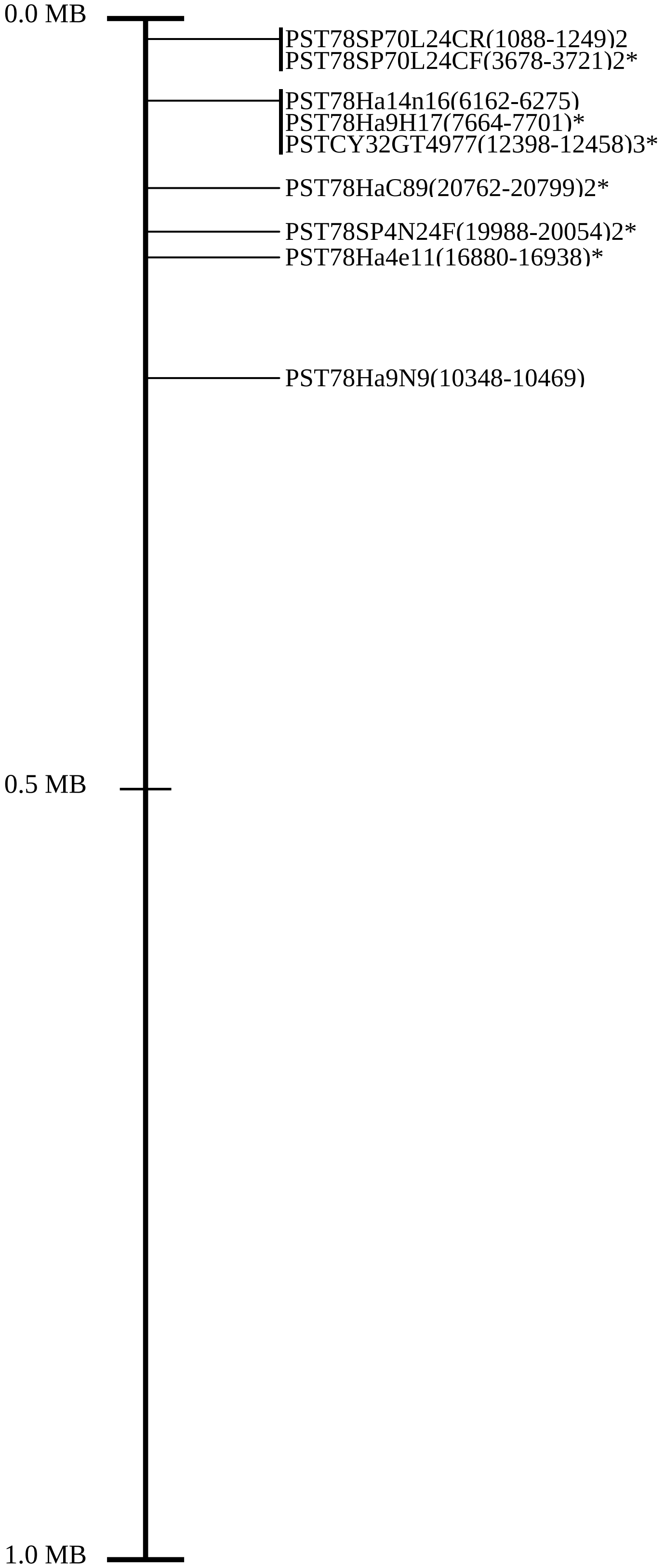

Supercontig 109
length: 232,811 bp

*** = less than 100bp alignment** **| = clones in the same contig**

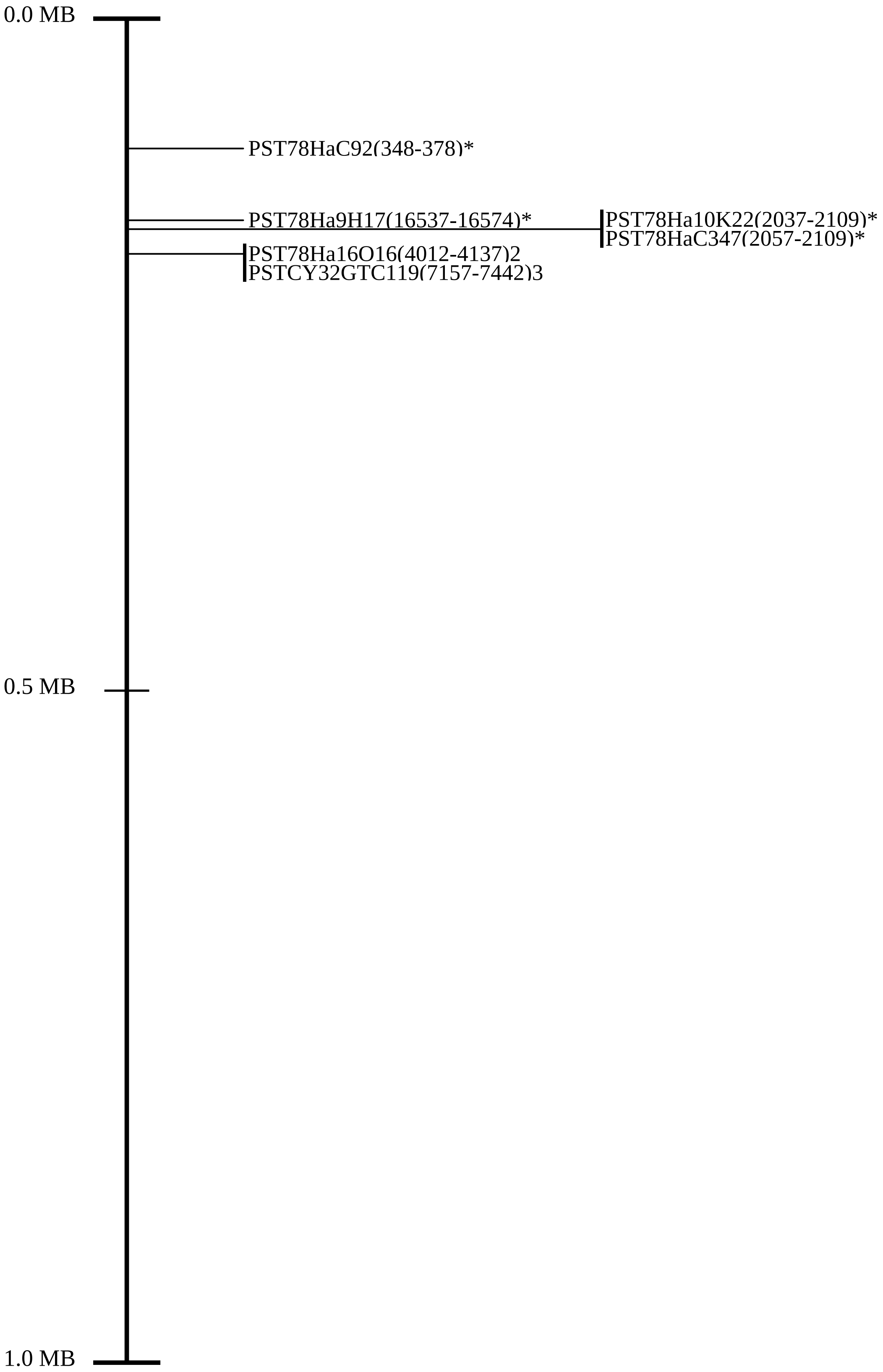

Supercontig 110
length: 174,613 bp

*** = less than 100bp alignment** **| = clones in the same contig**

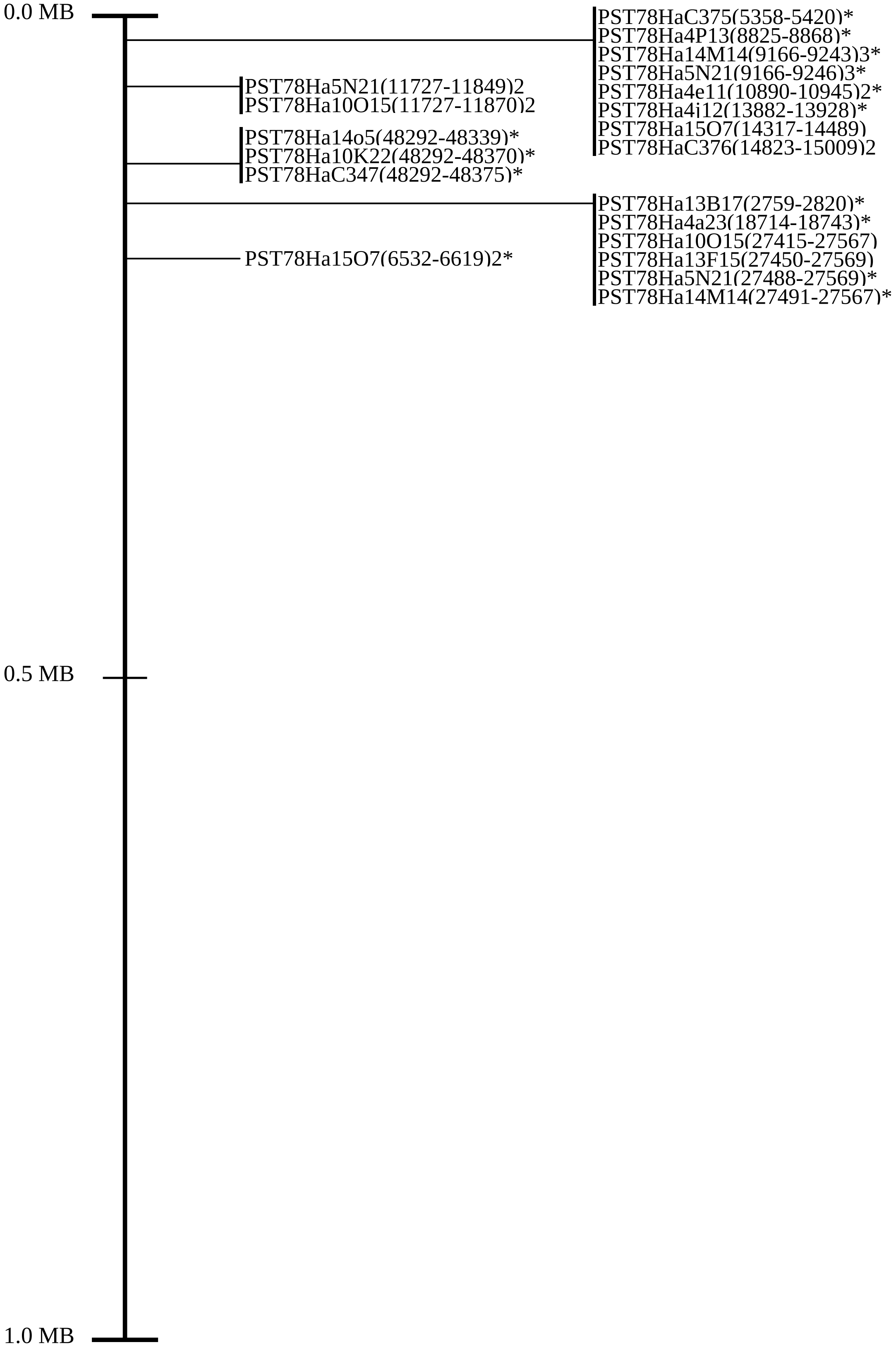

Supercontig 111
length: 183,422 bp

*** = less than 100bp alignment** **| = clones in the same contig**

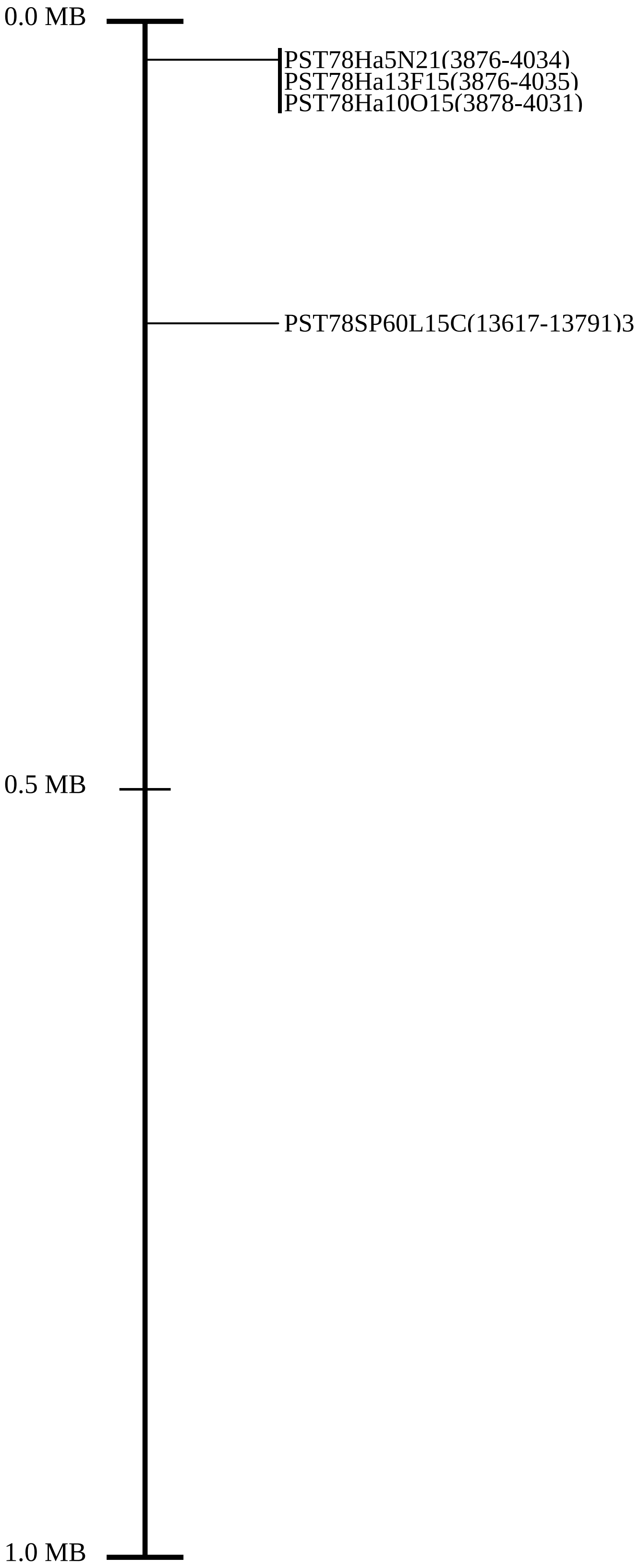

Supercontig 112
length: 205,437 bp

*** = less than 100bp alignment** **| = clones in the same contig**

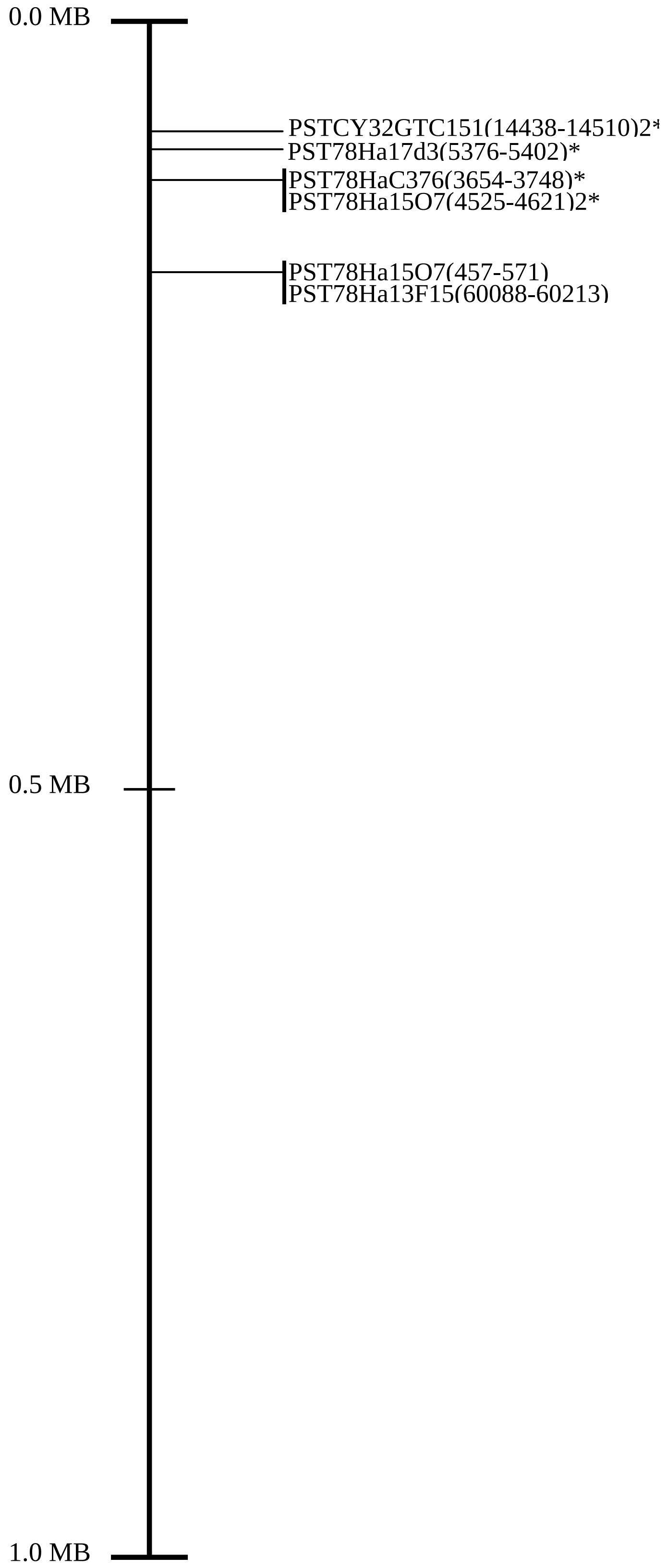

Supercontig 113
length: 163,692 bp

*** = less than 100bp alignment** **| = clones in the same contig**

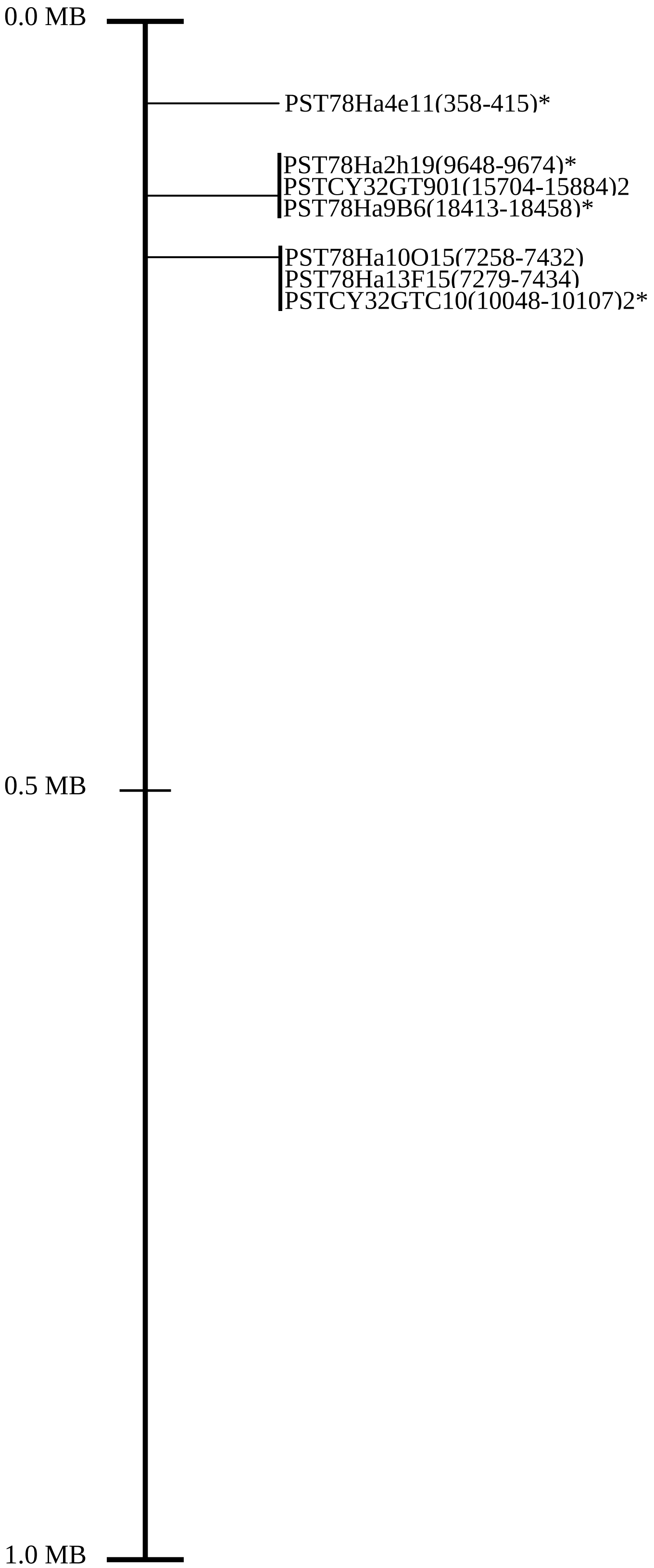

Supercontig 114
length: 173,085 bp

*** = less than 100bp alignment** **| = clones in the same contig**

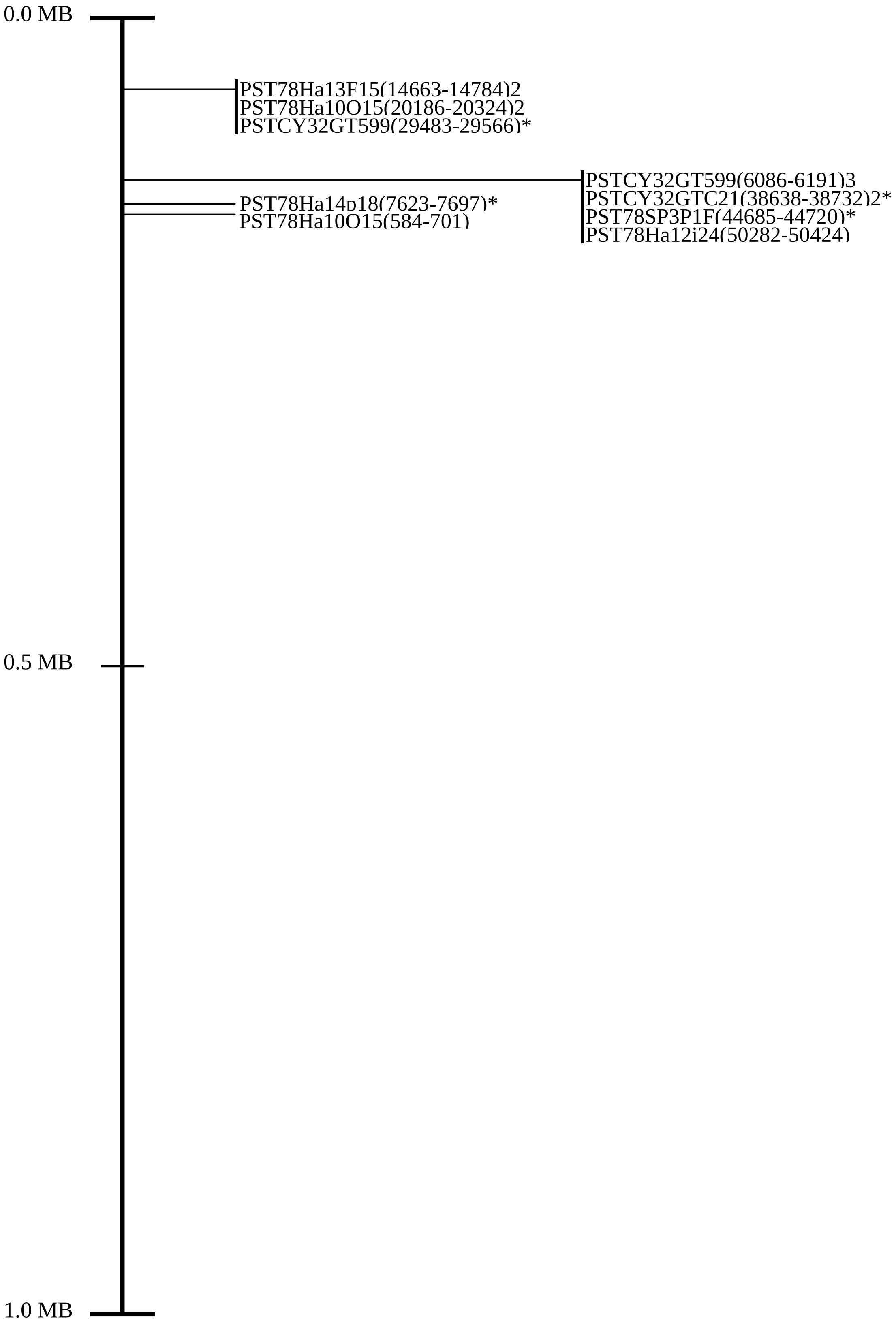

Supercontig 115
length: 158,618 bp

*** = less than 100bp alignment** **| = clones in the same contig**

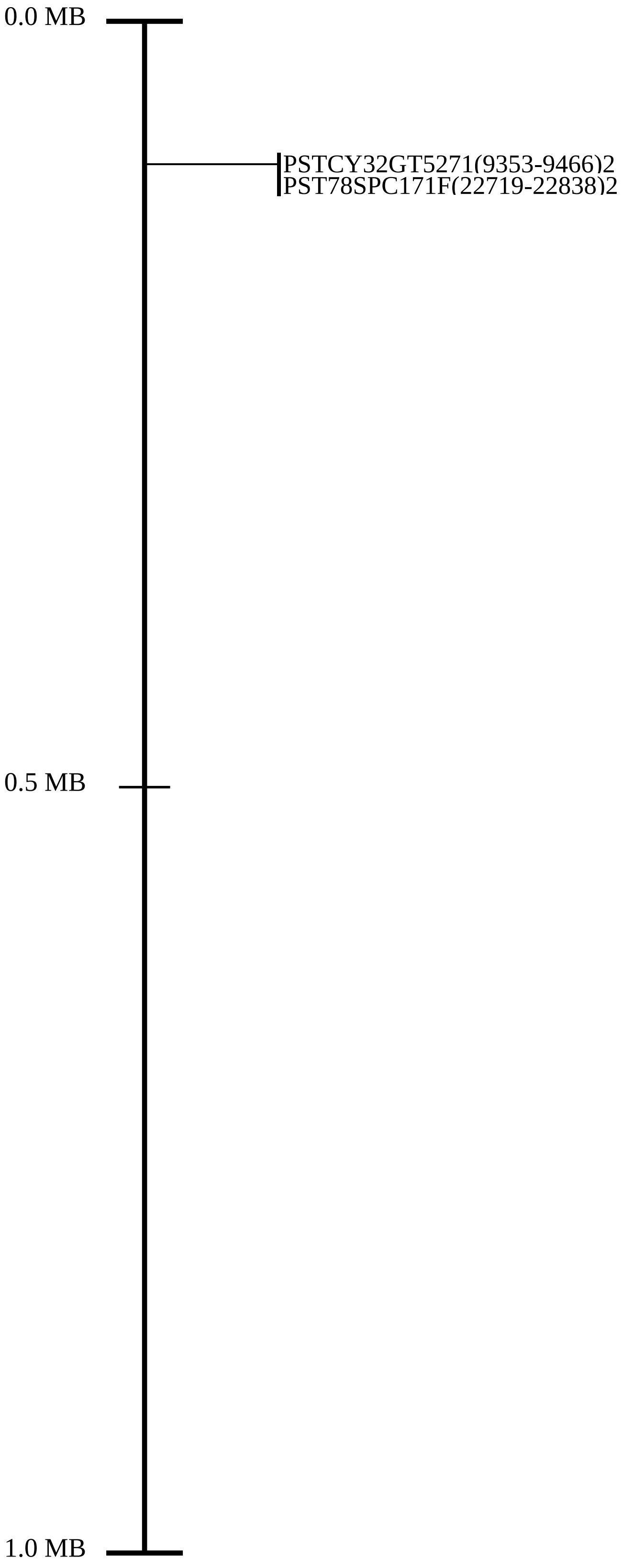

Supercontig 116
length: 160,911 bp

*** = less than 100bp alignment**

| = clones in the same contig

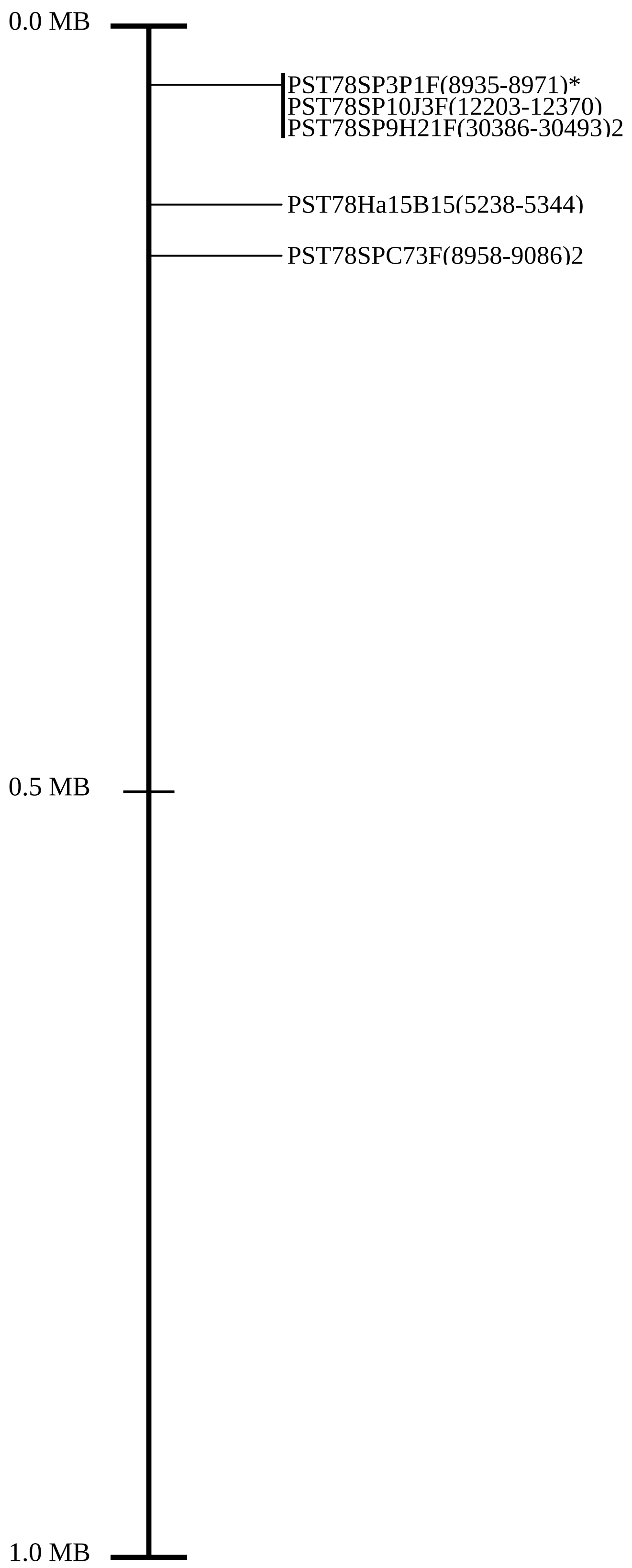

Supercontig 117
length: 159,147 bp

*** = less than 100bp alignment**

| = clones in the same contig

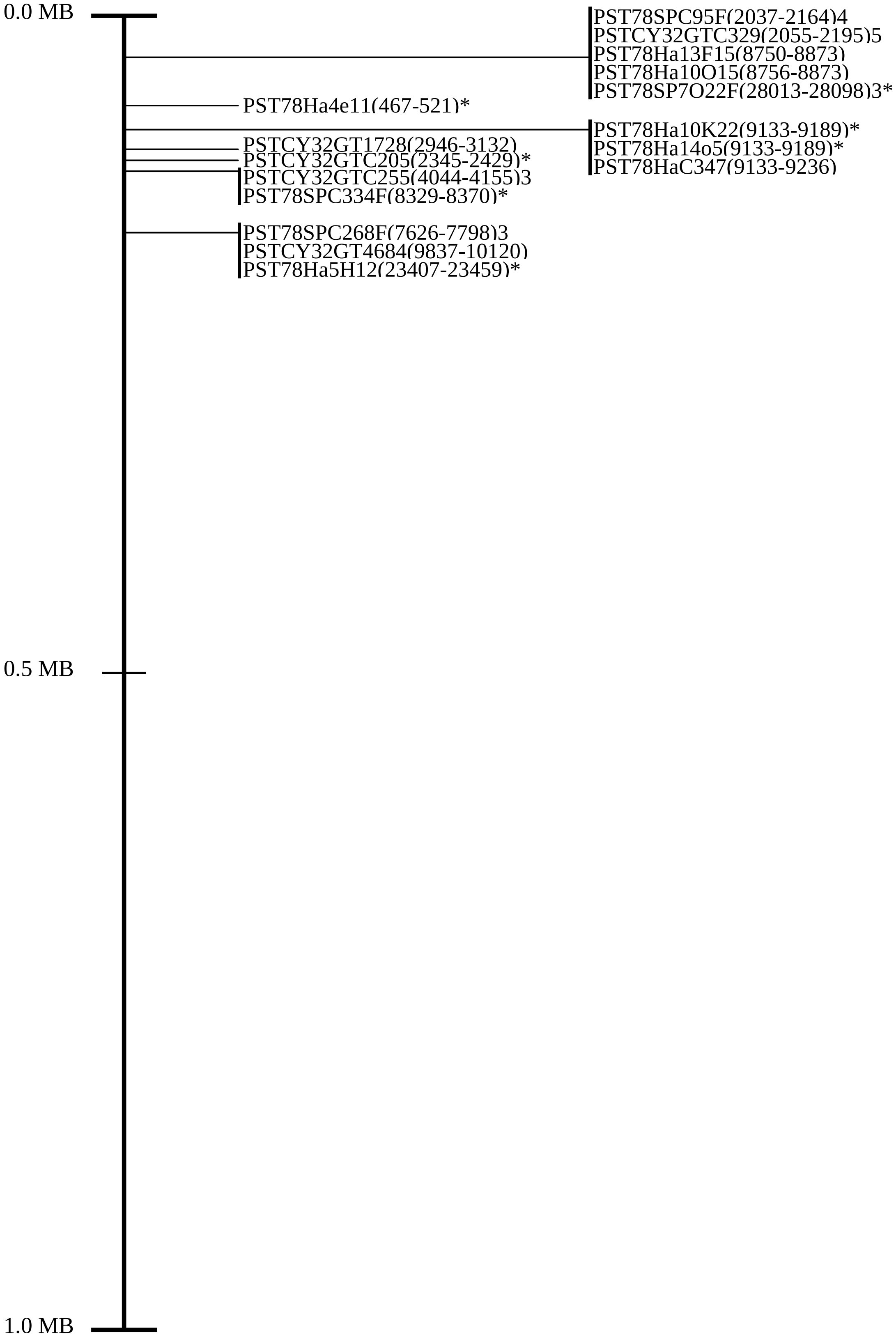

Supercontig 118
length: 164,903 bp

*** = less than 100bp alignment** **| = clones in the same contig**

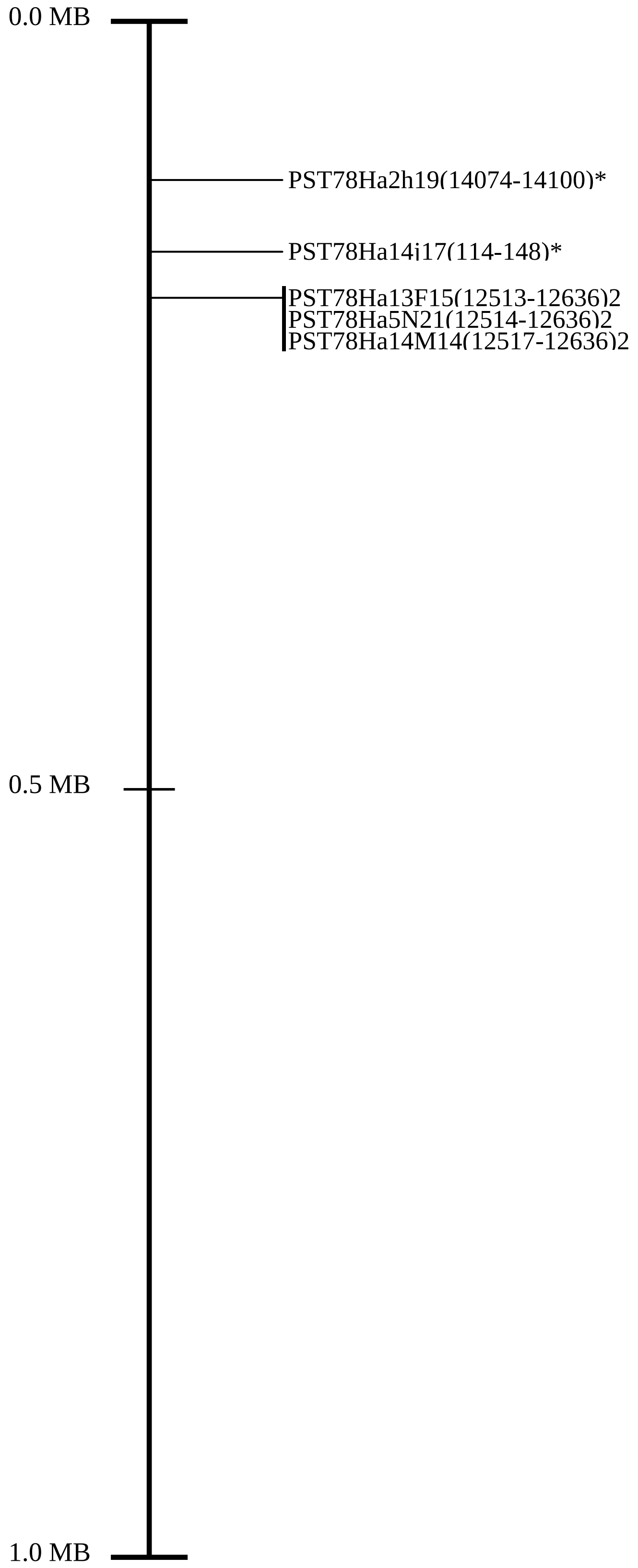

Supercontig 119
length: 180,073 bp

*** = less than 100bp alignment** **| = clones in the same contig**

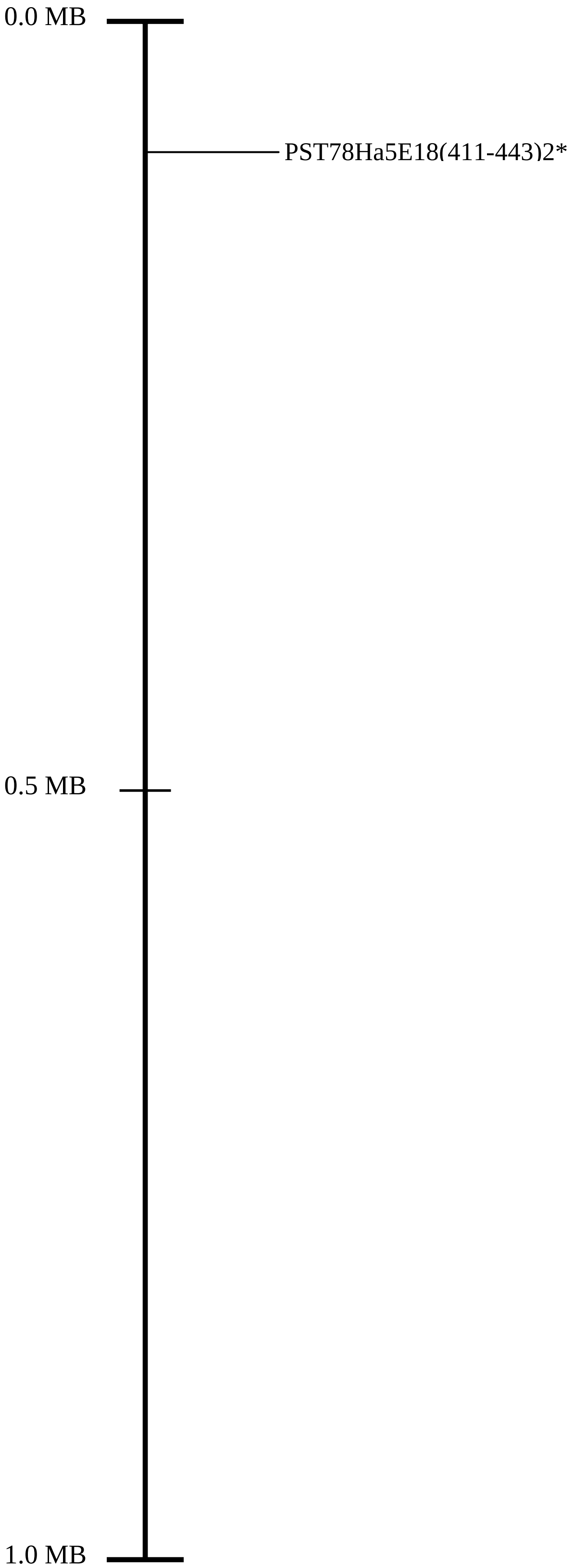

Supercontig 120
length: 132,002 bp

*** = less than 100bp alignment** **| = clones in the same contig**

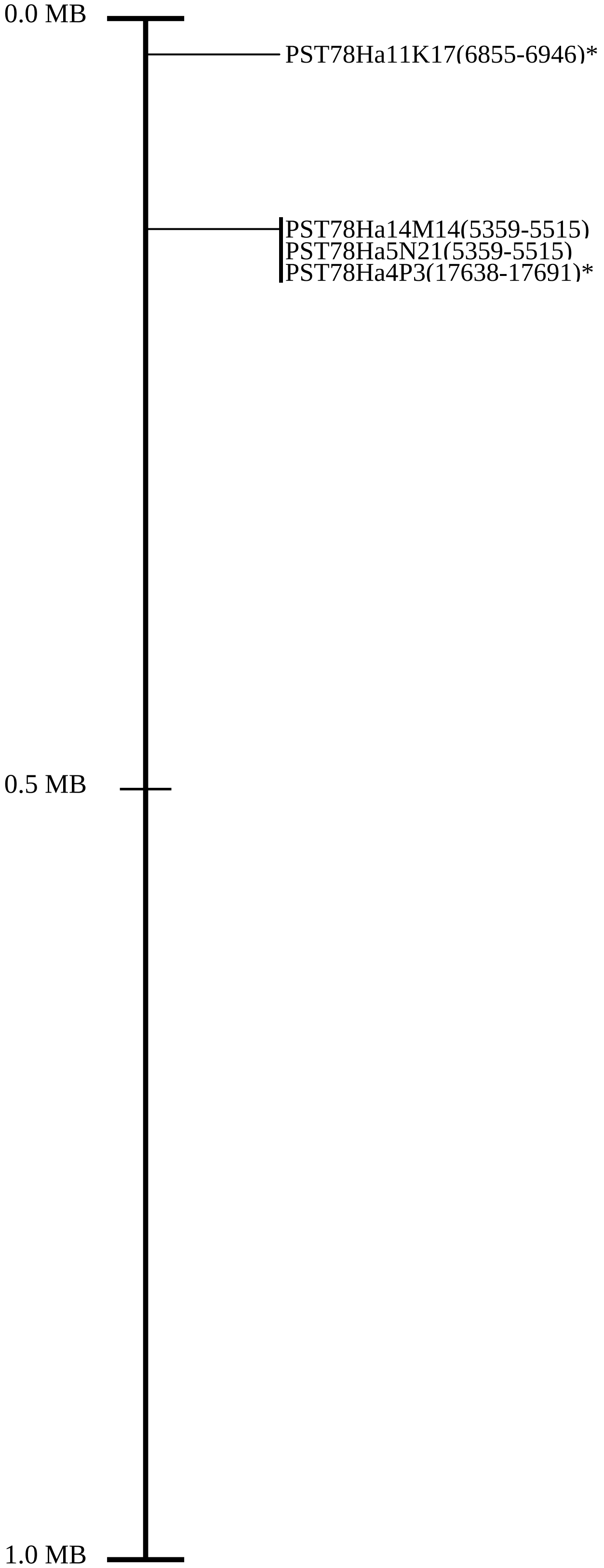

Supercontig 121
length: 136,681 bp

*** = less than 100bp alignment** **| = clones in the same contig**

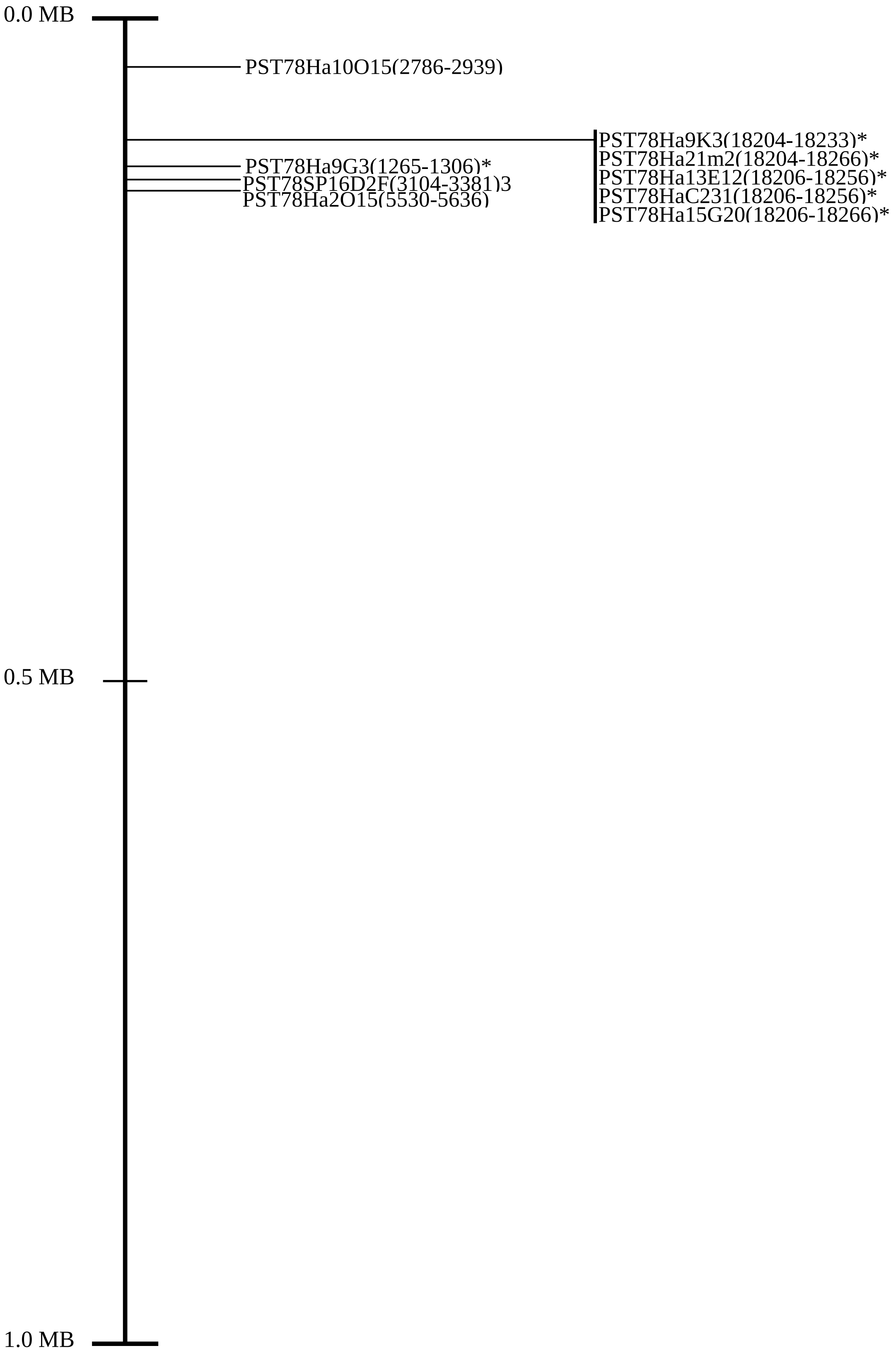

Supercontig 122
length: 141,975 bp

*** = less than 100bp alignment** **| = clones in the same contig**

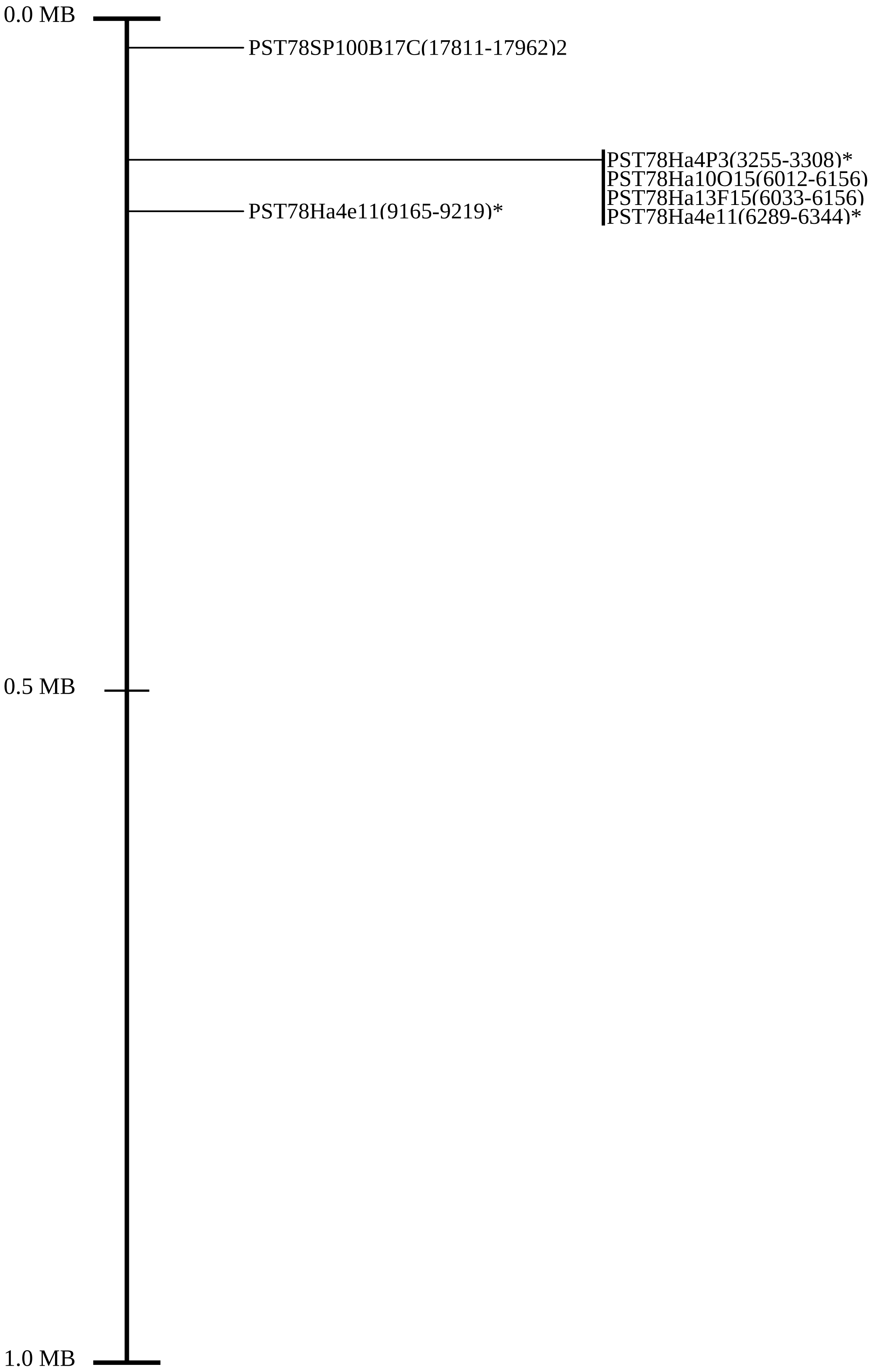

Supercontig 123
length: 142,526 bp

*** = less than 100bp alignment** **| = clones in the same contig**

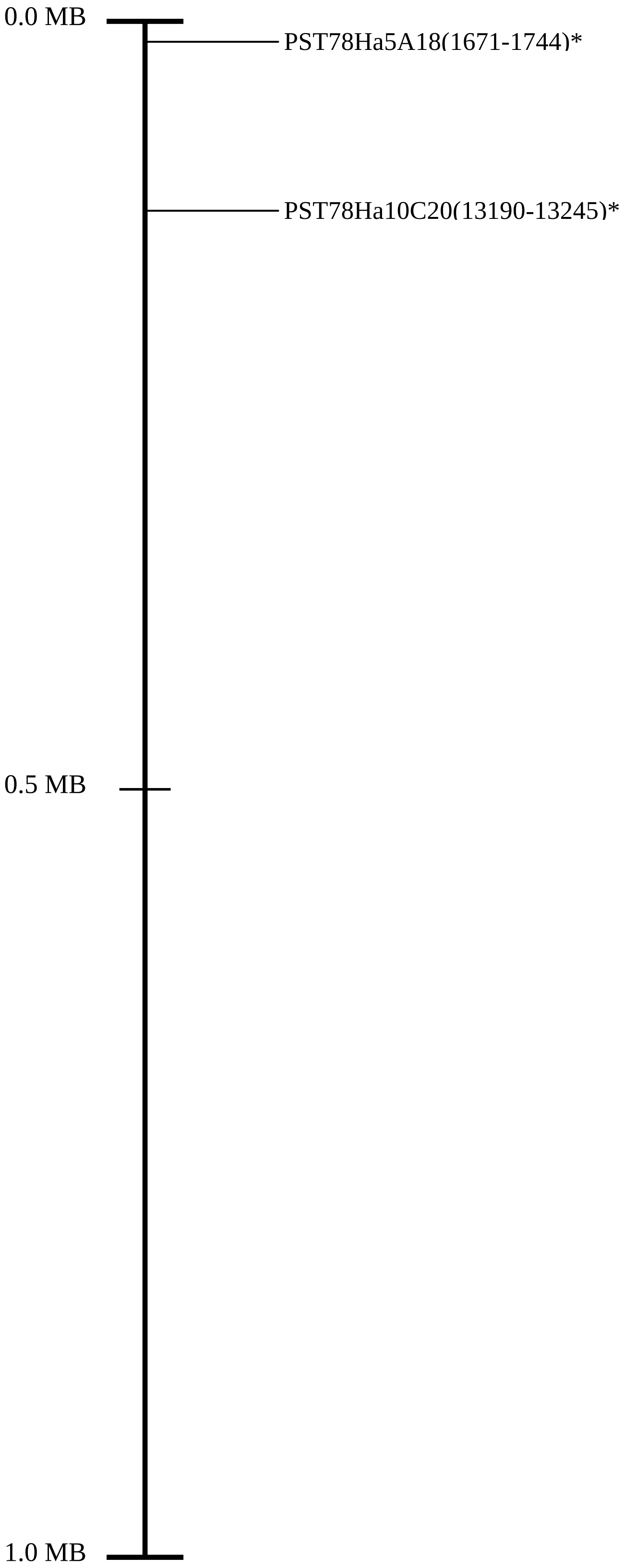

Supercontig 124
length: 122,507 bp

*** = less than 100bp alignment**   **| = clones in the same contig**

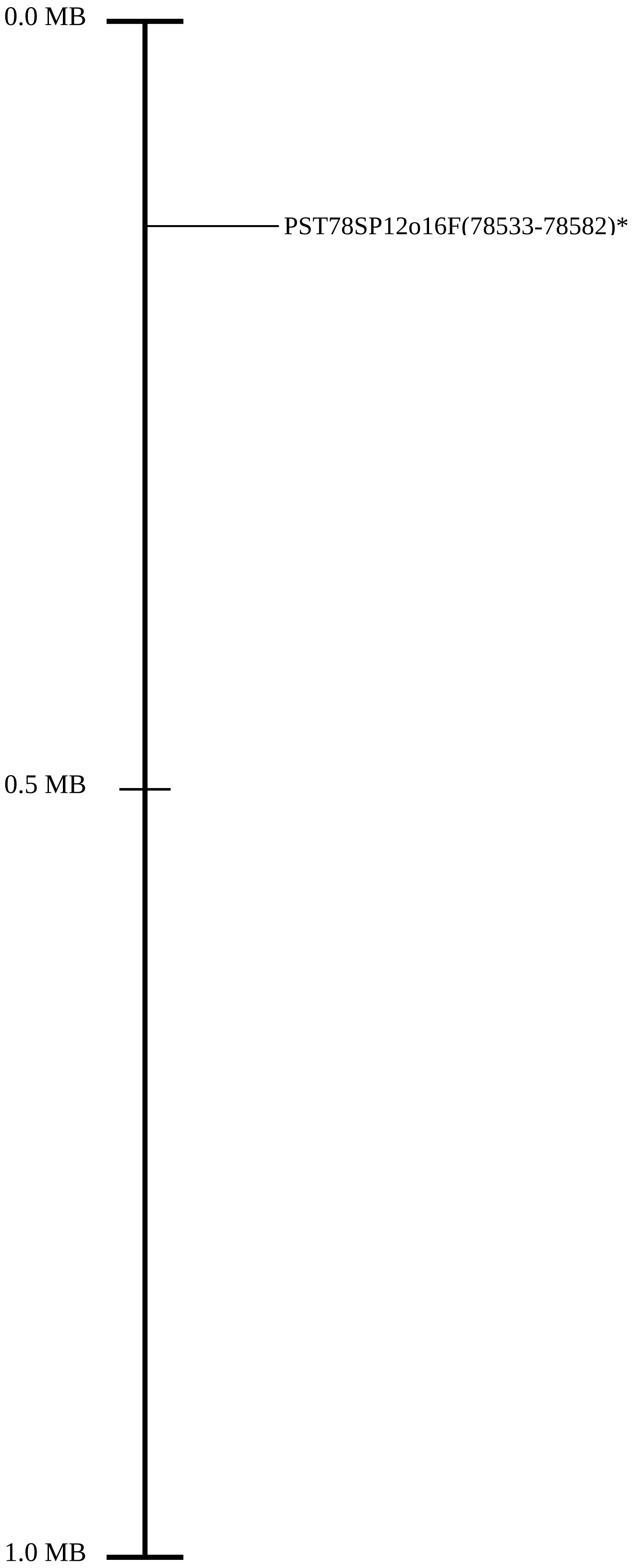

Supercontig 125
length: 141,384 bp

*** = less than 100bp alignment**

| = clones in the same contig

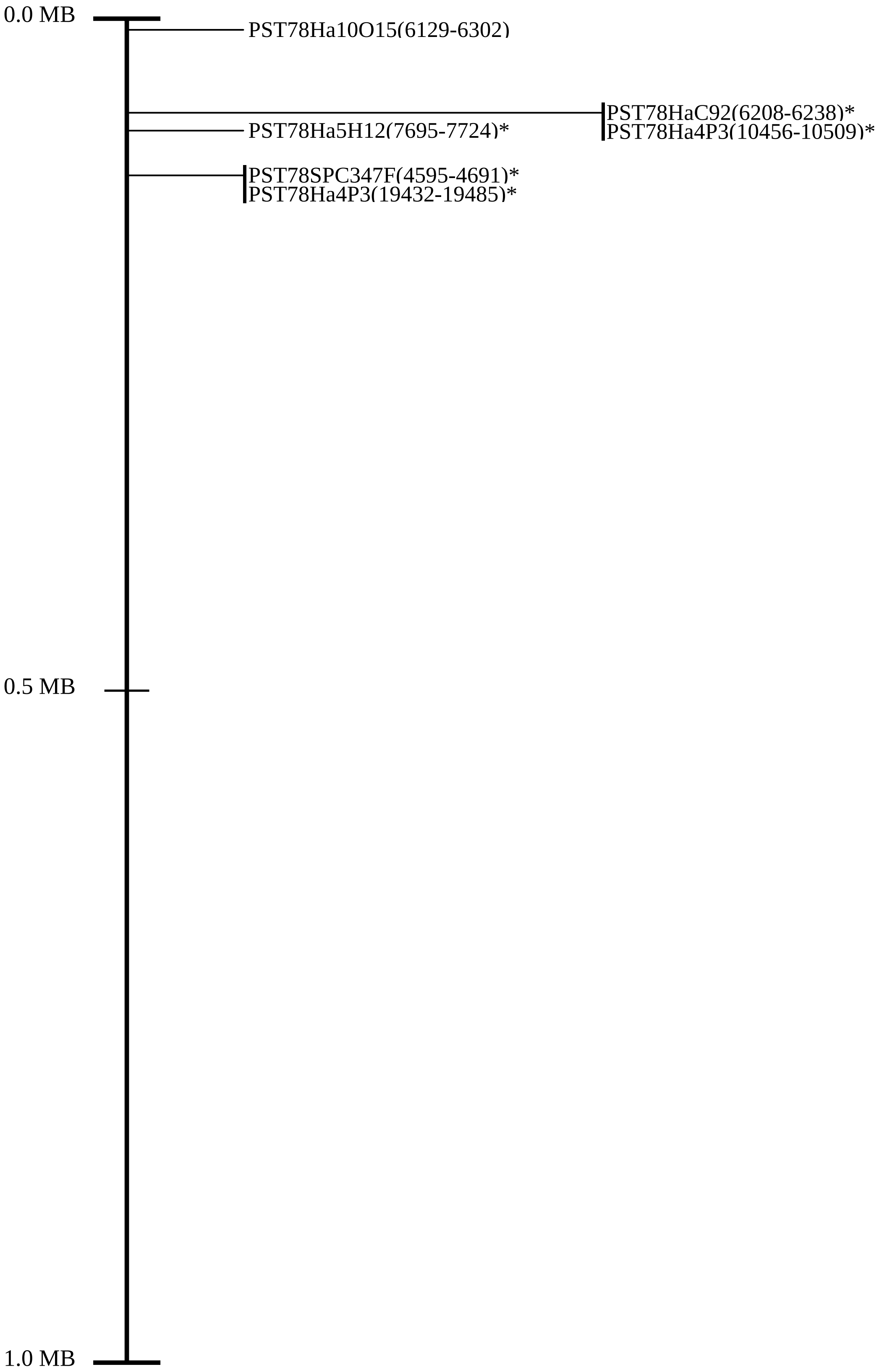

Supercontig 126
length: 132,883 bp

*** = less than 100bp alignment** **| = clones in the same contig**

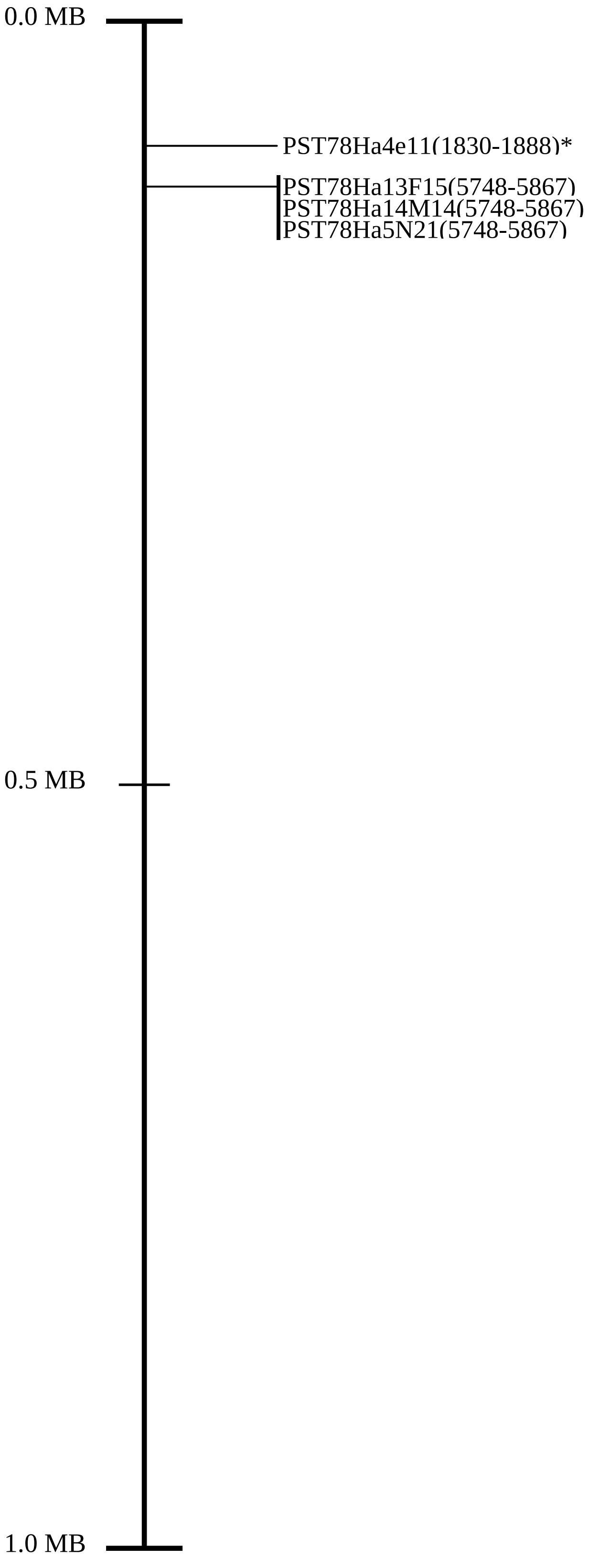

Supercontig 127
length: 158,529 bp

*** = less than 100bp alignment**

| = clones in the same contig

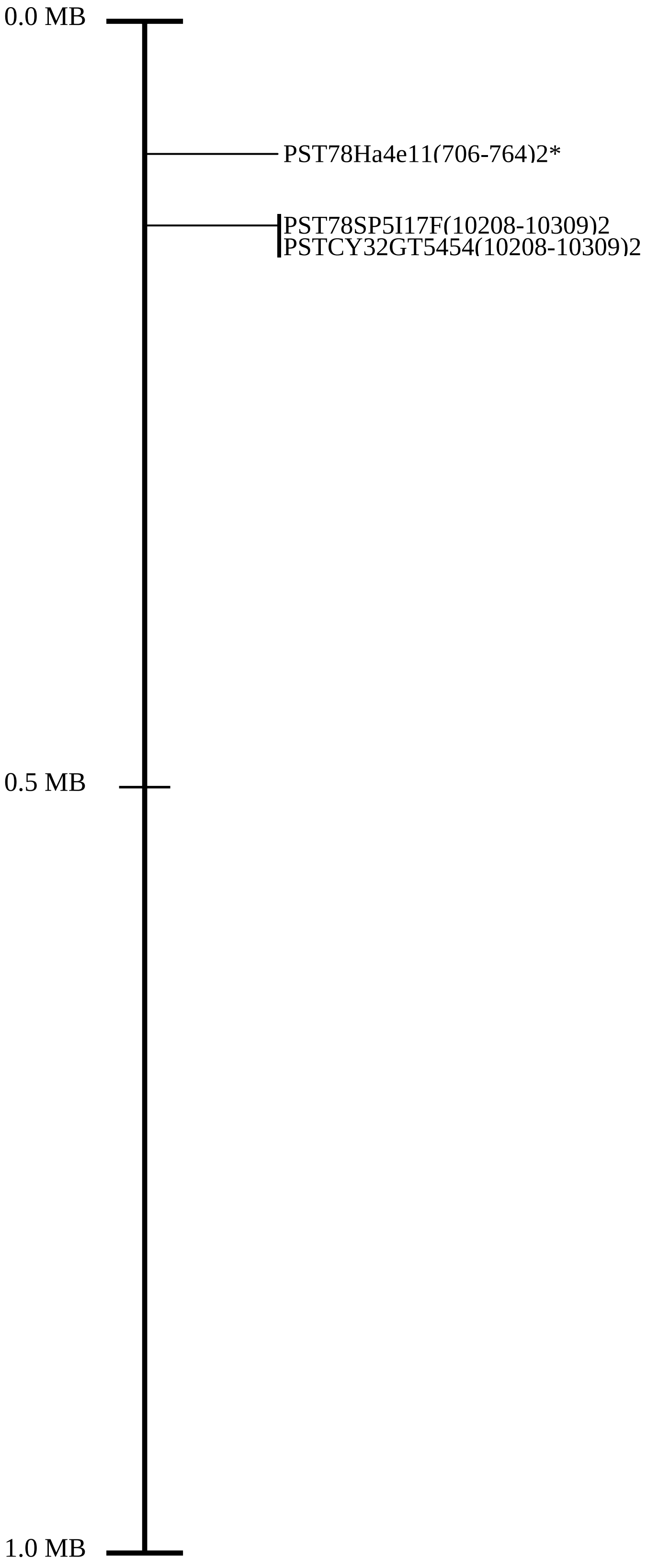

Supercontig 128
length: 133,434 bp

*** = less than 100bp alignment**

| = clones in the same contig

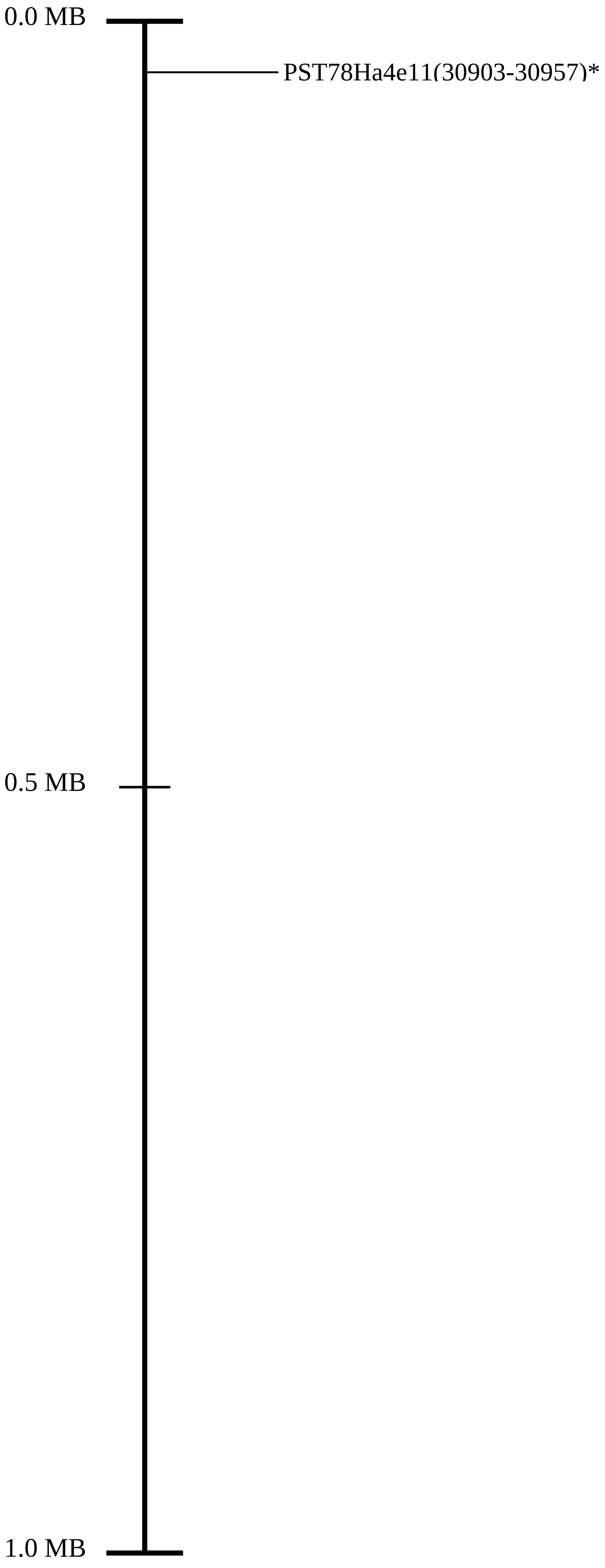

Supercontig 129
length: 156,670 bp

*** = less than 100bp alignment**

| = clones in the same contig

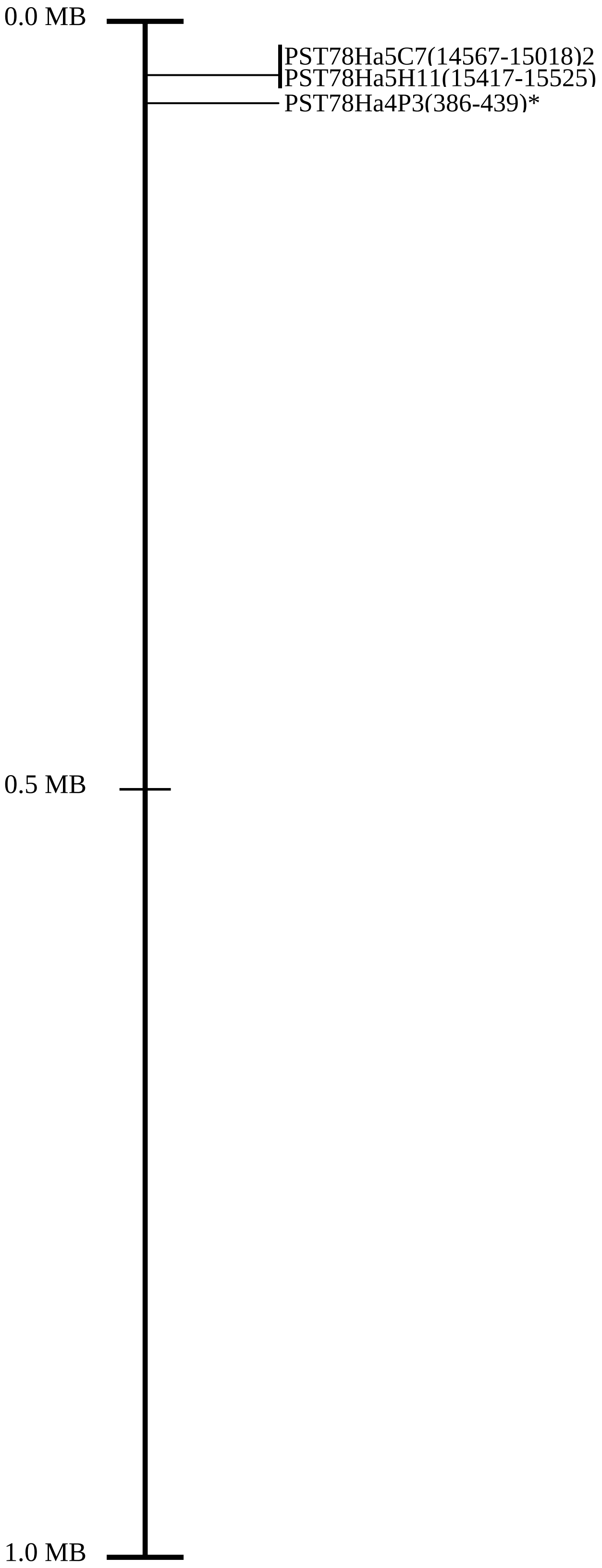

Supercontig 130
length: 127,762 bp

*** = less than 100bp alignment**

| = clones in the same contig

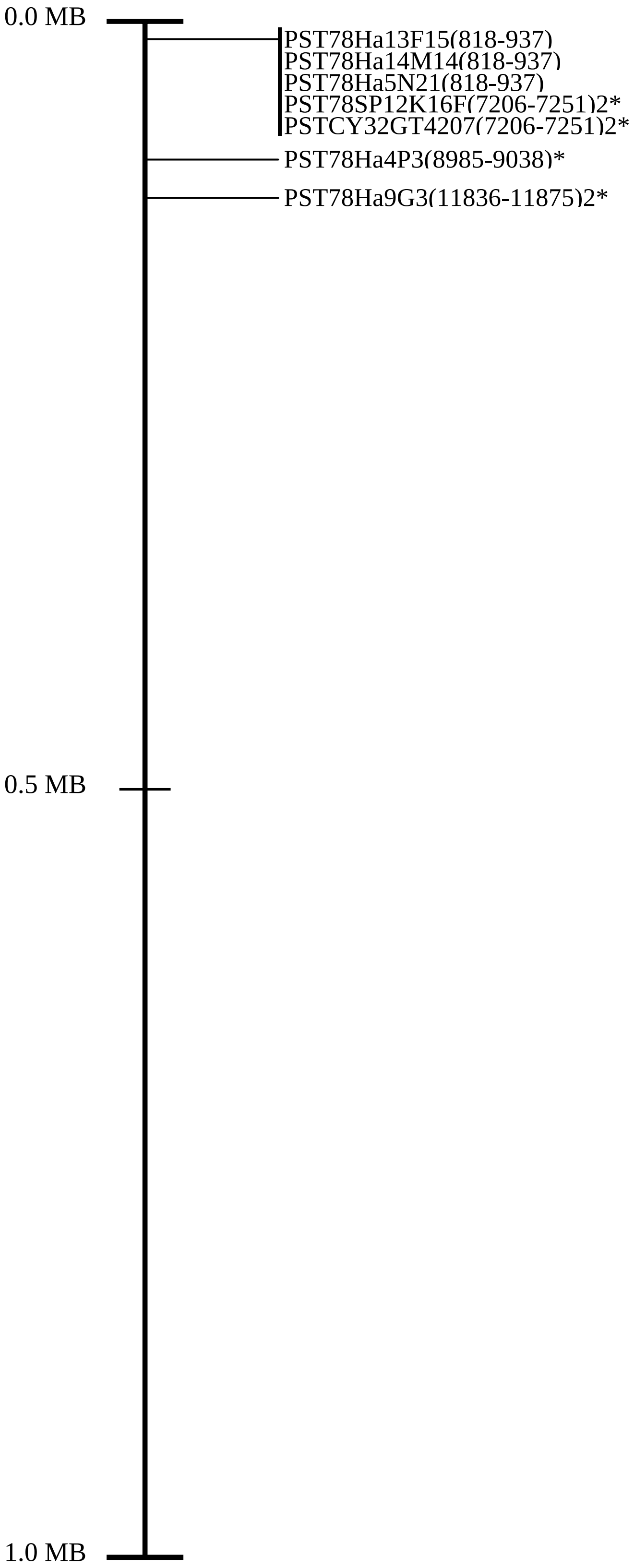

Supercontig 131
length: 114,788 bp

*** = less than 100bp alignment** **| = clones in the same contig**

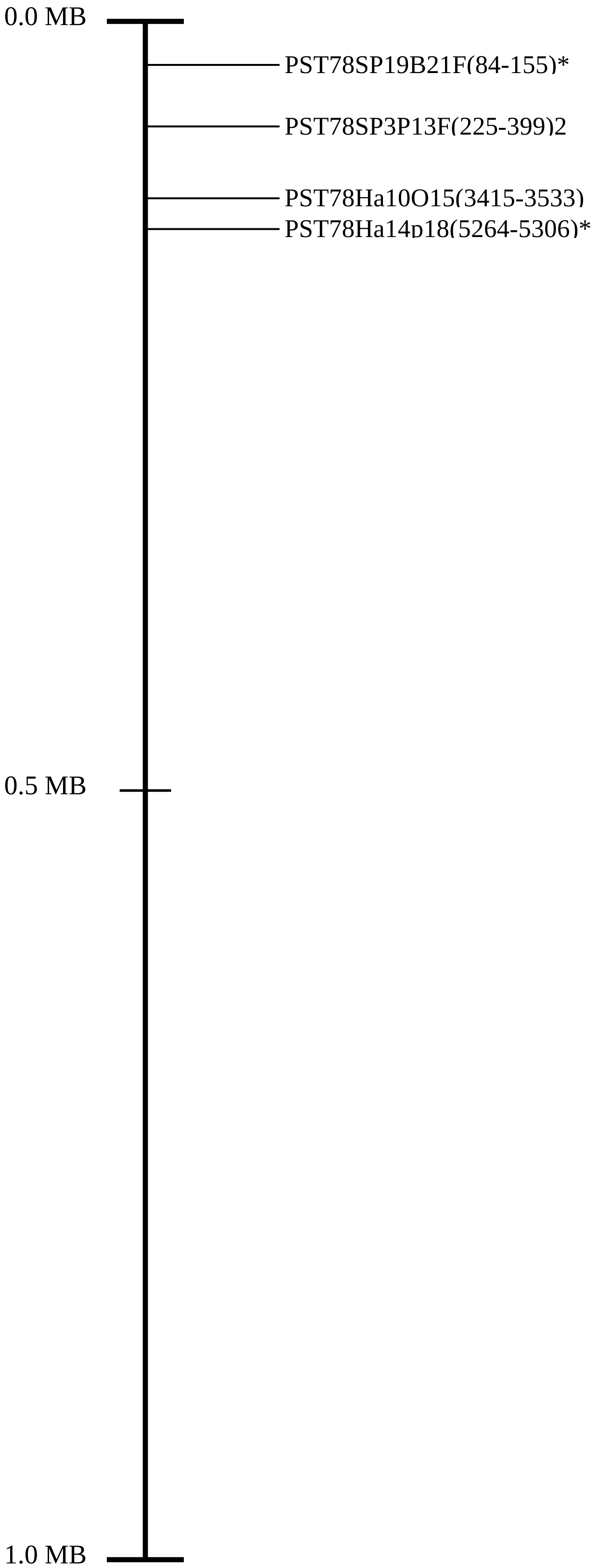

Supercontig 132
length: 135,433 bp

*** = less than 100bp alignment** **| = clones in the same contig**

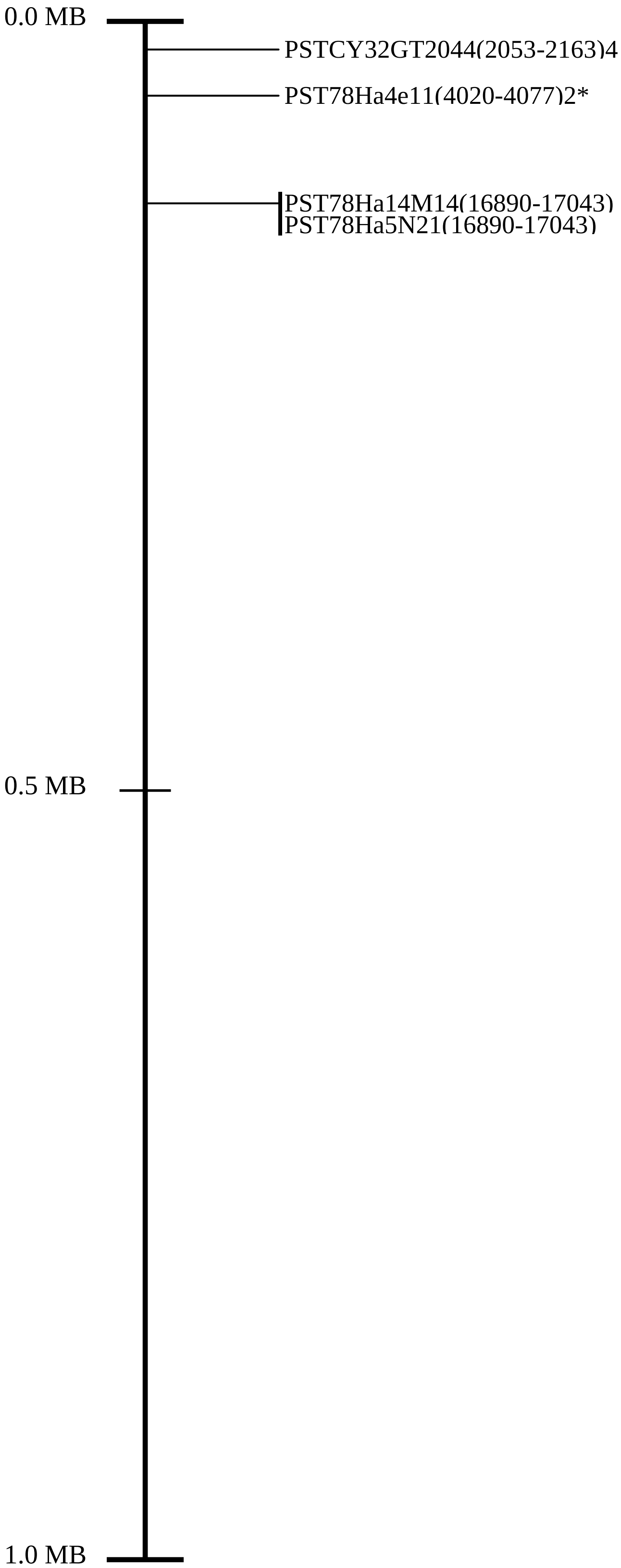

Supercontig 133
length: 128,998 bp

*** = less than 100bp alignment** **| = clones in the same contig**

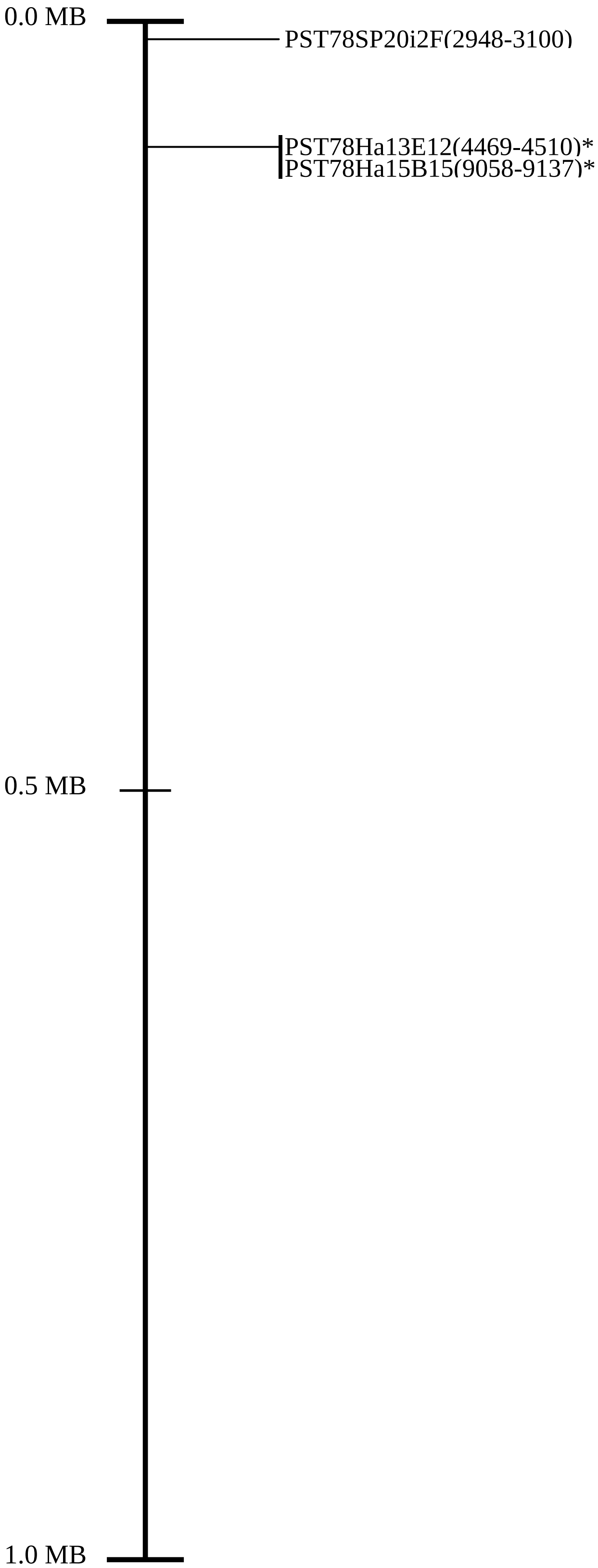

Supercontig 135
length: 163,446 bp

*** = less than 100bp alignment** **| = clones in the same contig**

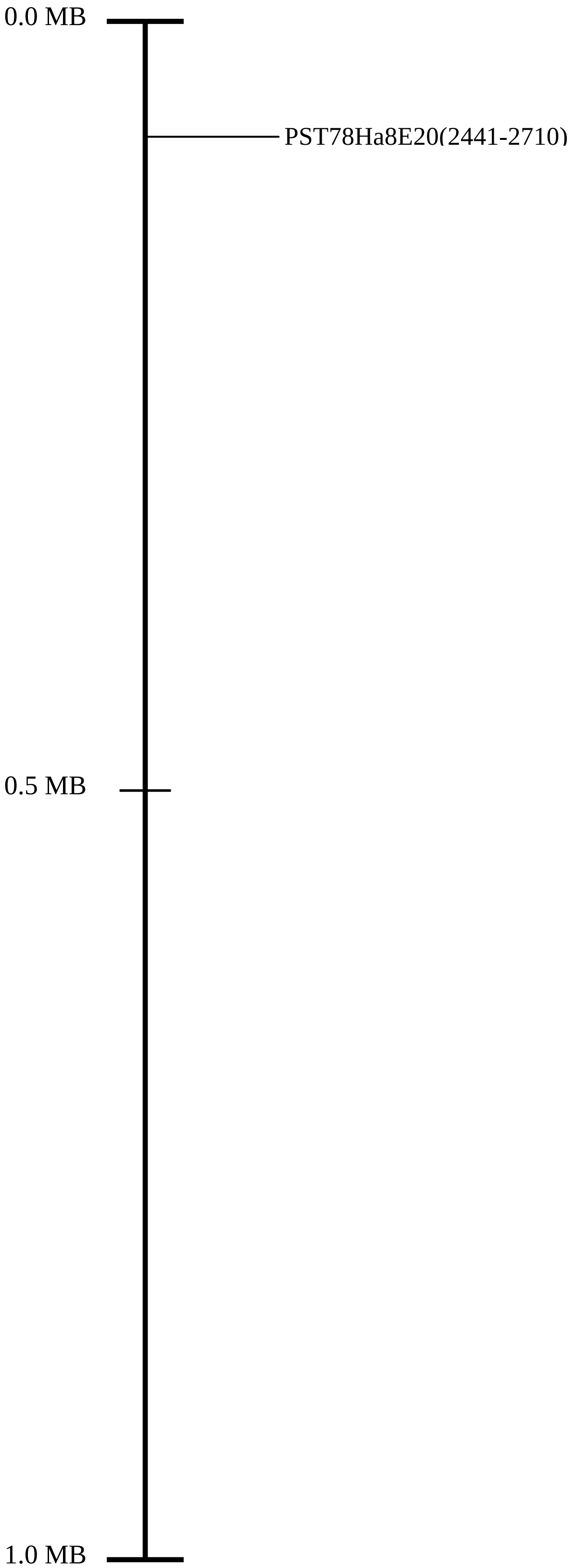

Supercontig 137
length: 105,823 bp

*** = less than 100bp alignment**

| = clones in the same contig

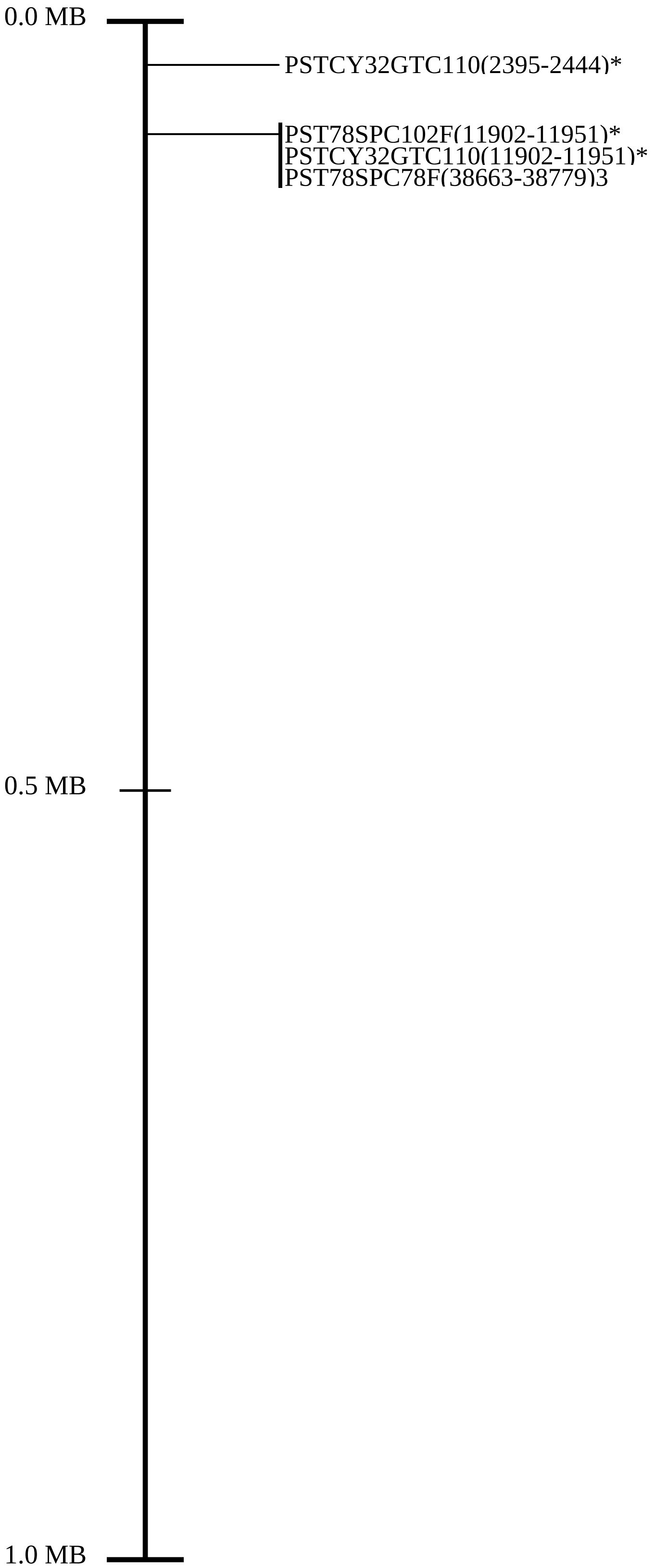

Supercontig 138
length: 90,221 bp

*** = less than 100bp alignment** **| = clones in the same contig**

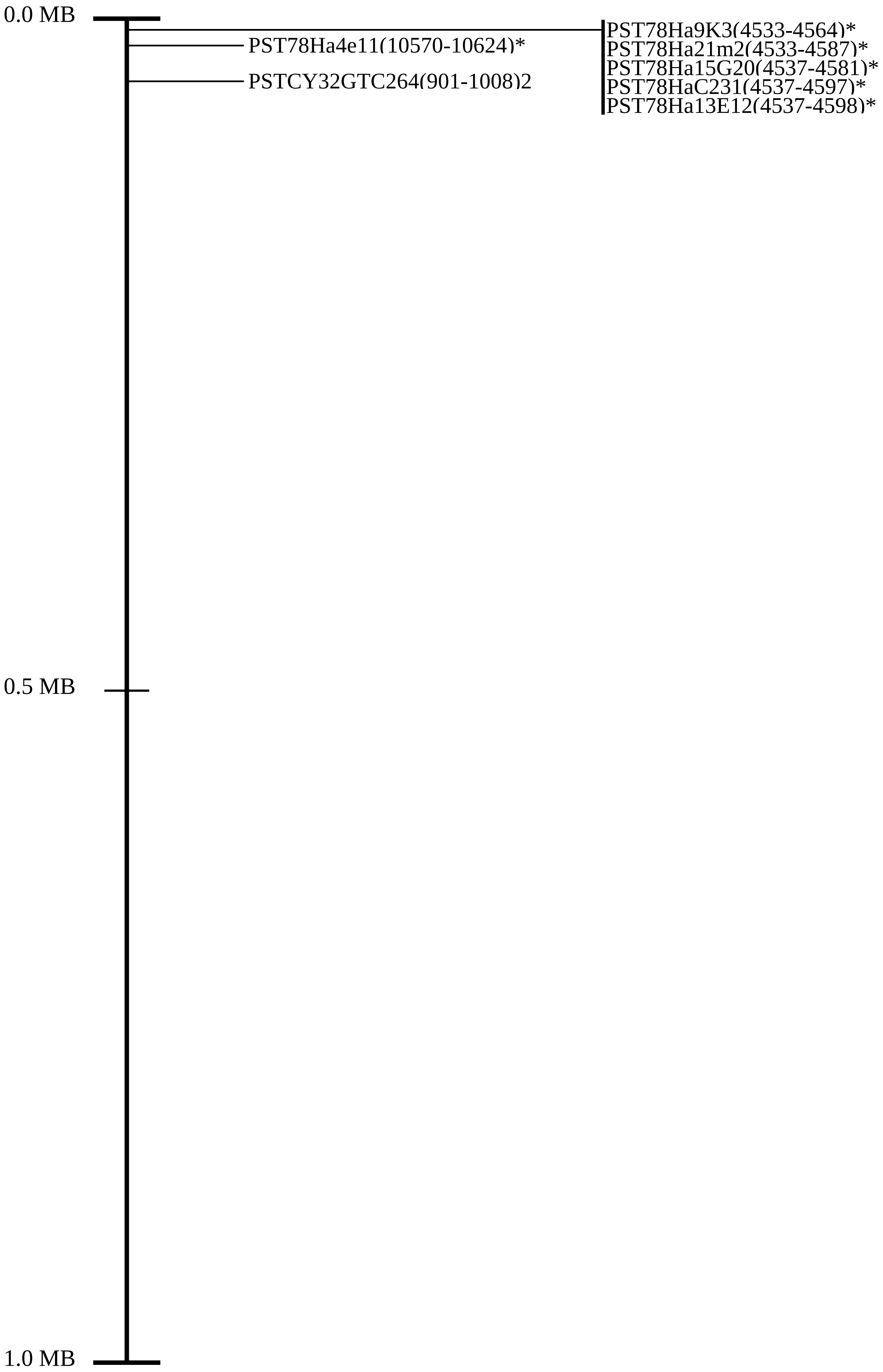

Supercontig 139
length: 93,586 bp

*** = less than 100bp alignment**

| = clones in the same contig

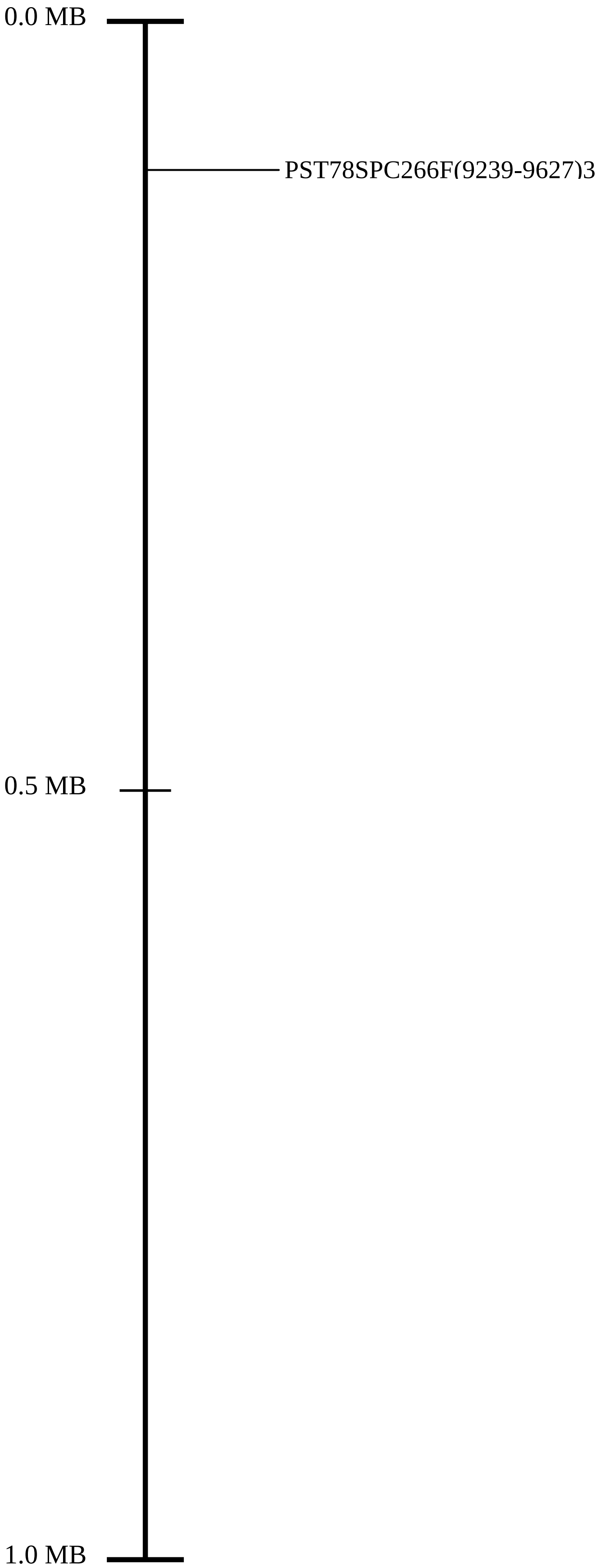

Supercontig 140
length: 96,224 bp

*** = less than 100bp alignment**

| = clones in the same contig

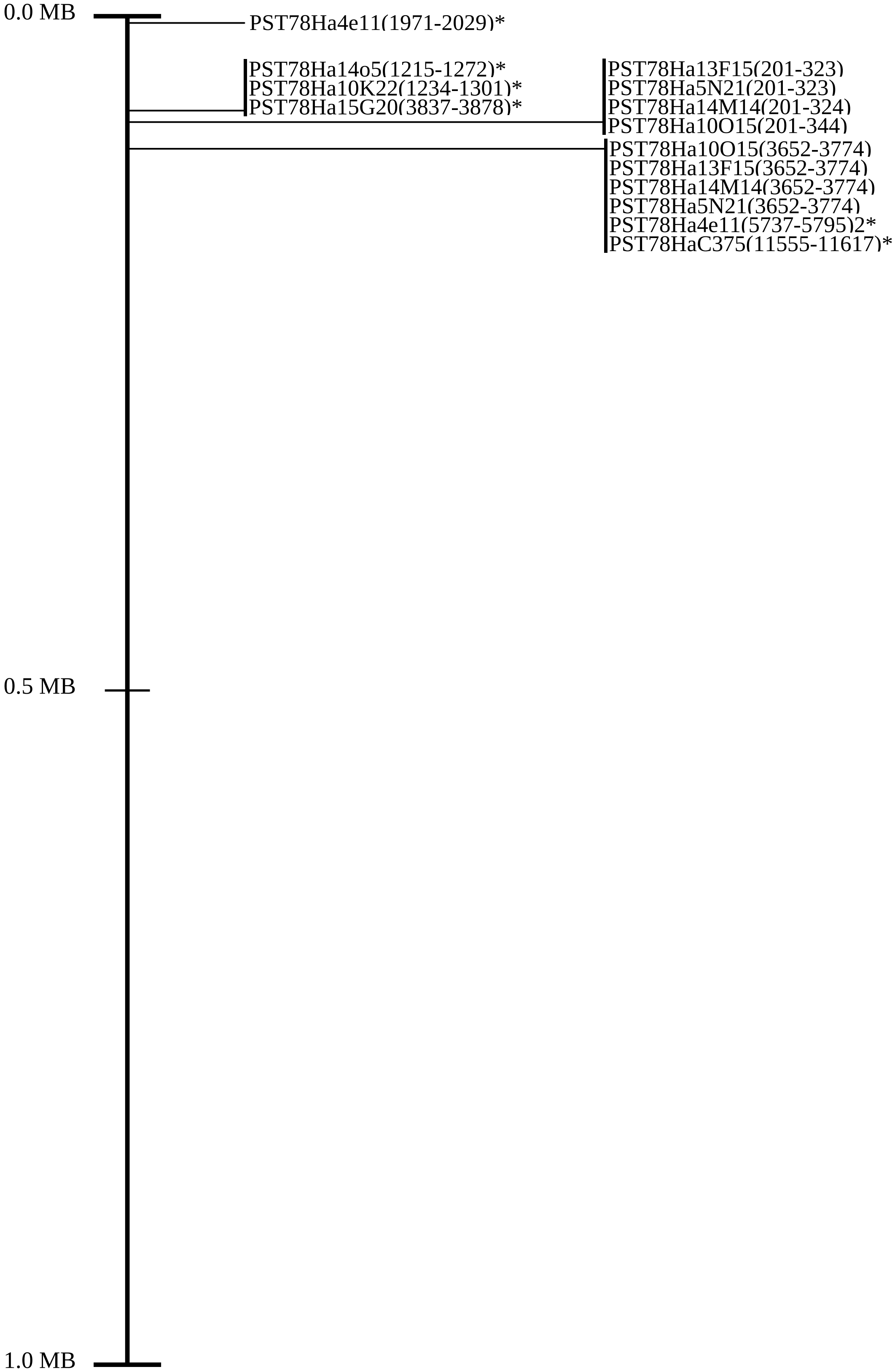

Supercontig 141
length: 98,031 bp

*** = less than 100bp alignment** **| = clones in the same contig**

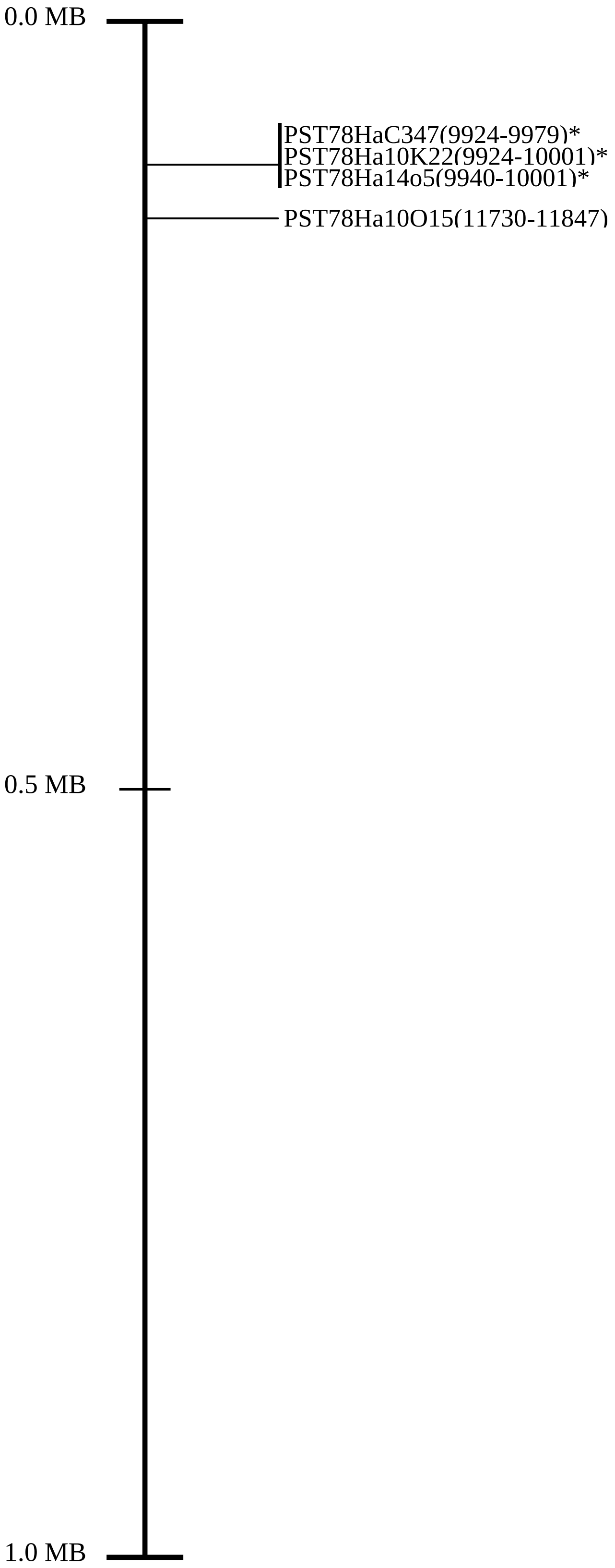

Supercontig 142
length: 132,305 bp

*** = less than 100bp alignment**

| = clones in the same contig

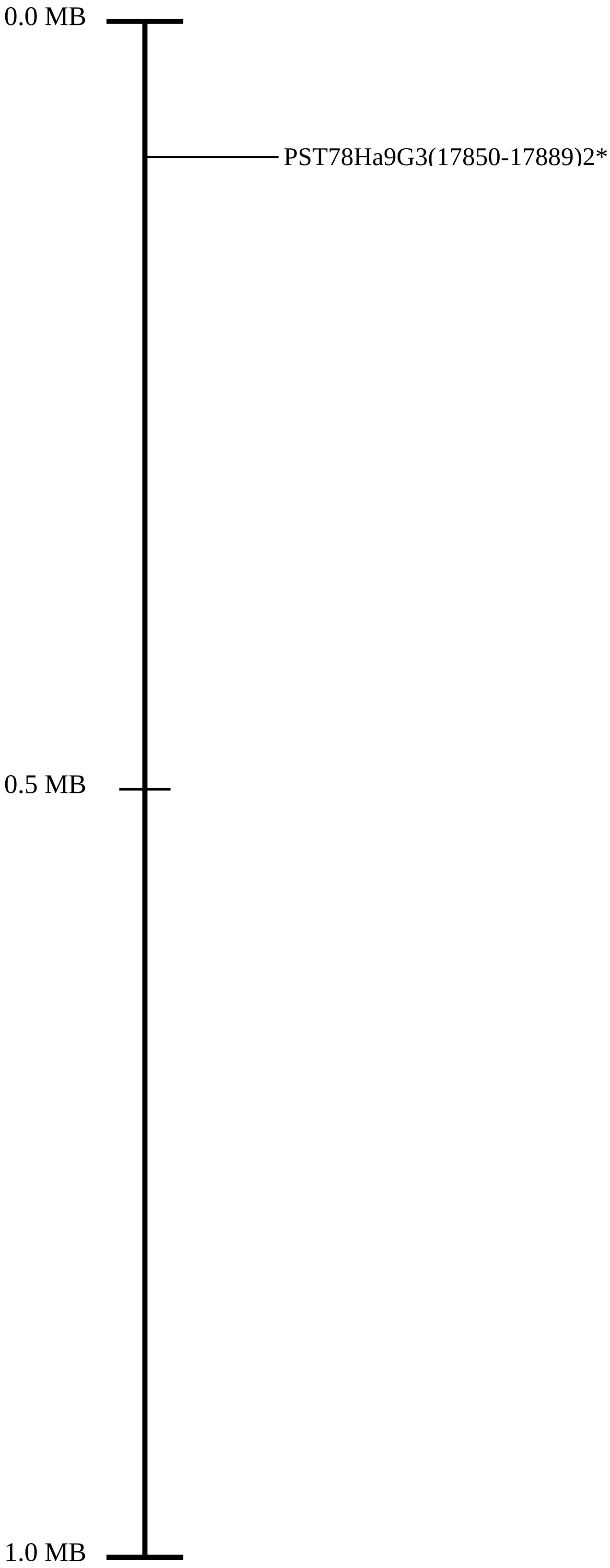

Supercontig 143
length: 87,623 bp

*** = less than 100bp alignment**

| = clones in the same contig

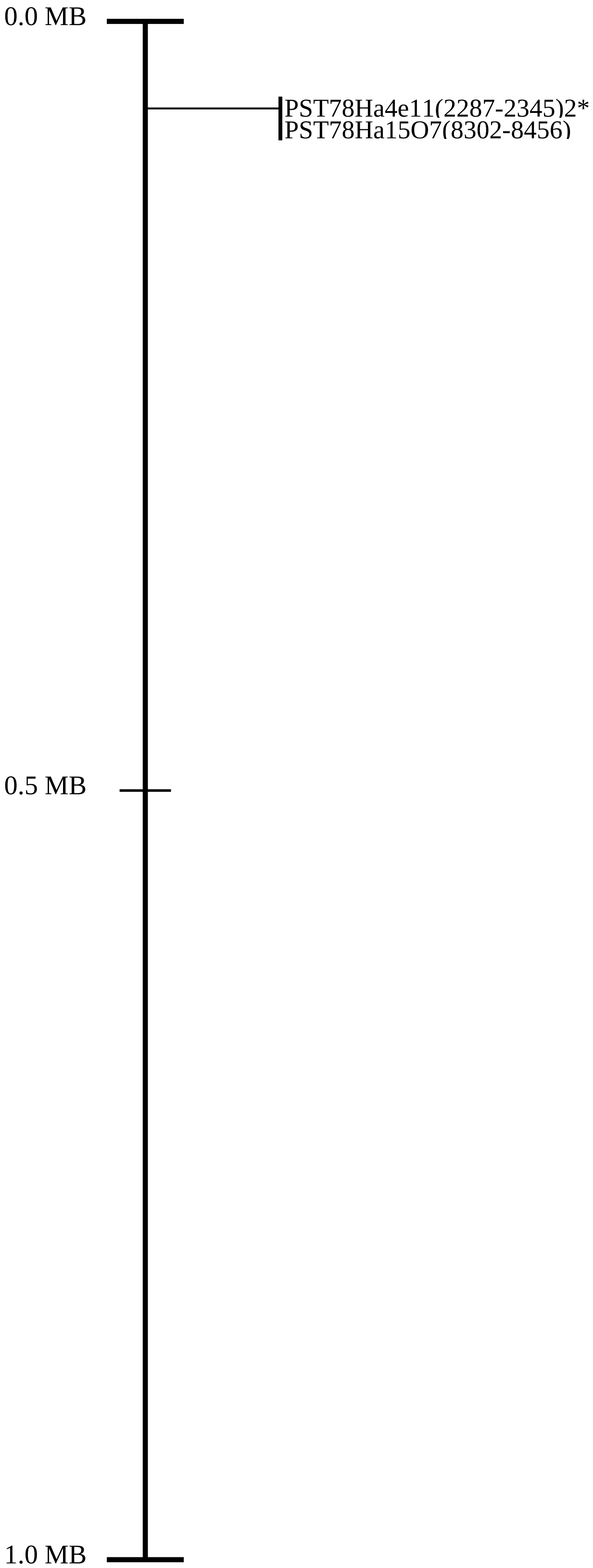

Supercontig 144
length: 85,648 bp

*** = less than 100bp alignment** **| = clones in the same contig**

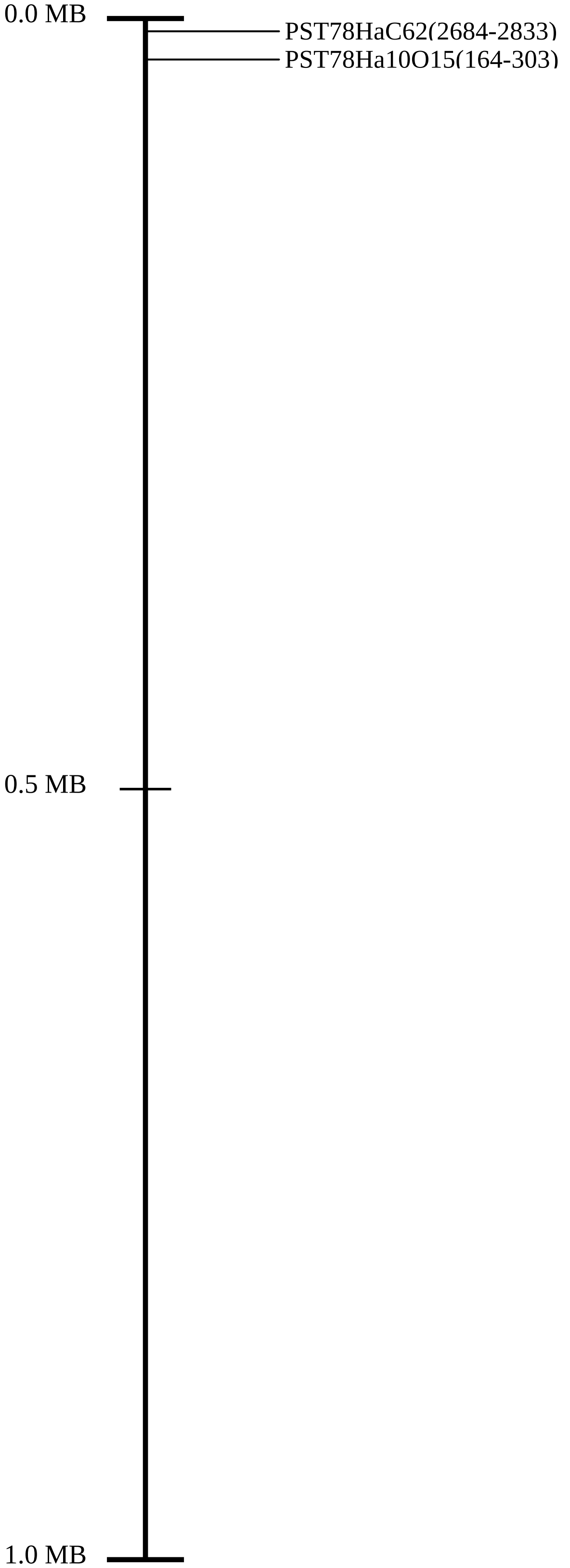

Supercontig 145
length: 74,269 bp

*** = less than 100bp alignment**

| = clones in the same contig

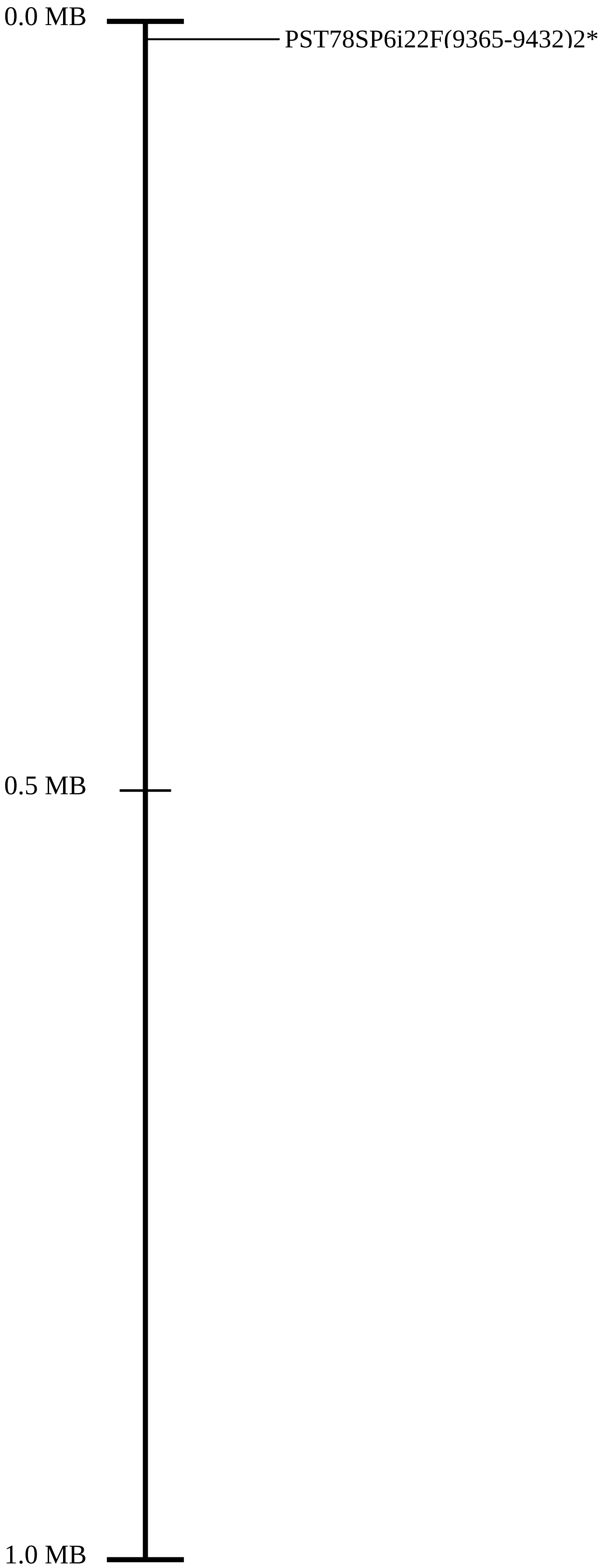

Supercontig 146
length: 66,839 bp

*** = less than 100bp alignment**

| = clones in the same contig

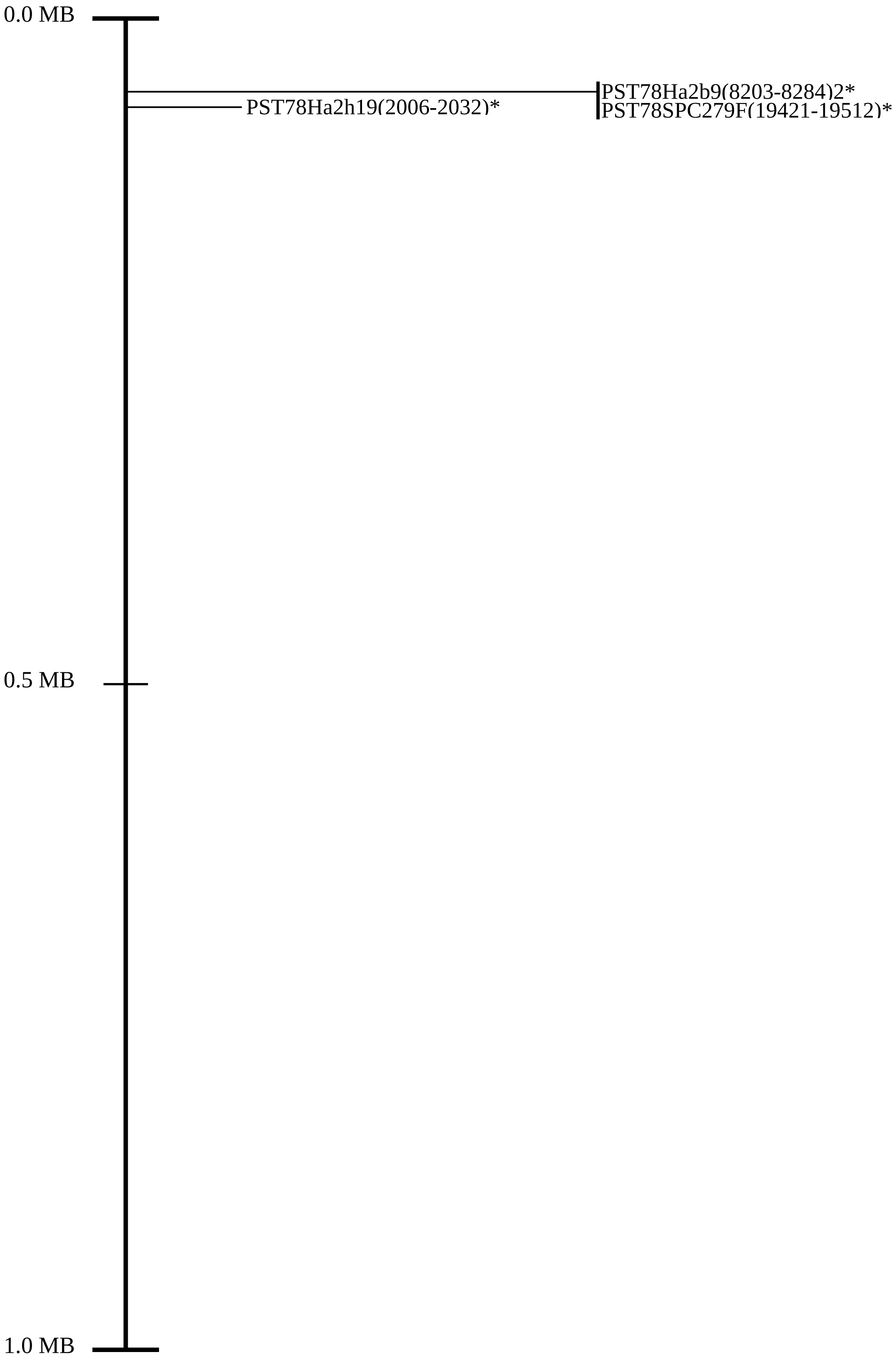

Supercontig 147
length: 66,397 bp

*** = less than 100bp alignment**

| = clones in the same contig

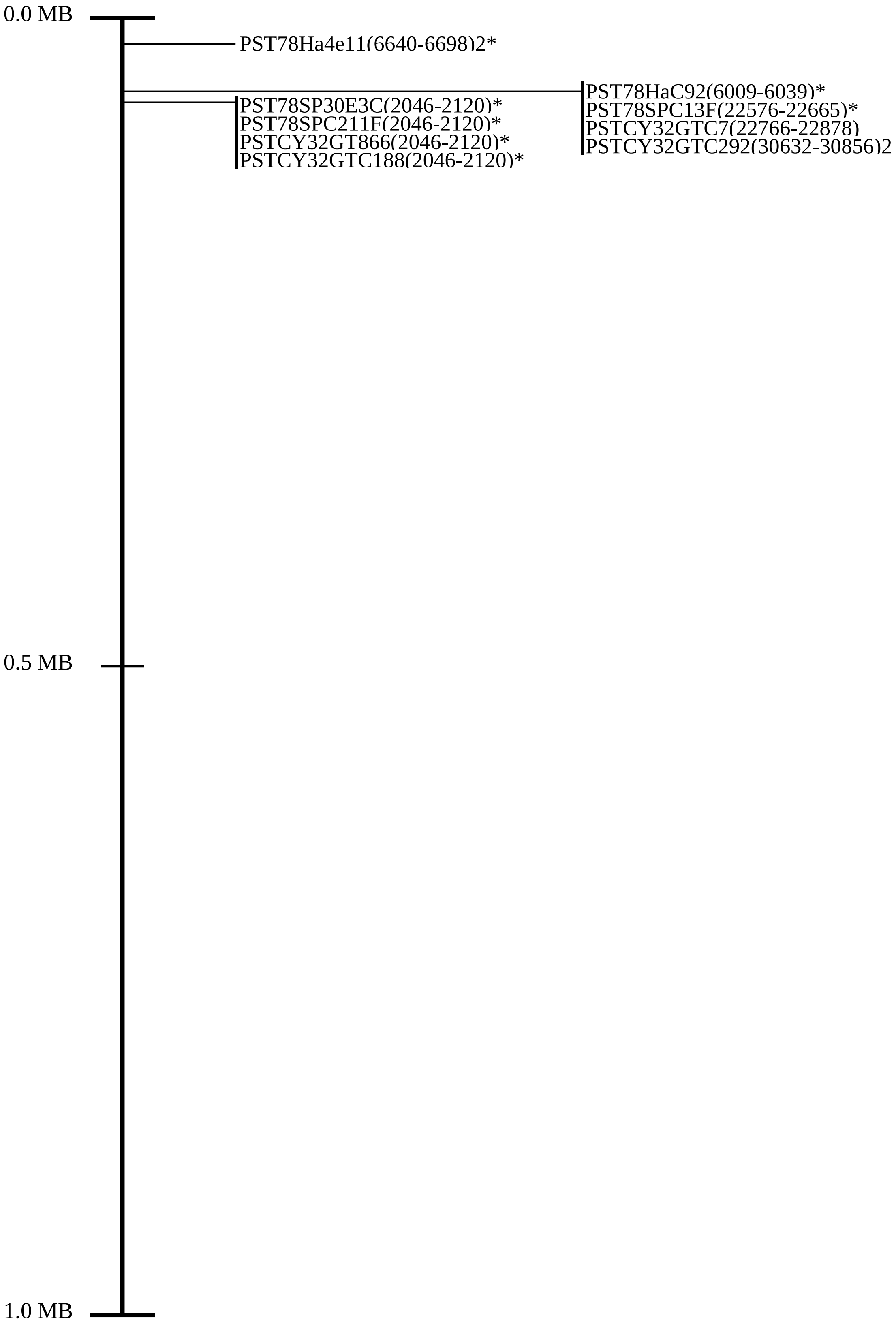

Supercontig 148
length: 64,884 bp

*** = less than 100bp alignment** **| = clones in the same contig**

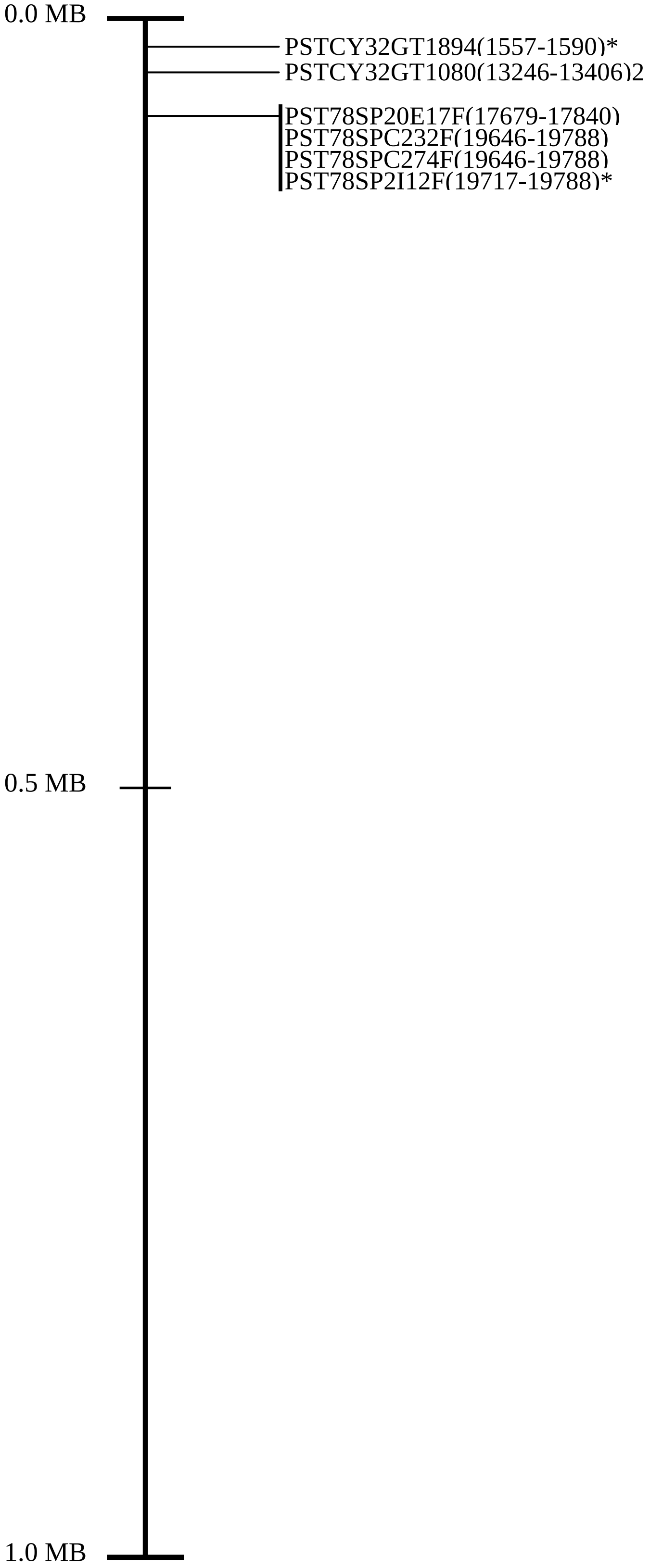

Supercontig 150
length: 63,036 bp

*** = less than 100bp alignment**

| = clones in the same contig

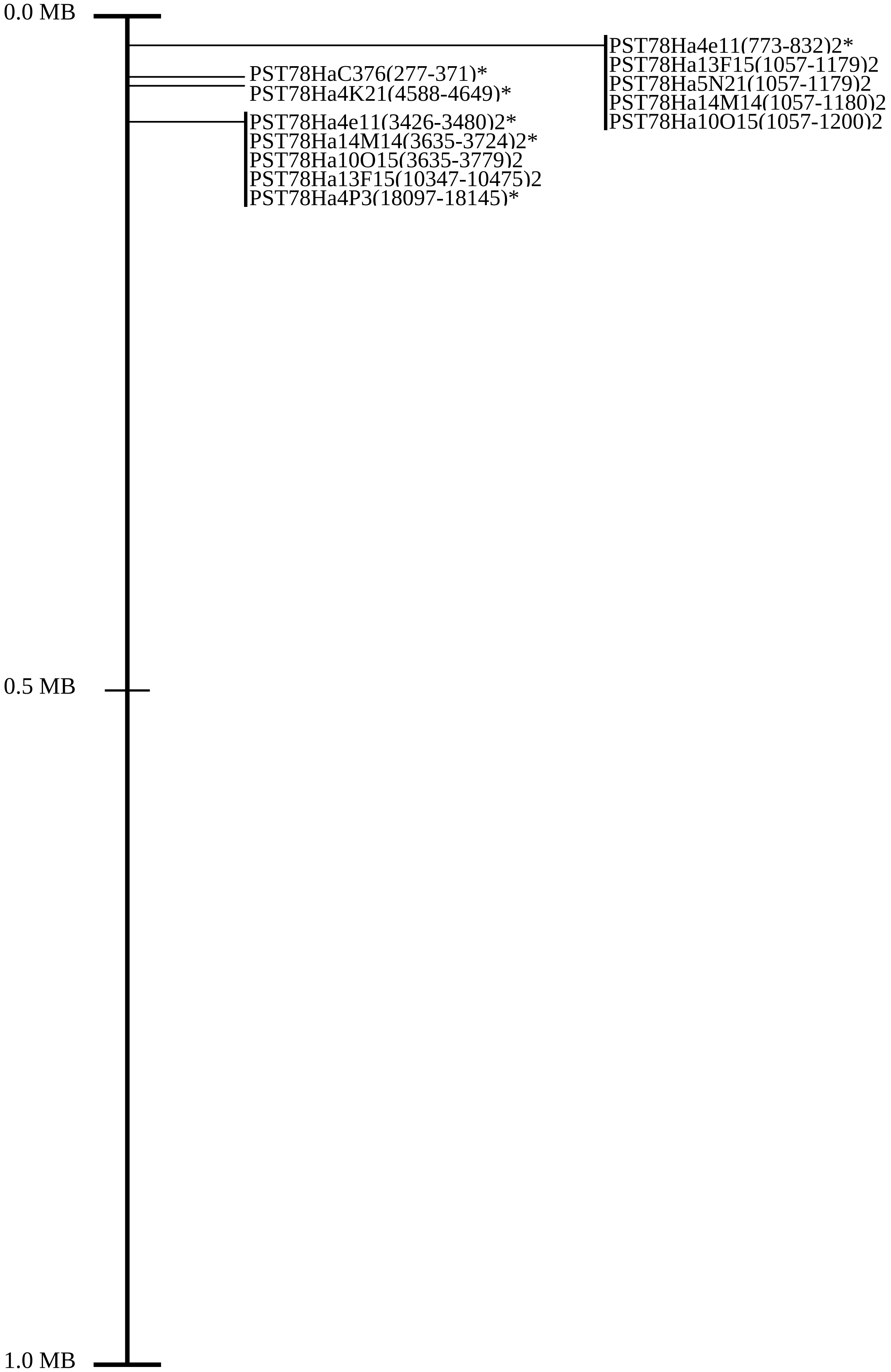

Supercontig 151
length: 78,814 bp

*** = less than 100bp alignment** **| = clones in the same contig**

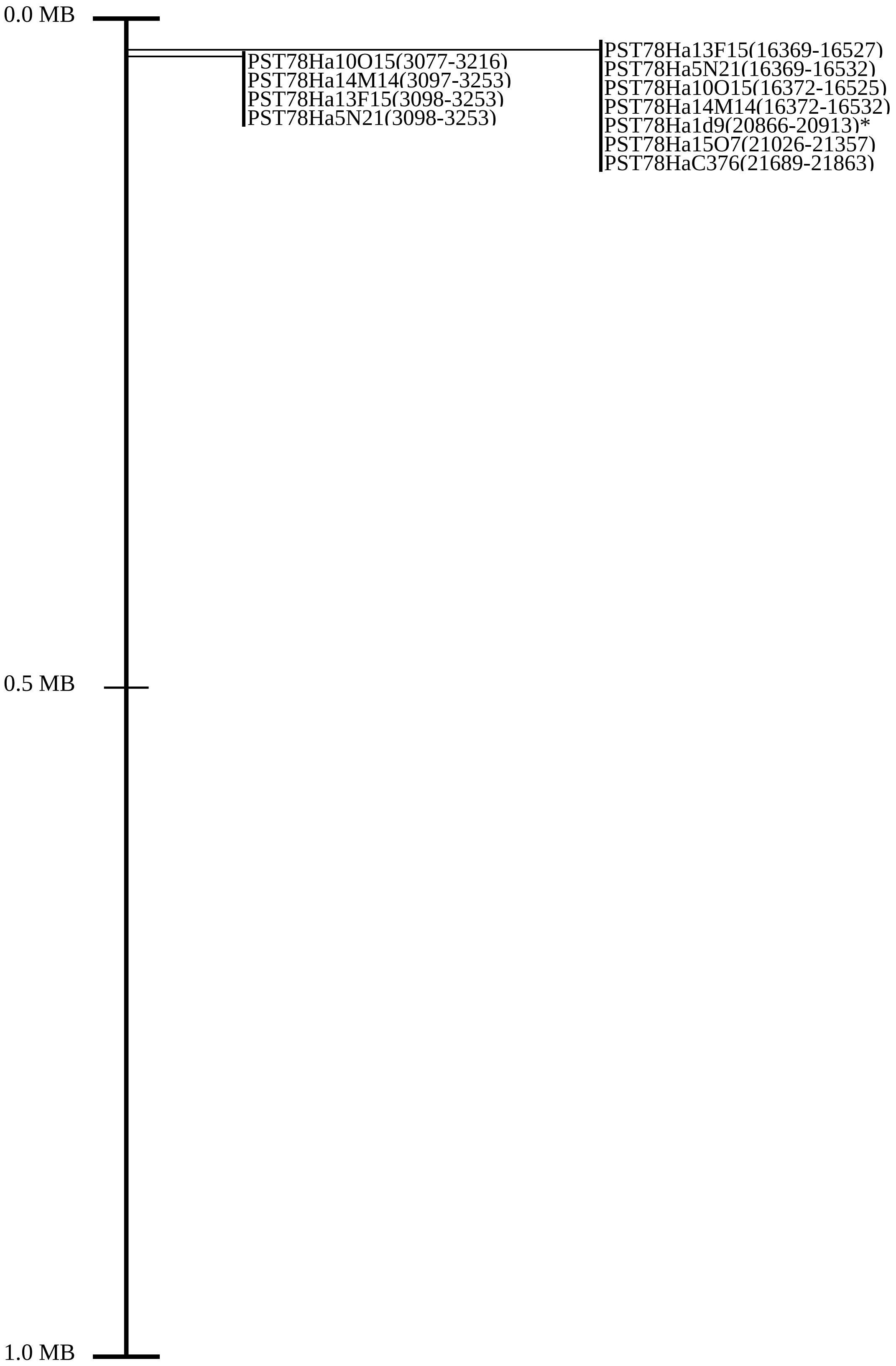

Supercontig 152
length: 68,096 bp

*** = less than 100bp alignment** **| = clones in the same contig**

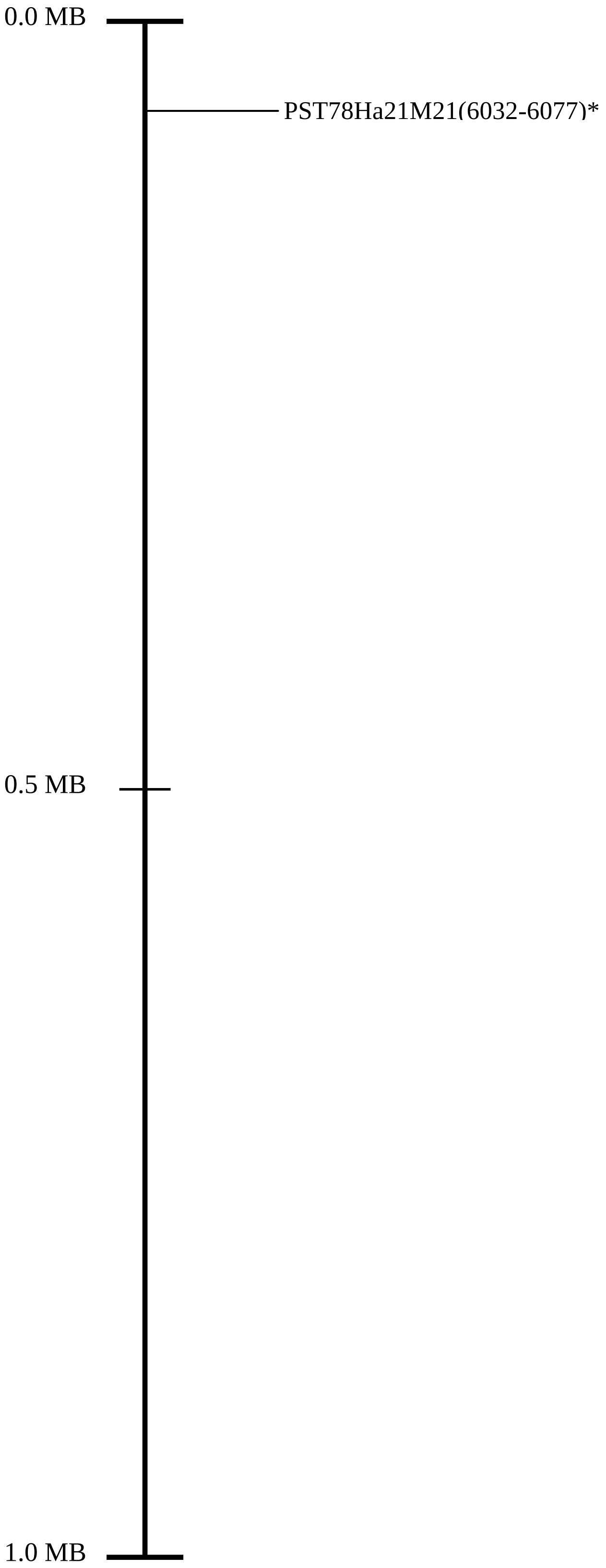

Supercontig 153
length: 58,505 bp

*** = less than 100bp alignment**

| = clones in the same contig

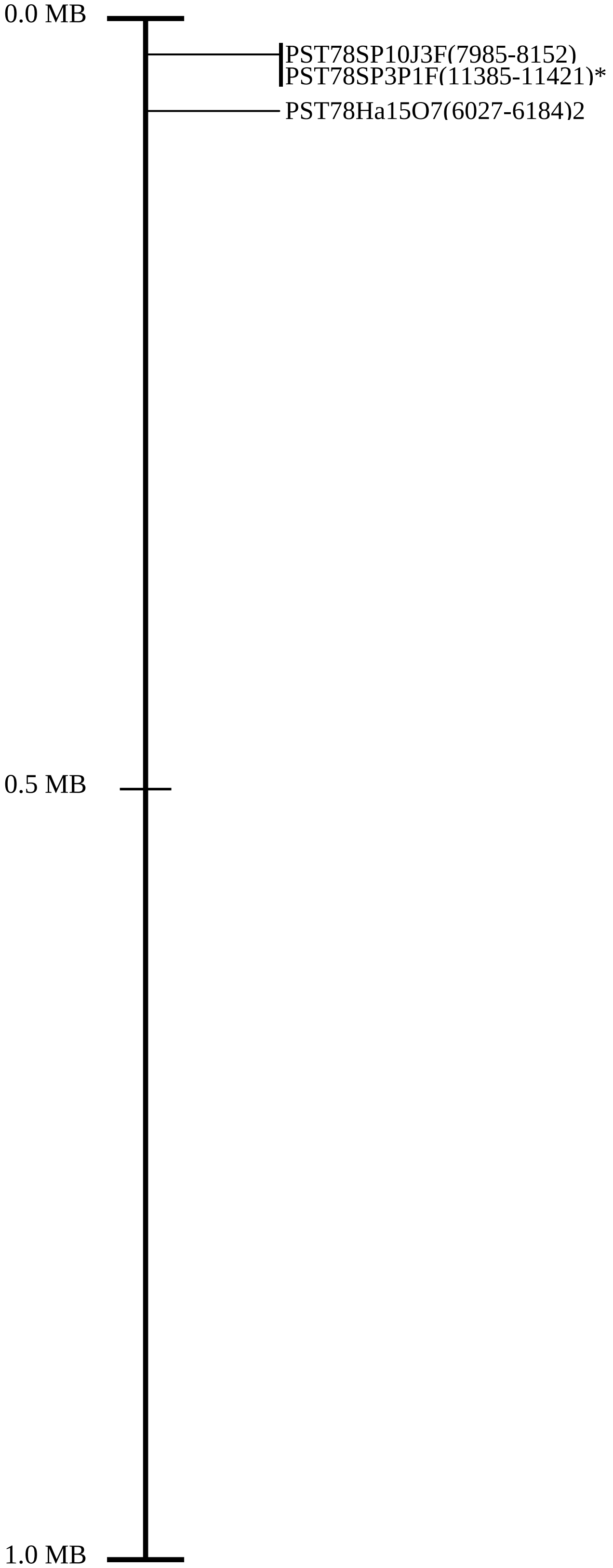

Supercontig 154
length: 59,544 bp

*** = less than 100bp alignment**

| = clones in the same contig

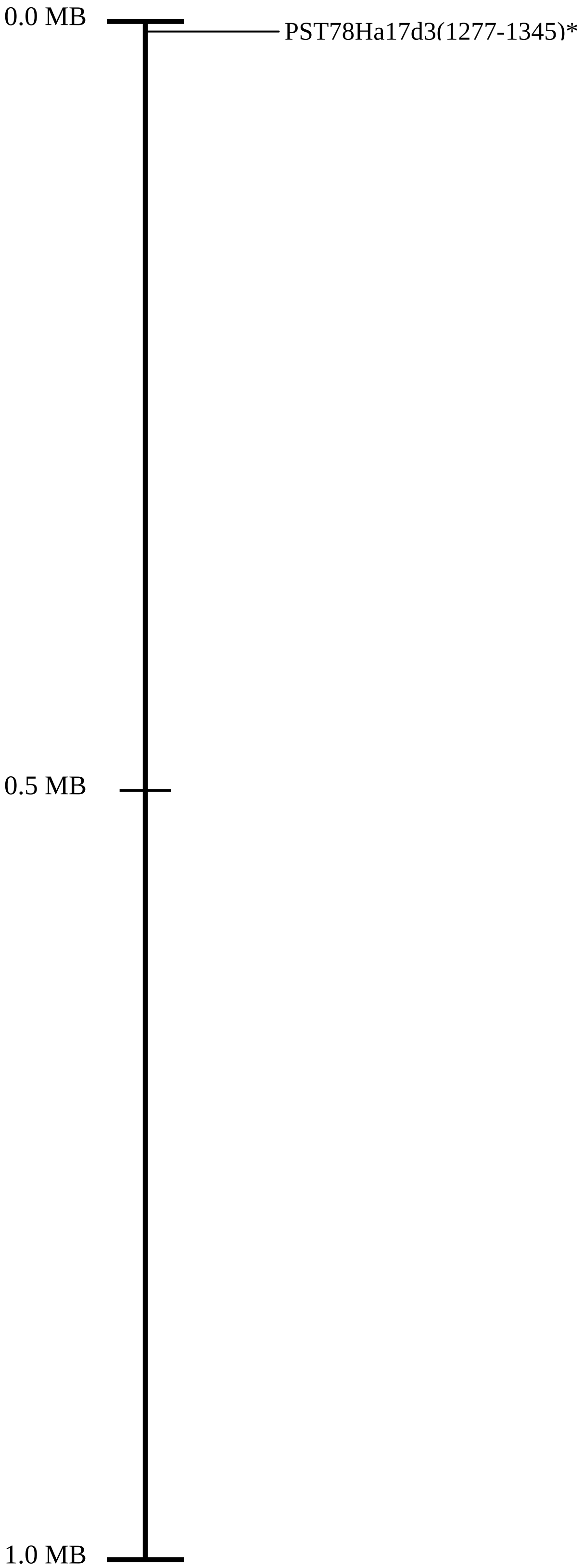

Supercontig 155
length: 57,757 bp

*** = less than 100bp alignment**

| = clones in the same contig

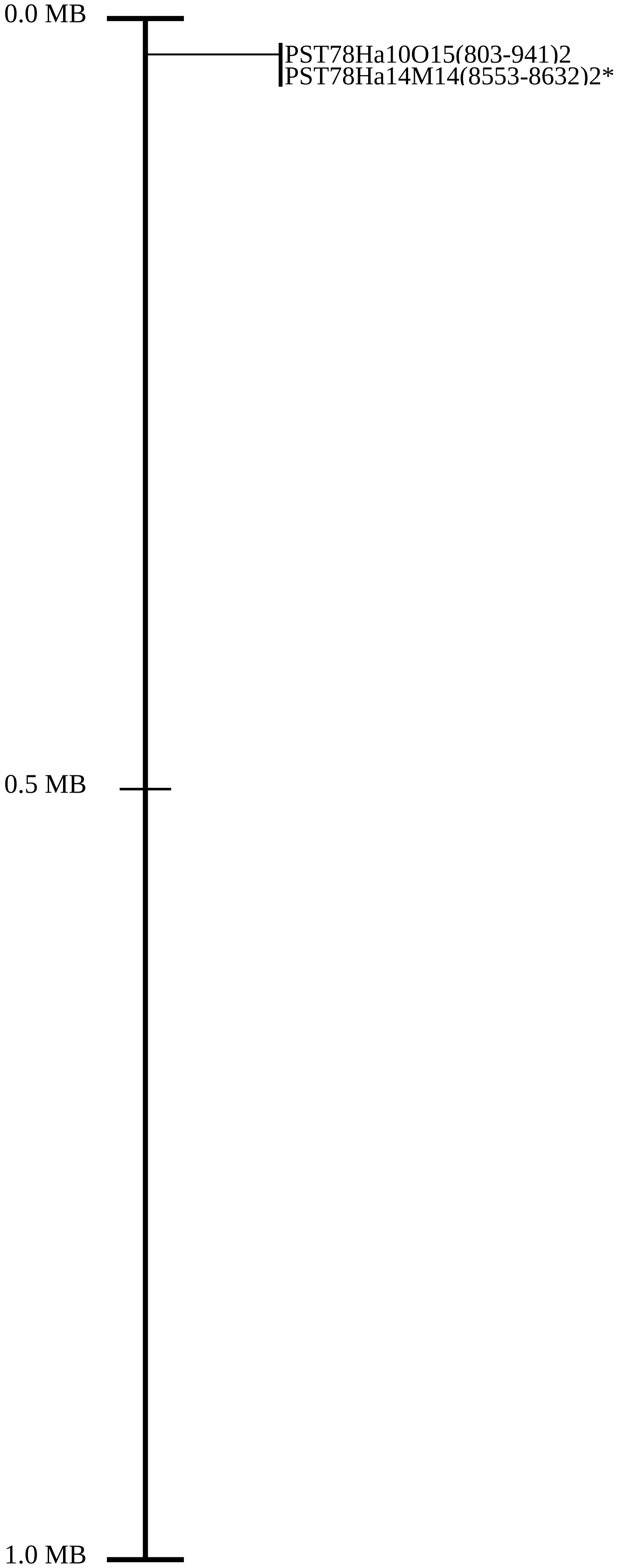

Supercontig 156
length: 63,803 bp

*** = less than 100bp alignment** **| = clones in the same contig**

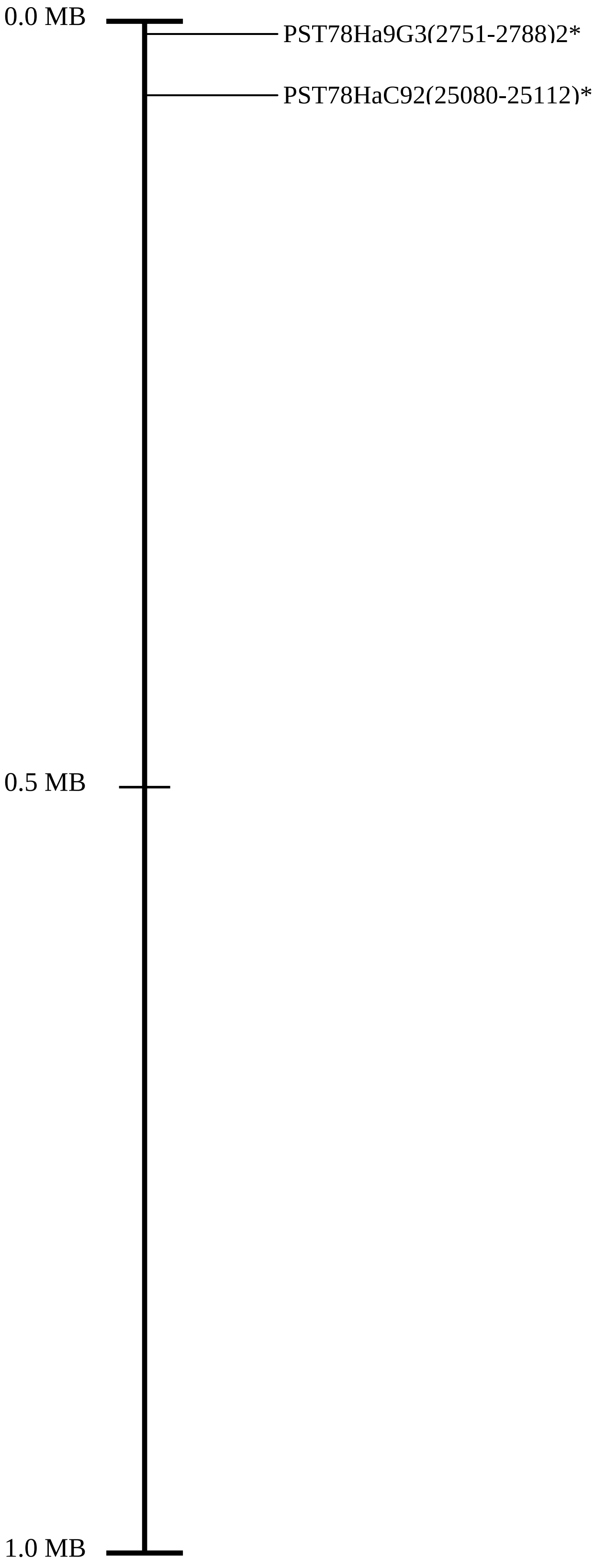

Supercontig 159
length: 48,833 bp

*** = less than 100bp alignment** **| = clones in the same contig**

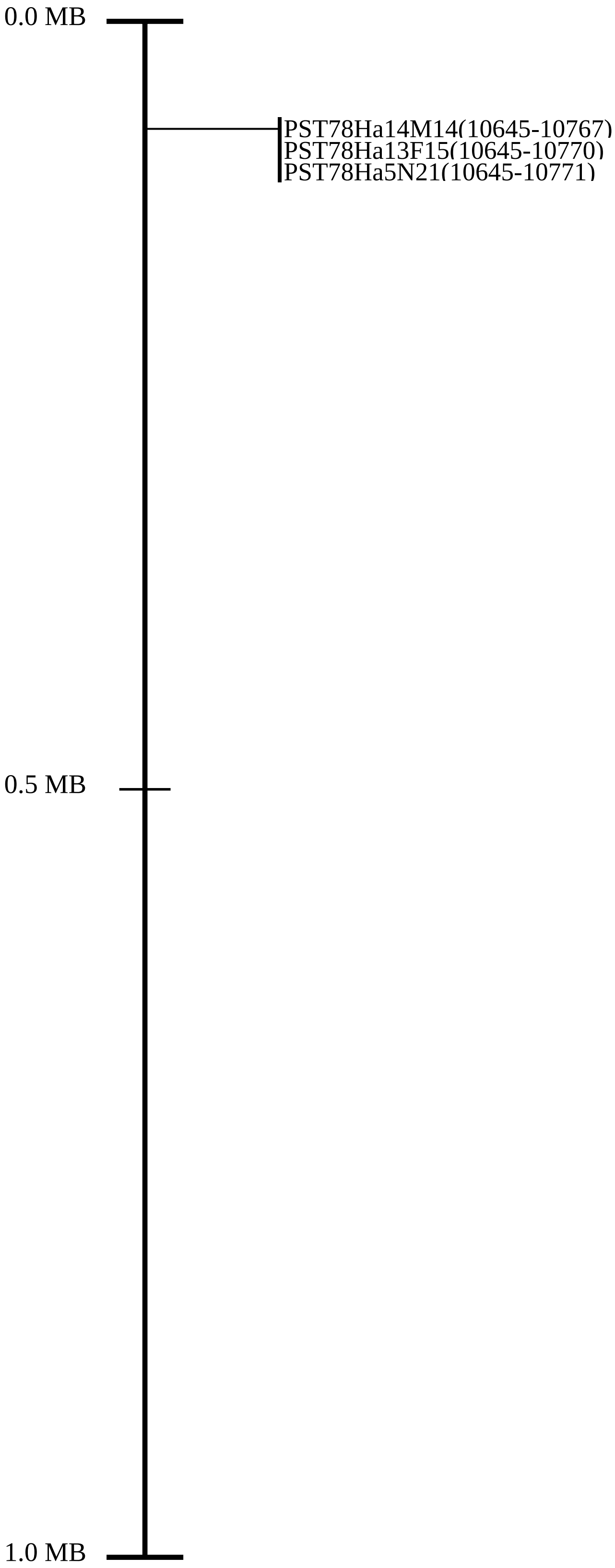

Supercontig 161
length: 70,147 bp

*** = less than 100bp alignment**   **| = clones in the same contig**

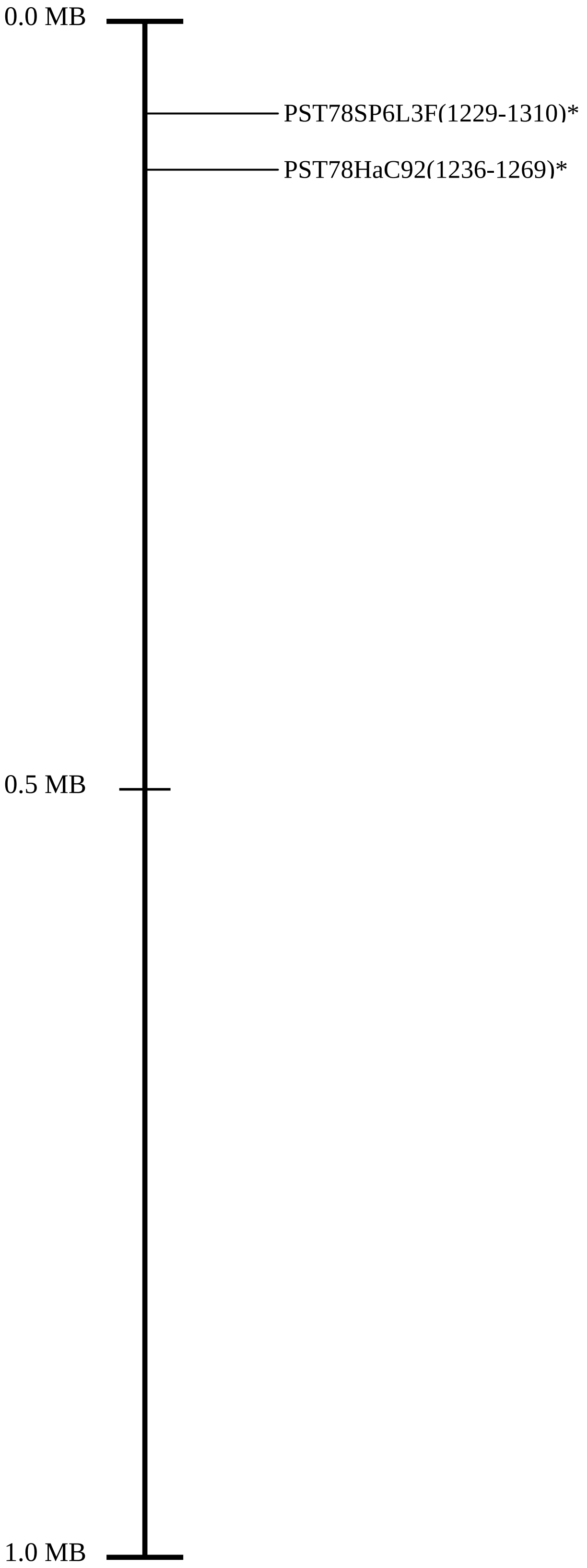

Supercontig 162
length: 96,527 bp

*** = less than 100bp alignment**

| = clones in the same contig

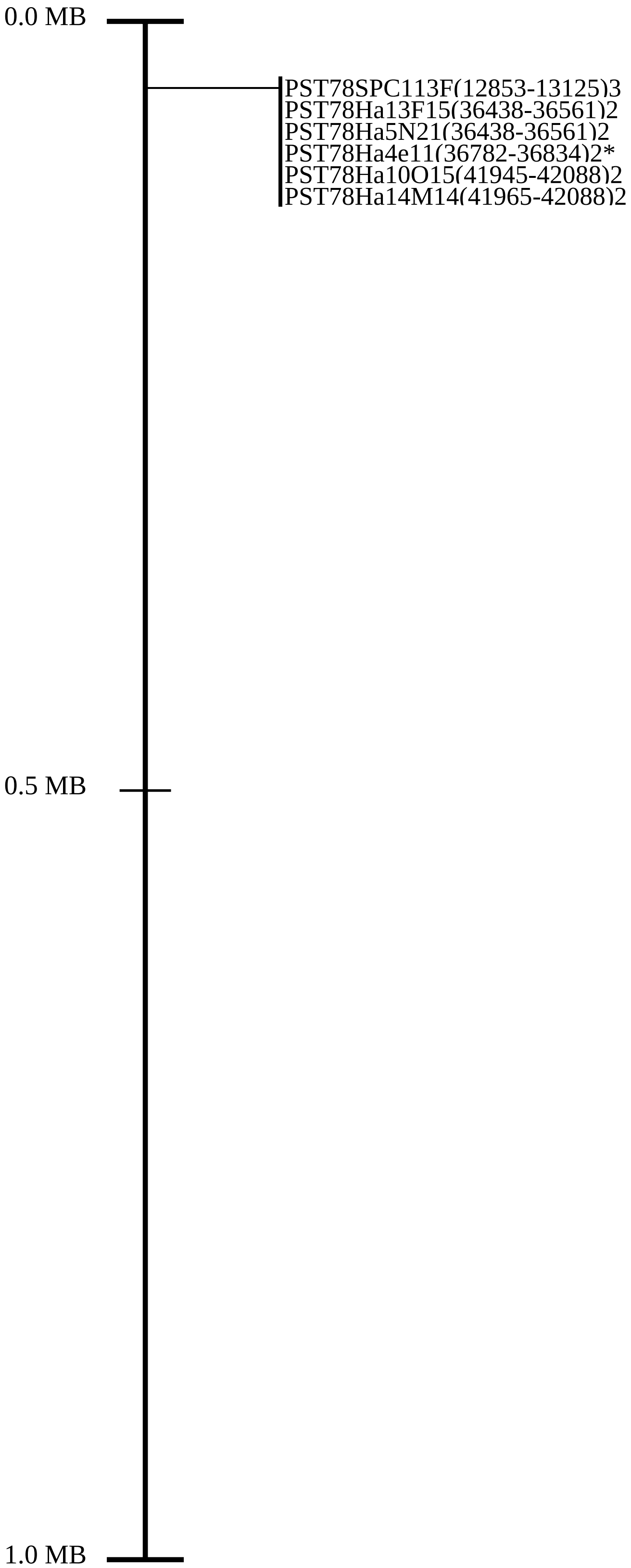

Supercontig 163
length: 42,923 bp

*** = less than 100bp alignment** **| = clones in the same contig**

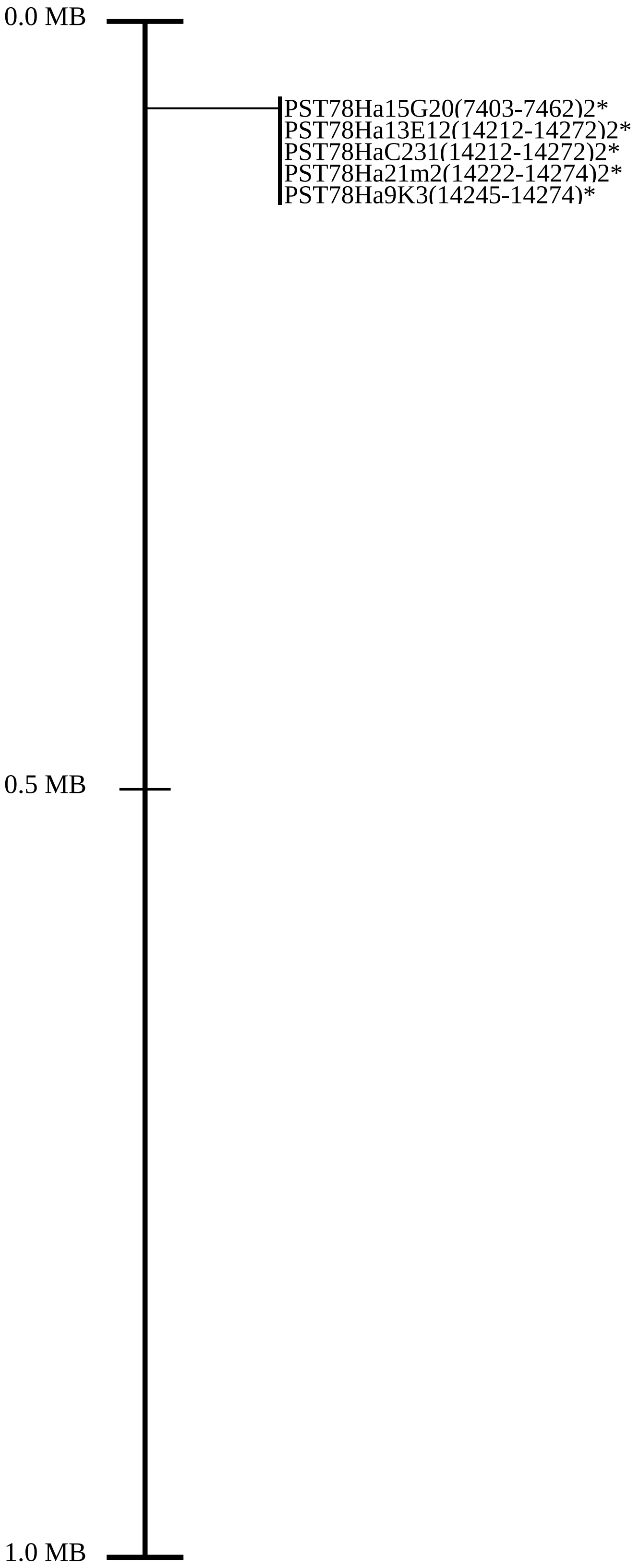

Supercontig 164
length: 56,756 bp

*** = less than 100bp alignment** **| = clones in the same contig**

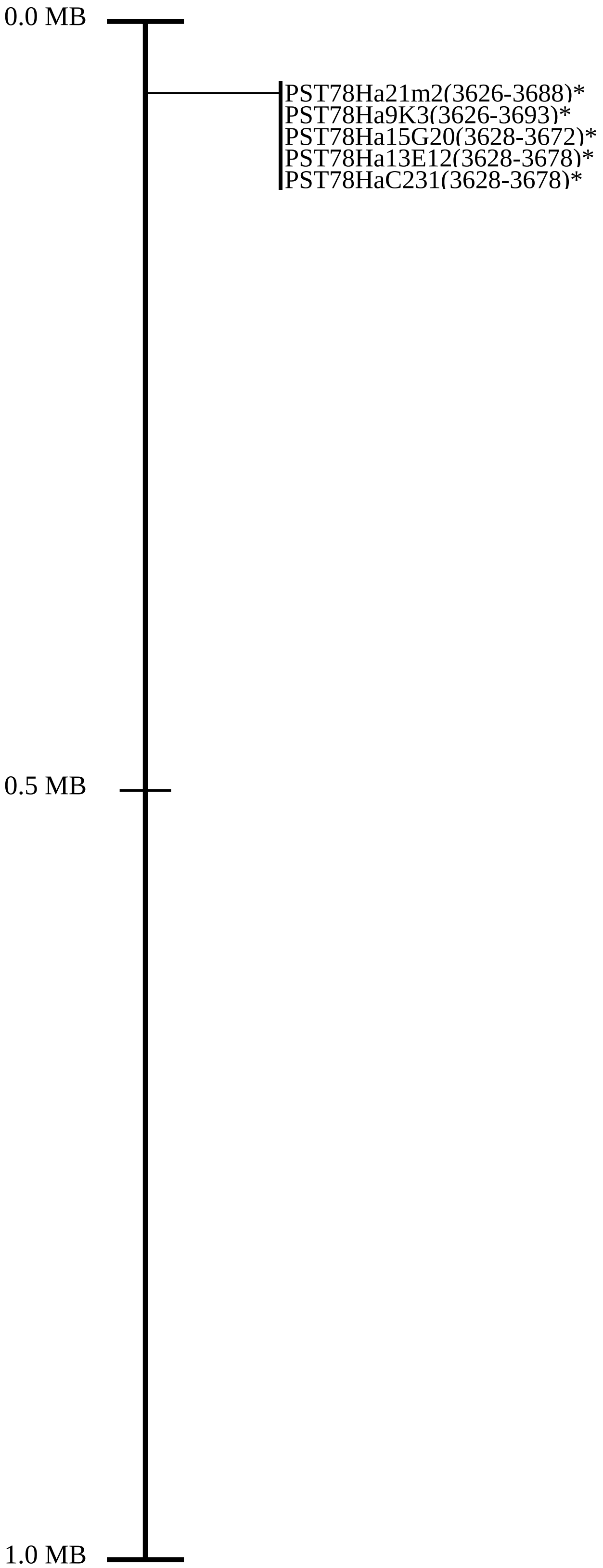

Supercontig 165
length: 46,219 bp

*** = less than 100bp alignment** **| = clones in the same contig**

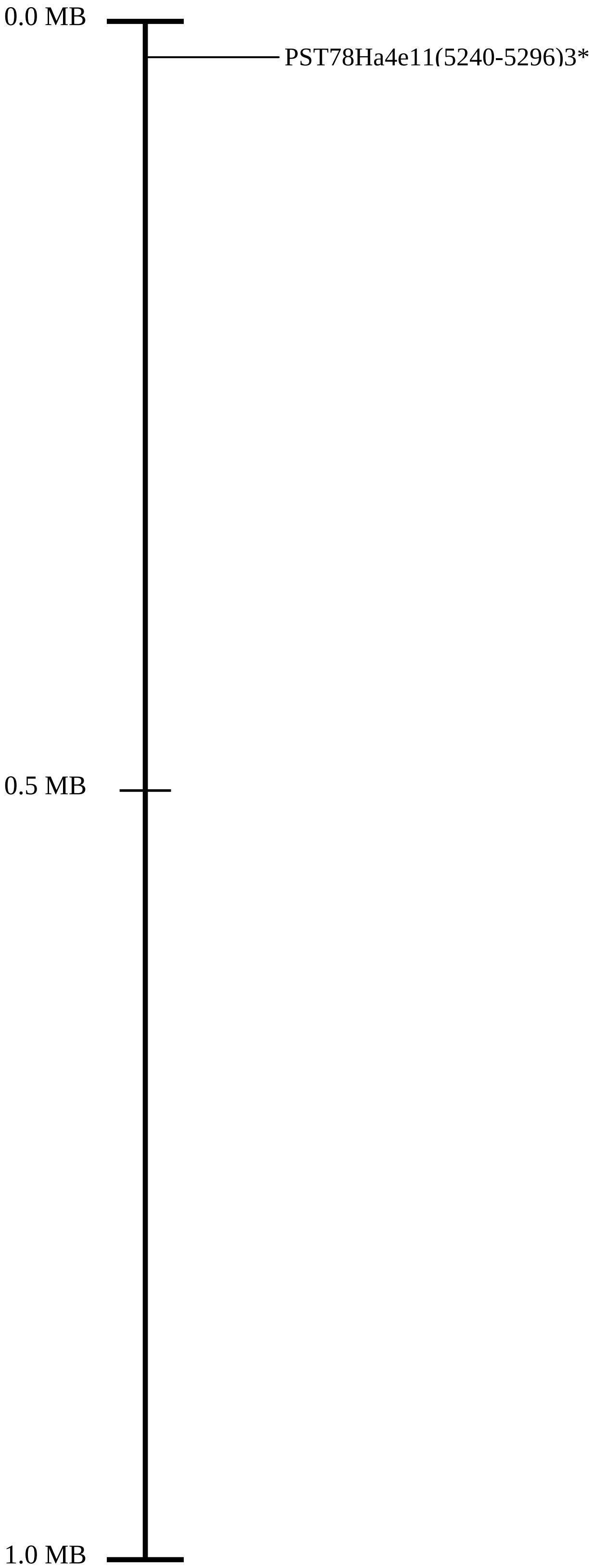

Supercontig 166
length: 62,331 bp

*** = less than 100bp alignment** **| = clones in the same contig**

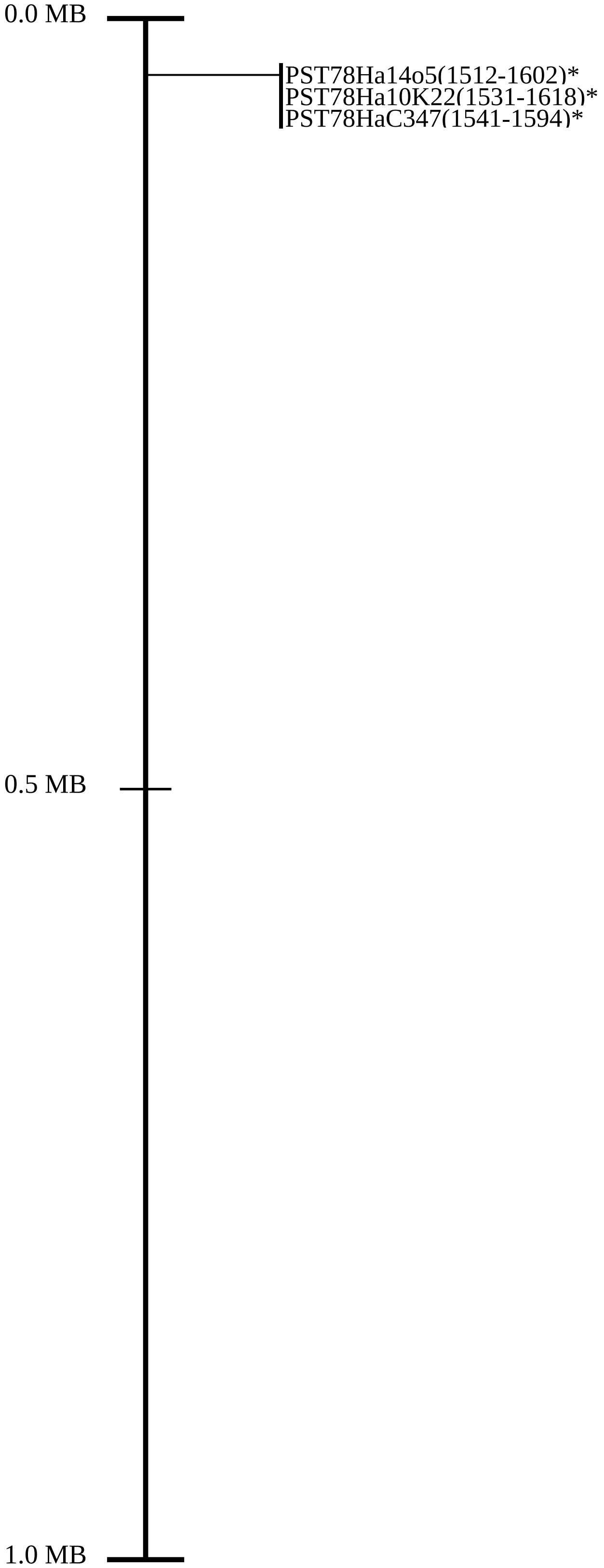

Supercontig 167
length: 55,078 bp

*** = less than 100bp alignment** **| = clones in the same contig**

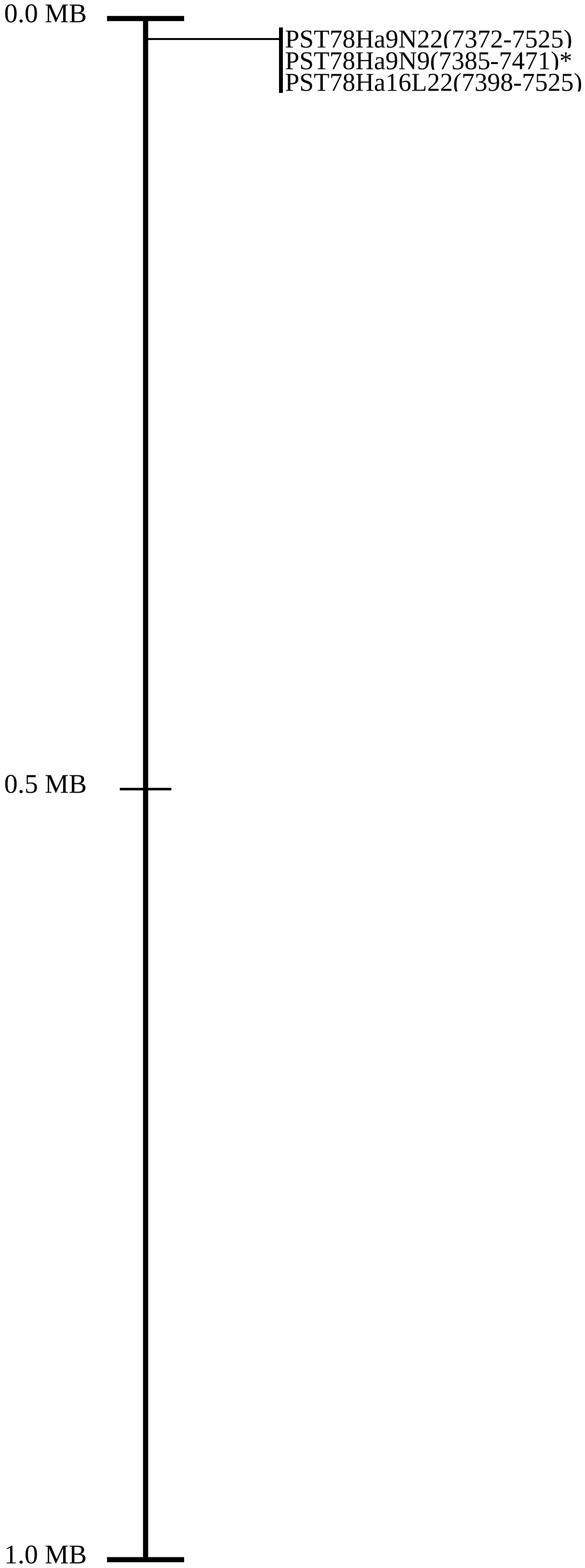

Supercontig 169
length: 56,434 bp

*** = less than 100bp alignment**

| = clones in the same contig

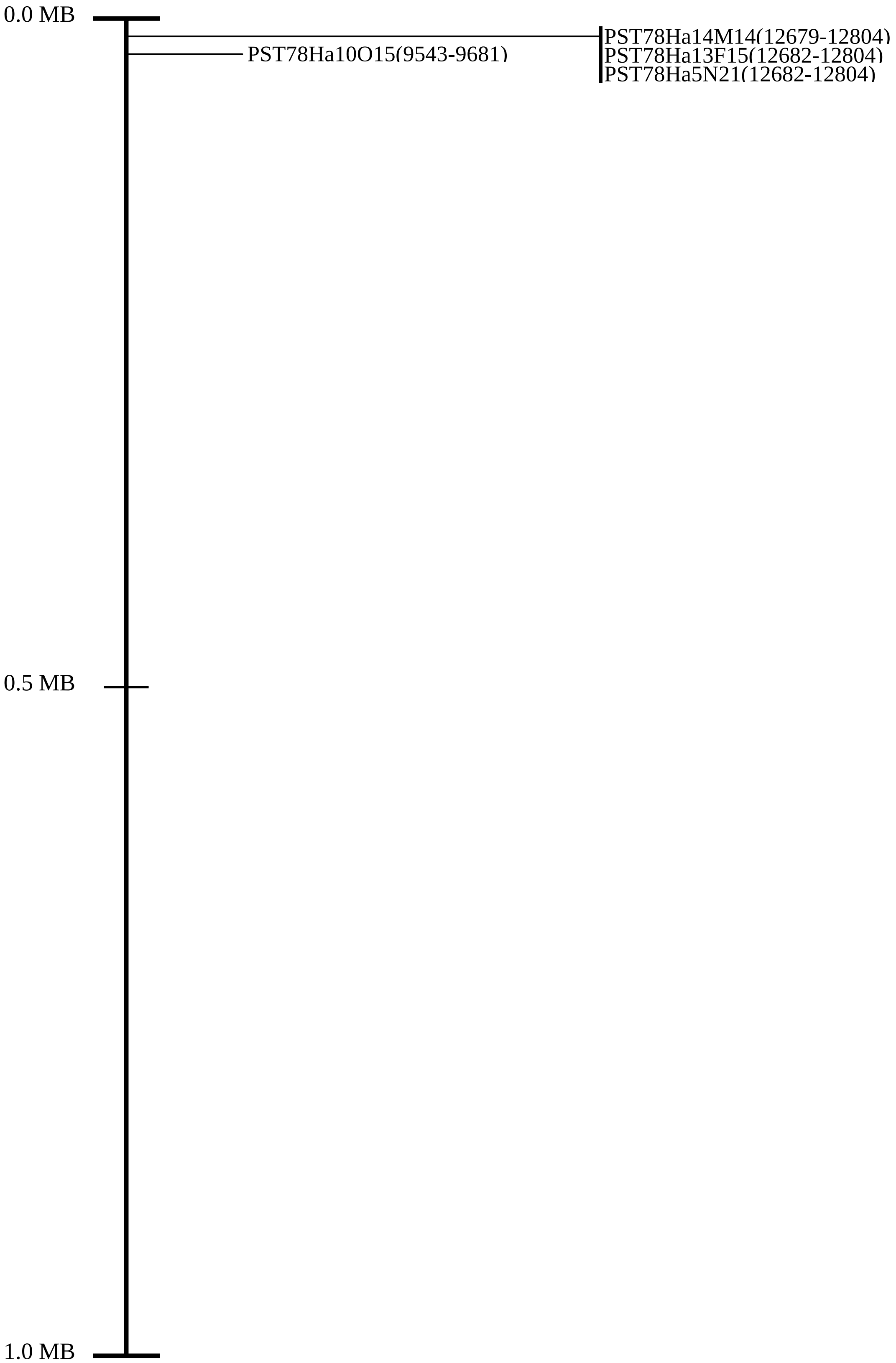

Supercontig 170
length: 41,057 bp

*** = less than 100bp alignment** **| = clones in the same contig**

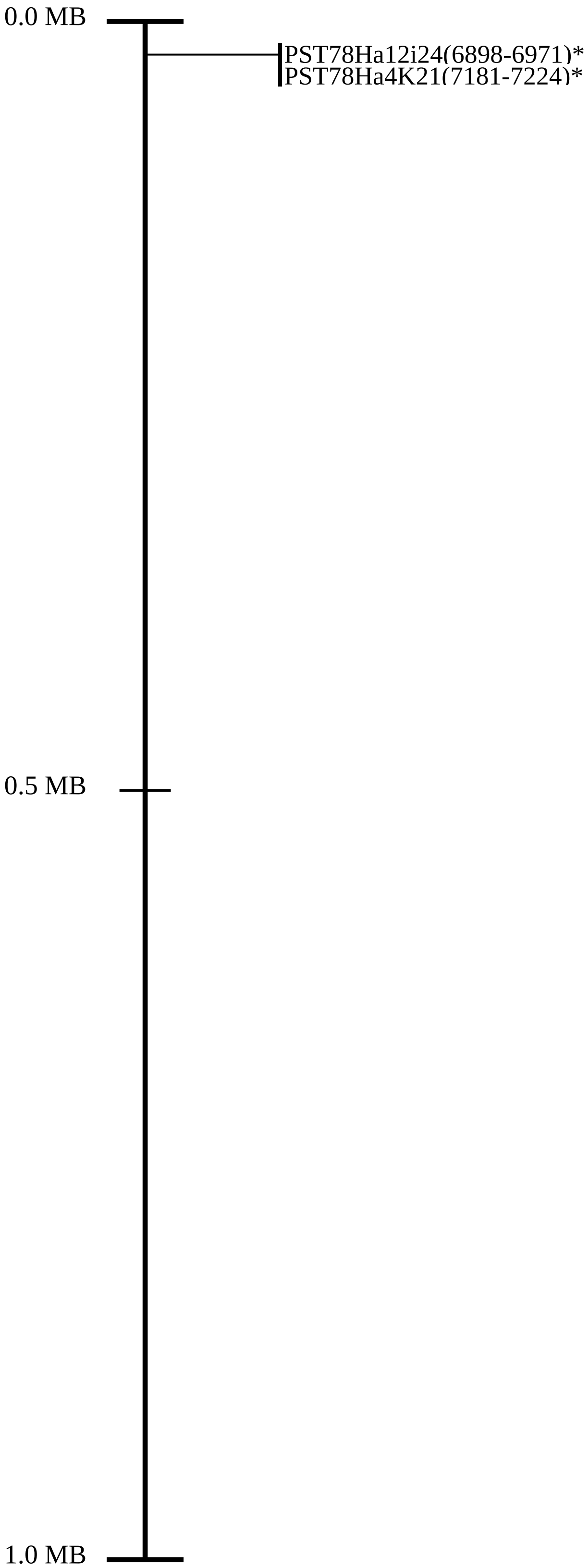

Supercontig 171
length: 47,090 bp

*** = less than 100bp alignment** **| = clones in the same contig**

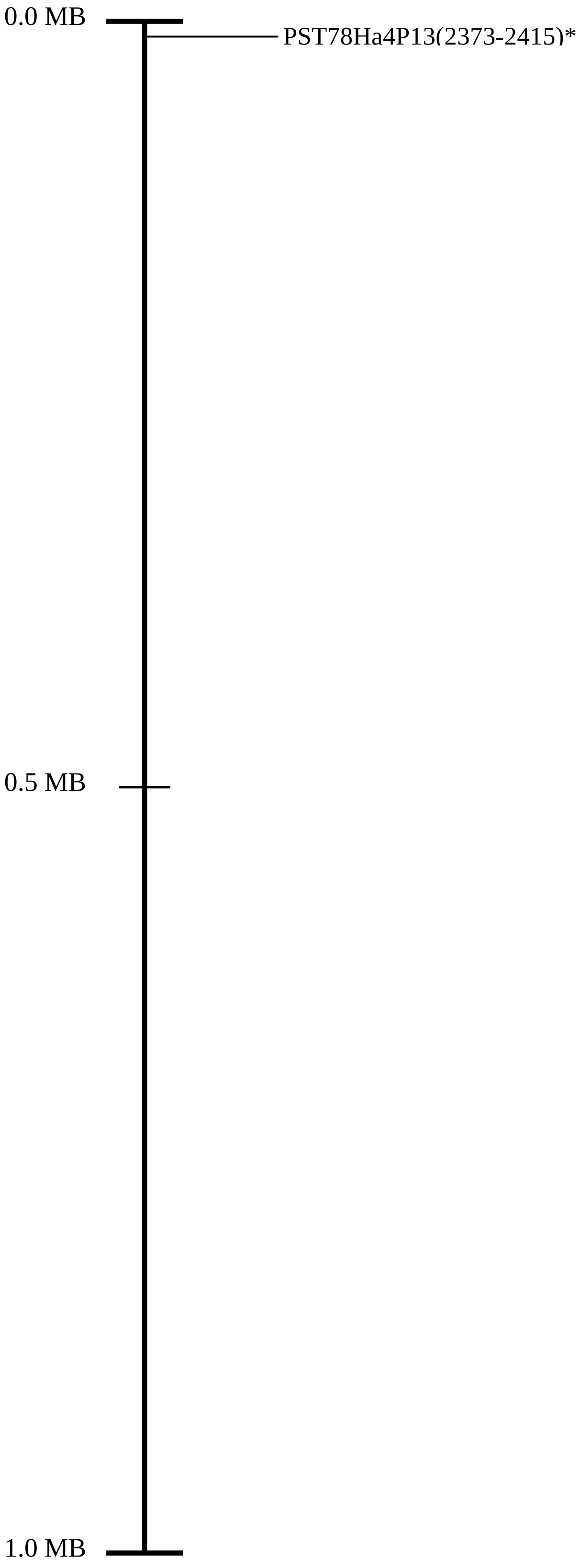

Supercontig 172
length: 51,493 bp

*** = less than 100bp alignment**

| = clones in the same contig

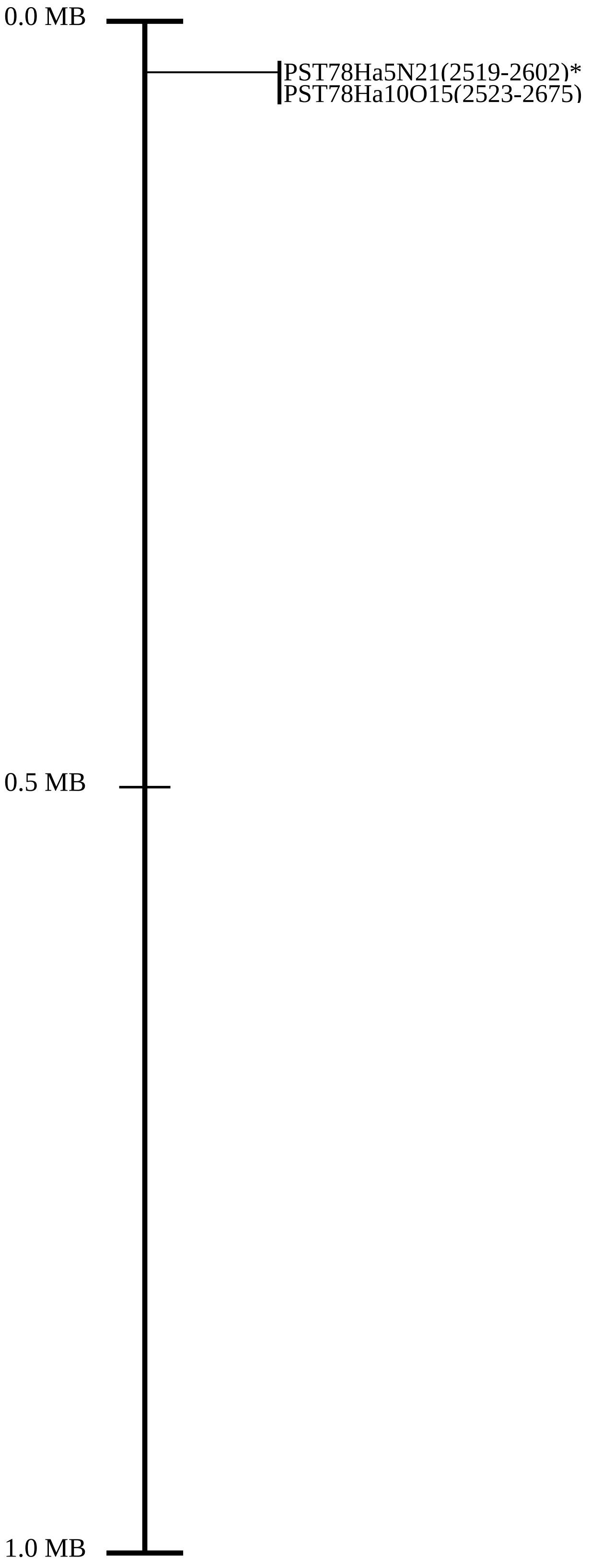

Supercontig 173
length: 32,945 bp

*** = less than 100bp alignment**

| = clones in the same contig

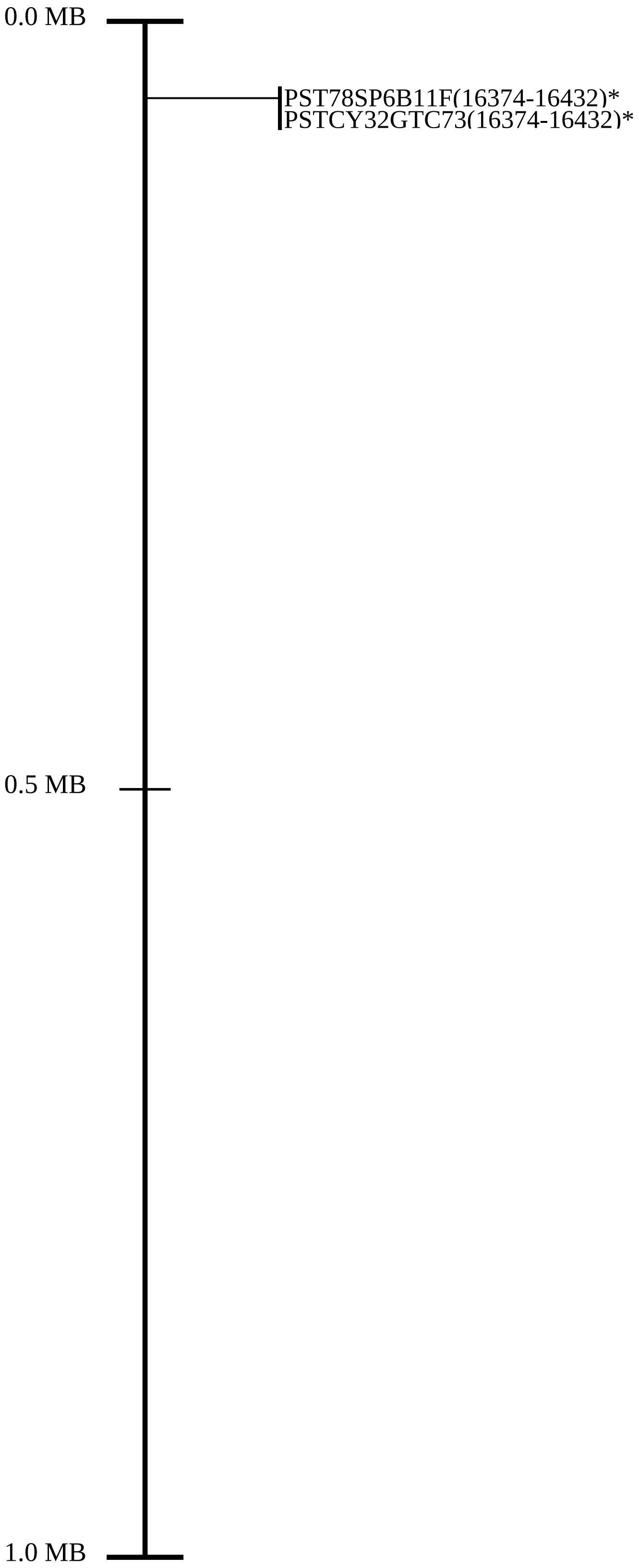

Supercontig 178
length: 30,831 bp

*** = less than 100bp alignment** **| = clones in the same contig**

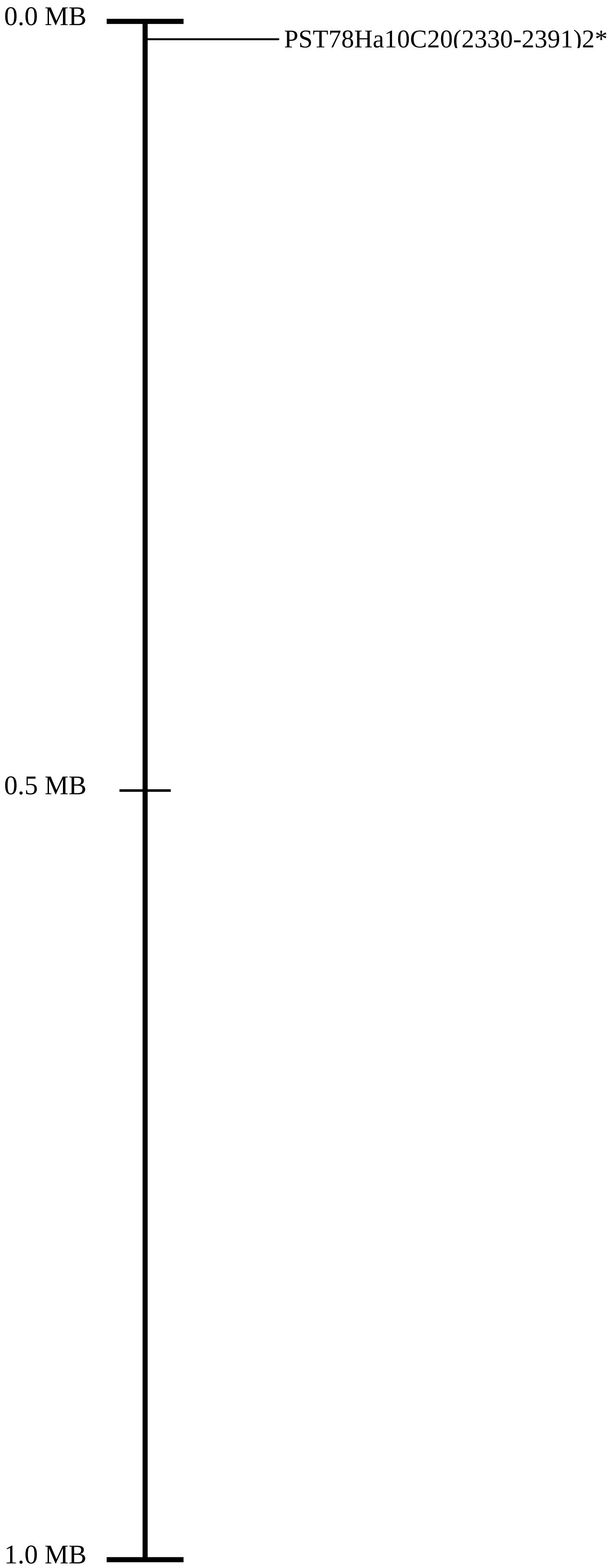

Supercontig 180
length: 26,426 bp

*** = less than 100bp alignment** **| = clones in the same contig**

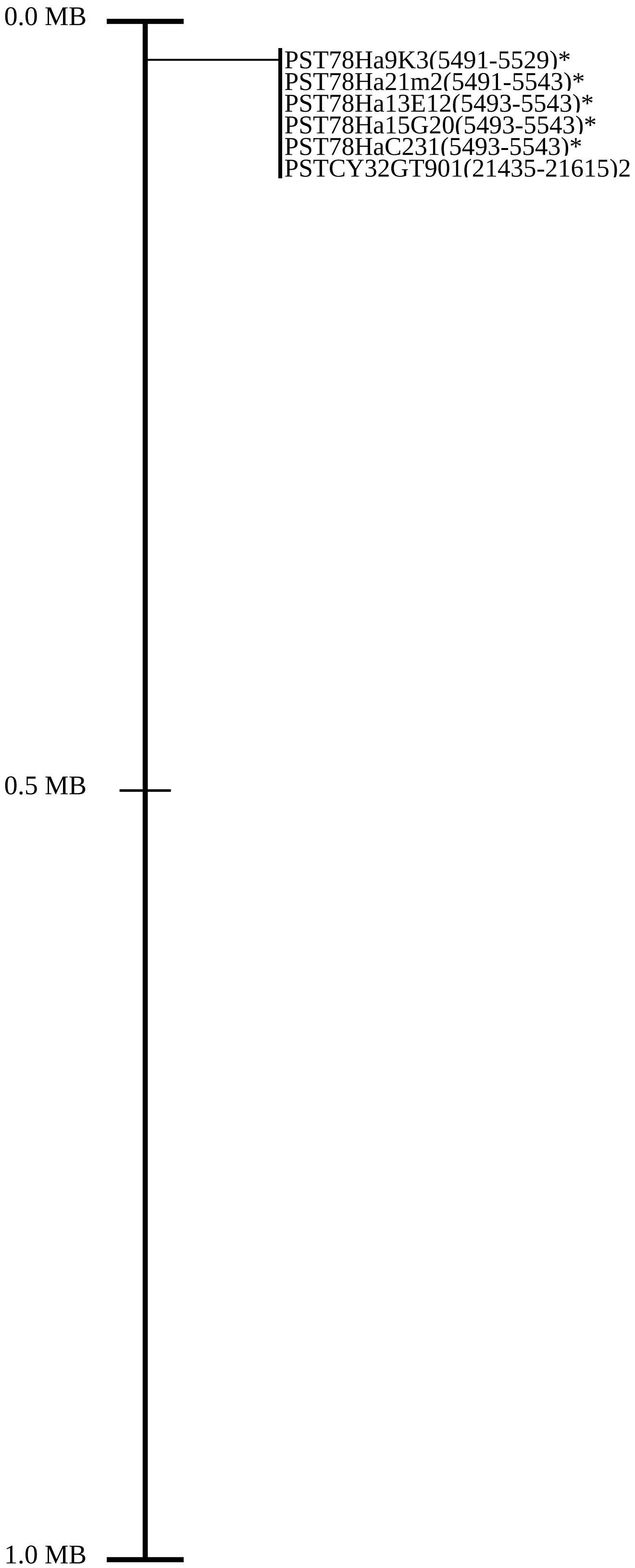

Supercontig 181
length: 25,288 bp

*** = less than 100bp alignment** **| = clones in the same contig**

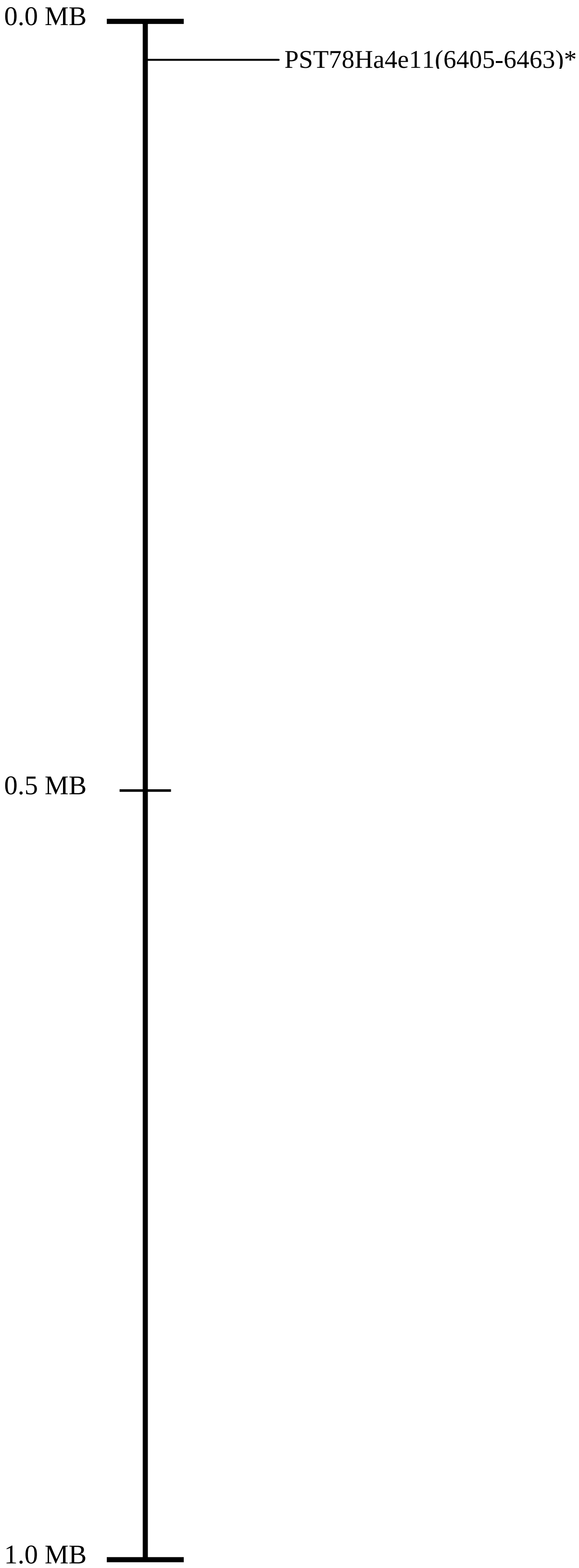

Supercontig 183
length: 25,255 bp

*** = less than 100bp alignment**

| = clones in the same contig

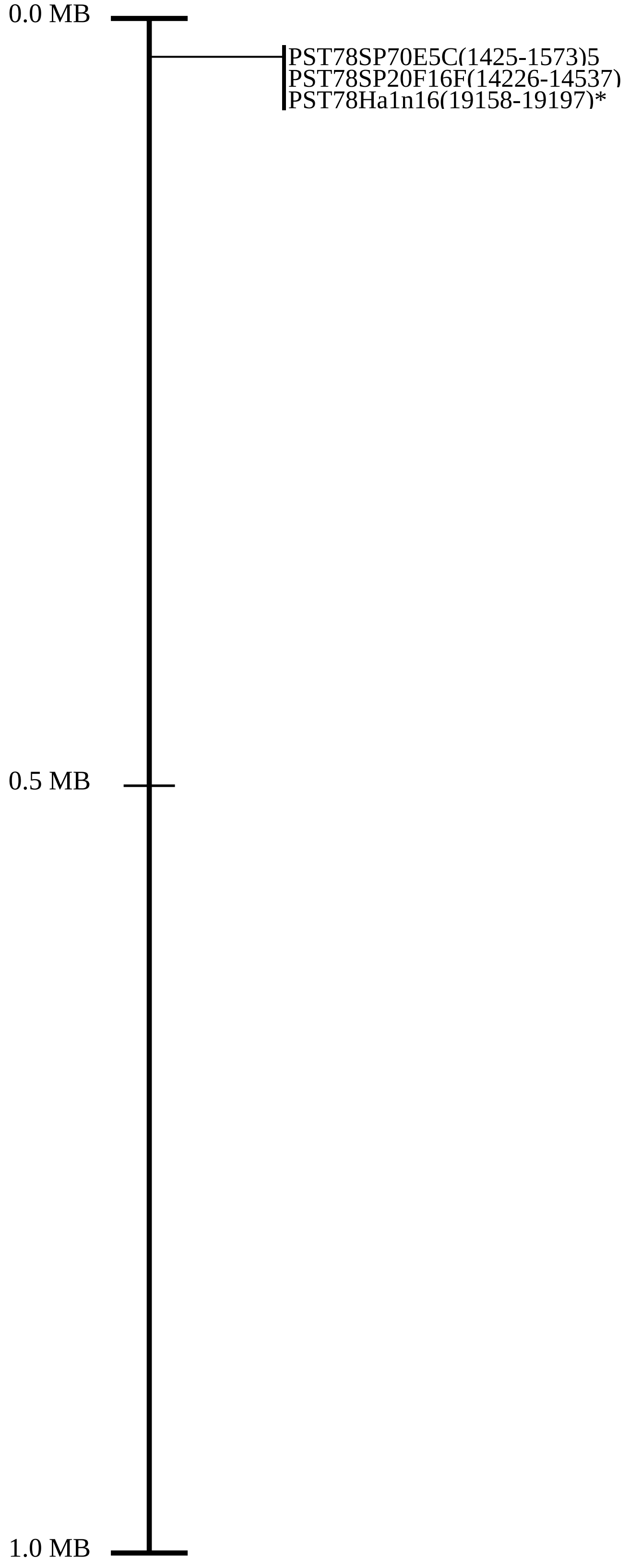

Supercontig 184
length: 25,182 bp

*** = less than 100bp alignment**

| = clones in the same contig

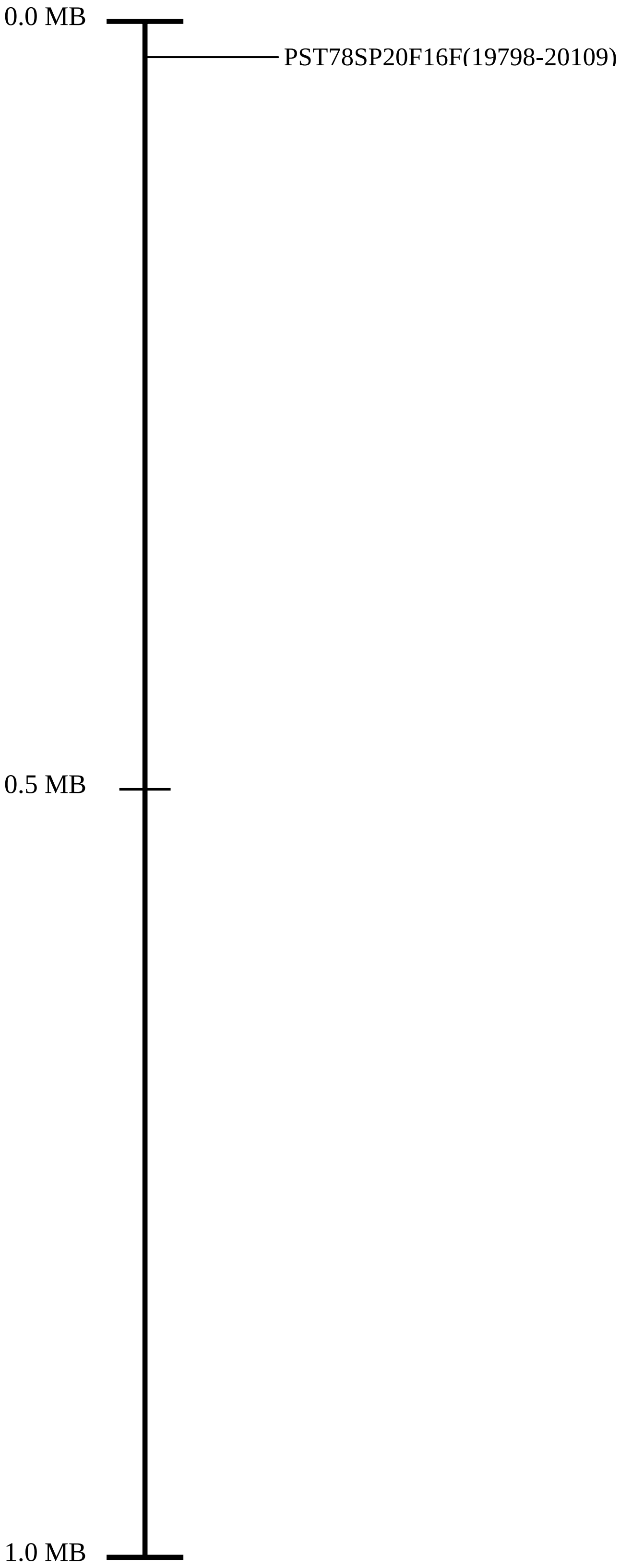

Supercontig 189
length: 22,821 bp

*** = less than 100bp alignment**

| = clones in the same contig

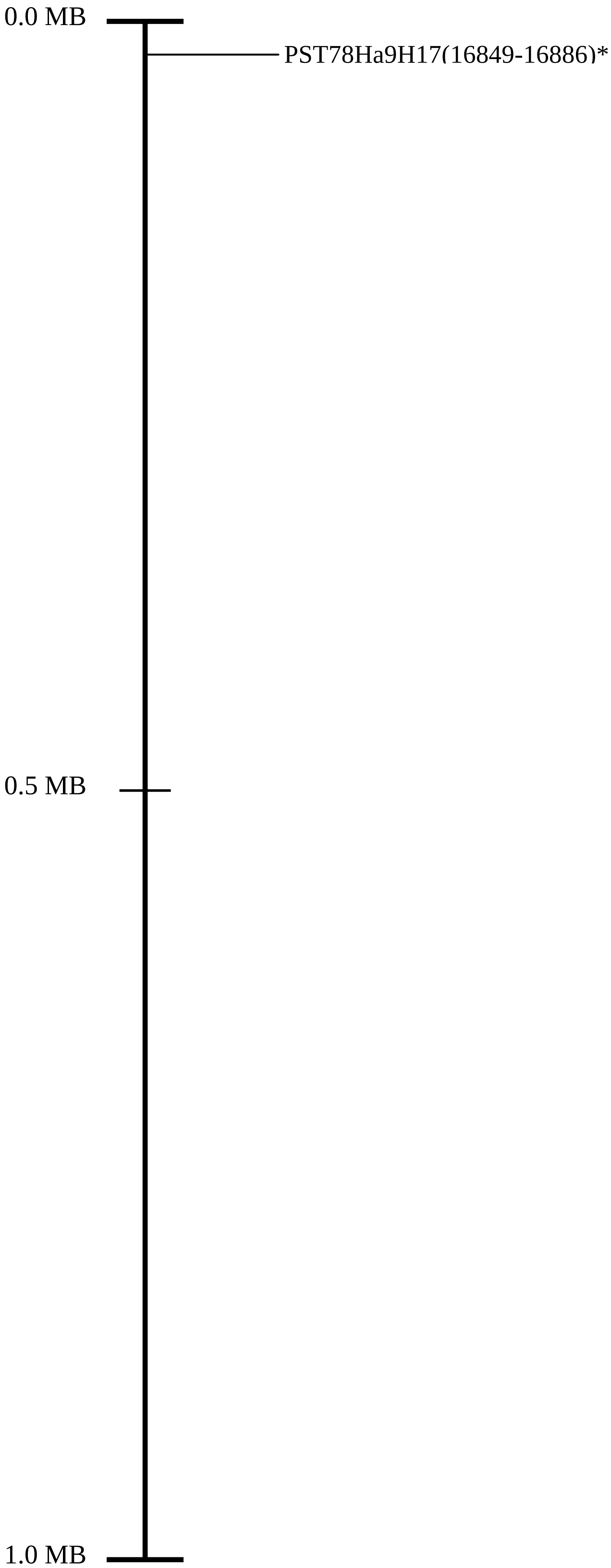

Supercontig 191
length: 21,049 bp

*** = less than 100bp alignment**

| = clones in the same contig

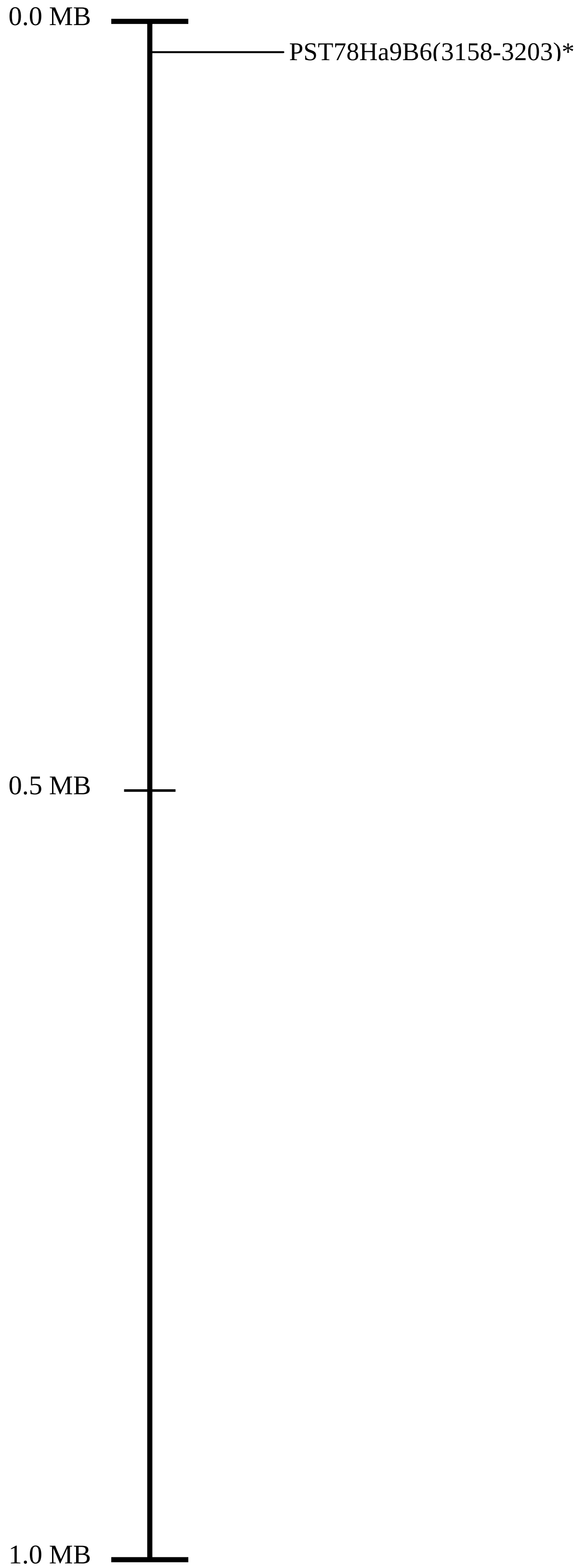

Supercontig 193
length: 20,340 bp

*** = less than 100bp alignment**

| = clones in the same contig

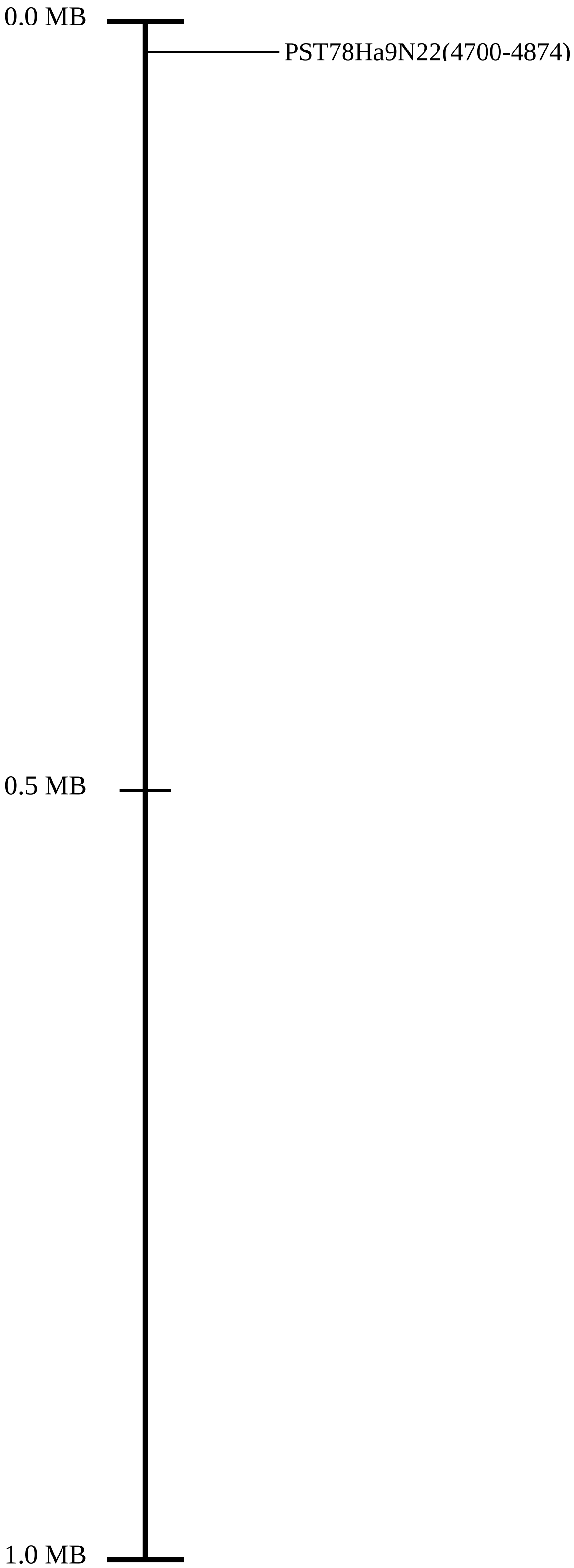

Supercontig 195
length: 20,131 bp

*** = less than 100bp alignment**

| = clones in the same contig

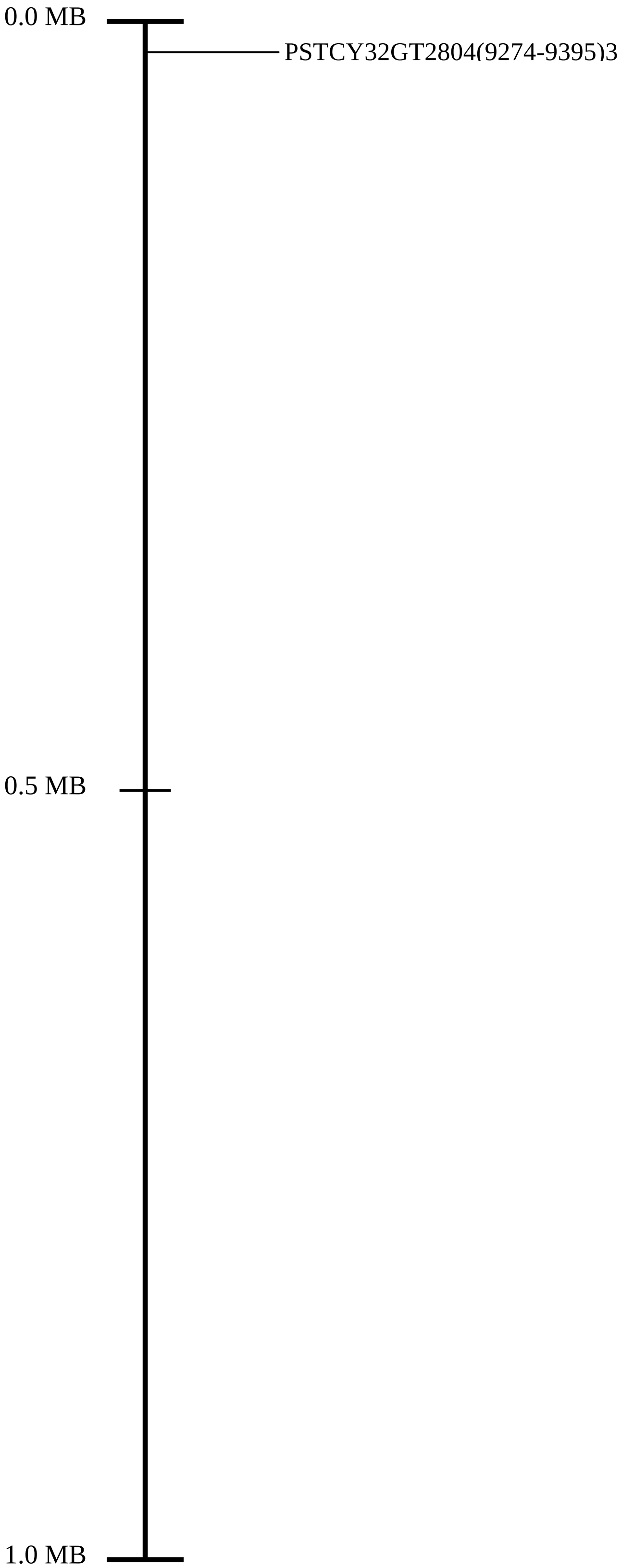

Supercontig 197
length: 19,986 bp

*** = less than 100bp alignment** **| = clones in the same contig**

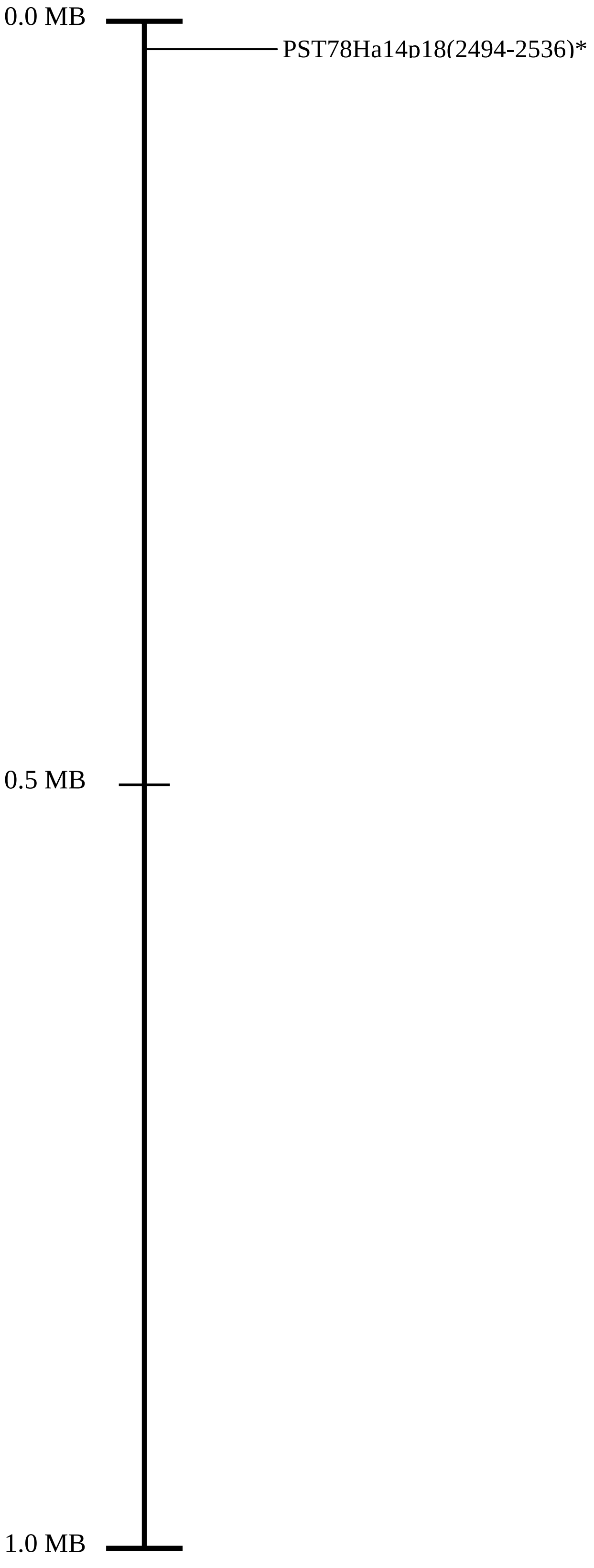

Supercontig 202
length: 18,478 bp

*** = less than 100bp alignment**

| = clones in the same contig

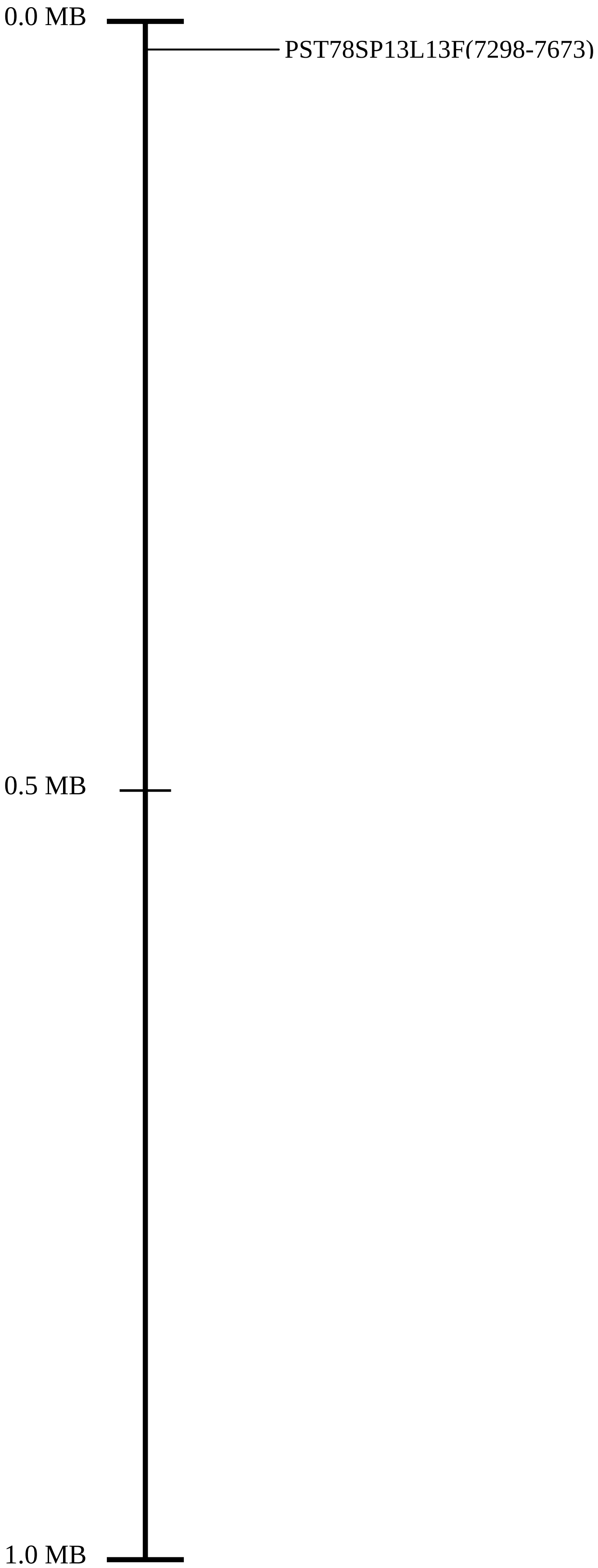

Supercontig 203
length: 18,097 bp

*** = less than 100bp alignment** **| = clones in the same contig**

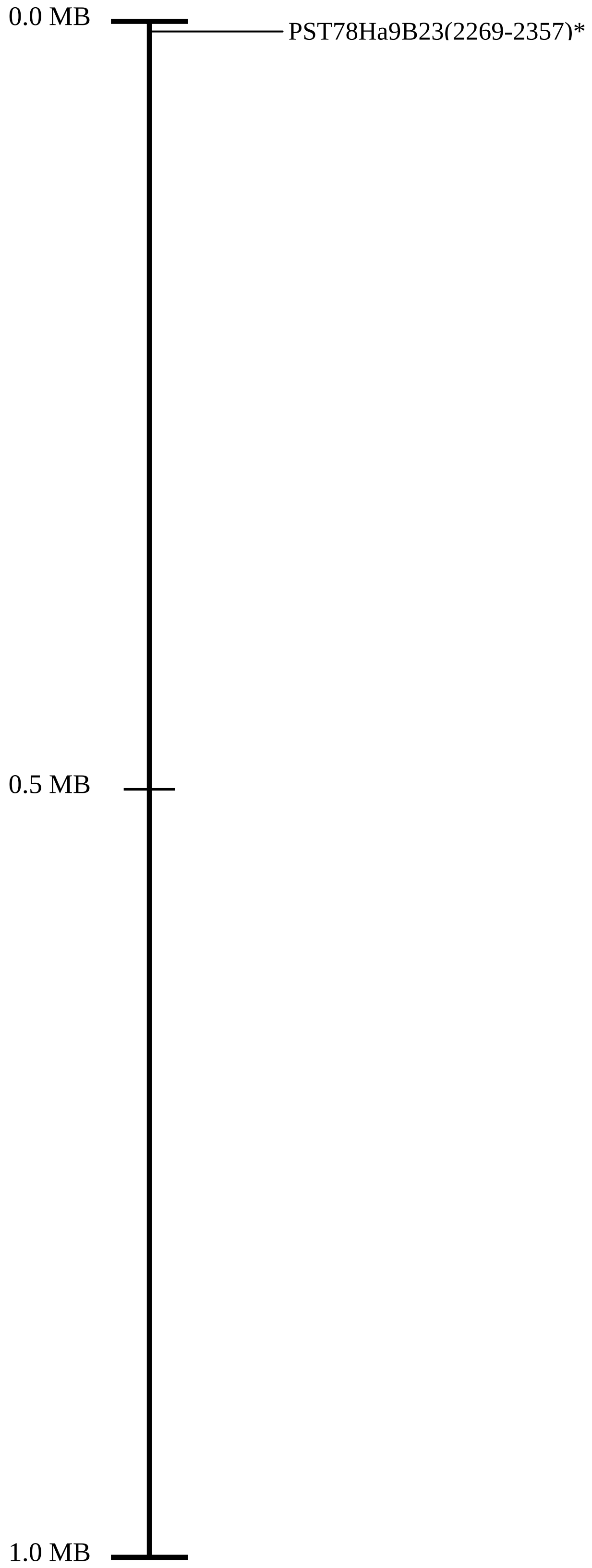

Supercontig 204
length: 19,139 bp

*** = less than 100bp alignment**

| = clones in the same contig

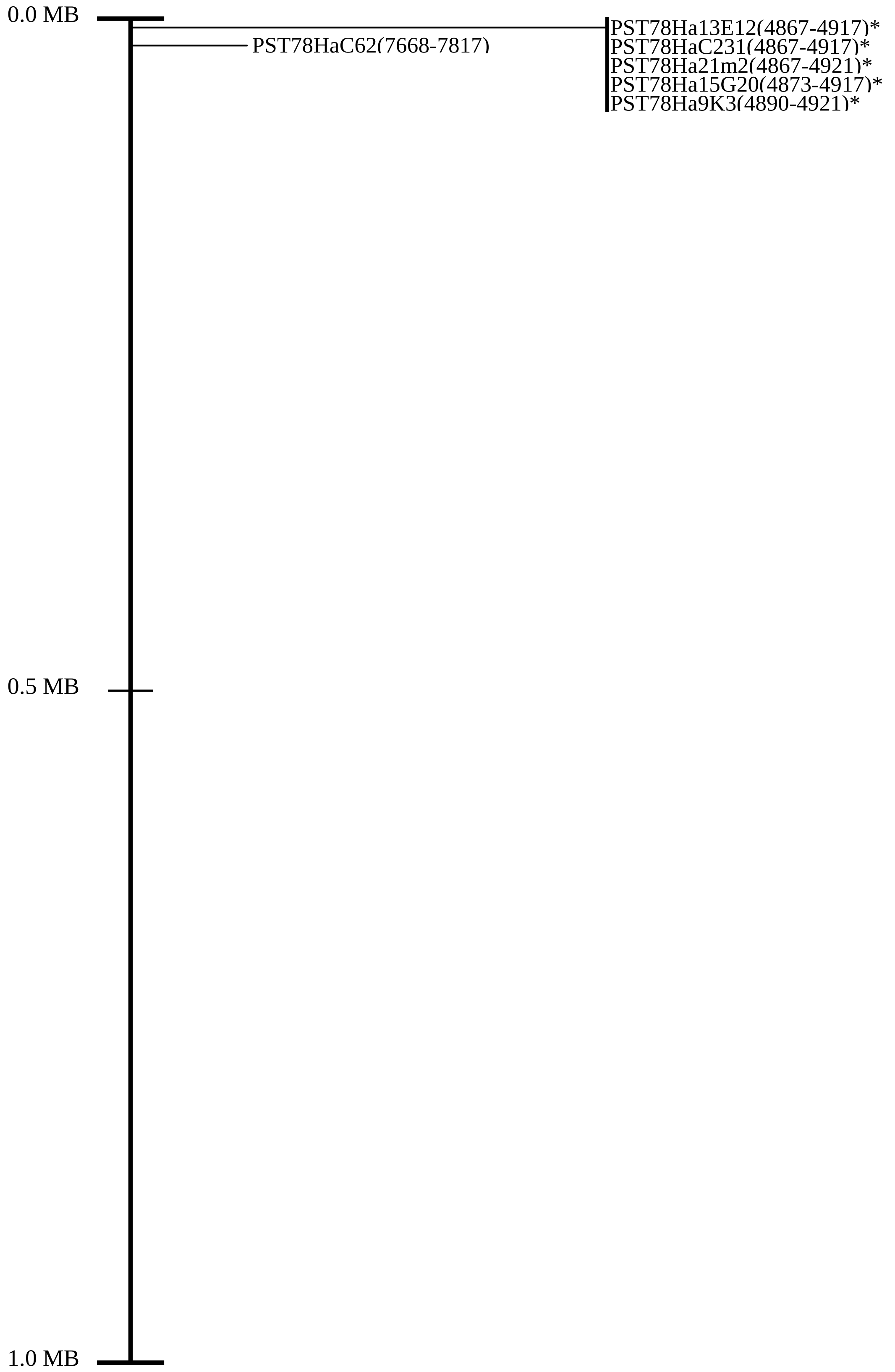

Supercontig 205
length: 19,822 bp

*** = less than 100bp alignment** **| = clones in the same contig**

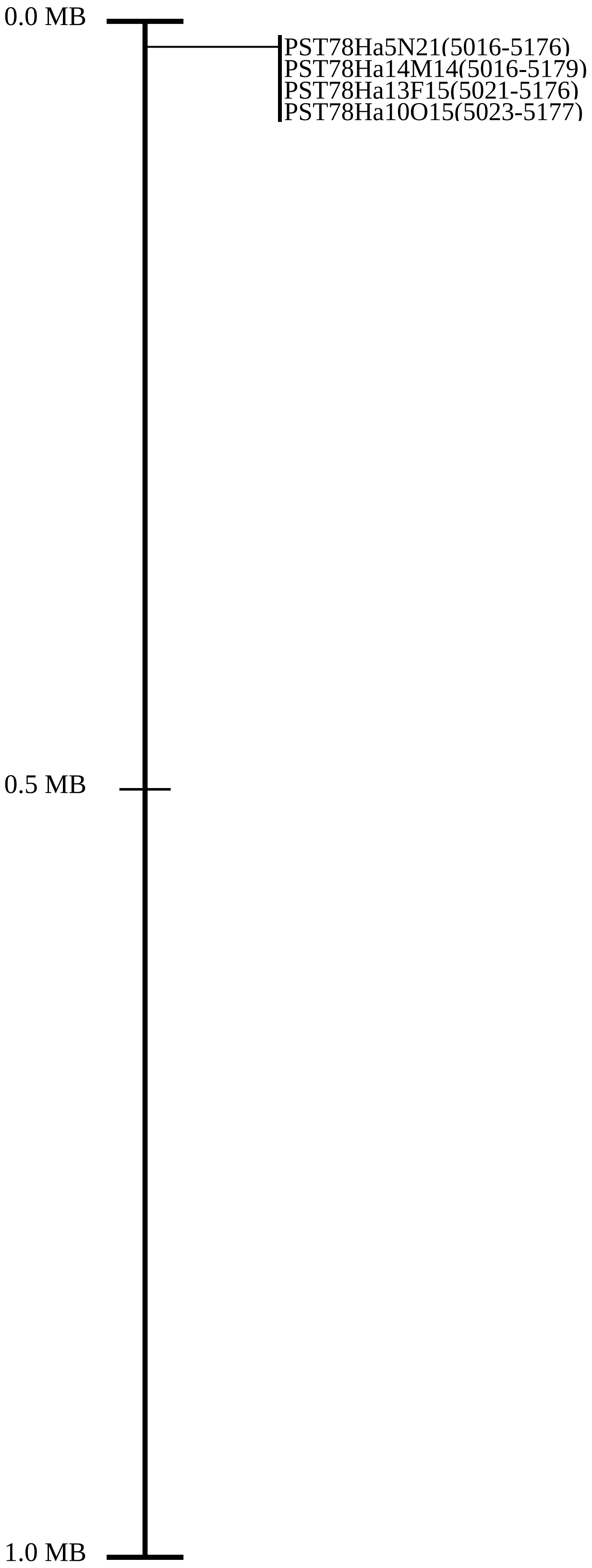

Supercontig 207
length: 17,451 bp

*** = less than 100bp alignment**

| = clones in the same contig

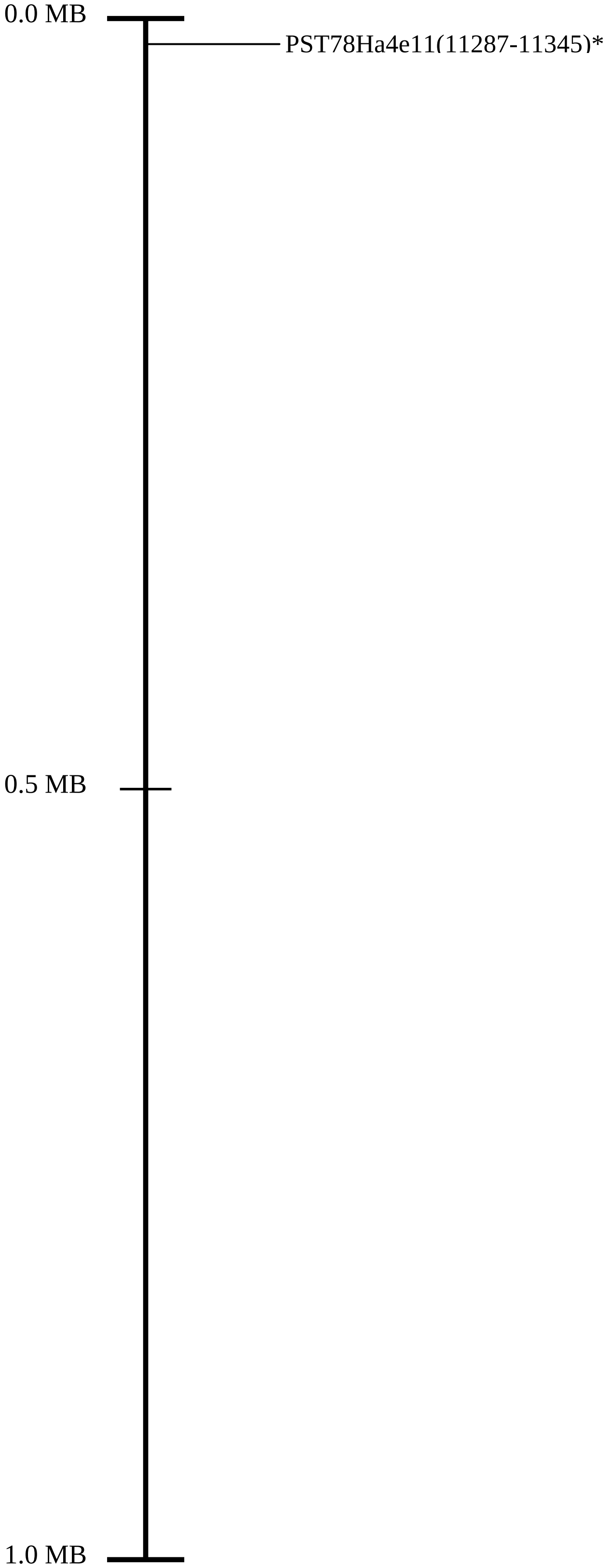

Supercontig 209
length: 16,612 bp

*** = less than 100bp alignment**

| = clones in the same contig

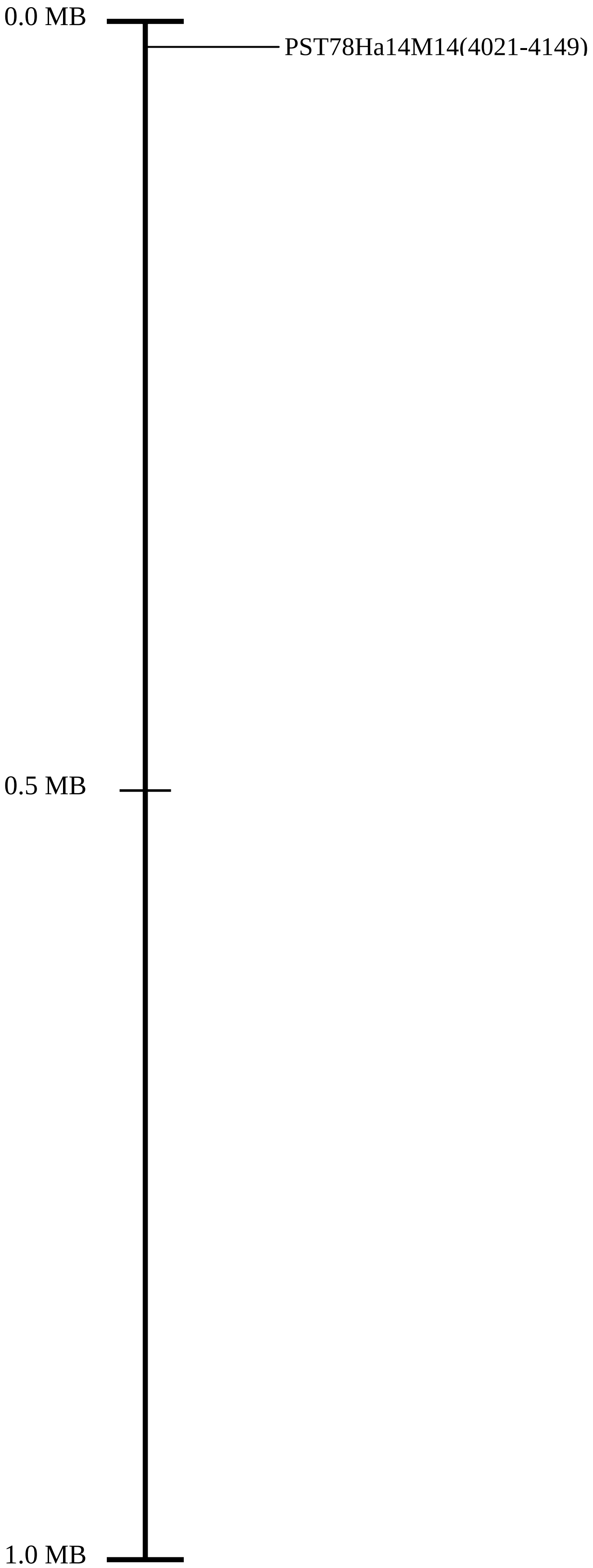

Supercontig 210
length: 16,297 bp

*** = less than 100bp alignment** **| = clones in the same contig**

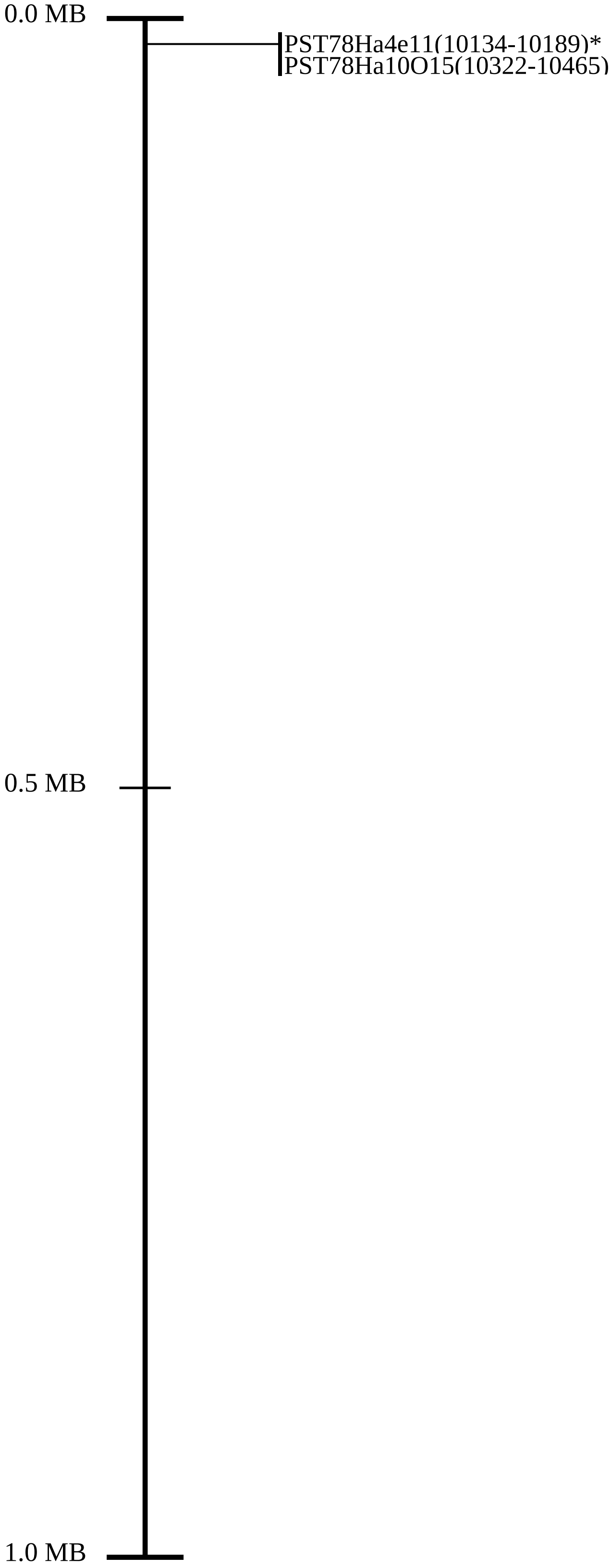

Supercontig 213
length: 15,902 bp

*** = less than 100bp alignment**

| = clones in the same contig

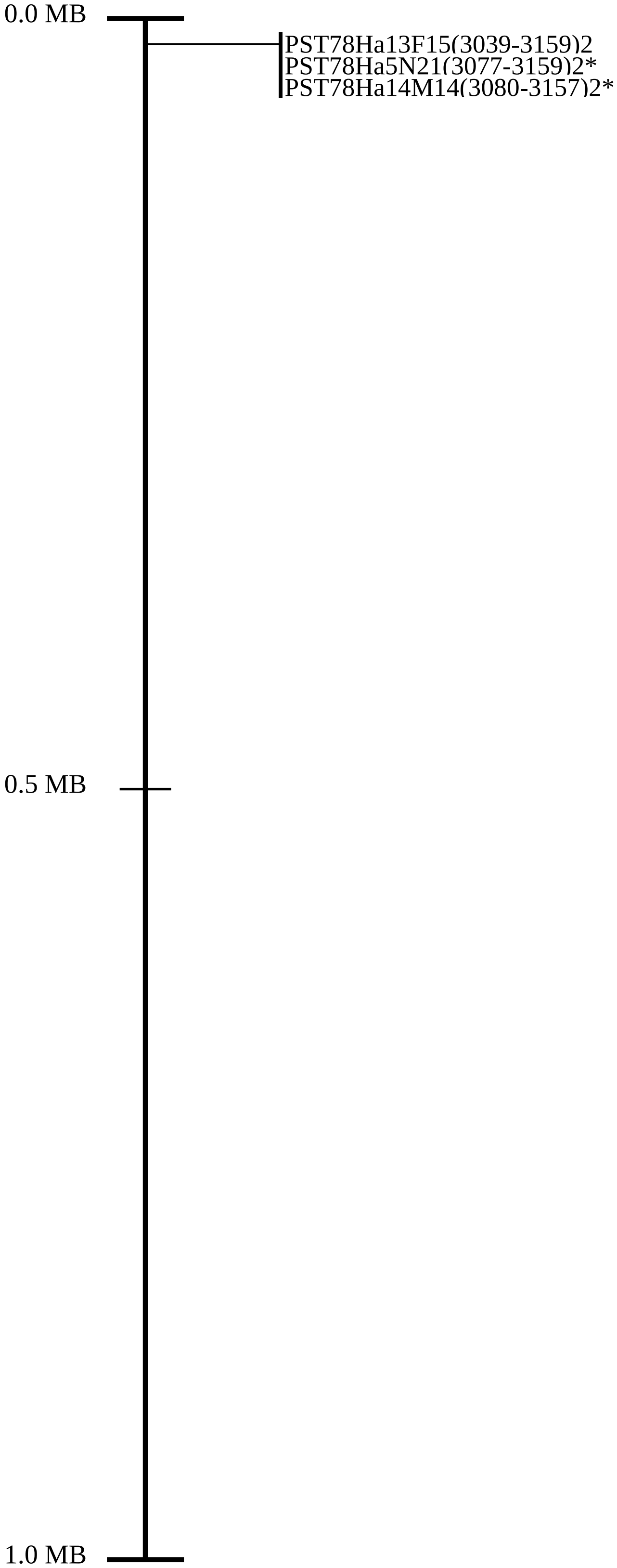

Supercontig 214
length: 16,066 bp

*** = less than 100bp alignment**

| = clones in the same contig

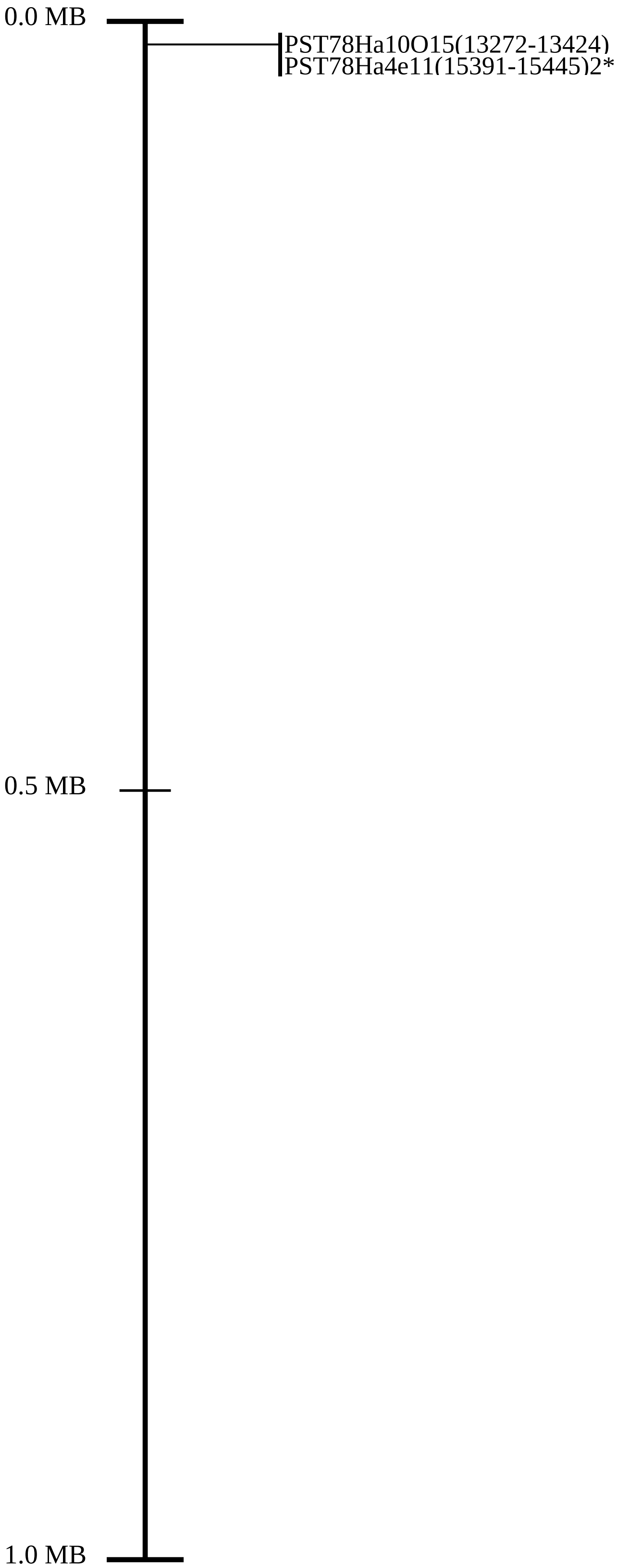

Supercontig 216
length: 15,718 bp

*** = less than 100bp alignment**

| = clones in the same contig

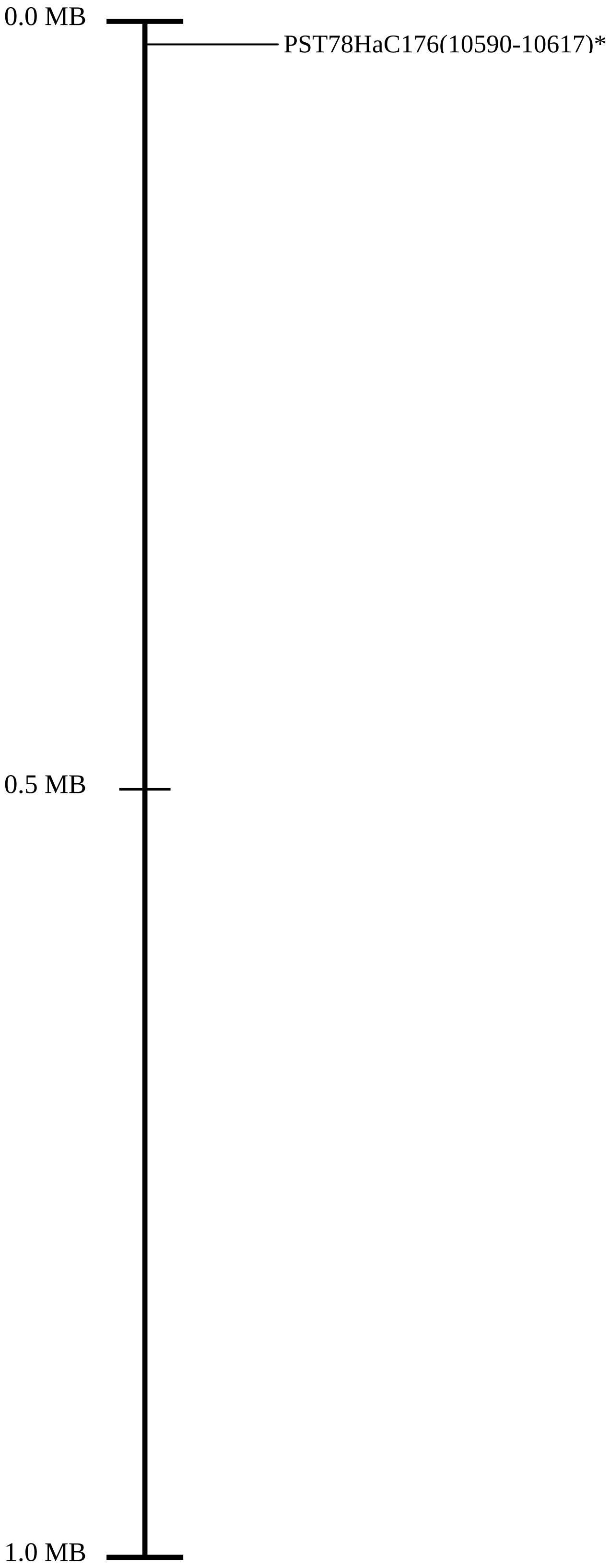

Supercontig 219
length: 15,090 bp

*** = less than 100bp alignment**

| = clones in the same contig

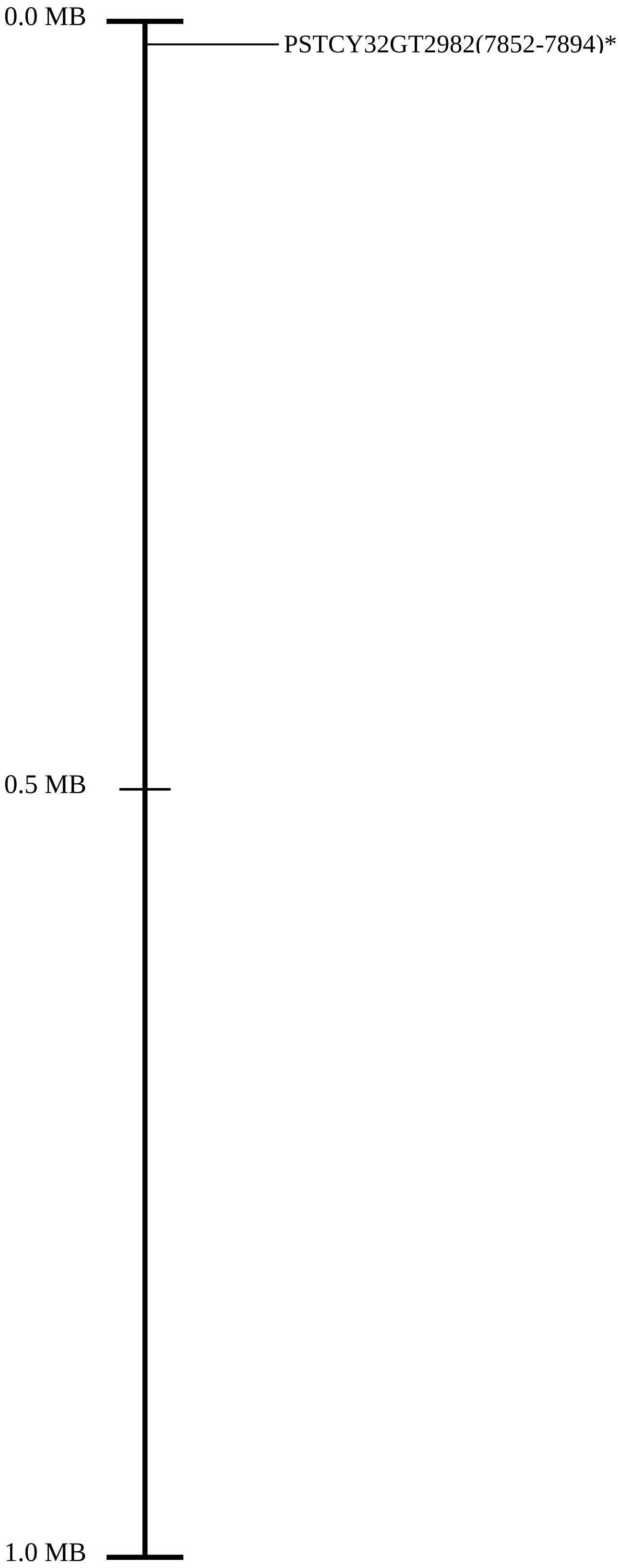

Supercontig 220
length: 14,899 bp

*** = less than 100bp alignment** **| = clones in the same contig**

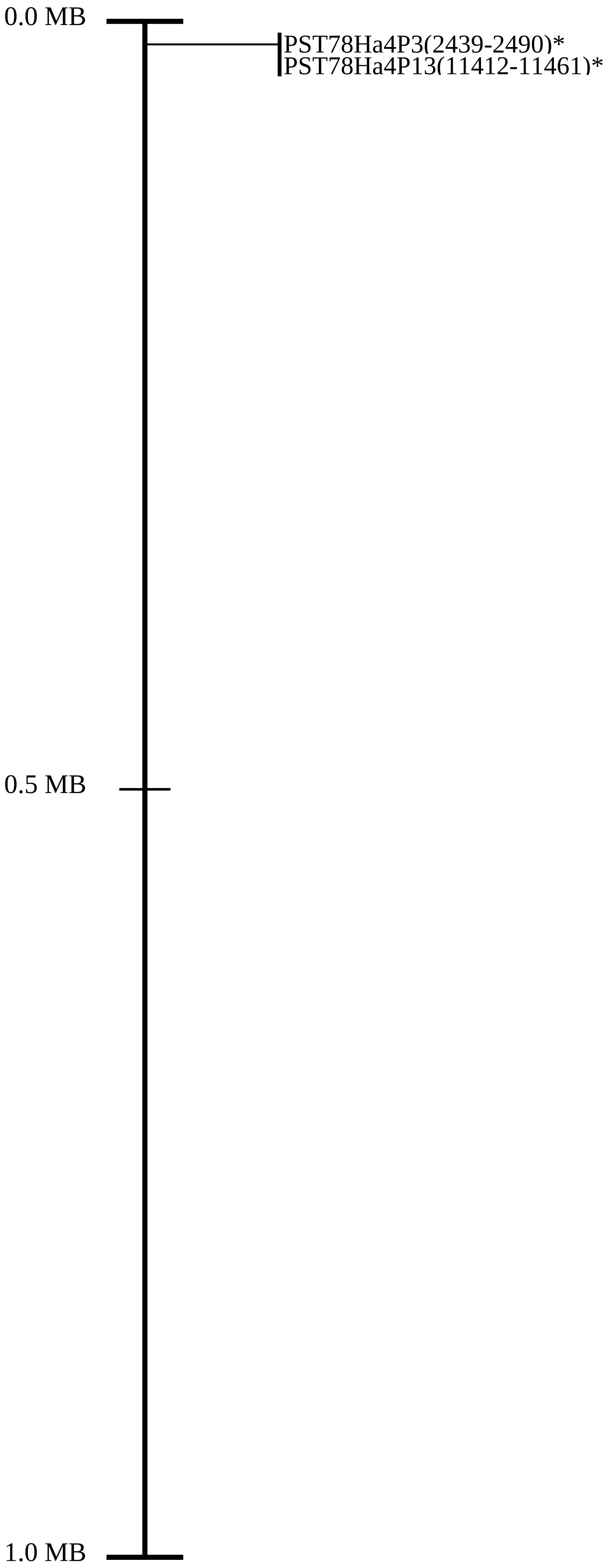

Supercontig 221
length: 14,629 bp

*** = less than 100bp alignment**

| = clones in the same contig

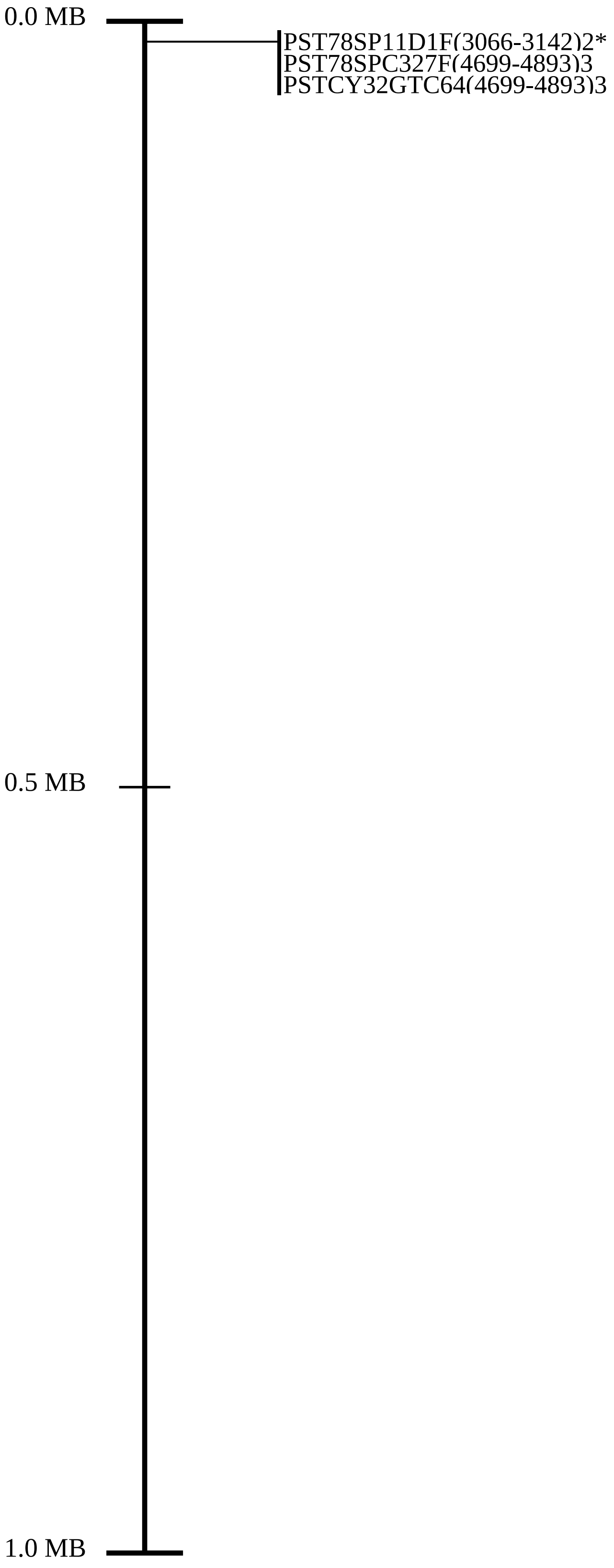

Supercontig 225
length: 13,856 bp

*** = less than 100bp alignment**

| = clones in the same contig

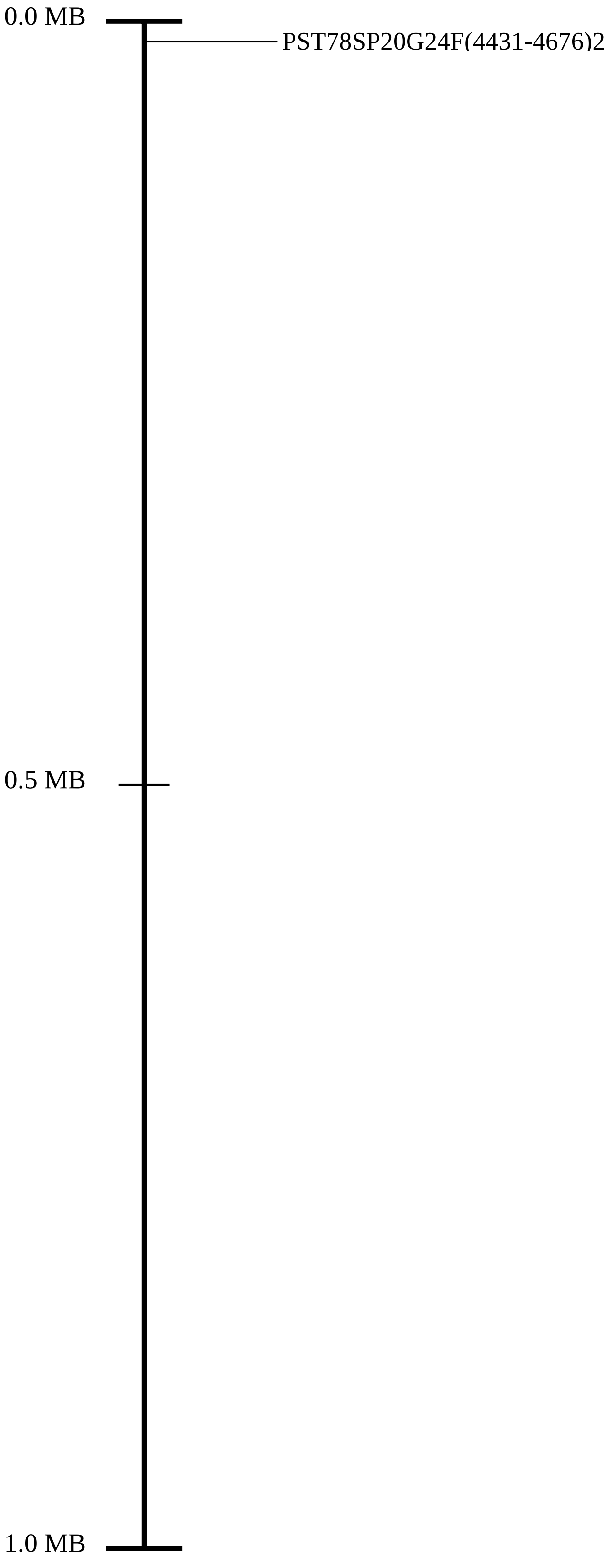

Supercontig 227
length: 13,483 bp

*** = less than 100bp alignment** **| = clones in the same contig**

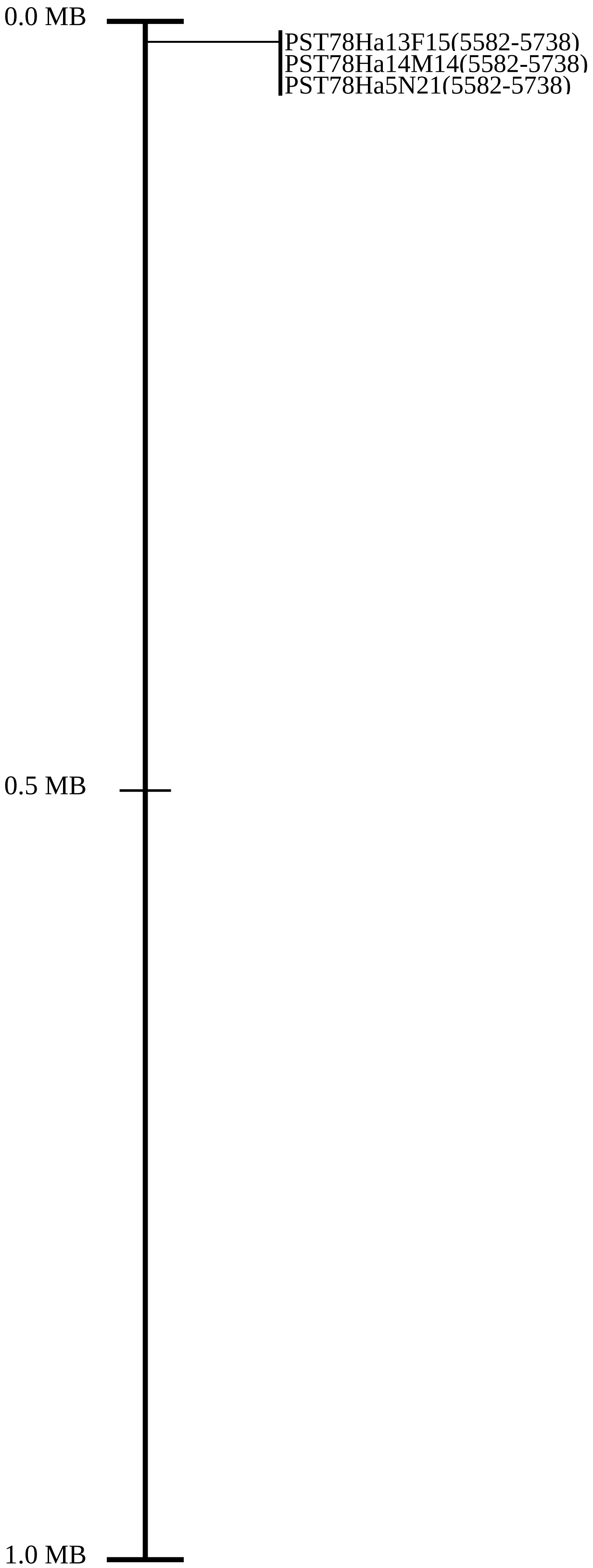

Supercontig 228
length: 13,460 bp

*** = less than 100bp alignment** **| = clones in the same contig**

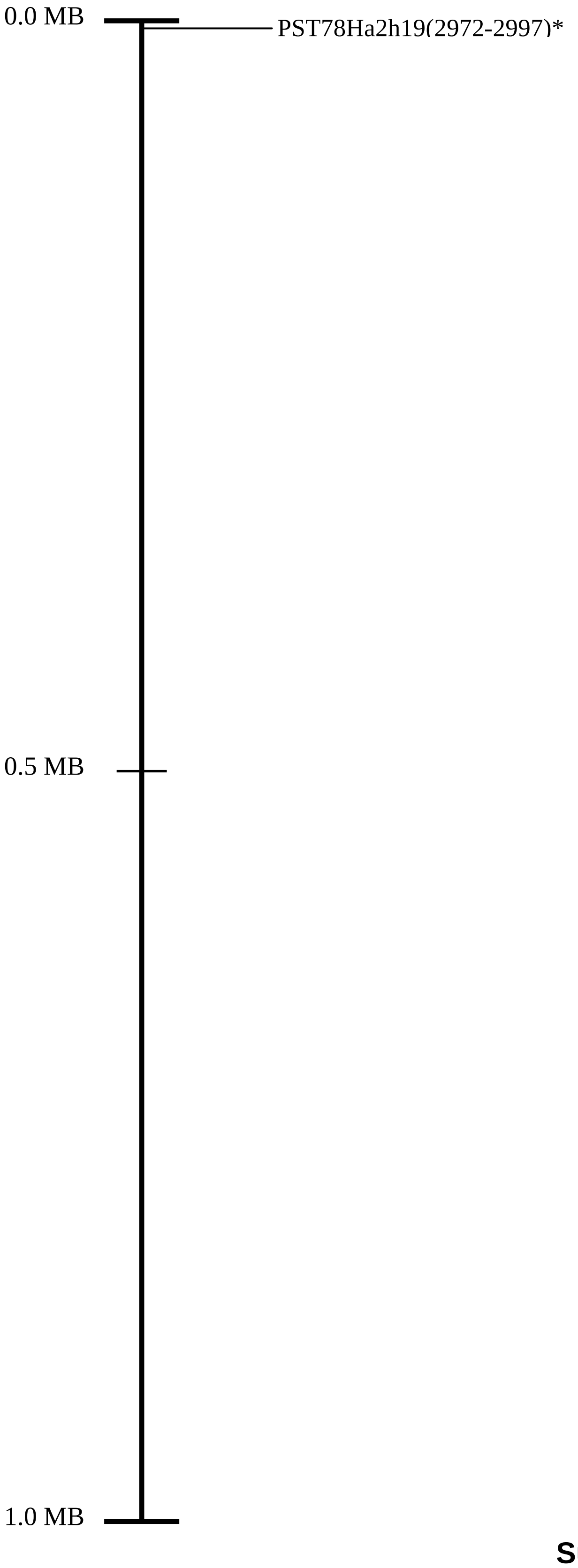

Supercontig 236
length: 12,526 bp

*** = less than 100bp alignment**

| = clones in the same contig

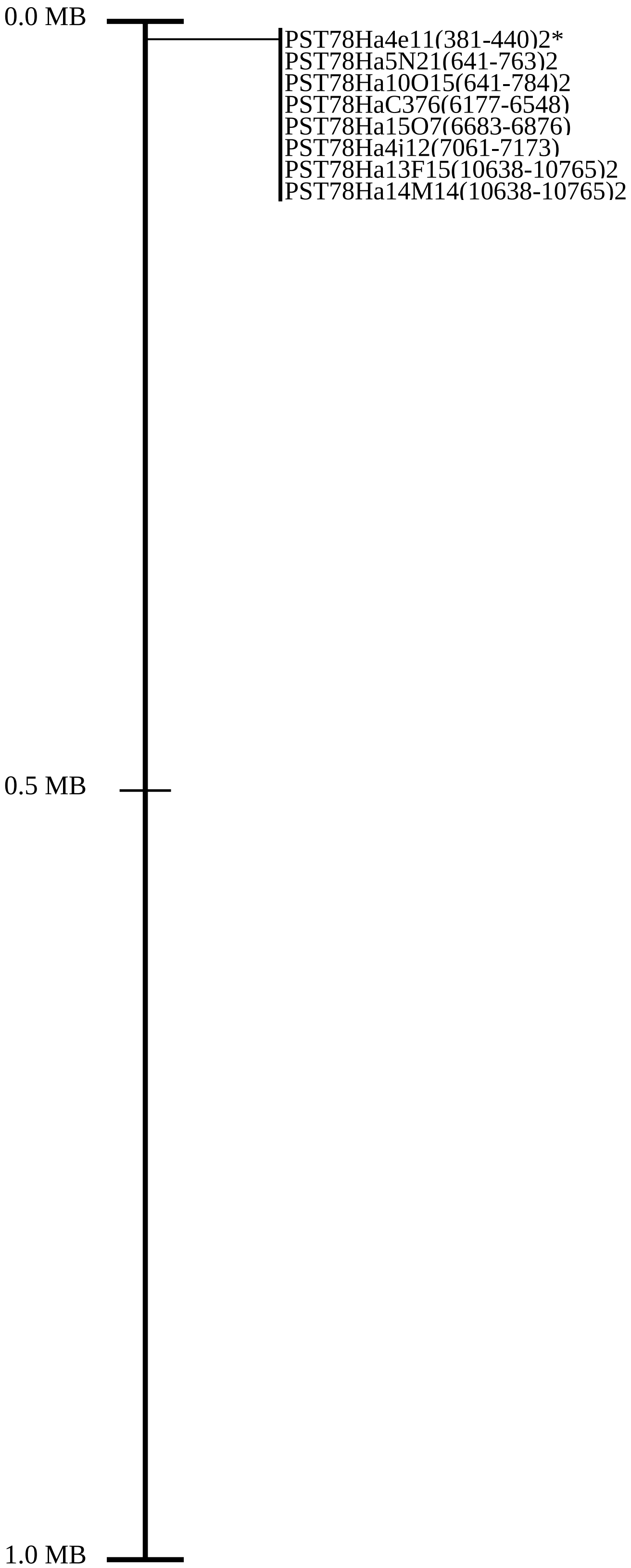

Supercontig 237
length: 12,336 bp

*** = less than 100bp alignment** **| = clones in the same contig**

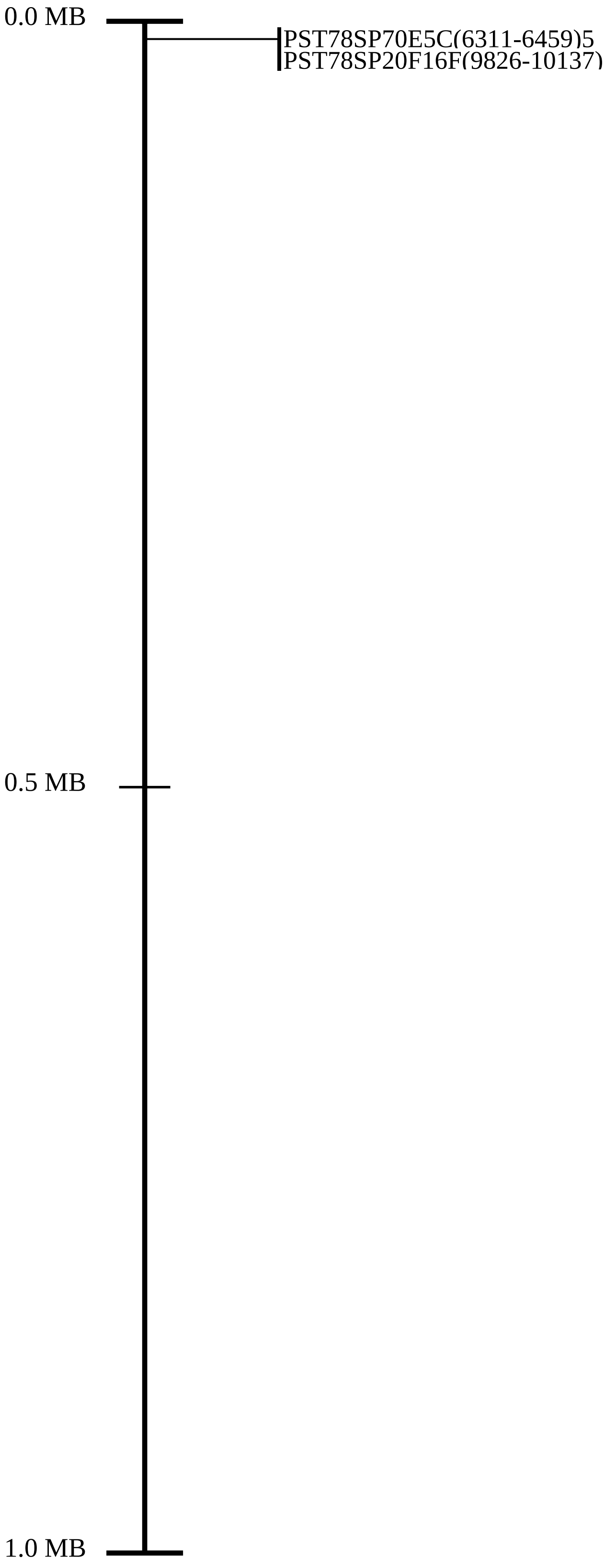

Supercontig 238
length: 12,284 bp

*** = less than 100bp alignment**

| = clones in the same contig

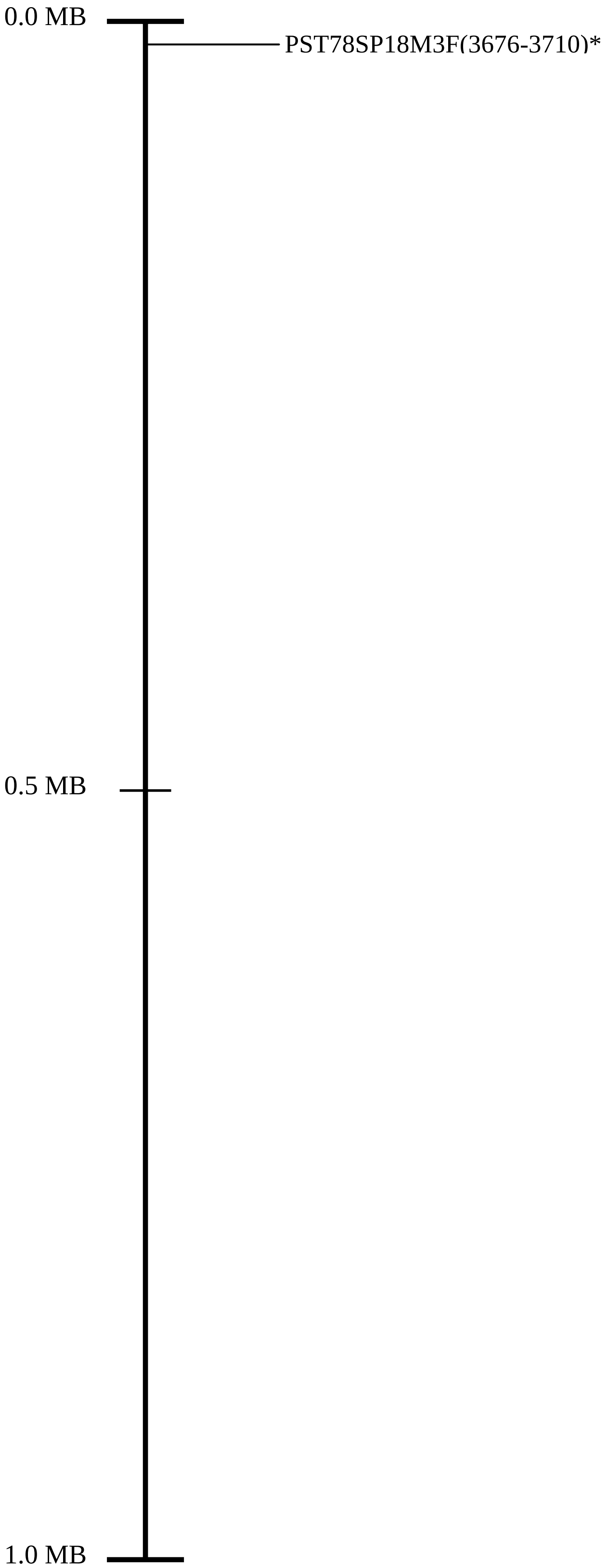

Supercontig 239
length: 15,052 bp

*** = less than 100bp alignment** **| = clones in the same contig**

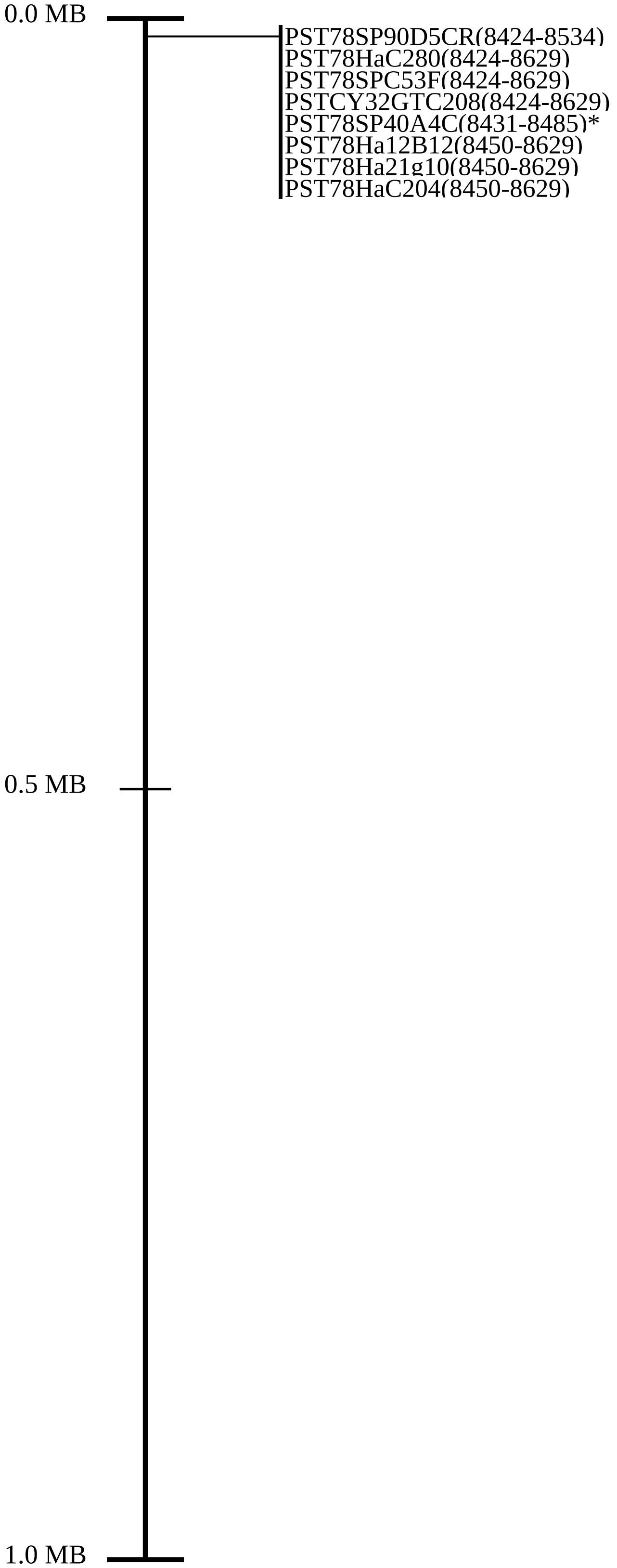

Supercontig 241
length: 12,067 bp

*** = less than 100bp alignment** **| = clones in the same contig**

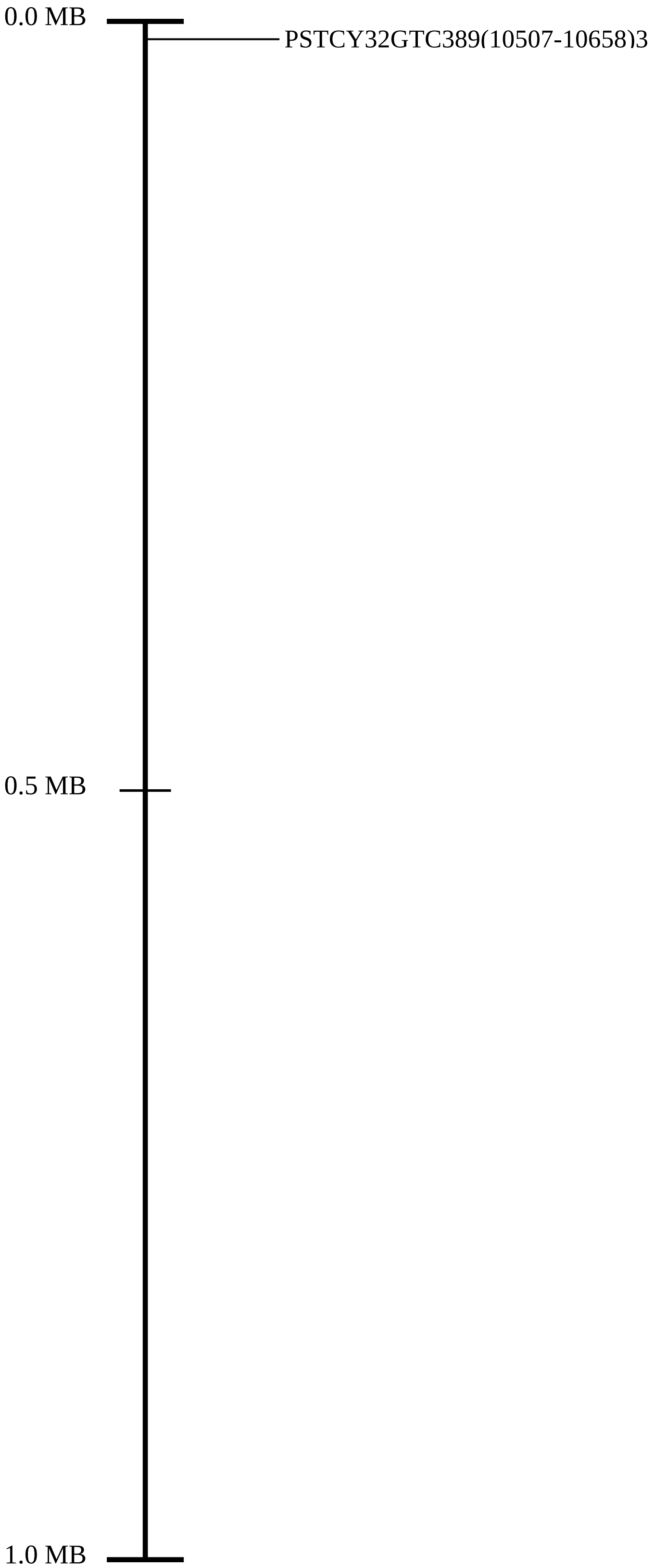

Supercontig 246
length: 11,389 bp

*** = less than 100bp alignment** **| = clones in the same contig**

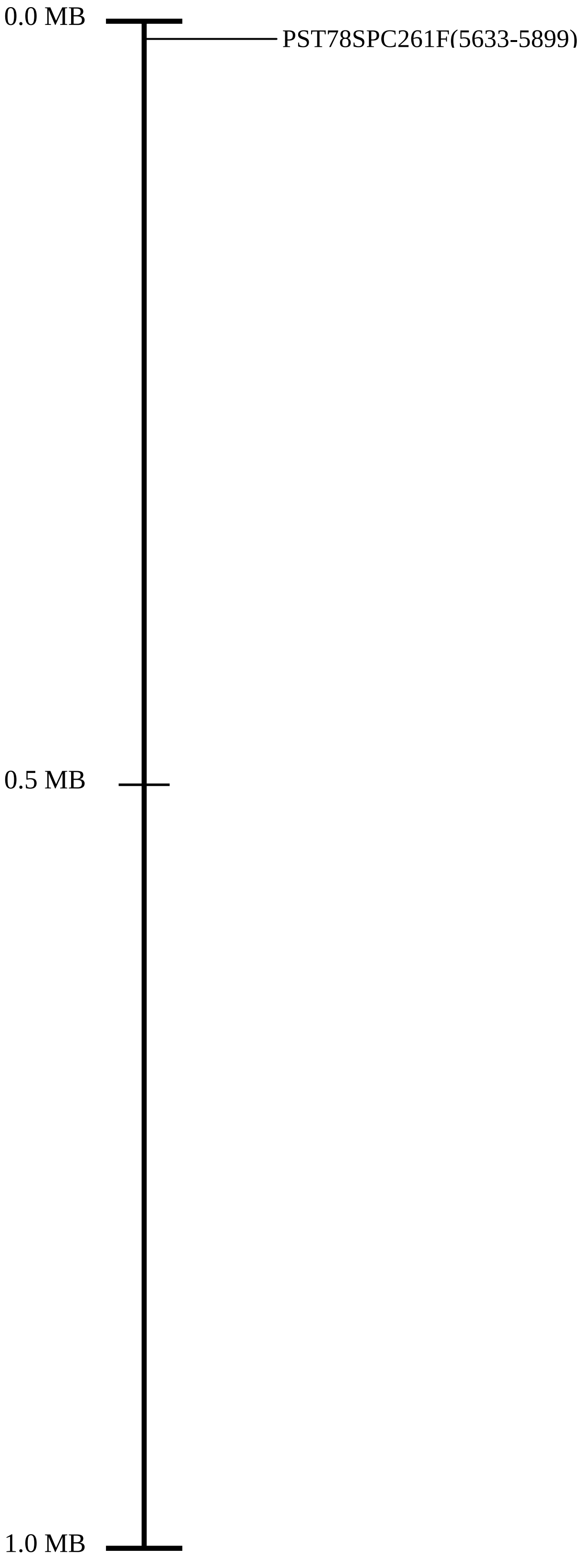

Supercontig 249
length: 10,855 bp

*** = less than 100bp alignment** **| = clones in the same contig**

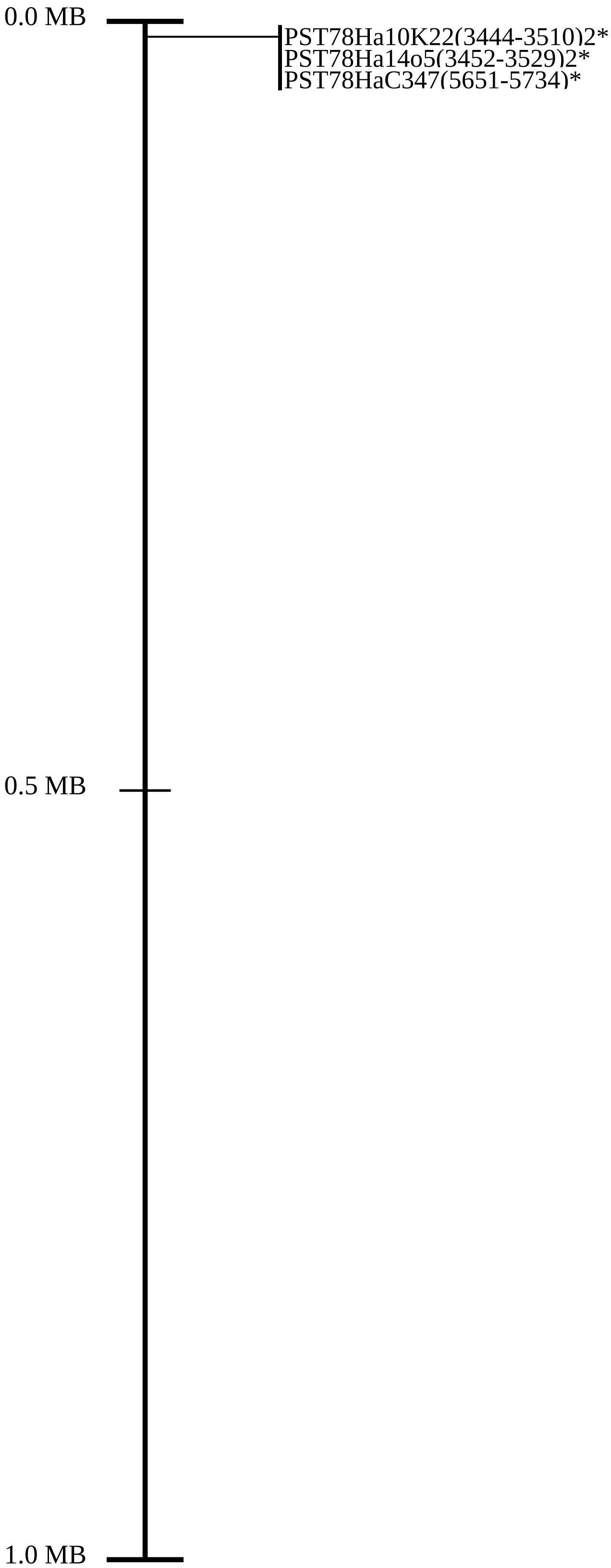

Supercontig 256
length: 10,451 bp

*** = less than 100bp alignment**

| = clones in the same contig

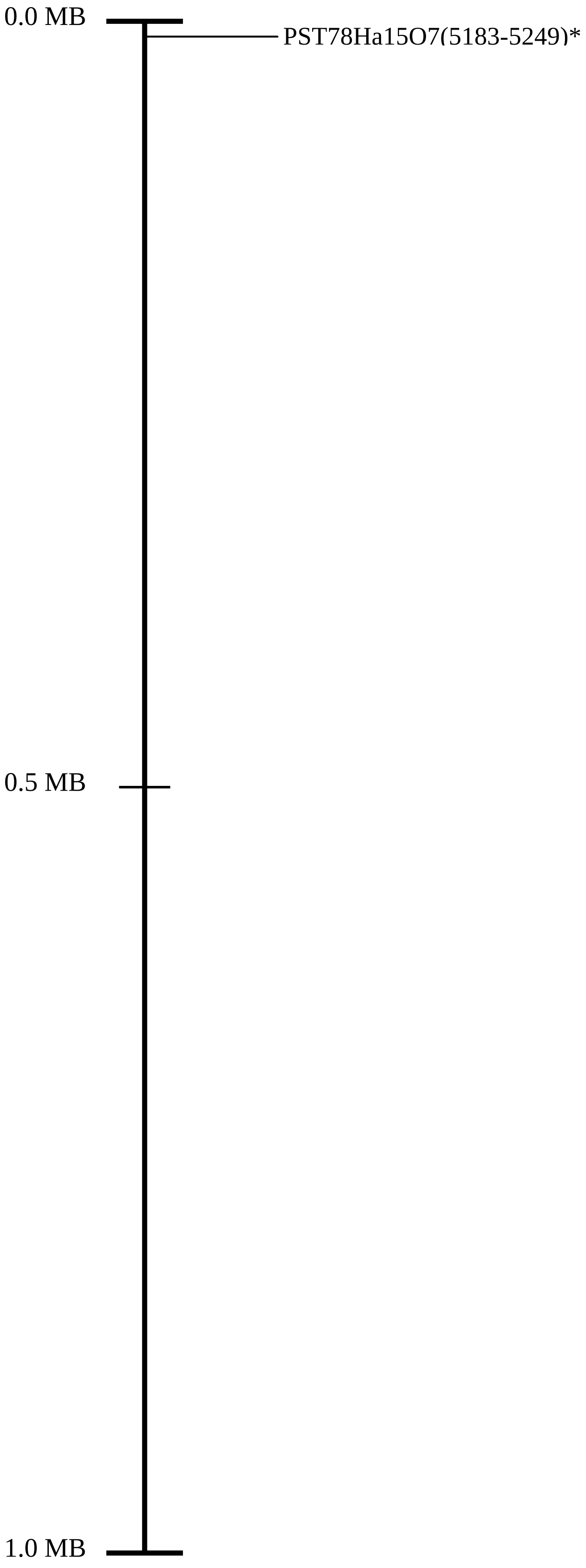

Supercontig 262
length: 10,127 bp

*** = less than 100bp alignment**

| = clones in the same contig

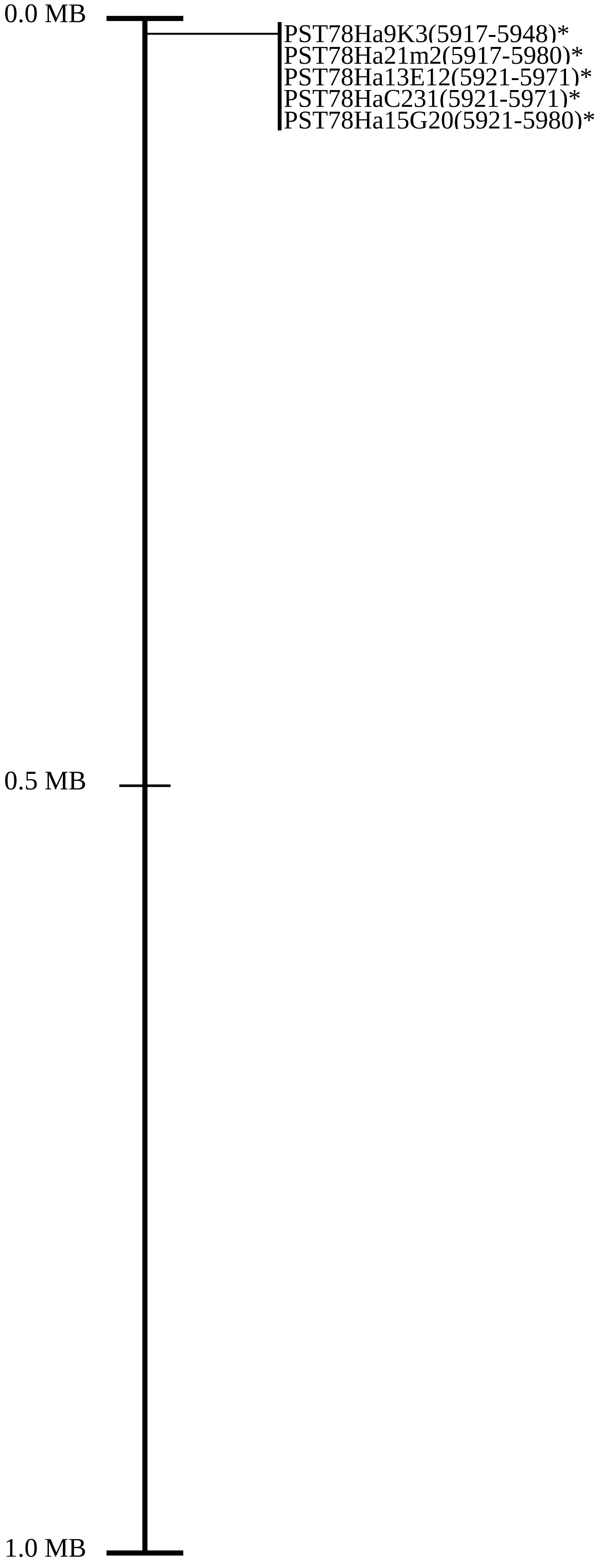

Supercontig 263
length: 10,113 bp

*** = less than 100bp alignment** **| = clones in the same contig**

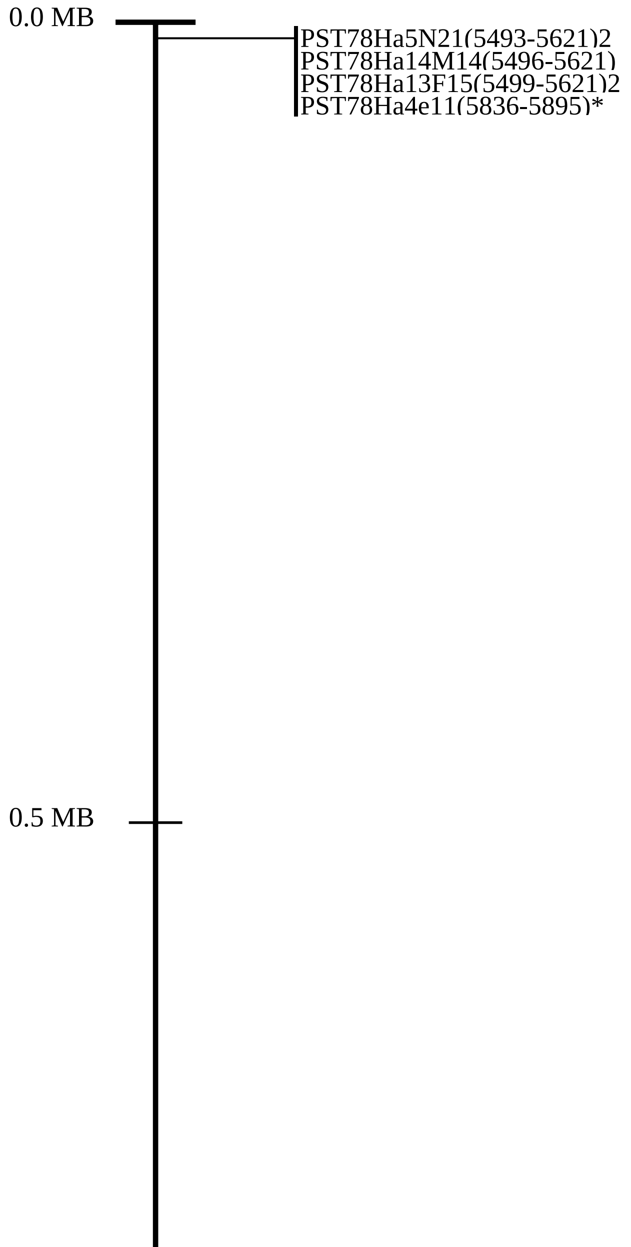

Supercontig 265
length: 10,029 bp

*** = less than 100bp alignment** **| = clones in the same contig**

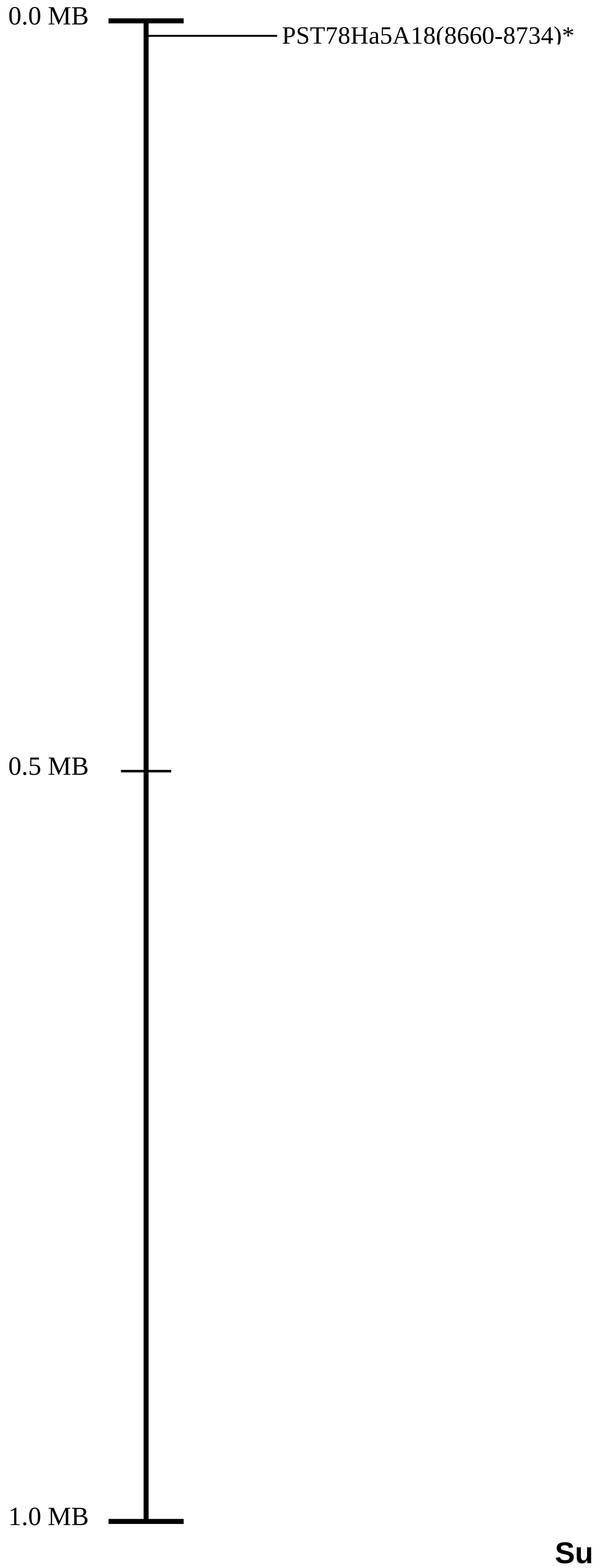

Supercontig 267
length: 9,743 bp

*** = less than 100bp alignment**

| = clones in the same contig

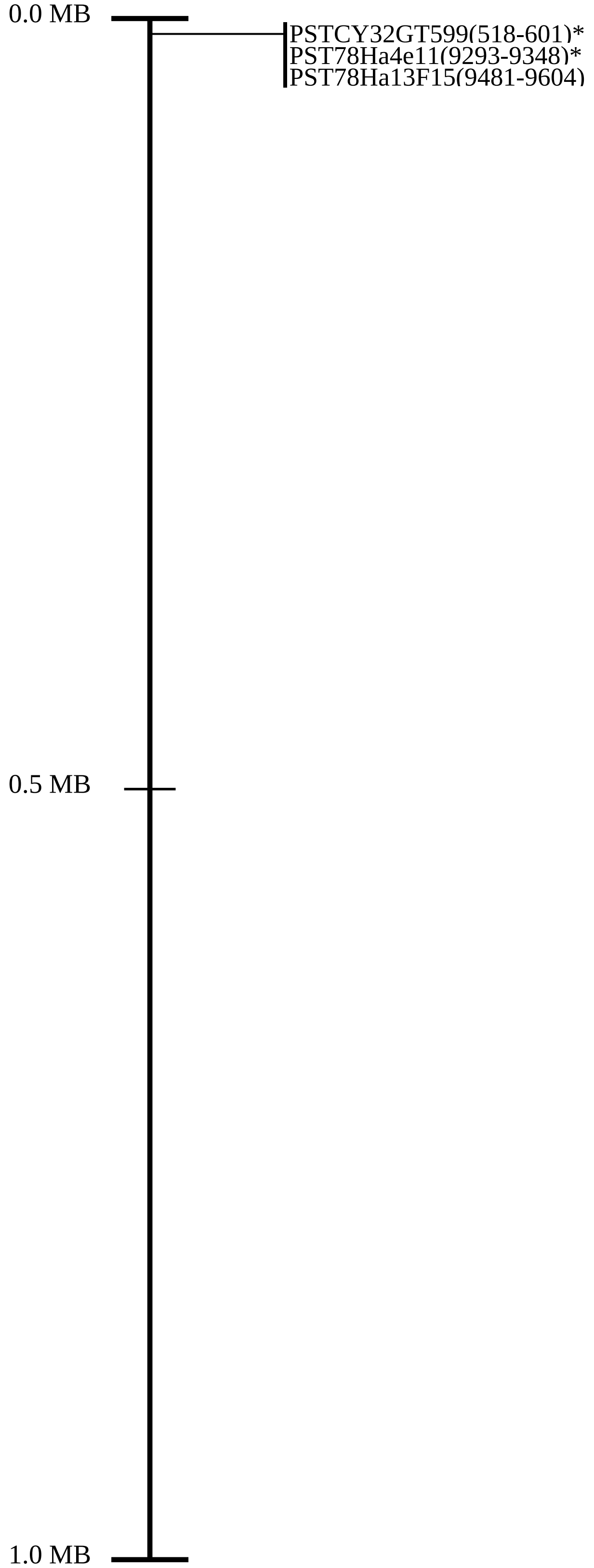

Supercontig 268
length: 9,738 bp

*** = less than 100bp alignment** **| = clones in the same contig**

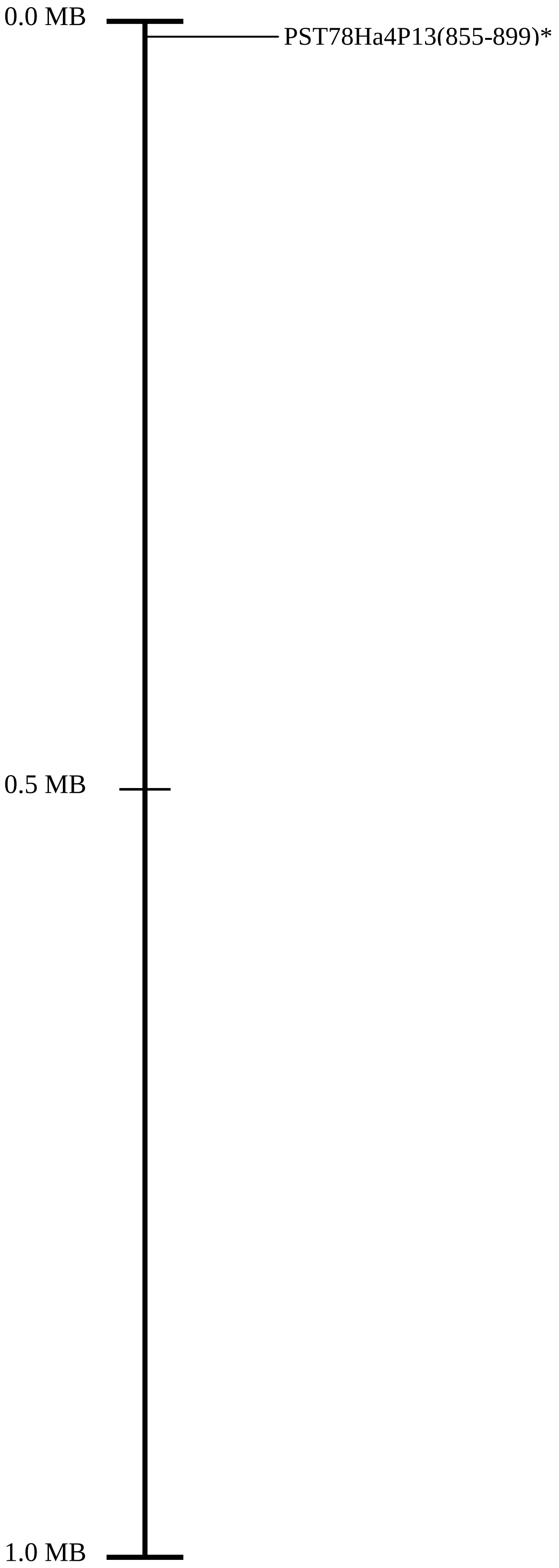

Supercontig 270
length: 9,523 bp

*** = less than 100bp alignment** **| = clones in the same contig**

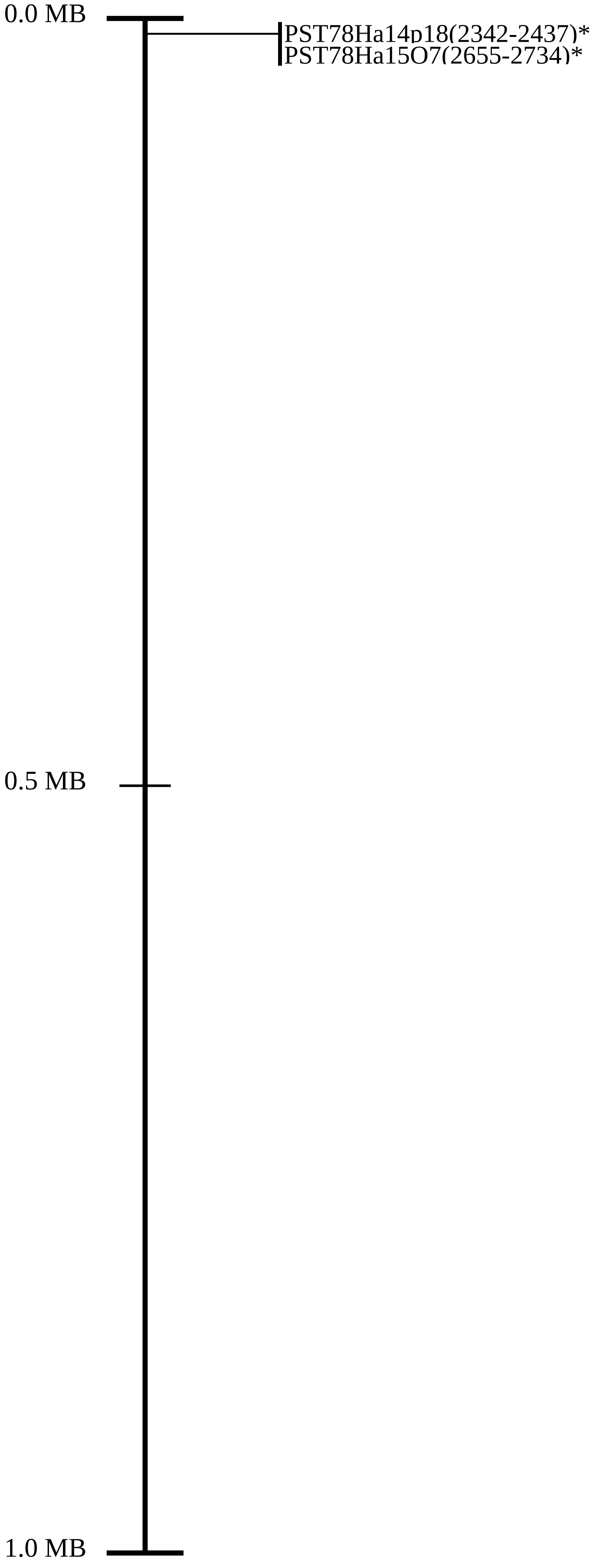

Supercontig 271
length: 9,385 bp

*** = less than 100bp alignment** **| = clones in the same contig**

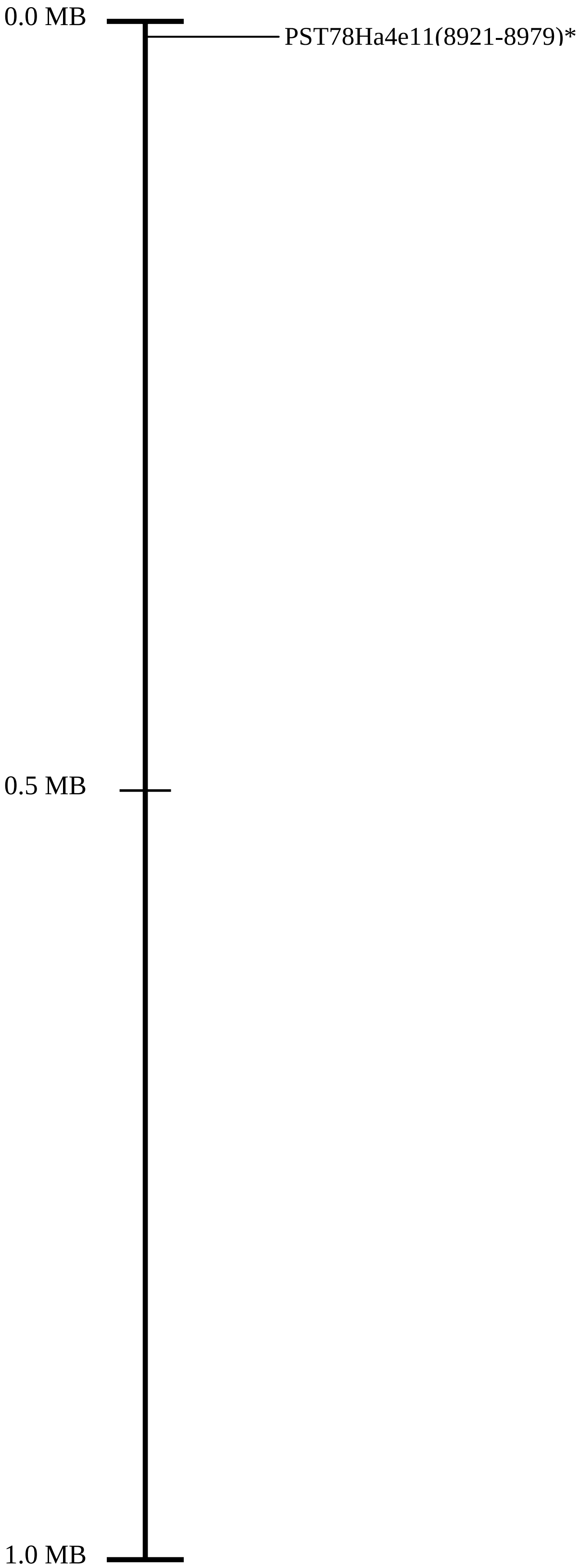

Supercontig 273
length: 9,255 bp

*** = less than 100bp alignment**

| = clones in the same contig

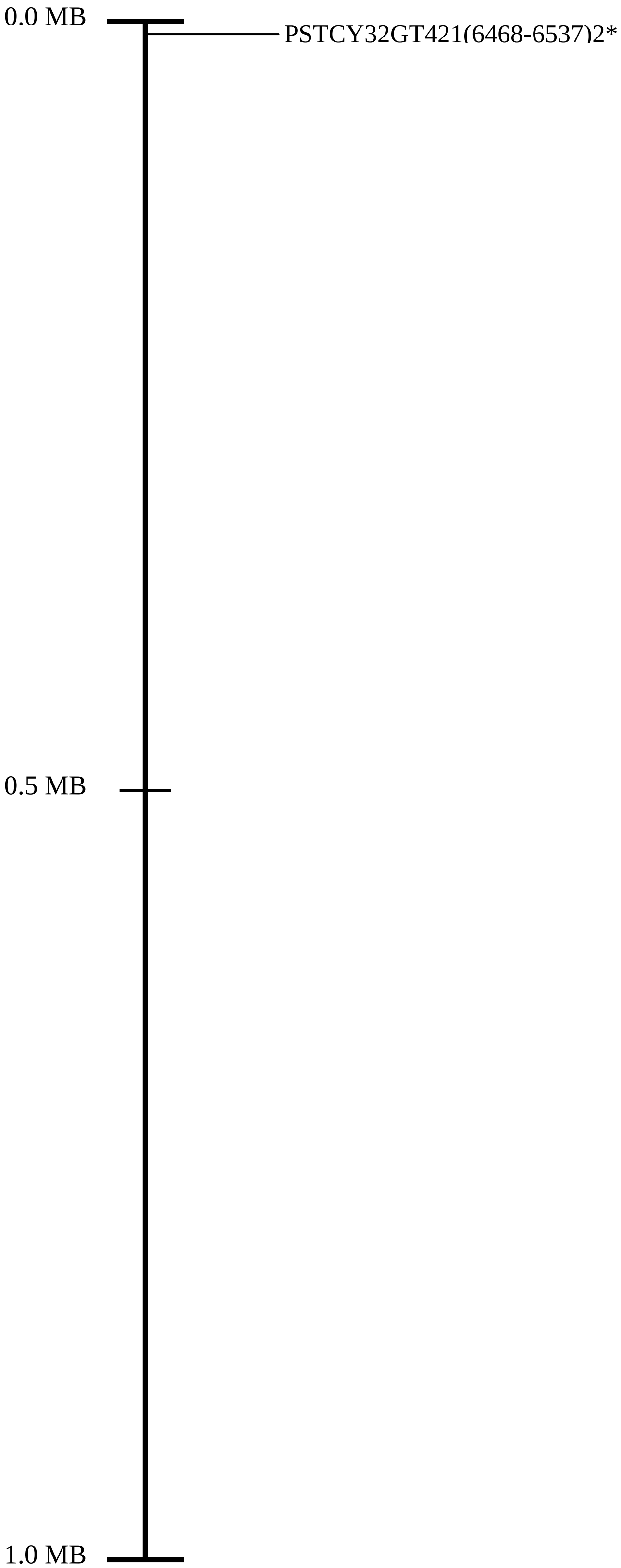

Supercontig 276
length: 8,976 bp

*** = less than 100bp alignment** **| = clones in the same contig**

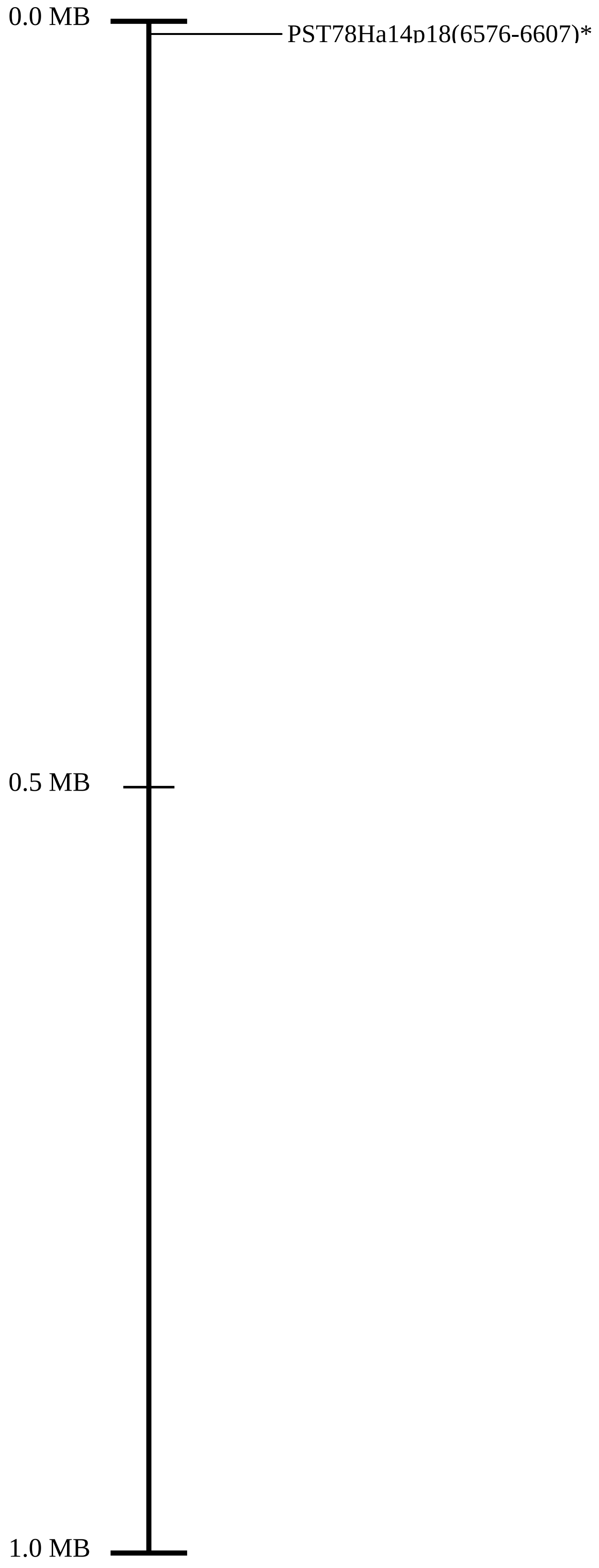

Supercontig 278
length: 8,937 bp

*** = less than 100bp alignment** **| = clones in the same contig**

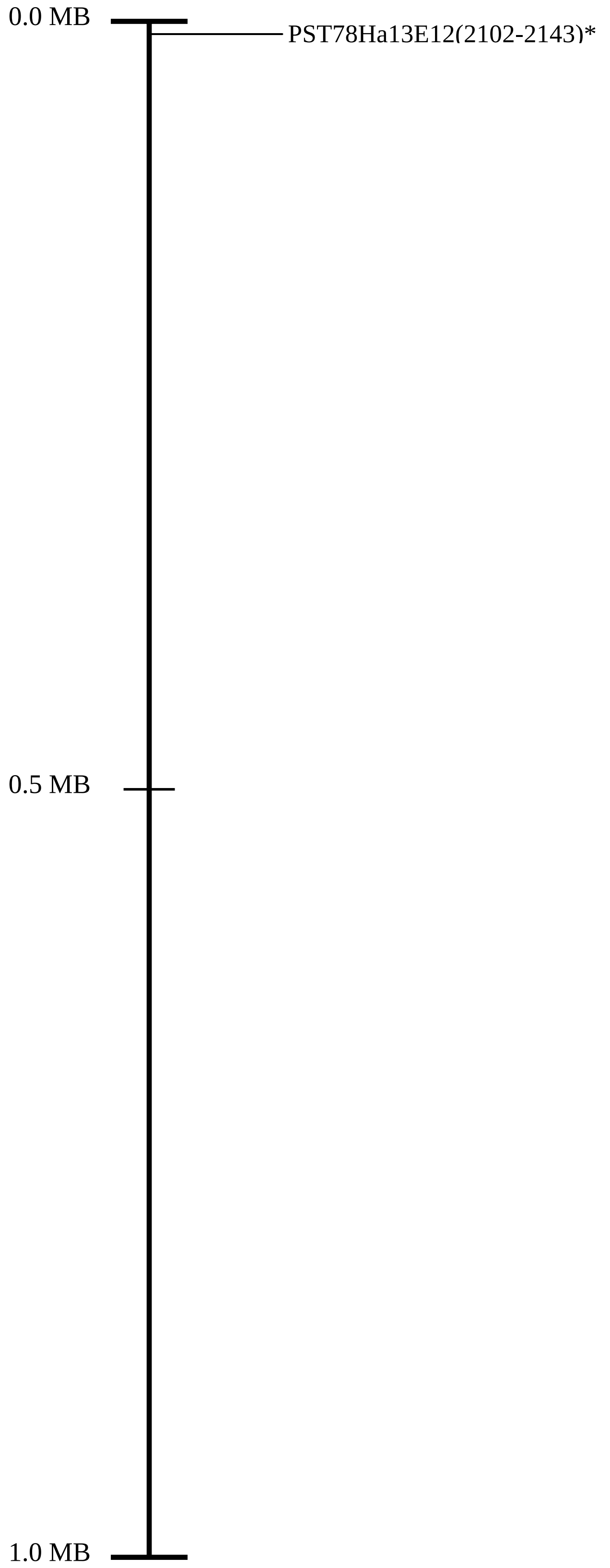

Supercontig 286
length: 8,437 bp

*** = less than 100bp alignment**

| = clones in the same contig

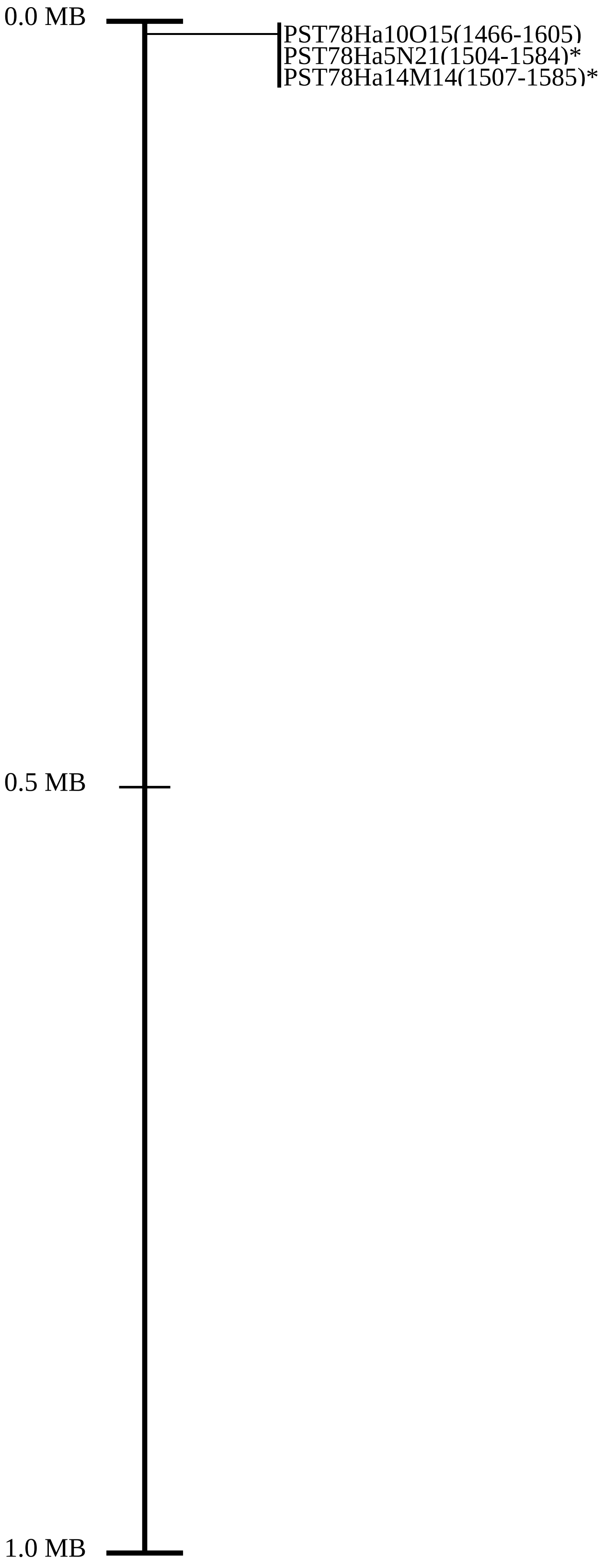

Supercontig 287
length: 8,404 bp

*** = less than 100bp alignment** **| = clones in the same contig**

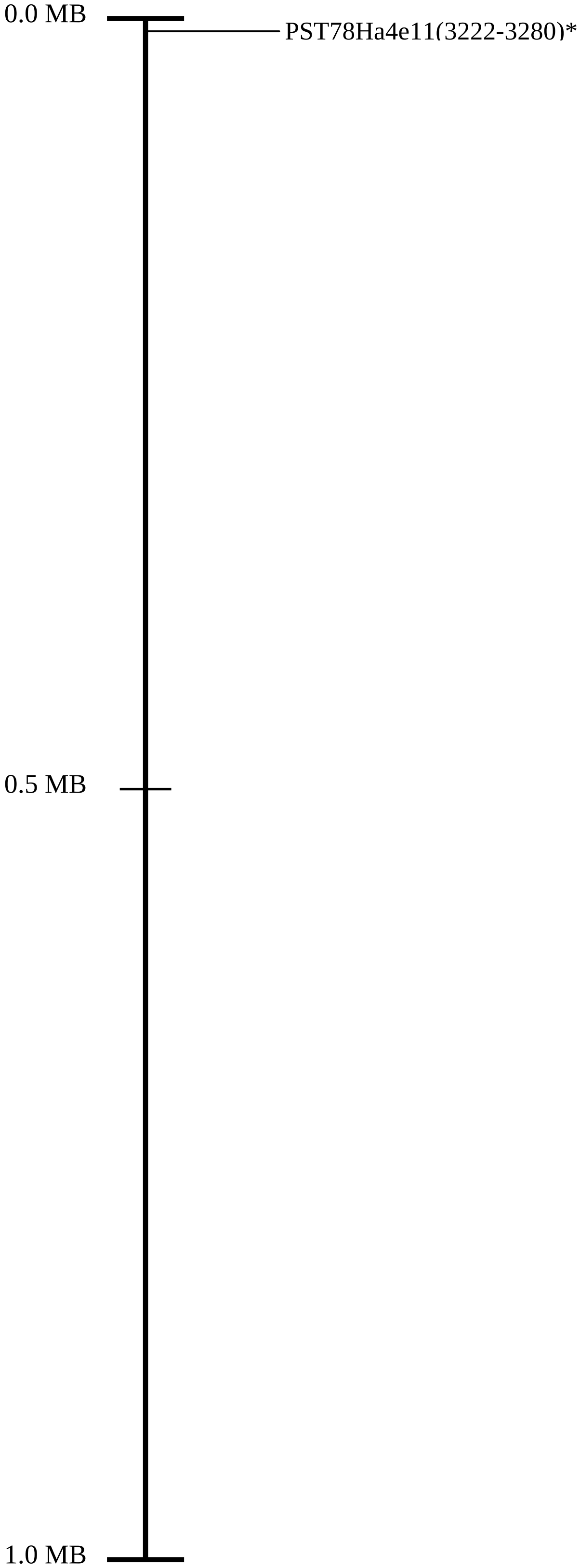

Supercontig 291
length: 8,194 bp

*** = less than 100bp alignment** **| = clones in the same contig**

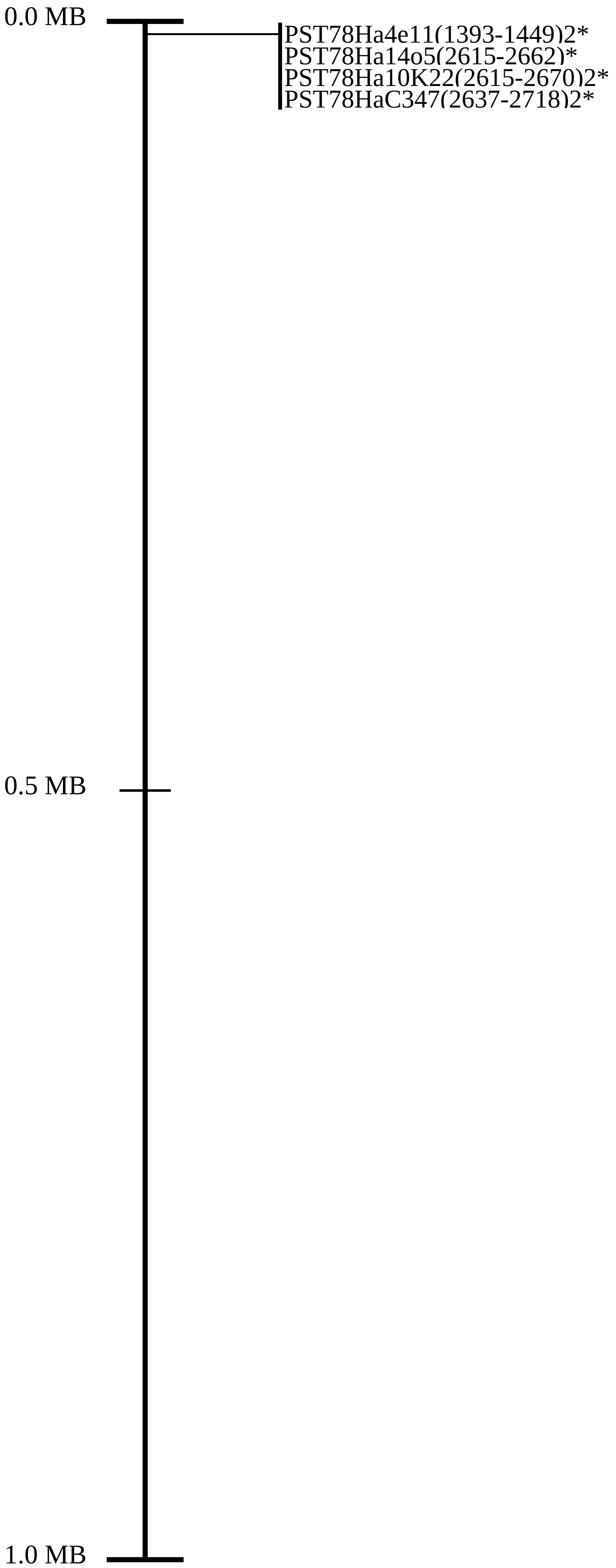

Supercontig 292
length: 8,128 bp

*** = less than 100bp alignment**

| = clones in the same contig

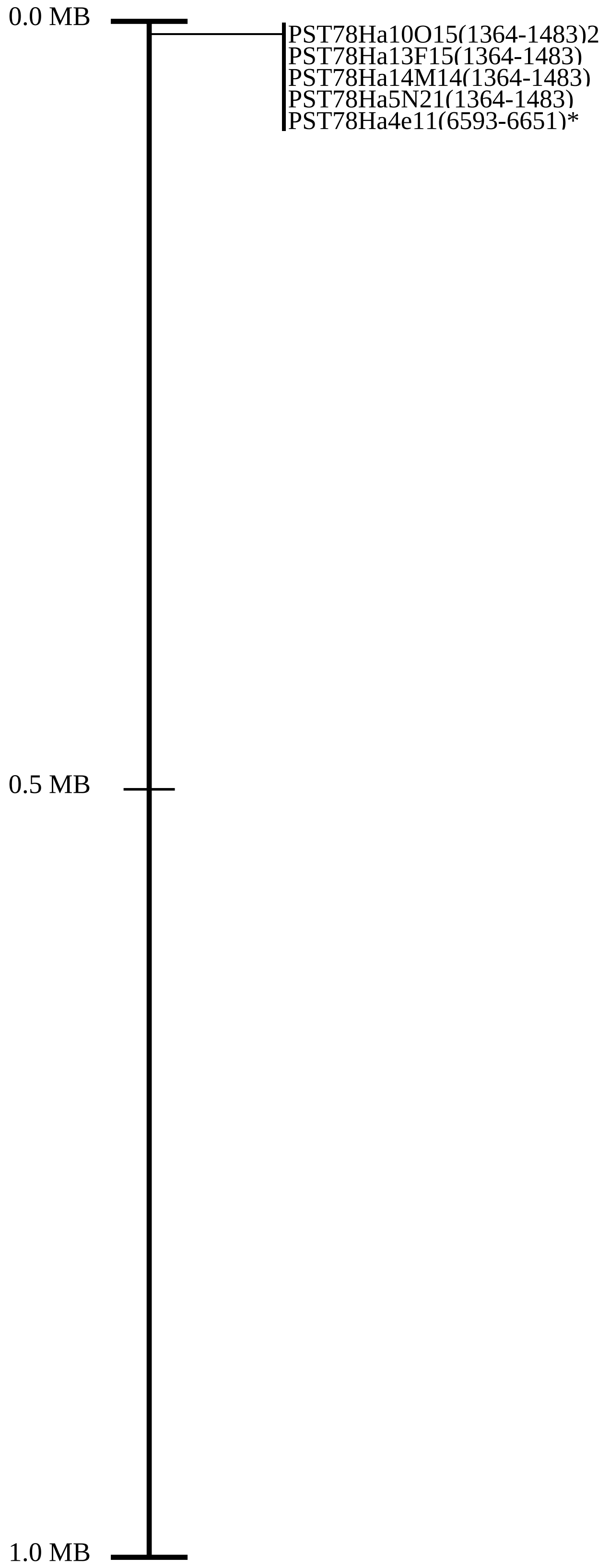

Supercontig 293
length: 8,101 bp

*** = less than 100bp alignment** **| = clones in the same contig**

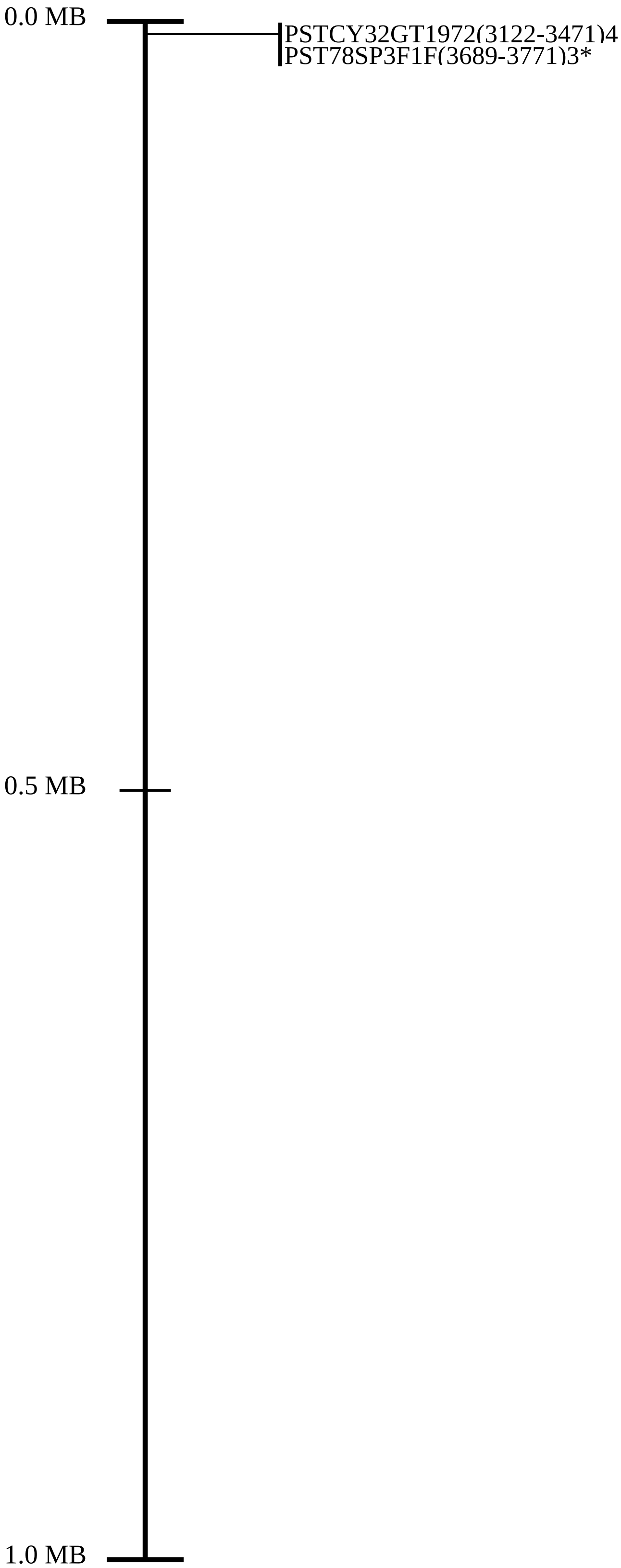

Supercontig 295
length: 8,046 bp

*** = less than 100bp alignment** **| = clones in the same contig**

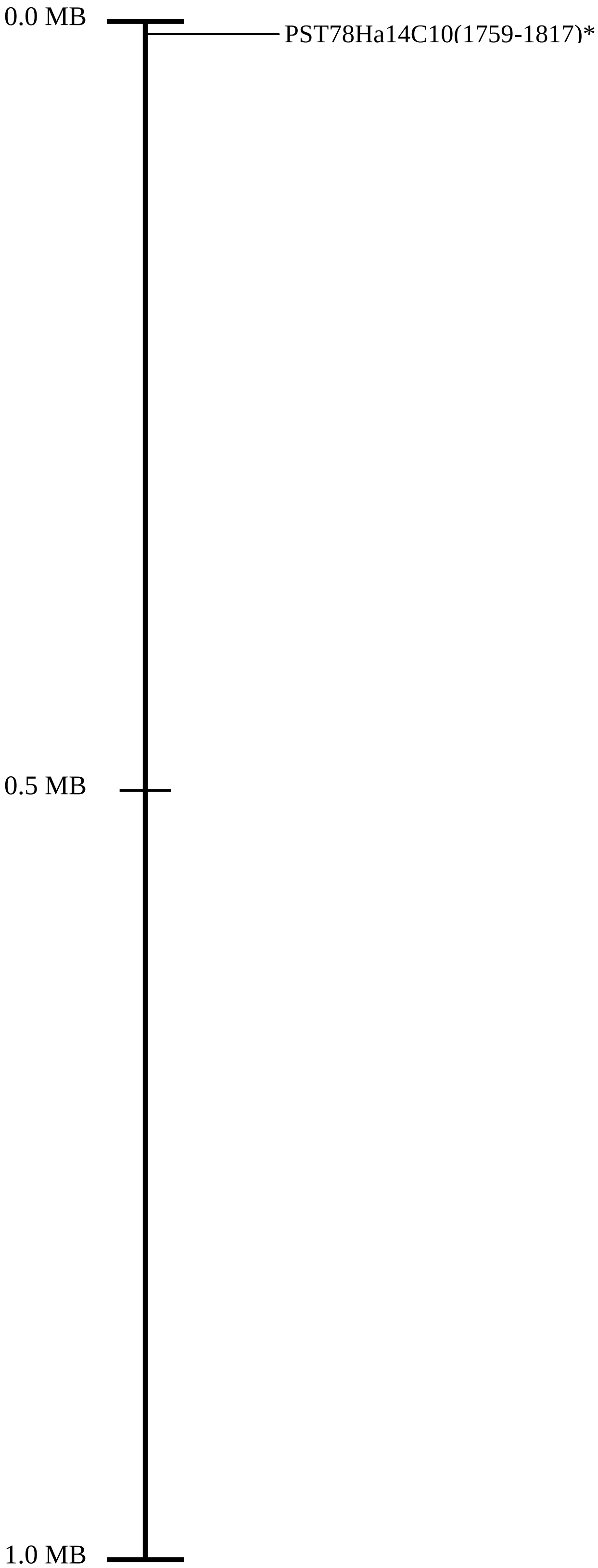

Supercontig 298
length: 7,881 bp

*** = less than 100bp alignment** **| = clones in the same contig**

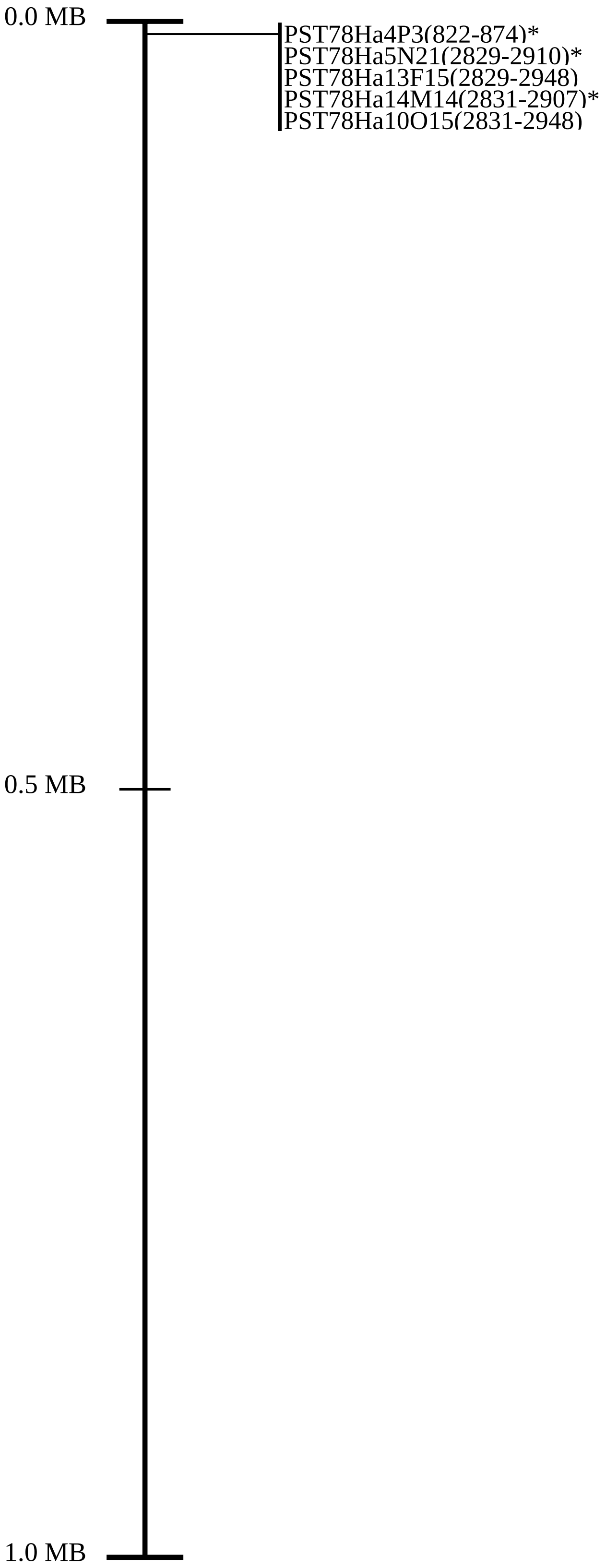

Supercontig 299
length: 7,678 bp

*** = less than 100bp alignment** **| = clones in the same contig**

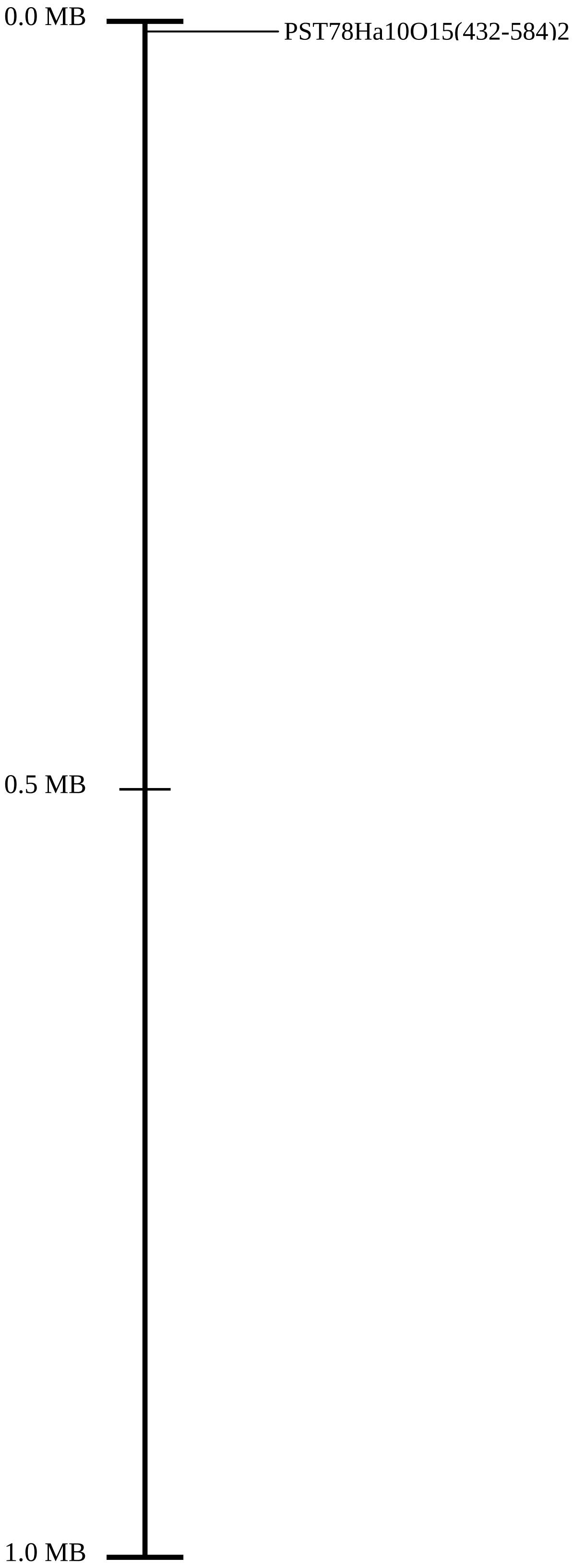

Supercontig 307
length: 7,428 bp

*** = less than 100bp alignment**

| = clones in the same contig

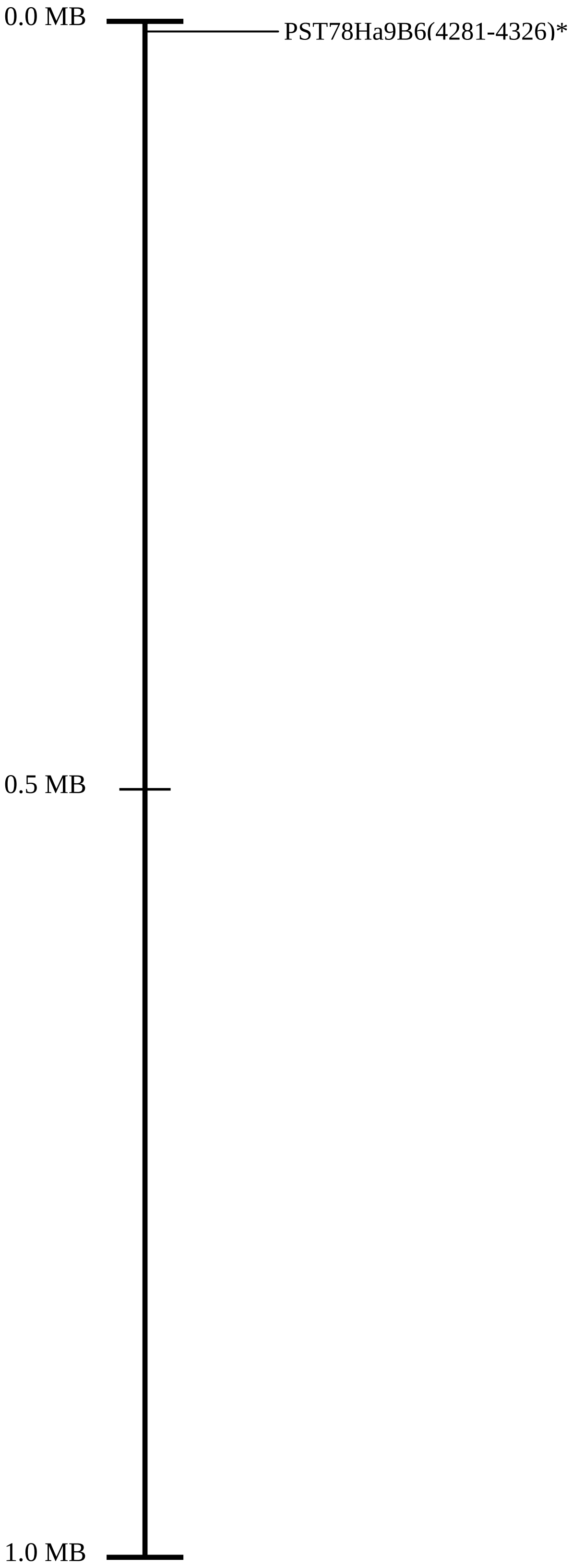

Supercontig 311
length: 7,373 bp

*** = less than 100bp alignment**

| = clones in the same contig

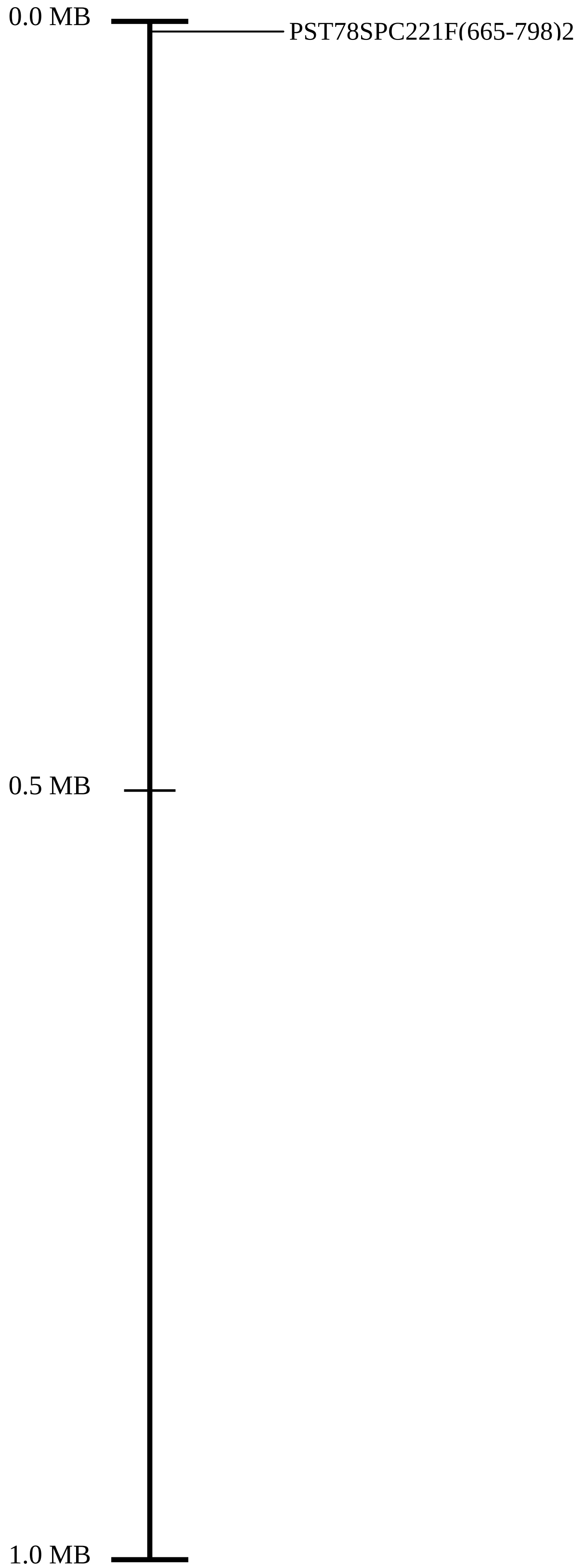

Supercontig 313
length: 7,328 bp

*** = less than 100bp alignment**

| = clones in the same contig

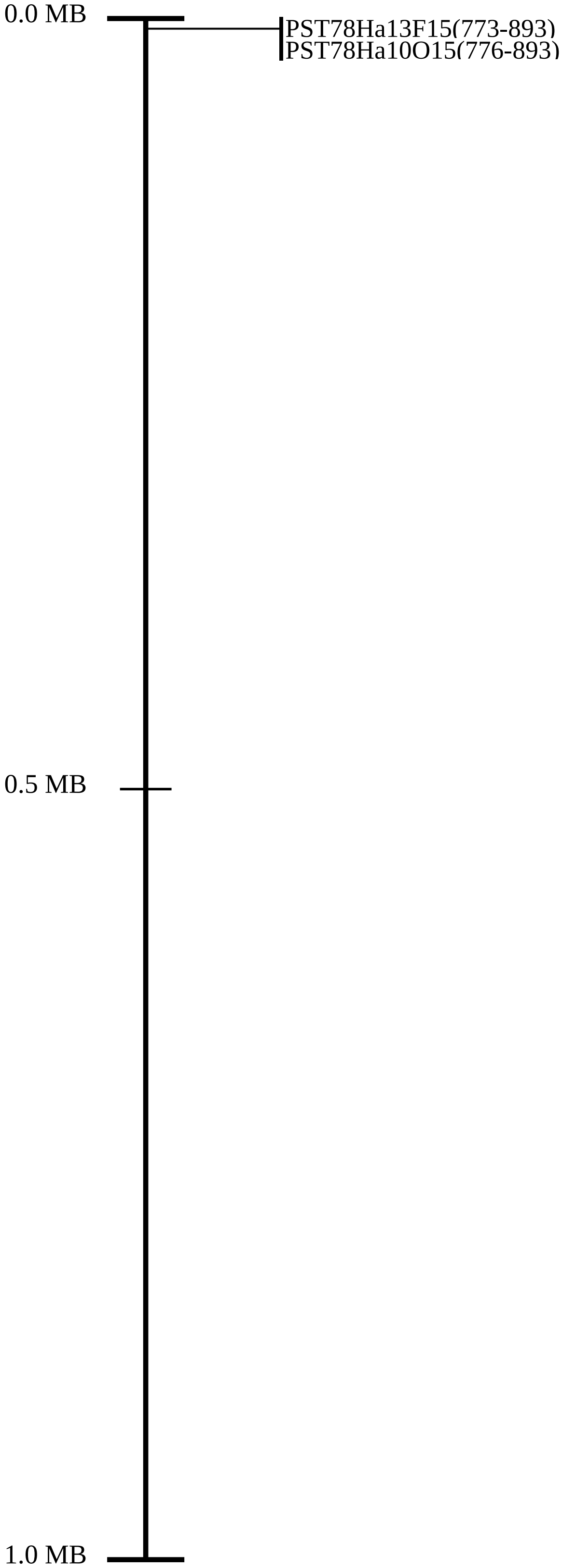

Supercontig 325
length: 6,734 bp

*** = less than 100bp alignment**

| = clones in the same contig

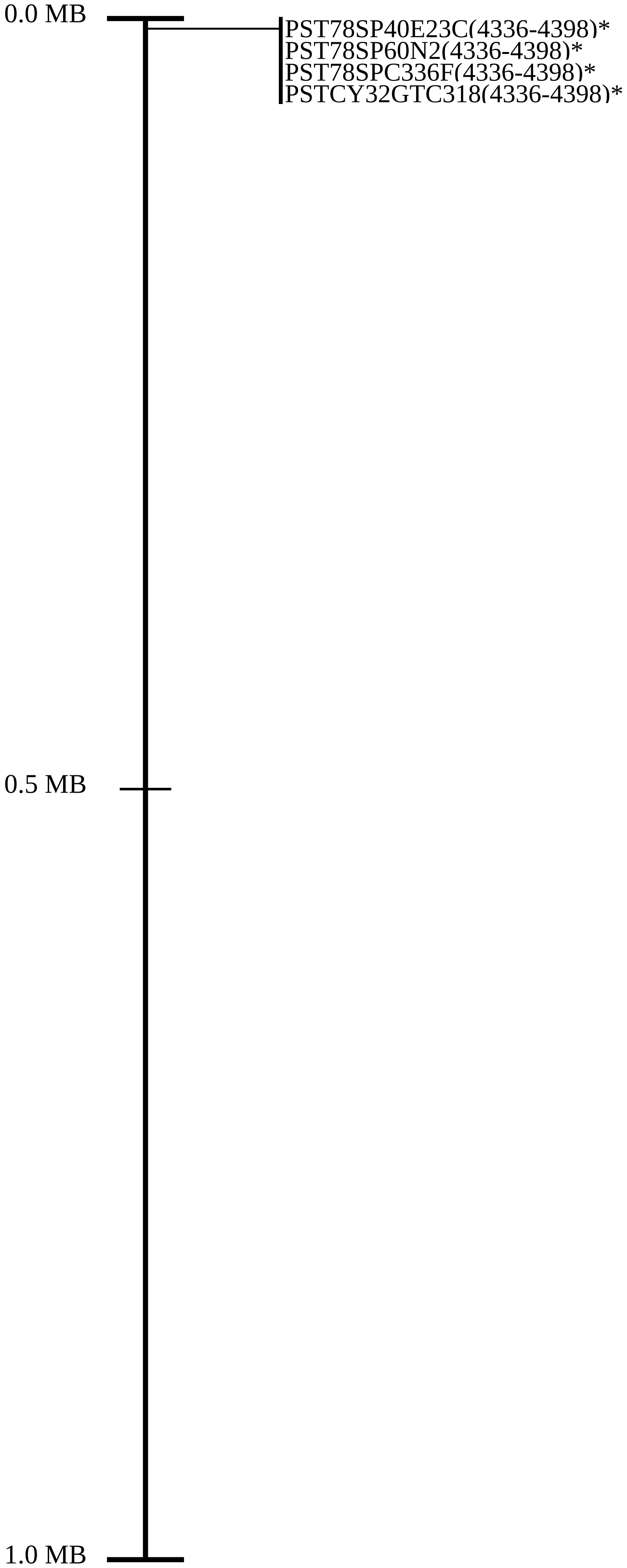

Supercontig 331
length: 6,471 bp

*** = less than 100bp alignment** **| = clones in the same contig**

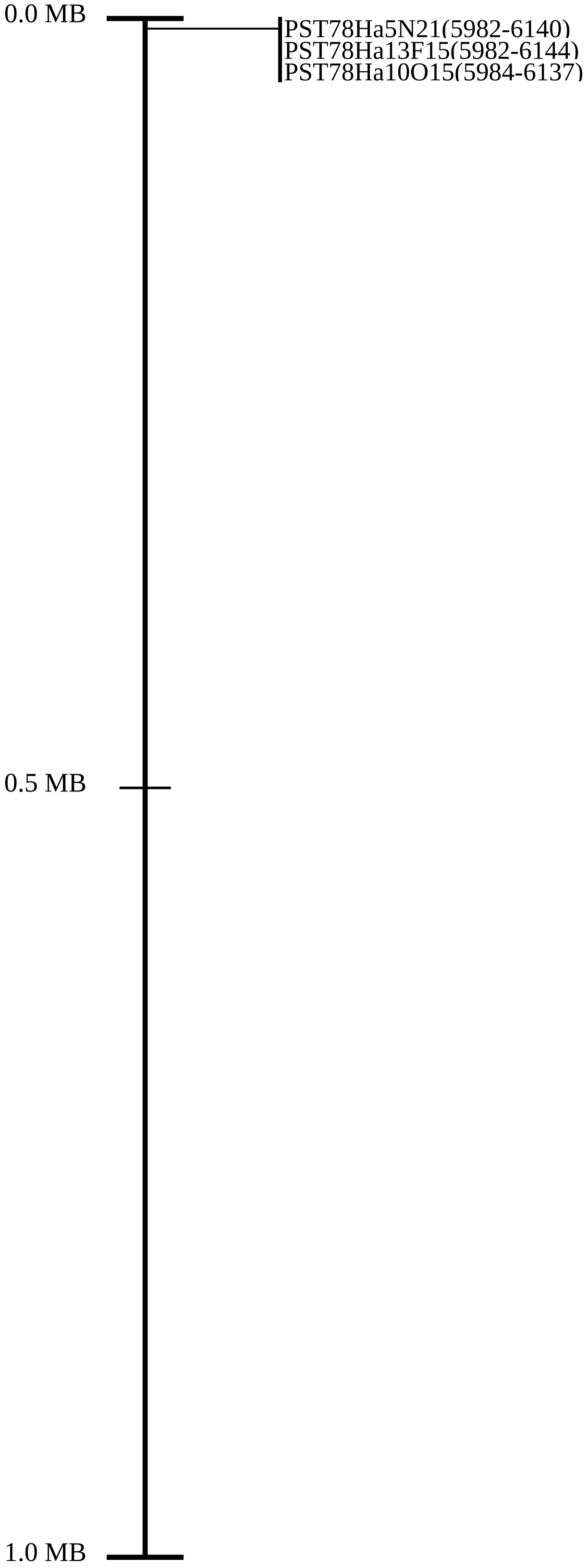

Supercontig 340
length: 6,192 bp

*** = less than 100bp alignment** **| = clones in the same contig**

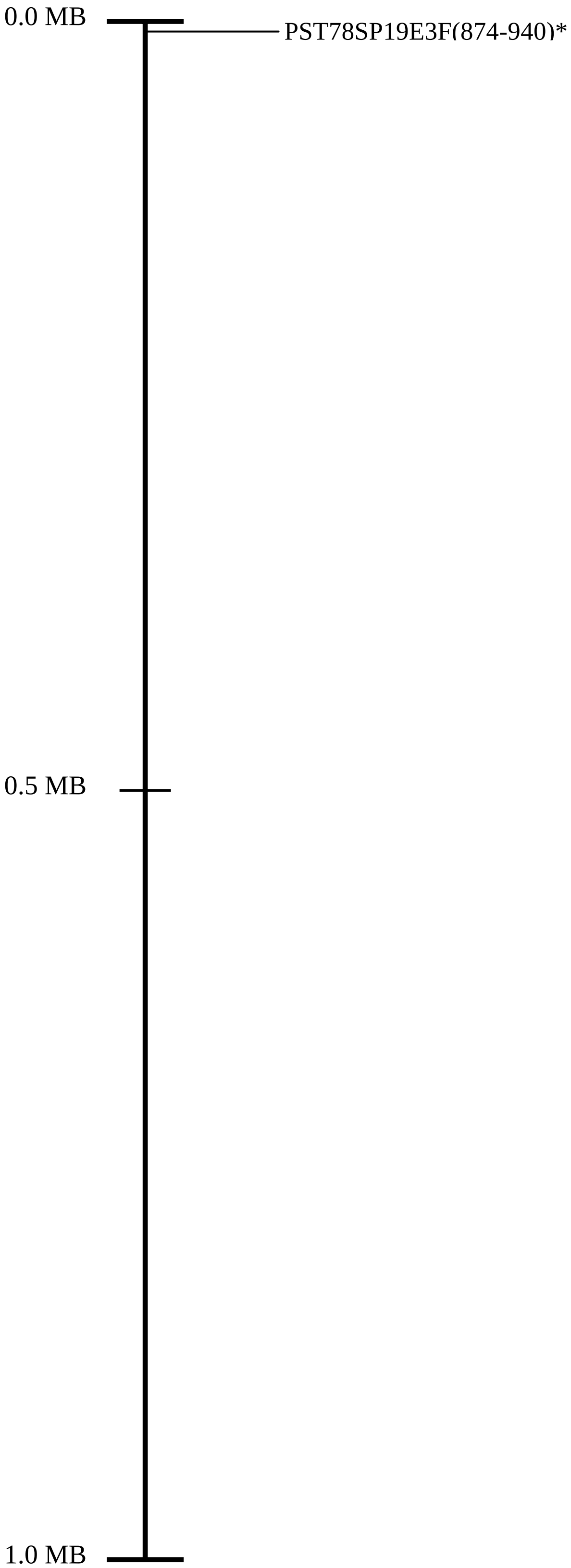

Supercontig 351
length: 5,919 bp

*** = less than 100bp alignment**

| = clones in the same contig

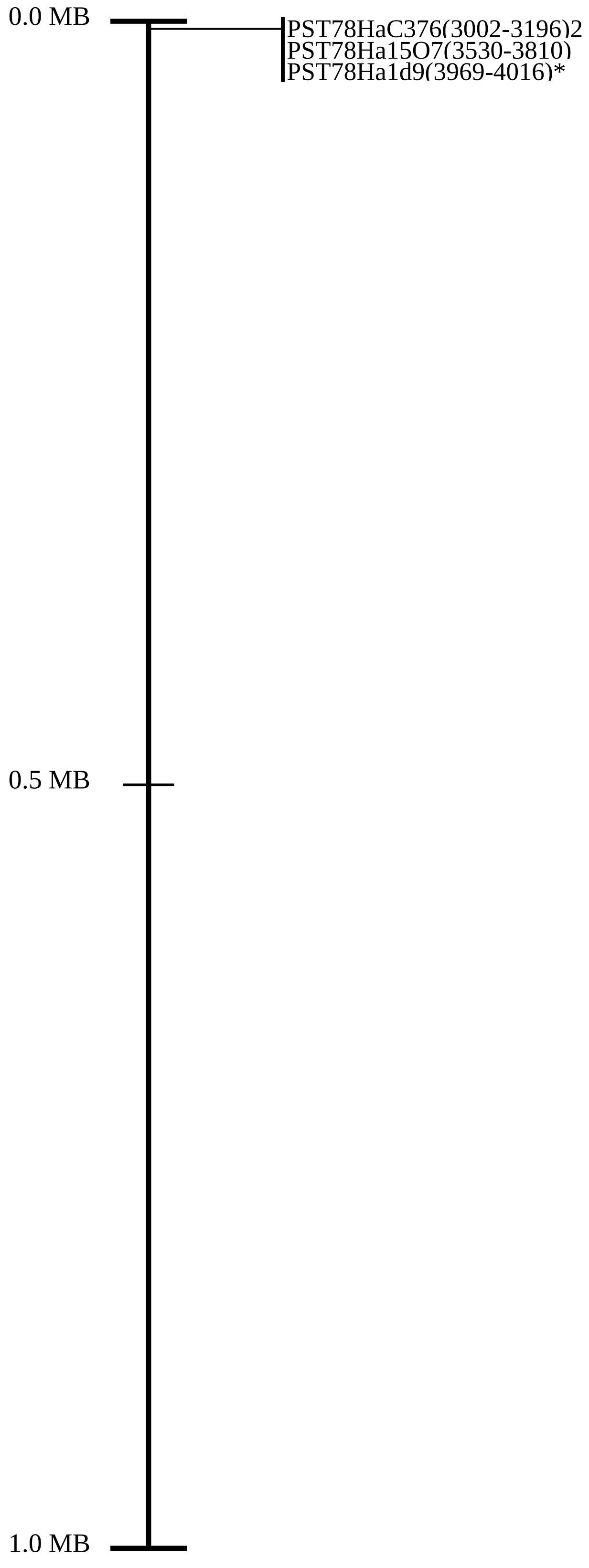

Supercontig 359
length: 5,526 bp

*** = less than 100bp alignment**

| = clones in the same contig

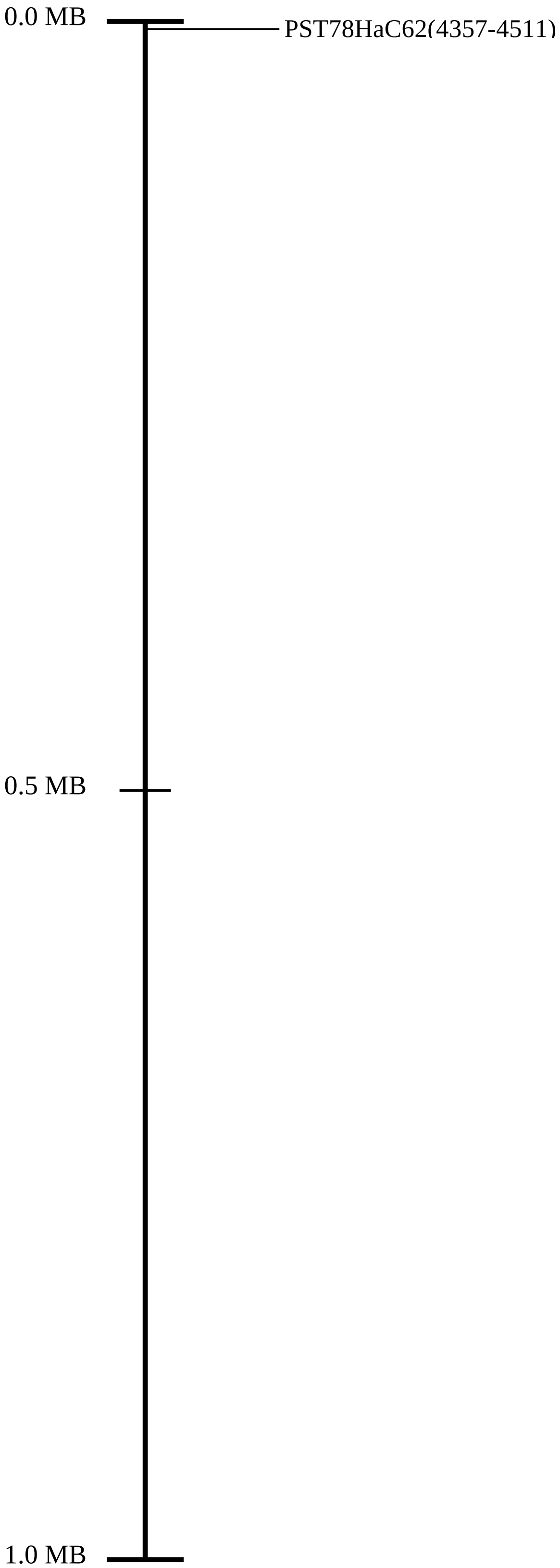

Supercontig 360
length: 5,501 bp

*** = less than 100bp alignment** **| = clones in the same contig**

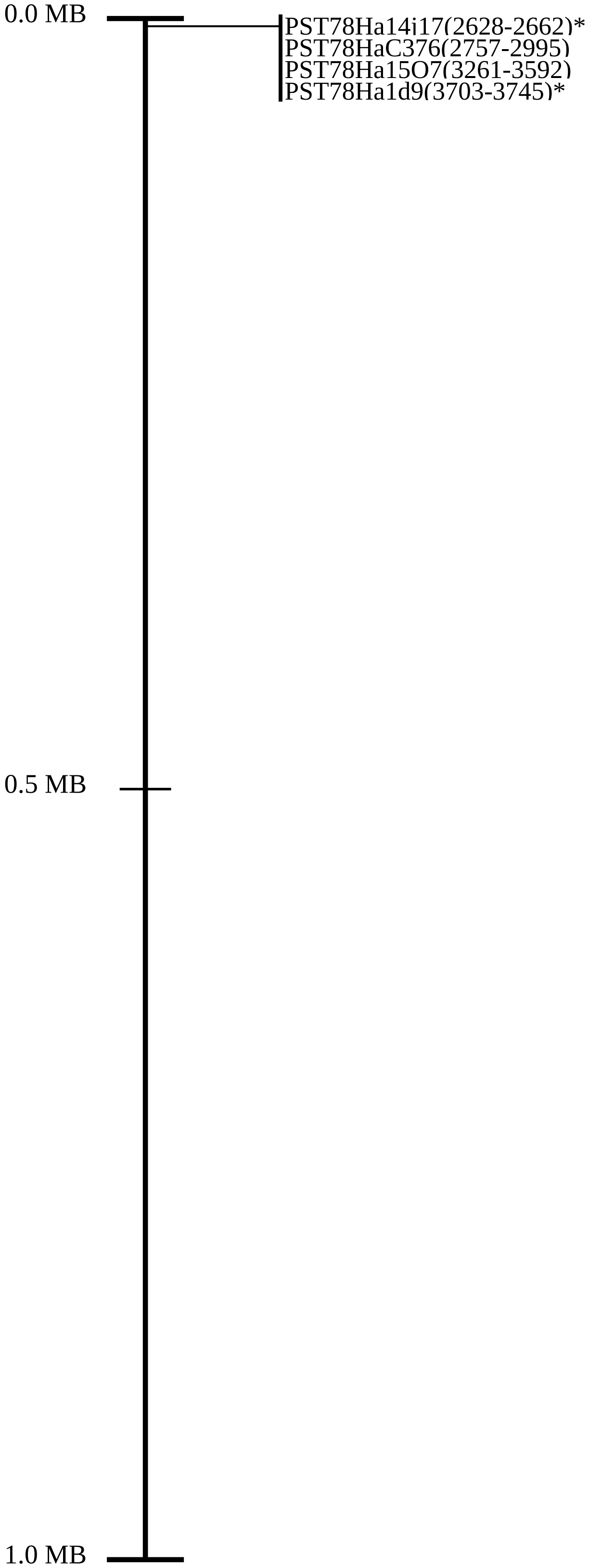

Supercontig 363
length: 5,438 bp

*** = less than 100bp alignment**

| = clones in the same contig

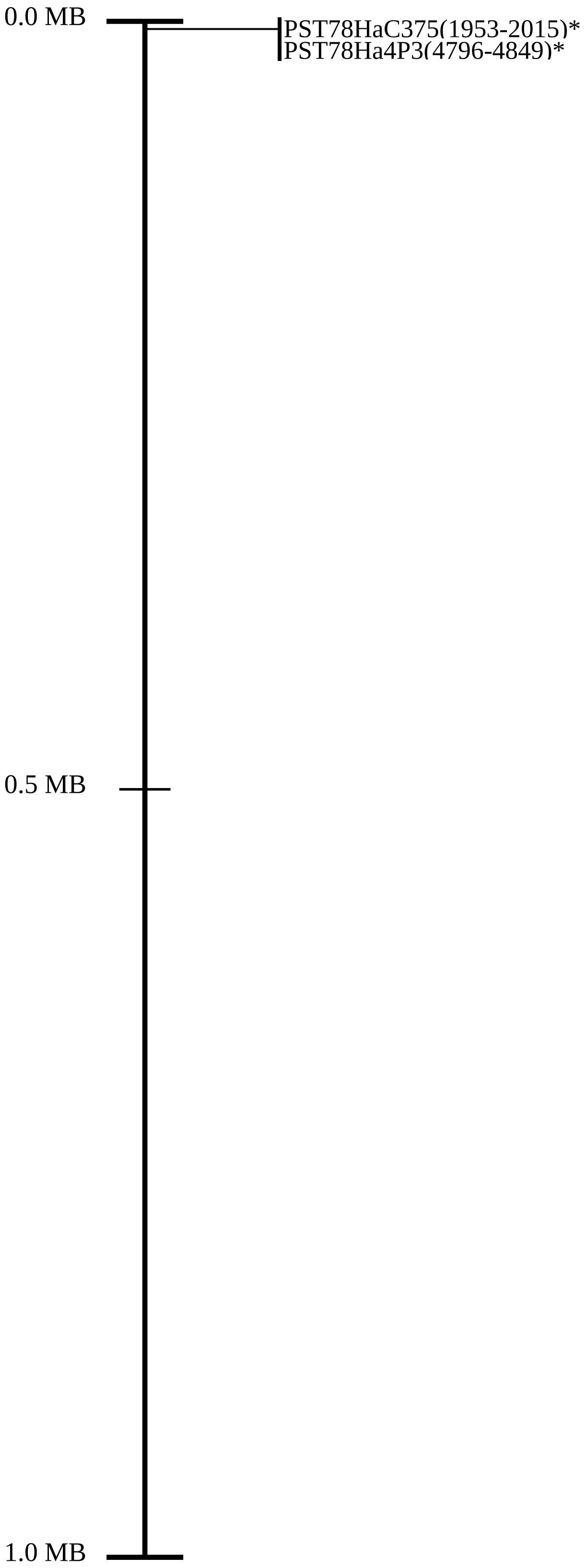

Supercontig 368
length: 5,333 bp

*** = less than 100bp alignment**

| = clones in the same contig

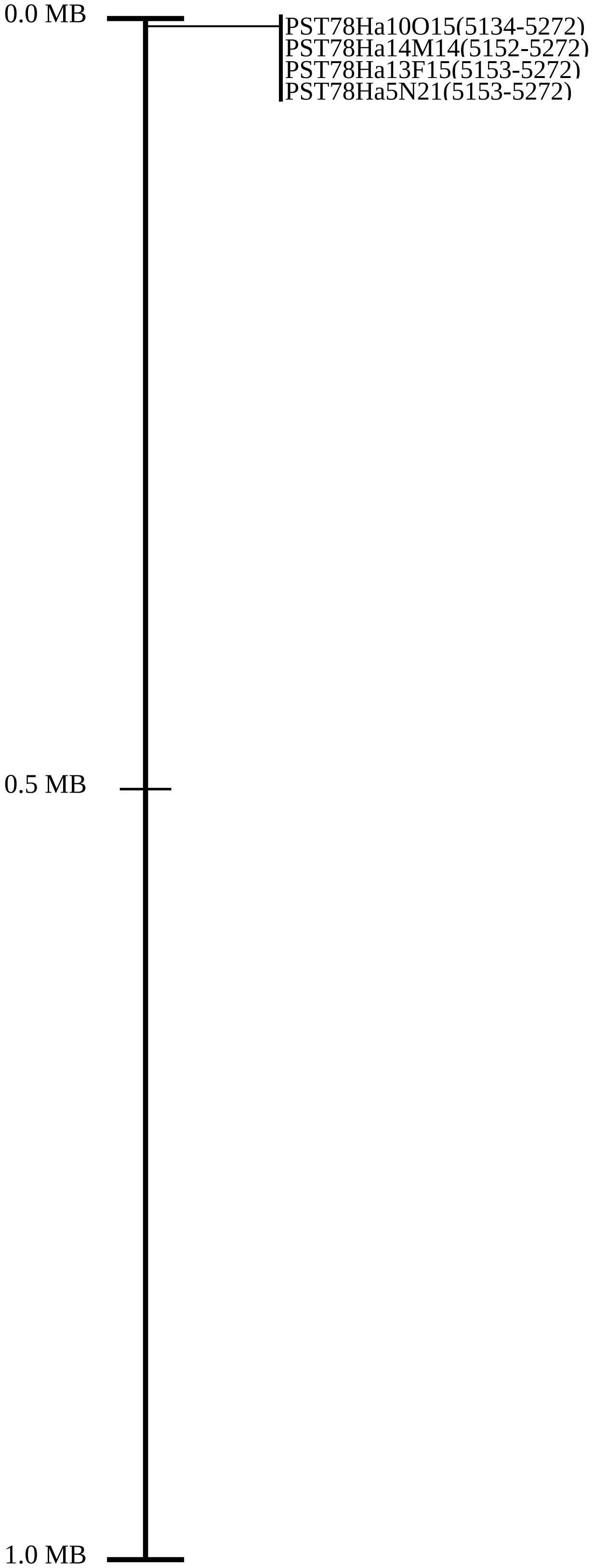

Supercontig 371
length: 5,291 bp

*** = less than 100bp alignment**

| = clones in the same contig

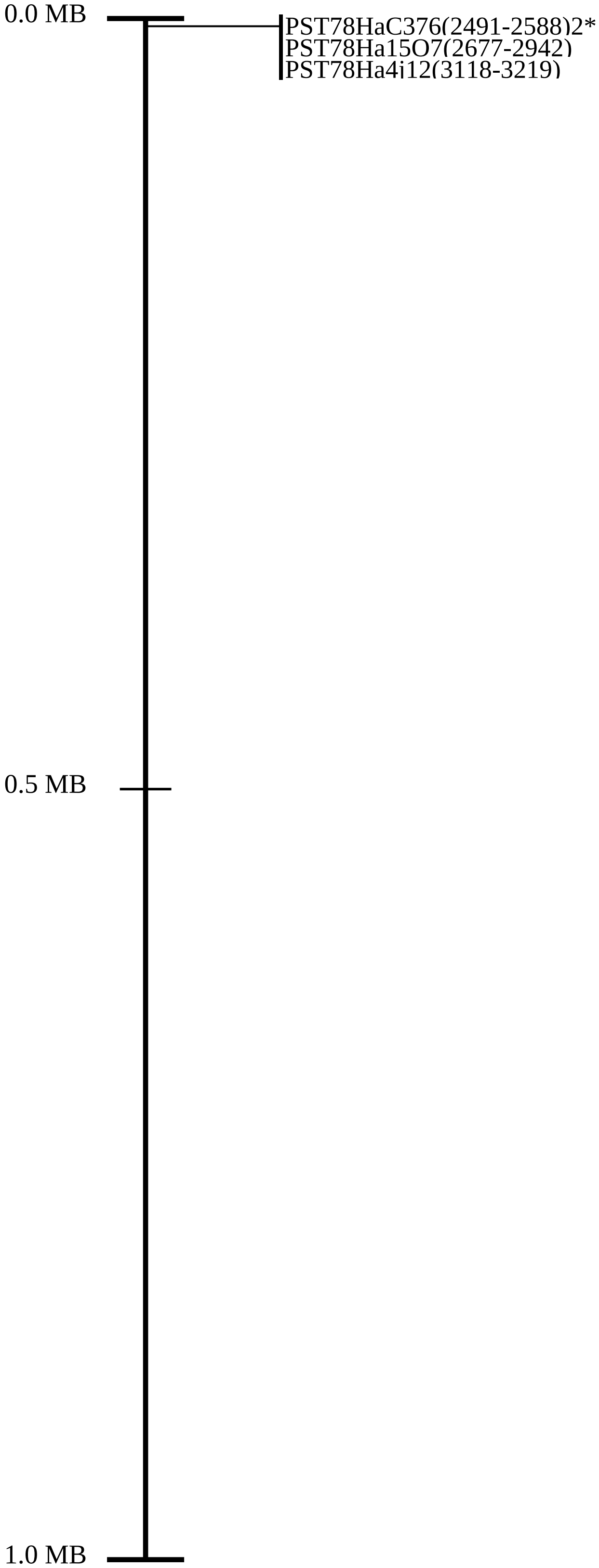

Supercontig 374
length: 5,126 bp

*** = less than 100bp alignment** **| = clones in the same contig**

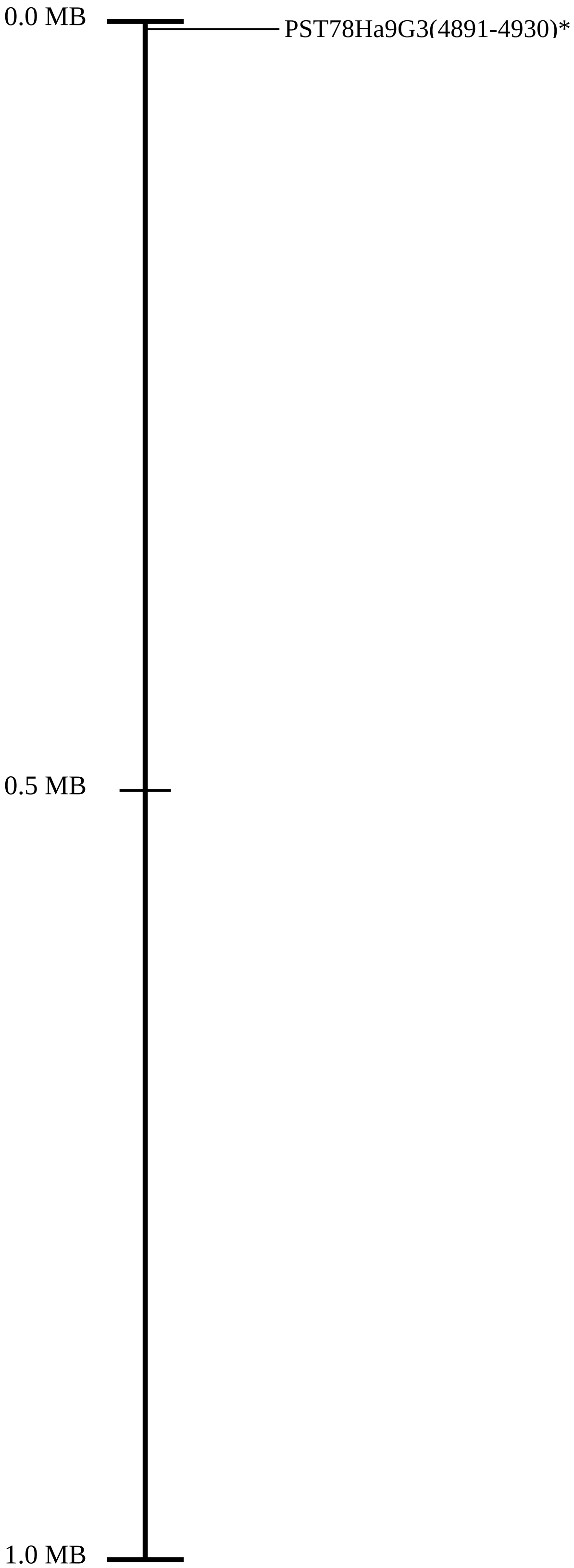

Supercontig 375
length: 5,108 bp

*** = less than 100bp alignment**

| = clones in the same contig

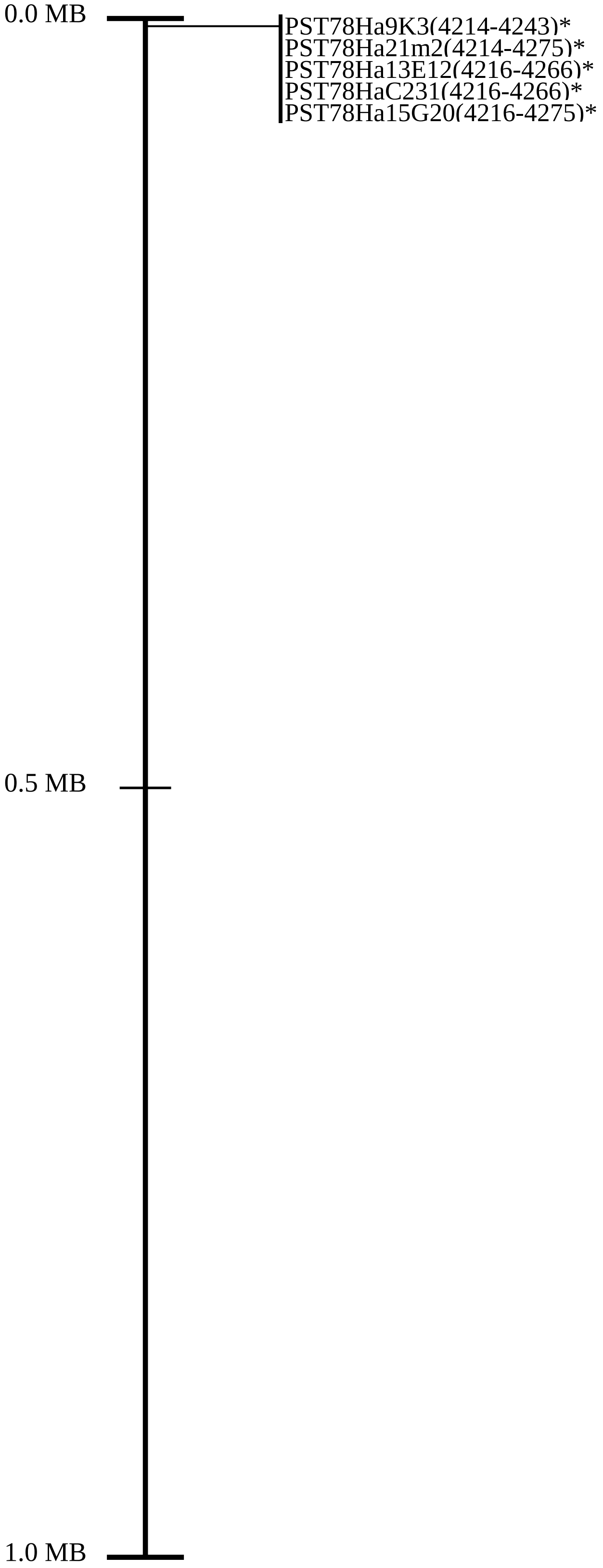

Supercontig 376
length: 5,100 bp

*** = less than 100bp alignment** **| = clones in the same contig**

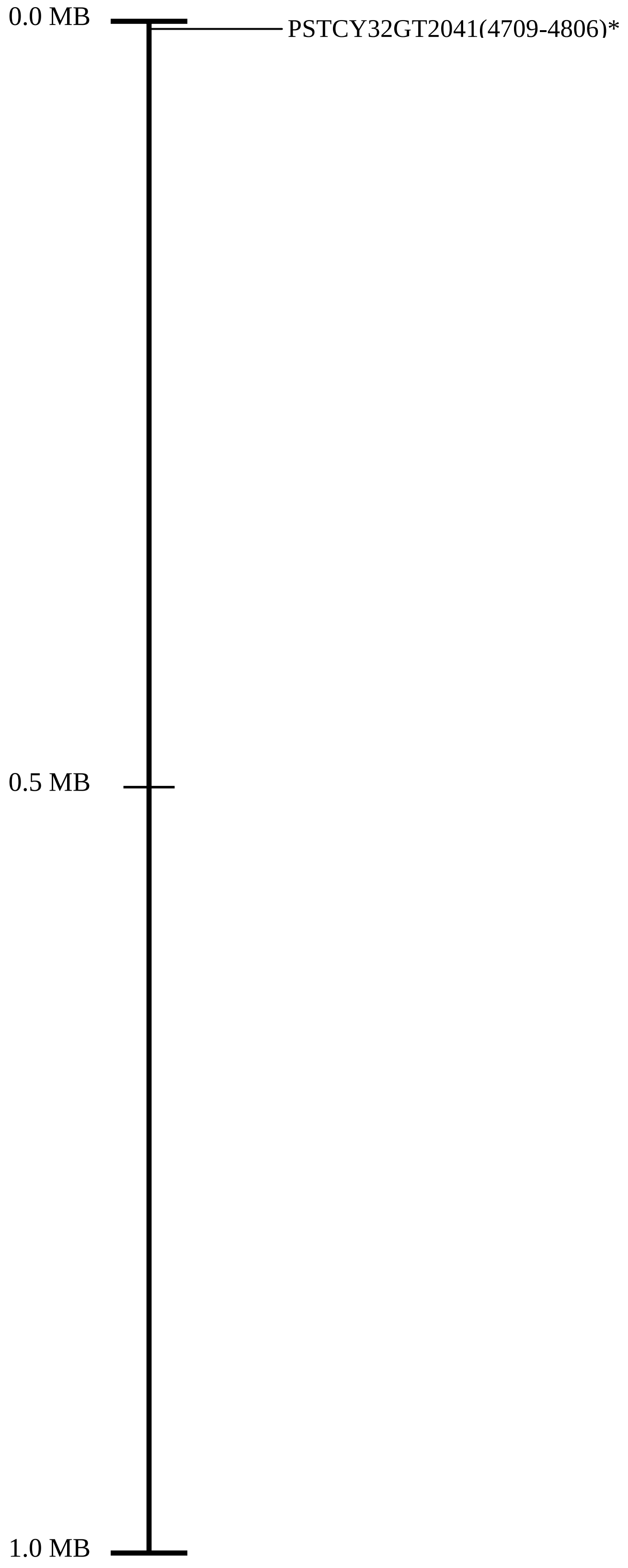

Supercontig 377
length: 5,096 bp

*** = less than 100bp alignment** **| = clones in the same contig**

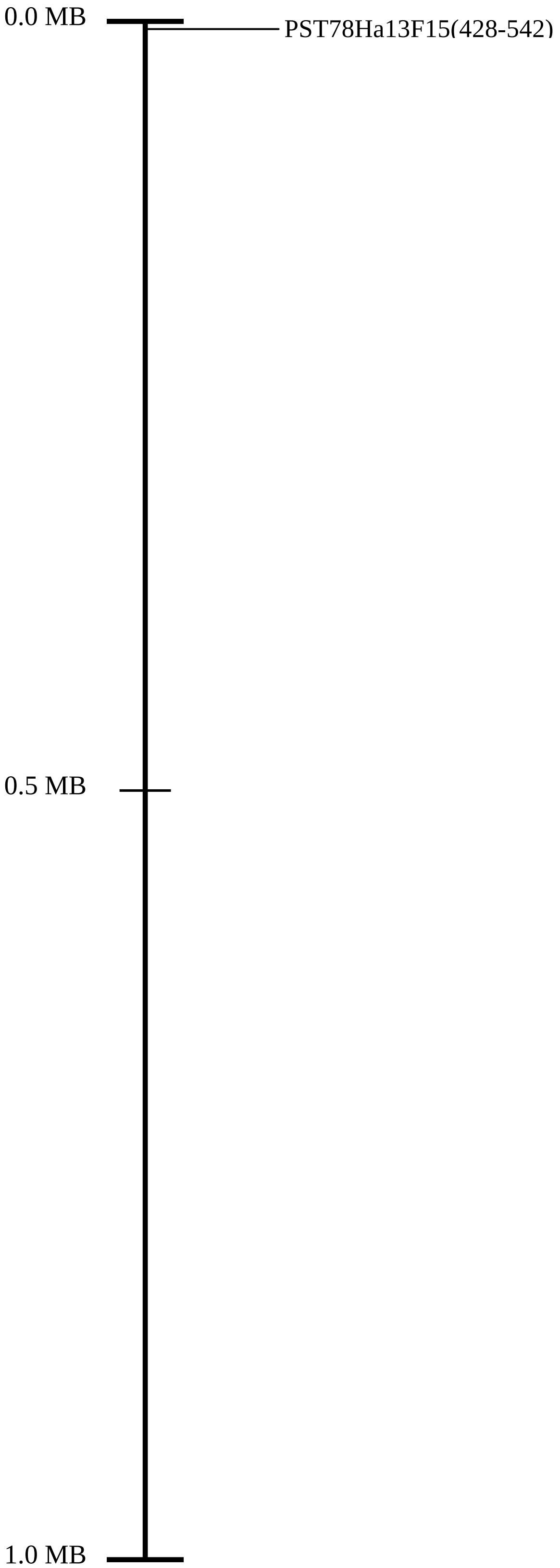

Supercontig 384
length: 4,789 bp

*** = less than 100bp alignment**

| = clones in the same contig

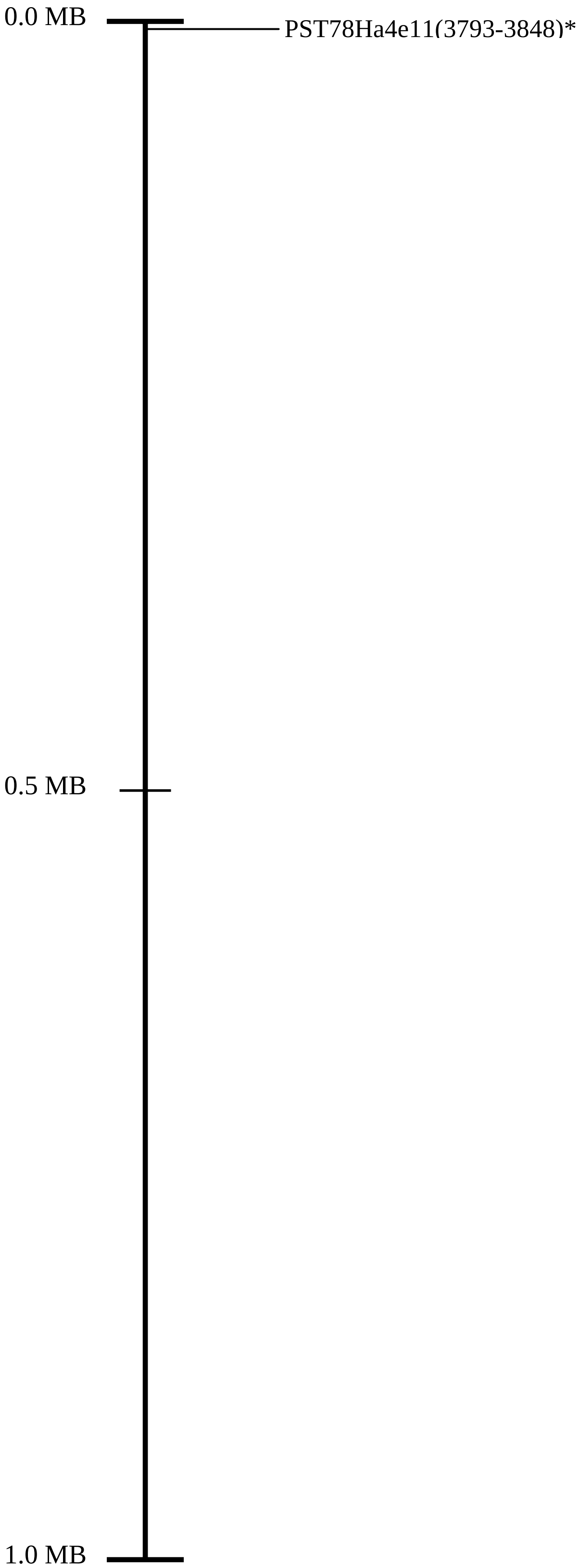

Supercontig 386
length: 4,740 bp

*** = less than 100bp alignment**

| = clones in the same contig

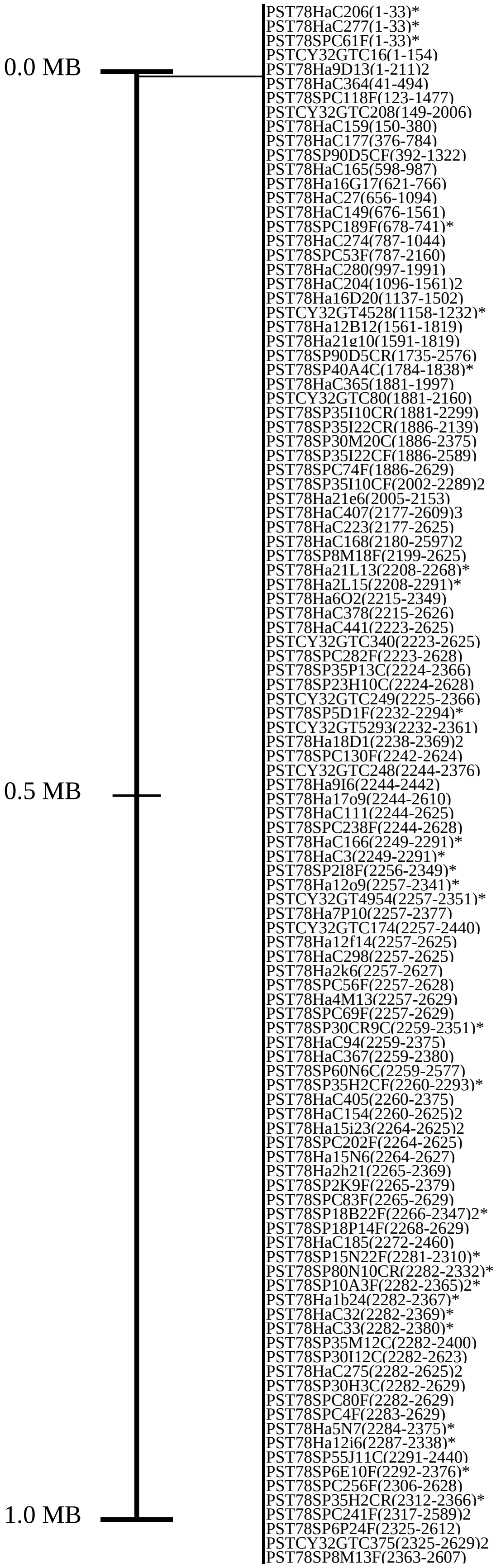

Supercontig 392
length: 2,878 bp

*** = less than 100bp alignment** **| = clones in the same contig**
